# Catalytic Asymmetric Synthesis of Unprotected β^2^-Amino Acids

**DOI:** 10.1021/jacs.1c00249

**Published:** 2021-03-01

**Authors:** Chendan Zhu, Francesca Mandrelli, Hui Zhou, Rajat Maji, Benjamin List

**Affiliations:** Max-Planck-Institut für Kohlenforschung, Kaiser-Wilhelm-Platz 1, 45470 Mülheim an der Ruhr, Germany

## Abstract

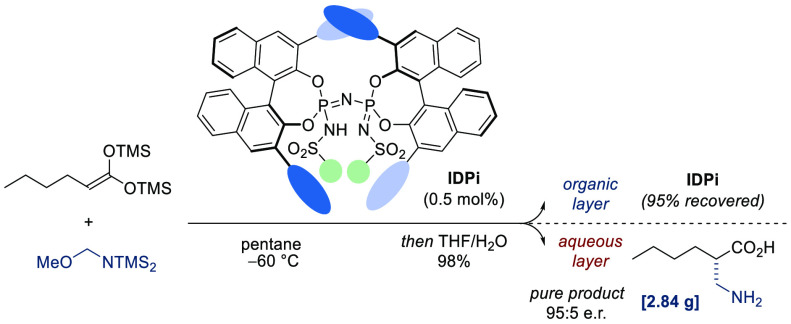

We report here a scalable, catalytic one-pot approach to enantiopure and unmodified β^2^-amino acids. A newly developed confined imidodiphosphorimidate (IDPi) catalyzes a broadly applicable reaction of diverse bis-silyl ketene acetals with a silylated aminomethyl ether, followed by hydrolytic workup, to give free β^2^-amino acids in high yields, purity, and enantioselectivity. Importantly, both aromatic and aliphatic β^2^-amino acids can be obtained using this method. Mechanistic studies are consistent with the aminomethylation to proceed via silylium-based asymmetric counteranion-directed catalysis (Si-ACDC) and a transition state to explain the enantioselectivity is suggested on the basis of density functional theory calculation.

Among the various classes of amino acids, β^2^-amino acids hold a particularly prominent place and occur in an increasing number of pharmaceuticals, natural products, and drug candidates.^1−11^ However, while chemists, in recent years, have delivered several methods toward the asymmetric synthesis of β^2^-amino acids,^12−39^ catalytic approaches that directly deliver the free, unmodified amino acid, without requiring separate redox- or protecting group manipulations, to our knowledge, have not yet been developed. Our inspirational blueprint to address this challenge is a hypothetical chiral acid catalyzed direct three-component-Mannich reaction of carboxylic acids with formaldehyde and ammonia (eq 1).
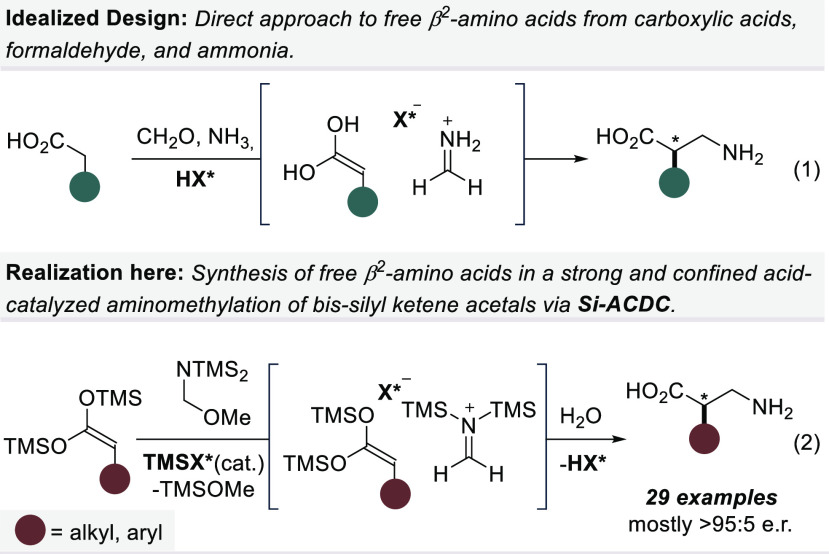
1

Unfortunately, except with malonic acid derivatives and nonenantioselectively so,^40,41^ such a “dream-reaction” has not yet been realized, arguably due to the current inability of chemists to catalytically enolize carboxylic acids.^42−44^ An attractive, even though less direct alternative would be a Mukaiyama-style reaction of preformed bis-silyl ketene acetals (bis-SKAs) with a formaldehyde imine equivalent. While this transformation has been described in a nonenantioselective fashion,^45^ asymmetric versions are entirely unknown. Encouraged by our recent studies on silylium-based asymmetric counteranion-directed catalysis (Si-ACDC),^46−66^ we envisaged to apply this approach to a TMSX*-catalyzed reaction of bis-SKAs with a silylated aminomethyl ether, followed by hydrolytic workup and extraction, which should deliver the free, unmodified β^2^-amino acids and enable a simple catalyst HX* recovery (eq 2, X*^–^ = enaniopure counteranion). Here we report on the realization of this concept with a general and highly enantioselective imidodiphosphorimidate (IDPi) catalyzed Mukaiyama Mannich-type reaction that delivers free β^2^-amino acids with either aromatic or aliphatic substituents.

We chose α-benzyl bis-SKA **1a** as our model substrate and commercially available α-aminomethyl ether **2a** as methylene imine equivalent to initiate our studies (Table 1). An initial catalyst exploration revealed that moderately acidic Brønsted acids, such as chiral phosphoric acids (CPAs),^67,68^ even upon warming, did not give any of the desired product, while imidodiphosphoric (IDP)^69^ acids promoted the reaction at 0 °C to give racemic product (see the Supporting Information). In contrast, the much more acidic IDPi catalysts provided both sufficient reactivity and promising enantioselectivity (at −40 °C in toluene). Among our IDPi libraries, spirocyclopentyl-3-fluorenyl substituted catalysts **3** turned out to be particularly promising in terms of reactivity and enantioselectivity. Extending the perfluoroalkyl sulfonyl chains in the inner core further increased the enantioselectivity (entries 1–4). With catalyst **3d**, temperature and solvent were further optimized. Lowering the temperature to −60 °C led to a slight increase in enantioselectivity (entry 5). Importantly, with pentane as the solvent instead of toluene, the enantiomeric ratio significantly increased (entry 6). Furthermore, we tested IDPi catalysts **3e**–**g**, possessing an additional substituent at the fluorenyl group (entries 7–10). Ultimately, we identified the *tert*-butyl substituted IDPi catalyst **3h** as the optimal one, giving an e.r. of 96:4 in almost quantitative yield (entry 10).

**Table 1:**
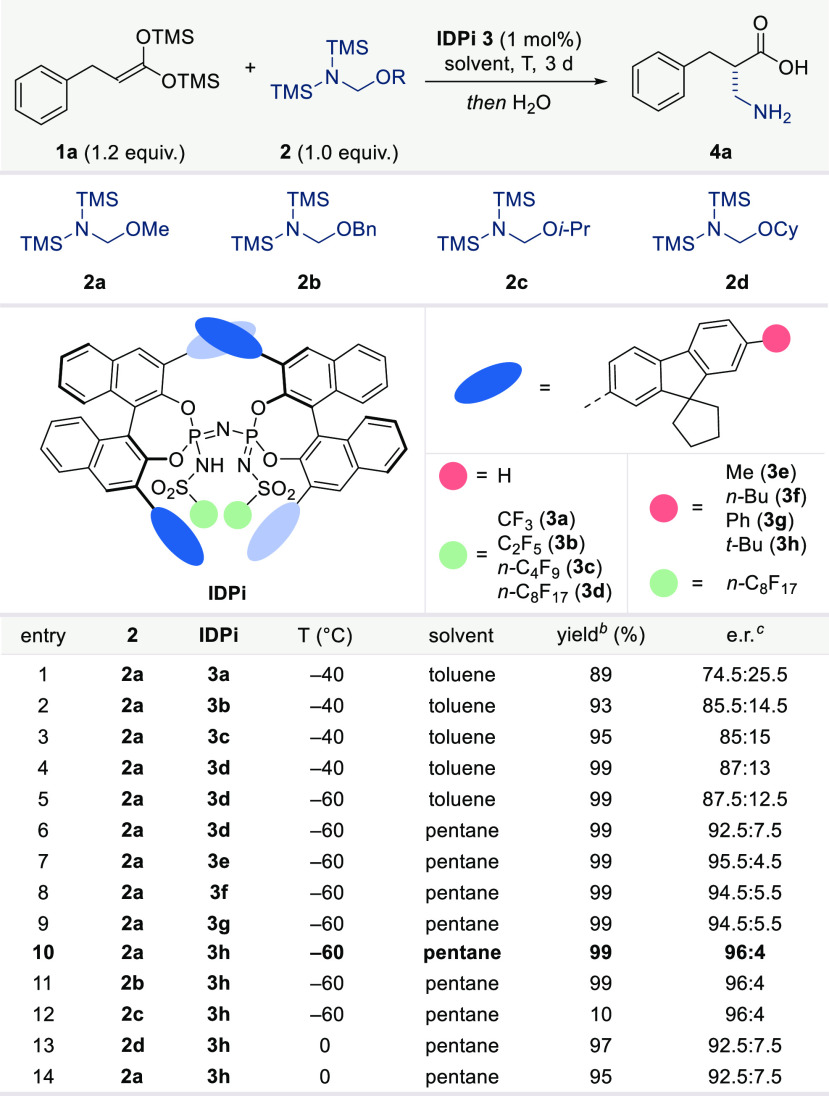
Reaction Development^a^

a Reactions were conducted on a 0.02 mmol scale: **1a:2** = 1.2:1.

b Yields were determined by ^1^H NMR using mesitylene as internal standard.

c After simplified workup, enantiomeric ratios (e.r.) were measured by HPLC. See the Supporting Information for further information.

We also studied the effect of the aminomethyl source on the conversion and stereochemical outcome (entries 11–14). Different ethers **2** with varying leaving groups were examined. Interestingly, while the alkoxy group had only an insignificant effect on the enantiocontrol, isopropyl ether **2c** gave only poor conversion at −60 °C (entries 10–12). These results are consistent with the absence of the leaving group of ether **2** in the enantiodetermining step and point toward an efficient association of the bis(silyl)iminium ion with the IDPi anion. This hypothesis could indeed be validated with a remarkably broad scope of both aromatic and aliphatic bis-SKAs (Table 2). Various free β^2^-amino acids with electronically and sterically diverse substituents were obtained in excellent yields and enantioselectivities. For example, bis-SKAs **1a**–**c** with different methylene tether lengths between a phenyl group and carboxylic acid functionality afforded the desired products in similar excellent yields and enantioselectivities. Similarly, either electron-neutral or electron-donating groups at the β-phenyl ring of the bis-SKA gave the corresponding free β^2^-amino acids in >90% yields with around 95:5 e.r. (**4d**–**e**). Notably, β^2^-amino acids with electron-withdrawing groups (F, CF_3_, Cl), either at the *ortho*-, *meta*-, or *para*-position of the β-phenyl ring were generated in >90% yield with higher enantioselectivities (>97:3 e.r.) (**4f**–**j**). Other substrates with aromatic and heteroaromatic groups, such as **1k** with naphthyl and **1l** bearing a thiophenyl substituent, were well tolerated, affording the aminomethylation products **4k** and **4l** in excellent yield and e.r..

**Table 2:**
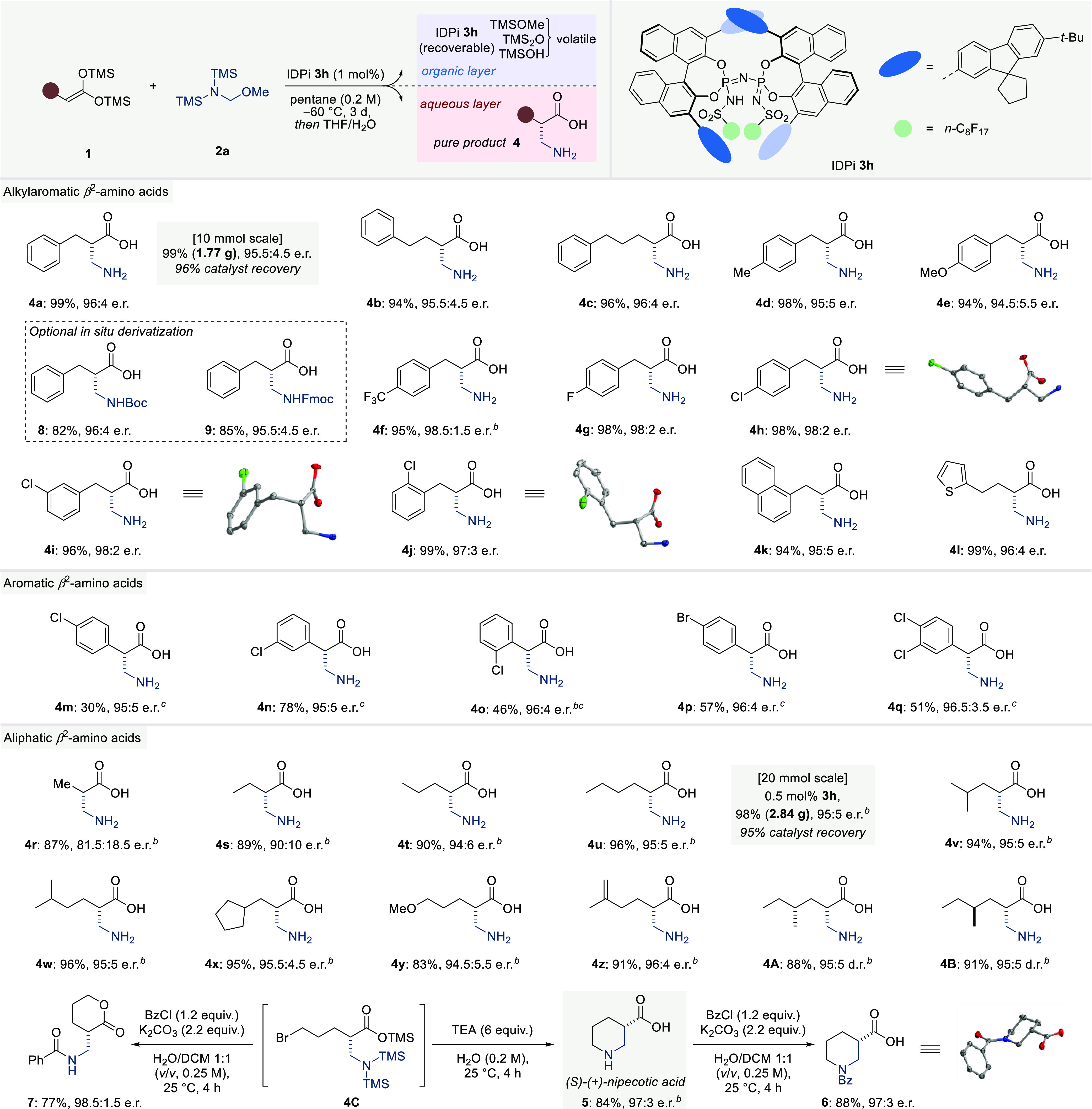
Substrate Scope^a^

a Reactions were conducted on a 0.2 mmol scale: **1**:**2a** = 1.2:1. Isolated yields with e.r. measured by HPLC. For derivatization, see Supporting Information.

b e.r. measured by HPLC after derivatization.

c 3 mol % **3h**. BzCl, benzoyl chloride; DCM, dichloromethane; TEA, triethylamine.

Directly aryl-substituted bis-SKAs **1m**–**q** were also examined and proved to be slightly less reactive, requiring 3 mol % of catalyst **3h** to furnish the corresponding products in moderate to good yields and excellent enantioselectivities.

The scope of this transformation also includes simple, aliphatic β^2^-amino acids. For example, bis-SKAs **1r**–**u**, which were generated from propionic acid, butyric acid, valeric acid, and hexanoic acid, respectively, reacted smoothly, where the enantioselectivities increased with longer alkyl chains. Branched and cyclic alkyl groups (**4v**–**x**) and a methoxy- (**4y**) and an olefin-substituted alkyl chain (**4z**) were all tolerated and provided the desired products in good to excellent yields and enantioselectivity. Interestingly, the enantiopure bis-SKA **1A** and its enantiomer *ent*-**1A** reacted to products **4A** and **4B** in good yields and, in both cases, featuring excellent and catalyst-controlled diastereoselectivity. Limitations of our method include the use of bis-silyl ketene acetals derived from α,α-disubstituted carboxylic acids and of C-substituted imine sources, which display reduced reactivity and lead to lower diastereoselectivity and enantioselectivity (see the Supporting Information).

The absolute configuration of our obtained β^2^-amino acids was determined from X-ray crystallographic analysis of products **4h**, **4i**, and **4j**. Furthermore, bromoalkyl substituted bis-SKA **1C** gave γ-aminobutyric acid uptake inhibitor (*S*)-(+)-nipecotic acid^70^**5** in a one-pot operation in 84% yield and 97:3 e.r. when treating the initial reaction product with triethylamine. The absolute configuration of amino acid **5** was determined by converting it to the corresponding benzamide **6**, crystals of which were subjected to an X-ray crystallographic analysis. ^1^H NMR investigation of the crude reaction mixture revealed the existence of silylated product **4C**, confirming that cyclization occurs only upon base treatment. In fact, oligomers were detected with concomitant formation of a small amount of compound **5** if the reaction mixture was treated with only water. Instead, treatment with benzoyl chloride and aqueous potassium carbonate enabled the access to the corresponding α-amidomethylated δ-valerolactone **7**.

The practicality of our method was illustrated with two scale-up experiments, involving an extremely concise product purification and catalyst recovery. Using 1 mol % of catalyst **3h**, 12 mmol of bis-SKA **1a** and 10 mmol of imine precursor **2a** gave 1.77 g of the free β^2^-amino acid **4a** in 99% isolated yield with an e.r. of 95.5:4.5. The workup of the reaction mixture included a simple extraction with water and washing with dichloromethane without further purification. Gratifyingly, catalyst **3h** could be easily recovered in 96% yield from the organic phase via flash chromatography and acidification. Similarly, 2.84 g of the aliphatic free β^2^-amino acid **4u** was obtained in 98% isolated yield with an e.r. of 95:5 from 20 mmol of reagent **2a** using only 0.5 mol % of catalyst **3h**, which was recovered in 95% yield from the organic phase after flash chromatography and acidification.

Optionally, the crude products can be readily derivatized in situ into a variety of synthetically useful building blocks such as the corresponding *N*-Boc- or *N*-Fmoc-protected β^2^-amino acids **8** and **9** by treating the reaction mixture with an appropriate derivatization reagent.

On the basis of the observation that the alkyl group of ethers **2** had an insignificant effect on the enantioselectivity (Table 1, entries 11–14), coupled with literature results,^45,58−66^ we envision a catalytic cycle as shown in Figure 1a. Accordingly, the reaction commences with the in situ silylation of the IDPi catalyst **3** by bis-SKA **1** to furnish the *N*-silylated catalyst **I** and/or its diastereomeric O–Si-silatropomers.^58−66^ α-Aminomethyl ether **2** then reacts with catalyst **I**, generating the methylene iminium ion-IDPi anion pair **II**, simultaneously liberating TMSOMe.^45^ Subsequently, bis-SKA **1** reacts with the cationic methylene iminium ion in the anionic catalyst pocket to give ion pair **III**. Intra-ion-pair silyl transfer from the cationic product back onto its counteranion then furnishes the silylated product **IV** and re-establishes the silylated catalyst **I**. Finally, hydrolytic workup and extraction of the reaction mixture delivers the free β^2^-amino acid **4**. On the basis of a detailed conformational search and subsequent Density Functional Theory (DFT) optimization of ion pair **II**, we tentatively propose a sterical hindrance-based selectivity model (Figure 1b), where *re*-facial addition of bis-SKA **1** to methylene iminium-IDPi anion pair **II** leads to the observed enantiomer (see the Supporting Information).

**Figure 1:**
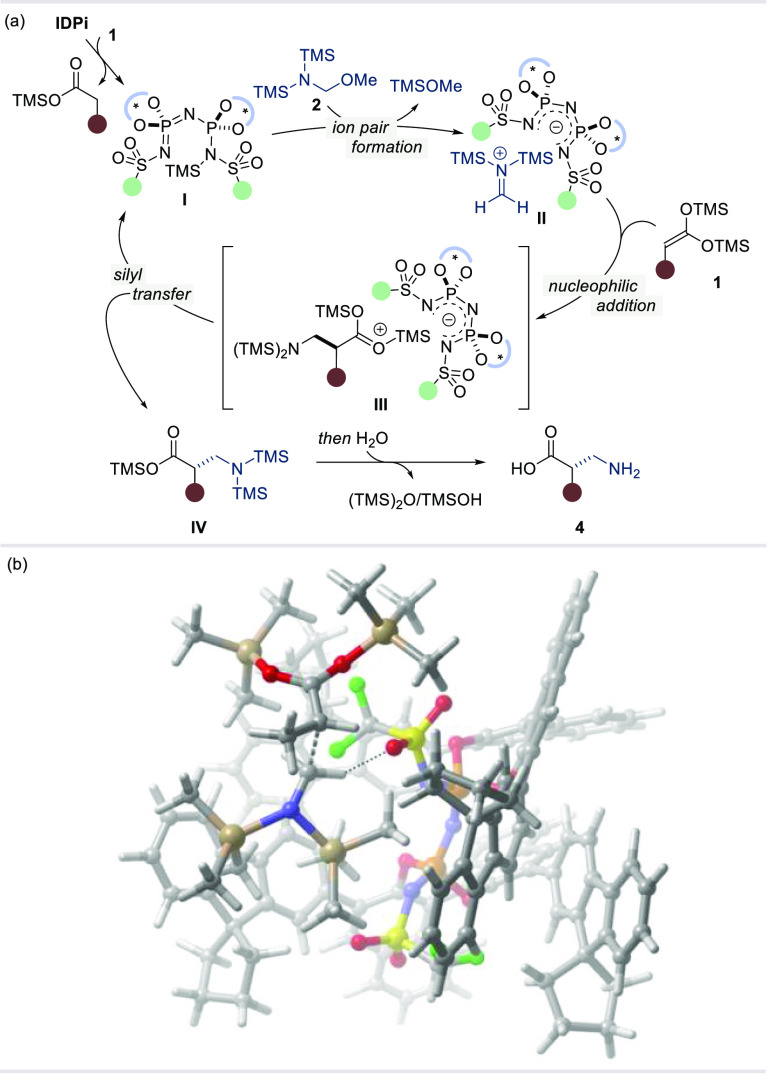
(a) Proposed catalytic cycle. (b) Suggested *re*-facial approach of the SKA onto the DFT optimized iminium-IDPi ion pair II.

We have developed a traceless and scalable approach to enantiopure free β^2^-amino acids via catalytic asymmetric aminomethylation of bis-silyl ketene acetals. A variety of aromatic and aliphatic bis-SKAs from carboxylic acids with diverse electronics and sterics were tolerated in this transformation and provided the corresponding amino acids in excellent yields and enantioselectivities. The purification process is extremely simple and concise and enables catalyst recovery. We conducted control experiments that are consistent with a mechanism that proceeds via Si-ACDC, while preliminary computational studies suggest steric effects to cause the observed enantioselectivity. As IDPi catalysts are currently being commercialized, the methodology reported here may facilitate the synthesis of pharmaceuticals, natural products, and peptidic foldamers.

## Supporting Information Available

Experimental details and analytical data for all new compounds, crystallographic data for compounds **4h**, **4i**, and **4j**, HPLC traces, NMR spectra, computational studies, optimized structures, and Cartesian coordinates (PDF)

## Accession Codes

CCDC 2056835–2056839 contain the supplementary crystallographic data for this paper. These data can be obtained free of charge via www.ccdc.cam.ac.uk/data_request/cif, or by emailing data_request@ccdc.cam.ac.uk, or by contacting The Cambridge Crystallographic Data Centre, 12 Union Road, Cambridge CB2 1EZ, UK; fax: +44 1223 336033.
