# Supplementary material for: Catalytic Asymmetric Synthesis of Unprotected β2-Amino Acids
Source: J Am Chem Soc. 2021 Mar 1;143(9):3312–7. doi: 10.1021/jacs.1c00249 (PMC7953379; doi:10.1021/jacs.1c00249)
Supplement: Supplementary file 1 — ja1c00249_si_001.pdf [file ja1c00249_si_001.pdf]

Catalytic Asymmetric Synthesis of Unprotected β^2-Amino Acids

(Supporting Information)

Chendan Zhu, Francesca Mandrelli, Hui Zhou, Rajat Maji, and Benjamin List*
Max-Planck-Institut für Kohlenforschung, Kaiser-Wilhelm-Platz 1, 45470 Mülheim an der Ruhr, Germany
*Email: list@kofo.mpg.de

Table of Contents

1. General Information and Instrumentation 2
2. Synthesis and Characterization of bis-Silyl Ketene Acetals 4
3. Synthesis and Characterization of Silylated Aminomethyl Ethers 13
4. Procedure for Reaction Development 15
5. Enantioselective Aminomethylation and Derivatization of Product 17
6. Synthesis and Characterization of Catalysts 37
7. References 46
8. Crystallographic Data 47
9. HPLC Traces of the Products 58
10. ^{1}H , ^{13}C, ^{31}P and ^{19}F NMR spectra of substrates and products 93
11. Computational Studies 190

1. General Information and Instrumentation

Chemicals: Unless otherwise indicated, starting materials were obtained from Sigma-Aldrich, ABCR-GmbH, TCI, or Acros Co. Ltd. Commercially available reagents were used without additional purification. The chiral imidodiphosphorimidate acids (IDPis) **3a–d**[1] were synthesized according to literature procedures.

Solvents: Solvents (Et_2O, THF, 1,4-Dioxane, Cyclohexane, CH_2Cl_2, $CHCl_3$, Benzene and Toluene) were dried by distillation from an appropriate drying agent in the technical department of the Max-Planck-Institut für Kohlenforschung and received in Schlenk flasks under argon. In addition, more solvents (MTBE, MeCN and Mesitylene) were purchased from commercial suppliers and dried over molecular sieves.

Inert Gas: Dry argon was purchased from Air Liquide with >99.5% purity.

Thin Layer Chromatography: Thin-layer chromatography (TLC) was performed using silica gel pre-coated plastic sheets (Polygram SIL G/UV_{254}, 0.2 mm, with fluorescent indicator; Macherey-Nagel) which was visualized with a UV lamp (254 nm) and/or phosphomolybdic acid (PMA), and/or Cerium Ammonium Molybdate (CAM), and/or ninhydrin. PMA stain: PMA (20 g) in EtOH (200 mL). CAM stain: Ammonium molybdate tetrahydrate (2.5 g), Cerium ammonium sulfate dihydrate (1 g) and Sulfuric acid (10 mL) in Water (90 mL). Ninhydrin stain: ninhydrin (1.5 g) in EtOH (200 mL) with AcOH (3 mL).

Column Chromatography: Column chromatography was carried out using Merck silica gel (60 Å, 230−400 mesh, particle size 0.040−0.063 mm) using technical grade solvents. Elution was accelerated using compressed argon. All reported yields, unless otherwise specified, refer to spectroscopically and chromatographically pure compounds.

Nomenclature: Nomenclature follows the suggestions proposed by the computer program ChemBioDraw (12.0.3.1216) of CBD/cambridgesoft.

Nuclear Magnetic Resonance Spectroscopy: ^{1}H, ^{13}C, ^{19}F, ^{31}P Nuclear magnetic resonance (NMR) spectra for compound characterization were recorded on Bruker AVIII-500 MHz, NMR spectrometer in a suitable deuterated solvent. The solvent employed and the respective measuring frequency are indicated for each experiment. Chemical shifts are reported with tetramethylsilane (TMS) serving as a universal reference of all nuclides. The resonance multiplicity is described as

s (singlet), d (doublet), t (triplet), q (quadruplet), p (pentet), h (heptet), m (multiplet), and br (broad). All spectra were recorded at 298 K, processed with MestReNova 14.1.2 suits of program, and coupling constants are reported as observed. The residual deuterated solvent signal relative to tetramethylsilane was used as the internal reference in ^{1}H NMR spectra (e.g. $CDCl_3$ = 7.26 ppm, CD_2Cl_2 = 5.32 ppm). Signals are reported as follows: chemical shift δ in ppm (multiplicity, coupling constant *J* in Hz, number of protons). All X-nuclei spectra were acquired proton decoupled unless otherwise noted.

Mass Spectrometry: Electrospray ionization (ESI) mass spectrometry was conducted on a Bruker ESQ 3000 spectrometer. High resolution mass spectrometry (HRMS) was performed on a Finnigan MAT 95 (EI) or Bruker APEX III FTMS (7 T magnet, ESI). The ionization method and mode of detection employed is indicated for the respective experiment. The ionization method and mode of detection employed is indicated for the respective experiment and all masses are reported in atomic units per elementary charge (*m/z*) with an intensity normalized to the most intense peak.

Specific Rotations: Specific rotations ($[\alpha]_D^t$) were measured with a Rudolph RA Autopol IV Automatic Polarimeter at the indicated temperature with a sodium lamp (sodium D line, λ = 589 nm). Measurements were performed in an acid resistant 1 mL cell (50 mm length) with concentrations (g/(100 mL)) reported in the corresponding solvent.

High Performance Liquid Chromatography: High performance liquid chromatography (HPLC) was performed on a Shimadzu LC-20AD liquid chromatograph SIL-20AC auto sampler, CMB-20A using Daicel/Merck columns with a chiral stationary phase. All solvents used were HPLC-grade solvents purchased from Sigma-Aldrich. The column employed and the respective solvent mixture are indicated for each experiment.

Abbreviations: e.r. = enantiomeric ratio, TLC = thin layer chromatography, THF = tetrahydrofuran, MTBE = methyl *tert*-butyl ether, MeCN = acetonitrile, Mesitylene = 1,3,5-trimethylbenzene, TBS = $SiMe_2{}^tBu$, TMS = $SiMe_3$, TES = triethylsilyl, Tf = SO_2CF_3, MOM = methoxymethyl ether.

2. Synthesis and Characterization of bis-Silyl Ketene Acetals

General Procedure:

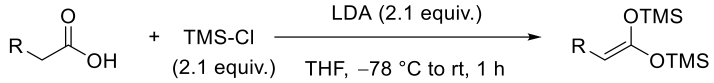

Preparation of lithium diisopropyl amine (LDA) solution: To a stirring solution of freshly distilled diisopropyl amine (21 mmol, 3.0 mL, 2.1 equiv.) in THF (10 mL) under an atmosphere of argon, *n*-BuLi (2.5 M solution in hexane, 21 mmol, 8.4 mL, 2.1 equiv.) was added dropwise at –78 °C and the resulting reaction mixture was stirred for additional 30 min at rt.

A stirring solution of acid (10 mmol, 1.0 equiv.) and TMSCl (2.6 mL, 21 mmol, 2.1 equiv.) in THF (5 mL) under an atmosphere of argon was cooled to –78 °C. The LDA solution was added via cannula. The cooling bath was removed after addition and the reaction solution was stirred for 1 h at rt. The reaction mixture was concentrated in vacuo, and then dry hexane (20 mL) was added to the residue and the mixture was filtered under an atmosphere of argon. After concentration under reduced pressure, the crude product was purified by distillation under reduced pressure to afford the bis-silyl ketene acetal as colorless liquid, which was stored in a Schlenk flask under an atmosphere of argon at 4 °C or –20 °C.

2,2,6,6-Tetramethyl-4-(2-phenylethylidene)-3,5-dioxa-2,6-disilaheptane (1a)

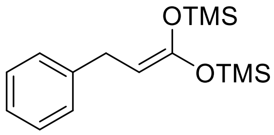

Prepared according to the general procedure using 3-phenylpropanoic acid (1.50 g, 10 mmol) and obtained after distillation (bp = 70 °C at $3.2*10^{-2}$ mbar) as colorless liquid (2.44 g, 83% yield). **^{1}H NMR (501 MHz, CD_2Cl_2)** δ 7.27–7.23 (m, 2H), 7.21–7.18 (m, 2H), 7.17–7.11 (m, 1H), 3.77 (t, *J* = 7.3 Hz, 1H), 3.27 (d, *J* = 7.3 Hz, 2H), 0.24 (s, 9H), 0.21 (s, 9H). **^{13}C NMR (126 MHz, CD_2Cl_2)** δ 151.9, 143.9, 128.7, 128.6, 125.9, 82.3, 31.9, 0.7, 0.1. **HRMS m/z (EI):** calculated for $C_{15}H_{26}O_2Si_2$ $[M]^+$: 294.1466, found 294.1463.

2,2,6,6-Tetramethyl-4-(3-phenylpropylidene)-3,5-dioxa-2,6-disilaheptane (1b)

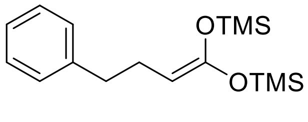

Prepared according to the general procedure using 4-phenylbutanoic acid (1.64 g, 10 mmol) and obtained after distillation (bp = 87 °C at

7.8*10^{-2} mbar) as colorless liquid (2.28 g, 74% yield). **^{1}H NMR (501 MHz, CD_2Cl_2)** δ 7.28–7.22 (m, 2H), 7.20–7.11 (m, 3H), 3.55 (t, *J* = 7.1 Hz, 1H), 2.63–2.55 (m, 2H), 2.25–2.17 (m, 2H), 0.19 (s, 9H), 0.17 (s, 9H). **^{13}C NMR (126 MHz, CD_2Cl_2)** δ 151.3, 143.4, 129.1, 128.6, 126.0, 82.6, 37.5, 27.8, 0.7, 0.0. **HRMS m/z (EI):** calculated for $C_{16}H_{29}O_2Si_2$ [M+H]$^{+}$: 309.1701, found 309.1699.

2,2,6,6-Tetramethyl-4-(4-phenylbutylidene)-3,5-dioxa-2,6-disilaheptane (1c)

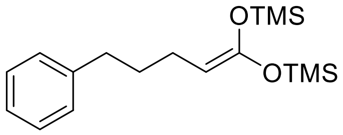

Prepared according to the general procedure using 5-phenylpentanoic acid (1.78 g, 10 mmol) and obtained after distillation (bp = 84 °C at 1.6*10^{-2} mbar) as colorless liquid (2.18 g, 67% yield). **^{1}H NMR (501 MHz, CD_2Cl_2)** δ 7.28–7.22 (m, 2H), 7.20–7.12 (m, 3H), 3.57 (t, *J* = 7.1 Hz, 1H), 2.62–2.55 (m, 2H), 1.94 (q, *J* = 7.2 Hz, 2H), 1.60 (p, *J* = 7.4 Hz, 2H), 0.23 (s, 9H), 0.18 (s, 9H). **^{13}C NMR (126 MHz, CD_2Cl_2)** δ 151.2, 143.8, 129.0, 128.7, 126.0, 83.3, 36.0, 33.3, 25.3, 0.7, 0.1. **HRMS m/z (EI):** calculated for $C_{17}H_{30}O_2Si_2$ [M]$^{+}$: 322.1779, found 322.1774.

2,2,6,6-Tetramethyl-4-(2-(p-tolyl)ethylidene)-3,5-dioxa-2,6-disilaheptane (1d)

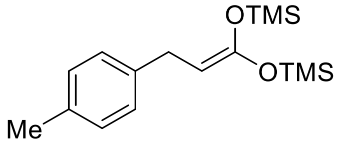

Prepared according to the general procedure using 3-(*p*-tolyl)propanoic acid (1.64 g, 10 mmol) and obtained after distillation (bp = 87 °C at 6.2*10^{-2} mbar) as colorless liquid (1.60 g, 77% yield). **^{1}H NMR (501 MHz, CD_2Cl_2)** δ 7.11–7.02 (m, 4H), 3.74 (t, *J* = 7.3 Hz, 1H), 3.22 (d, *J* = 7.3 Hz, 2H), 2.29 (s, 3H), 0.23 (s, 9H), 0.21 (s, 9H). **^{13}C NMR (126 MHz, CD_2Cl_2)** δ 151.7, 140.7, 135.4, 129.3, 128.5, 82.6, 31.4, 21.2, 0.7, 0.1. **HRMS m/z (EI):** calculated for $C_{16}H_{28}O_2Si_2$ [M]$^{+}$: 308.1622, found 308.1622.

4-(2-(4-Methoxyphenyl)ethylidene)-2,2,6,6-tetramethyl-3,5-dioxa-2,6-disilaheptane (1e)

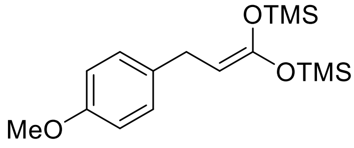

Prepared according to the general procedure using 3-(4-methoxyphenyl)propanoic acid (1.80 g, 10 mmol) and obtained after distillation (bp = 95 °C at 6*10^{-3} mbar) as colorless liquid (2.20 g, 68% yield). **^{1}H NMR (501 MHz, CD_2Cl_2)** δ 7.12–7.08 (m, 2H), 6.81–6.77 (m, 2H), 3.76 (s, 3H), 3.75–3.72 (m, 1H), 3.20 (d, *J* = 7.3 Hz, 2H), 0.23 (s, 9H), 0.21 (s, 9H). **^{13}C**

NMR (126 MHz, CD_2Cl_2)** δ 158.2, 151.7, 135.9, 129.4, 114.0, 82.8, 55.7, 30.9, 0.7, 0.1. **HRMS m/z (EI):** calculated for $C_{16}H_{28}O_3Si_2$ $[M]^+$: 324.1572, found 324.1568.

2,2,6,6-Tetramethyl-4-(2-(4-(trifluoromethyl)phenyl)ethylidene)-3,5-dioxa-2,6-disilaheptane (1f)

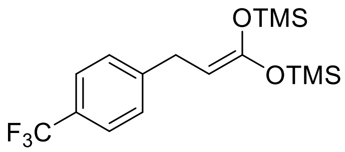

Prepared according to the general procedure using 3-(4-(trifluoromethyl)phenyl)propanoic acid (2.18 g, 10 mmol) and obtained after distillation (bp = 67 °C at $1.0*10^{-2}$ mbar) as colorless liquid (3.08 g, 85% yield). **^{1}H NMR (501 MHz, CD_2Cl_2)** δ 7.52 (d, *J* = 8.0 Hz, 2H), 7.33 (d, *J* = 8.0 Hz, 2H), 3.75 (t, *J* = 7.3 Hz, 1H), 3.33 (d, *J* = 7.3 Hz, 2H), 0.24 (s, 9H), 0.20 (s, 9H). **^{13}C NMR (126 MHz, CD_2Cl_2)** δ 152.5, 148.5, 129.3, 129.0, 125.5 (q, *J* = 3.8 Hz), 81.0, 31.8, 0.7, 0.1. (other signals not detected or observed). **^{19}F NMR (471 MHz, CD_2Cl_2)** δ –62.47. **HRMS m/z (EI):** calculated for $C_{16}H_{25}O_2Si_2F_3$ $[M]^+$: 362.1345, found 362.1342.

4-(2-(4-Fluorophenyl)ethylidene)-2,2,6,6-tetramethyl-3,5-dioxa-2,6-disilaheptane (1g)

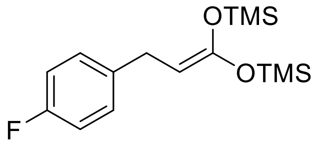

Prepared according to the general procedure using 3-(4-fluorophenyl)propanoic acid (1.68 g, 10 mmol) and obtained after distillation (bp = 77 °C at $2.6*10^{-2}$ mbar) as colorless liquid (2.46 g, 79% yield). **^{1}H NMR (501 MHz, CD_2Cl_2)** δ 7.19–7.13 (m, 2H), 6.99–6.89 (m, 2H), 3.74 (t, *J* = 7.3 Hz, 1H), 3.24 (d, *J* = 7.3 Hz, 2H), 0.23 (s, 9H), 0.20 (s, 9H). **^{13}C NMR (126 MHz, CD_2Cl_2)** δ 161.6 (d, *J* = 241.7 Hz), 152.1, 139.7 (d, *J* = 3.1 Hz), 130.0 (d, *J* = 7.8 Hz), 115.2 (d, *J* = 21.1 Hz), 82.1, 31.1, 0.7, 0.1. **^{19}F NMR (471 MHz, CD_2Cl_2)** δ –119.41. **HRMS m/z (EI):** calculated for $C_{15}H_{25}F_1O_2Si_2$ $[M]^+$: 312.1372, found 312.1371.

4-(2-(4-Chlorophenyl)ethylidene)-2,2,6,6-tetramethyl-3,5-dioxa-2,6-disilaheptane (1h)

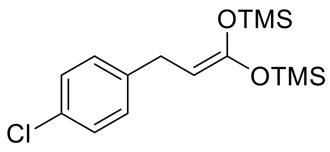

Prepared according to the general procedure using 3-(4-chlorophenyl)propanoic acid (1.84 g, 10 mmol) and obtained after distillation (bp = 90 °C at $2.3*10^{-2}$ mbar) as colorless liquid (2.41 g, 73% yield). **^{1}H NMR (501 MHz, CD_2Cl_2)** δ 7.24–7.18 (m, 2H), 7.16–7.12 (m, 2H), 3.73 (t, *J* = 7.3 Hz, 1H), 3.23 (d, *J* = 7.3 Hz, 2H), 0.23 (s, 9H), 0.20 (s, 9H). **^{13}C NMR (126 MHz,**

CD_2Cl_2) δ 152.2, 142.6, 131.4, 130.1, 128.6, 81.7, 31.3, 0.7, 0.1. **HRMS m/z (EI):** calculated for $C_{15}H_{25}Cl_1O_2Si_2$ $[M]^+$: 328.1076, found 328.1073.

4-(2-(3-Chlorophenyl)ethylidene)-2,2,6,6-tetramethyl-3,5-dioxa-2,6-disilaheptane (1i)

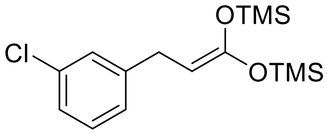

Prepared according to the general procedure using 3-(3-chlorophenyl)propanoic acid (1.84 g, 10 mmol) and obtained after distillation (bp = 83 °C at $1.1*10^{-2}$ mbar) as colorless liquid (2.47 g, 75% yield). **^{1}H NMR (501 MHz, CD_2Cl_2)** δ 7.22–7.17 (m, 2H), 7.16–7.07 (m, 2H), 3.74 (t, *J* = 7.3 Hz, 1H), 3.25 (d, *J* = 7.3 Hz, 2H), 0.24 (s, 9H), 0.20 (s, 9H). **^{13}C NMR (126 MHz, CD_2Cl_2)** δ 152.4, 146.3, 134.4, 130.0, 128.8, 127.0, 126.1, 81.4, 31.7, 0.8, 0.2. **HRMS m/z (EI):** calculated for $C_{15}H_{25}Cl_1O_2Si_2$ $[M]^+$: 328.1076, found 328.1073.

4-(2-(2-Chlorophenyl)ethylidene)-2,2,6,6-tetramethyl-3,5-dioxa-2,6-disilaheptane (1j)

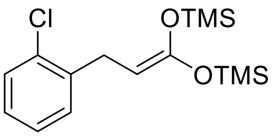

Prepared according to the general procedure using 3-(2-chlorophenyl)propanoic acid (1.84 g, 10 mmol) and obtained after distillation (bp = 85 °C at $2.8*10^{-2}$ mbar) as colorless liquid (2.27 g, 69% yield). **^{1}H NMR (501 MHz, CD_2Cl_2)** δ 7.35–7.27 (m, 2H), 7.22–7.09 (m, 2H), 3.77 (t, *J* = 7.3 Hz, 1H), 3.35 (d, *J* = 7.3 Hz, 2H), 0.24 (s, 9H), 0.19 (s, 9H). **^{13}C NMR (126 MHz, CD_2Cl_2)** δ 152.4, 141.2, 134.3, 130.5, 129.6, 127.5, 127.2, 80.2, 29.8, 0.8, 0.2. **HRMS m/z (EI):** calculated for $C_{15}H_{25}Cl_1O_2Si_2$ $[M]^+$: 328.1076, found 328.1072.

2,2,6,6-Tetramethyl-4-(2-(naphthalen-1-yl)ethylidene)-3,5-dioxa-2,6-disilaheptane (1k)

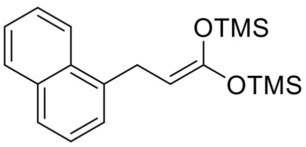

Prepared according to the general procedure using 3-(naphthalen-1-yl)propanoic acid (2.00 g, 10 mmol) and obtained after distillation (bp = 122 °C at $8.3*10^{-3}$ mbar) as colorless liquid (1.45 g, 29 mmol, 42% yield). **^{1}H NMR (501 MHz, CD_2Cl_2)** δ 8.16–8.12 (m, 1H), 7.86–7.82 (m, 1H), 7.71–7.67 (m, 1H), 7.52–7.44 (m, 2H), 7.40–7.35 (m, 2H), 3.81 (t, *J* = 7.1 Hz, 1H), 3.71 (d, *J* = 7.1 Hz, 2H), 0.25 (d, *J* = 0.5 Hz, 9H), 0.20 (s, 9H). **^{13}C NMR (126 MHz, CD_2Cl_2)** δ 151.4, 139.8, 134.4, 132.7, 129.0, 126.8, 126.1, 126.0, 125.9, 125.9, 124.97, 82.2, 29.7, 0.8, 0.1. **HRMS m/z (EI):** calculated for $C_{19}H_{28}O_2Si_2$ $[M]^+$: 344.1622, found 344.1621.

2,2,6,6-Tetramethyl-4-(3-(thiophen-2-yl)propylidene)-3,5-dioxa-2,6-disilaheptane (1l)

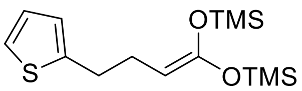

Prepared according to the general procedure using 4-(thiophen-2-yl)butanoic acid (1.70 g, 10 mmol) and obtained after distillation (bp = 55 °C at $1.2*10^{-2}$ mbar) as colorless liquid (2.51 g, 80% yield). **^{1}H NMR (501 MHz, CD_2Cl_2)** δ 7.10 (dd, *J* = 5.1, 1.2 Hz, 1H), 6.90 (dd, *J* = 5.1, 3.4 Hz, 1H), 6.81–6.76 (m, 1H), 3.58 (t, *J* = 7.1 Hz, 1H), 2.81 (td, *J* = 7.6, 1.0 Hz, 2H), 2.26 (q, *J* = 7.2 Hz, 2H), 0.20 (s, 9H), 0.18 (s, 9H). **^{13}C NMR (126 MHz, CD_2Cl_2)** δ 151.6, 146.3, 127.1, 124.5, 123.2, 82.1, 31.4, 28.2, 0.7, 0.0. **HRMS m/z (CI):** calculated for $C_{14}H_{27}S_1O_2Si_2$ $[M+H]^+$: 315.1265, found 315.1264.

4-(4-Chlorobenzylidene)-2,2,6,6-tetramethyl-3,5-dioxa-2,6-disilaheptane (1m)

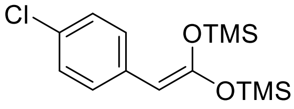

Prepared according to the general procedure using 2-(4-chlorophenyl)acetic acid (1.70 g, 10 mmol) and obtained after distillation (bp = 78 °C at $6.3*10^{-3}$ mbar) as colorless liquid (1.76 g, 56% yield). **^{1}H NMR (501 MHz, CD_2Cl_2)** δ 7.32 (d, *J* = 8.8 Hz, 2H), 7.16 (d, *J* = 8.7 Hz, 2H), 4.57 (s, 1H), 0.33 (s, 9H), 0.29 (s, 9H). **^{13}C NMR (126 MHz, CD_2Cl_2)** δ 153.2, 136.9, 128.8, 128.5, 127.9, 84.5, 0.8, 0.1. **HRMS m/z (EI):** calculated for $C_{14}H_{23}Cl_1O_2Si_2$ $[M]^+$: 314.0920, found 314.0916.

4-(3-Chlorobenzylidene)-2,2,6,6-tetramethyl-3,5-dioxa-2,6-disilaheptane (1n)

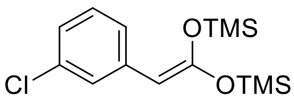

Prepared according to the general procedure using 2-(3-chlorophenyl)acetic acid (1.70 g, 10 mmol) and obtained after distillation (bp = 76 °C at $9.0*10^{-3}$ mbar) as colorless liquid (1.95 g, 62% yield). **^{1}H NMR (501 MHz, CD_2Cl_2)** δ 7.45 (t, *J* = 1.9 Hz, 1H), 7.21 (dt, *J* = 7.9, 1.4 Hz, 1H), 7.13 (t, *J* = 7.9 Hz, 1H), 6.95 (ddd, *J* = 7.9, 2.1, 1.1 Hz, 1H), 4.56 (s, 1H), 0.33 (s, 9H), 0.31 (s, 9H). **^{13}C NMR (126 MHz, CD_2Cl_2)** δ 153.7, 140.3, 134.3, 129.7, 126.3, 124.8, 123.6, 84.5, 0.8, 0.1. **HRMS m/z (EI):** calculated for $C_{14}H_{23}Cl_1O_2Si_2$ $[M]^+$: 314.0920, found 314.0916.

4-(2-Chlorobenzylidene)-2,2,6,6-tetramethyl-3,5-dioxa-2,6-disilaheptane (1o)

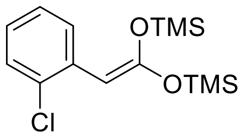

Prepared according to the general procedure using 2-(2-chlorophenyl)acetic acid (1.70 g, 10 mmol) and obtained after distillation (bp = 73 °C at $9.3*10^{-3}$ mbar) as colorless liquid (1.61 g, 51% yield). **^{1}H NMR (501 MHz, CD_2Cl_2)** δ 7.85 (d, *J* = 8.1 Hz, 1H), 7.28 (d, *J* = 8.0 Hz, 1H), 7.15 (t, *J* = 7.7 Hz, 1H), 6.93 (td, *J* = 7.7, 1.6 Hz, 1H), 5.02 (s, 1H), 0.37 (s, 9H), 0.29 (s, 9H). **^{13}C NMR (126 MHz, CD_2Cl_2)** δ 154.0, 135.8, 131.1, 129.4, 128.1, 126.9, 124.8, 81.1, 0.8, 0.0. **HRMS m/z (ESI):** calculated for $C_{14}H_{24}Cl_1O_2Si_2$ $[M+H]^+$: 315.0998, found 315.0997.

4-(4-Bromobenzylidene)-2,2,6,6-tetramethyl-3,5-dioxa-2,6-disilaheptane (1p)

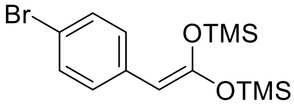

Prepared according to the general procedure using 2-(4-bromophenyl)acetic acid (2.15 g, 10 mmol) and obtained after distillation (bp = 83 °C at $1.0*10^{-3}$ mbar) as colorless liquid (1.98 g, 55% yield). **^{1}H NMR (501 MHz, CD_2Cl_2)** δ 7.37–7.19 (m, 4H), 4.55 (s, 1H), 0.33 (s, 9H), 0.29 (s, 9H). **^{13}C NMR (126 MHz, CD_2Cl_2)** δ 153.4, 137.4, 131.4, 128.3, 116.6, 84.5, 0.8, 0.1. **HRMS m/z (ESI):** calculated for $C_{14}H_{24}Br_1O_2Si_2$ $[M+H]^+$: 359.0493, found 359.0497.

4-(3,4-Dichlorobenzylidene)-2,2,6,6-tetramethyl-3,5-dioxa-2,6-disilaheptane (1q)

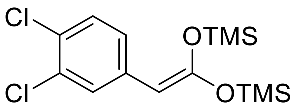

Prepared according to the general procedure using 2-(3,4-dichlorophenyl)acetic acid (2.05 g, 10 mmol) and obtained after distillation (bp = 100 °C at $1.5*10^{-2}$ mbar) as colorless liquid (2.06 g, 59% yield). **^{1}H NMR (501 MHz, CD_2Cl_2)** δ 7.56 (d, *J* = 2.1 Hz, 1H), 7.25 (d, *J* = 8.5 Hz, 1H), 7.18 (dd, *J* = 8.5, 2.1 Hz, 1H), 4.52 (s, 1H), 0.33 (s, 9H), 0.30 (s, 9H). **^{13}C NMR (126 MHz, CD_2Cl_2)** δ 154.1, 138.8, 132.2, 130.2, 127.9, 126.3, 126.1, 83.6, 0.8, 0.1. **HRMS m/z (EI):** calculated for $C_{14}H_{22}Cl_2O_2Si_2$ $[M]^+$: 348.0530, found 348.0525.

4-Ethylidene-2,2,6,6-tetramethyl-3,5-dioxa-2,6-disilaheptane (1r)

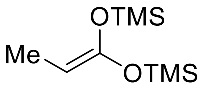

Prepared according to the general procedure using propionic acid (740 mg, 10 mmol) and obtained after distillation (bp = 61 °C at 20 mbar) as colorless liquid

(1.92 g, 88% yield). **^{1}H NMR (501 MHz, CD_2Cl_2)** δ 3.55 (q, *J* = 6.5 Hz, 1H), 1.44 (d, *J* = 6.5 Hz, 3H), 0.20 (s, 9H), 0.19 (s, 9H). **^{13}C NMR (126 MHz, CD_2Cl_2)** δ 151.3, 77.4, 10.4, 0.7, 0.1. **HRMS m/z (EI):** calculated for $C_9H_{22}O_2Si_2$ $[M]^+$: 218.1153, found 218.1152.

2,2,6,6-Tetramethyl-4-propylidene-3,5-dioxa-2,6-disilaheptane (1s)

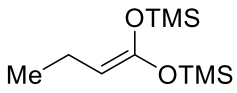

Prepared according to the general procedure using butyric acid (880 mg, 10 mmol) and obtained after distillation (bp = 67 °C at 17 mbar) as colorless liquid (1.86 g, 80% yield). **^{1}H NMR (501 MHz, CD_2Cl_2)** δ 3.55 (t, *J* = 7.1 Hz, 1H), 1.90 (p, *J* = 7.4 Hz, 2H), 0.90 (t, *J* = 7.5 Hz, 3H), 0.21 (s, 9H), 0.18 (s, 9H). **^{13}C NMR (126 MHz, CD_2Cl_2)** δ 150.5, 85.8, 19.1, 15.7, 0.6, 0.0. **HRMS m/z (ESI):** calculated for $C_{10}H_{25}O_2Si_2$ $[M+H]^+$: 233.1388, found 233.1287.

4-Butylidene-2,2,6,6-tetramethyl-3,5-dioxa-2,6-disilaheptane (1t)

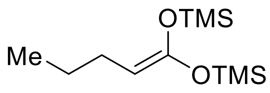

Prepared according to the general procedure using pentanoic acid (1.02 g, 10 mmol) and obtained after distillation (bp = 87 °C at 24 mbar) as colorless liquid (1.92 g, 78% yield). **^{1}H NMR (501 MHz, CD_2Cl_2)** δ 3.54 (t, *J* = 7.1 Hz, 1H), 1.87 (q, *J* = 7.2 Hz, 2H), 1.30 (h, *J* = 7.3 Hz, 2H), 0.87 (t, *J* = 7.4 Hz, 3H), 0.21 (s, 9H), 0.18 (s, 9H). **^{13}C NMR (126 MHz, CD_2Cl_2)** δ 151.0, 83.7, 27.8, 24.3, 14.1, 0.7, 0.1. **HRMS m/z (ESI):** calculated for $C_{11}H_{27}O_2Si_2$ $[M+H]^+$: 247.1544, found 247.1543.

2,2,6,6-Tetramethyl-4-pentylidene-3,5-dioxa-2,6-disilaheptane (1u)

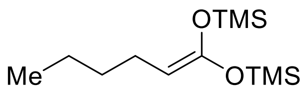

Prepared according to the general procedure using hexanoic acid (1.16 g, 10 mmol) and obtained after distillation (bp = 41 °C at $5.7*10^{-2}$ mbar) as colorless liquid (1.84 g, 71% yield). **^{1}H NMR (501 MHz, CD_2Cl_2)** δ 3.53 (t, *J* = 7.1 Hz, 1H), 1.89 (q, *J* = 7.1 Hz, 2H), 1.33–1.23 (m, 4H), 0.88 (t, *J* = 7.1 Hz, 3H), 0.21 (s, 9H), 0.18 (s, 9H). **^{13}C NMR (126 MHz, CD_2Cl_2)** δ 150.8, 83.9, 33.4, 25.2, 22.8, 14.3, 0.7, 0.0. **HRMS m/z (CI):** calculated for $C_{12}H_{29}O_2Si_2$ $[M+H]^+$: 261.1701, found 261.1699.

2,2,6,6-Tetramethyl-4-(3-methylbutylidene)-3,5-dioxa-2,6-disilaheptane (1v)

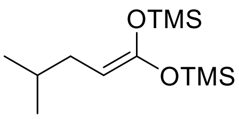

Prepared according to the general procedure using 4-methylpentanoic acid (1.16 g, 10 mmol) and obtained after distillation (bp = 35 °C at $8.0*10^{-2}$ mbar) as colorless liquid (1.72 g, 66% yield). **^{1}H NMR (501 MHz, CD_2Cl_2)** δ 3.54 (t, *J* = 7.3 Hz, 1H), 1.80–1.76 (m, 2H), 1.56–1.46 (m, 1H), 0.86 (d, *J* = 6.6 Hz, 6H), 0.22 (s, 9H), 0.18 (s, 9H). **^{13}C NMR (126 MHz, CD_2Cl_2)** δ 151.3, 82.2, 34.8, 30.0, 22.6, 0.7, 0.1. **HRMS m/z (ESI):** calculated for $C_{12}H_{29}O_2Si_2$ $[M+H]^+$: 261.1701, found 261.1700.

2,2,6,6-Tetramethyl-4-(4-methylpentylidene)-3,5-dioxa-2,6-disilaheptane (1w)

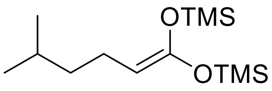

Prepared according to the general procedure using 5-methylhexanoic acid (1.30 g, 10 mmol) and obtained after distillation (bp = 53 °C at $9.0*10^{-2}$ mbar) as colorless liquid (2.08 g, 76% yield). **^{1}H NMR (501 MHz, CD_2Cl_2)** δ 3.52 (t, *J* = 7.1 Hz, 1H), 1.93–1.87 (m, 2H), 1.60–1.48 (m, 1H), 1.20–1.14 (m, 2H), 0.87 (d, *J* = 6.6 Hz, 6H), 0.21 (s, 9H), 0.18 (s, 9H). **^{13}C NMR (126 MHz, CD_2Cl_2)** δ 150.8, 84.0, 40.5, 28.0, 23.5, 22.9, 0.7, 0.1. **HRMS m/z (ESI):** calculated for $C_{13}H_{31}O_2Si_2$ $[M+H]^+$: 275.1857, found 275.1855.

4-(2-Cyclopentylethylidene)-2,2,6,6-tetramethyl-3,5-dioxa-2,6-disilaheptane (1x)

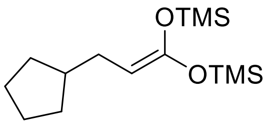

Prepared according to the general procedure using 3-cyclopentylpropanoic acid (1.42 g, 10 mmol) and obtained after distillation (bp = 57 °C at $8.6*10^{-3}$ mbar) as colorless liquid (2.06 g, 72% yield). **^{1}H NMR (501 MHz, CD_2Cl_2)** δ 3.56 (t, *J* = 7.2 Hz, 1H), 1.89 (t, *J* = 7.1 Hz, 2H), 1.77 (hept, *J* = 7.2 Hz, 1H), 1.72–1.65 (m, 2H), 1.63–1.54 (m, 2H), 1.54–1.44 (m, 2H), 1.18–1.08 (m, 2H), 0.21 (s, 9H), 0.18 (s, 9H). **^{13}C NMR (126 MHz, CD_2Cl_2)** δ 151.0, 83.1, 41.8, 32.7, 31.7, 25.8, 0.7, 0.1. **HRMS m/z (ESI):** calculated for $C_{14}H_{31}O_2Si_2$ $[M+H]^+$: 287.1857, found 287.1854.

4-(4-Methoxybutylidene)-2,2,6,6-tetramethyl-3,5-dioxa-2,6-disilaheptane (1y)

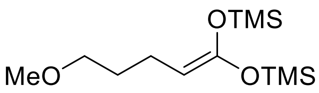

Prepared according to the general procedure using 5-methoxypentanoic acid (1.32 g, 10 mmol) and obtained after distillation (bp = 51 °C at $5.1*10^{-2}$ mbar) as colorless liquid (1.63 g, 59% yield). **^{1}H NMR (501 MHz, CD_2Cl_2)** δ 3.53 (t, *J* = 7.2 Hz, 1H), 3.33 (t, *J* = 6.7 Hz, 2H), 3.28 (s, 3H), 1.93 (q, *J* = 7.3 Hz, 2H), 1.53 (p, *J* = 6.9 Hz, 2H), 0.21 (s, 9H), 0.19 (s, 9H). **^{13}C NMR (126 MHz, CD_2Cl_2)** δ

151.2, 83.0, 73.0, 58.7, 31.1, 22.2, 0.7, 0.0. **HRMS m/z (ESI):** calculated for $C_{12}H_{29}O_3Si_2$ $[M+H]^+$: 277.1650, found 277.1649.

2,2,6,6-Tetramethyl-4-(4-methylpent-4-en-1-ylidene)-3,5-dioxa-2,6-disilaheptane (1z)

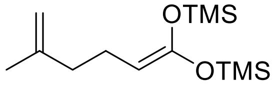

Prepared according to the general procedure using 5-methylhex-5-enoic acid[2] (1.28 g, 10 mmol) and obtained after distillation (bp = 50 °C at $5.2*10^{-2}$ mbar) as colorless liquid (1.22 g, 45% yield). **^{1}H NMR (501 MHz, CD_2Cl_2)** δ 4.71–4.63 (m, 2H), 3.53 (t, *J* = 6.8 Hz, 1H), 2.08–1.98 (m, 4H), 1.71 (s, 3H), 0.21 (s, 9H), 0.19 (s, 9H). **^{13}C NMR (126 MHz, CD_2Cl_2)** δ 151.0, 146.8, 110.1, 83.1, 39.3, 24.0, 22.5, 0.7, 0.0. **HRMS m/z (EI):** calculated for $C_{13}H_{28}O_2Si_2$ $[M]^+$: 272.1622, found 272.1621.

(*R*)-2,2,6,6-Tetramethyl-4-(3-methylpentylidene)-3,5-dioxa-2,6-disilaheptane (1A)

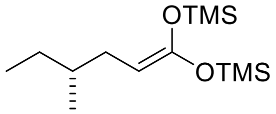

Prepared according to the general procedure using (*R*)-4-methylhexanoic acid[3] (1.30 g, 10 mmol) and obtained after distillation (bp = 42 °C at $1.4*10^{-2}$ mbar) as colorless liquid (2.07 g, 76% yield). **^{1}H NMR (501 MHz, CD_2Cl_2)** δ 3.53 (t, *J* = 7.3 Hz, 1H), 1.94–1.86 (m, 1H), 1.78–1.70 (m, 1H), 1.37–1.25 (m, 2H), 1.16–1.08 (m, 1H), 0.88–0.82 (m, 6H), 0.22 (s, 9H), 0.18 (s, 9H). **^{13}C NMR (126 MHz, CD_2Cl_2)** δ 151.3, 81.9, 36.4, 32.4, 29.6, 19.5, 12.0, 0.7, 0.1. **HRMS m/z (ESI):** calculated for $C_{13}H_{31}O_2Si_2$ $[M+H]^+$: 275.1857, found 275.1855.

(*S*)-2,2,6,6-Tetramethyl-4-(3-methylpentylidene)-3,5-dioxa-2,6-disilaheptane (1B)

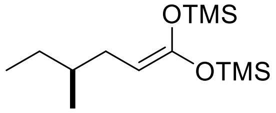

Prepared according to the general procedure using (*S*)-4-methylhexanoic acid[3] (1.30 g, 10 mmol) and obtained after distillation (bp = 42 °C at $1.2*10^{-2}$ mbar) as colorless liquid (1.96 g, 72% yield). **^{1}H NMR (501 MHz, CD_2Cl_2)** δ 3.53 (t, *J* = 7.3 Hz, 1H), 1.94–1.86 (m, 1H), 1.78–1.70 (m, 1H), 1.37–1.25 (m, 2H), 1.16–1.08 (m, 1H), 0.88–0.82 (m, 6H), 0.22 (s, 9H), 0.18 (s, 9H). **^{13}C NMR (126 MHz, CD_2Cl_2)** δ 151.3, 81.9, 36.4, 32.4, 29.6, 19.5, 11.9, 0.7, 0.1. **HRMS m/z (ESI):** calculated for $C_{13}H_{31}O_2Si_2$ $[M+H]^+$: 275.1857, found 275.1855.

4-(4-Bromobutylidene)-2,2,6,6-tetramethyl-3,5-dioxa-2,6-disilaheptane (1C)

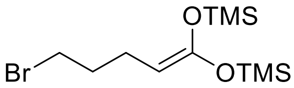

Prepared according to the general procedure using 5-bromopentanoic acid (1.81 g, 10 mmol) and obtained after distillation (bp = 60 °C at $4.1*10^{-2}$ mbar) as colorless liquid (1.40 g, 43% yield). **^{1}H NMR (501 MHz, CD_2Cl_2)** δ 3.50 (t, *J* = 7.2 Hz, 1H), 3.42 (t, *J* = 6.9 Hz, 2H), 2.04 (q, *J* = 7.1 Hz, 2H), 1.85 (p, *J* = 7.0 Hz, 2H), 0.22 (s, 9H), 0.19 (s, 9H). **^{13}C NMR (126 MHz, CD_2Cl_2)** δ 151.9, 81.2, 34.5, 34.5, 24.3, 0.7, 0.0. **HRMS m/z (ESI):** calculated for $C_{11}H_{26}Br_1O_2Si_2$ $[M+H]^+$: 325.0649, found 325.0646.

3. Synthesis and Characterization of Silylated Aminomethyl Ethers

General Procedure:

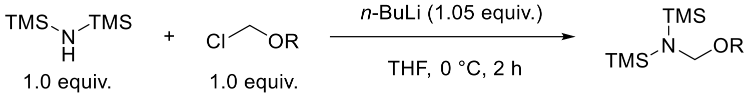

To a stirring solution of freshly distilled bis(trimethylsilyl)amine (HMDS; 10 mmol, 2.1 mL, 1.0 equiv.) in THF (10 mL) under an atmosphere of argon, *n*-BuLi (2.5 M solution in hexane, 10.5 mmol, 4.2 mL, 1.05 equiv.) was added dropwise at 0 °C and the resulting reaction mixture was stirred for additional 30 min at 0 °C. The corresponding chloromethyl ether (10 mmol, 1.0 equiv.) was added dropwise via cannula at 0 °C and the reaction was stirred for 2 h at 0 °C. After concentration under reduced pressure, the crude product was purified by distillation under reduced pressure to afford the silylated aminomethyl ether as colorless liquid, which was stored in Schlenk flask under an atmosphere of argon. (When the reaction was performed on a scale over 50 mmol, the dropping of chloromethyl ether must be very careful and slowly. It's easy to bumping at the moment when the lithium chloride precipitated. Optionally, lithium chloride could be filtered away under an atmosphere of argon with dry hexane (10–20 mL), and the crude product was concentrated in vacuo before distillation.)

***N*-(Methoxymethyl)-1,1,1-trimethyl-*N*-(trimethylsilyl)silanamine (2a)**

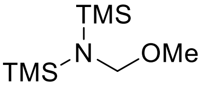

Prepared according to the general procedure using chloro(methoxy)methane (805 mg, 10 mmol) and obtained after distillation (bp = 90 °C at 110 mbar) as

colorless liquid (1.87 g, 91% yield). **^{1}H NMR (501 MHz, CD_2Cl_2)** δ 4.27 (s, 2H), 3.14 (s, 3H), 0.13 (s, 18H). **^{13}C NMR (126 MHz, CD_2Cl_2)** δ 81.2, 53.77, 2.1. **HRMS m/z (CI):** calculated for $C_8H_{22}N_1O_1Si_2$ $[M-H]^-$: 204.1234, found 204.1231.

_N_-((Benzyloxy)methyl)-1,1,1-trimethyl-_N_-(trimethylsilyl)silanamine (2b)

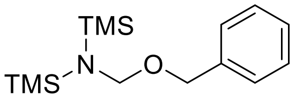

Prepared according to the general procedure using ((chloromethoxy)methyl)benzene (1.57 g, 10 mmol) and obtained after distillation as colorless liquid (2.28 g, 81% yield). **^{1}H NMR (501 MHz, CD_2Cl_2)** δ 7.37–7.22 (m, 5H), 4.44 (s, 2H), 4.39 (s, 2H), 0.15 (s, 18H). **^{13}C NMR (126 MHz, CD_2Cl_2)** δ 139.8, 128.7, 128.1, 127.7, 79.9, 68.8, 2.1. **HRMS m/z (ESI):** calculated for $C_{14}H_{27}N_1O_1Si_2Na_1$ $[M+Na]^+$: 304.1523, found 304.1523.

_N_-(Isopropoxymethyl)-1,1,1-trimethyl-_N_-(trimethylsilyl)silanamine (2c)

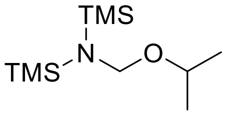

Prepared according to the general procedure using 2-(chloromethoxy)propane (1.09 g, 10 mmol) and obtained after distillation (bp = 73 °C at 17 mbar) as colorless liquid (2.05 g, 88% yield). **^{1}H NMR (501 MHz, CD_2Cl_2)** δ 4.31 (s, 2H), 3.59–3.48 (m, 1H), 1.10 (d, J = 6.1 Hz, 6H), 0.12 (s, 18H). **^{13}C NMR (126 MHz, CD_2Cl_2)** δ 77.78, 67.61, 22.89, 2.11. **HRMS m/z (ESI):** calculated for $C_{10}H_{27}N_1O_2Si_2Na_1$ $[M+Na]^+$: 256.1523, found 256.1524.

_N_-((Cyclohexyloxy)methyl)-1,1,1-trimethyl-_N_-(trimethylsilyl)silanamine (2d)

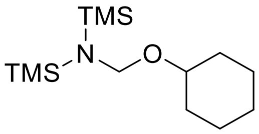

Prepared according to the general procedure using (chloromethoxy)cyclohexane (1.49 g, 10 mmol) and obtained after distillation (bp = 64 °C at $5.6*10^{-2}$ mbar) as colorless liquid (2.32 g, 85% yield). **^{1}H NMR (501 MHz, CD_2Cl_2)** δ 4.34 (s, 2H), 3.28–3.17 (m, 1H), 1.83–1.75 (m, 2H), 1.73–1.67 (m, 2H), 1.56–1.45 (m, 1H), 1.31–1.19 (m, 5H), 0.12 (s, 18H). **^{13}C NMR (126 MHz, CD_2Cl_2)** δ 77.6, 73.7, 33.2, 26.5, 24.7, 2.1. **HRMS m/z (ESI):** calculated for $C_{13}H_{31}N_1O_1Si_2Na_1$ $[M+Na]^+$: 296.1836, found 296.1835.

4. Procedure for Reaction Development

An oven-dried 1.5 mL vial was charged with catalyst and a magnetic stir bar under an atmosphere of argon. Dry solvent, bis-silyl ketene acetal **1a** (8 µL, 0.024 mmol, 1.2 equiv.), and mesitylene (internal standard, 0.9 µL, 0.02 mmol, 1.0 equiv.) were added. The vial was cooled to required reaction temperature. Then **2a** (5 µL, 0.02 mmol, 1.0 equiv.) was added and the reaction was stirred for 3 d.

The reaction mixture was treated with triethylamine (0.02 mmol, 2.8 µL) at reaction temperature. Then an aliquot of the reaction mixture was taken to determine the yield by crude ^{1}H NMR in Tol-d_8. The rest was diluted with diethyl ether (0.6 mL), water (0.6 mL) and MeOH (0.3 mL). The mixture was vigorously stirred for 30 s, then the aqueous phase was taken to determine the e.r. by chiral HPLC.

Table S1. Results of Initial Screening of Catalysts

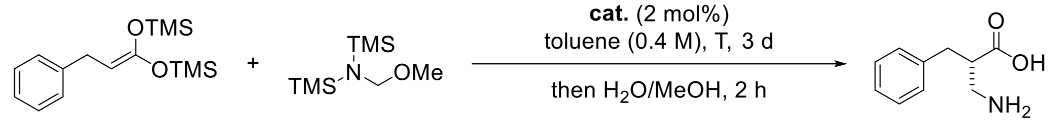

1a, 1.2 equiv. **2a**, 1.0 equiv. **4a**

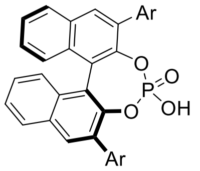

(*S*)-Phosphoric Acid
Ar = 3,5-(CF_3)-C_6H_3

–20 °C: **no reaction**
0 °C: **no reaction**
80 °C: **no reaction**

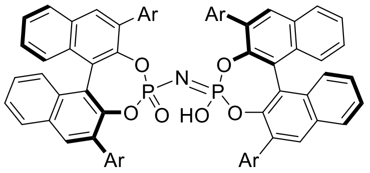

(*S*,*S*)-Imidodiphosphoric Acid
Ar = 3,5-(CF_3)-C_6H_3

–20 °C: **no reaction**
0 °C: 93%, 50:50 e.r.

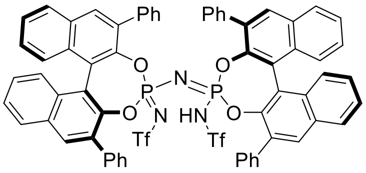

(*S*,*S*)-Imidodiphosphorimidate

–20 °C: 80%, 51.5:48.5 e.r.

Table S2. Initial Results of α,α-Disubstituted Carboxylic Acid-derived bis-Silyl Ketene Acetal[4]

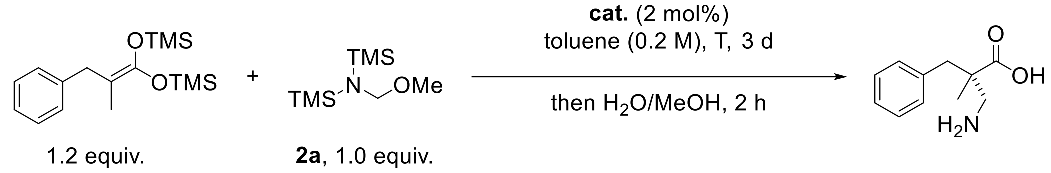

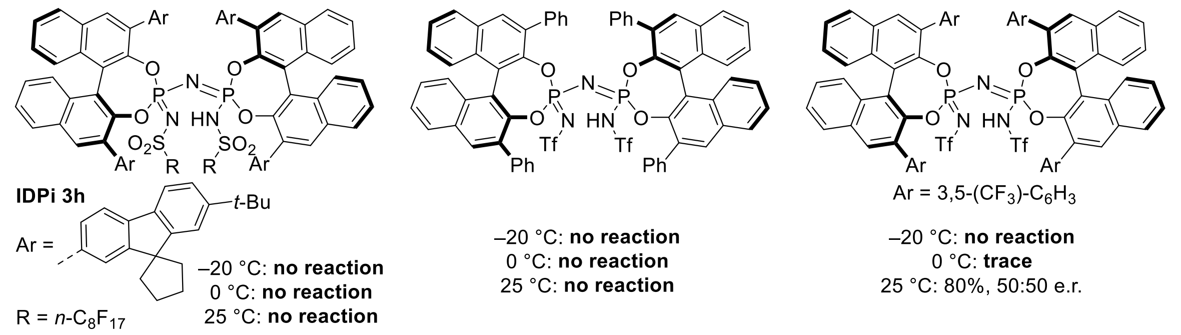

Table S3. Initial Results of *N*-Trimethylsilylbenzaldimin[5]

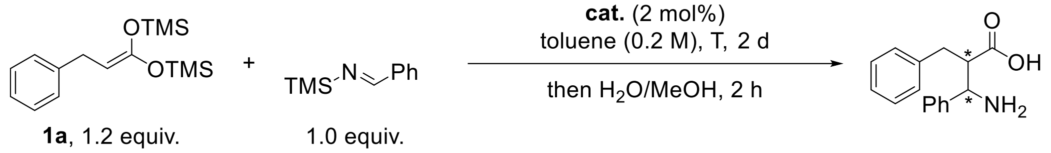

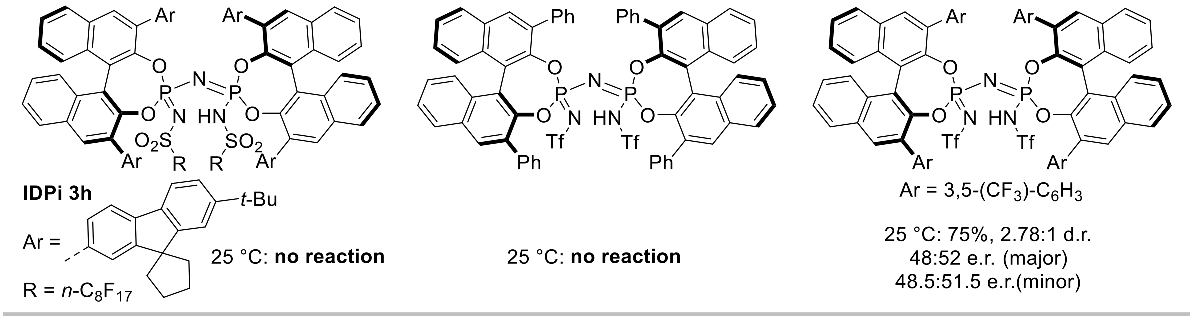

5. Enantioselective Aminomethylation and Derivatization of Product

General Procedure for Enantioselective Aminomethylation:

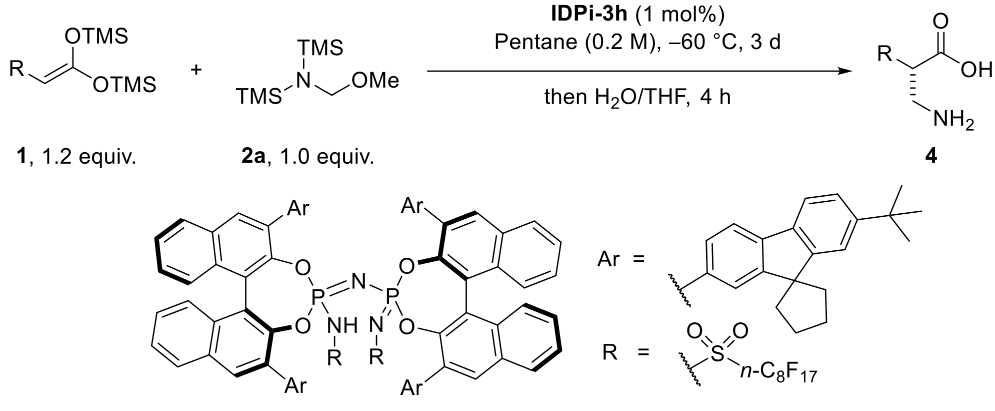

IDPi-3h

An oven-dried 5 ml vial was charged with **IDPi-3h** (2.0 µmol, 5.4 mg, 1 mol%) and a magnetic stir bar under an atmosphere of argon. Dry pentane (1 mL) and bis-silyl ketene acetal **1** (0.24 mmol, 1.2 equiv.) were added. The vial was cooled to –60 °C. The pre-cooled **2a** (0.2 mmol, 1.0 equiv.) was added dropwise at –60 °C and the reaction was stirred at –60 °C for 3 d.

Afterwards, the reaction mixture was treated with triethylamine (0.02 mmol, 2.8 µL, 0.1 equiv.) at –60 °C and diluted with water (2 mL) and THF (1 mL) at rt. After the mixture was stirred for 4 h, the solvent of the reaction mixture was directly removed under reduced pressure. The solid residue was washed with DCM using Büchner funnel to get the corresponding free β^2-amino acids in >99% purity (analyzed by ^{1}H NMR). The organic phase was collected and the solvent was removed under reduced pressure to recover the catalyst **IDPi-3h** by column chromatography on silica.

Alternatively, after diluting procedure and stirring for 4 h, the aqueous phase was separated and extracted with DCM (3x3 mL). The aqueous phase was collected and the water was removed under reduced pressure. The solid product was washed with DCM to get the corresponding free β^2-amino acids in >99% purity (analyzed by ^{1}H NMR). The organic phase was combined and the solvent was removed under reduced pressure to recover the catalyst **IDPi-3h** by column chromatography on silica. (This purification process is suitable for aliphatic β^2-amino acids and some aromatic β^2-amino acids, especially in gram-scale.)

Racemate synthesis: The racemic product was synthesized at rt for 2 h by using TMSOTf as the catalyst instead of **IDPi-3h** with the same purification process.[6]

General Procedure for Derivatization:

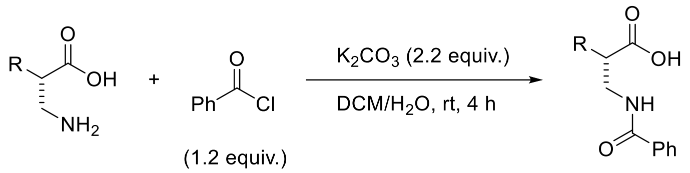

4 **10**

A vial was charged with a magnetic stir bar, amino acid (0.01 mmol, 1 equiv.), benzoyl chloride (0.012 mmol, 1.7 mg, 1.2 equiv.), K_2CO_3 (0.022 mmol, 2.5 mg, 2.2 equiv.), DCM (0.2 mL) and H_2O (0.2 mL). The mixture was stirred vigorously for 4 h and then 1 M HCl was added until the aqueous solution was acidic (pH<3). The aqueous phase was separated and extracted with DCM (3x3 mL). The organic phase was combined and the solvent was removed under reduced pressure. The crude product was purified by prep-TLC or column chromatography on silica (EtOAc/hexanes/AcOH = 33:66:1).

In situ Derivatization: After aminomethylation, the reaction mixture was treated with triethylamine (0.02 mmol, 2.8 µL) at –60 °C. Then the reaction mixture was directly treated according to the general procedure for derivatization and purified by prep-TLC. The obtained product was used to determine the e.r. by HPLC using a chiral stationary phase.

(*S*)-3-Amino-2-benzylpropanoic acid (4a)

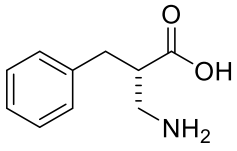

The reaction was performed according to the general procedure and after purification process afford 35.4 mg (99%, 96:4 e.r.) of **4a** as a white solid: **^{1}H NMR (501 MHz, D_2O)** δ 7.43–7.36 (m, 2H), 7.35–7.25 (m, 3H), 3.15–3.06 (m, 1H), 3.06–2.97 (m, 2H), 2.91–2.81 (m, 2H). **^{13}C NMR (126 MHz, D_2O)** δ 179.5, 138.5, 129.0, 128.7, 126.7, 46.9, 40.6, 36.2. **HRMS m/z (ESI):** calculated for $C_{10}H_{12}N_1O_2$ $[M-H]^-$: 178.0874, found 178.0875. $[\alpha]_D^{25}$ = –17.3 (*c* = 0.45, 1 M HCl). [Lit.[7] $[\alpha]_D^{25}$ = –17.3 (*c* = 1.85, 1 M HCl)]

The enantiomeric excess was determined by chiral HPLC analysis on Chirabiotic T2 column. Conditions: MeOH/water = 90:10, flow rate = 1.0 mL/min, uv-vis detection at λ = 220 nm, t_{R1} = 17.9 min (minor), t_{R2} = 21.5 min (major).

For 10 mmol scale: The reaction was performed in 10 mmol scale of **2a** according to the general procedure, which afforded 1.77 g (99%, 95.5:4.5 e.r.) of **4a** as a white solid after purification. Catalyst **IDPi-3h** (262 mg, 96%) was recovered from the organic phase after column chromatography on silica and acidified by HCl (see a detailed procedure in the paragraph of Synthesis and Characterization of Catalysts).

(*S*)-2-(Aminomethyl)-4-phenylbutanoic acid (4b)

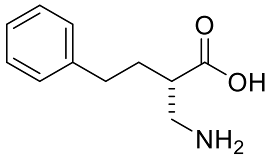

The reaction was performed according to the general procedure and after purification process afford 36.5 mg (94%, 95.4:4.5 e.r.) of **4b** as a white solid: **^{1}H NMR (501 MHz, D_2O)** δ 7.42–7.36 (m, 2H), 7.35–7.26 (m, 3H), 3.21–3.05 (m, 2H), 2.74–2.62 (m, 2H), 2.60–2.53 (m, 1H), 1.98–1.81 (m, 2H). **^{13}C NMR (126 MHz, D_2O)** δ 180.4, 141.9, 128.7, 128.5, 126.2, 45.0, 41.1, 32.5, 31.9. **HRMS m/z (ESI):** calculated for $C_{11}H_{14}N_1O_2$ $[M-H]^-$: 192.1030, found 192.1033. $[\alpha]_D^{25}$ = –22.5 (*c* = 0.47, 1 M HCl). [Lit.[8] $[\alpha]_D^{25}$ = –24.4 (*c* = 0.6, 1 M HCl)]

The enantiomeric excess was determined by chiral HPLC analysis on Chirabiotic T2 column. Conditions: MeOH/water = 90:10, flow rate = 1.0 mL/min, uv-vis detection at λ = 220 nm, t_{R1} = 19.3 min (minor), t_{R2} = 21.5 min (major).

(*S*)-2-(Aminomethyl)-5-phenylpentanoic acid (4c)

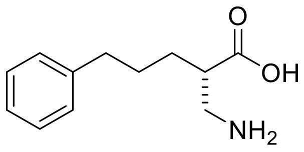

The reaction was performed according to the general procedure and after purification process afford 39.9 mg (96%, 96:4 e.r.) of **4c** as a white solid: **^{1}H NMR (501 MHz, D_2O)** δ 7.38 (t, *J* = 7.5 Hz, 2H), 7.33–7.25 (m, 3H), 3.11 (dd, *J* = 12.8, 8.8 Hz, 1H), 3.04 (dd, *J* = 12.8, 4.9 Hz, 1H), 2.68 (t, *J* = 7.2 Hz, 2H), 2.60–2.51 (m, 1H), 1.72–1.51 (m, 4H). **^{13}C NMR (126 MHz, D_2O)** δ 180.7, 142.7, 128.6, 128.6, 126.0, 45.2, 41.1, 34.8, 29.4, 28.2. **HRMS m/z (ESI):** calculated for $C_{12}H_{16}N_1O_2$ $[M-H]^-$: 206.1187, found 206.1188.

The enantiomeric excess was determined by chiral HPLC analysis on Chirabiotic T2 column. Conditions: MeOH/water = 90:10, flow rate = 1.0 mL/min, uv-vis detection at λ = 220 nm, t_{R1} = 19.3 min (minor), t_{R2} = 24.7 min (major).

(*S*)-3-Amino-2-(4-methylbenzyl)propanoic acid (4d)

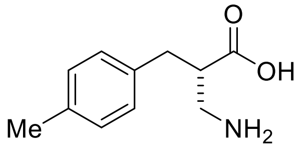

The reaction was performed according to the general procedure and after purification process afford 37.8 mg (98%, 95:5 e.r.) of **4d** as a white solid: **^{1}H NMR (501 MHz, D_2O)** δ 7.25–7.16 (m, 4H), 3.08 (dd, *J* = 12.8, 9.3 Hz, 1H), 3.03–2.93 (m, 2H), 2.86–2.76 (m, 2H), 2.32 (s, 3H). **^{13}C NMR (126 MHz, D_2O)** δ 179.7, 136.8, 135.4, 129.2, 129.0, 47.0, 40.6, 35.7, 20.0. **HRMS m/z (ESI):** calculated for $C_{11}H_{14}N_1O_2$ $[M-H]^-$: 192.1030, found 192.1033. $[\alpha]_D^{25}$ = –14.7 (*c* = 0.60, 1 M HCl).

The enantiomeric excess was determined by chiral HPLC analysis on Chirabiotic T2 column. Conditions: MeOH/water = 90:10, flow rate = 1.0 mL/min, uv-vis detection at λ = 220 nm, t_{R1} = 17.6 min (minor), t_{R2} = 20.3 min (major).

(*S*)-3-Amino-2-(4-methoxybenzyl)propanoic acid (4e)

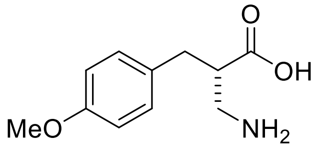

The reaction was performed according to the general procedure and after purification process afford 39.3 mg (94%, 94.5:5.5 e.r.) of **4e** as a white solid: **^{1}H NMR (501 MHz, D_2O)** δ 7.23 (d, *J* = 8.2 Hz, 2H), 6.98 (d, *J* = 8.1 Hz, 2H), 3.83 (s, 3H), 3.12–3.04 (m, 1H), 3.03–2.91 (m, 2H), 2.86–2.75 (m, 2H). **^{13}C NMR (126 MHz, D_2O)** δ 179.7, 157.5, 131.1, 130.1, 114.1, 55.4, 47.1, 40.6, 35.3. **HRMS m/z (ESI):** calculated for $C_{11}H_{14}N_1O_3$ $[M-H]^-$: 208.0979, found 208.0980. $[\alpha]_D^{25}$ = –18.3 (*c* = 0.46, 1 M HCl).

The enantiomeric excess was determined by chiral HPLC analysis on Chirabiotic T2 column. Conditions: MeOH/water = 90:10, flow rate = 1.0 mL/min, uv-vis detection at λ = 220 nm, t_{R1} = 20.9 min (minor), t_{R2} = 24.4 min (major).

(*S*)-3-Amino-2-(4-(trifluoromethyl)benzyl)propanoic acid (4f)

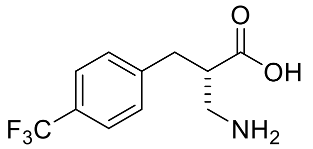

The reaction was performed according to the general procedure and after purification process afford 47.1 mg (95%, 985:1.5 e.r.) of **4a** as a white solid: **^{1}H NMR (501 MHz, D_2O)** δ 7.69 (d, *J* = 8.0 Hz, 2H), 7.44 (d, *J* = 8.0 Hz, 2H), 3.14–3.00 (m, 3H), 2.96 (dd, *J* = 13.7, 7.1 Hz, 1H), 2.90–2.83 (m, 1H). **^{13}C NMR (126 MHz, D_2O)** δ 179.2, 142.9, 129.4, 125.4 (q, *J* = 4.0 Hz), 46.8, 40.7, 36.0. (other signals not detected or observed) **^{19}F NMR (471 MHz, D_2O)** δ –62.14. **HRMS m/z (ESI):**

calculated for $C_{10}H_{13}N_1O_2F_3$ [M+H]$^+$: 248.0893, found 248.0896. $[\alpha]_D^{25}$ = –18.3 (*c* = 0.86, 1 M HCl).

(*S*)-3-Benzamido-2-(4-(trifluoromethyl)benzyl)propanoic acid (10f)

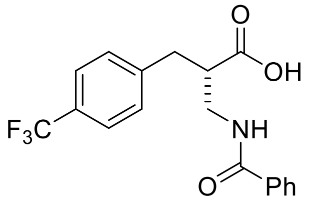

10f was obtained through the general procedure for derivatization. **^{1}H NMR (501 MHz, Methanol-*d*$_4$)** δ 7.77 (d, *J* = 7.6 Hz, 2H), 7.59–7.50 (m, 3H), 7.47–7.41 (m, 4H), 3.62 (d, *J* = 6.9 Hz, 2H), 3.14 (p, *J* = 6.9 Hz, 1H), 3.07 (dd, *J* = 13.7, 8.5 Hz, 1H), 2.98 (dd, *J* = 13.7, 6.0 Hz, 1H). **^{13}C NMR (126 MHz, Methanol-*d*$_4$)** δ 177.0, 170.5, 145.1, 135.6, 132.7, 130.7, 129.7 (d, *J* = 32.3 Hz), 129.5, 128.3, 126.3 (q, *J* = 3.8 Hz), 48.2, 42.7, 36.9. (other signals not detected or observed) **^{19}F NMR (471 MHz, Methanol-*d*$_4$)** δ –63.92. **HRMS m/z (ESI):** calculated for $C_{18}H_{15}F_3N_1O_3$ [M-H]$^-$: 350.1010, found 350.1013.

The enantiomeric excess was determined by chiral HPLC analysis on IA column. Conditions: heptane/isopropanol/trifluoroacetic acid = 90:10:0.1, flow rate = 0.5 mL/min, uv-vis detection at λ = 220 nm, t_{R1} = 40.0 min (major), t_{R2} = 45.9 min (minor).

(*S*)-3-Amino-2-(4-fluorobenzyl)propanoic acid (4g)

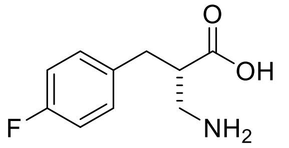

The reaction was performed according to the general procedure and after purification process afford 38.6 mg (98%, 98:2 e.r.) of **4g** as a white solid: **^{1}H NMR (501 MHz, D$_2$O)** δ 7.31–7.22 (m, 2H), 7.10 (t, *J* = 8.8 Hz, 2H), 3.09 (dd, *J* = 12.8, 9.4 Hz, 1H), 3.05–3.00 (m, 1H), 2.96 (dd, *J* = 13.8, 7.2 Hz, 1H), 2.91–2.78 (m, 2H). **^{13}C NMR (126 MHz, D$_2$O)** δ 179.4, 161.5 (d, *J* = 241.7 Hz), 134.2 (d, *J* = 3.1 Hz), 130.5 (d, *J* = 8.1 Hz), 115.2 (d, *J* = 21.4 Hz), 47.1, 40.7, 35.4. **^{19}F NMR (471 MHz, D$_2$O)** δ –117.19. **HRMS m/z (ESI):** calculated for $C_{10}H_{11}N_1O_2F_1$ [M-H]$^-$: 196.0779, found 196.0782. $[\alpha]_D^{25}$ = –18.2 (*c* = 0.37, 1 M HCl).

The enantiomeric excess was determined by chiral HPLC analysis on Chirabiotic T2 column. Conditions: MeOH/water = 90:10, flow rate = 1.0 mL/min, uv-vis detection at λ = 220 nm, t_{R1} = 16.7 min (minor), t_{R2} = 18.8 min (major).

(*S*)-3-Amino-2-(4-chlorobenzyl)propanoic acid (4h)

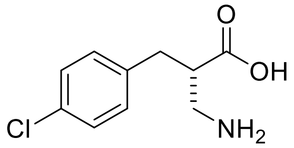

The reaction was performed according to the general procedure and after purification process afford 41.8 mg (98%, 98:2 e.r.) of **4h** as a white solid: **^{1}H NMR (501 MHz, D_2O)** δ 7.41–7.34 (m, 2H), 7.29–7.21 (m, 2H), 3.13–3.06 (m, 1H), 3.06–2.93 (m, 2H), 2.91–2.78 (m, 2H). **^{13}C NMR (126 MHz, D_2O)** δ 179.3, 137.1, 131.8, 130.4, 128.5, 47.0, 40.7, 35.5. **HRMS m/z (ESI):** calculated for $C_{10}H_{11}Cl_1N_1O_2$ [M-H]$^-$: 212.0484, found 212.0485. $[\alpha]_D^{25}$ = –34.2 (*c* = 0.37, 1 M HCl).

The enantiomeric excess was determined by chiral HPLC analysis on Chirabiotic T2 column. Conditions: MeOH/water = 90:10, flow rate = 1.0 mL/min, uv-vis detection at λ = 220 nm, t_{R1} = 18.9 min (minor), t_{R2} = 21.0 min (major).

(*S*)-3-Amino-2-(3-chlorobenzyl)propanoic acid (4i)

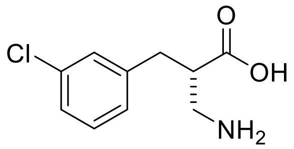

The reaction was performed according to the general procedure and after purification process afford 41.2 mg (96%, 98:2 e.r.) of **4i** as a white solid: **^{1}H NMR (501 MHz, D_2O)** δ 7.37–7.30 (m, 3H), 7.21 (dt, *J* = 6.9, 1.8 Hz, 1H), 3.10 (dd, *J* = 12.7, 9.2 Hz, 1H), 3.05–2.94 (m, 2H), 2.91–2.80 (m, 2H). **^{13}C NMR (126 MHz, D_2O)** δ 179.2, 140.6, 133.6, 130.1, 128.8, 127.3, 126.7, 46.8, 40.6, 35.8. **HRMS m/z (ESI):** calculated for $C_{10}H_{11}Cl_1N_1O_2$ [M-H]$^-$: 212.0484, found 212.0486. $[\alpha]_D^{25}$ = –20.0 (*c* = 0.50, 1 M HCl).

The enantiomeric excess was determined by chiral HPLC analysis on Chirabiotic T2 column. Conditions: MeOH/water = 90:10, flow rate = 1.0 mL/min, uv-vis detection at λ = 220 nm, t_{R1} = 15.7 min (minor), t_{R2} = 19.1 min (major).

(*S*)-3-Amino-2-(2-chlorobenzyl)propanoic acid (4j)

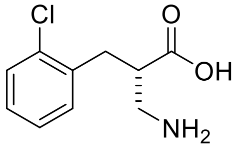

The reaction was performed according to the general procedure and after purification process afford 42.3 mg (99%, 97:3 e.r.) of **4j** as a white solid: **^{1}H NMR (501 MHz, D_2O)** δ 7.52–7.43 (m, 1H), 7.35–7.25 (m, 3H), 3.25–3.16 (m, 1H), 3.16–3.03 (m, 2H), 3.03–2.93 (m, 2H). **^{13}C NMR (126 MHz, D_2O)** δ 178.8, 135.8, 133.6, 131.3, 129.5, 128.5, 127.2, 45.3, 40.7, 33.8. **HRMS m/z (ESI):** calculated for $C_{10}H_{11}Cl_1N_1O_2$ [M-H]$^-$: 212.0484, found 212.0485. $[\alpha]_D^{25}$ = –39.0 (*c* = 0.46, 1 M HCl).

The enantiomeric excess was determined by chiral HPLC analysis on Chirabiotic T2 column. Conditions: MeOH/water = 90:10, flow rate = 1.0 mL/min, uv-vis detection at λ = 220 nm, t_{R1} = 17.6 min (minor), t_{R2} = 20.0 min (major).

(*S*)-3-Amino-2-(naphthalen-1-ylmethyl)propanoic acid (4k)

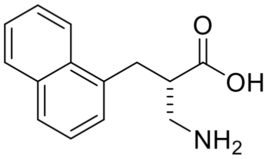

The reaction was performed according to the general procedure and after purification process afford 43.3 mg (94%, 96:4 e.r.) of **4k** as a white solid: **^{1}H NMR (501 MHz, D_2O)** δ 8.10 (d, *J* = 8.4 Hz, 1H), 7.97 (d, *J* = 7.4 Hz, 1H), 7.87 (d, *J* = 8.2 Hz, 1H), 7.61 (dt, *J* = 23.6, 6.9 Hz, 2H), 7.51–7.46 (m, 1H), 7.40 (d, *J* = 6.1 Hz, 1H), 3.54 (dd, *J* = 14.0, 7.0 Hz, 1H), 3.37–3.19 (m, 3H), 3.13 (dd, *J* = 12.9, 3.5 Hz, 1H). **^{13}C NMR (126 MHz, D_2O)** δ 176.2, 133.7, 133.3, 131.2, 129.0, 127.8, 127.6, 126.7, 126.2, 125.7, 123.3, 43.9, 39.9, 32.5. **HRMS m/z (ESI):** calculated for $C_{14}H_{16}N_1O_2$ $[M+H]^+$: 230.1176, found 230.1177. $[\alpha]_D^{25}$ = –38.2 (*c* = 0.50, 1 M HCl).

The enantiomeric excess was determined by chiral HPLC analysis on Chirabiotic T2 column. Conditions: MeOH/water = 50:50, flow rate = 0.5 mL/min, uv-vis detection at λ = 220 nm, t_{R1} = 48.3 min (minor), t_{R2} = 61.0 min (major).

(*S*)-2-(Aminomethyl)-4-(thiophen-2-yl)butanoic acid (4l)

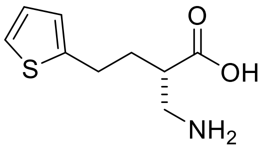

The reaction was performed according to the general procedure and after purification process afford 39.4 mg (99%, 96:4 e.r.) of **4l** as a white solid: **^{1}H NMR (501 MHz, D_2O)** δ 7.31 (d, *J* = 5.1 Hz, 1H), 7.07–7.00 (m, 1H), 6.99–6.93 (m, 1H), 3.22 (dd, *J* = 13.0, 8.9 Hz, 1H), 3.14 (dd, *J* = 12.9, 4.7 Hz, 1H), 3.01–2.88 (m, 2H), 2.75–2.65 (m, 1H), 2.13–2.00 (m, 1H), 2.00–1.90 (m, 1H). **^{13}C NMR (126 MHz, D_2O)** δ 178.8, 144.3, 127.2, 124.9, 124.0, 43.6, 40.6, 31.7, 26.5. **HRMS m/z (ESI):** calculated for $C_9H_{12}N_1O_2S_1$ $[M-H]^-$: 198.0594, found 198.0597. $[\alpha]_D^{25}$ = –21.8 (*c* = 0.46, 1 M HCl).

The enantiomeric excess was determined by chiral HPLC analysis on Chirabiotic T2 column. Conditions: MeOH/water = 90:10, flow rate = 1.0 mL/min, uv-vis detection at λ = 220 nm, t_{R1} = 19.0 min (minor), t_{R2} = 22.3 min (major).

(*S*)-3-Amino-2-(4-chlorophenyl)propanoic acid (4m)

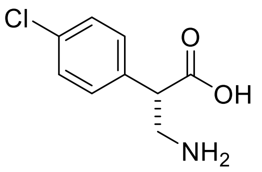

Following the general procedure, the reaction using 3 mol% catalyst **IDPi-3h** (6.0 μmol, 16.5 mg) after purification process afford 12.1 mg (30%, 95:5 e.r.) of **4m** as a white solid: **^{1}H NMR (501 MHz, D_2O)** δ 7.51–7.43 (m, 2H), 7.37–7.31 (m, 2H), 3.96 (t, *J* = 7.4 Hz, 1H), 3.56 (dd, *J* = 12.9, 7.9 Hz, 1H), 3.34 (dd, *J* = 13.1, 7.2 Hz, 1H). **^{13}C NMR (126 MHz, D_2O)** δ 176.1, 134.4, 133.6, 129.7, 129.3, 49.4, 41.5. **HRMS m/z (ESI):** calculated for $C_9H_{11}Cl_1N_1O_2$ $[M+H]^+$: 200.0473, found 200.0475. $[\alpha]_D^{25}$ = –98.4 (*c* = 0.33, 1 M HCl).

The enantiomeric excess was determined by chiral HPLC analysis on Chirabiotic T2 column. Conditions: MeOH/water = 50:50, flow rate = 0.5 mL/min, uv-vis detection at λ = 220 nm, t_{R1} = 26.9 min (minor), t_{R2} = 45.4 min (major).

(*S*)-3-Amino-2-(3-chlorophenyl)propanoic acid (4n)

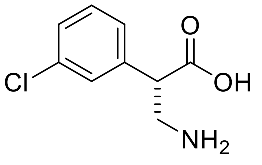

Following the general procedure, the reaction using 3 mol% catalyst **IDPi-3h** (6.0 μmol, 16.5 mg) after purification process afford 31.1 mg (78%, 95:5 e.r.) of **4n** as a white solid: **^{1}H NMR (501 MHz, D_2O)** δ 7.48–7.42 (m, 3H), 7.34–7.29 (m, 1H), 4.08 (t, *J* = 7.5 Hz, 1H), 3.63 (dd, *J* = 13.2, 7.8 Hz, 1H), 3.39 (dd, *J* = 13.2, 7.2 Hz, 1H). **^{13}C NMR (126 MHz, D_2O)** δ 174.6, 136.7, 134.5, 130.9, 128.7, 128.3, 126.7, 48.6, 41.0. **HRMS m/z (ESI):** calculated for $C_9H_{11}Cl_1N_1O_2$ $[M+H]^+$: 200.0473, found 200.0475. $[\alpha]_D^{25}$ = –94.0 (*c* = 0.42, 1 M HCl).

The enantiomeric excess was determined by chiral HPLC analysis on Chirabiotic T2 column. Conditions: MeOH/water = 90:10, flow rate = 1.0 mL/min, uv-vis detection at λ = 220 nm, t_{R1} = 15.8 min (minor), t_{R2} = 24.7 min (major).

(*S*)-3-Amino-2-(2-chlorophenyl)propanoic acid (4o)

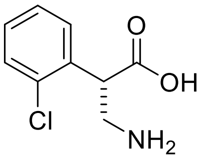

Following the general procedure, the reaction using 3 mol% catalyst **IDPi-3h** (6.0 μmol, 16.5 mg) after purification process afford 18.4 mg (46%, 96:4 e.r.) of **4o** as a white solid: **^{1}H NMR (501 MHz, D_2O)** δ 7.56–7.49 (m, 1H), 7.40–7.33 (m, 3H), 4.26 (dd, *J* = 8.3, 6.5 Hz, 1H), 3.56 (dd, *J* = 12.9, 8.3 Hz, 1H), 3.33 (dd, *J* = 12.9, 6.5 Hz, 1H). **^{13}C NMR (126 MHz, D_2O)** δ 177.4, 134.7, 133.7, 130.1, 130.0, 129.5, 127.8, 48.3, 40.9. **HRMS m/z (ESI):** calculated for $C_9H_{11}Cl_1N_1O_2$ $[M+H]^+$: 200.0473, found 200.0476

(*S*)-3-benzamido-2-(2-chlorophenyl)propanoic acid (10o)

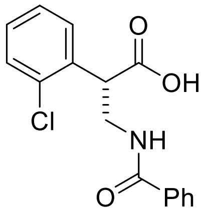

10o was obtained through the general procedure for derivatization. **^{1}H NMR (501 MHz, Methanol-*d*$_4$)** δ 7.73–7.64 (m, 2H), 7.52–7.46 (m, 1H), 7.46–7.38 (m, 4H), 7.32–7.23 (m, 2H), 4.62 (t, *J* = 7.5 Hz, 1H), 4.02 (dd, *J* = 13.5, 7.1 Hz, 1H), 3.75 (dd, *J* = 13.5, 7.9 Hz, 1H). **^{13}C NMR (126 MHz, Methanol-*d*$_4$)** δ 175.2, 170.5, 136.6, 135.6, 135.6, 132.6, 130.8, 130.7, 130.0, 129.5, 128.4, 128.2, 48.4, 42.8. **HRMS m/z (ESI):** calculated for $C_{16}H_{13}Cl_1N_1O_3$ $[M-H]^-$: 302.0589, found 302.0591.

The enantiomeric excess was determined by chiral HPLC analysis on IA column. Conditions: heptane/isopropanol/trifluoroacetic acid = 90:10:0.1, flow rate = 1.0 mL/min, uv-vis detection at λ = 220 nm, t_{R1} = 19.0 min (major), t_{R2} = 22.6 min (minor).

(*S*)-3-amino-2-(4-bromophenyl)propanoic acid (4p)

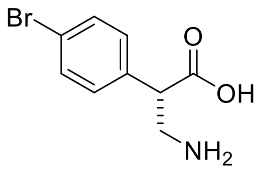

Following the general procedure, the reaction using 3 mol% catalyst **IDPi-3h** (6.0 µmol, 16.5 mg) after purification process afford 27.9 mg (57%, 96:4 e.r.) of **4p** as a white solid: **^{1}H NMR (501 MHz, D$_2$O)** δ 7.62 (d, *J* = 8.2 Hz, 2H), 7.28 (d, *J* = 8.4 Hz, 2H), 3.93 (t, *J* = 7.5 Hz, 1H), 3.55 (dd, *J* = 12.6, 8.4 Hz, 1H), 3.33 (dd, *J* = 13.2, 7.3 Hz, 1H). **^{13}C NMR (126 MHz, D$_2$O)** δ 176.2, 135.1, 132.3, 130.0, 121.7, 49.6, 41.5. **HRMS m/z (ESI):** calculated for $C_9H_{11}Br_1N_1O_2$ $[M+H]^+$: 243.9968, found 243.9968. $[\alpha]_D^{25}$ = –91.1 (*c* = 0.36, 1 M HCl).

The enantiomeric excess was determined by chiral HPLC analysis on Chirabiotic T2 column. Conditions: MeOH/water = 90:10, flow rate = 1.0 mL/min, uv-vis detection at λ = 220 nm, t_{R1} = 20.8 min (minor), t_{R2} = 41.3 min (major).

(*S*)-3-amino-2-(3,4-dichlorophenyl)propanoic acid (4q)

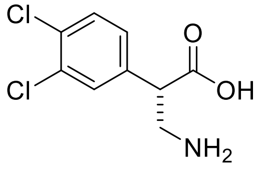

Following the general procedure, the reaction using 3 mol% catalyst **IDPi-3h** (6.0 µmol, 16.5 mg) after purification process afford 23.7 mg (51%, 96.5:3.5 e.r.) of **4q** as a white solid: **^{1}H NMR (501 MHz, D$_2$O)** δ 7.64–7.55 (m, 2H), 7.33–7.27 (m, 1H), 4.09 (t, *J* = 7.5 Hz, 1H), 3.64 (dd, *J* = 13.3, 8.0 Hz, 1H), 3.39 (dd, *J* = 13.3, 7.1 Hz, 1H). **^{13}C NMR (126 MHz, D$_2$O)** δ 174.2, 135.0, 132.6, 132.2, 131.2,

130.2, 128.1, 48.0, 40.8. **HRMS m/z (ESI):** calculated for $C_9H_{10}Cl_2N_1O_2$ $[M+H]^+$: 234.0083, found 234.0085. $[\alpha]_D^{25}$ = –29.3 (c = 0.23, 1 M HCl).

The enantiomeric excess was determined by chiral HPLC analysis on Chirabiotic T2 column. Conditions: MeOH/water = 90:10, flow rate = 1.0 mL/min, uv-vis detection at λ = 220 nm, t_{R1} = 18.1 min (minor), t_{R2} = 28.3 min (major).

(*S*)-3-Amino-2-methylpropanoic acid (4r)

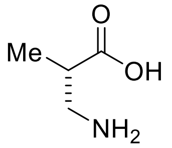

The reaction was performed according to the general procedure and after purification process afford 17.9 mg (87%, 81.5:18.5 e.r.) of **4r** as a white solid: **^{1}H NMR (501 MHz, D_2O)** δ 3.10 (dd, J = 12.7, 8.5 Hz, 1H), 3.03 (dd, J = 12.8, 5.2 Hz, 1H), 2.67–2.54 (m, 1H), 1.19 (d, J = 7.3 Hz, 3H). **^{13}C NMR (126 MHz, D_2O)** δ 181.7, 42.4, 39.3, 15.1. **HRMS m/z (ESI):** calculated for $C_4H_{10}N_1O_2$ $[M+H]^+$: 104.0706, found 104.0707. $[\alpha]_D^{25}$ = +7.6 (c = 0.14, H_2O). [Lit.[9] $[\alpha]_D^{25}$ = +17.0 (c = 1.0, H_2O)]

(*S*)-3-Benzamido-2-methylpropanoic acid (10r)

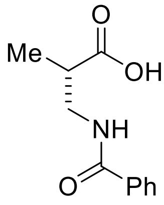

10r was obtained through the general procedure for derivatization. **^{1}H NMR (501 MHz, $CDCl_3$)** δ 9.57 (br s, 1H), 7.78–7.68 (m, 2H), 7.51–7.44 (m, 1H), 7.43–7.35 (m, 2H), 6.94 (t, J = 6.2 Hz, 1H), 3.77–3.66 (m, 1H), 3.57–3.45 (m, 1H), 2.90–2.78 (m, 1H), 1.26 (d, J = 7.2 Hz, 3H). **^{13}C NMR (126 MHz, $CDCl_3$)** δ 180.2, 168.2, 134.2, 131.8, 128.7, 127.1, 42.2, 39.6, 15.0. **HRMS m/z (ESI):** calculated for $C_{11}H_{13}N_1O_3Na_1$ $[M+Na]^+$: 230.0788, found 230.0790.

The enantiomeric excess was determined by chiral HPLC analysis on ID-3 column. Conditions: heptane/isopropanol/trifluoroacetic acid = 80 : 20 : 0.1, flow rate = 0.5 mL/min, uv-vis detection at λ = 220 nm, t_{R1} = 14.6 min (minor), t_{R2} = 16.0 min (major).

(*S*)-2-(Aminomethyl)butanoic acid (4s)

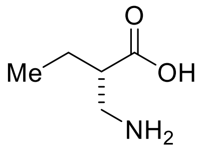

The reaction was performed according to the general procedure and after purification process afford 20.8 mg (89%, 90:10 e.r.) of **4s** as a white solid: **^{1}H NMR (501 MHz, D_2O)** δ 3.15 (dd, J = 12.8, 8.7 Hz, 1H), 3.06 (dd, J = 12.8, 5.0 Hz, 1H), 2.55–2.42 (m, 1H), 1.62 (p, J = 7.3 Hz, 2H), 0.92 (t, J = 7.5 Hz, 3H). **^{13}C NMR (126**

MHz, D_2O) δ 180.8, 46.6, 40.8, 23.1, 10.5. **HRMS m/z (ESI):** calculated for $C_5H_{10}N_1O_2$ $[M-H]^-$: 116.0717, found 116.0718. $[\alpha]_D^{25}$ = –6.5 (c = 0.12, H_2O). [Lit.[9] $[\alpha]_D^{25}$ = –6.8 (c = 0.4, H_2O)]

(*S*)-2-(Benzamidomethyl)butanoic acid (10s)

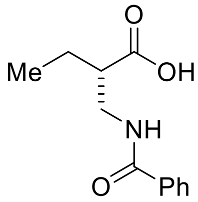

10s was obtained through the general procedure for derivatization. **^{1}H NMR (501 MHz, $CDCl_3$)** δ 7.76–7.71 (m, 2H), 7.51–7.44 (m, 1H), 7.43–7.35 (m, 2H), 6.81 (t, J = 6.1 Hz, 1H), 3.78–3.67 (m, 1H), 3.63–3.53 (m, 1H), 2.74–2.64 (m, 1H), 1.82–1.70 (m, 1H), 1.70–1.57 (m, 1H), 1.01 (t, J = 7.5 Hz, 3H). **^{13}C NMR (126 MHz, $CDCl_3$)** δ 179.8, 168.1, 134.3, 131.8, 128.7, 127.1, 46.7, 40.5, 23.1, 11.7. **HRMS m/z (EI):** calculated for $C_{12}H_{15}N_1O_3$ $[M]^+$: 221.1046, found 221.1045.

The enantiomeric excess was determined by chiral HPLC analysis on ID-3 column. Conditions: heptane/isopropanol/trifluoroacetic acid = 90:10:0.1, flow rate = 1.0 mL/min, uv-vis detection at λ = 220 nm, t_{R1} = 14.5 min (minor), t_{R2} = 17.6 min (major).

(*S*)-2-(Aminomethyl)pentanoic acid (4t)

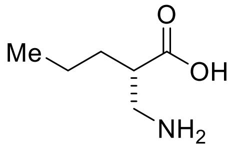

The reaction was performed according to the general procedure and after purification process afford 23.7 mg (90%, 94:6 e.r.) of **4t** as a white solid: **^{1}H NMR (501 MHz, D_2O)** δ 3.18–3.03 (m, 2H), 2.65–2.48 (m, 1H), 1.65–1.48 (m, 2H), 1.39–1.28 (m, 2H), 0.92 (t, J = 7.3 Hz, 3H). **^{13}C NMR (126 MHz, D_2O)** δ 180.9, 45.0, 41.0, 32.0, 19.6, 13.2. **HRMS m/z (ESI):** calculated for $C_6H_{14}N_1O_2$ $[M+H]^+$: 132.1019, found 132.1021. $[\alpha]_D^{25}$ = –1.1 (c = 0.37, H_2O).

(*S*)-2-(Benzamidomethyl)pentanoic acid (10t)

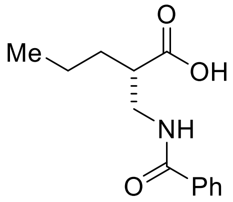

10t was obtained through the general procedure for derivatization. **^{1}H NMR (501 MHz, $CDCl_3$)** δ 7.77–7.69 (m, 2H), 7.51–7.45 (m, 1H), 7.43–7.36 (m, 2H), 6.78 (t, J = 6.1 Hz, 1H), 3.80–3.69 (m, 1H), 3.61–3.50 (m, 1H), 2.83–2.74 (m, 1H), 1.77–1.67 (m, 1H), 1.61–1.52 (m, 1H), 1.49–1.40 (m, 2H), 0.94 (t, J = 7.3 Hz, 3H). **^{13}C NMR (126 MHz, $CDCl_3$)** δ 180.0, 168.1, 134.3, 131.8, 128.7, 127.1, 44.9, 40.8, 32.0, 20.4, 14.1. **HRMS m/z (EI):** calculated for $C_{13}H_{17}N_1O_3$ $[M]^+$: 235.1203, found 235.1203.

The enantiomeric excess was determined by chiral HPLC analysis on IA column. Conditions: heptane/isopropanol/trifluoroacetic acid = 90:10:0.1, flow rate = 1.0 mL/min, uv-vis detection at λ = 220 nm, t_{R1} = 10.1 min (major), t_{R2} = 11.7 min (minor).

(*S*)-2-(Aminomethyl)hexanoic acid (4u)

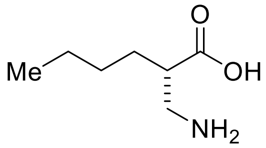

The reaction was performed according to the general procedure and after purification process afford 27.8 mg (96%, 95:5 e.r.) of **4a** as a white solid: **^{1}H NMR (501 MHz, D_2O)** δ 3.17–3.03 (m, 2H), 2.58–2.50 (m, 1H), 1.66–1.51 (m, 2H), 1.38–1.25 (m, 4H), 0.89 (t, *J* = 7.0 Hz, 3H). **^{13}C NMR (126 MHz, D_2O)** δ 181.1, 45.3, 41.1, 29.6, 28.4, 21.9, 13.1. **HRMS m/z (ESI):** calculated for $C_7H_{14}N_1O_2$ $[M\text{-}H]^-$: 144.1030, found 144.1031. $[\alpha]_D^{25}$ = –3.5 (*c* = 0.33, H_2O).

For 20 mmol scale: The reaction was performed in 20 mmol scale of **2a** with 0.1 mol **IDPi-3h** (0.5 mol%) according to the general procedure, which afforded 2.84 g (98%, 95:4 e.r.) of **4u** as a white solid after purification. Catalyst **IDPi-3h** (259 mg, 95%) was recovered from the organic phase after column chromatography on silica and acidified by HCl (see a detailed procedure in the paragraph of Synthesis and Characterization of Catalysts).

(*S*)-2-(Benzamidomethyl)hexanoic acid (10u)

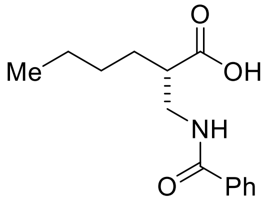

10u was obtained through the general procedure for derivatization. **^{1}H NMR (501 MHz, $CDCl_3$)** δ 7.74 (dd, *J* = 8.4, 1.4 Hz, 2H), 7.52–7.45 (m, 1H), 7.44–7.39 (m, 2H), 6.70 (t, *J* = 6.3 Hz, 1H), 3.82–3.72 (m, 1H), 3.61–3.52 (m, 1H), 2.83–2.74 (m, 1H), 1.80–1.70 (m, 1H), 1.65–1.55 (m, 1H), 1.43–1.31 (m, 4H), 0.91 (t, *J* = 7.1 Hz, 3H). **^{13}C NMR (126 MHz, $CDCl_3$)** δ 179.5, 167.9, 134.4, 131.8, 128.8, 127.1, 45.0, 40.7, 29.6, 29.3, 22.7, 14.0. **HRMS m/z (ESI):** calculated for $C_{14}H_{18}N_1O_3$ $[M\text{-}H]^-$: 248.1292, found 148.1294.

The enantiomeric excess was determined by chiral HPLC analysis on IA column. Conditions: heptane/isopropanol/trifluoroacetic acid = 90:10:0.1, flow rate = 0.5 mL/min, uv-vis detection at λ = 220 nm, t_{R1} = 20.7 min (major), t_{R2} = 23.7 min (minor).

(*S*)-2-(Aminomethyl)-4-methylpentanoic acid (4v)

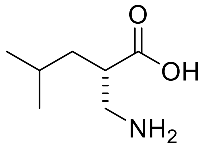

The reaction was performed according to the general procedure and after purification process afford 27.2 mg (94%, 95:5 e.r.) of **4v** as a white solid: **^{1}H NMR (501 MHz, D$_2$O)** δ 3.17–3.04 (m, 2H), 2.71–2.58 (m, 1H), 1.67–1.50 (m, 2H), 1.40–1.27 (m, 1H), 0.92 (t, J = 6.0 Hz, 6H). **^{13}C NMR (126 MHz, D$_2$O)** δ 180.8, 43.4, 41.2, 39.0, 25.3, 21.8, 21.5. **HRMS m/z (ESI):** calculated for $C_7H_{14}N_1O_2$ [M-H]$^-$: 144.1030, found 144.1031. $[\alpha]_D^{25}$ = –10.3 (c = 0.33, H_2O). [Lit.[10] $[\alpha]_D^{25}$ = –4 (c = 1.0, H_2O)]

(*S*)-2-(Benzamidomethyl)-4-methylpentanoic acid (10v)

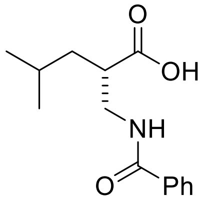

10v was obtained through the general procedure for derivatization. **^{1}H NMR (501 MHz, CDCl$_3$)** δ 9.08 (br s, 1H), 7.76–7.68 (m, 2H), 7.50–7.42 (m, 1H), 7.41–7.32 (m, 2H), 6.89 (t, J = 6.1 Hz, 1H), 3.75–3.66 (m, 1H), 3.58–3.48 (m, 1H), 2.88–2.76 (m, 1H), 1.76–1.67 (m, 1H), 1.67–1.58 (m, 1H), 1.41–1.31 (m, 1H), 0.92 (dd, J = 6.6, 1.7 Hz, 6H). **^{13}C NMR (126 MHz, CDCl$_3$)** δ 180.3, 168.2, 134.2, 131.8, 128.7, 127.1, 43.4, 41.2, 38.9, 26.0, 22.6, 22.5. **HRMS m/z (ESI):** calculated for $C_{14}H_{18}N_1O_3$ [M-H]$^-$: 248.1292, found 148.1294.

The enantiomeric excess was determined by chiral HPLC analysis on AS-3 column. Conditions: heptane/isopropanol/trifluoroacetic acid = 90:10:0.1, flow rate = 0.5 mL/min, uv-vis detection at λ = 220 nm, t_{R1} = 16.8 min (major), t_{R2} = 20.1 min (minor).

(*S*)-2-(Aminomethyl)-5-methylhexanoic acid (4w)

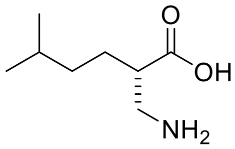

The reaction was performed according to the general procedure and after purification process afford 30.5 mg (96%, 95:5 e.r.) of **4w** as a white solid: **^{1}H NMR (501 MHz, D$_2$O)** δ 3.19–3.02 (m, 2H), 2.57–2.44 (m, 1H), 1.67–1.49 (m, 3H), 1.21 (q, J = 8.2 Hz, 2H), 0.88 (d, J = 6.6 Hz, 6H). **^{13}C NMR (126 MHz, D$_2$O)** δ 181.0, 45.6, 41.2, 35.4, 27.8, 27.3, 21.7, 21.6. **HRMS m/z (ESI):** calculated for $C_8H_{16}N_1O_2$ [M-H]$^-$: 158.1187, found 158.1188. $[\alpha]_D^{25}$ = –3.0 (c = 0.27, H_2O).

(*S*)-2-(Benzamidomethyl)-5-methylhexanoic acid (10w)

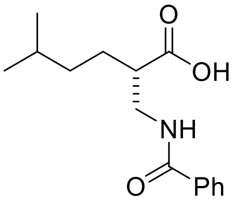

10w was obtained through the general procedure for derivatization. **^{1}H NMR (501 MHz, CDCl$_3$)** δ 7.78–7.67 (m, 2H), 7.52–7.44 (m, 1H), 7.44–7.36 (m, 2H), 6.77 (t, J = 6.1 Hz, 1H), 3.83–3.68 (m, 1H), 3.62–3.48 (m, 1H), 2.80–2.68 (m, 1H), 1.79–1.67 (m, 1H), 1.63–1.49 (m, 2H), 1.36–1.23 (m, 2H), 0.88 (dd, J = 6.6, 2.4 Hz, 6H). **^{13}C NMR (126 MHz, CDCl$_3$)** δ 180.0, 168.1, 134.4, 131.8, 128.7, 127.1, 45.4, 40.8, 36.2, 28.1, 27.8, 22.6, 22.5. **HRMS m/z (ESI):** calculated for $C_{15}H_{20}N_1O_3$ [M-H]$^-$: 262.1449, found 262.1450.

The enantiomeric excess was determined by chiral HPLC analysis on OJ-3 column. Conditions: heptane/isopropanol/trifluoroacetic acid = 90:10:0.1, flow rate = 0.5 mL/min, uv-vis detection at λ = 220 nm, t_{R1} = 6.3 min (major), t_{R2} = 7.6 min (minor).

(*S*)-3-Amino-2-(cyclopentylmethyl)propanoic acid (4x)

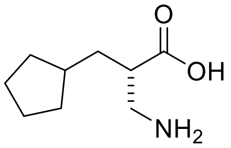

The reaction was performed according to the general procedure and after purification process afford 32.6 mg (95% ee, 95.5:4.5 e.r.) of **4x** as a white solid: **^{1}H NMR (501 MHz, D$_2$O)** δ 3.27–3.13 (m, 2H), 2.84–2.71 (m, 1H), 1.90–1.70 (m, 4H), 1.67–1.47 (m, 5H), 1.19–1.06 (m, 2H). **^{13}C NMR (126 MHz, D$_2$O)** δ 178.7, 43.1, 40.5, 37.1, 35.8, 32.1, 32.0, 24.6, 24.6. **HRMS m/z (ESI):** calculated for $C_9H_{18}N_1O_2$ [M+H]$^+$: 172.1332, found 172.1334. $[\alpha]_D^{25}$ = –20.0 (c = 0.45, H_2O).

(*S*)-3-Benzamido-2-(cyclopentylmethyl)propanoic acid (10x)

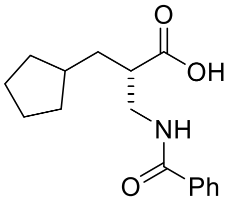

10x was obtained through the general procedure for derivatization. **^{1}H NMR (501 MHz, CDCl$_3$)** δ 7.78–7.70 (m, 2H), 7.52–7.45 (m, 1H), 7.45–7.37 (m, 2H), 6.71 (t, J = 5.3 Hz, 1H), 3.81–3.72 (m, 1H), 3.62–3.52 (m, 1H), 2.87–2.78 (m, 1H), 1.99–1.88 (m, 1H), 1.88–1.74 (m, 3H), 1.66–1.48 (m, 5H), 1.17–1.05 (m, 2H). **^{13}C NMR (126 MHz, CDCl$_3$)** δ 180.1, 168.0, 134.5, 131.9, 128.9, 127.2, 44.6, 41.1, 38.0, 36.3, 33.0, 32.9, 25.3, 25.3. **HRMS m/z (ESI):** calculated for $C_{16}H_{20}N_1O_3$ [M-H]$^-$: 274.1449, found 274.1453.

The enantiomeric excess was determined by chiral HPLC analysis on OJ-3 column. Conditions: heptane/isopropanol/trifluoroacetic acid = 90:10:0.1, flow rate = 1.0 mL/min, uv-vis detection at λ = 220 nm, t_{R1} = 3.9 min (major), t_{R2} = 4.7 min (minor).

(*S*)-2-(Aminomethyl)-5-methoxypentanoic acid (4y)

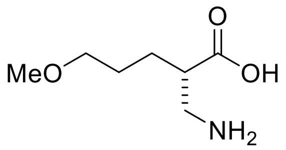

The reaction was performed according to the general procedure and after purification process afford 26.7 mg (83%, 94.5:5.5 e.r.) of **4y** as a white solid: **^{1}H NMR (501 MHz, D_2O)** δ 3.55–3.46 (m, 2H), 3.36 (s, 3H), 3.18–3.04 (m, 2H), 2.61–2.50 (m, 1H), 1.68–1.55 (m, 4H). **^{13}C NMR (126 MHz, D_2O)** δ 180.4, 72.1, 57.6, 45.0, 41.0, 26.4, 26.0. **HRMS m/z (ESI):** calculated for $C_7H_{14}N_1O_3$ $[M\text{-}H]^-$: 160.0979, found 160.0980. $[\alpha]_D^{25}$ = –1.7 (*c* = 0.27, H_2O).

(*S*)-2-(Benzamidomethyl)-5-methoxypentanoic acid (10y)

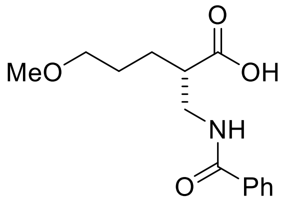

10y was obtained through the general procedure for derivatization. **^{1}H NMR (501 MHz, $CDCl_3$)** δ 7.77–7.71 (m, 2H), 7.49–7.43 (m, 1H), 7.41–7.35 (m, 2H), 6.96 (t, *J* = 6.1 Hz, 1H), 3.76–3.65 (m, 1H), 3.61–3.51 (m, 1H), 3.41 (t, *J* = 5.8 Hz, 2H), 3.32 (s, 3H), 2.82–2.70 (m, 1H), 1.81–1.59 (m, 4H). **^{13}C NMR (126 MHz, $CDCl_3$)** δ 178.7, 168.1, 134.3, 131.7, 128.7, 127.1, 72.6, 58.7, 45.0, 41.1, 27.0, 26.9. **HRMS m/z (ESI):** calculated for $C_{14}H_{18}N_1O_4$ $[M\text{-}H]^-$: 264.1241, found 264.1243.

The enantiomeric excess was determined by chiral HPLC analysis on OJ-3 column. Conditions: heptane/isopropanol/trifluoroacetic acid = 90:10:0.1, flow rate = 0.5 mL/min, uv-vis detection at λ = 220 nm, t_{R1} = 11.3 min (major), t_{R2} = 13.6 min (minor).

(*S*)-2-(Aminomethyl)-5-methylhex-5-enoic acid (4z)

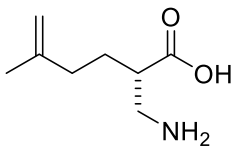

The reaction was performed according to the general procedure and after purification process afford 28.6 mg (91%, 96:4 e.r.) of **4z** as a white solid: **^{1}H NMR (501 MHz, D_2O)** δ 4.81 (s, 1H), 4.77 (s, 1H), 3.19–3.06 (m, 2H), 2.59–2.49 (m, 1H), 2.08 (t, *J* = 7.9 Hz, 2H), 1.82–1.65 (m, 2H), 1.75 (s, 3H). **^{13}C NMR (126 MHz, D_2O)** δ 180.6, 146.6, 110.1, 44.9, 41.0, 34.4, 28.0, 21.4. **HRMS m/z (ESI):** calculated for $C_8H_{14}N_1O_2$ $[M\text{-}H]^-$: 156.1030, found 156.1030. $[\alpha]_D^{25}$ = –1.0 (*c* = 0.19, H_2O).

(*S*)-2-(Benzamidomethyl)-5-methylhex-5-enoic acid (10z)

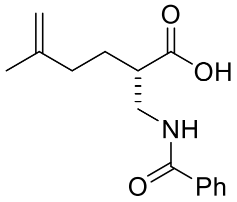

10z was obtained through the general procedure for derivatization. **^{1}H NMR (501 MHz, CDCl$_3$)** δ 9.46 (br s, 1H), 7.77–7.68 (m, 2H), 7.51–7.43 (m, 1H), 7.43–7.34 (m, 2H), 6.82 (t, J = 6.1 Hz, 1H), 4.73 (d, J = 14.1 Hz, 2H), 3.79–3.68 (m, 1H), 3.66–3.56 (m, 1H), 2.80–2.71 (m, 1H), 2.13 (t, J = 7.8 Hz, 2H), 1.94–1.84 (m, 1H), 1.75–1.65 (m, 1H), 1.71 (s, 3H). **^{13}C NMR (126 MHz, CDCl$_3$)** δ 179.9, 168.2, 144.5, 134.3, 131.8, 128.7, 127.1, 111.1, 44.7, 40.7, 35.1, 27.6, 22.4. **HRMS m/z (ESI):** calculated for $C_{15}H_{18}N_1O_3$ [M-H]$^-$: 260.1292, found 260.1293.

The enantiomeric excess was determined by chiral HPLC analysis on IA column. Conditions: heptane/isopropanol/trifluoroacetic acid = 90:10:0.1, flow rate = 0.5 mL/min, uv-vis detection at λ = 220 nm, t_{R1} = 20.7 min (major), t_{R2} = 24.0 min (minor).

(2*S*,4*R*)-2-(Aminomethyl)-4-methylhexanoic acid (4A)

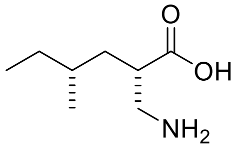

The reaction was performed according to the general procedure and after purification process afford 28.0 mg (88%, 95:5 d.r.) of **4A** as a white solid: **^{1}H NMR (501 MHz, D$_2$O)** δ 3.16–3.01 (m, 2H), 2.67–2.59 (m, 1H), 1.71–1.59 (m, 1H), 1.44–1.28 (m, 2H), 1.28–1.14 (m, 2H), 0.91 (d, J = 6.5 Hz, 3H), 0.87 (t, J = 7.3 Hz, 3H). **^{13}C NMR (126 MHz, D$_2$O)** δ 181.3, 43.9, 41.8, 37.0, 31.8, 29.1, 18.3, 10.4. **HRMS m/z (ESI):** calculated for $C_8H_{18}Cl_1N_1O_2$ [M+H]$^+$: 160.1332, found 160.1334. $[\alpha]_D^{25}$ = –22.6 (c = 0.32, H_2O).

(2*S*,4*R*)-2-(Benzamidomethyl)-4-methylhexanoic acid (10A)

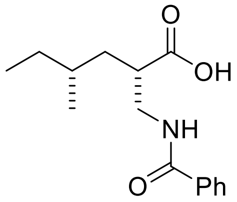

10A was obtained through the general procedure for derivatization. **^{1}H NMR (501 MHz, CDCl$_3$)** δ 7.74 (dd, J = 8.4, 1.3 Hz, 2H), 7.52–7.45 (m, 1H), 7.44–7.36 (m, 2H), 6.71 (t, J = 6.3 Hz, 1H), 3.73 (ddd, J = 13.7, 6.1, 3.9 Hz, 1H), 3.55 (ddd, J = 13.9, 8.6, 6.1 Hz, 1H), 2.93–2.84 (m, 1H), 1.77 (ddd, J = 14.2, 9.0, 5.7 Hz, 1H), 1.57–1.46 (m, 1H), 1.39 (ddd, J = 11.0, 7.6, 5.2 Hz, 1H), 1.30 (ddd, J = 14.0, 8.5, 5.8 Hz, 1H), 1.19 (dt, J = 13.4, 7.4 Hz, 1H), 0.92 (d, J = 6.6 Hz, 3H), 0.88 (t, J = 7.4 Hz, 3H). **^{13}C NMR (126 MHz, CDCl$_3$)** δ 179.9, 168.0, 134.4, 131.8, 128.8, 127.1, 43.2, 41.6, 36.9, 32.3, 29.5, 19.1, 11.2. **HRMS m/z (ESI):** calculated for $C_{15}H_{20}N_1O_3$ [M-H]$^-$: 262.1449, found 262.1449.

The enantiomeric excess was determined by chiral HPLC analysis on AS-3 column. Conditions: heptane/isopropanol/trifluoroacetic acid = 90:10:0.1, flow rate = 1.0 mL/min, uv-vis detection at λ = 220 nm, t_{R1} = 8.2 min (major), t_{R2} = 10.2 min (minor).

(2*S*,4*S*)-2-(Aminomethyl)-4-methylhexanoic acid (4B)

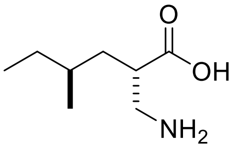

The reaction was performed according to the general procedure and after purification process afford 28.9 mg (91%, 95:5 d.r.) of **4B** as a white solid: **^{1}H NMR (501 MHz, D_2O)** δ 3.08 (d, *J* = 6.8 Hz, 2H), 2.61 (p, *J* = 7.1 Hz, 1H), 1.53–1.34 (m, 4H), 1.23–1.12 (m, 1H), 0.90 (d, *J* = 6.5 Hz, 3H), 0.87 (t, *J* = 7.3 Hz, 3H). **^{13}C NMR (126 MHz, D_2O)** δ 181.5, 43.5, 41.2, 37.1, 31.6, 28.6, 18.3, 10.4. **HRMS m/z (ESI):** calculated for $C_8H_{18}Cl_1N_1O_2$ [M-H]$^-$: 158.1187, found 158.1187. $[\alpha]_D^{25}$ = +5.2 (*c* = 0.27, H_2O).

(2*S*,4*S*)-2-(Benzamidomethyl)-4-methylhexanoic acid (10B)

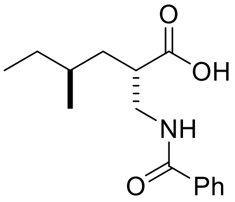

10B was obtained through the general procedure for derivatization. **^{1}H NMR (501 MHz, $CDCl_3$)** δ 7.75 (dd, *J* = 8.4, 1.4 Hz, 2H), 7.53–7.45 (m, 1H), 7.45–7.36 (m, 2H), 6.76 (br s, 1H), 3.75 (ddd, *J* = 13.7, 6.0, 3.8 Hz, 1H), 3.53 (ddd, *J* = 13.7, 8.5, 6.0 Hz, 1H), 2.90–2.82 (m, 1H), 1.58–1.52 (m, 2H), 1.43–1.32 (m, 2H), 1.23–1.14 (m, 1H), 0.93 (d, *J* = 6.5 Hz, 3H), 0.87 (t, *J* = 7.4 Hz, 3H). **^{13}C NMR (126 MHz, $CDCl_3$)** δ 179.4, 167.8, 134.5, 131.7, 128.7, 127.1, 42.9, 40.7, 36.7, 32.2, 29.5, 19.1, 11.3. **HRMS m/z (ESI):** calculated for $C_{15}H_{20}N_1O_3$ [M-H]$^-$: 262.1449, found 262.1450.

The enantiomeric excess was determined by chiral HPLC analysis on IA column. Conditions: heptane/isopropanol/trifluoroacetic acid = 90:10:0.1, flow rate = 0.5 mL/min, uv-vis detection at λ = 220 nm, t_{R1} = 18.0 min (minor), t_{R2} = 19.4 min (major).

(*S*)-Piperidine-3-carboxylic acid (5)

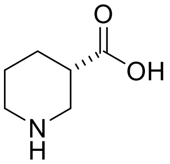

Following the general procedure, the reaction was performed at –60 °C for 3 d. Then the reaction mixture was treated with triethylamine (1.2 mmol, 170 µL, 6 equiv.) at –60 °C and diluted with water (1 mL). The mixture was stirred vigorously for 4 h at rt and then 1 M HCl (0.5 mL) was added. The aqueous phase was separated and the water was removed under reduced pressure. The solid residue was purified by short

column chromatography on silica by eluting with acetone and MeOH (R_f = 0.3 in MeOH). The reaction after purification process afford 21.6 mg (84%, 97:3 e.r.) of **5** as a white solid: **^{1}H NMR (501 MHz, D_2O)** δ 3.37 (dd, *J* = 12.7, 3.8 Hz, 1H), 3.31–3.22 (m, 1H), 3.13 (dd, *J* = 12.7, 9.1 Hz, 1H), 3.10–3.03 (m, 1H), 2.67–2.57 (m, 1H), 2.07–2.00 (m, 1H), 1.95–1.87 (m, 1H), 1.81–1.68 (m, 2H). **^{13}C NMR (126 MHz, D_2O)** δ 180.1, 45.8, 44.0, 40.9, 25.7, 21.0. **HRMS m/z (ESI):** calculated for $C_6H_{10}N_1O_2$ $[M-H]^-$: 128.0717, found 128.0718. $[\alpha]_D^{25}$ = +5.0 (*c* = 0.67, H_2O). [Lit.[11] $[\alpha]_D^{25}$ = +3.6 (*c* = 5, H_2O)]

(*S*)-1-Benzoylpiperidine-3-carboxylic acid (6)

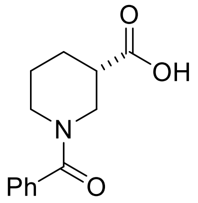

6 (20.5 mg, 88%) was obtained through the general procedure for derivatization in 0.1 mmol scale. **^{1}H NMR (501 MHz, Methanol-*d*$_4$)** δ 7.49–7.37 (m, 5H), 4.53 and 4.17 (br s, 1H), 3.76 and 3.57 (br s, 1H), 3.42–3.09 (m, 2H), 2.58 and 2.51 (s, 1H), 2.11 (br s, 1H), 1.89–1.42 (m, 3H). **^{13}C NMR (126 MHz, Methanol-*d*$_4$)** δ 176.6, 176.2, 172.8, 137.1, 131.0, 129.7, 127.8, 50.7, 45.2, 43.6, 42.7, 42.3, 28.3, 25.9, 24.9. **HRMS m/z (ESI):** calculated for $C_{13}H_{14}N_1O_3$ $[M-H]^-$: 232.0979, found 232.0981.

The enantiomeric excess was determined by chiral HPLC analysis on OJ-3 column. Conditions: heptane/isopropanol/trifluoroacetic acid = 90:10:0.1, flow rate = 1.0 mL/min, uv-vis detection at λ = 220 nm, t_{R1} = 20.9 min (minor), t_{R2} = 26.3 min (major).

(*S*)-*N*-((2-oxotetrahydro-2H-pyran-3-yl)methyl)benzamide (7)

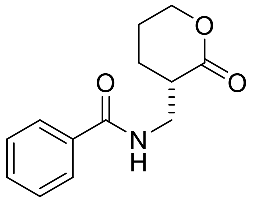

Following the general procedure for enantioselective aminomethylation, the reaction (0.1 mmol) was performed at −60 °C for 3 d. Then benzoyl chloride (0.12 mmol, 17 mg, 1.2 equiv.), K_2CO_3 (0.22 mmol, 25 mg, 2.2 equiv.), DCM (0.2 mL) and H_2O (0.2 mL) were added into the reaction mixture. The mixture was stirred vigorously for 4 h before the aqueous phase was separated and extracted with DCM (3x3 mL). The organic phase was combined and the solvent was removed under reduced pressure. The crude product was purified by column chromatography on silica to obtaine **7** (17.9 mg, 77%, 98.5:1.5 e.r.) as a white solid. **^{1}H NMR (501 MHz, $CDCl_3$)** δ 7.79–7.70 (m, 2H), 7.50–7.43 (m, 1H), 7.43–7.36 (m, 2H), 7.18 (br s, 1H), 4.29 (t, *J* = 5.5 Hz,

2H), 3.88 (ddd, J = 13.7, 7.6, 3.9 Hz, 1H), 3.45 (ddd, J = 13.4, 8.1, 4.9 Hz, 1H), 2.77 (dtd, J = 11.9, 8.0, 3.9 Hz, 1H), 2.22–2.12 (m, 1H), 1.96–1.87 (m, 2H), 1.61 (ddt, J = 13.6, 11.9, 7.5 Hz, 1H). **^{13}C NMR (126 MHz, $CDCl_3$)** δ 175.1, 167.5, 134.2, 131.6, 128.6, 127.0, 68.2, 40.2, 40.2, 22.3, 21.7. **HRMS m/z (EI):** calculated for $C_{13}H_{15}N_1O_3$ $[M]^+$: 233.1046, found 233.1046.

The enantiomeric excess was determined by chiral HPLC analysis on IA column. Conditions: heptane/isopropanol/trifluoroacetic acid = 90:10:0.1, flow rate = 1.0 mL/min, uv-vis detection at λ = 220 nm, t_{R1} = 16.8 min (major), t_{R2} = 22.2 min (minor).

Optional in situ derivatization

(*S*)-2-benzyl-3-((tert-butoxycarbonyl)amino)propanoic acid (8)

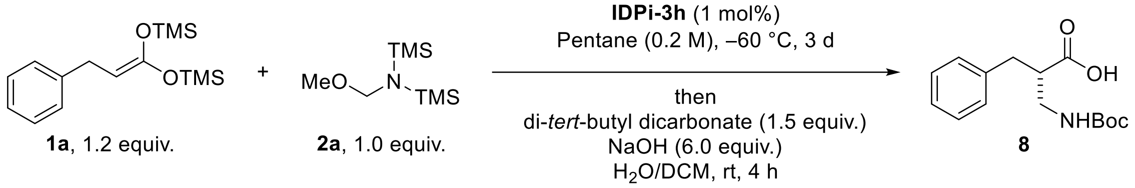

Following the general procedure, the reaction (0.1 mmol) was performed at –60 °C for 3 d. Then di-*tert*-butyl dicarbonate (0.15 mmol, 32.7 mg, 1.5 equiv.), NaOH (0.6 mmol, 24 mg, 6.0 equiv.), DCM (0.5 mL) and H_2O (0.5 mL) were added into the reaction mixture. The mixture was stirred vigorously for 4 h and then 1 M HCl was added until the aqueous solution was acidic (pH<3). The aqueous phase was separated and extracted with DCM (3x3 mL). The organic phase was combined and the solvent was removed under reduced pressure. The crude product was purified by column chromatography on silica by eluting with 33:66:1 EtOAc/hexanes/AcOH to give the titled product (22.8 mg, 82%, 96:4 e.r.) as a white solid.[12] **^{1}H NMR (501 MHz, $CDCl_3$)** δ 7.32–7.26 (m, 2H), 7.24–7.17 (m, 3H), 6.51 and 4.94 (2s, 1H), 3.45–2.61 (m, 5H), 1.43 and 1.38 (2s, 9H). **^{13}C NMR (126 MHz, $CDCl_3$)** δ 179.0, 158.0, 156.0, 138.2, 129.0, 128.7, 126.8, 79.9, 47.7, 47.3, 42.3, 41.4, 35.8, 28.5, 28.4. **HRMS m/z (ESI):** calculated for $C_{15}H_{20}N_1O_4$ $[M-H]^-$: 278.1398, found 278.1399.

The enantiomeric excess was determined by chiral HPLC analysis on AS-3 column. Conditions: heptane/isopropanol/trifluoroacetic acid = 90:10:0.1, flow rate = 1.0 mL/min, uv-vis detection at λ = 220 nm, t_{R1} = 4.1 min (major), t_{R2} = 5.6 min (minor).

(*S*)-3-((((9*H*-fluoren-9-yl)methoxy)carbonyl)amino)-2-benzylpropanoic acid (9)

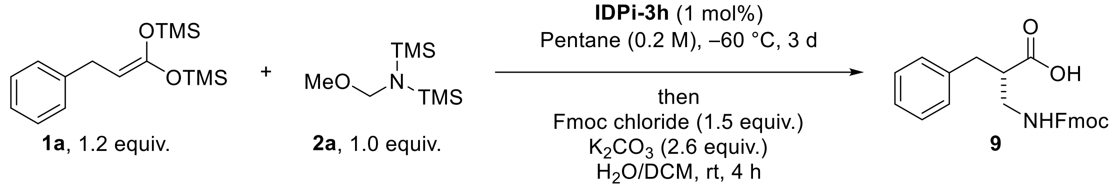

Following the general procedure, the reaction (0.1 mmol) was performed at –60 °C for 3 d. Then Fmoc chloride (0.15 mmol, 38.8 mg, 1.5 equiv.), K_2CO_3 (0.26 mmol, 36 mg, 2.6 equiv.), DCM (0.5 mL) and H_2O (0.5 mL) were added into the reaction mixture. The mixture was stirred vigorously for 4 h [*The reaction mixture contained a lot of insoluble compound*] and then 1 M HCl was added until the aqueous solution was acidic (pH<3). The aqueous phase was separated and extracted with DCM (3x3 mL). The organic phase (contained the insoluble compound) was combined and the solvent was removed under reduced pressure. The crude product was purified by column chromatography on silica by eluting with 33:66:1 EtOAc/hexanes/AcOH and 50:50:1 EtOAc/hexanes/AcOH to give the titled product (34.0 mg, 85%, 95.5:4.5 e.r.) as a white solid. **^{1}H NMR (501 MHz, DMSO-*d*$_6$)** δ 12.25 (br s, 1H), 7.88 (d, *J* = 7.4 Hz, 2H), 7.73–7.60 (m, 2H), 7.50 (t, *J* = 5.9 Hz, 1H), 7.41 (t, *J* = 7.4 Hz, 2H), 7.32 (td, *J* = 7.4, 1.2 Hz, 2H), 7.27 (t, *J* = 7.5 Hz, 2H), 7.22–7.10 (m, 3H), 4.33–4.26 (m, 2H), 4.21 (t, *J* = 7.0 Hz, 1H), 3.29–3.18 (m, 1H), 3.17–3.08 (m, 1H), 2.82–2.66 (m, 3H). **^{13}C NMR (126 MHz, DMSO-*d*$_6$)** δ 174.6, 156.1, 143.9, 143.8, 140.7, 139.1, 128.7, 128.2, 127.6, 127.0, 126.1, 125.2, 120.1, 65.4, 47.1, 46.7, 42.0, 35.1. **HRMS m/z (ESI):** calculated for $C_{25}H_{23}N_1O_4Na_1$ $[M+Na]^+$: 424.1519, found 424.1513.

The enantiomeric excess was determined by chiral HPLC analysis on AS-3 column. Conditions: heptane/isopropanol/trifluoroacetic acid = 90:10:0.1, flow rate = 1.0 mL/min, uv-vis detection at λ = 220 nm, t_{R1} = 11.8 min (major), t_{R2} = 15.2 min (minor).

6. Synthesis and Characterization of Catalysts

The chiral imidodiphosphorimidate acids (IDPis) **3a–d**[1] were synthesized according to literature procedures.

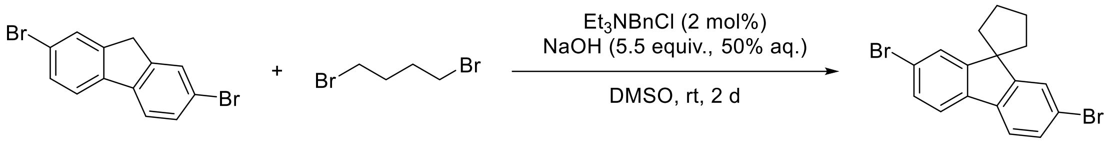

2',7'-dibromospiro[cyclopentane-1,9'-fluorene]: Following a reported procedure,[1] NaOH (50% aq., 8.8 mL, 110 mmol, 5.5 equiv.) was added dropwise to the mixture of 2,7-dibromo-9*H*-fluorene (6.48 g, 20.0 mmol, 1.0 equiv.) and benzyl-triethylammonium chloride (91.1 g, 0.4 mmol, 2 mol%) in DMSO (20 mL) under argon at rt. After the subsequent dropwise addition of 1,4-dibromobutane (2.4 mL, 20.0 mmol, 1.0 equiv.), the reaction was stirred for 2 d at rt. Then, water (100 mL) and toluene (100 mL) were added and the organic layer was separated, dried over $MgSO_4$ and concentrated under reduced pressure. The crude product was purified by column chromatography (hexanes) to yield 2',7'-dibromospiro[cyclopentane-1,9'-fluorene] as a white solid (6.80 g, 18.0 mmol, 90%). **^{1}H NMR (501 MHz, $CDCl_3$)** δ 7.55–7.49 (m, 4H), 7.44 (dd, *J* = 8.1, 1.8 Hz, 2H), 2.15–2.04 (m, 8H). **^{13}C NMR (126 MHz, $CDCl_3$)** δ 156.2, 137.6, 130.2, 126.5, 121.7, 121.1, 58.0, 39.8, 27.1. **HRMS m/z (APPI):** calculated for $C_{17}H_{14}Br_2$ $[M]^+$: 375.9457, found 375.9458.

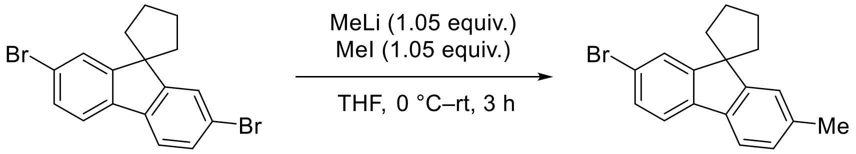

2'-bromo-7'-methylspiro[cyclopentane-1,9'-fluorene]: To a solution of 2',7'-dibromospiro[cyclopentane-1,9'-fluorene] (756 mg, 2.0 mmol, 1.0 equiv.) in 6 mL THF was added MeLi (1.25 mL, 1.6 M in Et_2O, 2.0 mmol, 1.0 equiv.) and MeI (142 mg, 1.0 mmol, 0.5 equiv.) at 0 ˚C under argon atmosphere. The mixture was stirred at 0 ˚C for 30 min and at rt for 3 h, then water (10 mL) and DCM (10 mL) was added. The organic layer was separated and dried over $MgSO_4$. The solvent was removed under reduced pressure and the product was purified by column chromatography by eluting with hexanes (363 mg, 58%). **^{1}H NMR (501 MHz, $CDCl_3$)** δ 7.56–7.48 (m, 3H), 7.42 (dd, *J* = 8.0, 1.8 Hz, 1H), 7.22 (s, 1H), 7.16–7.10 (m, 1H), 2.42 (s, 3H), 2.15–2.04 (m, 8H). **^{13}C NMR (126 MHz, $CDCl_3$)** δ 156.5, 154.3, 138.7, 138.0, 136.0, 129.8,

127.9, 126.3, 123.7, 120.7, 120.7, 119.5, 57.8, 39.9, 27.1, 22.0. **HRMS m/z (APPI):** calculated for $C_{18}H_{17}Br_1$ $[M]^+$: 312.0508, found 312.0504.

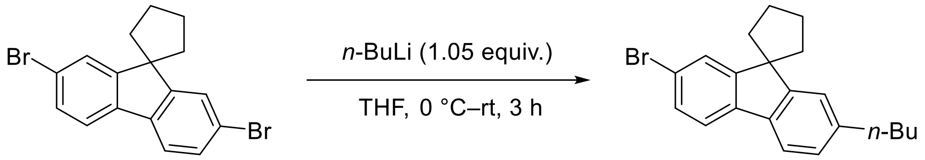

2'-bromo-7'-butylspiro[cyclopentane-1,9'-fluorene]: To a solution of 2',7'-dibromospiro[cyclopentane-1,9'-fluorene] (756 mg, 2.0 mmol, 1.0 equiv.) in 6 mL THF was added *n*-BuLi (0.8 mL, 2.5 M in hexane, 2.0 mmol, 1.0 equiv.) at 0 ˚C under argon atmosphere. The mixture was stirred at 0 ˚C for 30 min and at rt for 3 h, then water (10 mL) and DCM (10 mL) was added. The organic layer was separated and dried over $MgSO_4$. The solvent was removed under reduced pressure and the product was purified by column chromatography by eluting with hexanes (455 mg, 64%). **^{1}H NMR (501 MHz, $CDCl_3$)** δ 7.56 (d, *J* = 7.7 Hz, 1H), 7.52–7.49 (m, 2H), 7.41 (dd, *J* = 8.0, 1.8 Hz, 1H), 7.21 (s, 1H), 7.14 (dd, *J* = 7.7, 1.5 Hz, 1H), 2.67 (t, *J* = 7.9 Hz, 2H), 2.15–2.10 (m, 4H), 2.09–2.03 (m, 4H), 1.67–1.59 (m, 2H), 1.44–1.35 (m, 2H), 0.95 (t, *J* = 7.4 Hz, 3H). **^{13}C NMR (126 MHz, $CDCl_3$)** δ 156.6, 154.3, 143.2, 138.8, 136.2, 129.8, 127.3, 126.3, 123.0, 120.7, 120.7, 119.5, 57.8, 39.9, 36.2, 34.1, 27.1, 22.6, 14.1. **HRMS m/z (APPI):** calculated for $C_{21}H_{23}Br_1$ $[M]^+$: 354.0978, found 354.0980.

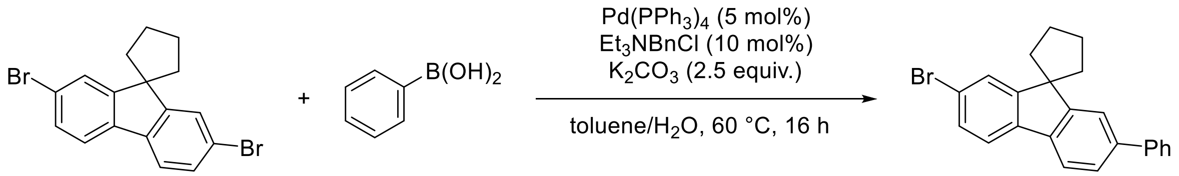

2'-bromo-7'-phenylspiro[cyclopentane-1,9'-fluorene]: To a schlenk tube was added 2',7'-dibromospiro[cyclopentane-1,9'-fluorene] (756 mg, 2.0 mmol, 1.0 equiv.), phenylboronic acid (244 mg, 2.0 mmol, 1.0 equiv.), tetrakis(triphenylphosphine)palladium (115.5 mg, 0.1 mmol, 0.05 equiv), Benzyltriethylammonium chloride (45.6 mg, 0.2 mmol, 0.1 equiv) and K_2CO_3 (690 mg, 4.0 mmol, 2.5 equiv) under argon atmosphere. Degassed toluene (10 mL) and water (10 mL) were sequentially added. The mixture was then heated to 60 °C and stirred for 16 h. After cooling the reaction to rt, the organic layer was separated and the aqueous phase was extracted with EtOAc (3x5 mL). The solvent was removed under reduced pressure and the product was purified

by column chromatography by eluting with hexanes (368 mg, 49%). **^{1}H NMR (501 MHz, $CDCl_3$)** δ 7.72 (d, *J* = 7.9 Hz, 1H), 7.66–7.61 (m, 3H), 7.60–7.53 (m, 3H), 7.50–7.43 (m, 3H), 7.37 (td, *J* = 7.3, 1.3 Hz, 1H), 2.21–2.07 (m, 8H). **^{13}C NMR (126 MHz, $CDCl_3$)** δ 156.8, 154.8, 141.7, 141.3, 138.3, 137.8, 130.0, 129.0, 127.4, 127.4, 126.5, 126.3, 121.9, 121.3, 121.1, 120.1, 58.0, 40.0, 27.2. **HRMS m/z (APPI):** calculated for $C_{23}H_{19}Br_1$ $[M]^+$: 374.0665, found 374.0668.

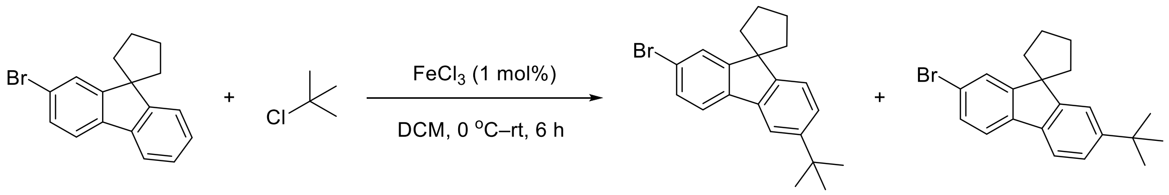

2'-Bromo-7'-(*tert*-butyl)spiro[cyclopentane-1,9'-fluorene]

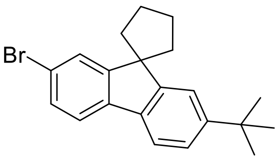

To a solution of 2'-bromospiro[cyclopentane-1,9'-fluorene] (598 mg, 2.0 mmol, 1.0 equiv.) in 5 mL of dichloromethane was added anhydrous $FeCl_3$ (1.7 mg, 0.02 mmol, 0.01 equiv.) at 0 ˚C under argon atmosphere. *Tert*-butyl chloride (222 mg, 2.4 mmol, 1.2 equiv.) was added over a period of 10 min at 0 ˚C. The mixture was stirred at 0 °C for 3 h before it was warmed up slowly to rt and stirred for another 3 h. Then water (10 mL) was added into the reaction mixture. The organic layer was separated and washed with 10% of HCl solution and then dried over $MgSO_4$. The solvent was removed under reduced pressure. The product 2'-Bromo-7'-(*tert*-butyl)spiro[cyclopentane-1,9'-fluorene] was purified by column chromatography on Al_2O_3 by eluting with hexanes (328 mg, 46%). The configuration of the product was confirmed by HMBC and X-ray crystallographic analysis. **^{1}H NMR (501 MHz, $CDCl_3$)** δ 7.59 (d, *J* = 8.0 Hz, 1H), 7.54–7.49 (m, 2H), 7.45–7.40 (m, 2H), 7.40–7.35 (m, 1H), 2.19–2.02 (m, 8H), 1.38 (s, 9H). **^{13}C NMR (126 MHz, $CDCl_3$)** δ 156.8, 153.8, 151.5, 138.6, 136.0, 129.8, 126.3, 124.3, 120.8, 120.7, 119.7, 119.2, 58.0, 39.9, 35.2, 31.8, 27.1. **HRMS m/z (APPI):** calculated for $C_{21}H_{23}Br_1$ $[M]^+$: 354.0978, found 354.0982.

General procedure for cross-coupling reactions and subsequent MOM-deprotections and IDPi synthesis:

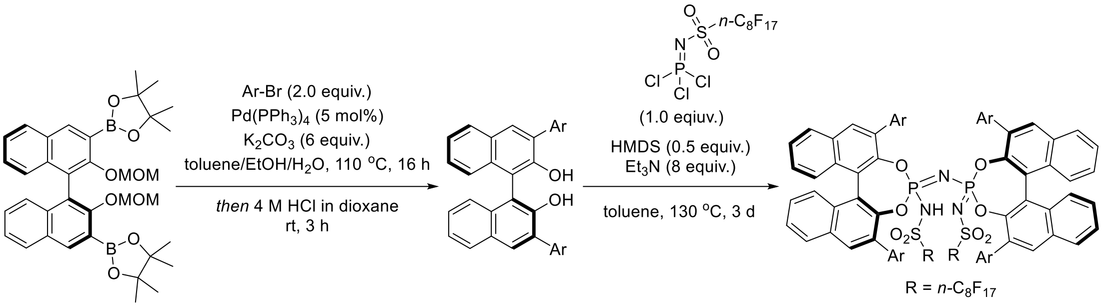

To a two-neck round-bottom flask with a condenser was added (*S*)-2,2'-(2,2'-bis(methoxymethoxy)-1,1'binaphthyl-3,3'-diyl)bis(4,4,-5,5-tetramethyl-1,3,2-dioxaborolane) (250 mg, 0.4 mmol, 1.0 equiv), 2'-Bromo-7'-substituted-spiro[cyclopentane-1,9'-fluorene] (0.8 mmol, 2.0 equiv), tetrakis(triphenylphosphine)palladium (23.1 mg, 0.02 mmol, 0.5 equiv) and K_2CO_3 (332 mg, 2.4 mmol, 6 equiv) under argon atmosphere. Degassed toluene (3 mL), ethanol (2 mL) and water (1 mL) were sequentially added. The mixture was then heated to 108 °C and stirred at that temperature overnight. After cooling the reaction to rt, the organic layer was separated and the aqueous phase was extracted with EtOAc (3x10 mL). The organic phase was combined and filter through a thin layer of silica gel using a Büchner funnel, and the silica gel layer washed with some extra EtOAc. The solvent was removed under reduced pressure and the crude MOM-protected diol was obtained. Subsequently, the crude product was dissolved in a small amount of DCM (1 mL). A solution of HCl (4 M in 1,4-dioxane, 3 mL) was added at rt and the mixture was stirred at rt for 3 h. The solvent was removed under reduced pressure and the crude was purified by column chromatography to afford the corresponding diol.

In a Schlenk tube under argon, a suspension of 3,3’-substituted (*S*,*S*)-BINOL (0.21 mmol, 2.1 equiv.) in toluene (0.8 mL) was treated with ((perfluorooctyl)sulfonyl)phosphorimidoyl trichloride[1] (0.21 mmol, 2.1 equiv.) and Et_3N (162 mg, 1.6 mmol, 16 equiv.). The reaction mixture was stirred for 45 min at rt, then neat hexamethyldisilazane (HMDS, 21 μL, 0.1 mmol, 1 equiv.) was added dropwise. The reaction mixture was stirred for additional 15 min at rt, then the Schlenk tube was subsequently sealed and heated to 130 °C for 3 d. After cooling to rt, aq. HCl (10%) was added and the mixture was extracted with DCM. The combined organic layers were washed with brine, dried over $MgSO_4$ and concentrated under reduced pressure. The crude material was purified by column chromatography on silica gel (hexanes:DCM = 4:1→1:1) to afford the desired product as a salt. The corresponding IDPi Brønsted acids were obtained after

acidification in DCM with aq. HCl (6 M) and evaporation of the solvent followed by drying under high vacuum as typically off-white solids. [Acidification procedure: The product as a salt was dissolved in 5 mL DCM, then aq. HCl (6 M, 8 mL) was added, and the mixture was stirred vigorously for 10 minutes. The organic layer was separated and the aqueous phase was extracted with DCM for several times until there was no product could be detected by TLC in the last DCM extract. The organic layers were combined and the solvent was removed under reduced pressure. The obtained IDPi solid was dried under high vacuum for 16 h at rt.]

(*S*)-3,3'-bis(2'-methylspiro[cyclopentane-1,9'-fluoren]-7'-yl)-[1,1'-binaphthalene]-2,2'-diol

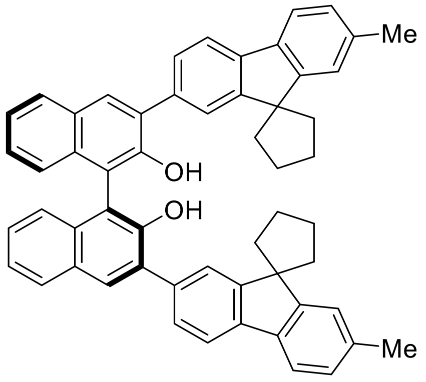

Prepared according to the general procedure with 2'-bromo-7'-methylspiro[cyclopentane-1,9'-fluorene] (251 mg, 0.8 mmol) to give the title compound as an off-white solid (243.4 mg, 81%). **^{1}H NMR (501 MHz, $CDCl_3$)** δ 8.08 (s, 2H), 7.95 (d, *J* = 8.0 Hz, 2H), 7.81–7.74 (m, 4H), 7.69 (dd, *J* = 7.8, 1.6 Hz, 2H), 7.63 (d, *J* = 7.6 Hz, 2H), 7.44–7.38 (m, 2H), 7.36–7.31 (m, 2H), 7.29–7.26 (m, 4H), 7.16 (d, *J* = 7.2 Hz, 2H), 5.48 (s, 2H), 2.45 (s, 6H), 2.20–2.09 (m, 16H). **^{13}C NMR (126 MHz, $CDCl_3$)** δ 155.1, 154.7, 150.3, 139.5, 137.7, 136.6, 136.1, 133.1, 131.3, 131.3, 129.7, 128.5, 128.4, 127.8, 127.3, 124.6, 124.4, 124.3, 123.8, 119.7, 119.4, 112.8, 57.8, 40.1, 40.0, 27.2, 22.0. **HRMS m/z (ESI):** calculated for $C_{56}H_{45}O_2$ [M-H]$^-$: 749.3425, found 749.3433.

(*S*)-3,3'-bis(2'-butylspiro[cyclopentane-1,9'-fluoren]-7'-yl)-[1,1'-binaphthalene]-2,2'-diol

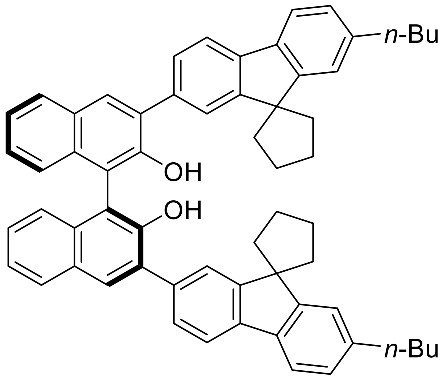

Prepared according to the general procedure with 2'-bromo-7'-butylspiro[cyclopentane-1,9'-fluorene] (284 mg, 0.8 mmol) to give the title compound as an off-white solid (298 mg, 89%). **^{1}H NMR (501 MHz, $CDCl_3$)** δ 8.09 (s, 2H), 7.95 (d, *J* = 8.1 Hz, 2H), 7.84–7.73 (m, 4H), 7.72–7.62 (m, 4H), 7.41 (t, *J* = 6.9 Hz, 2H), 7.34 (t, *J* = 7.0 Hz, 2H), 7.27 (d, *J* = 12.3 Hz, 4H), 7.17 (dd, *J* = 7.7, 1.4 Hz, 2H), 5.48 (s, 2H), 2.70 (t, *J* = 7.8 Hz, 4H), 2.20–2.11 (m, 16H), 1.71–1.63 (m, 4H), 1.45–1.38 (m, 4H), 0.97 (t,

J = 7.4 Hz, 6H). **^{13}C NMR (126 MHz, CDCl$_3$)** δ 155.0, 154.8, 150.3, 142.9, 139.5, 136.8, 136.1, 133.1, 131.3, 131.3, 129.7, 128.5, 128.4, 127.3, 127.1, 124.6, 124.4, 124.3, 123.1, 119.6, 119.4, 112.9, 57.8, 40.1, 40.0, 36.3, 34.2, 27.2, 22.7, 14.2. **HRMS m/z (ESI):** calculated for $C_{62}H_{57}O_2$ $[M-H]^-$: 833.4364, found 833.4374.

(*S*)-3,3'-bis(2'-phenylspiro[cyclopentane-1,9'-fluoren]-7'-yl)-[1,1'-binaphthalene]-2,2'-diol

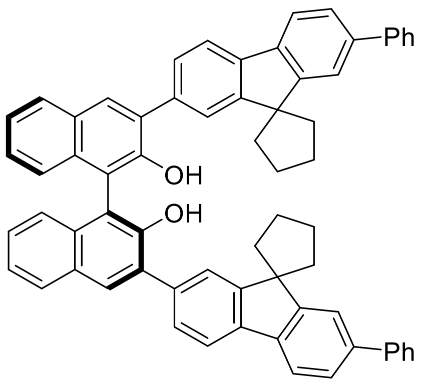

Prepared according to the general procedure with 2'-bromo-7'-phenylspiro[cyclopentane-1,9'-fluorene] (300 mg, 0.8 mmol) to give the title compound as an off-white solid (306 mg, 87%). **^{1}H NMR (501 MHz, CDCl$_3$)** δ 8.12 (s, 2H), 8.01–7.95 (m, 2H), 7.88–7.78 (m, 6H), 7.76–7.73 (m, 2H), 7.70–7.65 (m, 6H), 7.60 (dd, *J* = 7.8, 1.6 Hz, 2H), 7.48 (t, *J* = 7.7 Hz, 4H), 7.45–7.41 (m, 2H), 7.40–7.33 (m, 4H), 7.30 (dd, *J* = 8.3, 1.2 Hz, 2H), 5.50 (s, 2H), 2.28–2.15 (m, 16H). **^{13}C NMR (126 MHz, CDCl$_3$)** δ 155.5, 155.0, 150.3, 141.8, 140.9, 139.0, 138.5, 136.7, 133.1, 131.4, 131.2, 129.7, 128.9, 128.6, 128.6, 127.4, 127.4, 127.3, 126.2, 124.5, 124.5, 124.4, 121.9, 120.2, 119.8, 112.8, 58.0, 40.1, 40.1, 27.2. **HRMS m/z (ESI):** calculated for $C_{66}H_{49}O_2$ $[M-H]^-$: 873.3738, found 873.3745.

(*S*)-3,3'-Bis(2'-(tert-butyl)spiro[cyclopentane-1,9'-fluoren]-7'-yl)-[1,1'-binaphthalene]-2,2'-diol

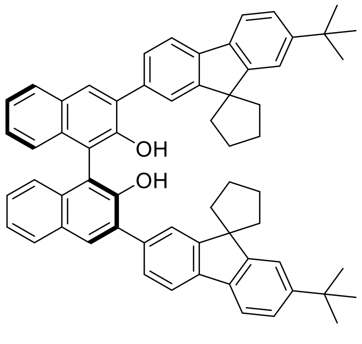

Prepared according to the general procedure with 2'-Bromo-7'-(*tert*-butyl)spiro[cyclopentane-1,9'-fluorene] (284 mg, 0.8 mmol) to give the title compound as an off-white solid (313 mg, 93%). **^{1}H NMR (501 MHz, CDCl$_3$)** δ 8.11 (s, 2H), 7.97 (d, *J* = 8.1 Hz, 2H), 7.83–7.77 (m, 4H), 7.73–7.65 (m, 4H), 7.49 (d, *J* = 1.7 Hz, 2H), 7.45–7.39 (m, 4H), 7.38–7.33 (m, 2H), 7.29 (dd, *J* = 8.3, 1.3 Hz, 2H), 5.50 (s, 2H), 2.24–2.12 (m, 16H), 1.41 (s, 18H). **^{13}C NMR (126 MHz, CDCl$_3$)** δ 154.8, 154.5, 151.0, 150.2, 139.3, 136.6, 136.0, 133.0, 131.2, 131.2, 129.6, 128.4, 128.2, 127.2, 124.5, 124.3, 124.2, 124.0, 119.7, 119.4, 119.2, 112.8, 57.9, 40.0, 39.9, 35.1, 31.7, 27.0. **HRMS m/z (ESI):** calculated for $C_{62}H_{57}O_2$ $[M-H]^-$: 833.4364, found 833.4373.

(*S*)-Imidodiphosphorimidate (3e)

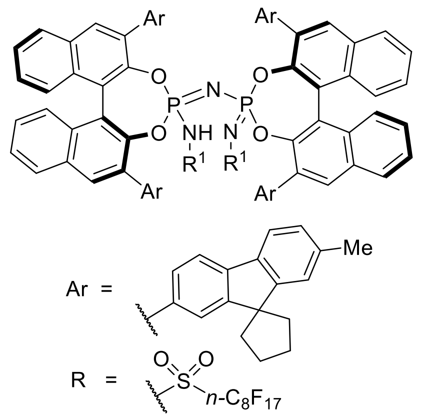

Prepared according to the general procedure with diol (100 mg, 0.133 mmol) to give the title compound as an off-white solid (106 mg, 62%). **^{1}H NMR (501 MHz, $CDCl_3$)** δ 8.05–7.99 (m, 4H), 7.96 (d, *J* = 8.4 Hz, 2H), 7.83 (t, *J* = 7.6 Hz, 2H), 7.77 (d, *J* = 8.5 Hz, 2H), 7.66 (dd, *J* = 8.4, 6.8 Hz, 2H), 7.58–7.54 (m, 2H), 7.49 (d, *J* = 1.6 Hz, 2H), 7.42 (d, *J* = 7.8 Hz, 2H), 7.38 (d, *J* = 7.9 Hz, 2H), 7.36–7.30 (m, 4H), 7.24–7.16 (m, 6H), 7.12 (d, *J* = 1.5 Hz, 2H), 7.03–6.92 (m, 6H), 6.64–6.50 (m, 4H), 6.43 (dd, *J* = 8.0, 1.6 Hz, 2H), 2.35 (s, 6H), 2.34 (s, 6H), 2.22–2.16 (m, 2H), 2.14–1.90 (m, 22H), 1.83–1.61 (m, 8H). **^{13}C NMR (126 MHz, $CDCl_3$)** δ 155.3, 155.1, 154.3, 153.9, 144.3, 143.1, 140.1, 139.2, 137.7, 136.9, 136.8, 136.2, 135.1, 134.5, 134.0, 133.8, 132.3, 132.1, 131.8, 131.8, 131.6, 131.2, 129.4, 128.8, 128.8, 128.6, 127.8, 127.2, 127.1, 127.0, 126.7, 126.5, 126.4, 123.8, 123.7, 123.5, 123.4, 121.8, 119.7, 119.0, 118.3, 118.2, 57.9, 57.7, 39.8, 39.7, 39.4, 39.1, 27.2, 26.8, 26.6, 26.5, 21.9, 21.7 (other signals not detected or observed). **^{19}F NMR (471 MHz, $CDCl_3$)** δ –80.78 (t, *J* = 10.0 Hz, 6F), –110.54 to –112.65 (m, 4F), –119.97 (dt, *J* = 58.4, 15.6 Hz, 4F), –121.33 to –121.67 (m, 4F), –121.81 to –122.19 (m, 4F), –122.66 to –122.92 (m, 4F), –126.02 to –126.33 (m, 4F). **^{31}P NMR (203 MHz, $CDCl_3$)** δ –16.74. **HRMS m/z (ESI):** calculated for $C_{128}H_{88}F_{34}N_3O_8P_2S_2$ $[M-H]^-$: 2566.4951, found 2566.4940.

(*S*)-Imidodiphosphorimidate (3f)

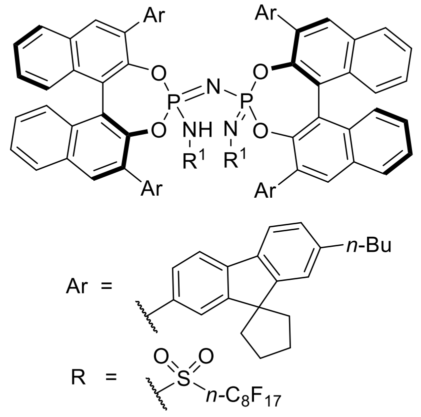

Prepared according to the general procedure with diol (100 mg, 0.120 mmol) to give the title compound as an off-white solid (110 mg, 67%). **^{1}H NMR (501 MHz, CD_2Cl_2)** δ 8.17–8.03 (m, 4H), 7.98 (d, *J* = 8.2 Hz, 2H), 7.87 (t, *J* = 7.5 Hz, 2H), 7.76 (d, *J* = 8.4 Hz, 2H), 7.69 (t, *J* = 7.7 Hz, 2H), 7.64–7.57 (m, 2H), 7.54 (s, 2H), 7.46 (dd, *J* = 8.0, 5.3 Hz, 4H), 7.42–7.31 (m, 4H), 7.30–7.17 (m, 6H), 7.14 (s, 2H), 7.06–6.92 (m, 6H), 6.86–6.50

(m, 4H), 6.47–6.37 (m, 2H), 2.59 (t, *J* = 7.8 Hz, 8H), 2.21–1.96 (m, 22H), 1.96–1.88 (m, 2H), 1.86–1.78 (m, 2H), 1.68–1.61 (m, 4H), 1.60–1.52 (m, 8H), 1.39–1.26 (m, 10H), 0.91 (t, *J* = 7.3 Hz, 6H), 0.85 (t, *J* = 7.3 Hz, 6H). **^{13}C NMR (126 MHz, CD_2Cl_2)** δ 155.7, 155.4, 155.1, 154.5, 144.6, 143.7, 143.0, 140.5, 139.8, 137.1, 136.8, 135.2, 135.1, 134.2, 134.1, 132.8, 132.5, 132.3, 132.2, 132.0, 131.8, 130.1, 129.2, 129.0, 127.9, 127.6, 127.4, 127.2, 127.1, 127.0, 124.4, 123.9, 123.7, 123.5, 123.1, 122.4, 119.9, 119.5, 118.9, 118.5, 58.4, 58.2, 40.4, 40.2, 39.9, 39.5, 36.6, 36.5, 34.6, 34.5, 27.6, 27.3, 27.0, 26.9, 23.1, 23.0, 14.3, 14.2 (other signals not detected or observed). **^{19}F NMR (471 MHz, CD_2Cl_2)** δ –81.14 (t, *J* = 10.1 Hz, 6F), –110.87 to –112.43 (m, 4F), –120.20 (s, 4F), –121.52 (s, 4F), –122.07 (d, *J* = 49.8 Hz, 4F), –122.89 (s, 4F), –126.34 (s, 4F). **^{31}P NMR (203 MHz, CD_2Cl_2)** δ –17.17. **HRMS m/z (ESI):** calculated for $C_{140}H_{112}F_{34}N_3O_8P_2S_2$ $[M-H]^-$: 2734.6829, found 2734.6808.

(*S*)-Imidodiphosphorimidate (3g)

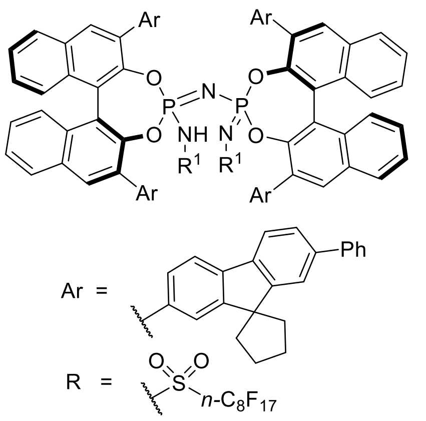

Prepared according to the general procedure with diol (100 mg, 0.114 mmol) to give the title compound as an off-white solid (92 mg, 57%). **^{1}H NMR (501 MHz, $CDCl_3$)** δ 8.09–8.03 (m, 4H), 7.99 (d, *J* = 8.3 Hz, 2H), 7.86 (t, *J* = 7.5 Hz, 2H), 7.81 (d, *J* = 8.5 Hz, 2H), 7.69 (t, *J* = 7.7 Hz, 2H), 7.64–7.61 (m, 4H), 7.58 (d, *J* = 7.4 Hz, 6H), 7.56–7.53 (m, 6H), 7.51–7.46 (m, 4H), 7.45–7.34 (m, 16H), 7.33–7.30 (m, 6H), 7.29–7.27 (m, 2H), 7.01 (s, 2H), 6.73 (d, *J* = 8.0 Hz, 2H), 6.64 (d, *J* = 8.0 Hz, 2H), 6.53 (d, *J* = 7.9 Hz, 2H), 2.30–2.22 (m, 2H), 2.18–2.07 (m, 16H), 2.05–1.97 (m, 6H), 1.89–1.67 (m, 8H). **^{13}C NMR (126 MHz, $CDCl_3$)** δ 155.8, 155.5, 154.8, 154.3, 142.1, 141.8, 141.1, 140.4, 139.6, 138.9, 138.5, 138.0, 135.1, 134.9, 134.4, 133.9, 132.3, 132.2, 131.9, 131.8, 131.7, 131.3, 129.5, 128.8, 128.7, 127.4, 127.2, 127.1, 127.0, 126.9, 126.8, 126.6, 126.5, 126.3, 125.6, 124.0, 123.6, 123.5, 121.9, 121.8, 121.5, 120.2, 119.6, 118.8, 118.7, 58.2, 58.0, 39.8, 39.5, 39.1, 27.2, 26.8, 26.7, 26.7 (other signals not detected or observed). **^{19}F NMR (471 MHz, $CDCl_3$)** δ –80.80 (t, *J* = 10.0 Hz, 6F), –110.40 to –112.73 (m, 4F), –119.92 (d, *J* = 62.7 Hz, 4F), –121.40, –121.77 to –122.15 (m, 4F), –122.56 to –122.86 (m, 4F), –126.15 (t, *J* = 12.7 Hz, 4F). **^{31}P NMR (203**

MHz, $CDCl_3$) δ –16.55. **HRMS m/z (ESI):** calculated for $C_{148}H_{96}F_{34}N_3O_8P_2S_2$ $[M-H]^-$: 2814.5577, found 2814.5560.

(*S*)-Imidodiphosphorimidate (3h)

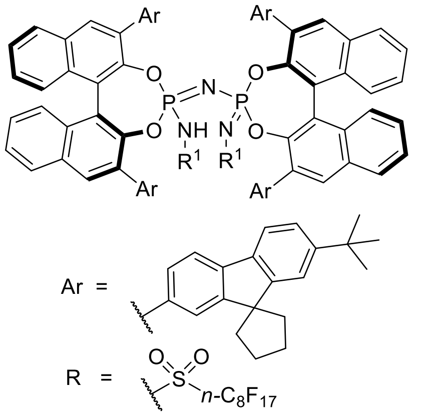

Prepared according to the general procedure with diol (100 mg, 0.120 mmol) to give the title compound as an off-white solid (166 mg, 71%). **^{1}H NMR (501 MHz, $CDCl_3$)** δ 8.07–8.01 (m, 4H), 7.97 (d, *J* = 8.3 Hz, 2H), 7.83 (t, *J* = 7.5 Hz, 2H), 7.78 (d, *J* = 8.5 Hz, 2H), 7.68–7.63 (m, 2H), 7.58–7.54 (m, 2H), 7.51 (d, *J* = 1.6 Hz, 2H), 7.44 (d, *J* = 8.0 Hz, 2H), 7.41 (d, *J* = 1.7 Hz, 2H), 7.38 (d, *J* = 7.9 Hz, 2H), 7.36–7.31 (m, 6H), 7.23–7.15 (m, 8H), 6.97 (s, 2H), 6.63 (d, *J* = 8.0 Hz, 2H), 6.57–6.39 (m, 4H), 2.22–2.16 (m, 2H), 2.12–1.90 (m, 22H), 1.83–1.63 (m, 8H), 1.30 (d, *J* = 1.9 Hz, 36H). **^{13}C NMR (126 MHz, $CDCl_3$)** δ 154.8, 154.6, 154.5, 154.1, 151.2, 150.4, 144.3, 143.1, 140.0, 139.2, 136.7, 136.1, 135.1, 134.5, 134.1, 133.8, 132.3, 132.1, 131.8, 131.7, 131.7, 131.3, 129.4, 128.8, 128.7, 127.2, 127.2, 127.0, 126.7, 126.5, 126.4, 124.1, 123.9, 123.5, 123.4, 123.3, 121.8, 119.6, 119.3, 119.3, 118.9, 118.4, 118.4, 58.1, 58.0, 39.9, 39.8, 39.3, 39.1, 35.1, 34.9, 31.7, 31.6, 27.1, 26.7, 26.6, 26.5 (other signals not detected or observed). **^{19}F NMR (471 MHz, $CDCl_3$)** δ –80.80 (t, *J* = 10.1 Hz, 6F), –110.50 to –112.58 (m, 4F), –119.67 to –120.33 (m, 4F), –121.11 to –121.58 (m, 4F), –121.83 to –122.33 (m, 4F), –122.48 to –122.91 (m, 4F), –126.13 (t, *J* = 14.4 Hz, 4F). **^{31}P NMR (203 MHz, $CDCl_3$)** δ –16.69. **HRMS m/z (ESI):** calculated for $C_{140}H_{112}F_{34}N_3O_8P_2S_2$ $[M-H]^-$: 2734.6829, found 2734.6811.

7. References

(1) Gatzenmeier, T.; Turberg, M.; Yepes, D.; Xie, Y. W.; Neese, F.; Bistoni, G.; List, B., Scalable and highly diastereo- and enantioselective catalytic Diels-Alder reaction of *α*,*β*-unsaturated methyl esters. *J. Am. Chem. Soc.* **2018**, *140*, 12671–12676.

(2) Kobayashi, T.; Tanaka, K.; Ishida, M.; Yamakita, N.; Abe, H.; Ito, H., Asymmetric total synthesis of pleurospiroketals A and B. *Chem. Commun.* **2018**, *54*, 10316–10319.

(3) Bruckner, S.; Weise, M.; Schobert, R., Synthesis of the entomopathogenic fungus metabolites Militarinone C and Fumosorinone A. *J. Org. Chem.* **2018**, *83*, 10805–10812.

(4) (a) Mandrelli, F.; Blond, A.; James, T.; Kim, H.; List, B., Deracemizing *α*-branched carboxylic acids by catalytic asymmetric protonation of bis-silyl ketene acetals with water or methanol. *Angew. Chem., Int. Ed.* **2019**, *58*, 11479–11482. (b) Badorrey, R.; Cativiela, C.; Díaz-de-Villegas, M. a. D.; Gálvez, J. A.; Gil, A., Efficient enantioconvergent synthesis of (*S*)-*α*-benzyl-*α*-methyl-*β*-alanine from (*R*)- and (*S*)-2-cyano-2-methyl-3-phenylpropanoic acid. *Tetrahedron: Asymmetry* **2003**, *14*, 2209–2214.

(5) Ege, M.; Wanner, K. T., Diastereoselective synthesis of *β*-amino acid derivatives from dihydropyridones. *Tetrahedron* **2008**, *64*, 7273–7282.

(6) Okano, K.; Morimoto, T.; Sekiya, M., Primary aminomethylation at the *α*-position of carboxylic-acids and esters. Trimethylsilyl triflate-catalyzed reaction of ketene silyl acetals with *N*,*N*-bis(trimethylsilyl)methoxymethylamine. *Chem. Pharm. Bull.* **1985**, *33*, 2228–2234.

(7) Ma, D.-Y.; Wang, D.-X.; Zheng, Q.-Y.; Wang, M.-X., Nitrile biotransformations for the practical synthesis of highly enantiopure azido carboxylic acids and amides, 'click' to functionalized chiral triazoles and chiral *β*-amino acids. *Tetrahedron: Asymmetry* **2006**, *17*, 2366–2376.

(8) Diaz-Sanchez, B. R.; Iglesias-Arteaga, M. A.; Melgar-Fernandez, R.; Juaristi, E., Synthesis of 2-substituted-5-halo-2,3-dihydro-4(*H*)-pyrimidin-4-ones and their derivatization utilizing the Sonogashira coupling reaction in the enantioselective synthesis of *α*-substituted *β*-amino acids. *J. Org. Chem.* **2007**, *72*, 4822–4825.

(9) Beddow, J. E.; Davies, S. G.; Ling, K. B.; Roberts, P. M.; Russell, A. J.; Smith, A. D.; Thomson, J. E., Asymmetric synthesis of β^2-amino acids: 2-substituted-3-aminopropanoic acids from *N*-acryloyl SuperQuat derivatives. *Org. Biomol. Chem.* **2007**, *5*, 2812–2825.

(10) Reyes-Rangel, G.; Jiménez-González, E.; Olivares-Romero, J. L.; Juaristi, E., Enantioselective synthesis of *β* -amino acids using hexahydrobenzoxazolidinones as chiral auxiliaries. *Tetrahedron: Asymmetry* **2008**, *19*, 2839–2849.

(11) Akkerman, A. M.; Dejongh, D. K.; Veldstra, H., Synthetic oxytocics .1. 3-(piperidyl-(*N*)-methyl-)indoles and related compounds. *Recl. Trav. Chim. Pay. B* **1951**, *70*, 899–916.

(12) Moumne, R.; Lavielle, S.; Karoyan, P., Efficient synthesis of β^2-amino acid by homologation of *α*-amino acids involving the Reformatsky reaction and Mannich-type imminium electrophile. *J. Org. Chem.* **2006**, *71*, 3332–3334.

8. Crystallographic Data

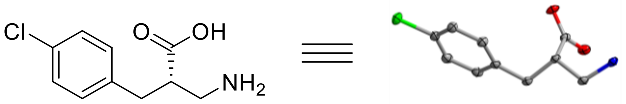

Crystal data and structure refinement.

Identification code	12992	
Empirical formula	$C_{10}H_{12}ClNO_2$	
Color	colourless	
Formula weight	213.66 g · mol^{-1}	
Temperature	100(2) K	
Wavelength	0.71073 Å	
Crystal system	MONOCLINIC	
Space group	**P2$_1$, (no. 4)**	
Unit cell dimensions	a = 6.5737(4) Å	α= 90°.
	b = 6.2674(3) Å	β= 97.520(5)°.
	c = 12.4511(12) Å	γ = 90°.
Volume	508.57(6) Å^3	
Z	2	
Density (calculated)	1.395 Mg · m^{-3}	
Absorption coefficient	0.348 mm^{-1}	
F(000)	224 e	
Crystal size	0.25 x 0.09 x 0.06 mm^3	
θ range for data collection	3.126 to 33.142°.	
Index ranges	-10 ≤ h ≤ 10, -9≤ k ≤ 9, -19 ≤ l ≤ 19	
Reflections collected	16619	
Independent reflections	3858 [R_{int} = 0.0433]	
Reflections with I>2σ(I)	3612	
Completeness to θ = 25.242°	99.4 %	
Absorption correction	Gaussian	
Max. and min. transmission	0.98 and 0.94	
Refinement method	Full-matrix least-squares on F^2	
Data / restraints / parameters	3858 / 1 / 151	
Goodness-of-fit on F^2	1.088	
Final R indices [I>2σ(I)]	R_1 = 0.0360	wR^2 = 0.0928
R indices (all data)	R_1 = 0.0400	wR^2 = 0.0958
Absolute structure parameter	-0.01(3)	
Remarks	**H-atoms at C2, C3 and N1 found !**	
Largest diff. peak and hole	0.6 and -0.4 e · Å^{-3}	

Bond lengths [Å] and angles [°].

Cl(1)-C(8)	1.7409(17)	O(1)-C(1)	1.251(2)
O(2)-C(1)	1.2643(18)	N(1)-C(3)	1.492(2)
N(1)-H(1A)	0.80(3)	N(1)-H(1B)	0.84(3)
N(1)-H(1C)	0.85(4)	C(1)-C(2)	1.538(2)
C(5)-C(10)	1.396(2)	C(5)-C(4)	1.509(2)
C(5)-C(6)	1.400(2)	C(10)-C(9)	1.395(2)
C(9)-C(8)	1.385(3)	C(4)-C(2)	1.543(2)
C(7)-C(8)	1.387(3)	C(7)-C(6)	1.392(2)
C(2)-C(3)	1.525(2)	C(2)-H(2)	0.98(3)
C(3)-H(3A)	0.93(3)	C(3)-H(3B)	0.96(3)
C(3)-N(1)-H(1A)	114(2)	C(3)-N(1)-H(1B)	110.8(19)
C(3)-N(1)-H(1C)	106(2)	H(1A)-N(1)-H(1B)	110(3)
H(1A)-N(1)-H(1C)	102(3)	H(1B)-N(1)-H(1C)	113(3)
O(1)-C(1)-O(2)	125.51(14)	O(1)-C(1)-C(2)	116.66(13)
O(2)-C(1)-C(2)	117.79(14)	C(10)-C(5)-C(4)	120.48(15)
C(10)-C(5)-C(6)	118.41(15)	C(6)-C(5)-C(4)	121.01(14)
C(9)-C(10)-C(5)	121.14(15)	C(8)-C(9)-C(10)	119.02(16)
C(5)-C(4)-C(2)	115.67(12)	C(8)-C(7)-C(6)	119.16(16)
C(1)-C(2)-C(4)	108.92(12)	C(1)-C(2)-H(2)	108.1(16)
C(4)-C(2)-H(2)	106.9(16)	C(3)-C(2)-C(1)	112.34(13)
C(3)-C(2)-C(4)	107.48(12)	C(3)-C(2)-H(2)	112.8(17)
C(9)-C(8)-Cl(1)	119.74(14)	C(9)-C(8)-C(7)	121.26(16)
C(7)-C(8)-Cl(1)	118.99(15)	N(1)-C(3)-C(2)	112.75(13)
N(1)-C(3)-H(3A)	108.2(17)	N(1)-C(3)-H(3B)	104.4(15)
C(2)-C(3)-H(3A)	107.9(19)	C(2)-C(3)-H(3B)	111.3(16)
H(3A)-C(3)-H(3B)	112(2)	C(7)-C(6)-C(5)	121.00(16)

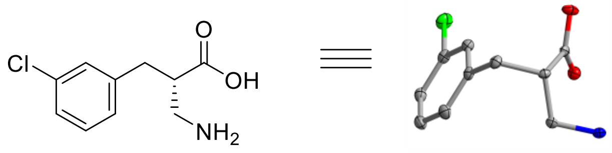

Crystal data and structure refinement.

Identification code	12998
Empirical formula	$C_{10} H_{12} Cl N O_2$
Color	colourless

Formula weight	213.66 g · mol^{-1}
Temperature	100(2) K
Wavelength	0.71073 Å
Crystal system	ORTHORHOMBIC
Space group	**$P2_12_12_1$, (no. 19)**
Unit cell dimensions	a = 5.3943(5) Å α= 90°.
	b = 6.7038(7) Å β= 90°.
	c = 28.063(3) Å γ = 90°.
Volume	1014.82(18) $Å^3$
Z	4
Density (calculated)	1.398 Mg · m^{-3}
Absorption coefficient	0.349 mm^{-1}
F(000)	448 e
Crystal size	0.26 x 0.08 x 0.05 mm^3
θ range for data collection	2.903 to 33.199°.
Index ranges	-8 ≤ h ≤ 7, -10≤ k ≤ 10, -43 ≤ l ≤ 43
Reflections collected	19048
Independent reflections	3897 [R_{int} = 0.0436]
Reflections with I>2σ(I)	3481
Completeness to θ = 25.242°	99.9 %
Absorption correction	Gaussian
Max. and min. transmission	0.98 and 0.93
Refinement method	Full-matrix least-squares on F^2
Data / restraints / parameters	3897 / 0 / 151
Goodness-of-fit on F^2	1.075
Final R indices [I>2σ(I)]	R_1 = 0.0311 wR^2 = 0.0733
R indices (all data)	R_1 = 0.0398 wR^2 = 0.0773
Absolute structure parameter	0.03(2)
Remarks	H-atoms at C2,C3 and NH_3-group are found !
Largest diff. peak and hole	0.3 and -0.3 e · $Å^{-3}$

Bond lengths [Å] and angles [°].

Cl(1)-C(7)	1.7457(16)	O(1)-C(1)	1.2479(19)
O(2)-C(1)	1.2686(17)	N(1)-H(1A)	0.91(2)
N(1)-H(1B)	0.88(3)	N(1)-H(1C)	0.94(2)
N(1)-C(3)	1.4934(18)	C(1)-C(2)	1.531(2)
C(2)-H(2)	0.98(2)	C(2)-C(3)	1.5181(19)

C(2)-C(4)	1.546(2)	C(3)-H(3A)	0.98(2)
C(3)-H(3B)	0.99(2)	C(4)-C(5)	1.509(2)
C(5)-C(6)	1.399(2)	C(5)-C(10)	1.395(2)
C(6)-C(7)	1.385(2)	C(7)-C(8)	1.388(2)
C(8)-C(9)	1.386(2)	C(9)-C(10)	1.391(2)
H(1A)-N(1)-H(1B)	108(2)	H(1A)-N(1)-H(1C)	108(2)
H(1B)-N(1)-H(1C)	103(2)	C(3)-N(1)-H(1A)	112.7(14)
C(3)-N(1)-H(1B)	112.6(16)	C(3)-N(1)-H(1C)	111.7(13)
O(1)-C(1)-O(2)	124.66(14)	O(1)-C(1)-C(2)	118.92(12)
O(2)-C(1)-C(2)	116.42(13)	C(1)-C(2)-H(2)	106.1(12)
C(1)-C(2)-C(4)	110.68(11)	C(3)-C(2)-C(1)	112.20(11)
C(3)-C(2)-H(2)	109.1(13)	C(3)-C(2)-C(4)	112.29(11)
C(4)-C(2)-H(2)	106.1(13)	N(1)-C(3)-C(2)	110.75(11)
N(1)-C(3)-H(3A)	108.1(12)	N(1)-C(3)-H(3B)	105.5(13)
C(2)-C(3)-H(3A)	112.3(13)	C(2)-C(3)-H(3B)	111.4(14)
H(3A)-C(3)-H(3B)	108.5(17)	C(5)-C(4)-C(2)	114.32(12)
C(6)-C(5)-C(4)	119.83(13)	C(10)-C(5)-C(4)	121.86(13)
C(10)-C(5)-C(6)	118.30(14)	C(7)-C(6)-C(5)	119.87(14)
C(6)-C(7)-Cl(1)	118.67(12)	C(6)-C(7)-C(8)	122.01(14)
C(8)-C(7)-Cl(1)	119.33(12)	C(9)-C(8)-C(7)	118.12(14)
C(8)-C(9)-C(10)	120.67(14)	C(9)-C(10)-C(5)	121.01(14)

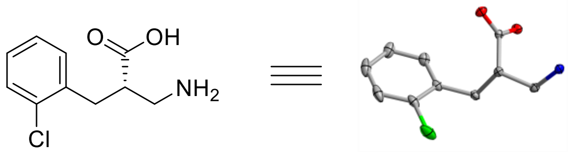

Crystal data and structure refinement.

Identification code	12994	
Empirical formula	$C_{10} H_{14} Cl N O_3$	
Color	colourless	
Formula weight	231.67 $g \cdot mol^{-1}$	
Temperature	100(2) K	
Wavelength	0.71073 Å	
Crystal system	MONOCLINIC	
Space group	**$P2_1$, (no. 4)**	
Unit cell dimensions	a = 8.2774(4) Å	α= 90°.
	b = 8.4515(4) Å	β= 94.717(2)°.

	c = 15.9735(8) Å	γ = 90°.
Volume	1113.66(9) $Å^3$	
Z	4	
Density (calculated)	1.382 Mg · m^{-3}	
Absorption coefficient	0.330 mm^{-1}	
F(000)	488 e	
Crystal size	0.124 x 0.101 x 0.091 mm^3	
θ range for data collection	1.279 to 33.504°.	
Index ranges	-12 ≤ h ≤ 12, -13≤ k ≤ 13, -24 ≤ l ≤ 24	
Reflections collected	41184	
Independent reflections	8687 [R_{int} = 0.0299]	
Reflections with I>2σ(I)	8117	
Completeness to θ = 25.242°	100.0 %	
Absorption correction	Gaussian	
Max. and min. transmission	0.98 and 0.97	
Refinement method	Full-matrix least-squares on F^2	
Data / restraints / parameters	8687 / 1 / 319	
Goodness-of-fit on F^2	1.017	
Final R indices [I>2σ(I)]	R_1 = 0.0317	wR^2 = 0.0808
R indices (all data)	R_1 = 0.0350	wR^2 = 0.0827
Absolute structure parameter	-0.062(12)	
Largest diff. peak and hole	0.8 and -0.3 e · $Å^{-3}$	

Bond lengths [Å] and angles [°].

Cl(1)-C(6)	1.7464(16)	O(1)-C(4)	1.2522(17)
O(2)-C(4)	1.2660(17)	N(1)-H(1A)	0.80(3)
N(1)-H(1B)	0.85(2)	N(1)-H(1C)	0.90(2)
N(1)-C(3)	1.4855(19)	C(1)-C(2)	1.548(2)
C(1)-C(5)	1.502(2)	C(2)-H(2)	0.98(2)
C(2)-C(3)	1.529(2)	C(2)-C(4)	1.5376(19)
C(5)-C(6)	1.398(2)	C(5)-C(10)	1.403(2)
C(6)-C(7)	1.390(2)	C(7)-C(8)	1.387(3)
C(8)-C(9)	1.386(3)	C(9)-C(10)	1.389(2)
Cl(2)-C(16)	1.7383(18)	O(3)-C(14)	1.2543(18)
O(4)-C(14)	1.2578(18)	N(2)-H(2A)	0.90(2)
N(2)-H(2B)	0.88(3)	N(2)-H(2C)	0.88(3)
N(2)-C(13)	1.4889(19)	C(11)-C(12)	1.550(2)

C(11)-C(15)	1.504(2)	C(12)-H(12)	0.94(2)
C(12)-C(13)	1.514(2)	C(12)-C(14)	1.5318(19)
C(15)-C(16)	1.399(2)	C(15)-C(20)	1.398(2)
C(16)-C(17)	1.391(3)	C(17)-C(18)	1.381(3)
C(18)-C(19)	1.387(3)	C(19)-C(20)	1.392(2)
O(5)-H(5A)	0.82(3)	O(5)-H(5B)	0.87(3)
O(6)-H(6A)	0.74(3)	O(6)-H(6B)	0.82(3)
H(1A)-N(1)-H(1B)	106(2)	H(1A)-N(1)-H(1C)	100(2)
H(1B)-N(1)-H(1C)	115(2)	C(3)-N(1)-H(1A)	113.3(17)
C(3)-N(1)-H(1B)	110.6(15)	C(3)-N(1)-H(1C)	111.6(15)
C(5)-C(1)-C(2)	114.41(12)	C(1)-C(2)-H(2)	110.9(12)
C(3)-C(2)-C(1)	107.24(11)	C(3)-C(2)-H(2)	109.5(13)
C(3)-C(2)-C(4)	110.37(11)	C(4)-C(2)-C(1)	111.55(11)
C(4)-C(2)-H(2)	107.3(13)	N(1)-C(3)-C(2)	114.00(12)
O(1)-C(4)-O(2)	124.43(13)	O(1)-C(4)-C(2)	118.07(12)
O(2)-C(4)-C(2)	117.46(12)	C(6)-C(5)-C(1)	122.15(14)
C(6)-C(5)-C(10)	116.69(14)	C(10)-C(5)-C(1)	121.15(14)
C(5)-C(6)-Cl(1)	119.33(12)	C(7)-C(6)-Cl(1)	118.23(12)
C(7)-C(6)-C(5)	122.44(14)	C(8)-C(7)-C(6)	119.25(15)
C(9)-C(8)-C(7)	119.98(16)	C(8)-C(9)-C(10)	120.04(15)
C(9)-C(10)-C(5)	121.60(15)	H(2A)-N(2)-H(2B)	111(2)
H(2A)-N(2)-H(2C)	106(2)	H(2B)-N(2)-H(2C)	106(2)
C(13)-N(2)-H(2A)	108.7(17)	C(13)-N(2)-H(2B)	110.2(16)
C(13)-N(2)-H(2C)	115.0(17)	C(15)-C(11)-C(12)	113.95(12)
C(11)-C(12)-H(12)	112.8(12)	C(13)-C(12)-C(11)	107.22(11)
C(13)-C(12)-H(12)	108.0(12)	C(13)-C(12)-C(14)	110.67(11)
C(14)-C(12)-C(11)	108.98(11)	C(14)-C(12)-H(12)	109.1(12)
N(2)-C(13)-C(12)	112.69(12)	O(3)-C(14)-O(4)	124.15(13)
O(3)-C(14)-C(12)	117.92(12)	O(4)-C(14)-C(12)	117.92(12)
C(16)-C(15)-C(11)	122.42(14)	C(20)-C(15)-C(11)	120.85(14)
C(20)-C(15)-C(16)	116.73(14)	C(15)-C(16)-Cl(2)	119.68(12)
C(17)-C(16)-Cl(2)	117.83(14)	C(17)-C(16)-C(15)	122.48(16)
C(18)-C(17)-C(16)	119.13(17)	C(17)-C(18)-C(19)	120.11(17)
C(18)-C(19)-C(20)	120.03(17)	C(19)-C(20)-C(15)	121.43(16)
H(5A)-O(5)-H(5B)	104(3)	H(6A)-O(6)-H(6B)	113(3)

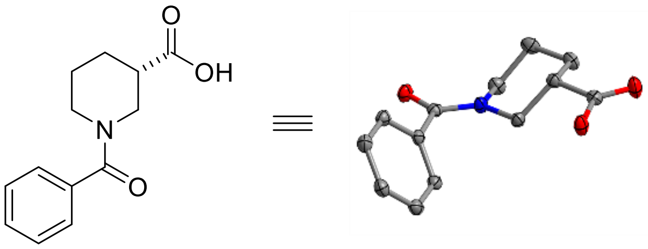

Crystal data and structure refinement.

Identification code	13452	
Empirical formula	$C_{13} H_{15} N O_3$	
Color	colourless	
Formula weight	233.26 g·mol^{-1}	
Temperature	100(2) K	
Wavelength	1.54178 Å	
Crystal system	MONOCLINIC	
Space group	**$P2_1$, (no. 4)**	
Unit cell dimensions	a = 5.9188(3) Å	α= 90°.
	b = 17.9358(9) Å	β= 101.130(2)°.
	c = 10.8133(6) Å	γ = 90°.
Volume	1126.33(10) Å^3	
Z	4	
Density (calculated)	1.376 Mg · m^{-3}	
Absorption coefficient	0.804 mm^{-1}	
F(000)	496 e	
Crystal size	0.221 x 0.125 x 0.107 mm^3	
θ range for data collection	4.167 to 72.056°.	
Index ranges	$-7 \le h \le 7$, $-22 \le k \le 22$, $-13 \le l \le 13$	
Reflections collected	38156	
Independent reflections	4108 [R_{int} = 0.0329]	
Reflections with I>2σ(I)	3992	
Completeness to θ = 67.679°	100.0 %	
Absorption correction	Gaussian	
Max. and min. transmission	0.96and 0.92	
Refinement method	Full-matrix least-squares on F^2	
Data / restraints / parameters	4108 / 1 / 324	
Goodness-of-fit on F^2	1.149	
Final R indices [I>2σ(I)]	R_1 = 0.0303	wR^2 = 0.0783
R indices (all data)	R_1 = 0.0320	wR^2 = 0.0800
Absolute structure parameter	-0.03(5)	

Extinction coefficient	0.0066(7)
Largest diff. peak and hole	0.2 and -0.2 e · Å^{-3}

Bond lengths [Å] and angles [°].

O(1)-C(1)	1.245(3)	O(2)-C(7)	1.208(3)
O(3)-H(3)	0.91(4)	O(3)-C(7)	1.332(3)
N(1)-C(1)	1.339(3)	N(1)-C(2)	1.464(3)
N(1)-C(6)	1.477(3)	C(1)-C(8)	1.504(3)
C(2)-C(3)	1.537(3)	C(3)-H(3A)	0.99(3)
C(3)-C(4)	1.526(3)	C(3)-C(7)	1.517(3)
C(4)-C(5)	1.522(3)	C(5)-C(6)	1.519(3)
C(8)-C(9)	1.393(3)	C(8)-C(13)	1.393(3)
C(9)-C(10)	1.386(3)	C(10)-C(11)	1.390(3)
C(11)-C(12)	1.390(3)	C(12)-C(13)	1.387(3)
O(4)-C(21)	1.247(3)	O(5)-C(27)	1.202(3)
O(6)-H(6)	0.88(4)	O(6)-C(27)	1.335(3)
N(2)-C(21)	1.341(3)	N(2)-C(22)	1.470(3)
N(2)-C(26)	1.469(3)	C(21)-C(28)	1.498(3)
C(22)-C(23)	1.532(3)	C(23)-H(23)	0.98(3)
C(23)-C(24)	1.523(3)	C(23)-C(27)	1.520(3)
C(24)-C(25)	1.525(3)	C(25)-C(26)	1.517(3)
C(28)-C(29)	1.395(3)	C(28)-C(33)	1.395(3)
C(29)-C(30)	1.387(3)	C(30)-C(31)	1.383(3)
C(31)-C(32)	1.391(3)	C(32)-C(33)	1.385(3)
C(7)-O(3)-H(3)	112(3)	C(1)-N(1)-C(2)	125.06(18)
C(1)-N(1)-C(6)	120.40(18)	C(2)-N(1)-C(6)	114.38(18)
O(1)-C(1)-N(1)	121.7(2)	O(1)-C(1)-C(8)	118.4(2)
N(1)-C(1)-C(8)	119.93(19)	N(1)-C(2)-C(3)	109.69(17)
C(2)-C(3)-H(3A)	107.0(15)	C(4)-C(3)-C(2)	110.42(17)
C(4)-C(3)-H(3A)	109.1(15)	C(7)-C(3)-C(2)	111.47(17)
C(7)-C(3)-H(3A)	106.8(15)	C(7)-C(3)-C(4)	111.86(18)
C(5)-C(4)-C(3)	110.09(19)	C(6)-C(5)-C(4)	111.17(18)
N(1)-C(6)-C(5)	110.16(18)	O(2)-C(7)-O(3)	123.4(2)
O(2)-C(7)-C(3)	124.8(2)	O(3)-C(7)-C(3)	111.76(18)
C(9)-C(8)-C(1)	118.6(2)	C(13)-C(8)-C(1)	121.6(2)
C(13)-C(8)-C(9)	119.6(2)	C(8)-C(9)-H(9)	119.9

C(10)-C(9)-C(8)	120.3(2)	C(9)-C(10)-C(11)	120.1(2)
C(12)-C(11)-C(10)	119.6(2)	C(13)-C(12)-C(11)	120.4(2)
C(12)-C(13)-C(8)	119.9(2)	C(27)-O(6)-H(6)	108(2)
C(21)-N(2)-C(22)	124.94(18)	C(21)-N(2)-C(26)	119.93(18)
C(26)-N(2)-C(22)	115.03(17)	O(4)-C(21)-N(2)	121.0(2)
O(4)-C(21)-C(28)	118.9(2)	N(2)-C(21)-C(28)	120.03(19)
N(2)-C(22)-C(23)	111.12(17)	C(22)-C(23)-H(23)	108.1(16)
C(24)-C(23)-C(22)	111.85(17)	C(24)-C(23)-H(23)	110.4(16)
C(27)-C(23)-C(22)	110.60(18)	C(27)-C(23)-H(23)	105.2(15)
C(27)-C(23)-C(24)	110.43(18)	C(23)-C(24)-C(25)	110.69(19)
C(26)-C(25)-C(24)	110.11(19)	N(2)-C(26)-C(25)	111.14(17)
O(5)-C(27)-O(6)	123.1(2)	O(5)-C(27)-C(23)	124.2(2)
O(6)-C(27)-C(23)	112.67(19)	C(29)-C(28)-C(21)	122.3(2)
C(29)-C(28)-C(33)	119.3(2)	C(33)-C(28)-C(21)	118.2(2)
C(30)-C(29)-C(28)	120.2(2)	C(31)-C(30)-C(29)	120.1(2)
C(30)-C(31)-C(32)	120.1(2)	C(33)-C(32)-C(31)	120.0(2)
C(32)-C(33)-C(28)	120.3(2)		

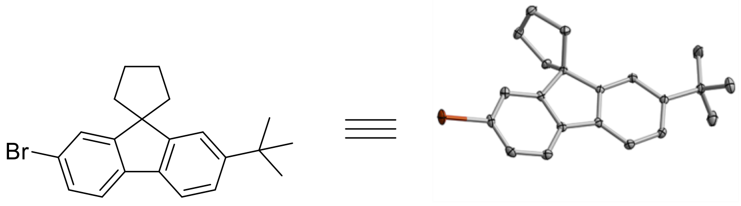

Crystal data and structure refinement.

Identification code	12675	
Empirical formula	$C_{21} H_{23} Br$	
Color	colourless	
Formula weight	355.30 g · mol^{-1}	
Temperature	100(2) K	
Wavelength	0.71073 Å	
Crystal system	MONOCLINIC	
Space group	**C2/c, (no. 15)**	
Unit cell dimensions	a = 26.513(5) Å	α= 90°.
	b = 6.6908(13) Å	β= 119.06(3)°.
	c = 22.337(5) Å	γ = 90°.
Volume	3463.6(15) Å^3	
Z	8	

Density (calculated)	1.363 Mg · m^{-3}
Absorption coefficient	2.369 mm^{-1}
F(000)	1472 e
Crystal size	0.14 x 0.08 x 0.07 mm^3
θ range for data collection	3.097 to 29.574°.
Index ranges	-36 ≤ h ≤ 36, -9≤ k ≤ 7, -31 ≤ l ≤ 31
Reflections collected	18801
Independent reflections	4816 [R_{int} = 0.0265]
Reflections with I>2σ(I)	4223
Completeness to θ = 25.242°	99.4 %
Absorption correction	Gaussian
Max. and min. transmission	0.87 and 0.76
Refinement method	Full-matrix least-squares on F^2
Data / restraints / parameters	4816 / 0 / 202
Goodness-of-fit on F^2	1.094
Final R indices [I>2σ(I)]	R_1 = 0.0258 wR^2 = 0.0582
R indices (all data)	R_1 = 0.0332 wR^2 = 0.0615
Largest diff. peak and hole	0.5 and -0.4 e · Å^{-3}

Bond lengths [Å] and angles [°].

Br(1)-C(1)	1.9046(13)	C(1)-C(2)	1.386(2)
C(1)-C(13)	1.3958(19)	C(2)-C(3)	1.3958(18)
C(3)-C(4)	1.3906(18)	C(4)-C(5)	1.4662(17)
C(4)-C(12)	1.4069(18)	C(5)-C(6)	1.3932(18)
C(5)-C(10)	1.3973(18)	C(6)-C(7)	1.3879(18)
C(7)-C(8)	1.4077(19)	C(8)-C(9)	1.4021(18)
C(8)-C(18)	1.5336(18)	C(9)-C(10)	1.3951(17)
C(10)-C(11)	1.5195(18)	C(11)-C(12)	1.5283(18)
C(11)-C(14)	1.5483(18)	C(11)-C(17)	1.545(2)
C(12)-C(13)	1.3914(17)	C(14)-C(15)	1.5381(19)
C(15)-C(16)	1.548(2)	C(16)-C(17)	1.5406(19)
C(18)-C(19)	1.531(2)	C(18)-C(20)	1.535(2)
C(18)-C(21)	1.537(2)		
C(2)-C(1)-Br(1)	118.51(10)	C(2)-C(1)-C(13)	122.98(12)
C(13)-C(1)-Br(1)	118.50(11)	C(1)-C(2)-C(3)	119.05(12)
C(4)-C(3)-C(2)	119.04(13)	C(3)-C(4)-C(5)	130.24(12)

C(3)-C(4)-C(12)	121.23(12)	C(12)-C(4)-C(5)	108.52(11)
C(6)-C(5)-C(4)	131.12(12)	C(6)-C(5)-C(10)	120.44(12)
C(10)-C(5)-C(4)	108.40(11)	C(7)-C(6)-C(5)	118.68(12)
C(6)-C(7)-C(8)	122.17(12)	C(7)-C(8)-C(18)	118.93(12)
C(9)-C(8)-C(7)	118.18(12)	C(9)-C(8)-C(18)	122.90(12)
C(10)-C(9)-C(8)	120.13(12)	C(5)-C(10)-C(11)	111.35(11)
C(9)-C(10)-C(5)	120.41(12)	C(9)-C(10)-C(11)	128.23(12)
C(10)-C(11)-C(12)	101.14(10)	C(10)-C(11)-C(14)	113.86(11)
C(10)-C(11)-C(17)	113.98(11)	C(12)-C(11)-C(14)	112.61(11)
C(12)-C(11)-C(17)	113.74(11)	C(17)-C(11)-C(14)	102.05(10)
C(4)-C(12)-C(11)	110.56(11)	C(13)-C(12)-C(4)	120.03(12)
C(13)-C(12)-C(11)	129.41(12)	C(12)-C(13)-C(1)	117.68(13)
C(15)-C(14)-C(11)	105.61(11)	C(14)-C(15)-C(16)	106.09(11)
C(17)-C(16)-C(15)	106.21(11)	C(16)-C(17)-C(11)	106.16(11)
C(8)-C(18)-C(20)	109.39(12)	C(8)-C(18)-C(21)	109.78(11)
C(19)-C(18)-C(8)	112.31(11)	C(19)-C(18)-C(20)	107.74(13)
C(19)-C(18)-C(21)	108.61(13)	C(20)-C(18)-C(21)	108.93(12)

9. HPLC Traces of the Products

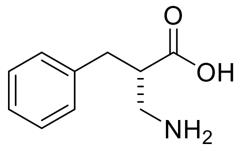

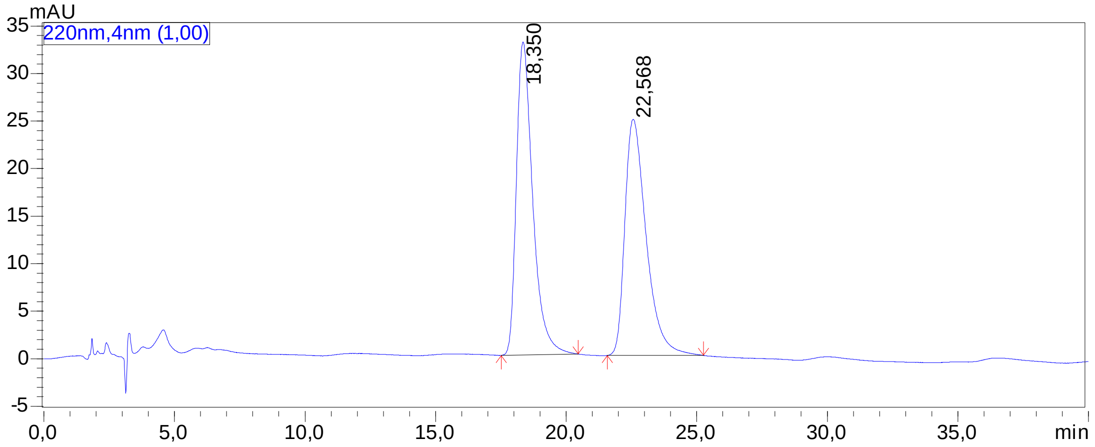

Peak#	Ret. Time	Area%
1	18.350	50.395
2	22.568	49.605
Total		100.000

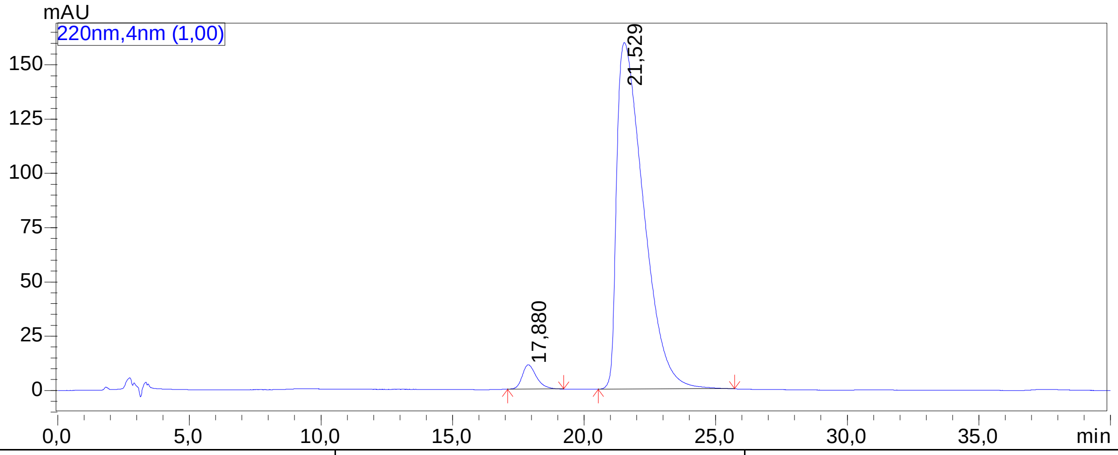

Peak#	Ret. Time	Area%
1	17.880	3.610
2	21.529	96.390
Total		100.000

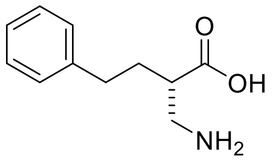

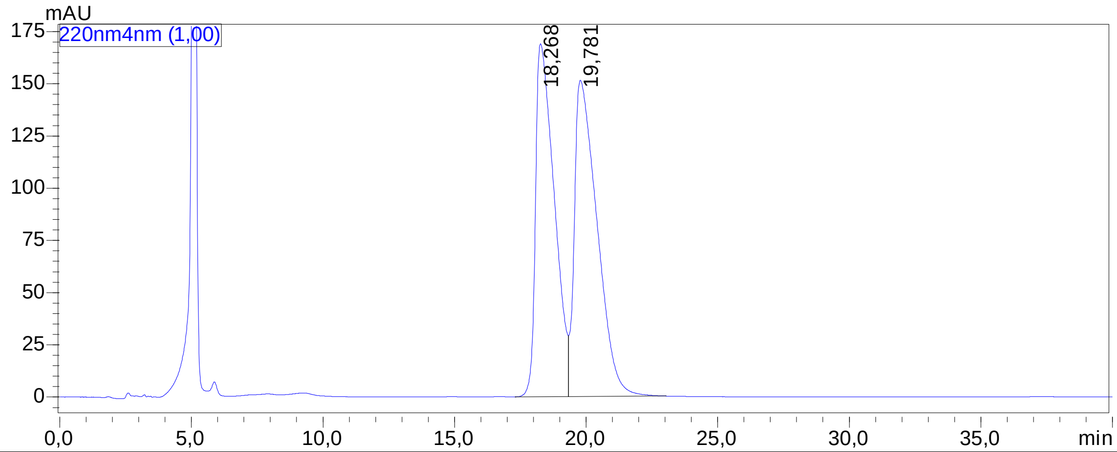

Peak#	Ret. Time	Area%
1	18.268	47.874
2	19.781	52.126
Total		100.000

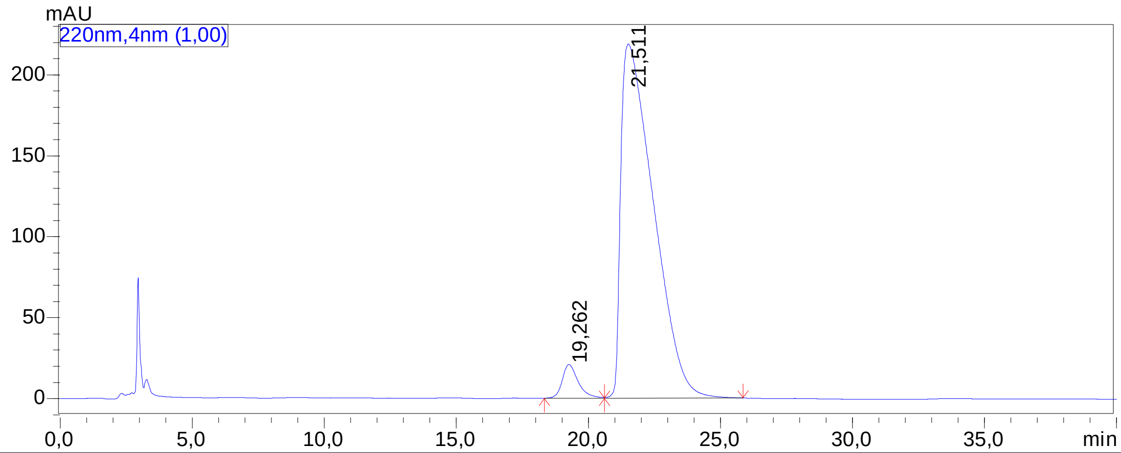

Peak#	Ret. Time	Area%
1	19.262	4.441
2	21.511	95.559
Total		100.000

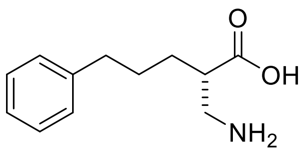

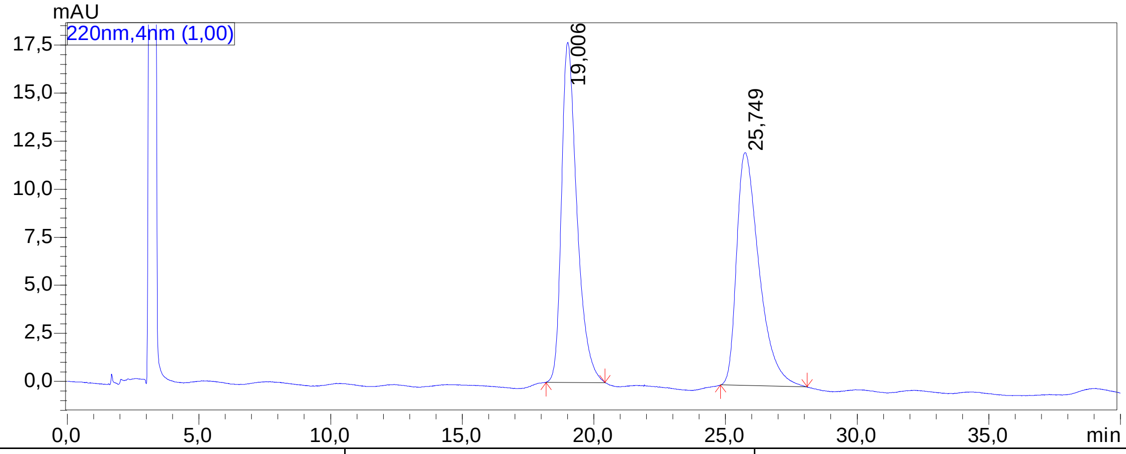

Peak#	Ret. Time	Area%
1	19.006	50.089
2	25.749	49.911
Total		100.000

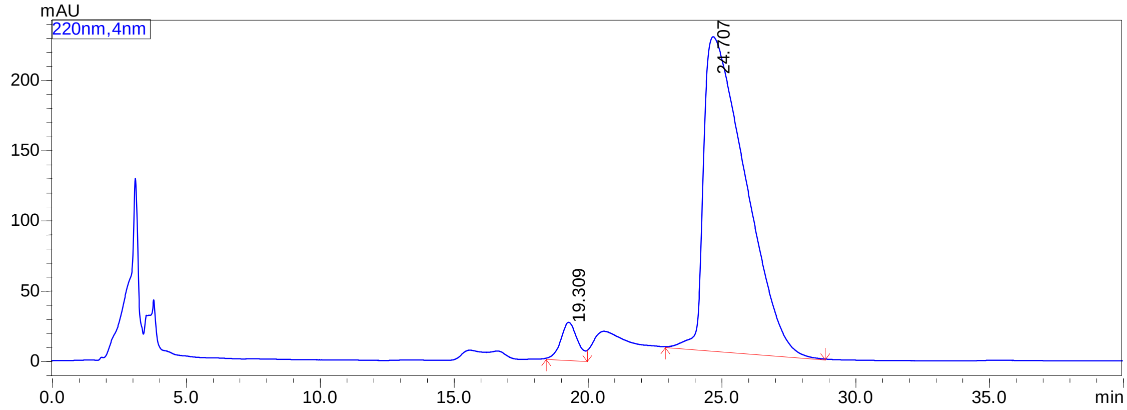

Peak#	Ret. Time	Area%
1	19.309	3.278
2	24.707	96.722
Total		100.000

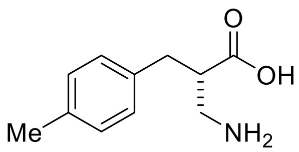

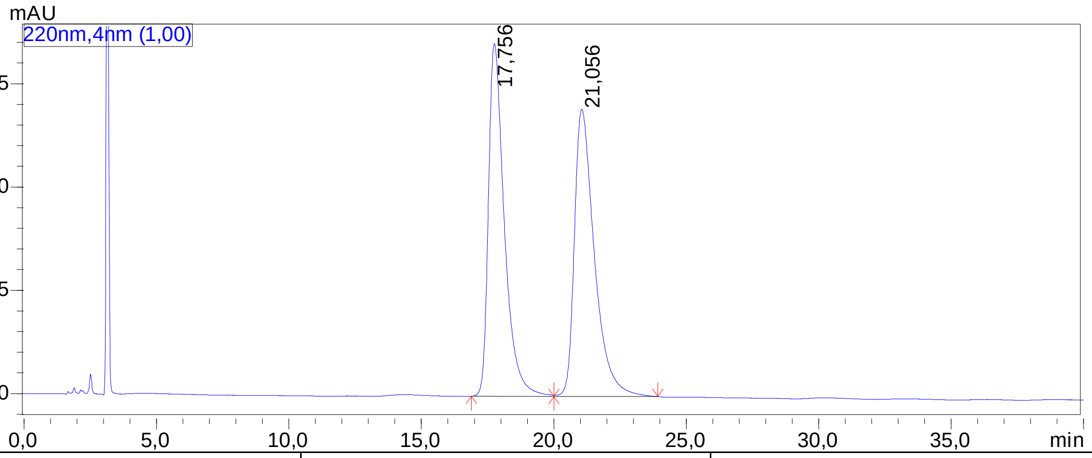

Peak#	Ret. Time	Area%
1	17.756	49.434
2	21.056	50.566
Total		100.000

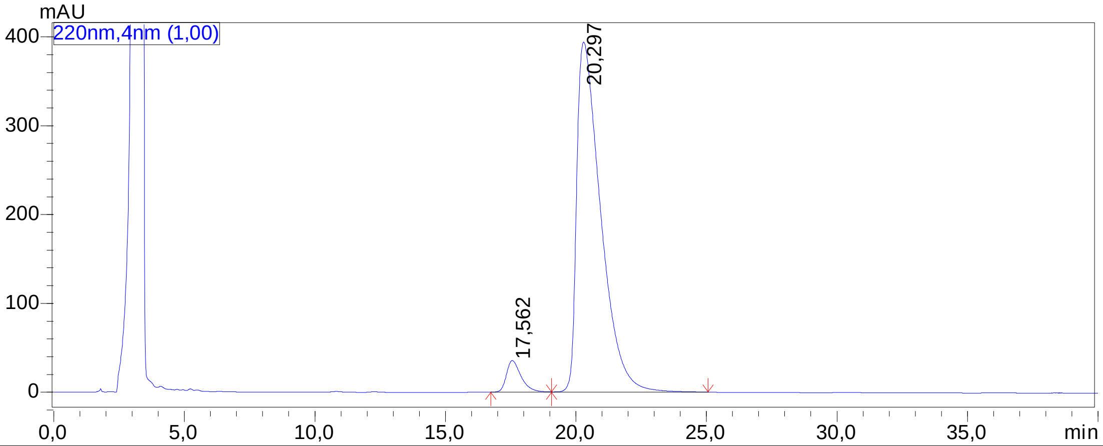

Peak#	Ret. Time	Area%
1	17.562	5.083
2	20.297	94.917
Total		100.000

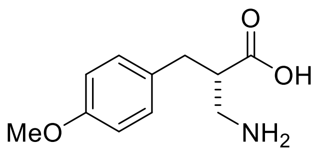

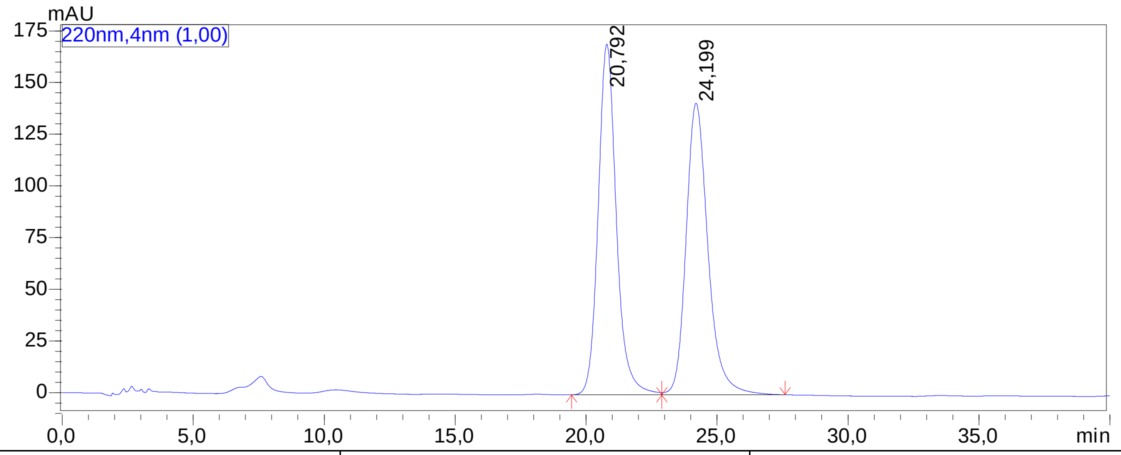

Peak#	Ret. Time	Area%
1	20.792	49.929
2	24.199	50.071
Total		100.000

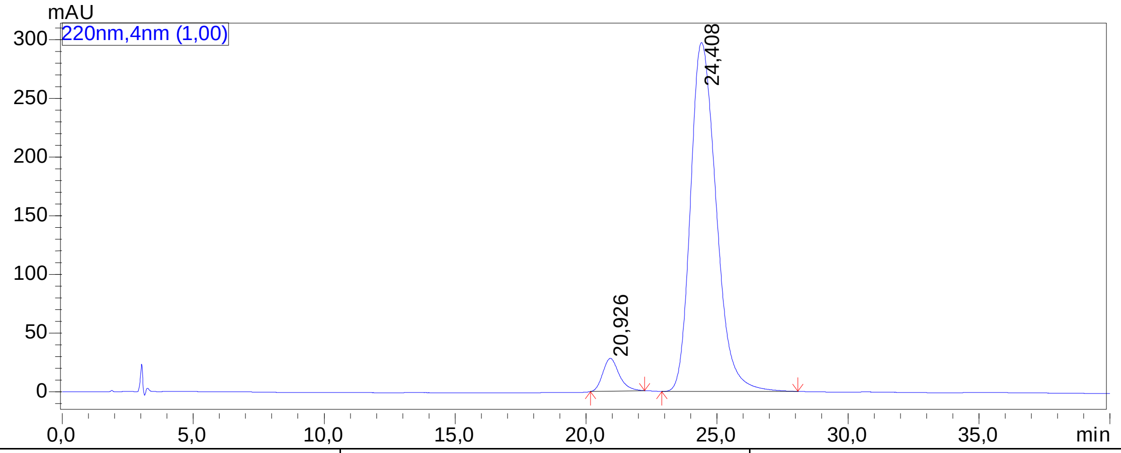

Peak#	Ret. Time	Area%
1	20.926	5.547
2	24.408	94.453
Total		100.000

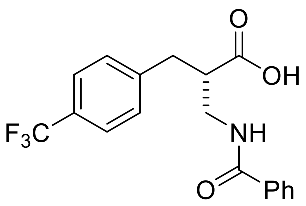

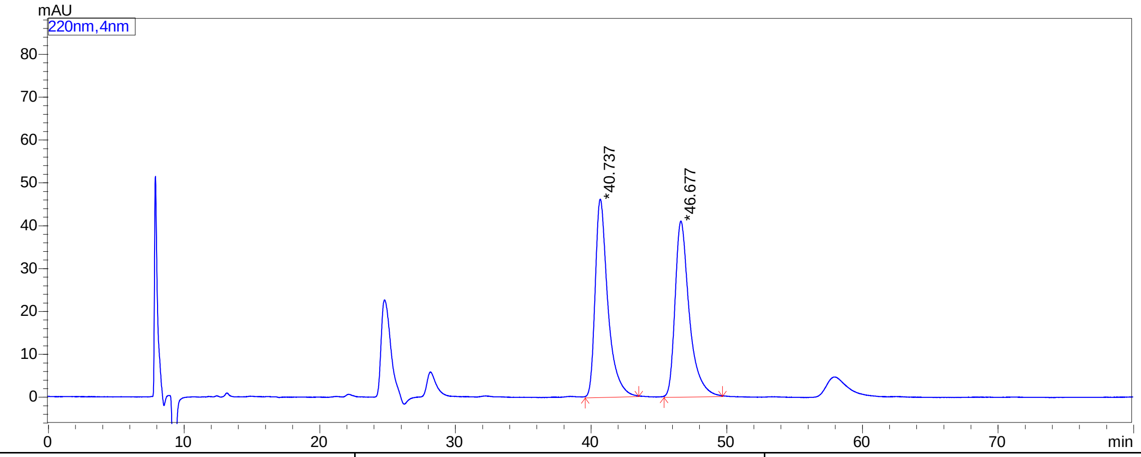

Peak#	Ret. Time	Area%
1	40.737	50.122
2	46.677	49.878
Total		100.000

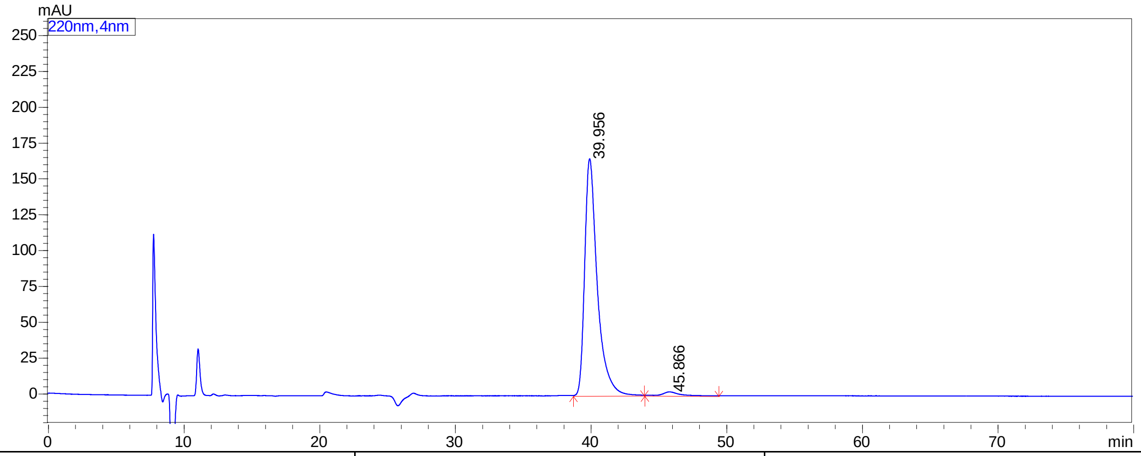

Peak#	Ret. Time	Area%
1	39.956	98.524
2	45.866	1.476
Total		100.000

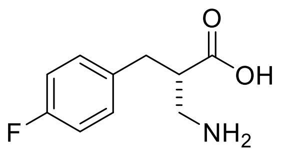

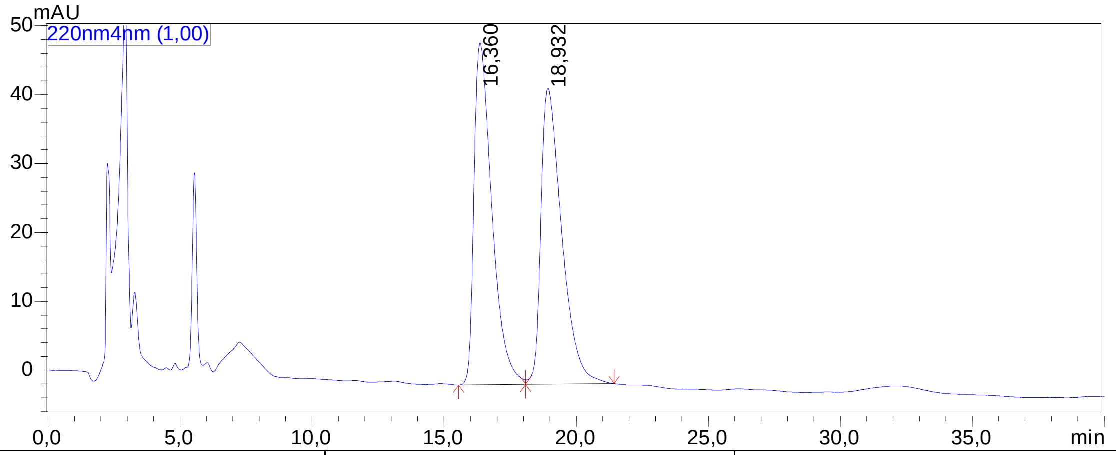

Peak#	Ret. Time	Area%
1	16.360	49.873
2	18.932	50.127
Total		100.000

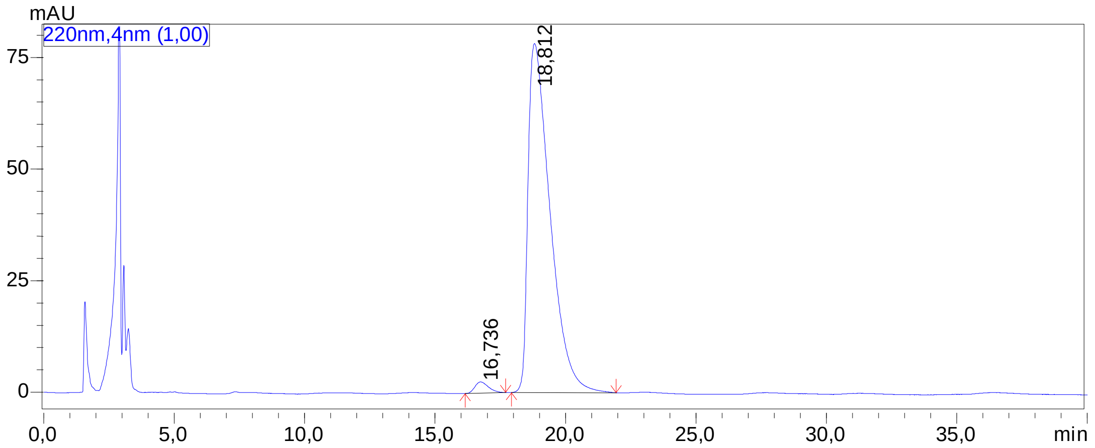

Peak#	Ret. Time	Area%
1	16.736	1.963
2	18.812	98.037
Total		100.000

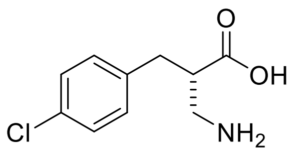

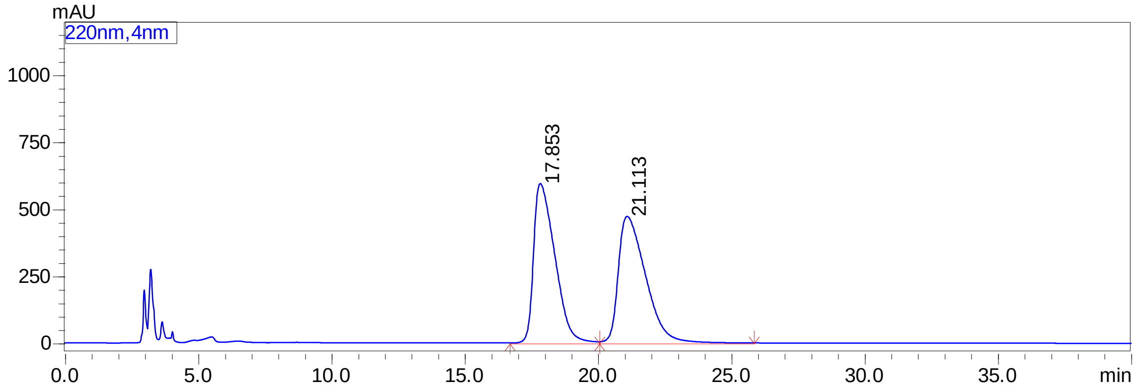

Peak#	Ret. Time	Area%
1	17.853	49.554
2	21.113	50.446
Total		100.000

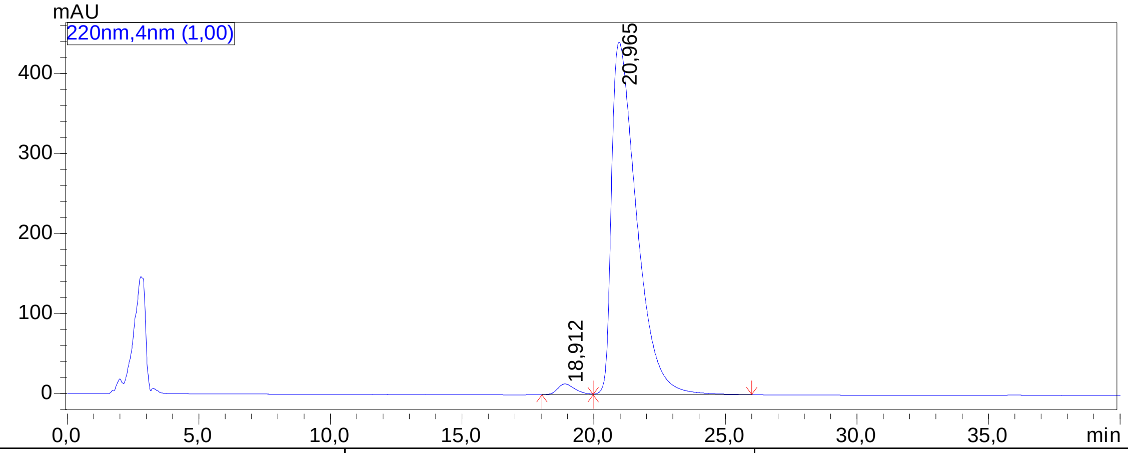

Peak#	Ret. Time	Area%
1	18.912	2.077
2	20.965	97.923
Total		100.000

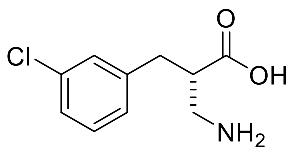

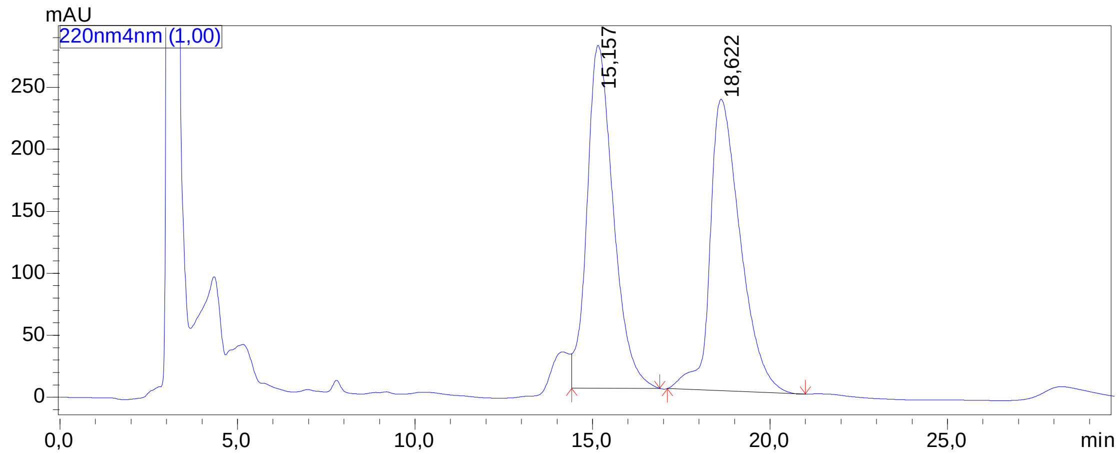

Peak#	Ret. Time	Area%
1	15.157	50.418
2	18.622	49.582
Total		100.000

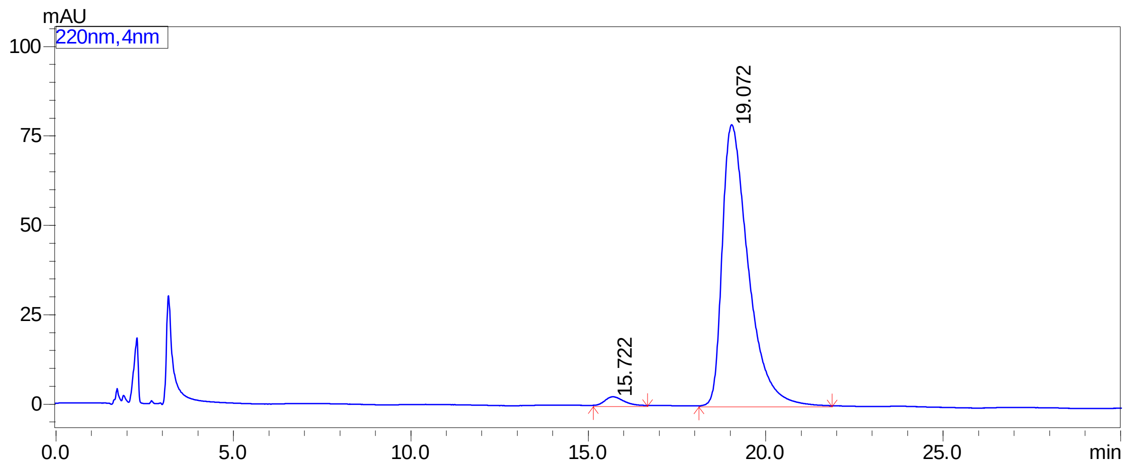

Peak#	Ret. Time	Area%
1	15.722	2.125
2	19.072	97.875
Total		100.000

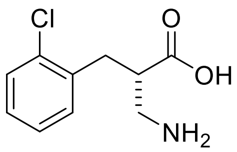

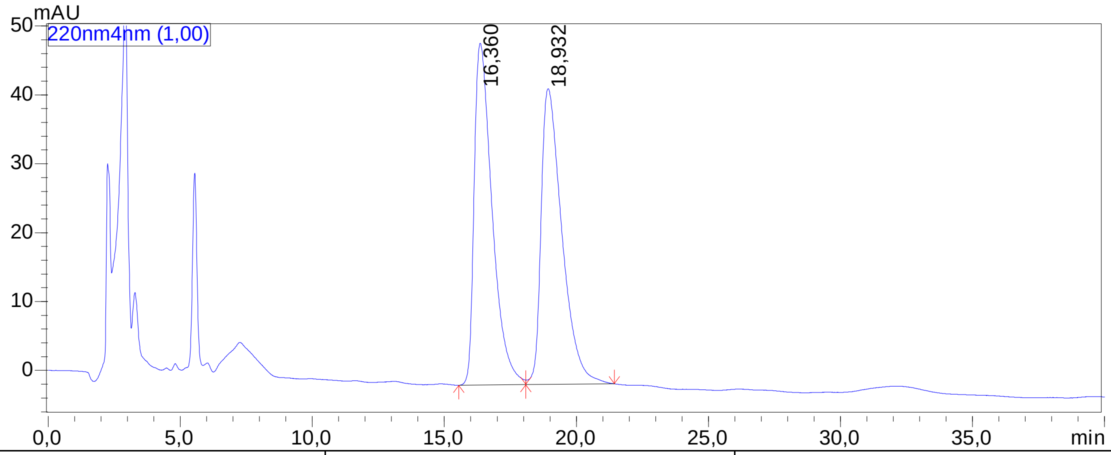

Peak#	Ret. Time	Area%
1	16.360	49.873
2	18.932	50.127
Total		100.000

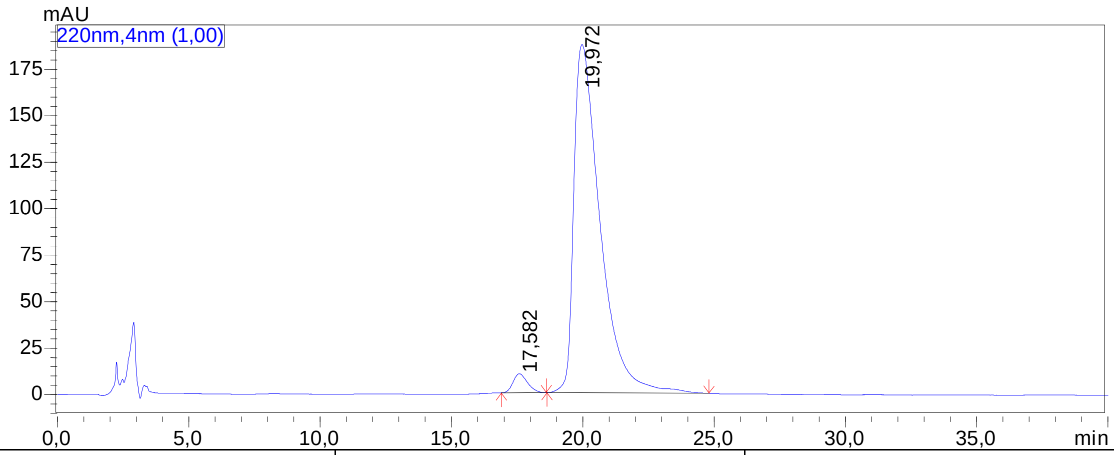

Peak#	Ret. Time	Area%
1	17.582	2.902
2	19.972	97.098
Total		100.000

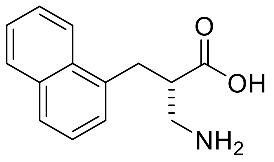

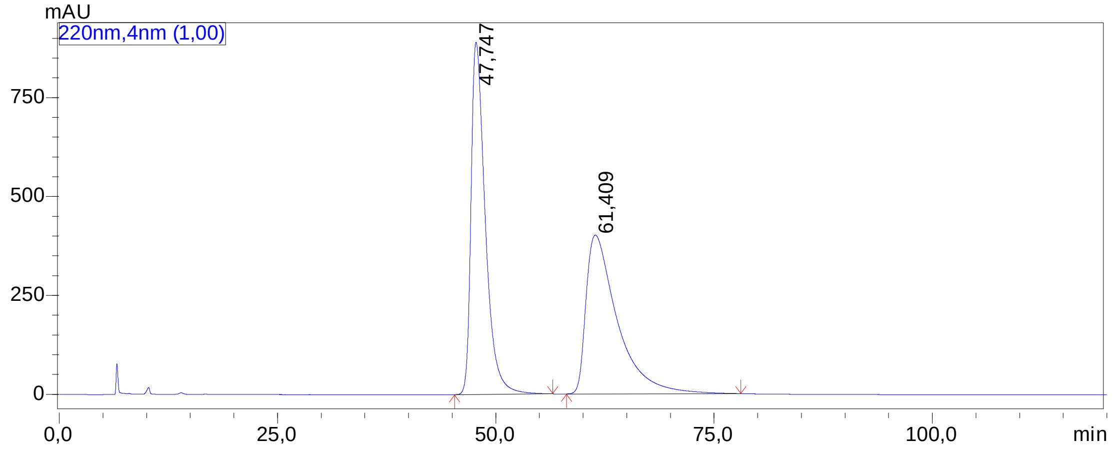

Peak#	Ret. Time	Area%
1	47.747	50.161
2	61.409	49.839
Total		100.000

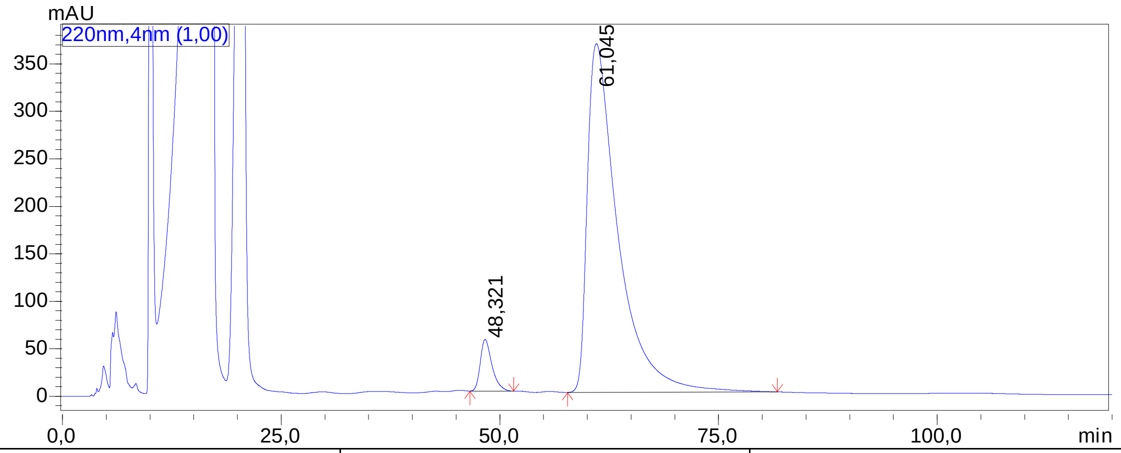

Peak#	Ret. Time	Area%
1	48.321	5.046
2	61.045	94.954
Total		100.000

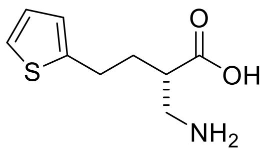

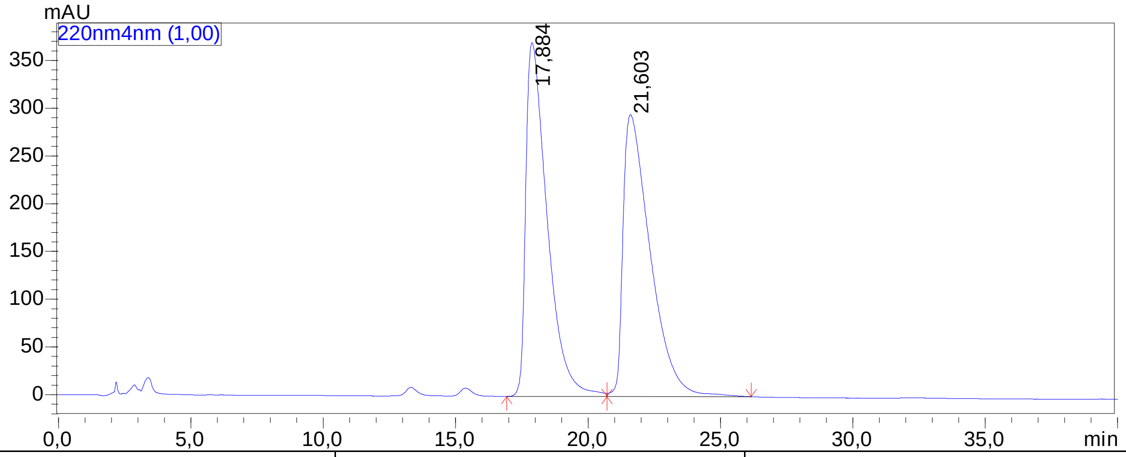

Peak#	Ret. Time	Area%
1	17.884	49.742
2	21.603	50.258
Total		100.000

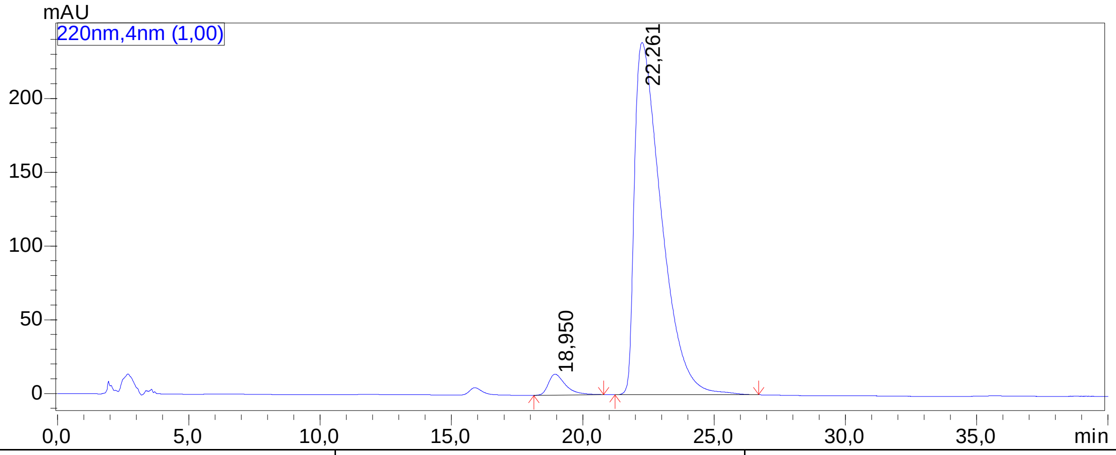

Peak#	Ret. Time	Area%
1	18.950	3.663
2	22.261	96.337
Total		100.000

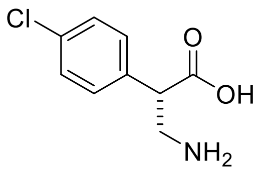

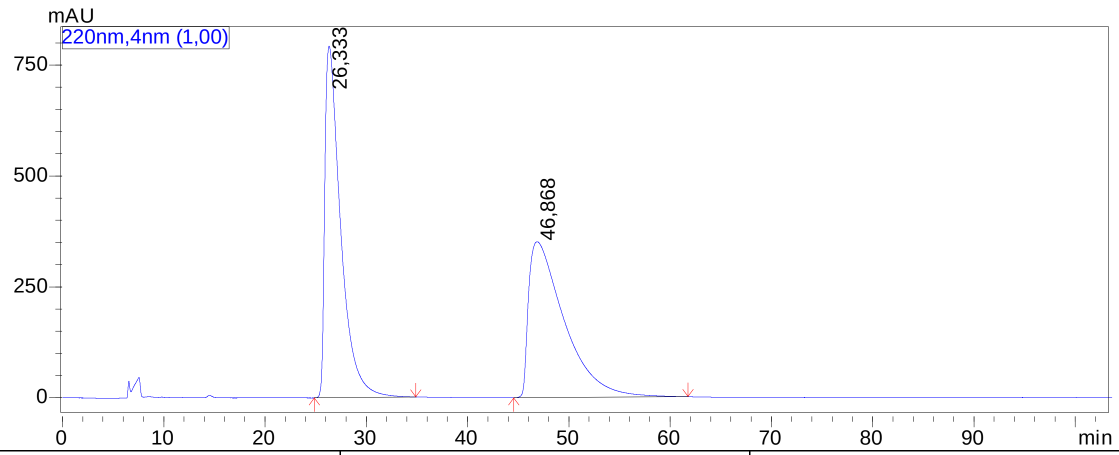

Peak#	Ret. Time	Area%
1	26.333	50.034
2	46.868	49.966
Total		100.000

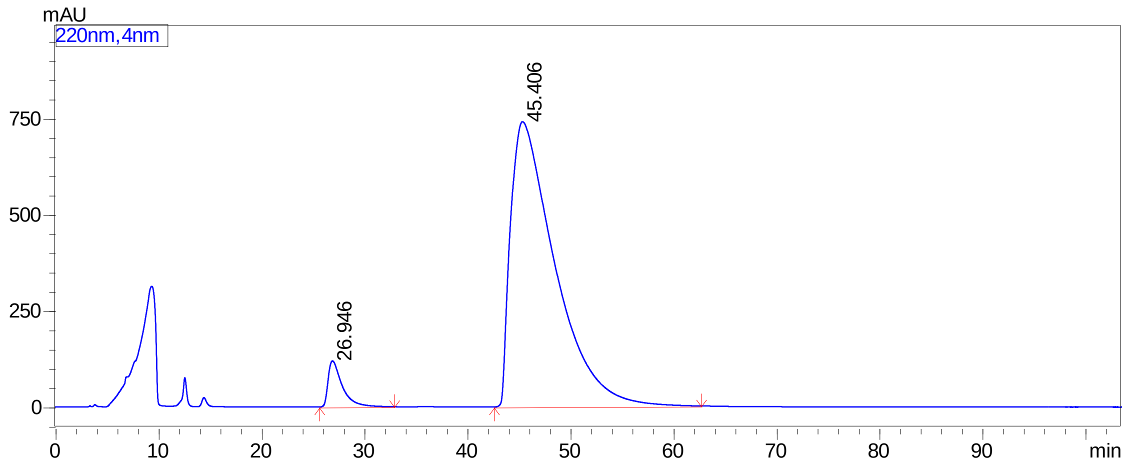

Peak#	Ret. Time	Area%
1	26.946	4.865
2	45.406	95.135
Total		100.000

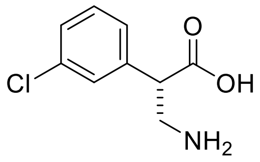

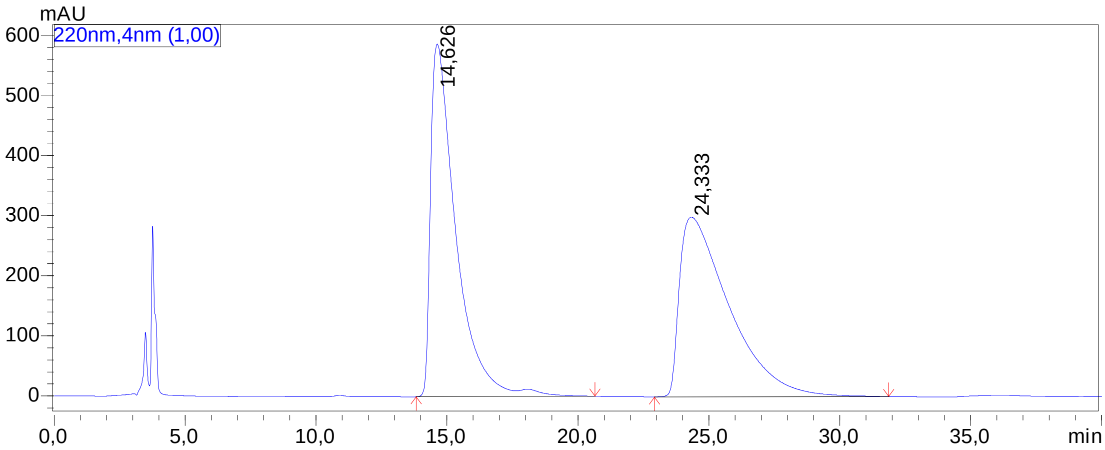

Peak#	Ret. Time	Area%
1	14.626	50.041
2	24.333	49.959
Total		100.000

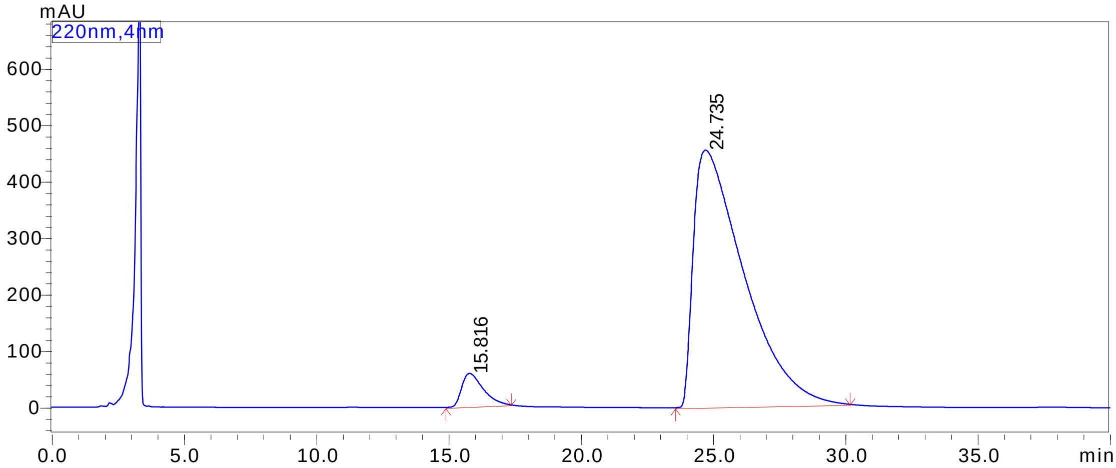

Peak#	Ret. Time	Area%
1	15.816	5.098
2	24.735	94.902
Total		100.000

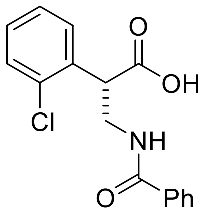

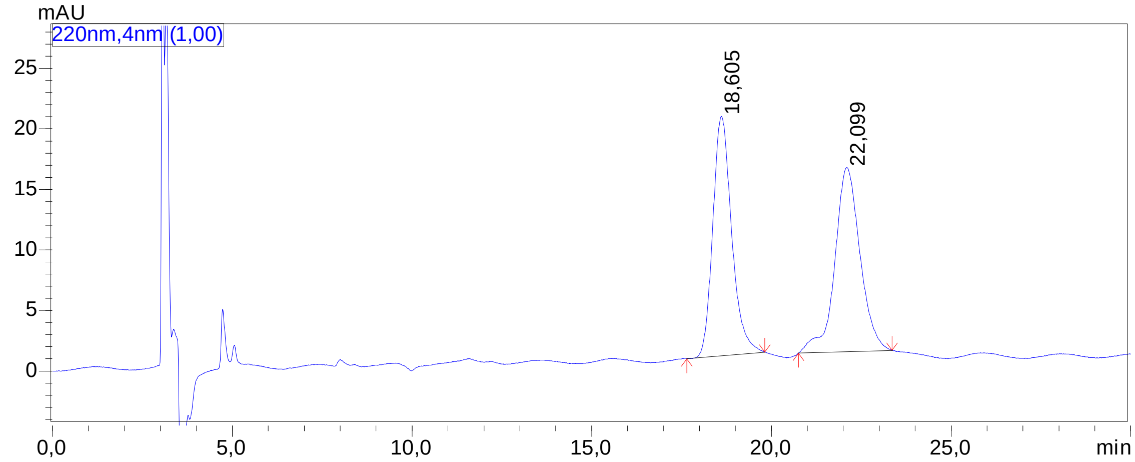

Peak#	Ret. Time	Area%
1	18.605	49.240
2	22.099	50.760
Total		100.000

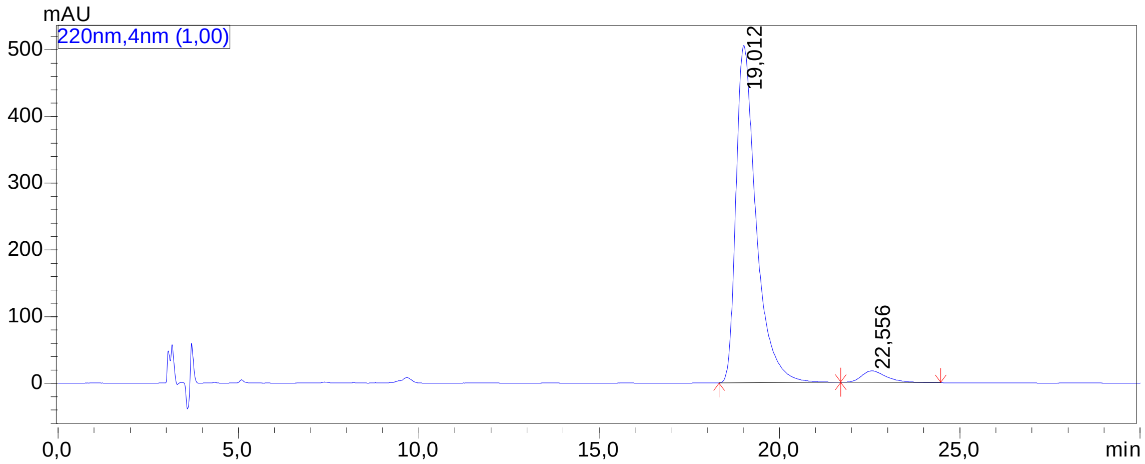

Peak#	Ret. Time	Area%
1	19.012	95.966
2	22.556	4.034
Total		100.000

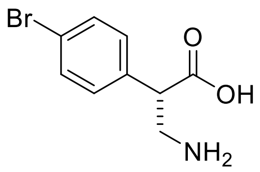

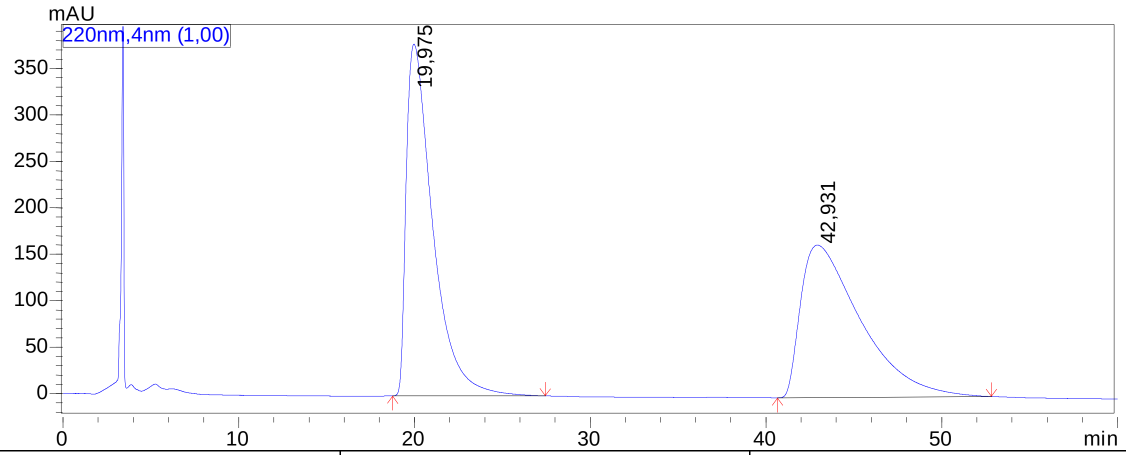

Peak#	Ret. Time	Area%
1	19.975	50.482
2	42.931	49.518
Total		100.000

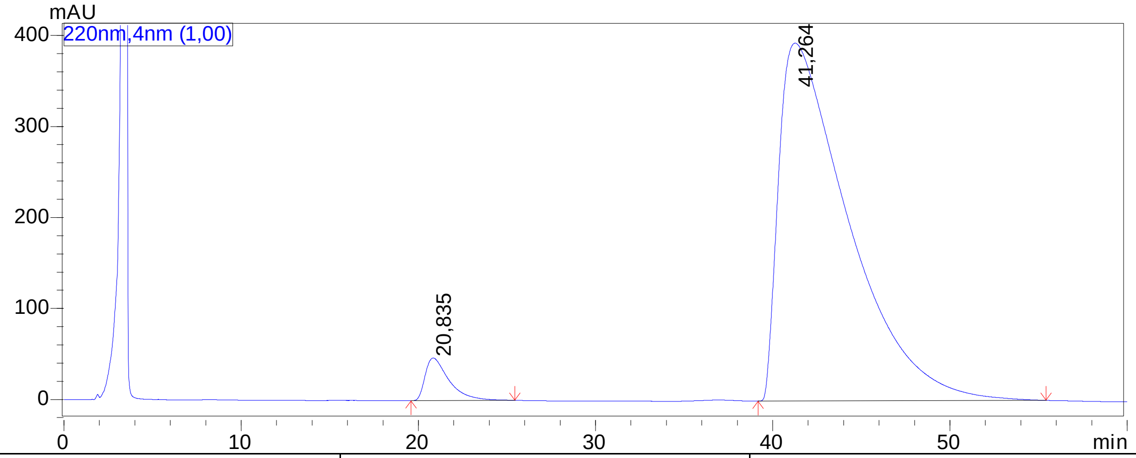

Peak#	Ret. Time	Area%
1	20.835	3.916
2	41.264	96.084
Total		100.000

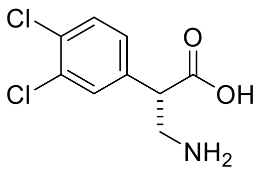

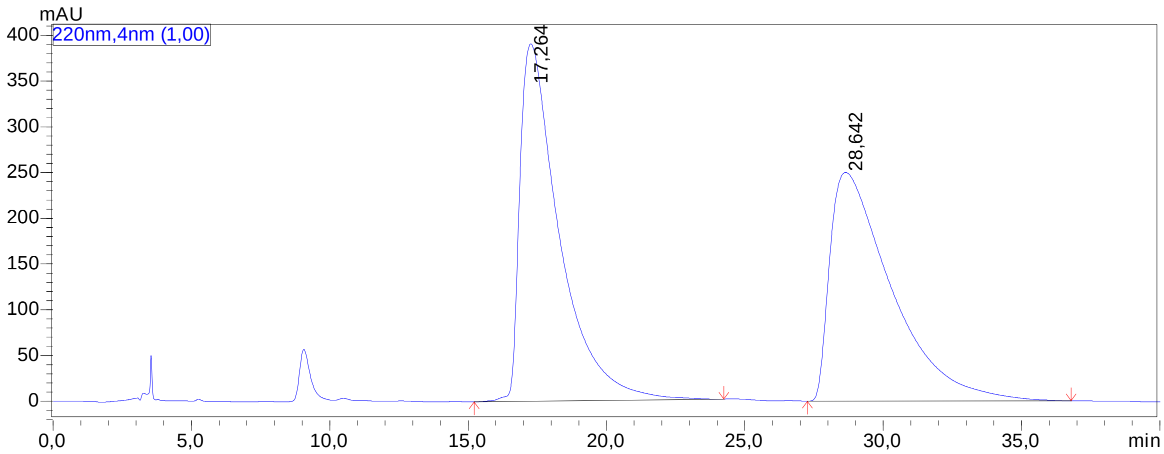

Peak#	Ret. Time	Area%
1	17.264	49.999
2	28.642	50.001
Total		100.000

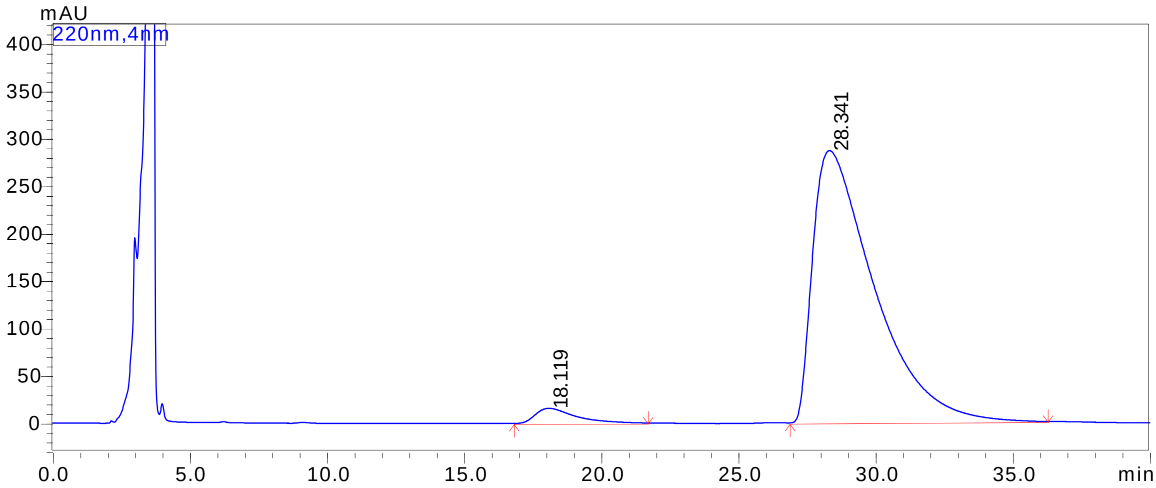

Peak#	Ret. Time	Area%
1	18.119	3.414
2	28.341	96.586
Total		100.000

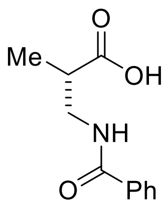

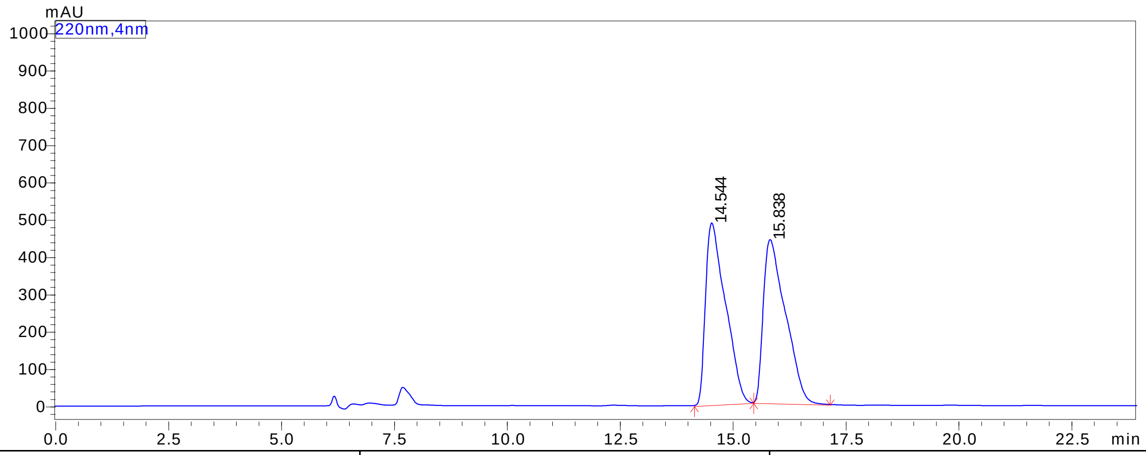

Peak#	Ret. Time	Area%
1	14.544	49.975
2	15.838	50.025
Total		100.000

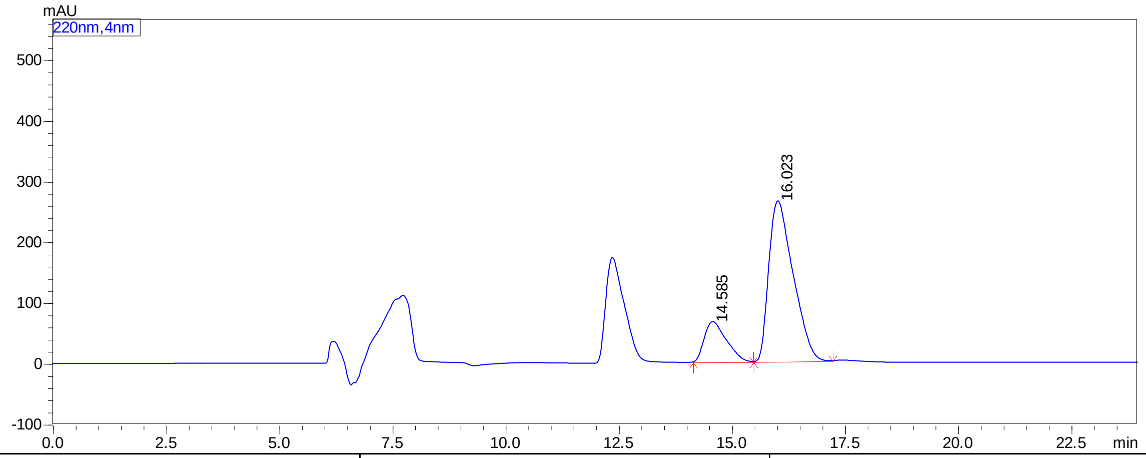

Peak#	Ret. Time	Area%
1	14.585	18.495
2	16.023	81.505
Total		100.000

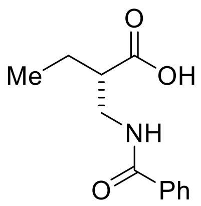

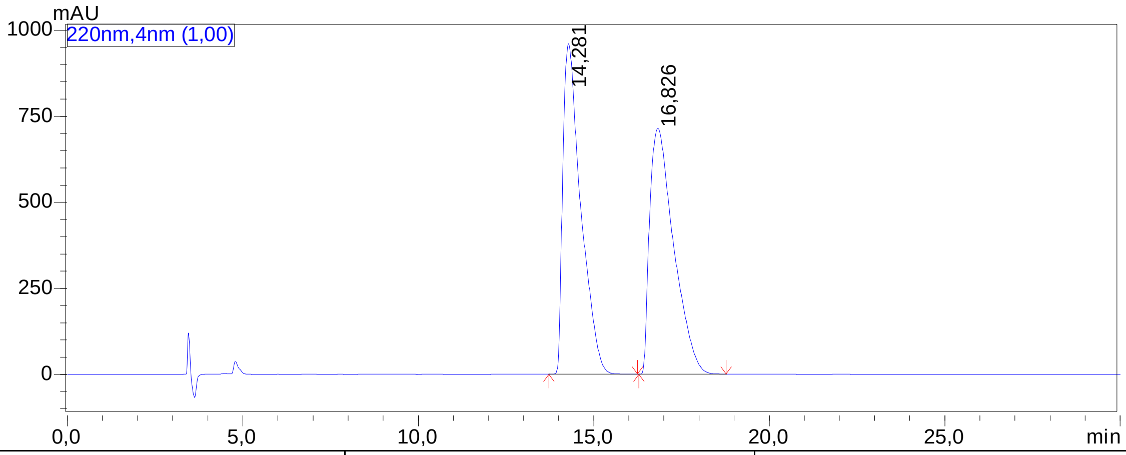

Peak#	Ret. Time	Area%
1	14.281	50.144
2	16.826	49.856
Total		100.000

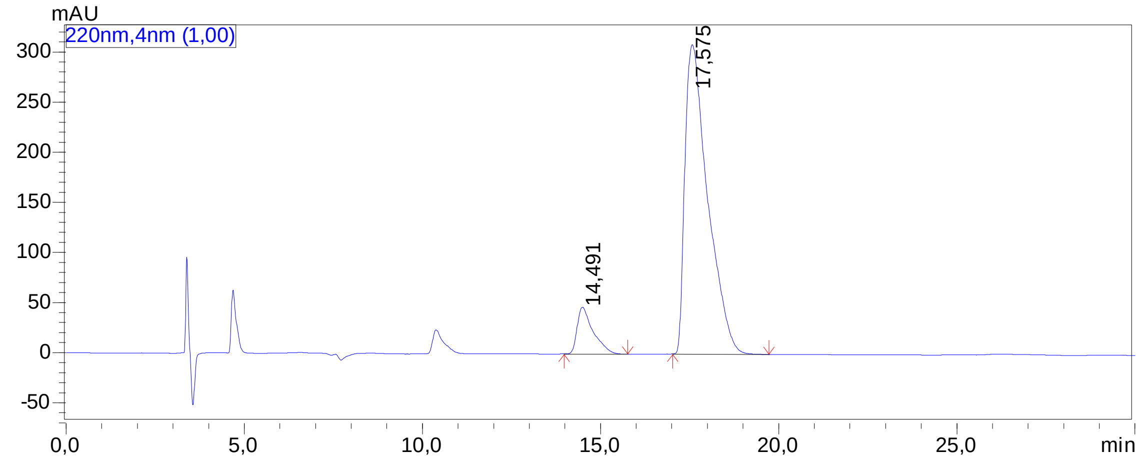

Peak#	Ret. Time	Area%
1	14.491	9.666
2	17.575	90.334
Total		100.000

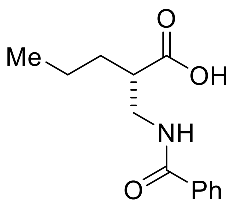

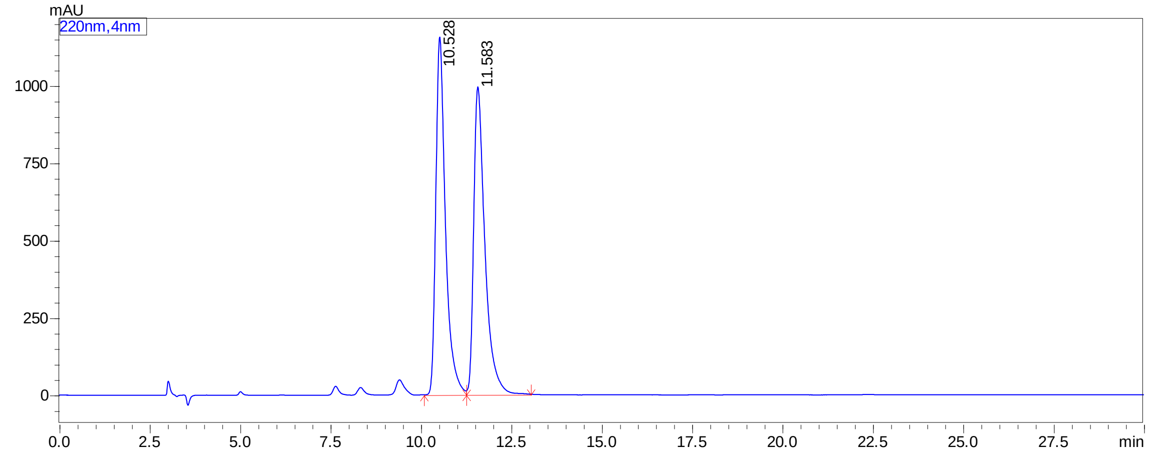

Peak#	Ret. Time	Area%
1	10.528	49.948
2	11.583	50.052
Total		100.000

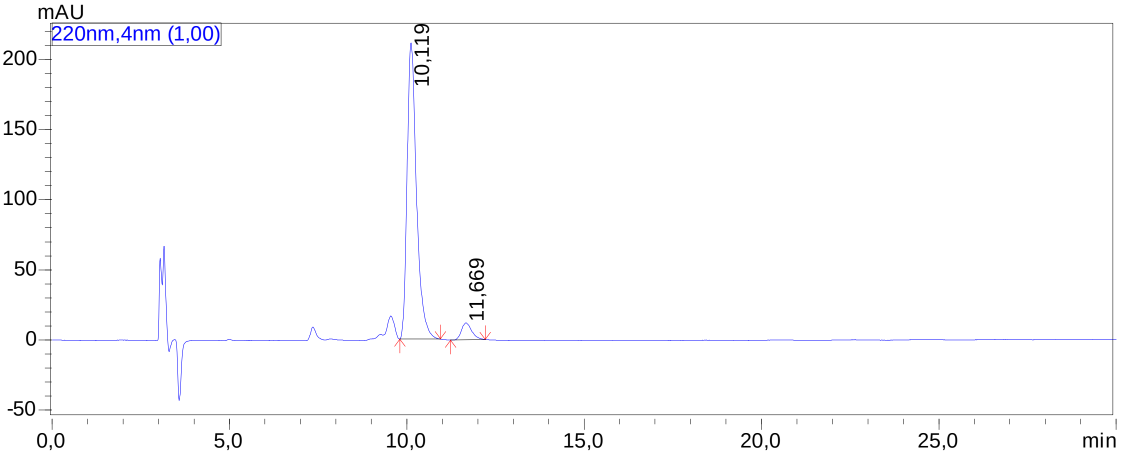

Peak#	Ret. Time	Area%
1	10.119	94.081
2	11.669	5.919
Total		100.000

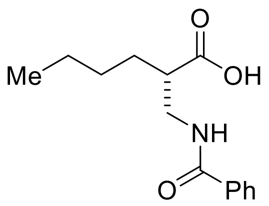

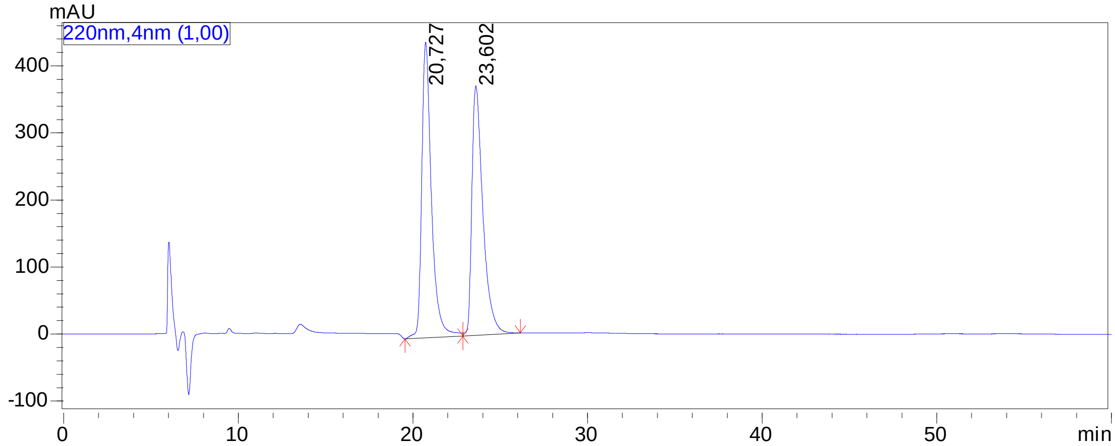

Peak#	Ret. Time	Area%
1	20.727	50.990
2	23.602	49.010
Total		100.000

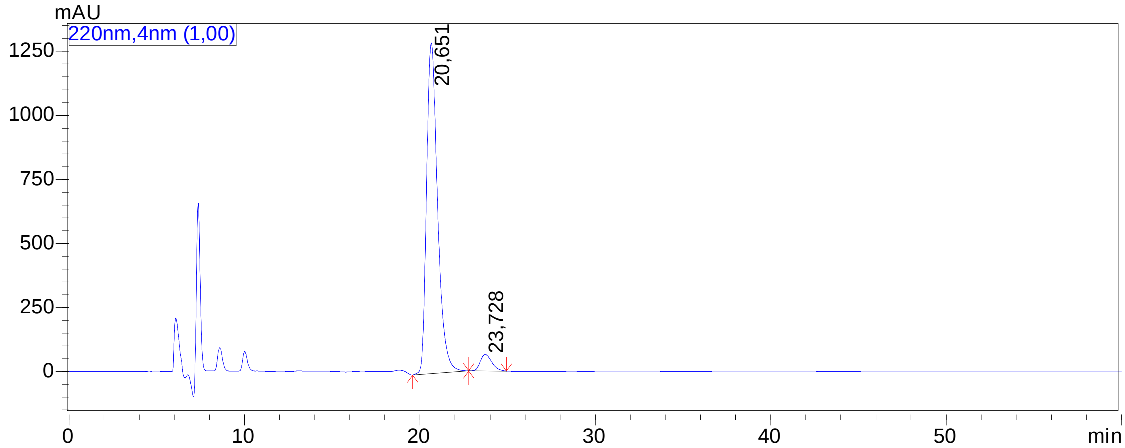

Peak#	Ret. Time	Area%
1	20.651	95.078
2	23.728	4.922
Total		100.000

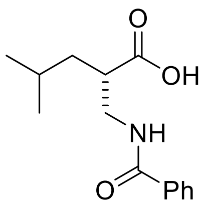

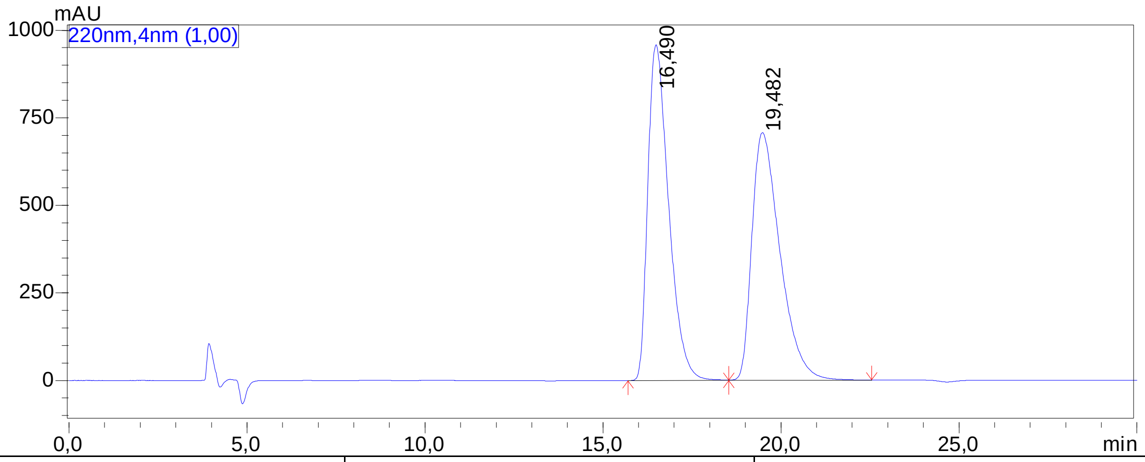

Peak#	Ret. Time	Area%
1	16.490	50.138
2	19.482	49.862
Total		100.000

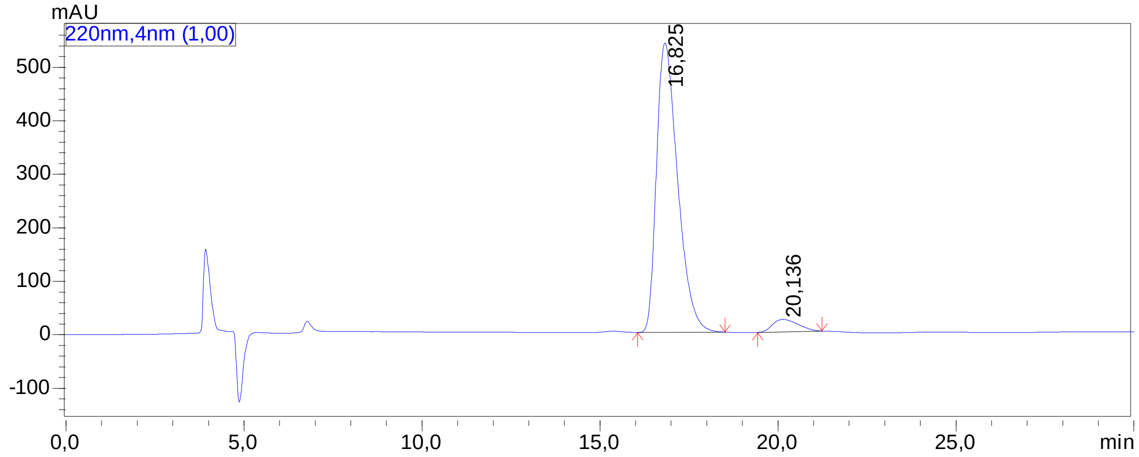

Peak#	Ret. Time	Area%
1	16.825	95.012
2	20.136	4.988
Total		100.000

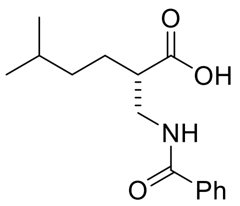

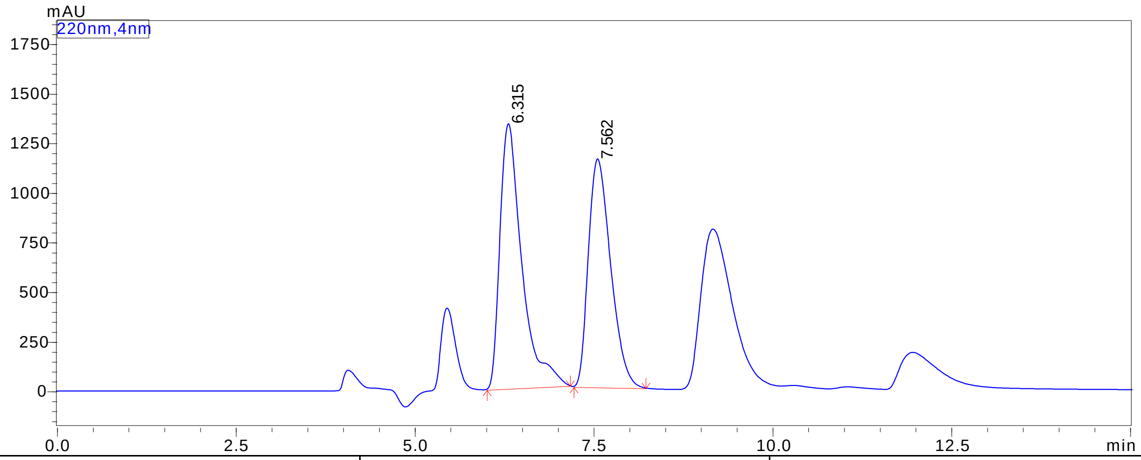

Peak#	Ret. Time	Area%
1	6.315	53.216
2	7.562	46.784
Total		100.000

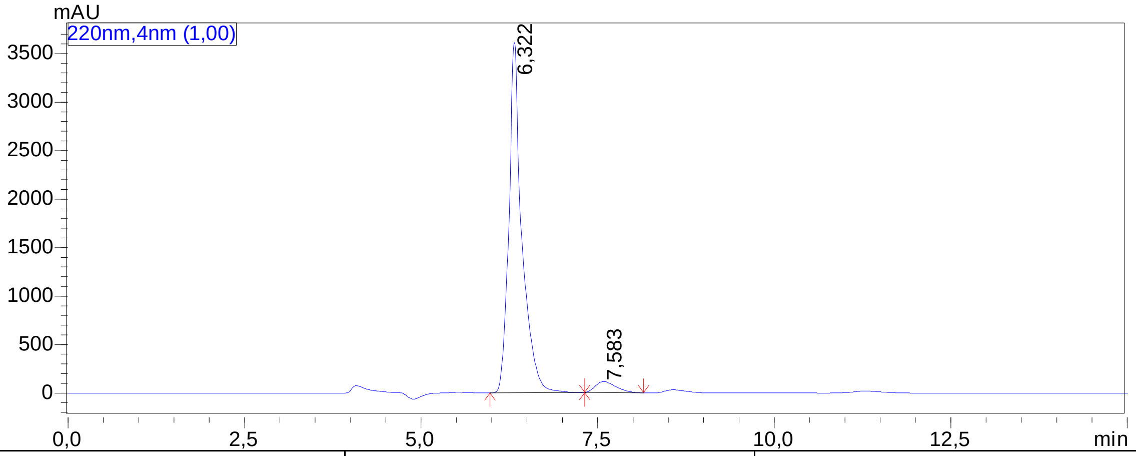

Peak#	Ret. Time	Area%
1	6.322	95.277
2	7.583	4.723
Total		100.000

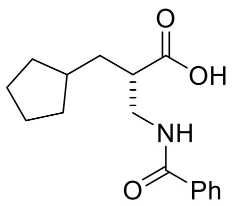

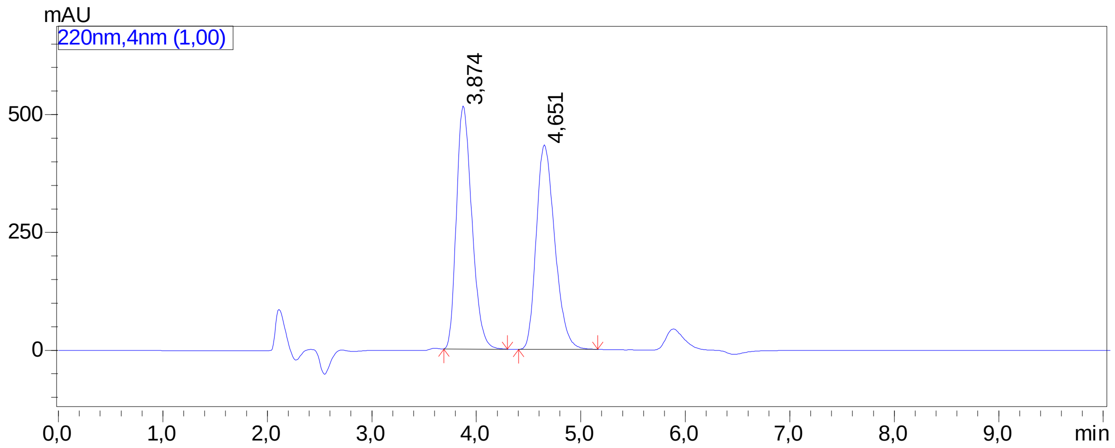

Peak#	Ret. Time	Area%
1	3.874	49.882
2	4.651	50.118
Total		100.000

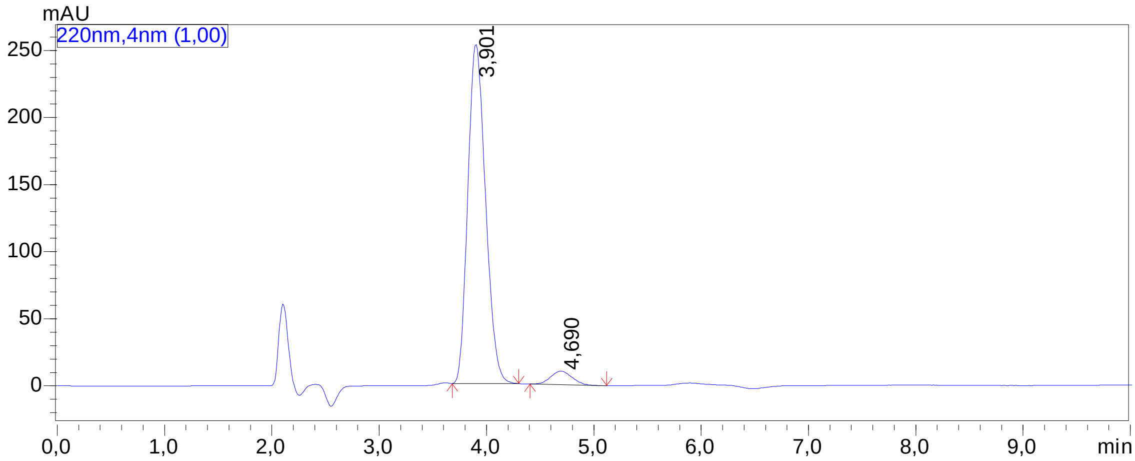

Peak#	Ret. Time	Area%
1	3.901	95.429
2	4.690	4.571
Total		100.000

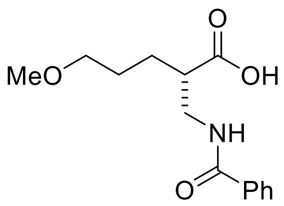

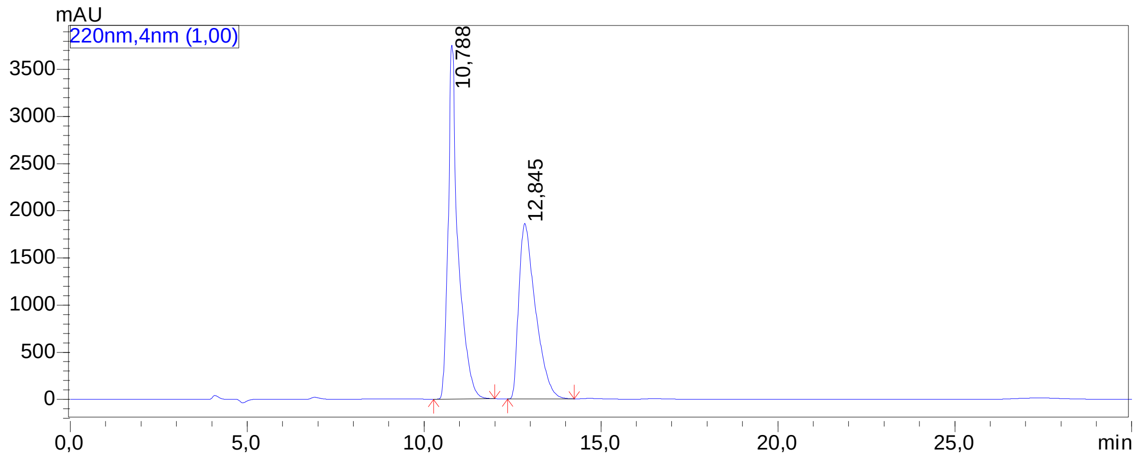

Peak#	Ret. Time	Area%
1	10.788	54.709
2	12.845	45.291
Total		100.000

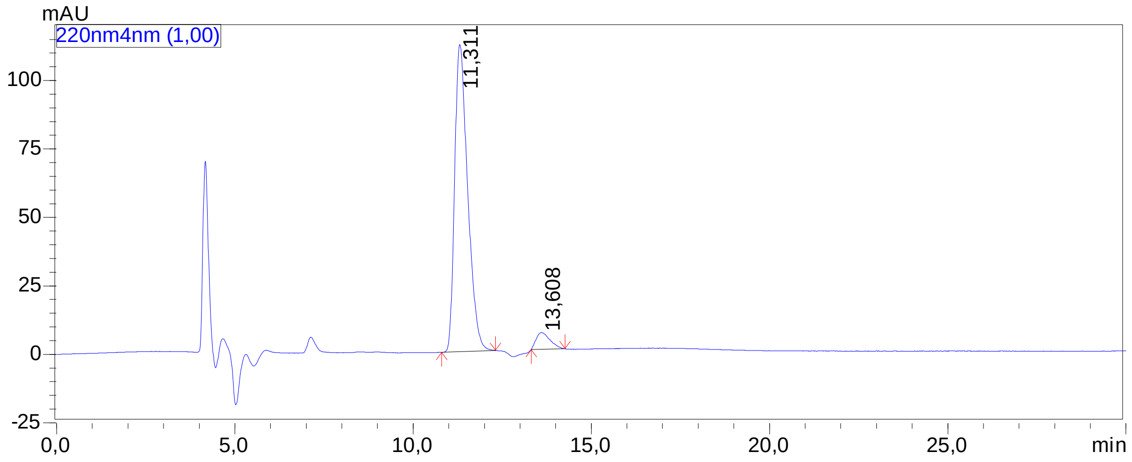

Peak#	Ret. Time	Area%
1	11.311	94.548
2	13.608	5.452
Total		100.000

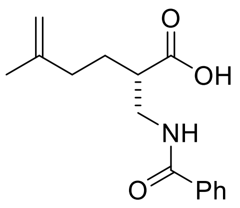

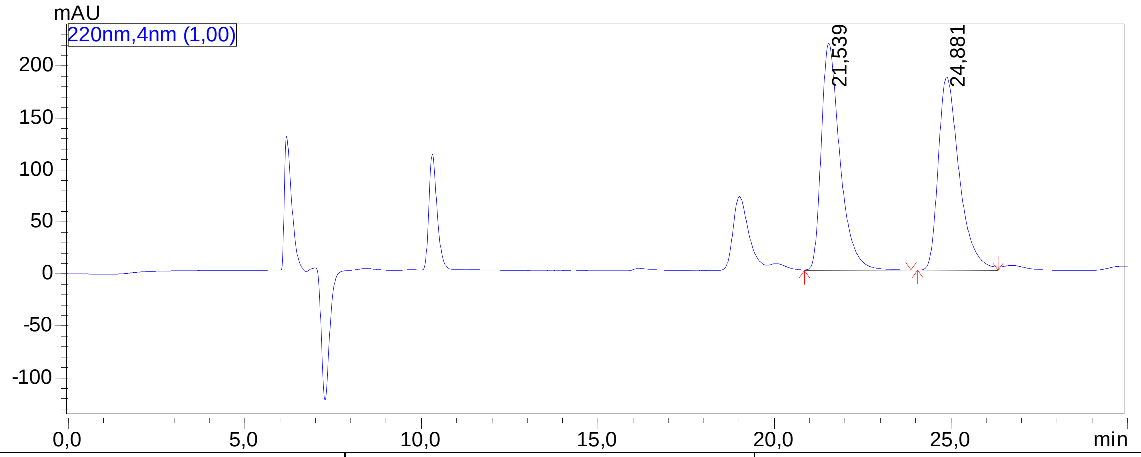

Peak#	Ret. Time	Area%
1	21.539	51.083
2	24.881	48.917
Total		100.000

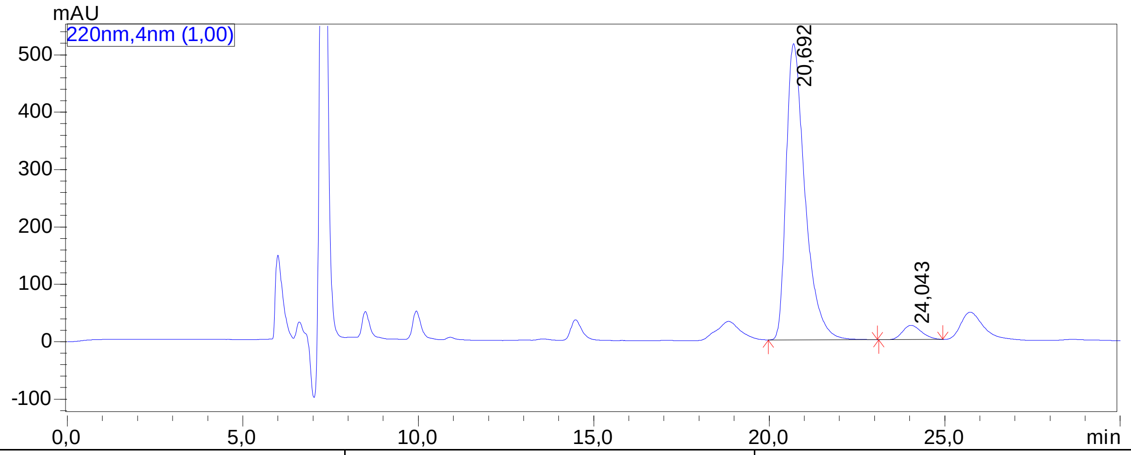

Peak#	Ret. Time	Area%
1	20.692	96.089
2	24.043	3.911
Total		100.000

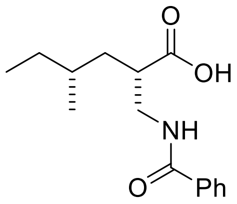

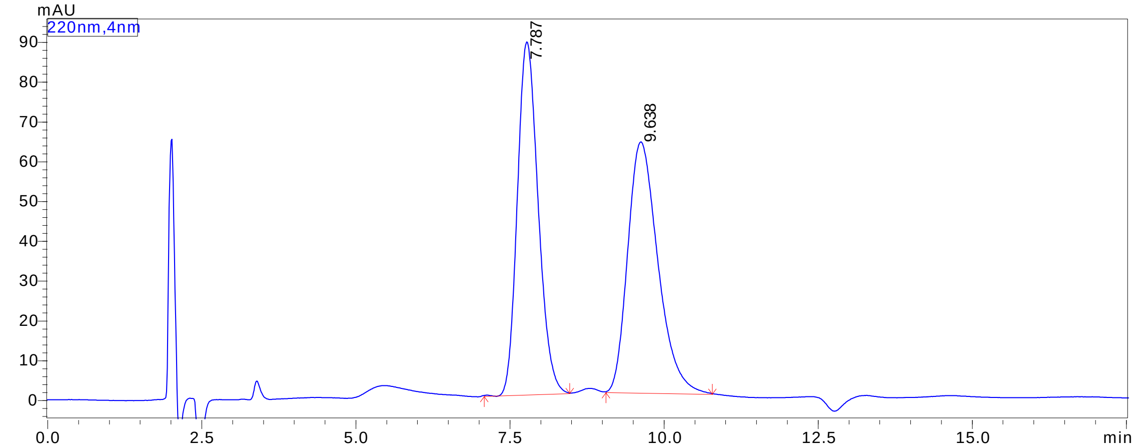

Peak#	Ret. Time	Area%
1	7.787	49.232
2	9.638	50.768
Total		100.000

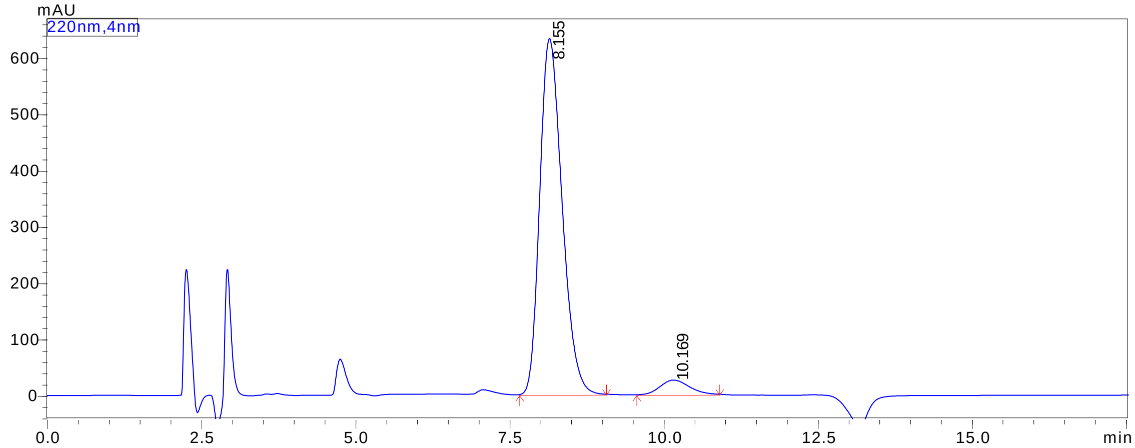

Peak#	Ret. Time	Area%
1	8.155	95.058
2	10.169	4.942
Total		100.000

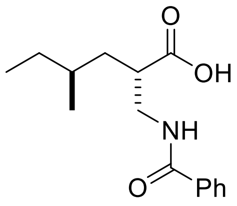

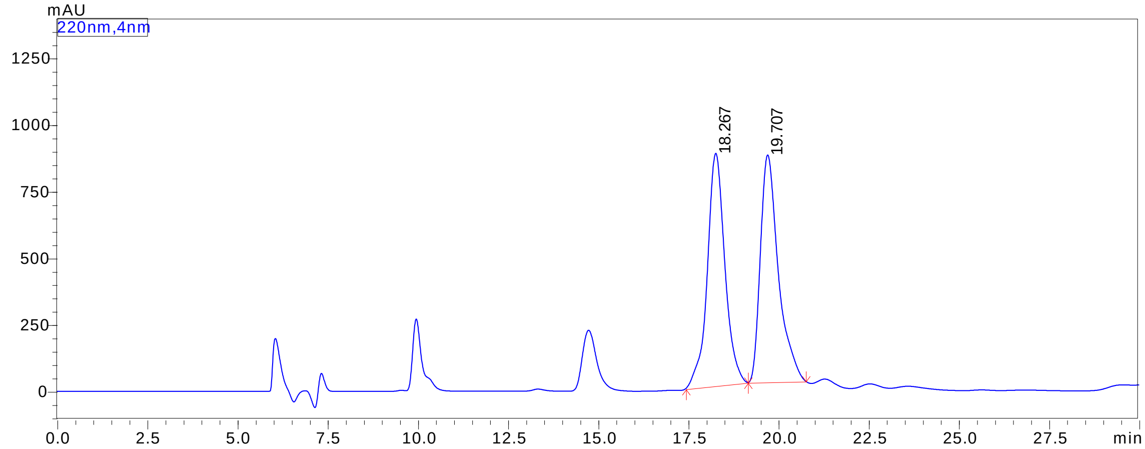

Peak#	Ret. Time	Area%
1	18.267	49.987
2	19.707	50.013
Total		100.000

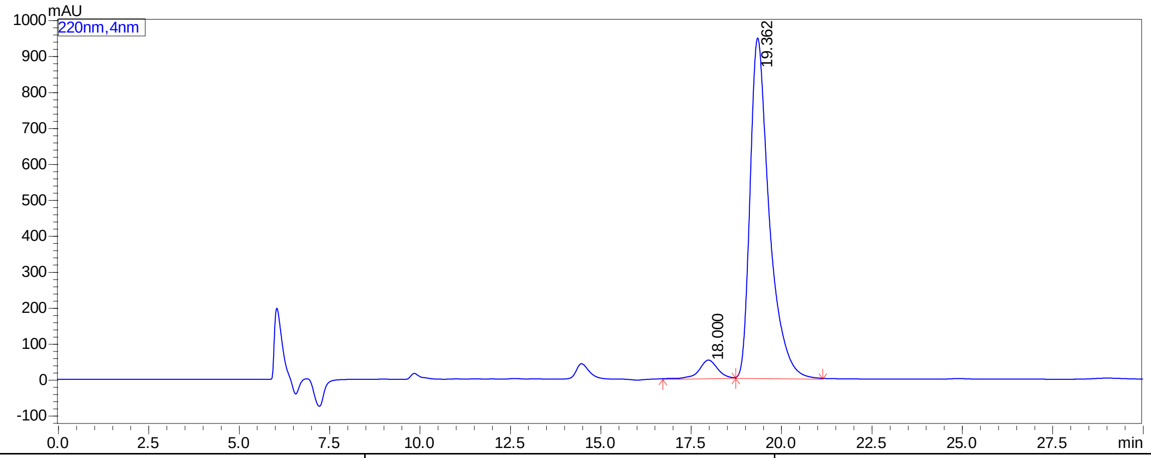

Peak#	Ret. Time	Area%
1	18.000	4.603
2	19.362	95.397
Total		100.000

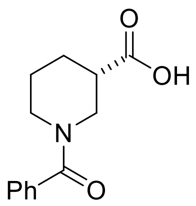

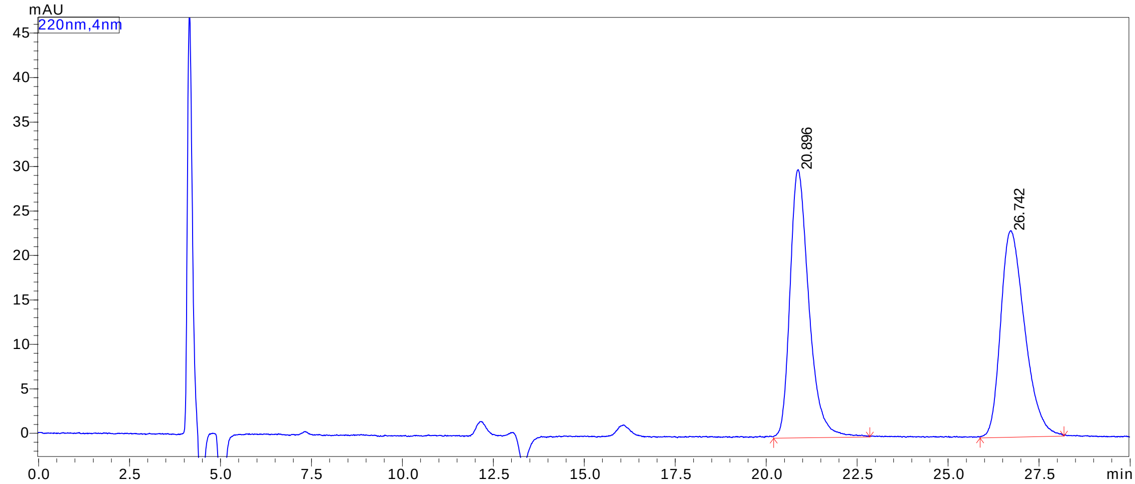

Peak#	Ret. Time	Area%
1	20.896	50.405
2	26.742	49.595
Total		100.000

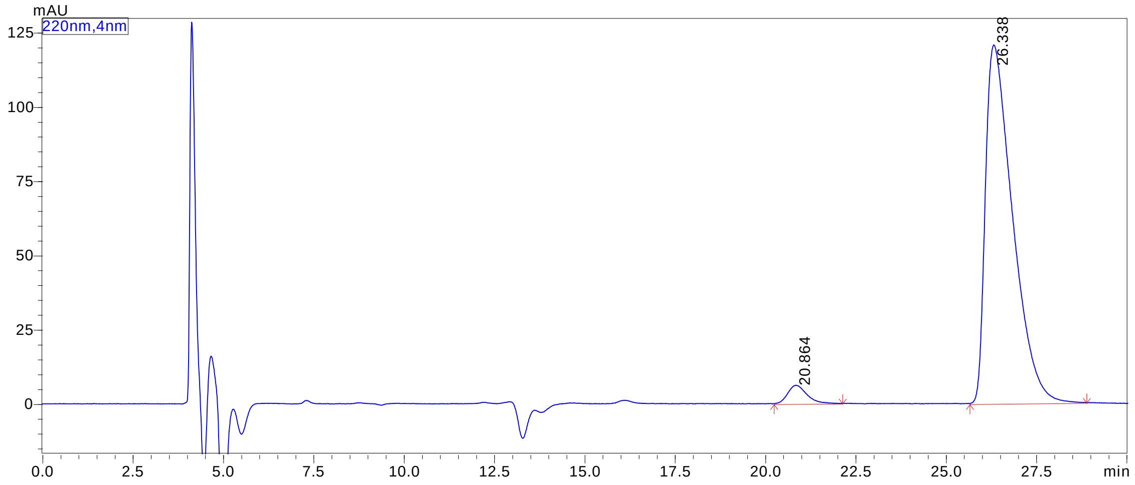

Peak#	Ret. Time	Area%
1	20.864	3.139
2	26.338	96.861
Total		100.000

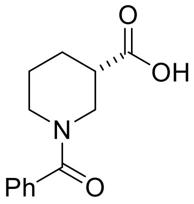

from (*S*)-(+)-3-Piperidinecarboxylic acid

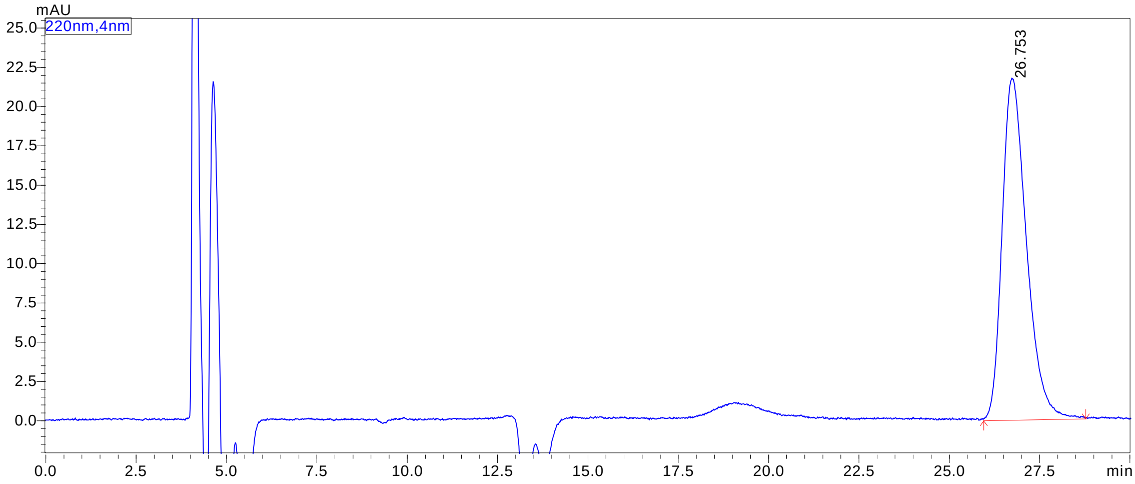

Peak#	Ret. Time	Area%
1	26.753	100.000
Total		100.000

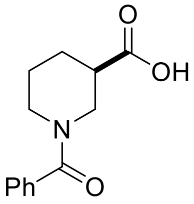

from (*R*)-(-)-3-Piperidinecarboxylic acid

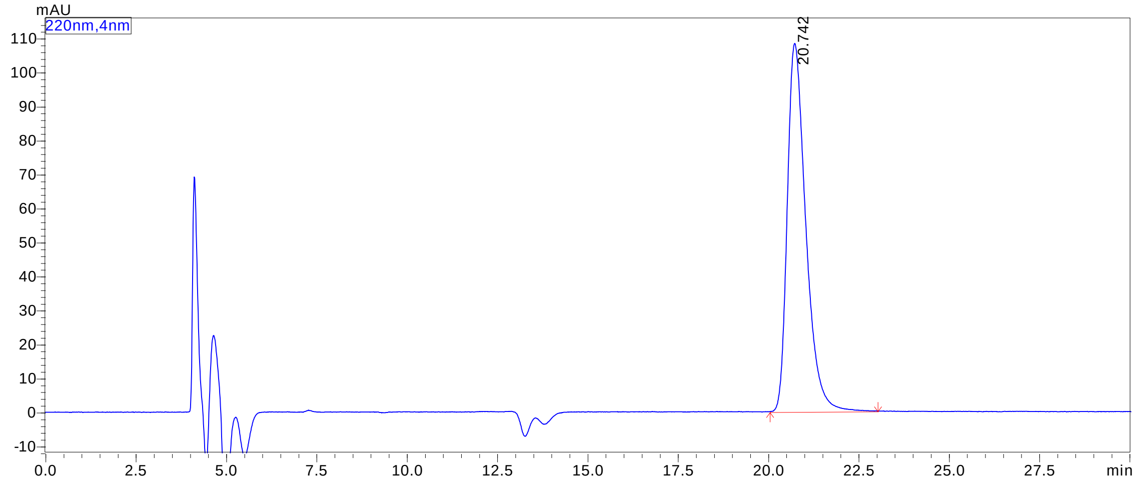

Peak#	Ret. Time	Area%
1	20.742	100.000
Total		100.000

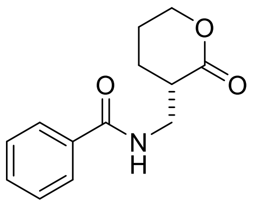

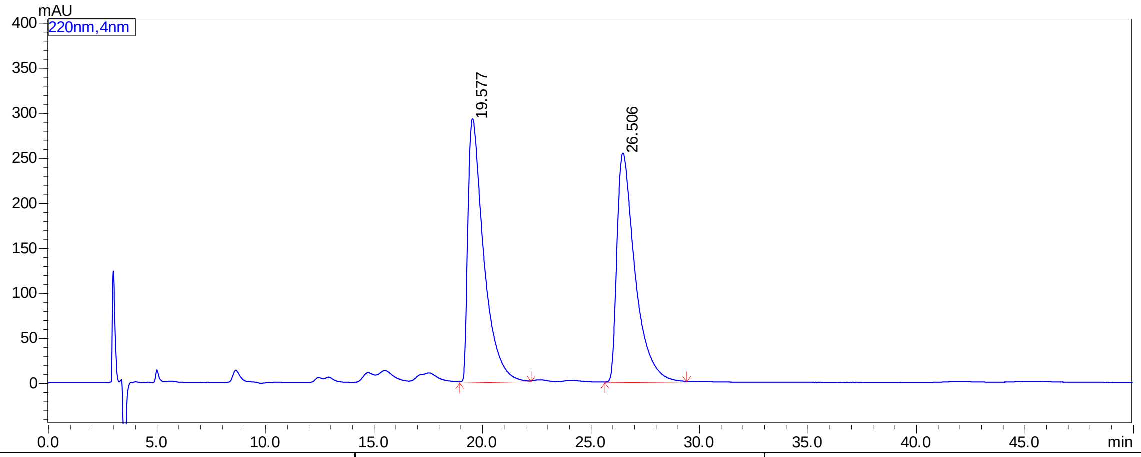

Peak#	Ret. Time	Area%
1	19.577	50.178
2	26.506	49.822
Total		100.000

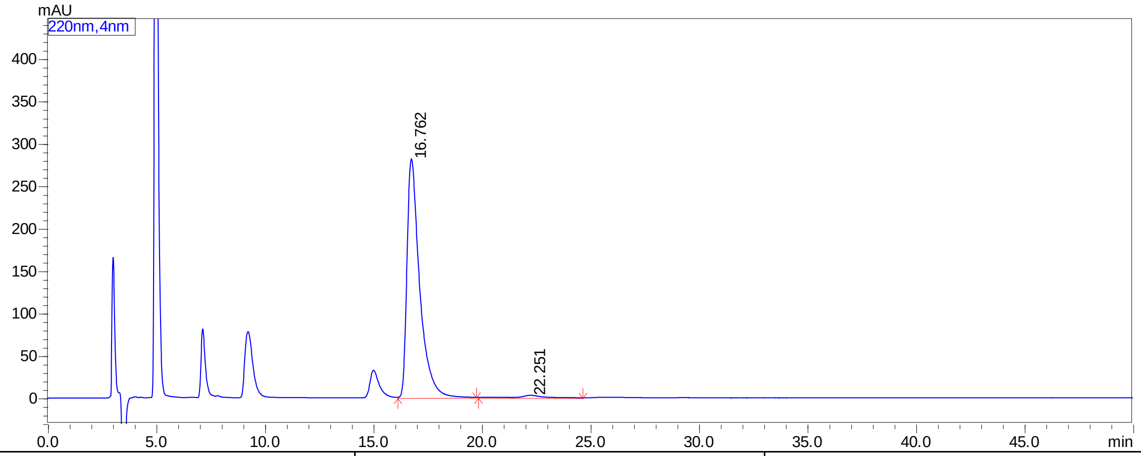

Peak#	Ret. Time	Area%
1	16.762	98.601
2	22.251	1.399
Total		100.000

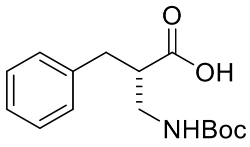

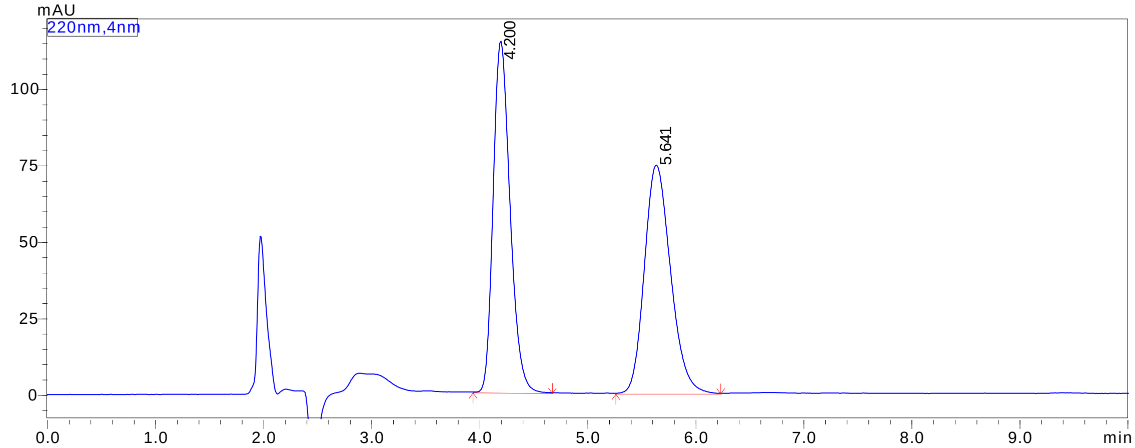

Peak#	Ret. Time	Area%
1	4.200	49.783
2	5.641	50.217
Total		100.000

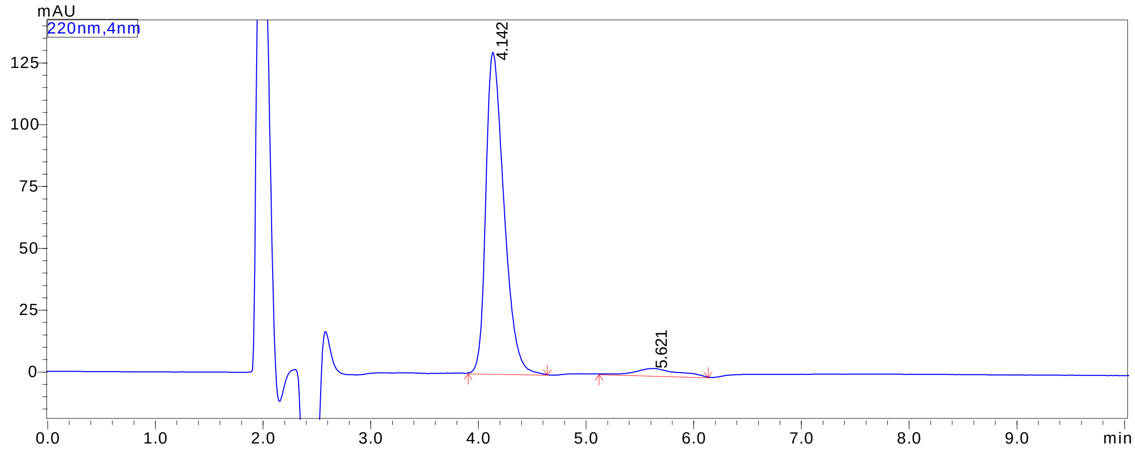

Peak#	Ret. Time	Area%
1	4.142	95.856
2	5.621	4.144
Total		100.000

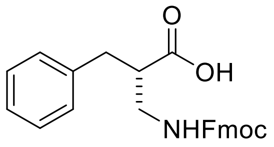

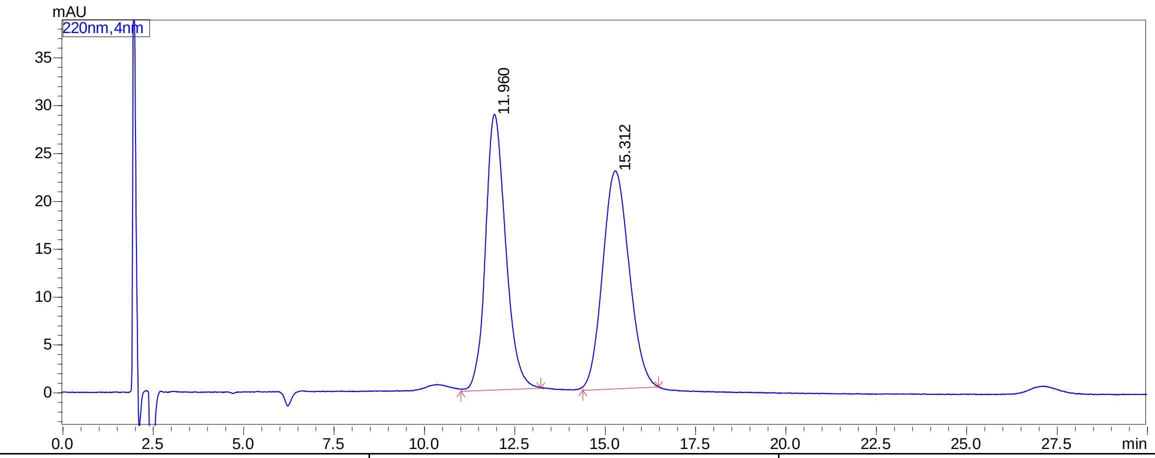

Peak#	Ret. Time	Area%
1	11.960	49.019
2	15.312	50.981
Total		100.000

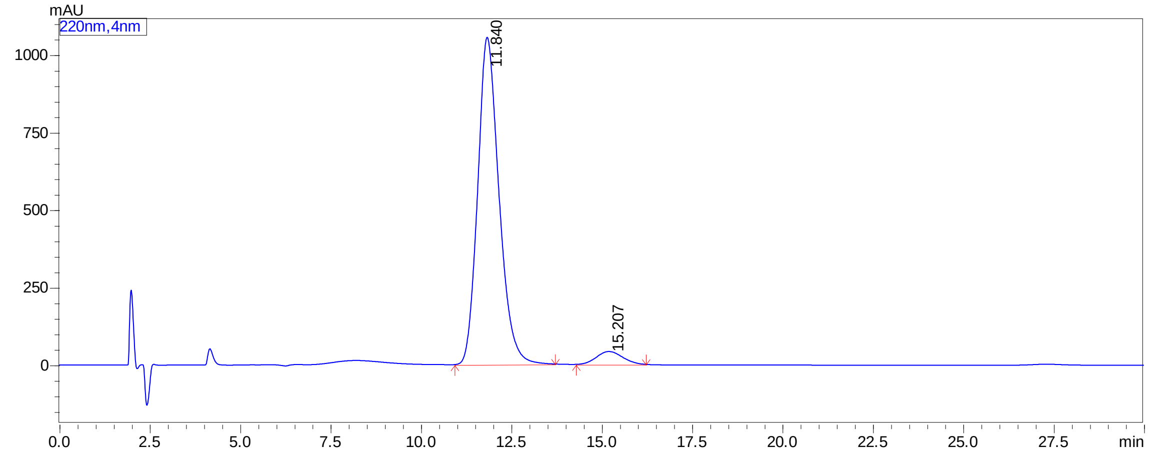

Peak#	Ret. Time	Area%
1	11.840	95.444
2	15.207	4.556
Total		100.000

HPLC Traces for Computational Studies (see a detailed procedure in the paragraph of *Procedure for Reaction Development*)

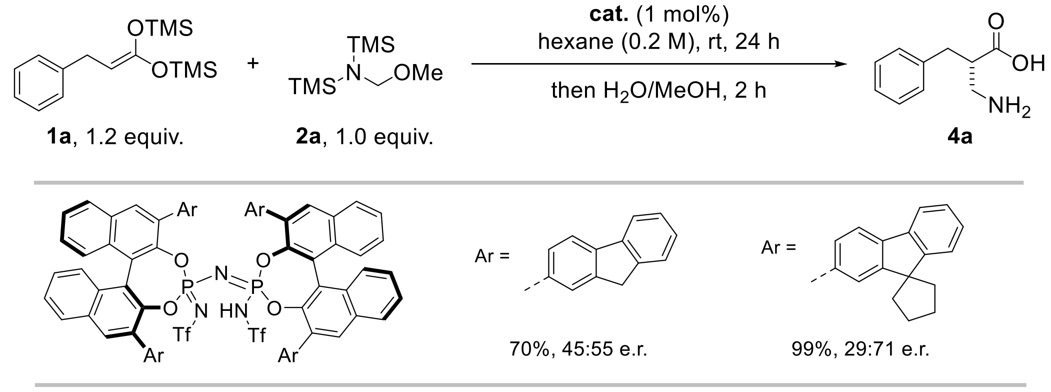

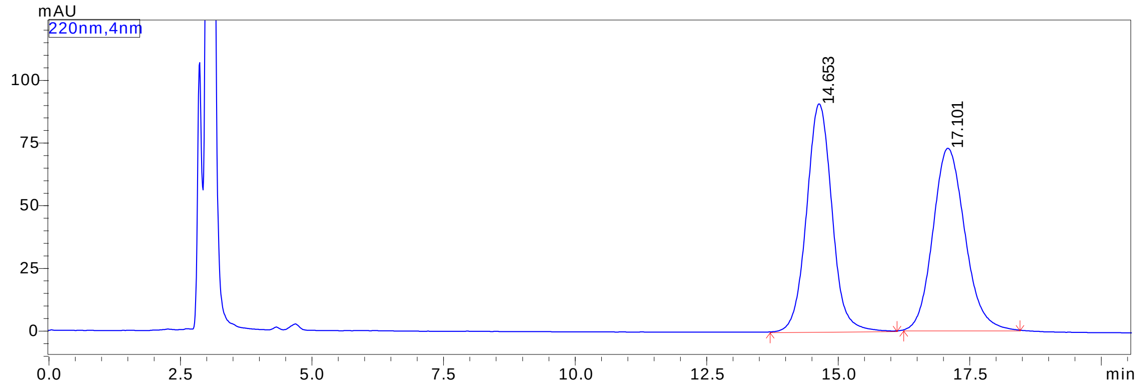

Peak#	Ret. Time	Area%
1	14.653	49.855
2	17.101	50.145
Total		100.000

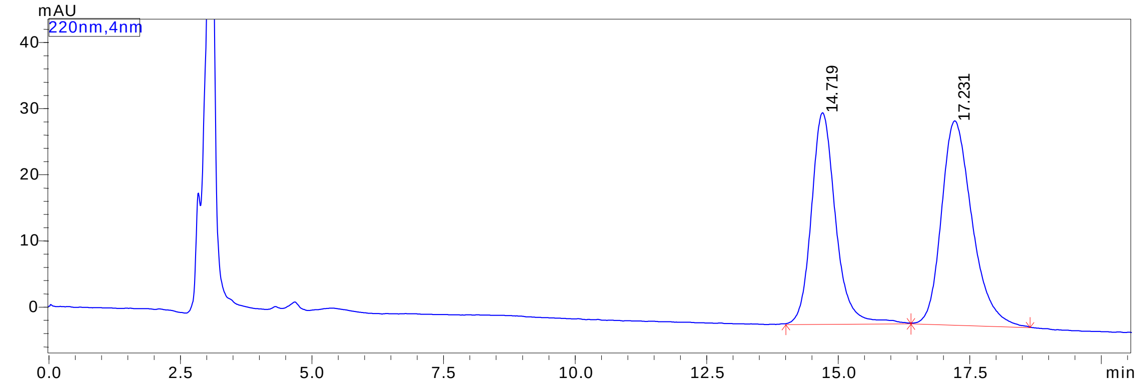

Peak#	Ret. Time	Area%
1	14.719	44.965
2	17.231	55.035
Total		100.000

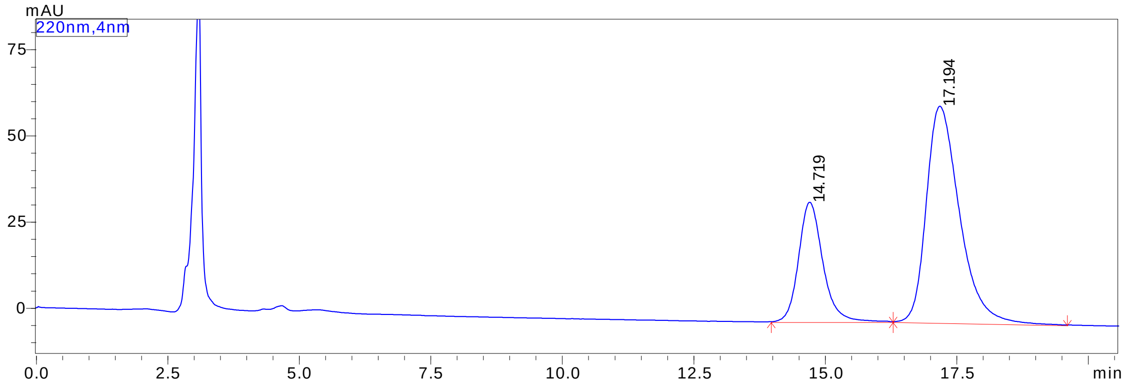

Peak#	Ret. Time	Area%
1	14.719	28.647
2	17.194	71.353
Total		100.000

10. ^{1}H , ^{13}C, ^{31}P and ^{19}F NMR spectra of substrates and products

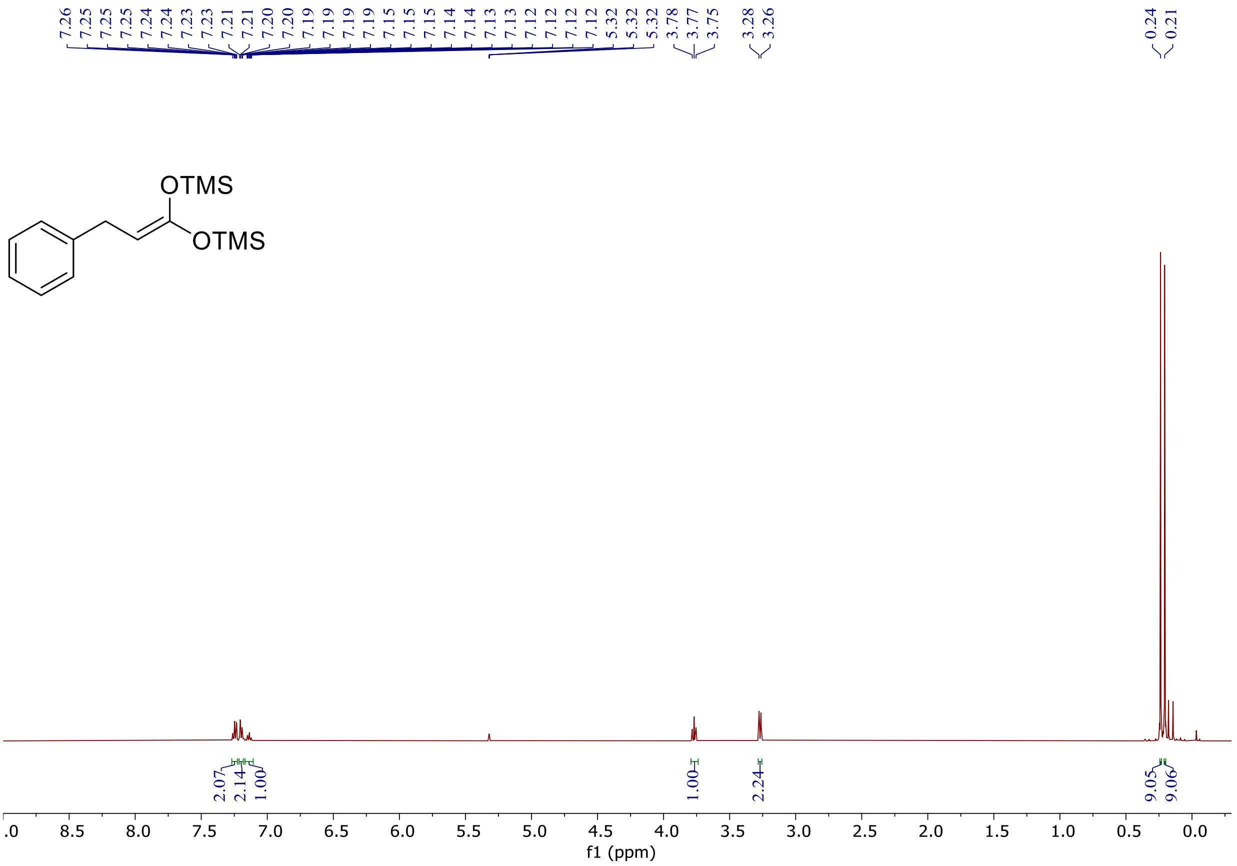

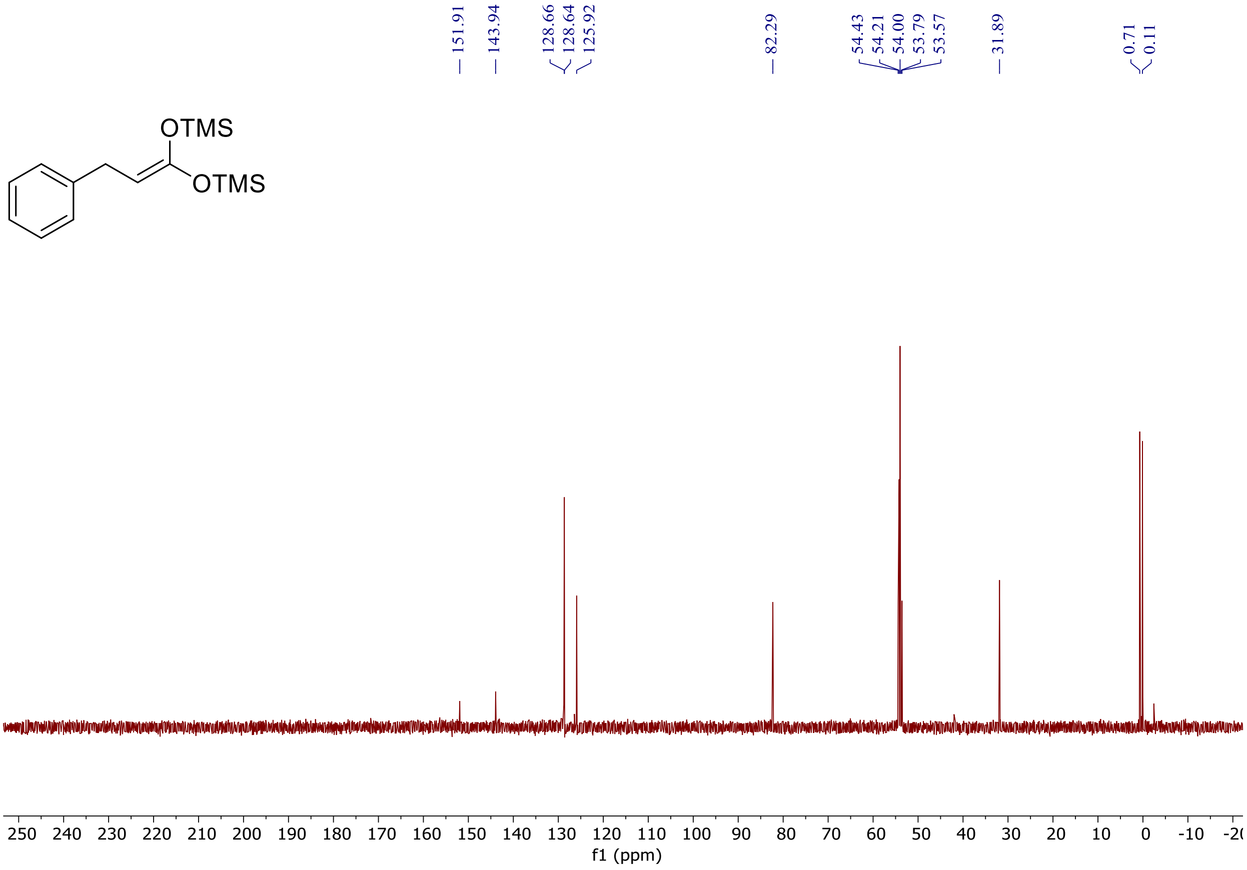

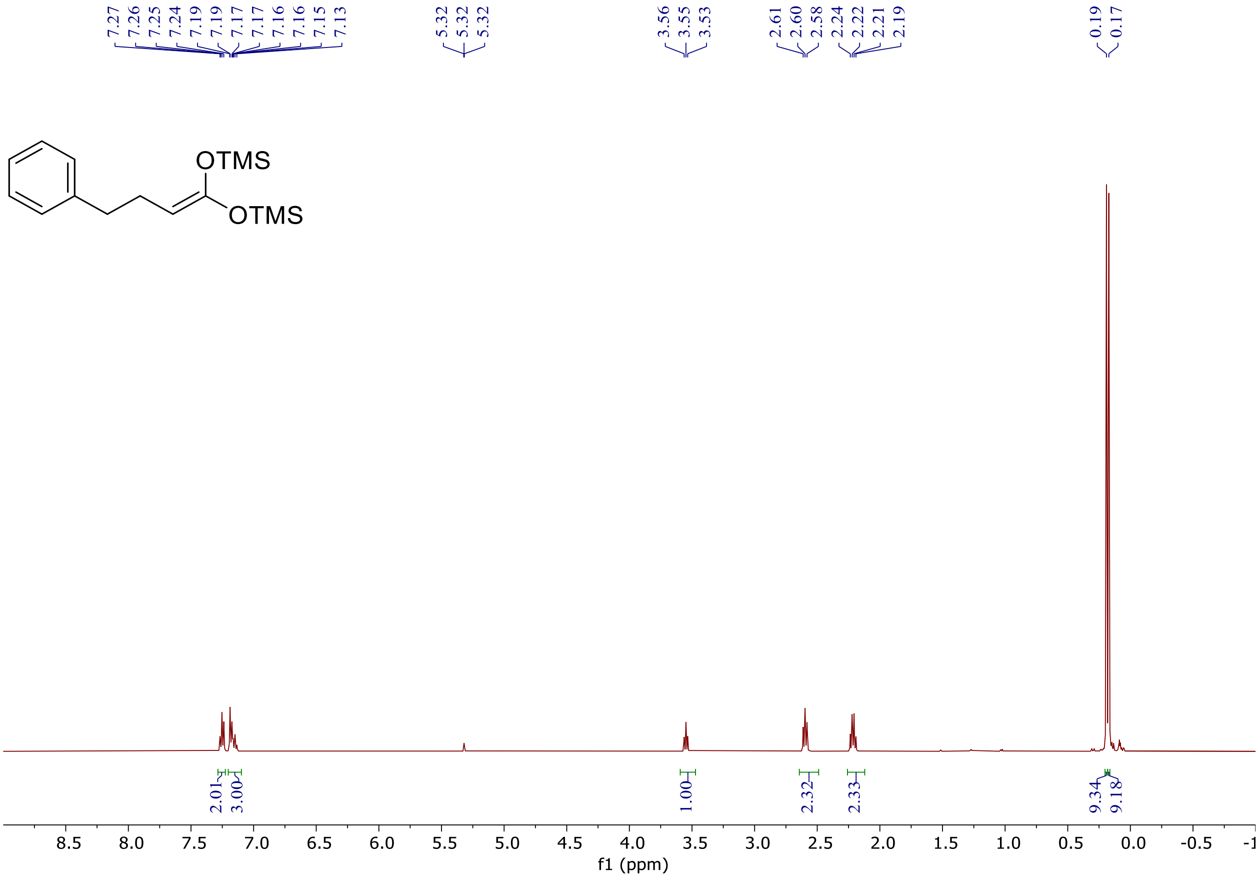
7.27
7.26
7.25
7.24
7.19
7.19
7.17
7.17
7.16
7.16
7.15
7.13
5.32
5.32
5.32
3.56
3.55
3.53
2.61
2.60
2.58
2.24
2.22
2.21
2.19
0.19
0.17
OTMS
OTMS
2.01
3.00
1.00
2.32
2.33
9.34
9.18
f1 (ppm)

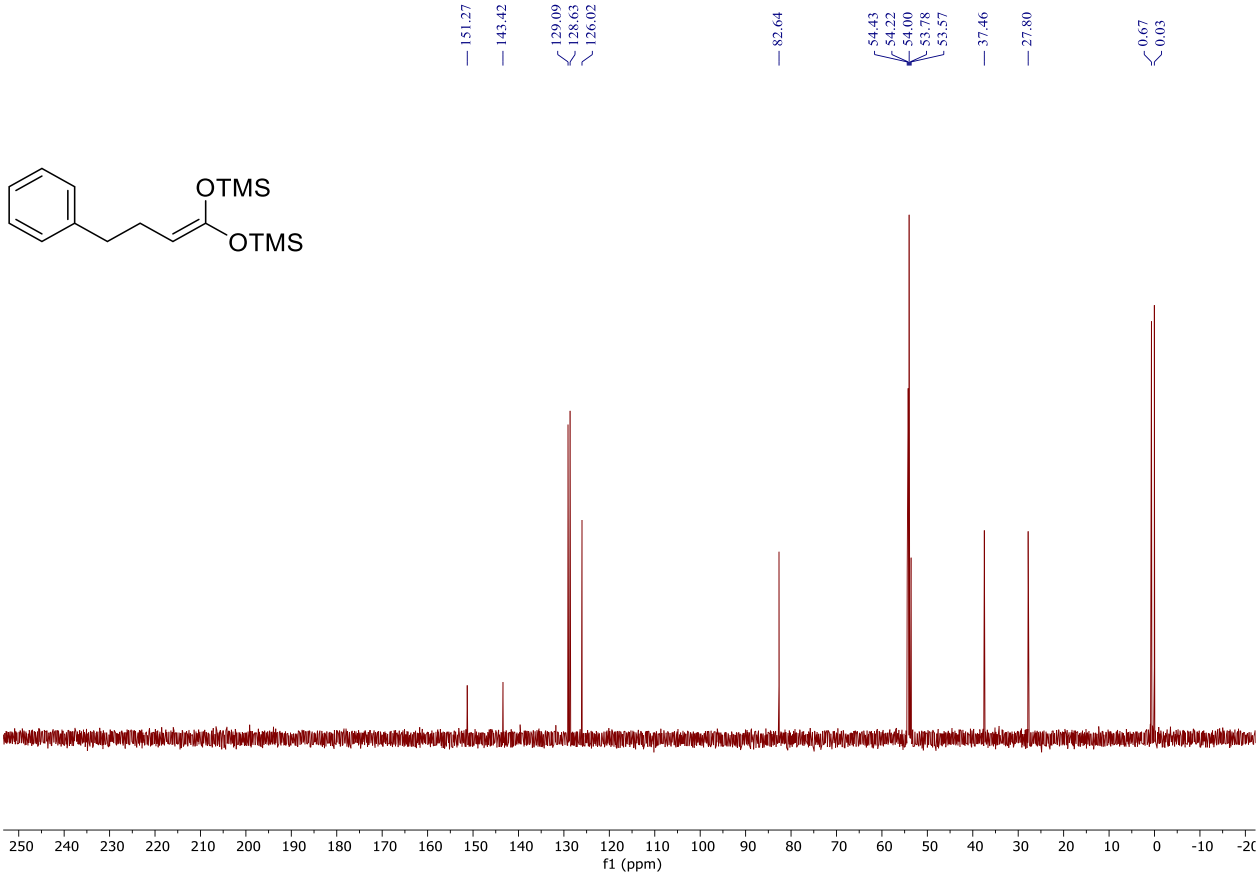
151.27
143.42
129.09
128.63
126.02
82.64
54.43
54.22
54.00
53.78
53.57
37.46
27.80
0.67
0.03
OTMS
OTMS
f1 (ppm)

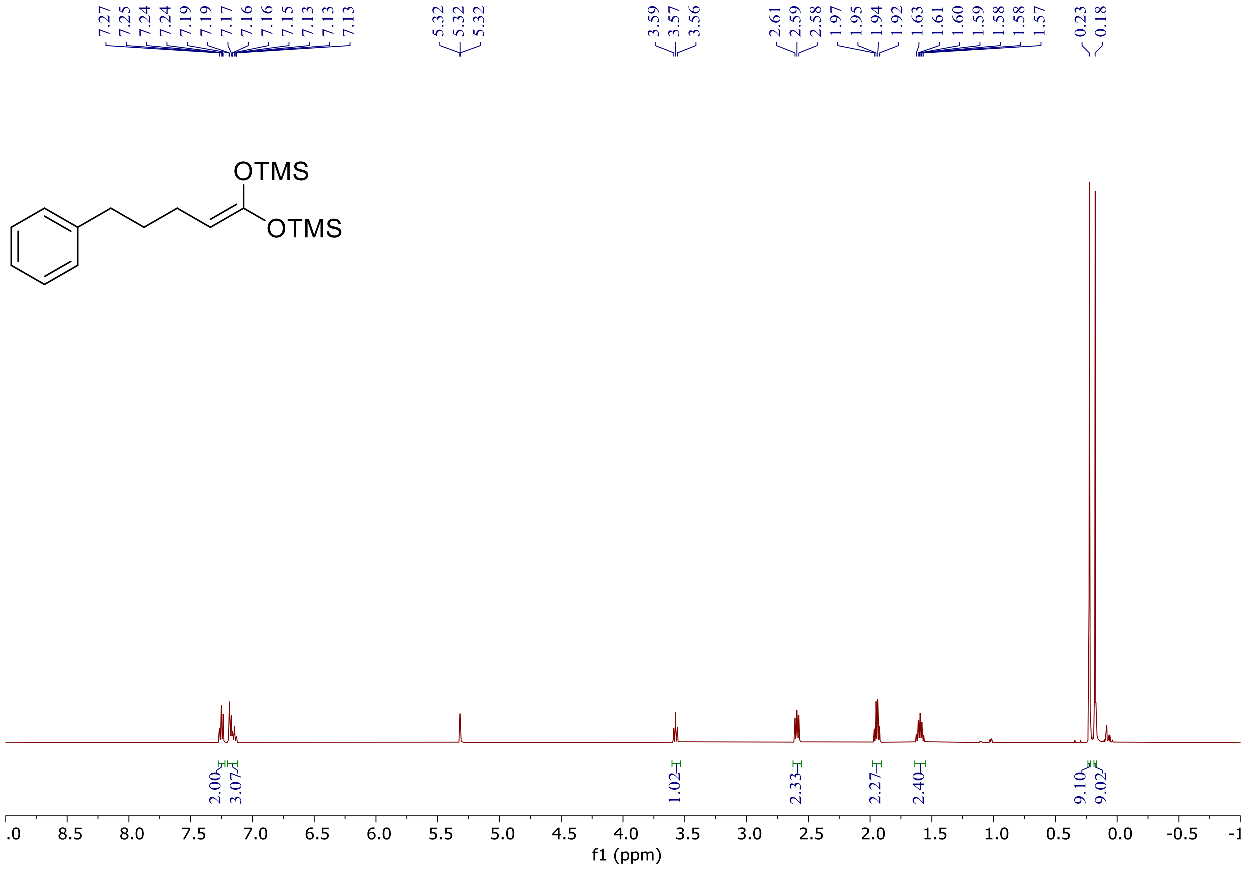
7.27
7.25
7.24
7.24
7.19
7.19
7.17
7.16
7.16
7.15
7.13
7.13
7.13
5.32
5.32
5.32
3.59
3.57
3.56
2.61
2.59
2.58
1.97
1.95
1.94
1.92
1.63
1.61
1.60
1.59
1.58
1.58
1.57
0.23
0.18
OTMS
OTMS
2.00
3.07
1.02
2.33
2.27
2.40
9.10
9.02
f1 (ppm)

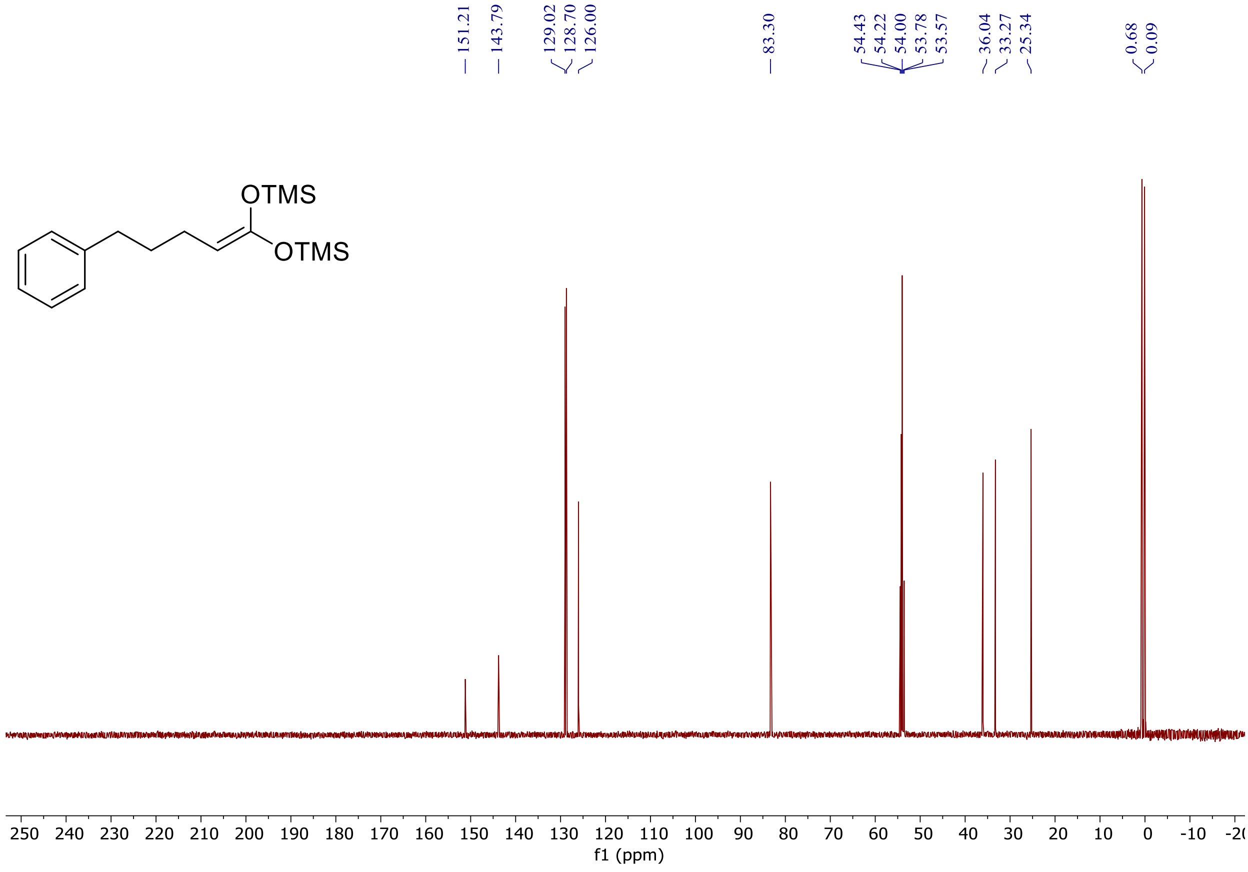
151.21
143.79
129.02
128.70
126.00
83.30
54.43
54.22
54.00
53.78
53.57
36.04
33.27
25.34
0.68
0.09
OTMS
OTMS
f1 (ppm)

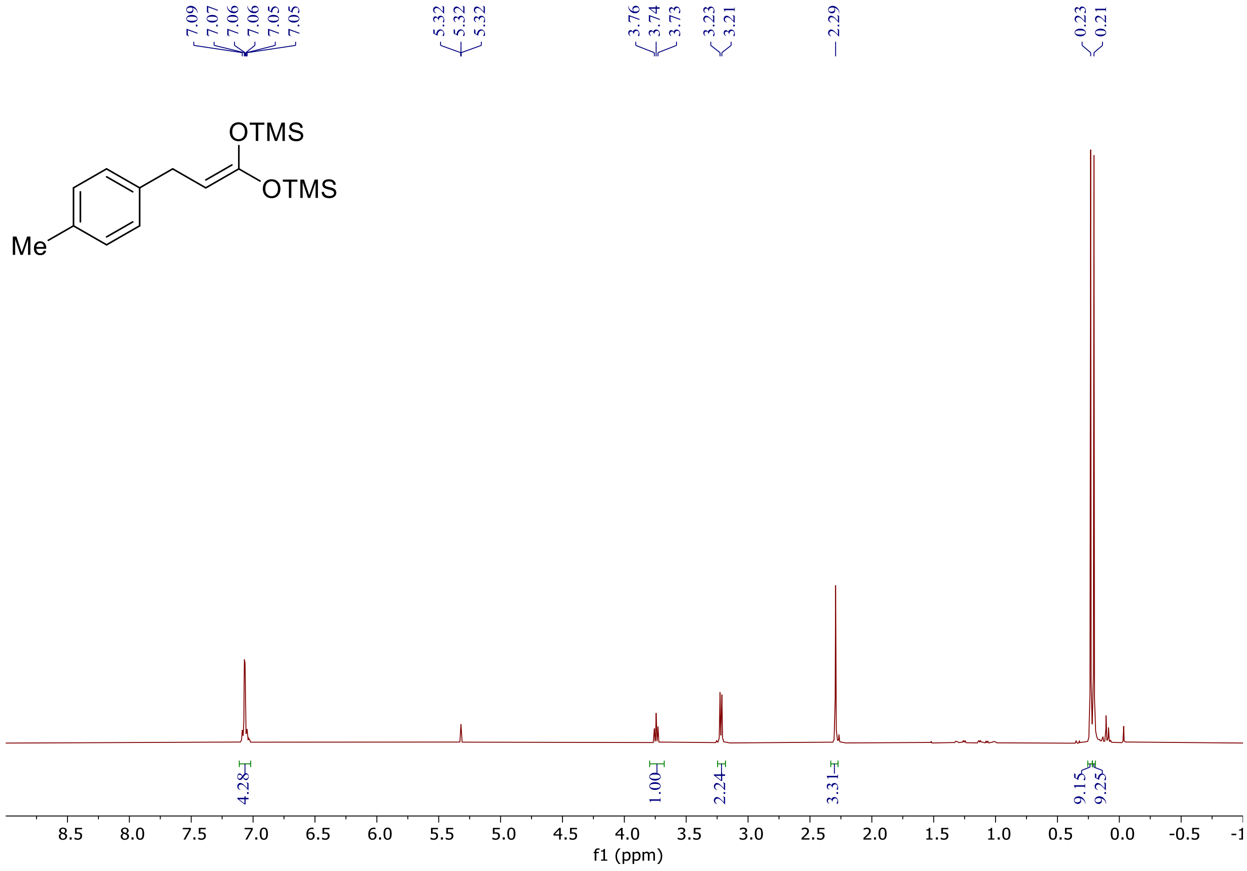
7.09
7.07
7.06
7.06
7.05
7.05
5.32
5.32
5.32
3.76
3.74
3.73
3.23
3.21
2.29
0.23
0.21
OTMS
OTMS
Me
4.28
1.00
2.24
3.31
9.15
9.25
f1 (ppm)

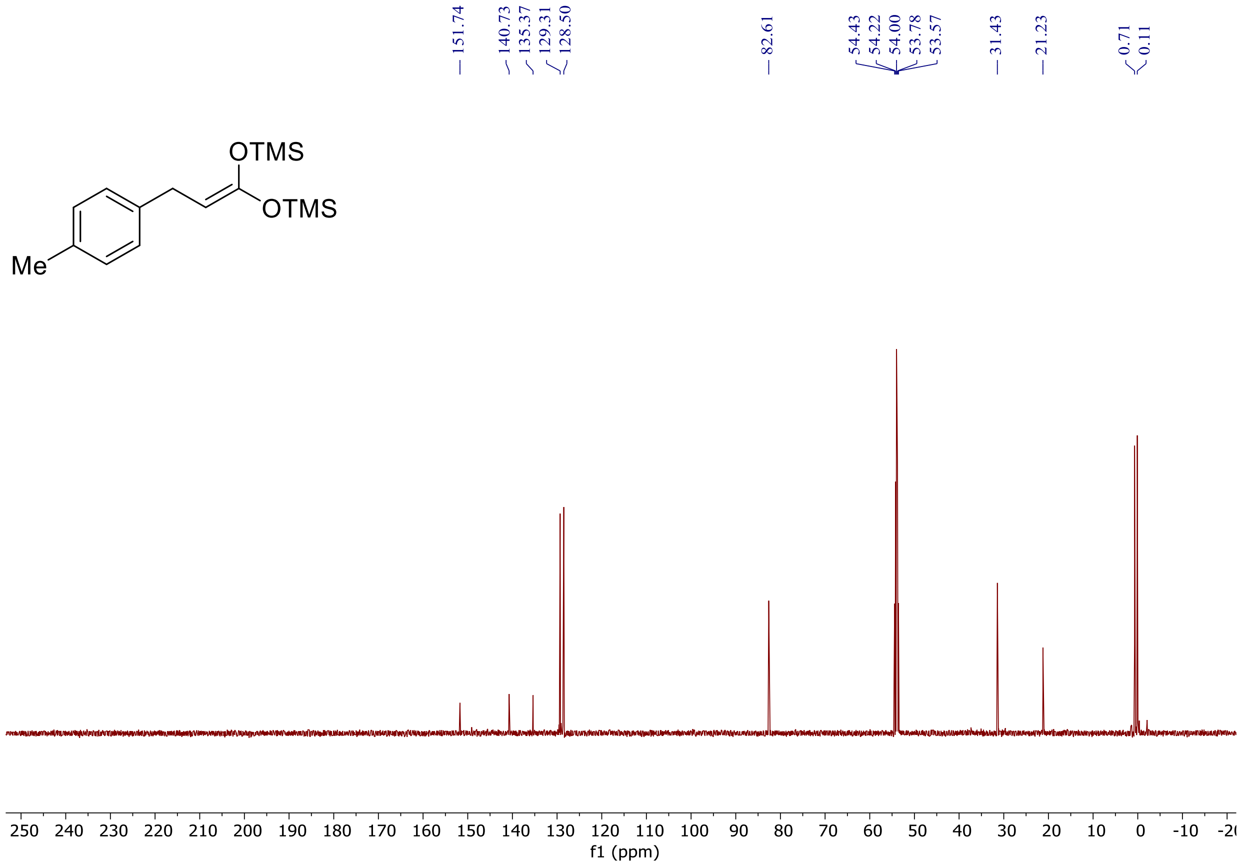
151.74
140.73
135.37
129.31
128.50
82.61
54.43
54.22
54.00
53.78
53.57
31.43
21.23
0.71
0.11
OTMS
OTMS
Me
f1 (ppm)

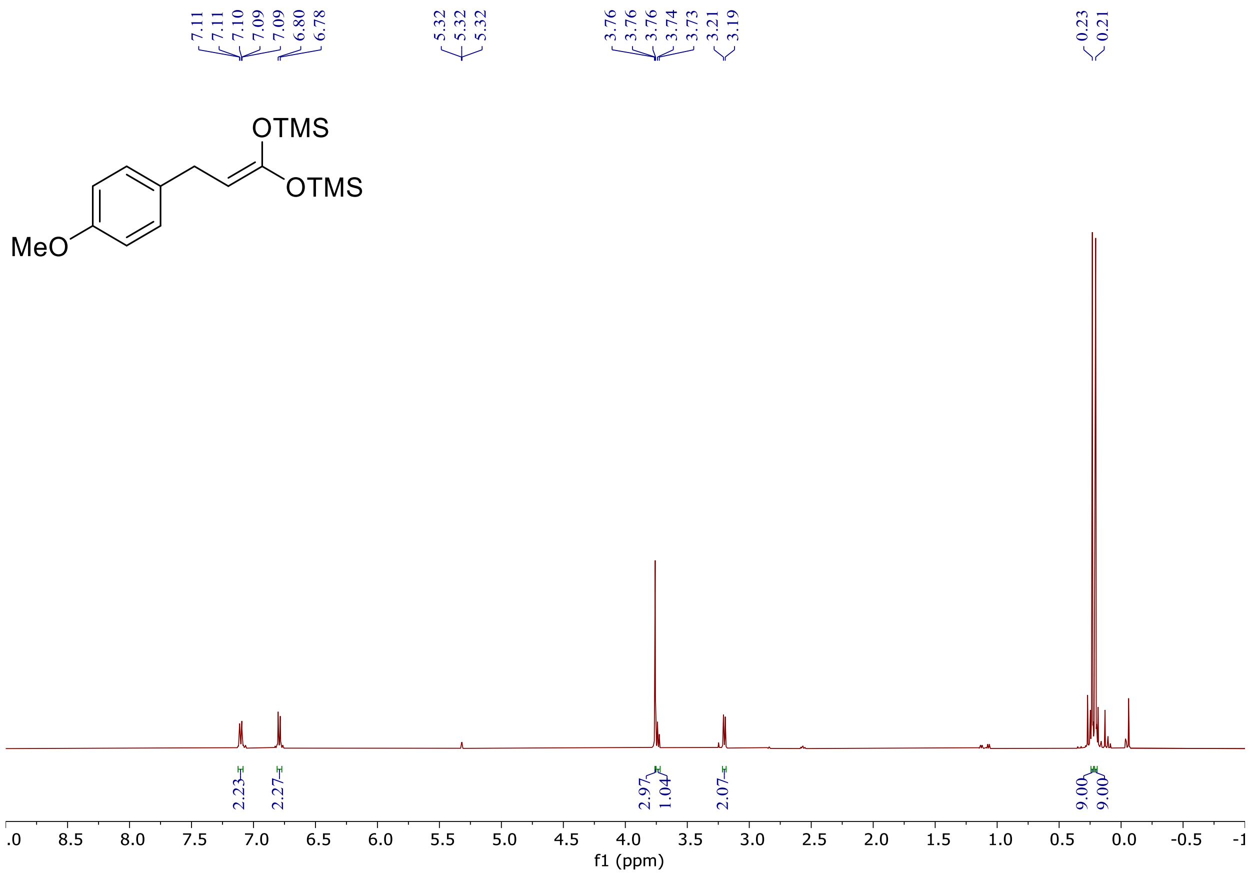
7.11
7.11
7.10
7.09
7.09
6.80
6.78
5.32
5.32
5.32
3.76
3.76
3.76
3.74
3.73
3.21
3.19
0.23
0.21
OTMS
OTMS
MeO
2.23
2.27
2.97
1.04
2.07
9.00
9.00
f1 (ppm)

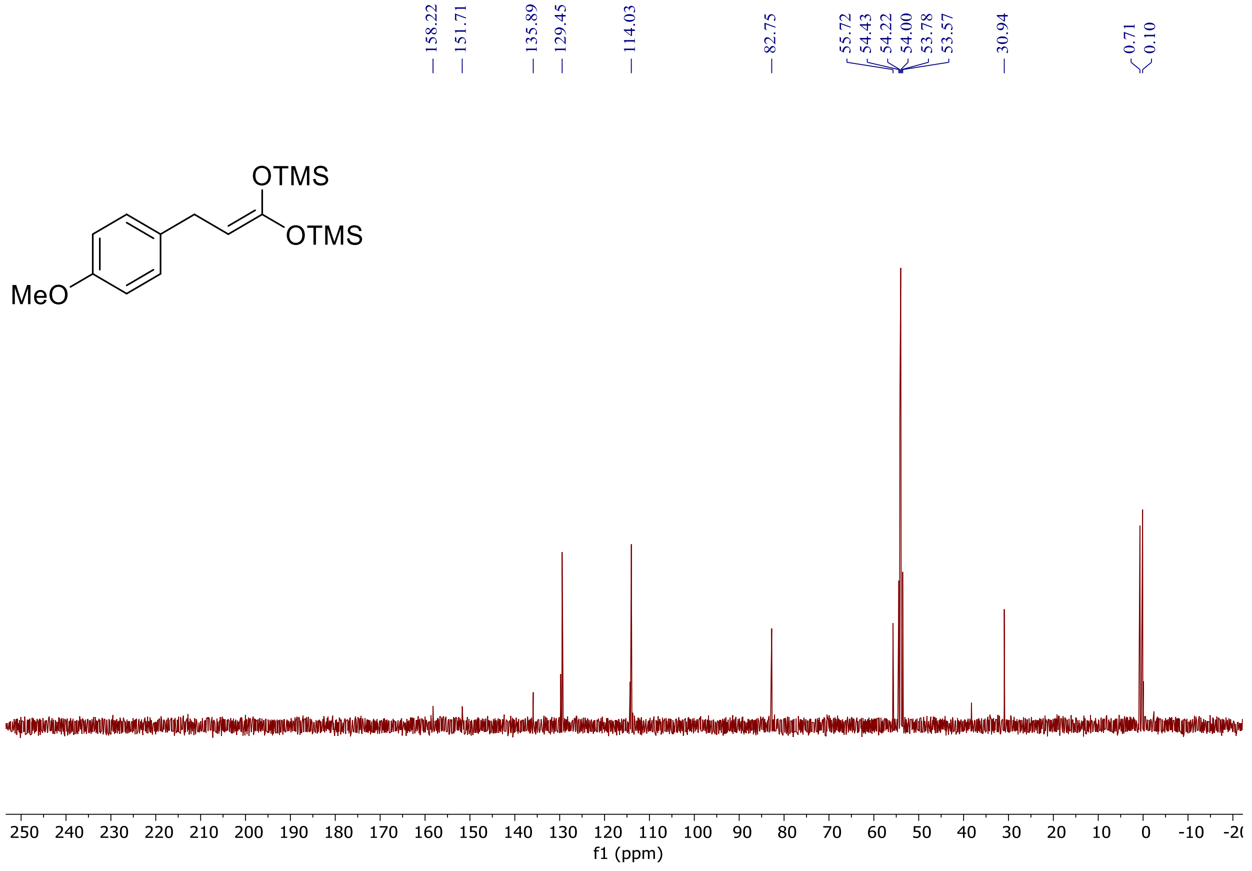
158.22
151.71
135.89
129.45
114.03
82.75
55.72
54.43
54.22
54.00
53.78
53.57
30.94
0.71
0.10
OTMS
OTMS
MeO
f1 (ppm)

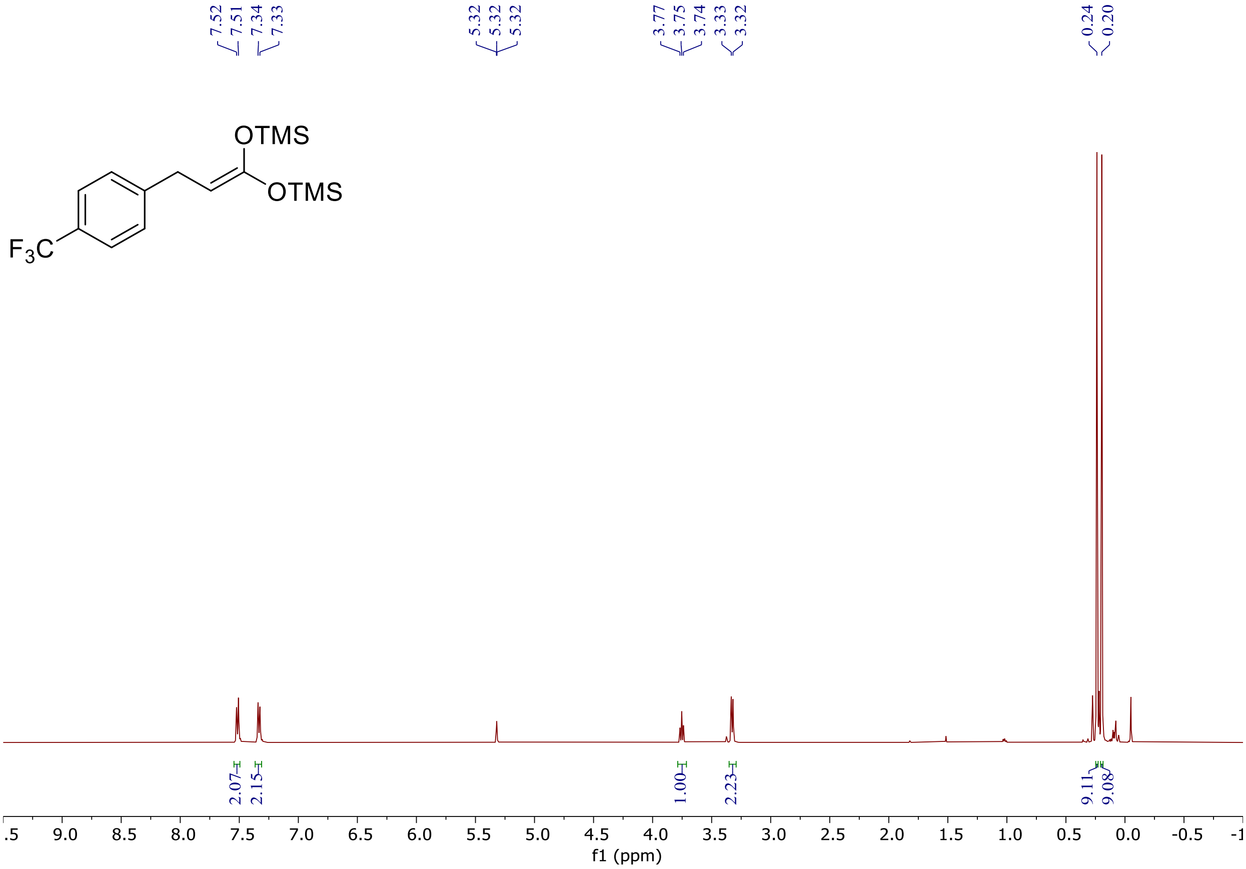
7.52
7.51
7.34
7.33
5.32
5.32
5.32
3.77
3.75
3.74
3.33
3.32
0.24
0.20
OTMS
OTMS
F3C
2.07
2.15
1.00
2.23
9.11
9.08
f1 (ppm)

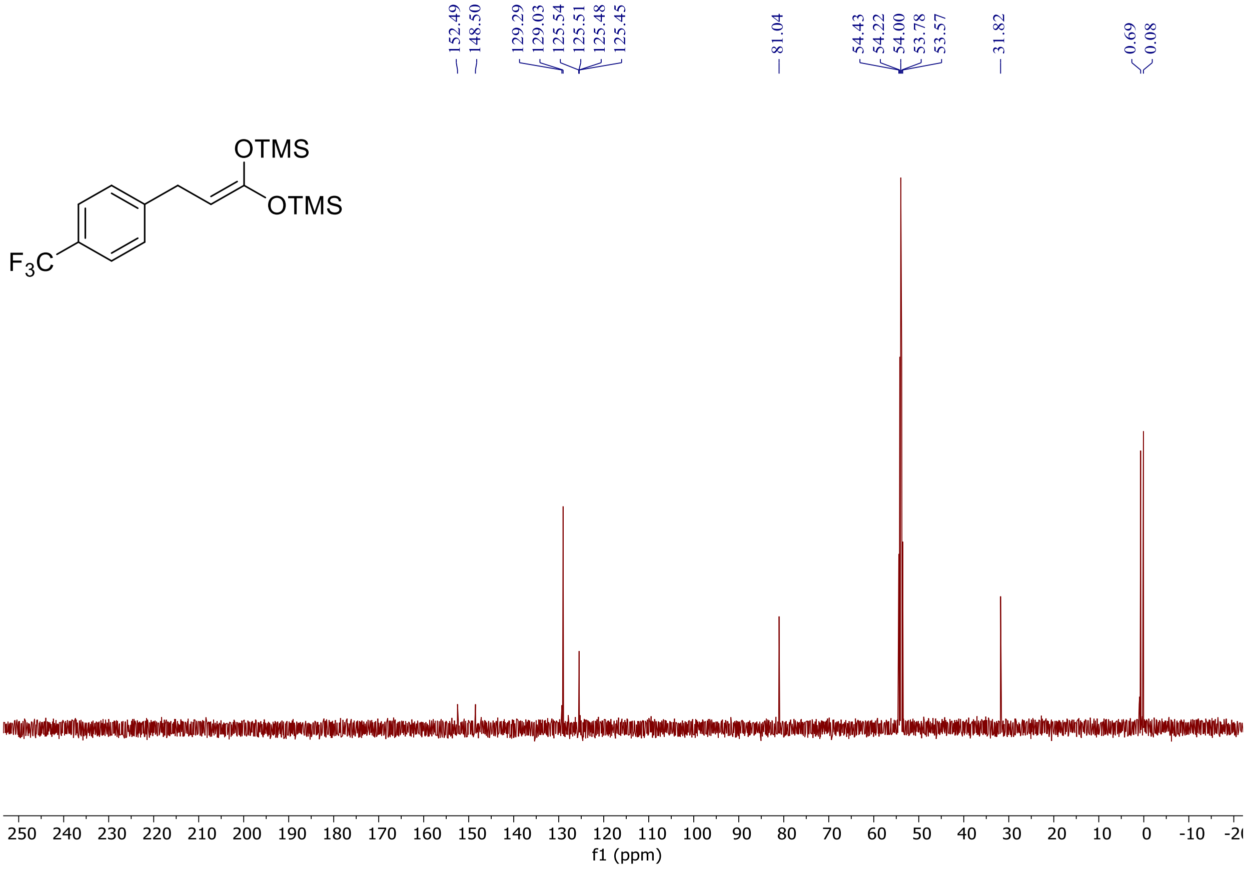
152.49
148.50
129.29
129.03
125.54
125.51
125.48
125.45
81.04
54.43
54.22
54.00
53.78
53.57
31.82
0.69
0.08
OTMS
OTMS
F3C
f1 (ppm)

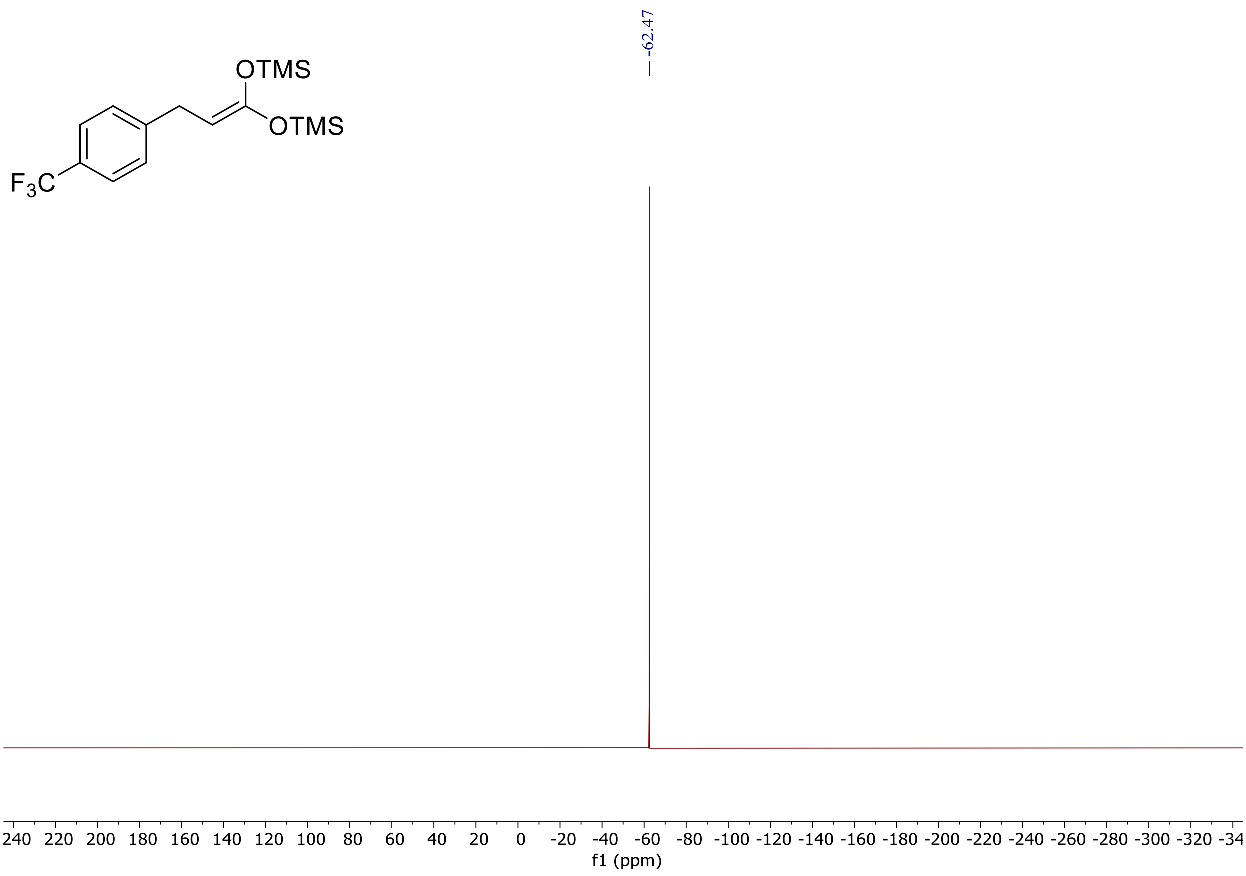

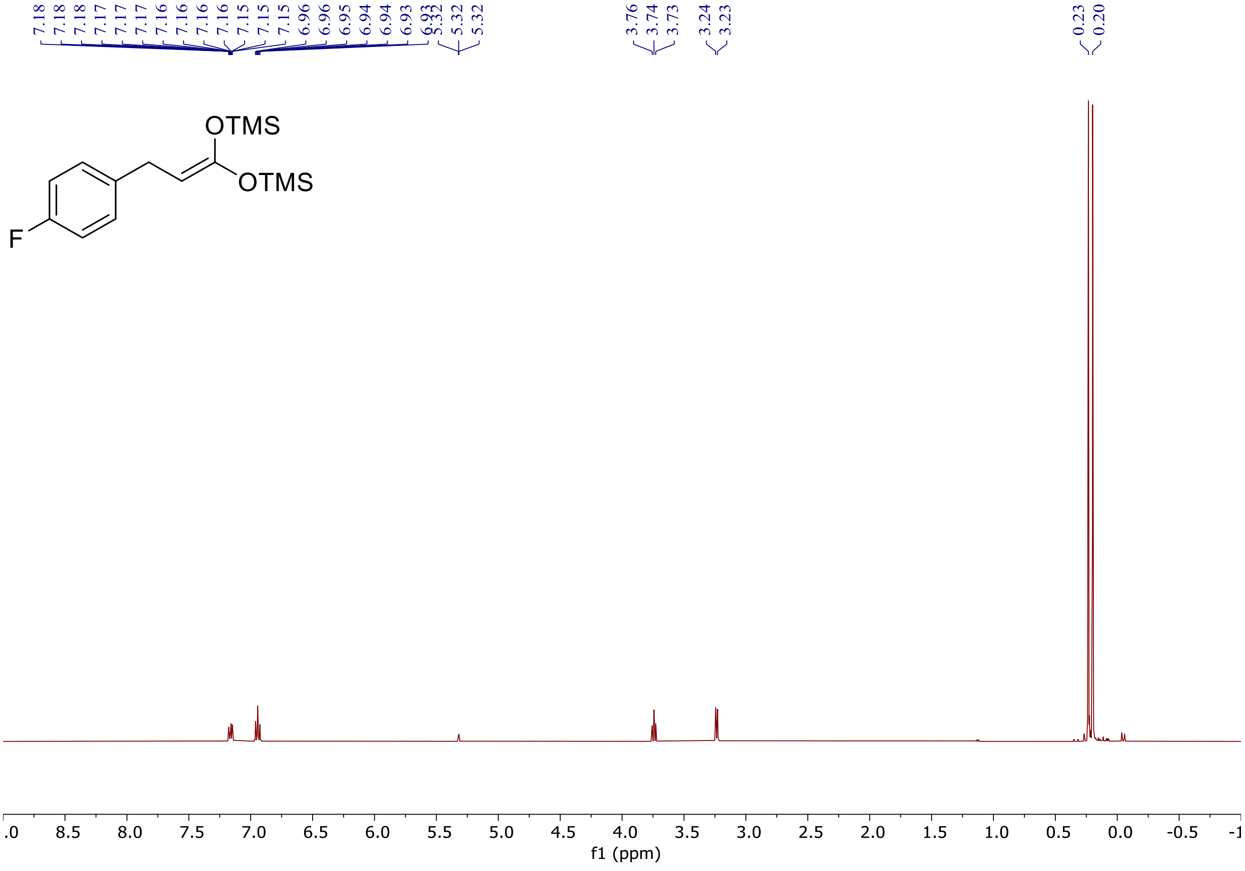

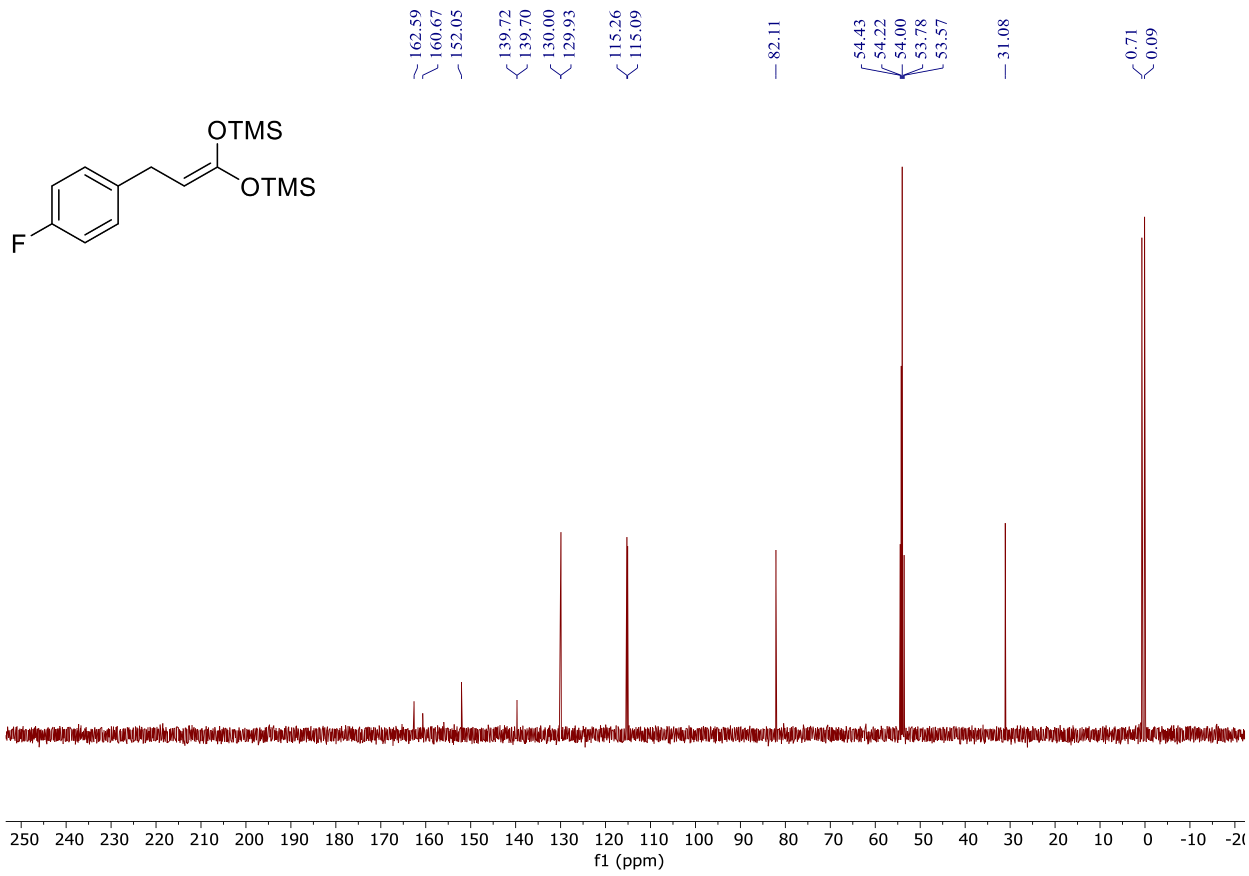
162.59
160.67
152.05
139.72
139.70
130.00
129.93
115.26
115.09
82.11
54.43
54.22
54.00
53.78
53.57
31.08
0.71
0.09
OTMS
OTMS
F
f1 (ppm)

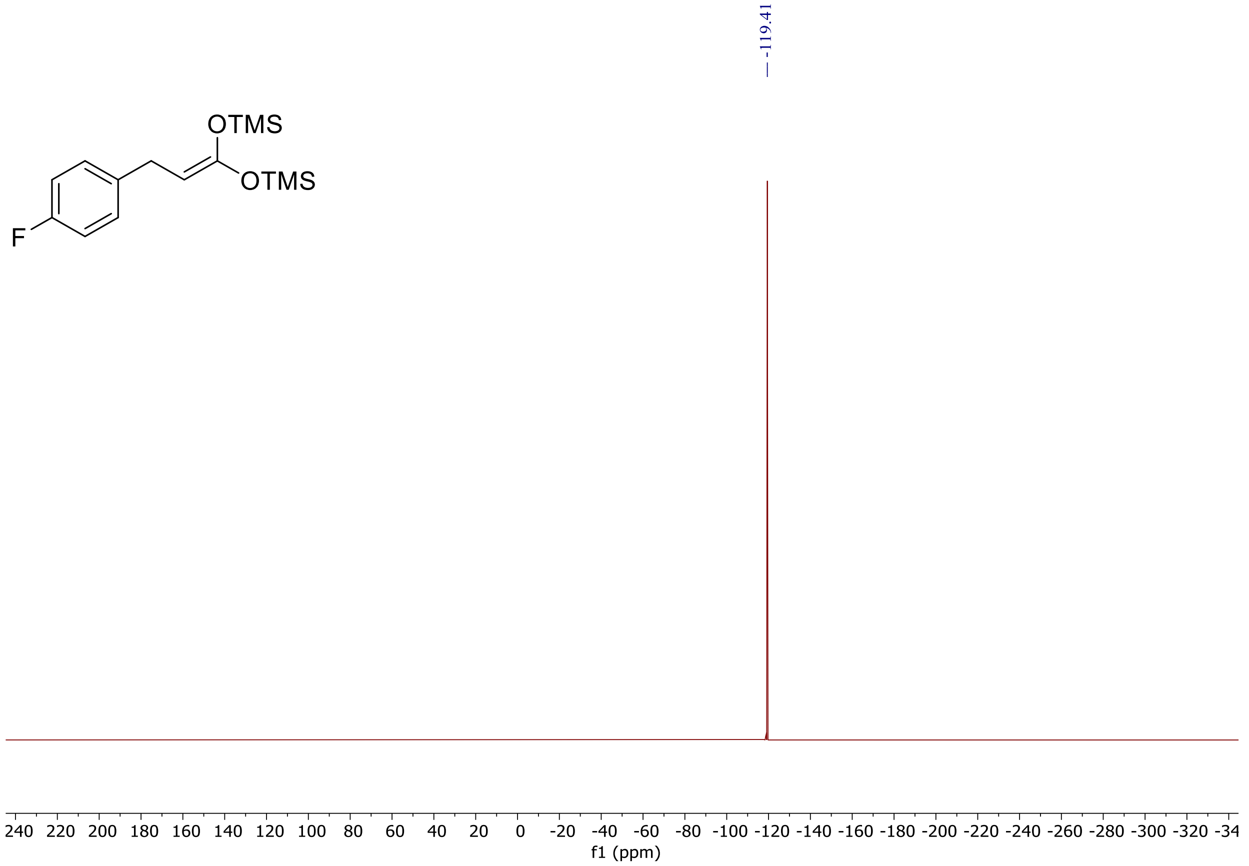
-119.41
OTMS
OTMS
F
f1 (ppm)

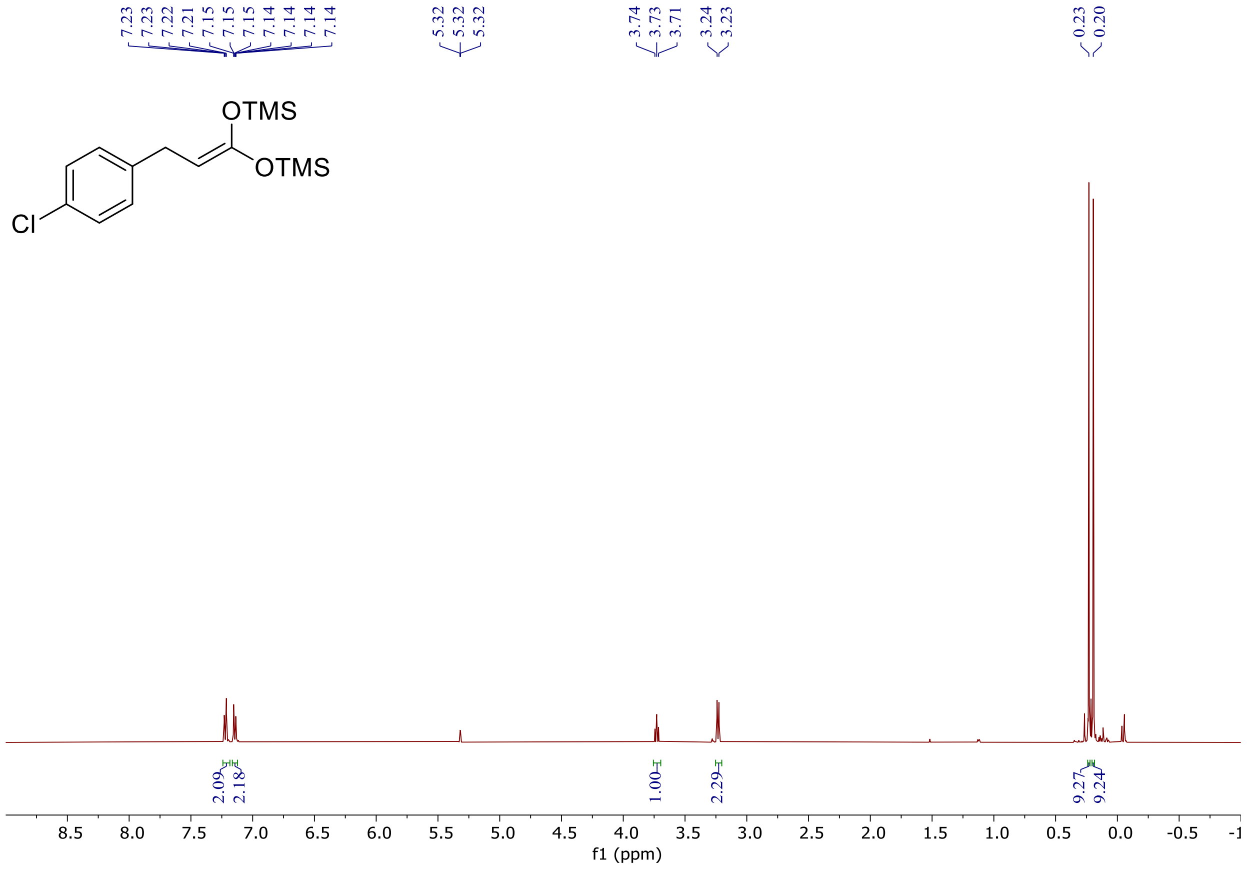

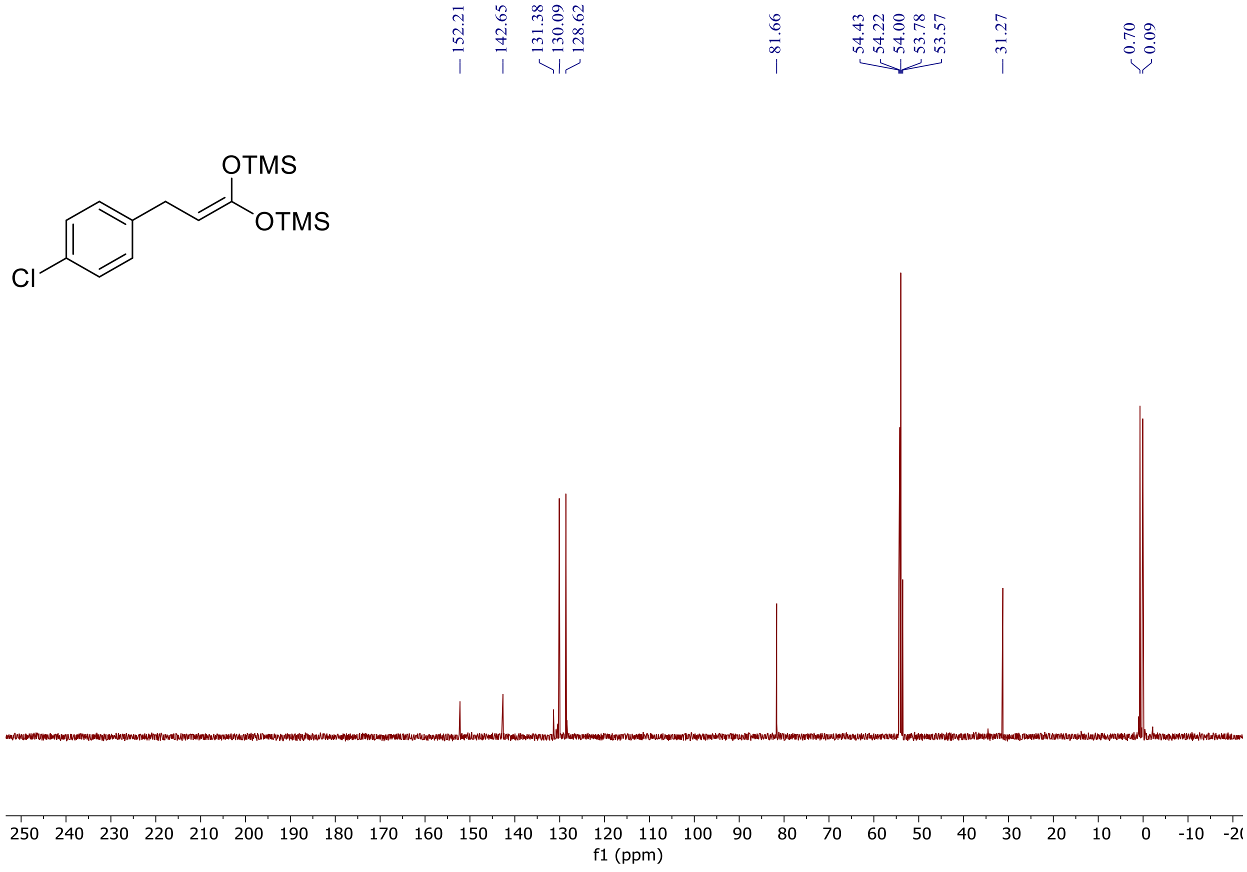

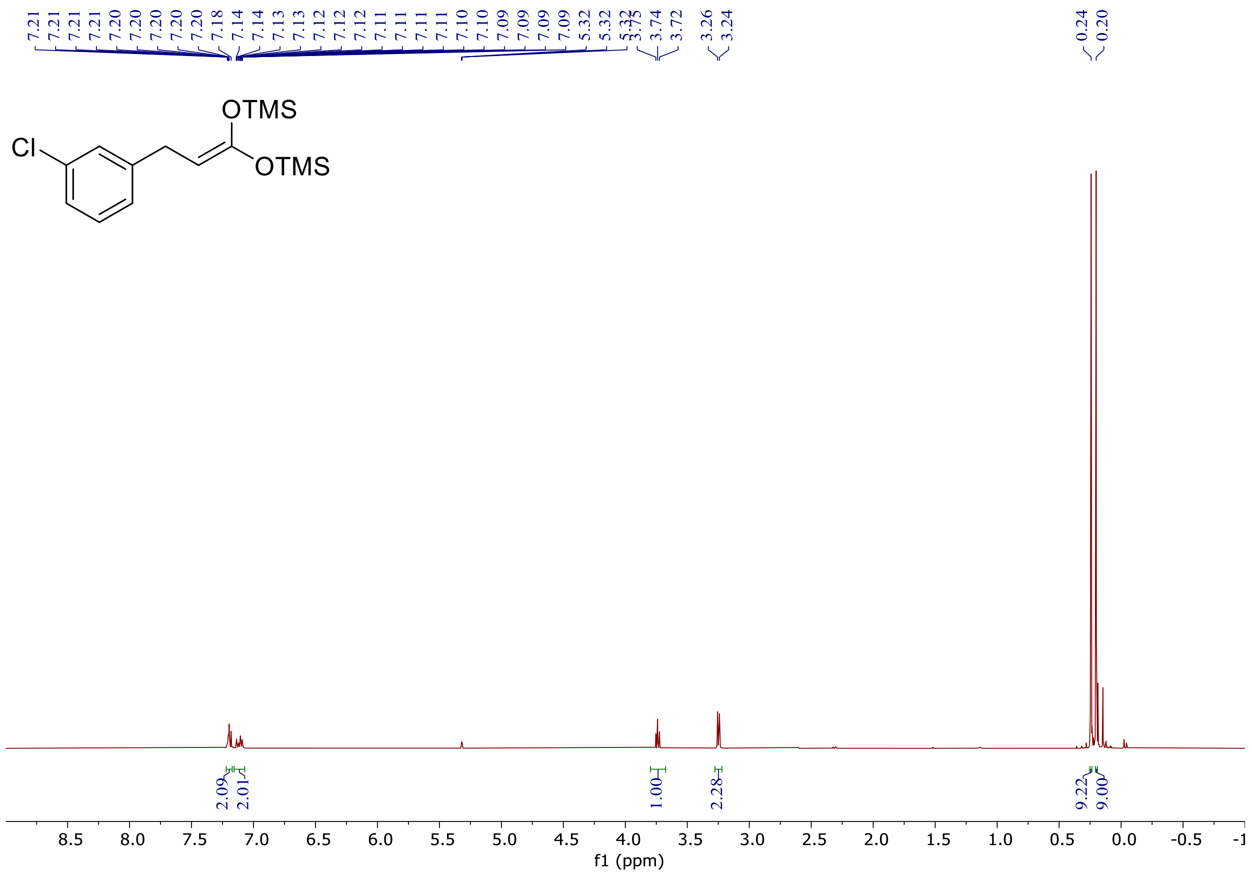

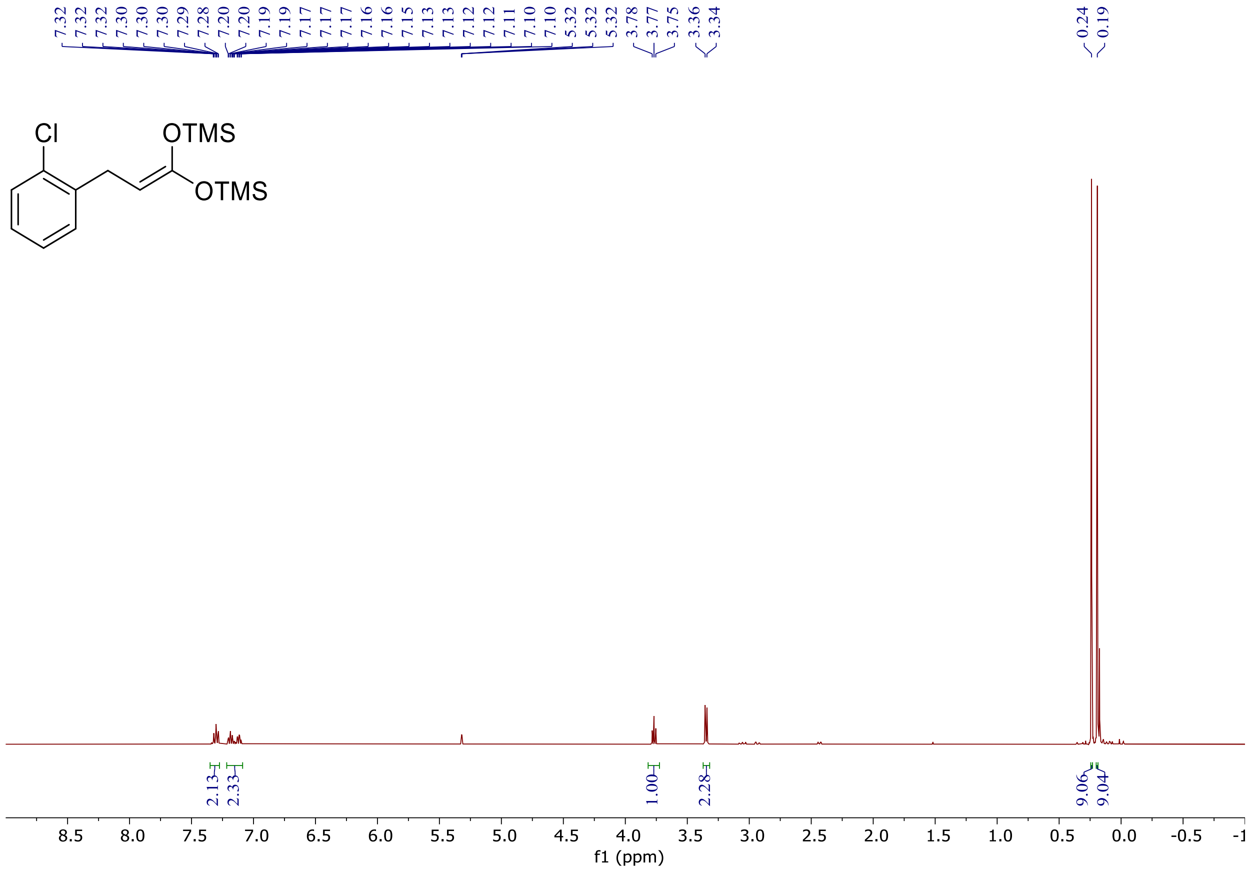

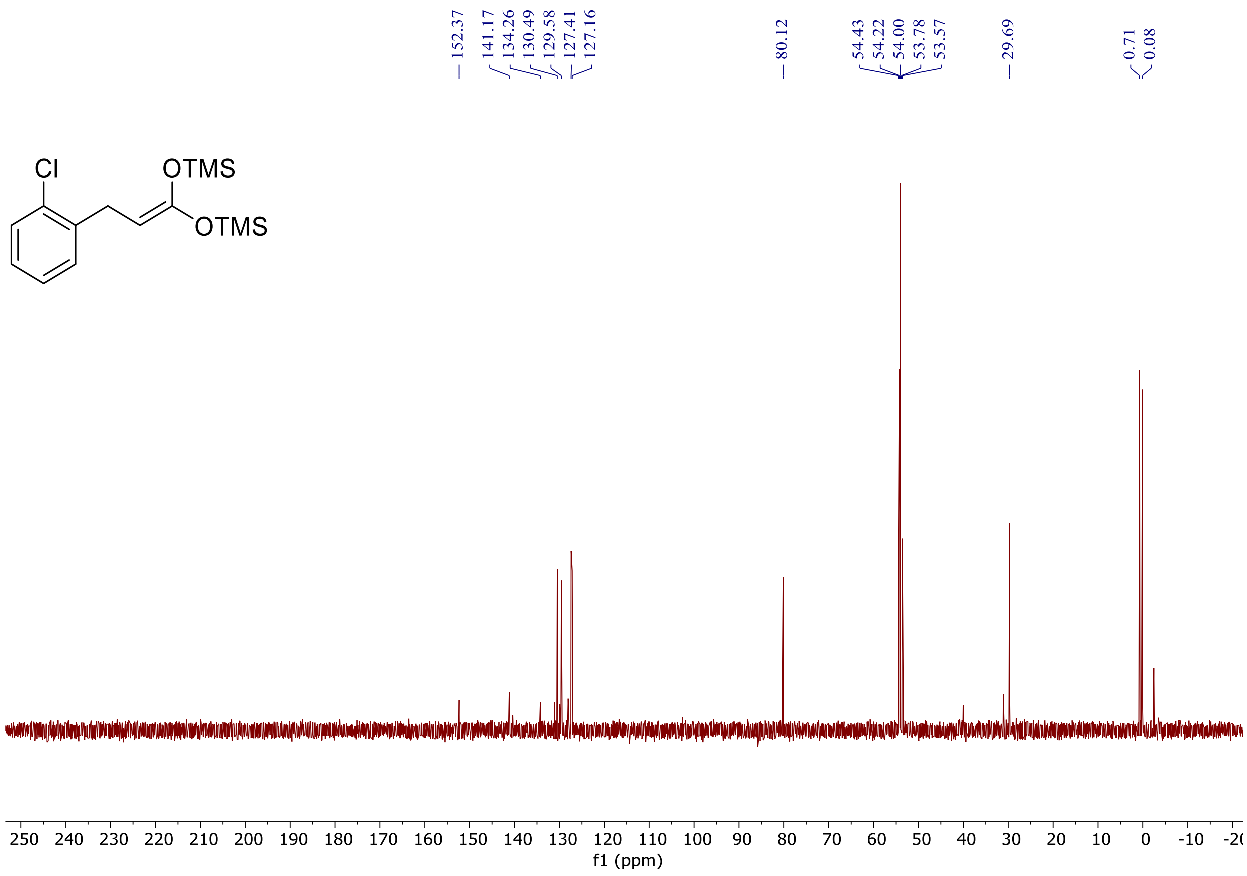

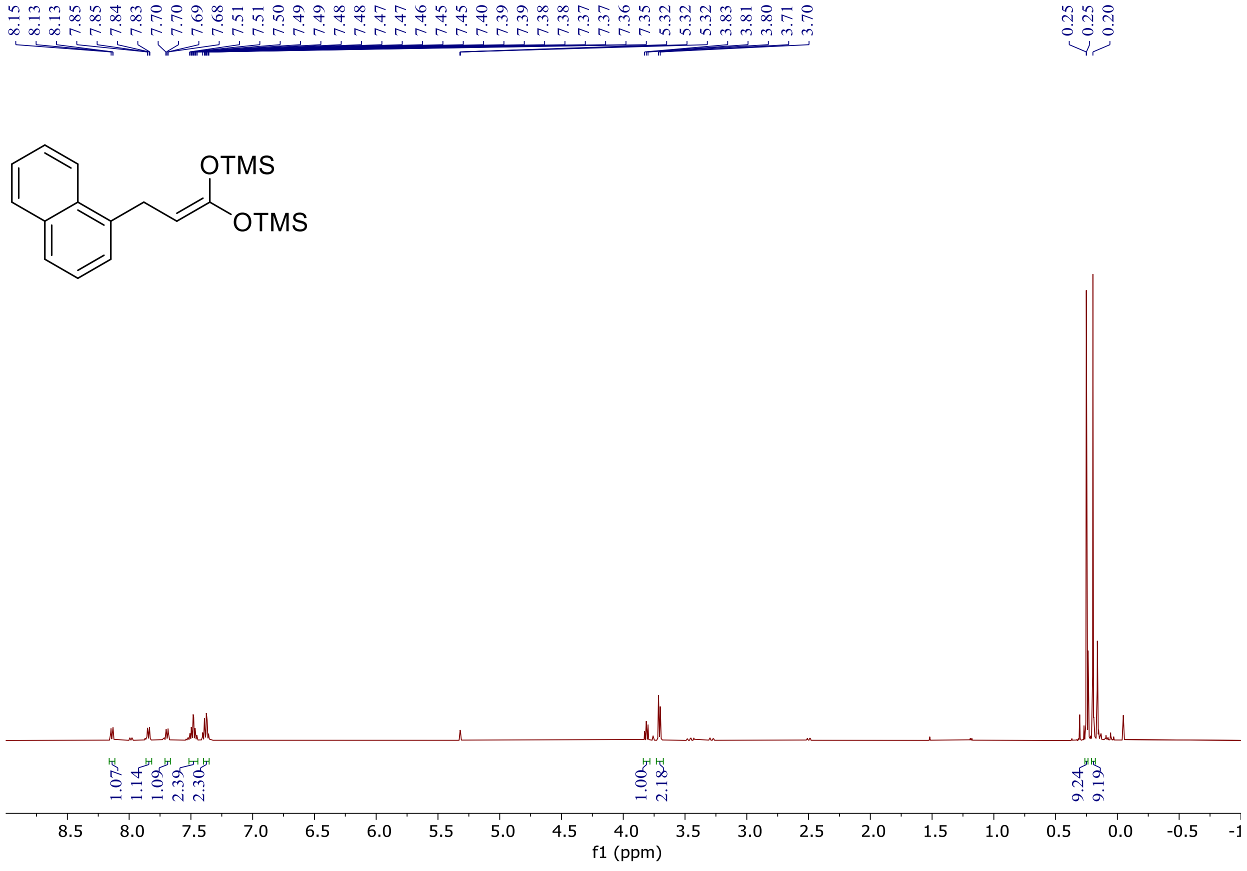

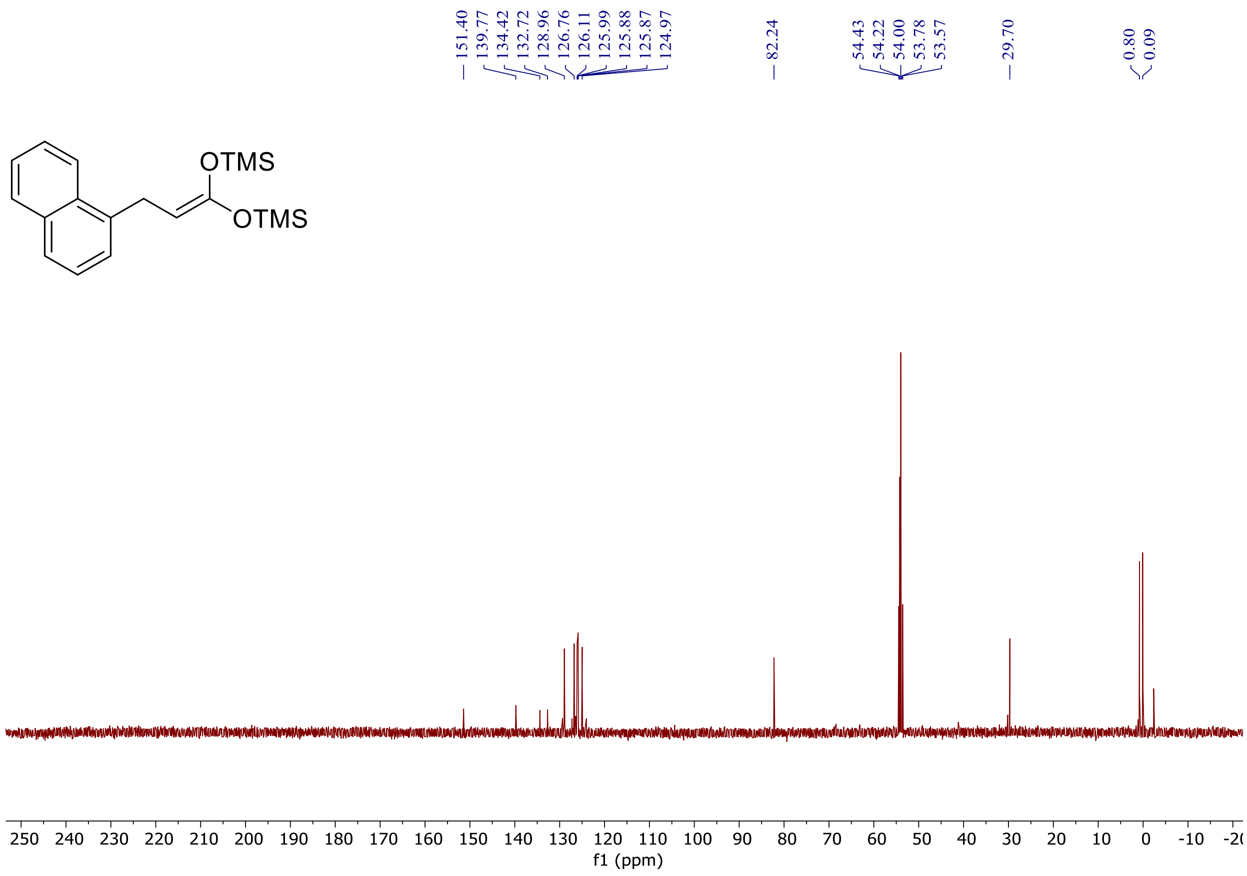
151.40
139.77
134.42
132.72
128.96
126.76
126.11
125.99
125.88
125.87
124.97
82.24
54.43
54.22
54.00
53.78
53.57
29.70
0.80
0.09
OTMS
OTMS
250 240 230 220 210 200 190 180 170 160 150 140 130 120 110 100 90 80 70 60 50 40 30 20 10 0 -10 -20
f1 (ppm)

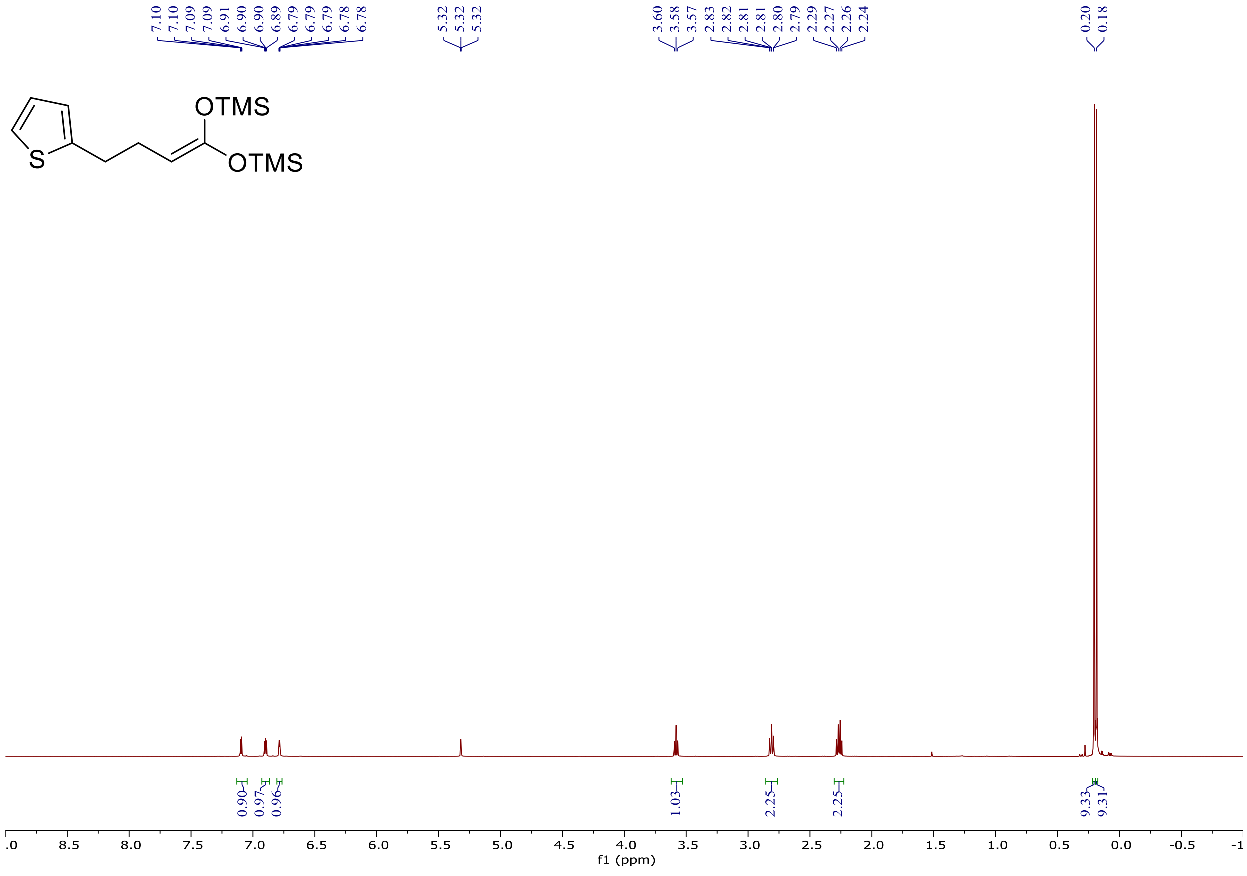
7.10
7.10
7.09
7.09
6.91
6.90
6.89
6.79
6.79
6.78
6.78
5.32
5.32
5.32
3.60
3.58
3.57
2.83
2.82
2.81
2.81
2.80
2.79
2.29
2.27
2.26
2.24
0.20
0.18
S
OTMS
OTMS
0.90
0.97
0.96
1.03
2.25
2.25
9.33
9.31
9.0 8.5 8.0 7.5 7.0 6.5 6.0 5.5 5.0 4.5 4.0 3.5 3.0 2.5 2.0 1.5 1.0 0.5 0.0 -0.5 -1
f1 (ppm)

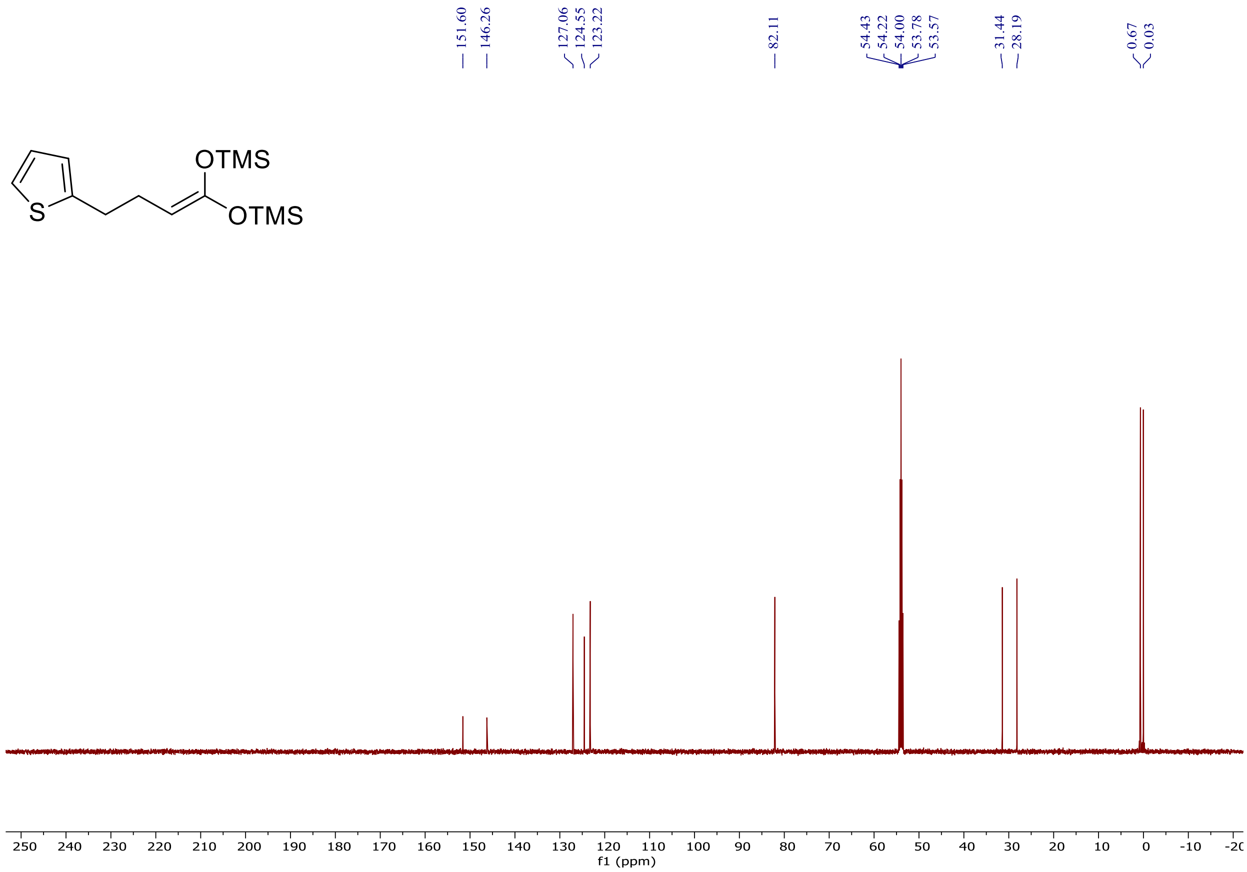
151.60
146.26
127.06
124.55
123.22
82.11
54.43
54.22
54.00
53.78
53.57
31.44
28.19
0.67
0.03
OTMS
OTMS
S
f1 (ppm)

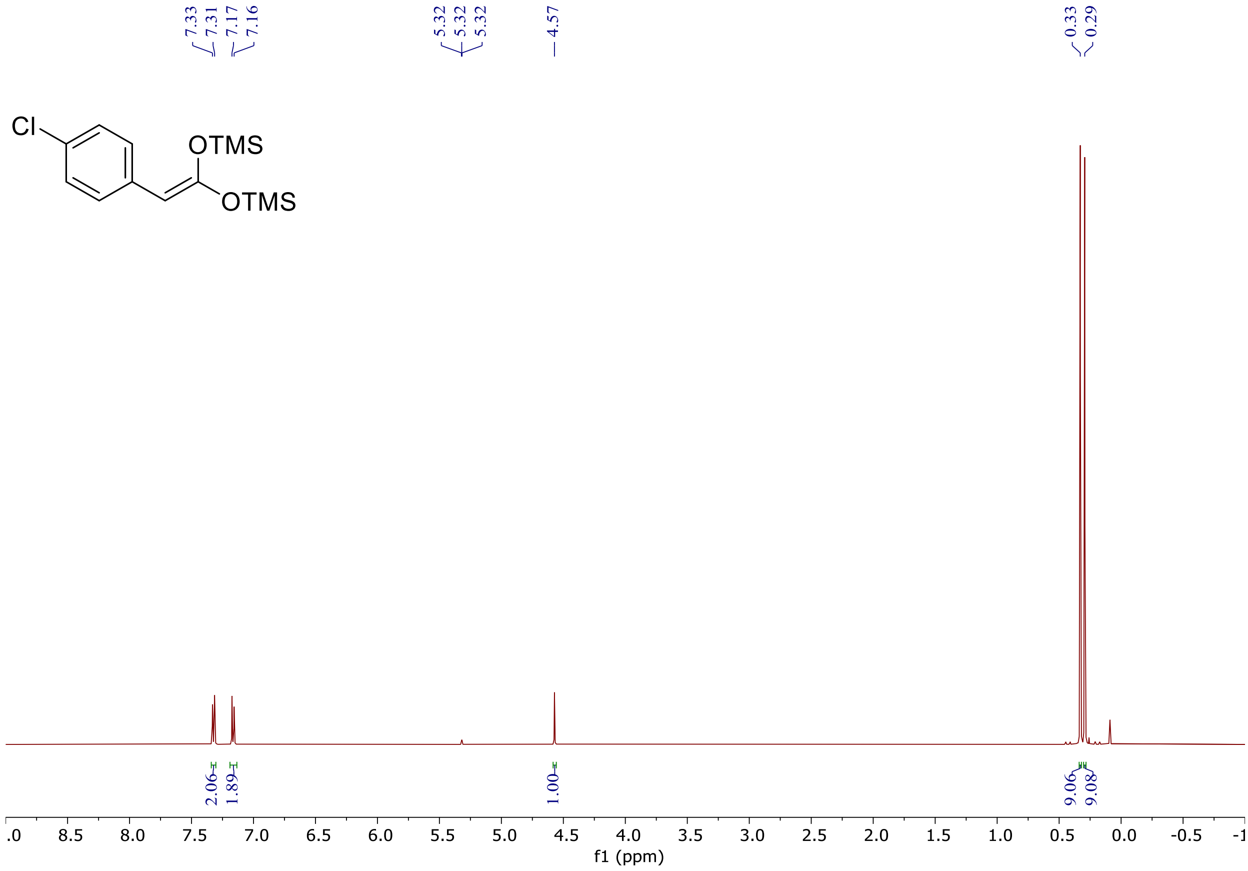
7.33
7.31
7.17
7.16
5.32
5.32
5.32
4.57
0.33
0.29
Cl
OTMS
OTMS
2.06
1.89
1.00
9.06
9.08
f1 (ppm)

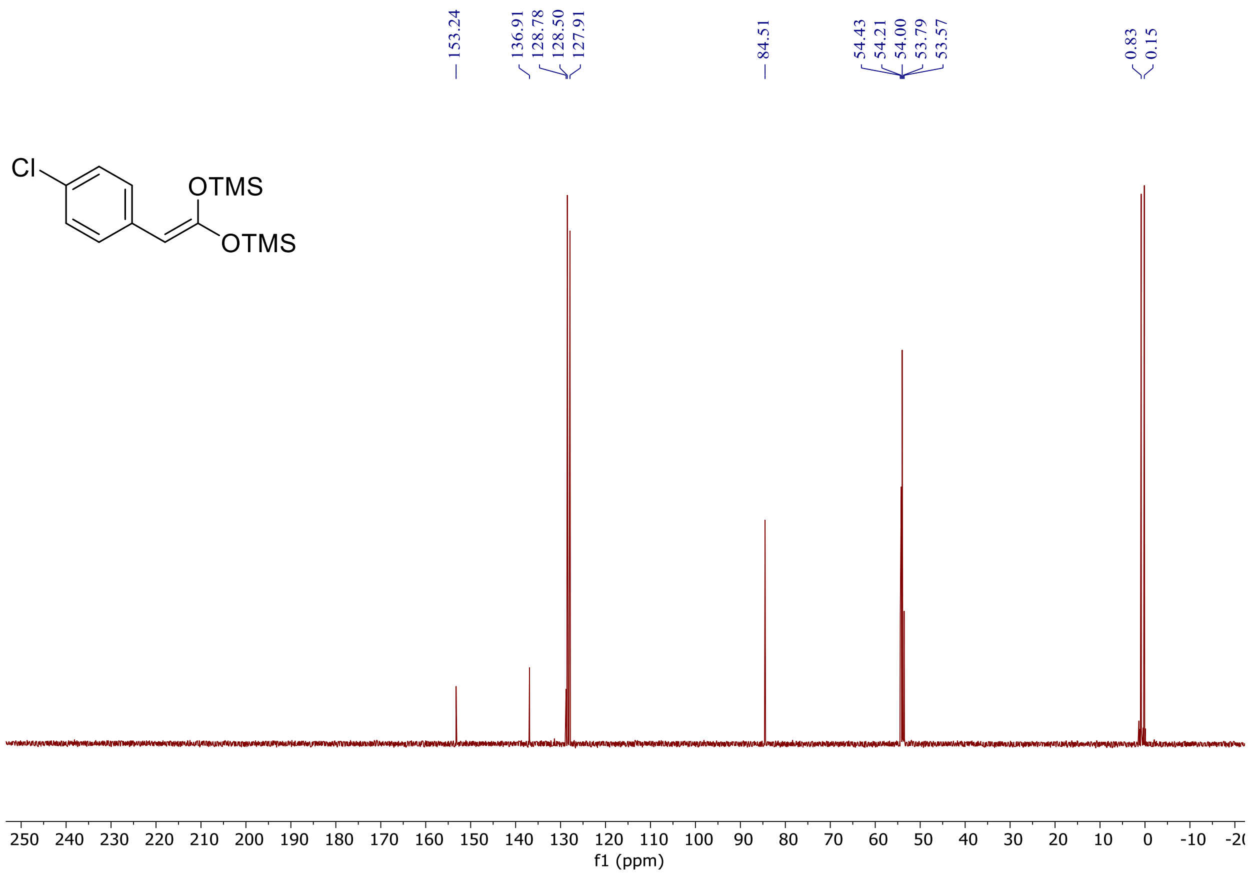

153.24
136.91
128.78
128.50
127.91
84.51
54.43
54.21
54.00
53.79
53.57
0.83
0.15
Cl
OTMS
OTMS
f1 (ppm)

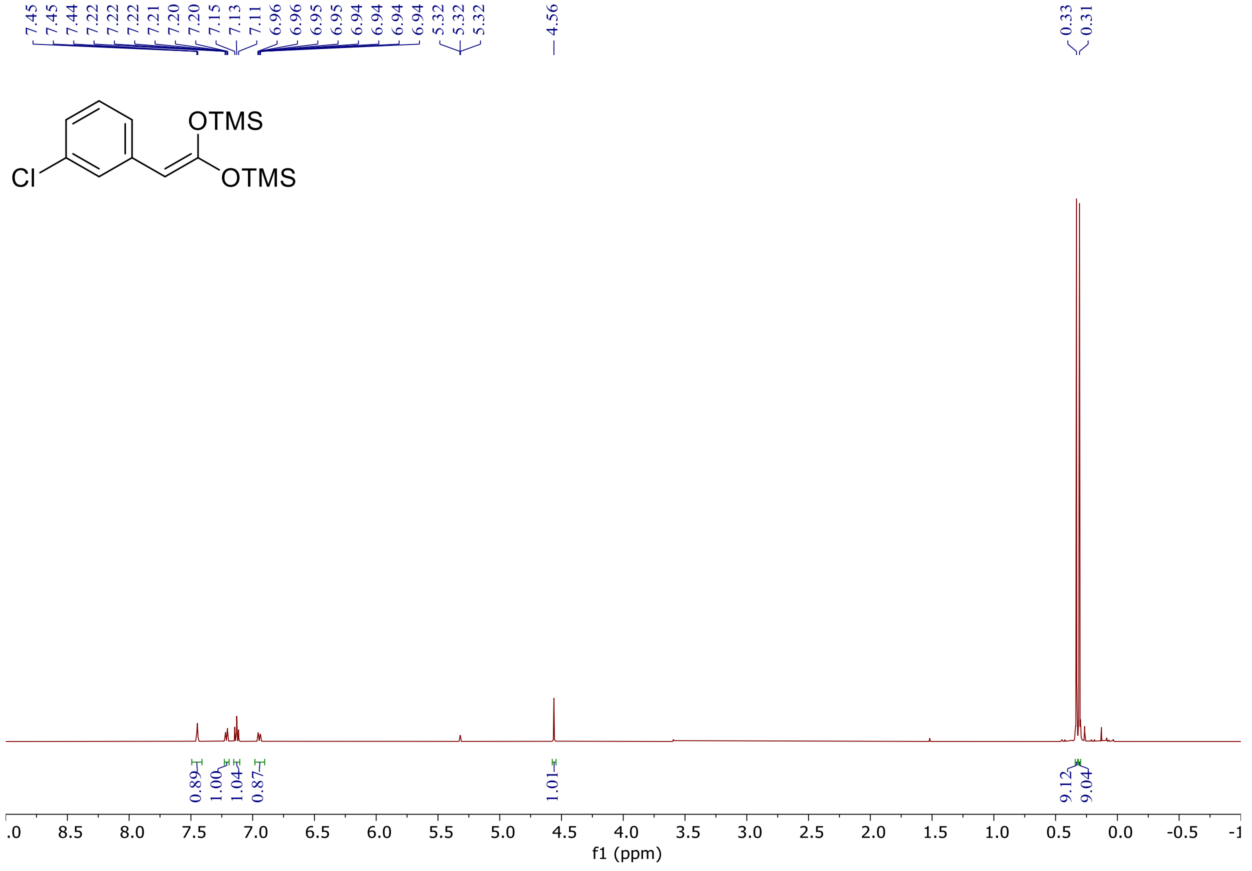

7.45
7.45
7.44
7.22
7.22
7.22
7.21
7.20
7.20
7.15
7.13
7.11
6.96
6.96
6.95
6.95
6.94
6.94
6.94
5.32
5.32
5.32
4.56
0.33
0.31
OTMS
Cl
OTMS
0.89
1.00
1.04
0.87
1.01
9.12
9.04
f1 (ppm)

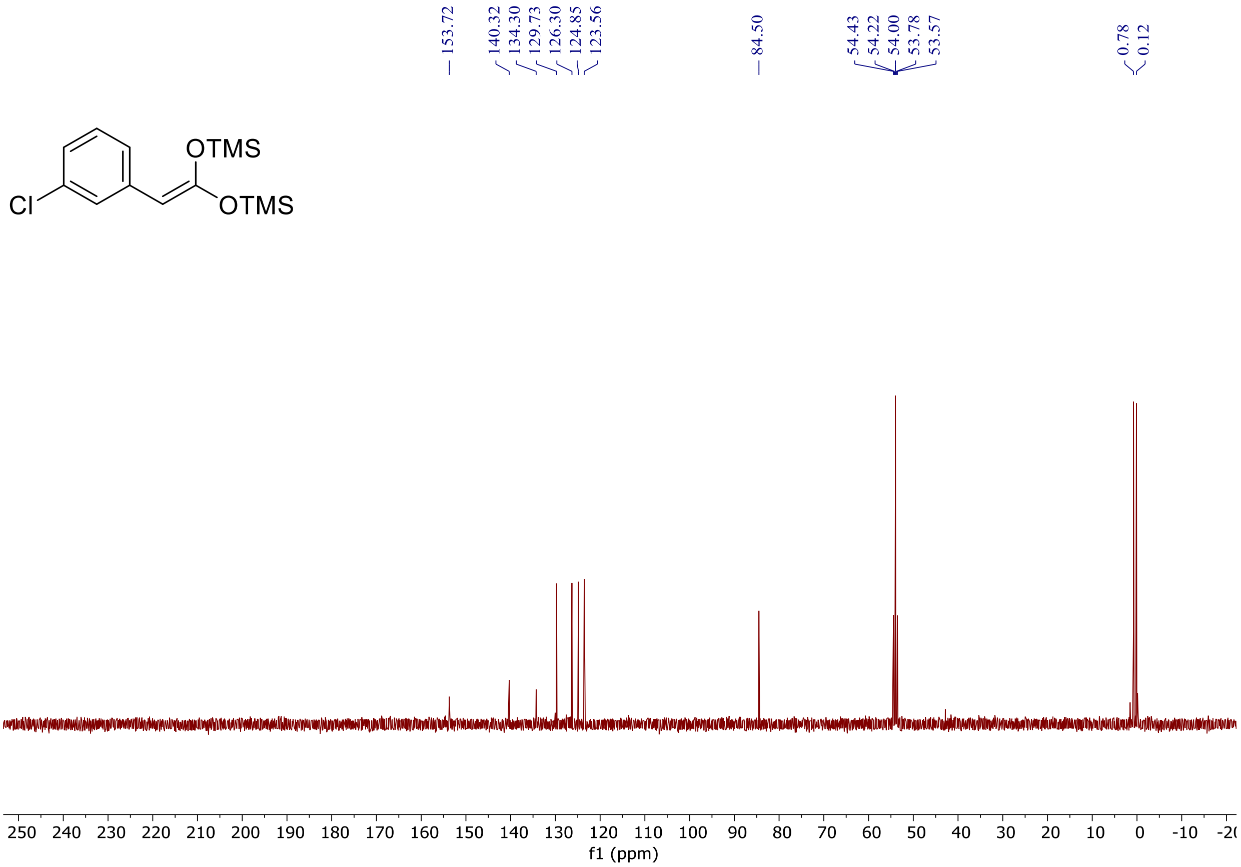
153.72
140.32
134.30
129.73
126.30
124.85
123.56
84.50
54.43
54.22
54.00
53.78
53.57
0.78
0.12
OTMS
Cl
OTMS
250 240 230 220 210 200 190 180 170 160 150 140 130 120 110 100 90 80 70 60 50 40 30 20 10 0 -10
f1 (ppm)

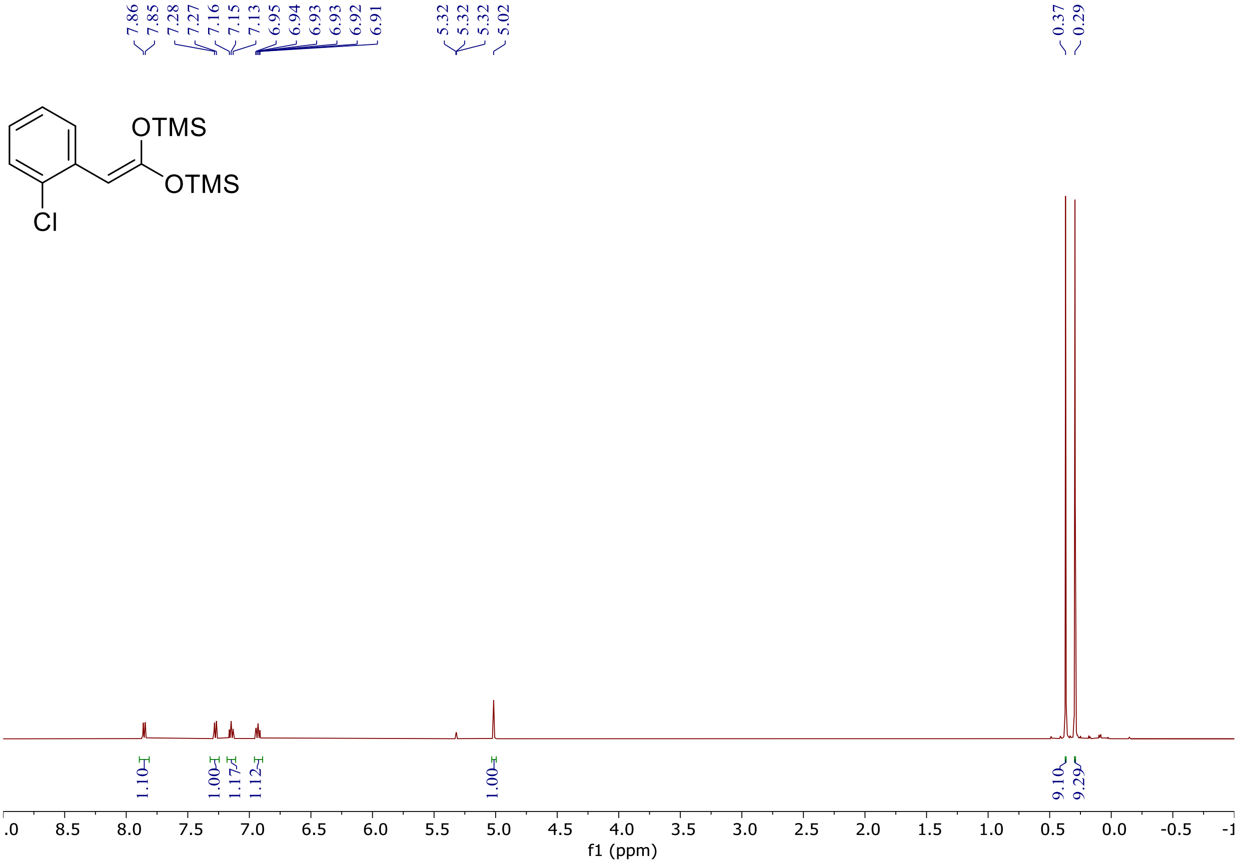
7.86
7.85
7.28
7.27
7.16
7.15
7.13
6.95
6.94
6.93
6.93
6.92
6.91
5.32
5.32
5.32
5.02
0.37
0.29
OTMS
OTMS
Cl
1.10
1.00
1.17
1.12
1.00
9.10
9.29
8.5 8.0 7.5 7.0 6.5 6.0 5.5 5.0 4.5 4.0 3.5 3.0 2.5 2.0 1.5 1.0 0.5 0.0 -0.5
f1 (ppm)

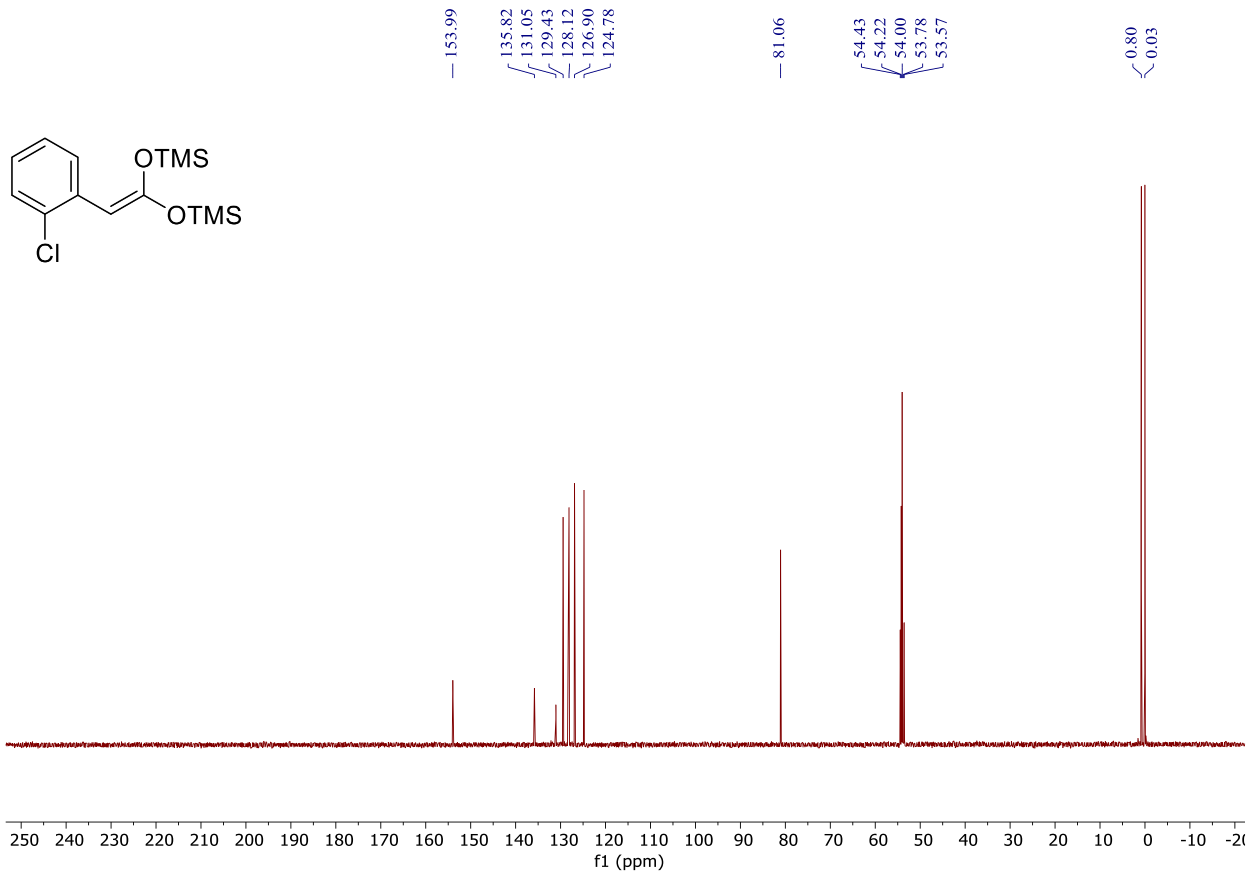
153.99
135.82
131.05
129.43
128.12
126.90
124.78
81.06
54.43
54.22
54.00
53.78
53.57
0.80
0.03
OTMS
OTMS
Cl
250 240 230 220 210 200 190 180 170 160 150 140 130 120 110 100 90 80 70 60 50 40 30 20 10 0 -10
f1 (ppm)

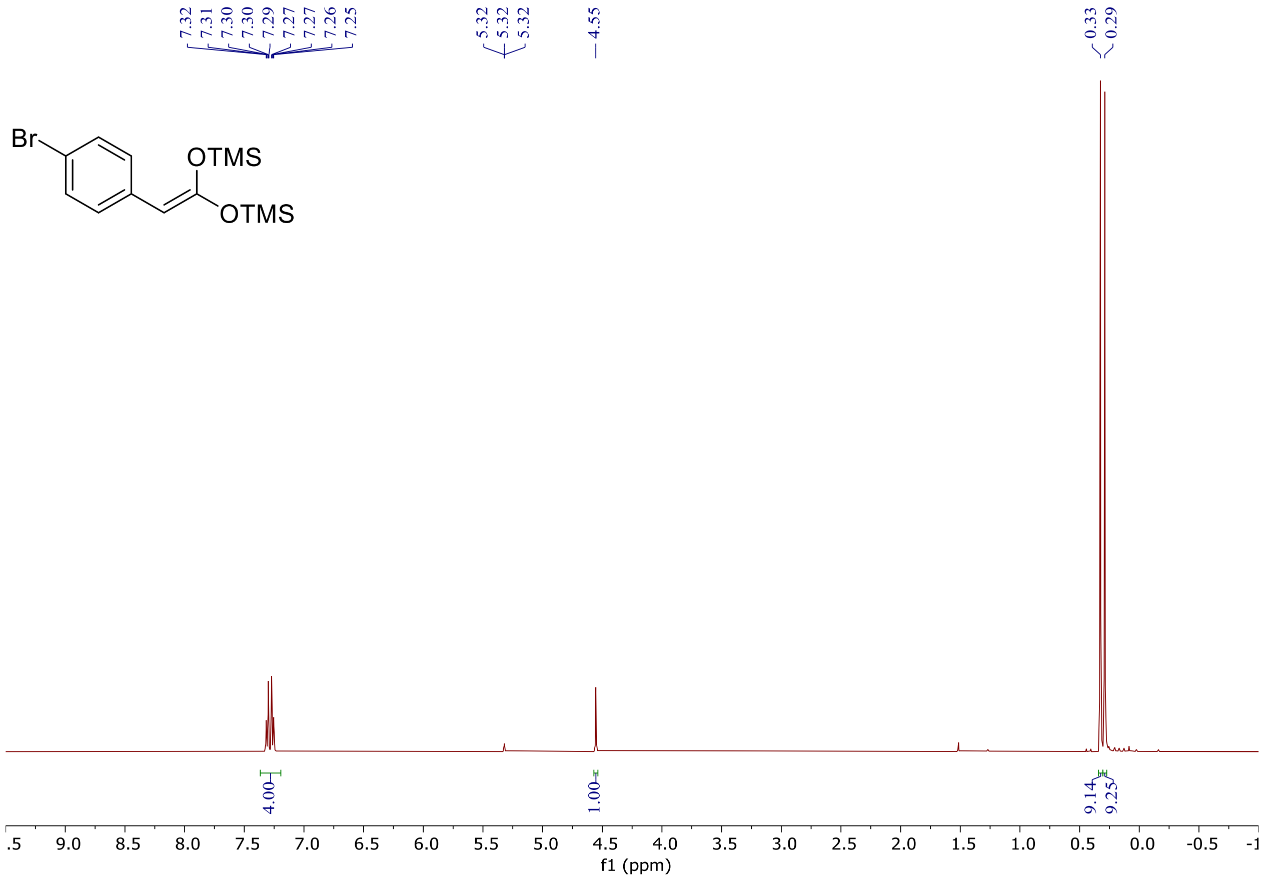
7.32
7.31
7.30
7.30
7.29
7.27
7.27
7.26
7.25
5.32
5.32
5.32
4.55
0.33
0.29
Br
OTMS
OTMS
4.00
1.00
9.14
9.25
9.0 8.5 8.0 7.5 7.0 6.5 6.0 5.5 5.0 4.5 4.0 3.5 3.0 2.5 2.0 1.5 1.0 0.5 0.0 -0.5
f1 (ppm)

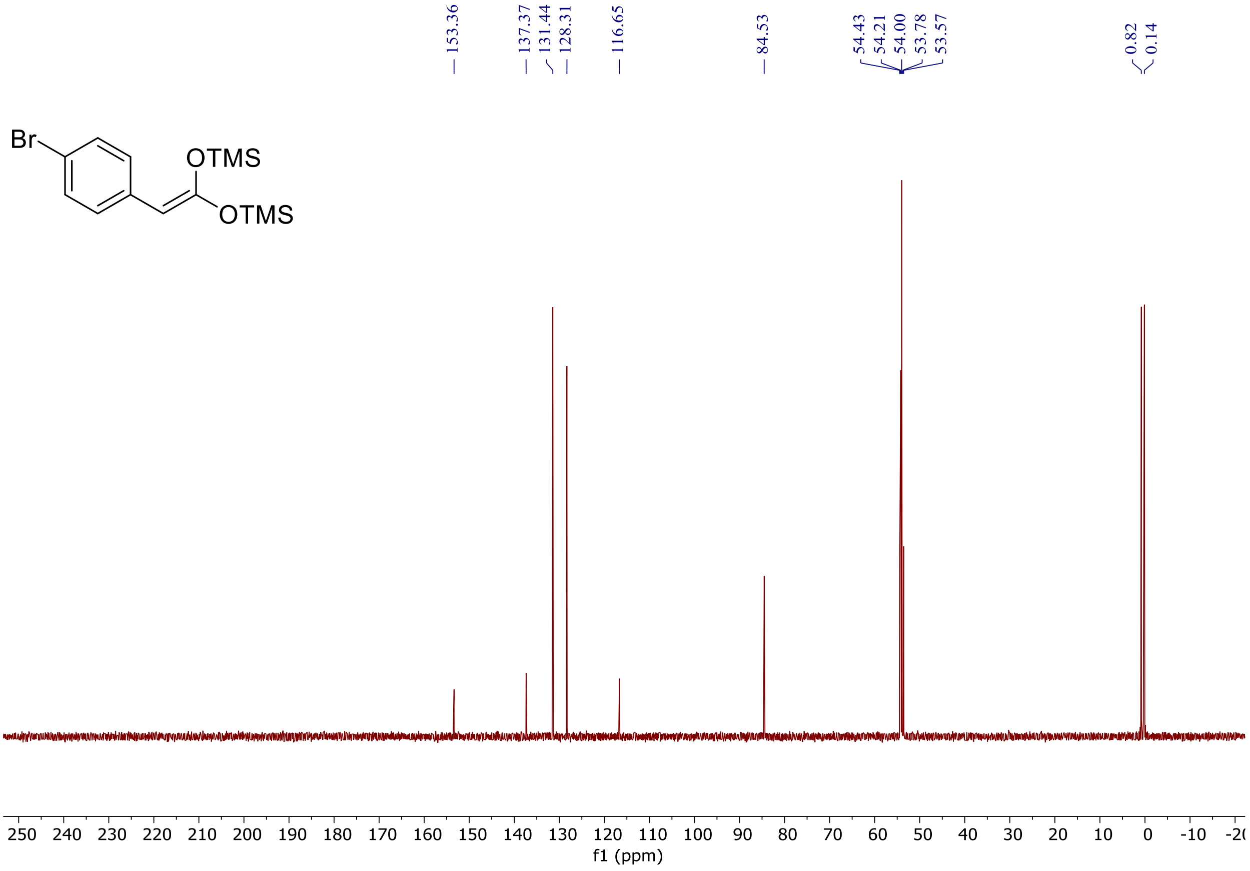
153.36
137.37
131.44
128.31
116.65
84.53
54.43
54.21
54.00
53.78
53.57
0.82
0.14
Br
OTMS
OTMS
250 240 230 220 210 200 190 180 170 160 150 140 130 120 110 100 90 80 70 60 50 40 30 20 10 0 -10
f1 (ppm)

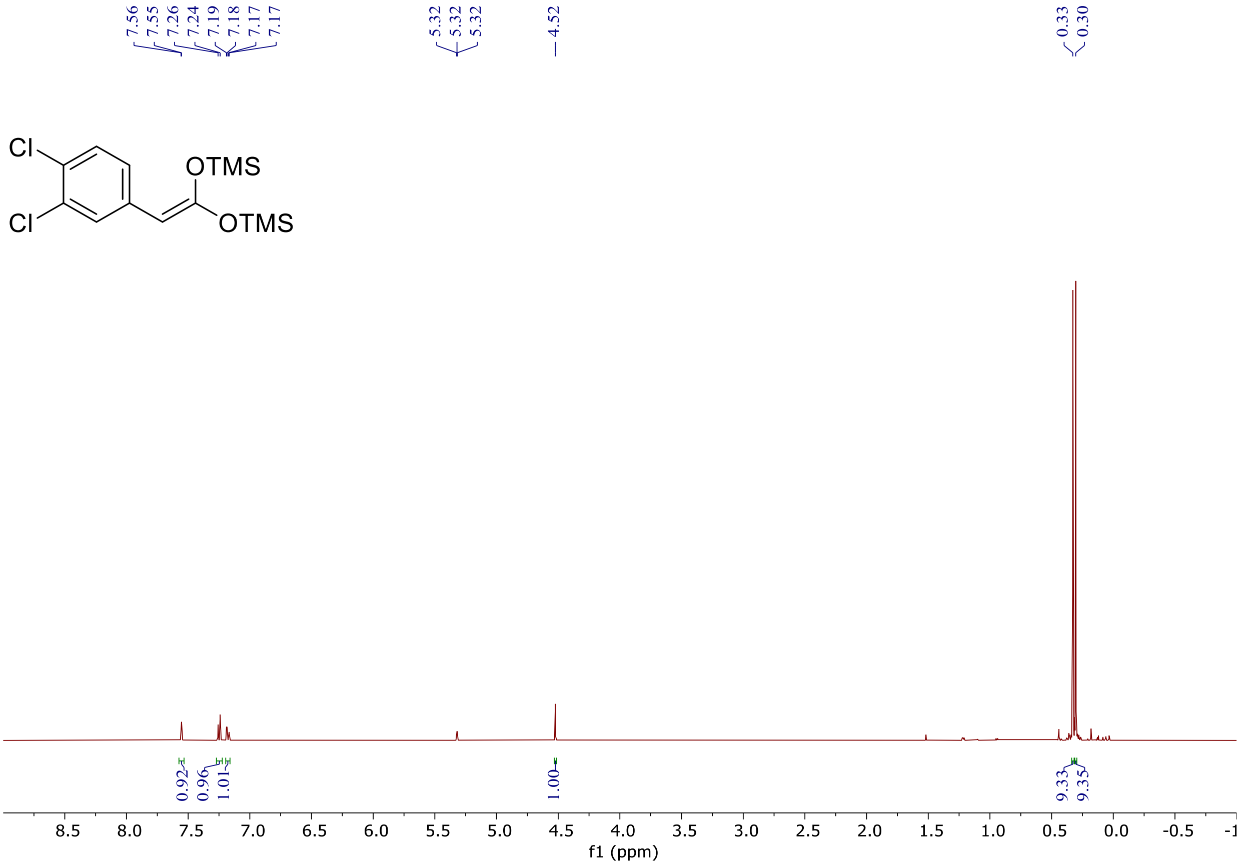
7.56
7.55
7.26
7.24
7.19
7.18
7.17
7.17
5.32
5.32
5.32
4.52
0.33
0.30
Cl
Cl
OTMS
OTMS
0.92
0.96
1.01
1.00
9.33
9.35
8.5 8.0 7.5 7.0 6.5 6.0 5.5 5.0 4.5 4.0 3.5 3.0 2.5 2.0 1.5 1.0 0.5 0.0 -0.5
f1 (ppm)

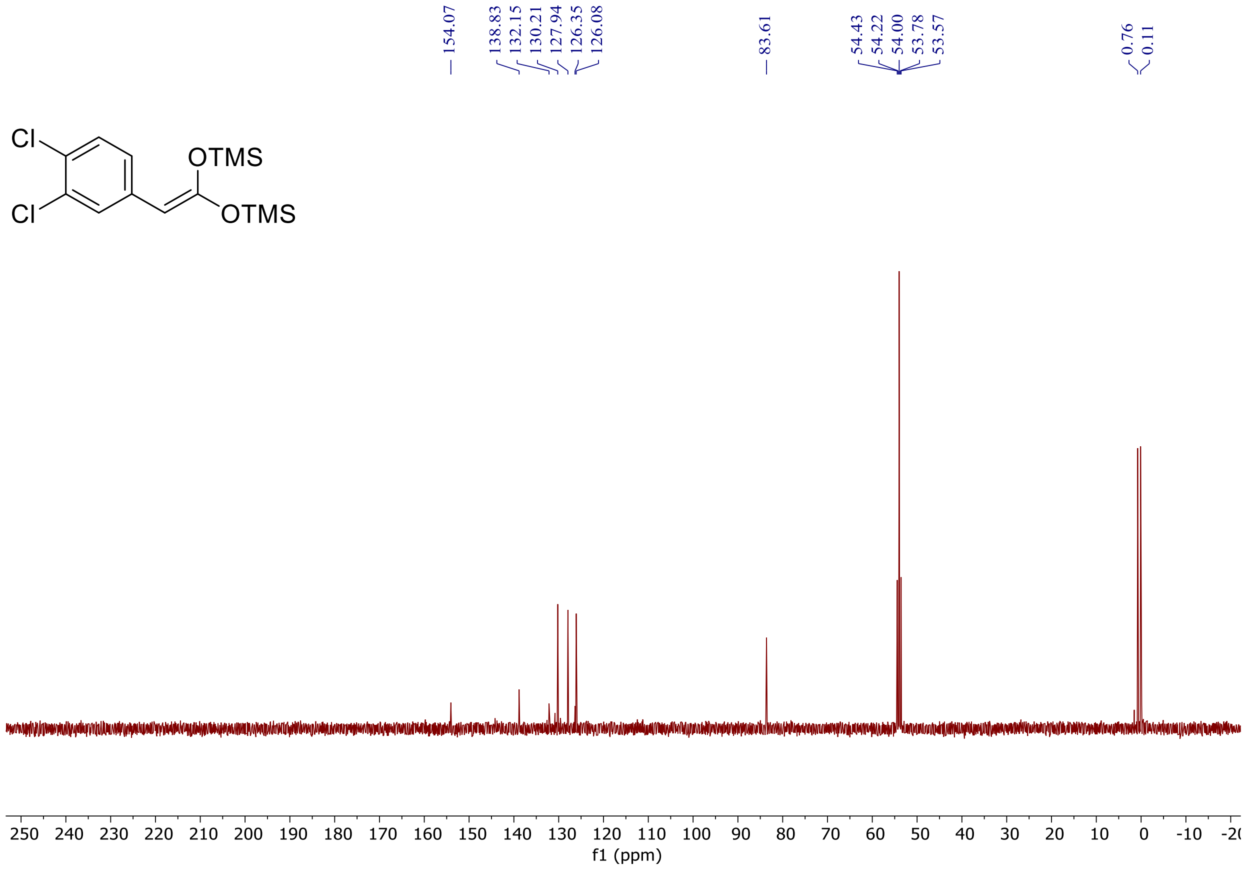

Cl
Cl
OTMS
OTMS
154.07
138.83
132.15
130.21
127.94
126.35
126.08
83.61
54.43
54.22
54.00
53.78
53.57
0.76
0.11
250 240 230 220 210 200 190 180 170 160 150 140 130 120 110 100 90 80 70 60 50 40 30 20 10 0 -10
f1 (ppm)

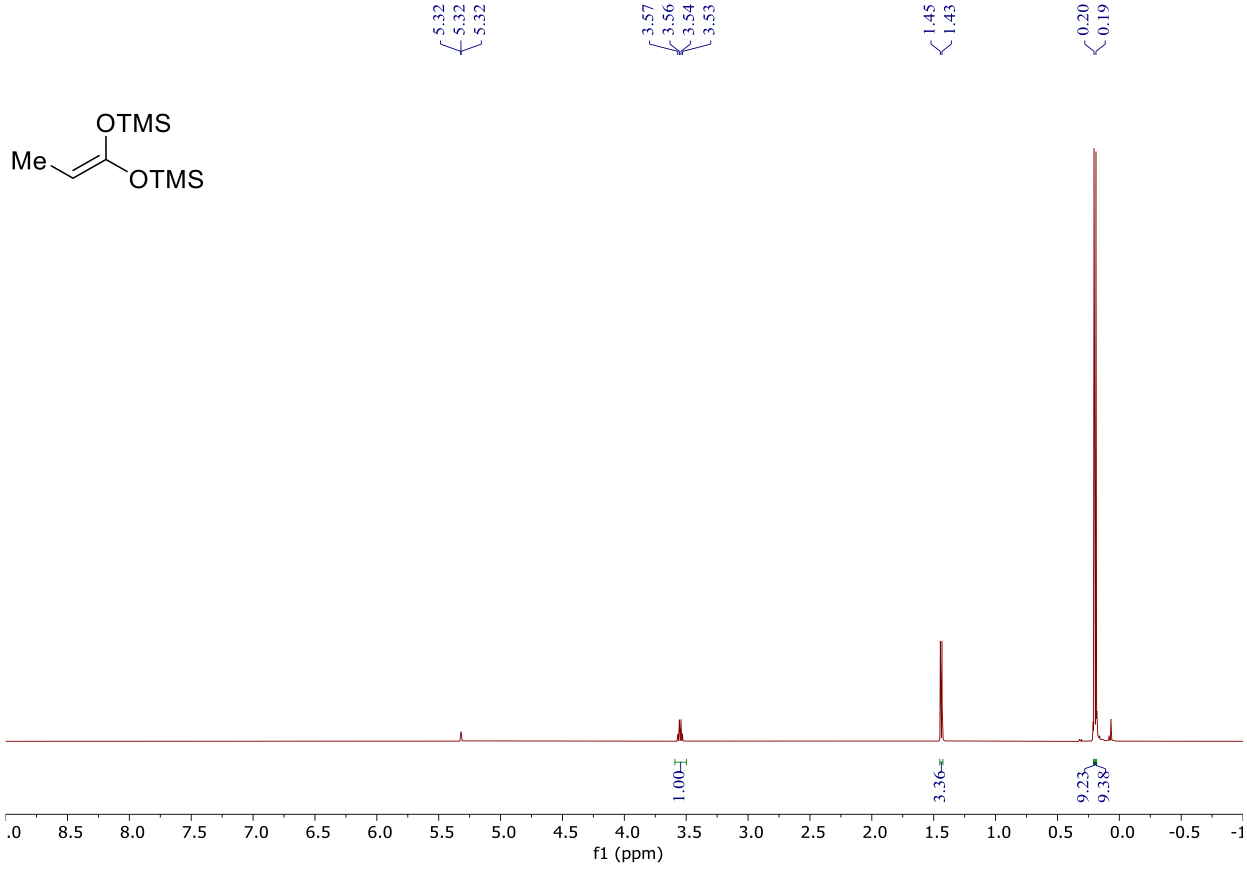

OTMS
Me
OTMS
5.32
5.32
5.32
3.57
3.56
3.54
3.53
1.45
1.43
0.20
0.19
1.00
3.36
9.23
9.38
8.5 8.0 7.5 7.0 6.5 6.0 5.5 5.0 4.5 4.0 3.5 3.0 2.5 2.0 1.5 1.0 0.5 0.0 -0.5
f1 (ppm)

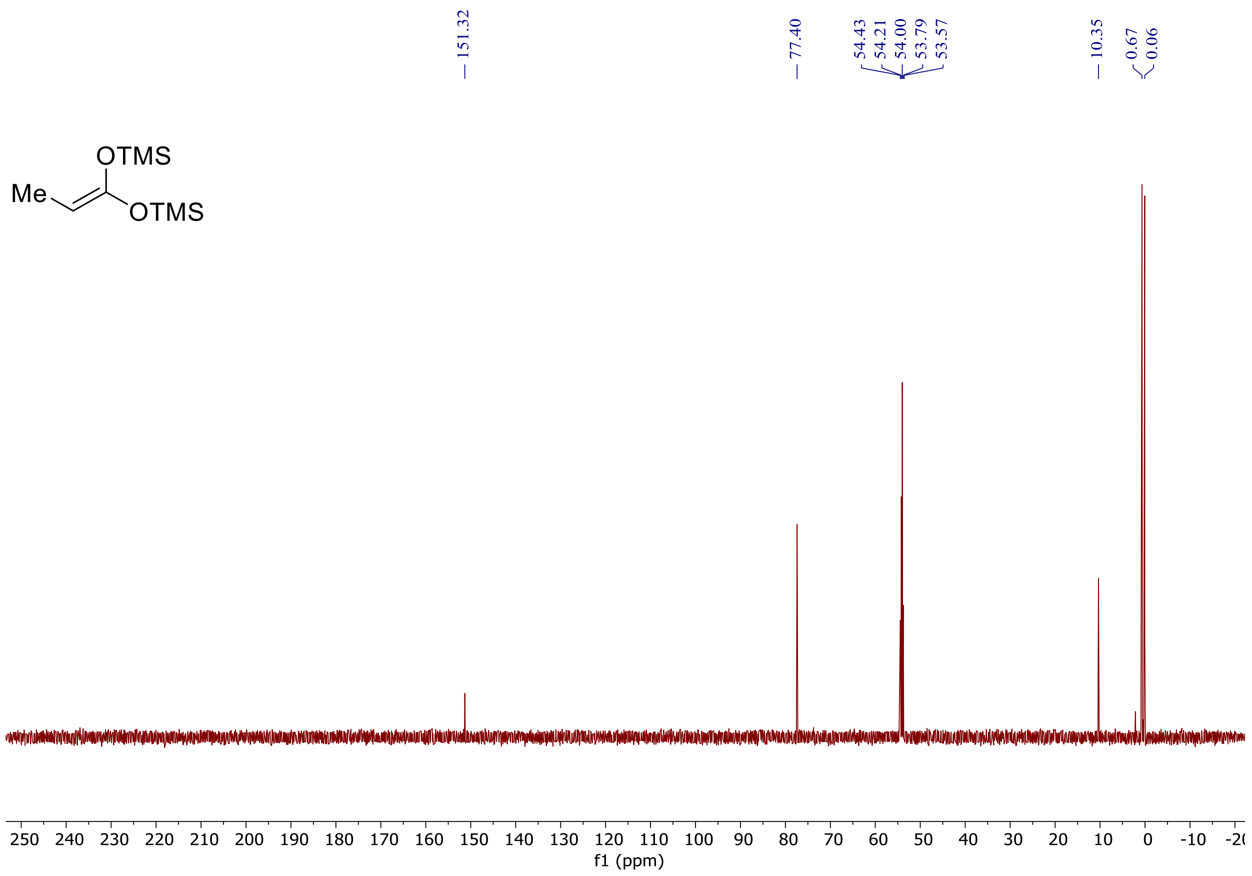

OTMS
Me
OTMS
151.32
77.40
54.43
54.21
54.00
53.79
53.57
10.35
0.67
0.06
f1 (ppm)

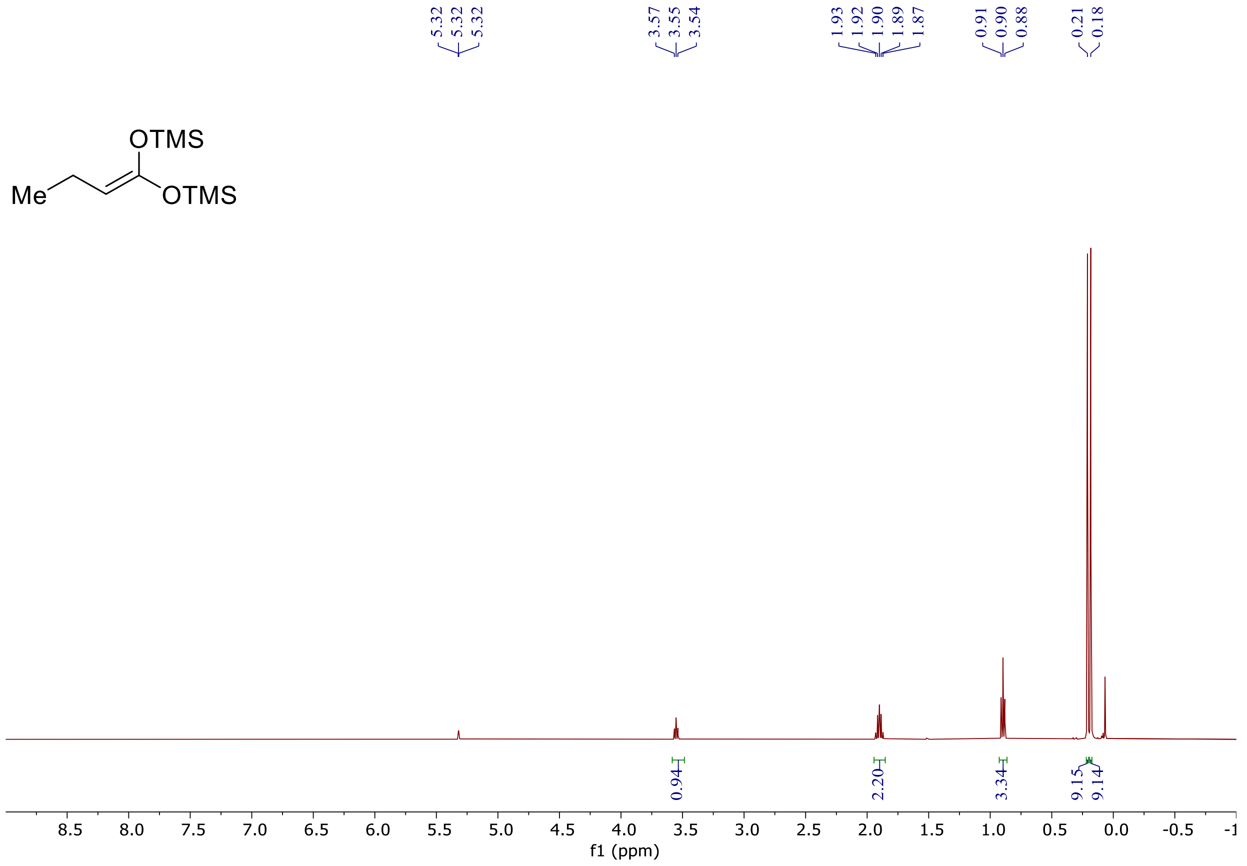

OTMS
Me
OTMS
5.32
5.32
5.32
3.57
3.55
3.54
1.93
1.92
1.90
1.89
1.87
0.91
0.90
0.88
0.21
0.18
0.94
2.20
3.34
9.15
9.14
f1 (ppm)

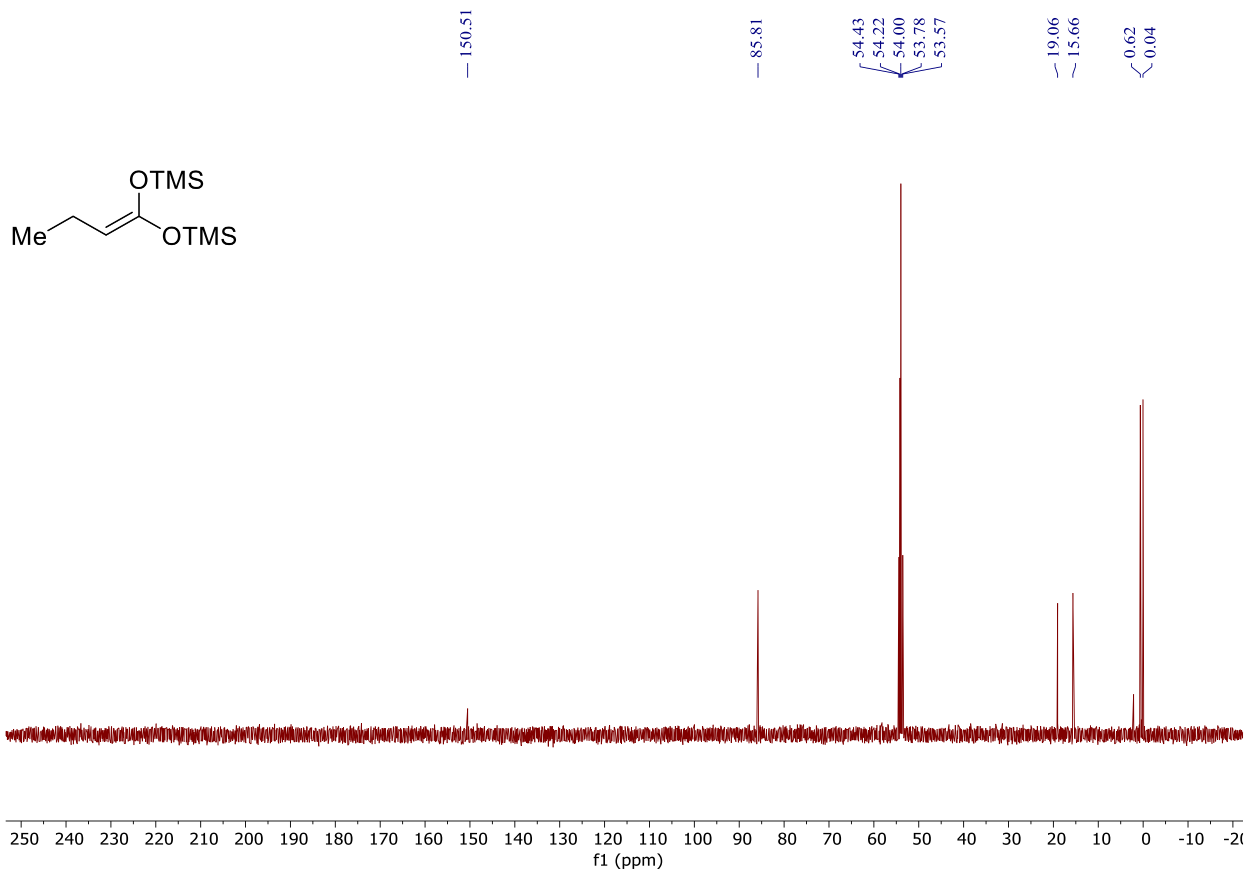
150.51
85.81
54.43
54.22
54.00
53.78
53.57
19.06
15.66
0.62
0.04
OTMS
Me
OTMS
250 240 230 220 210 200 190 180 170 160 150 140 130 120 110 100 90 80 70 60 50 40 30 20 10 0 -10
f1 (ppm)

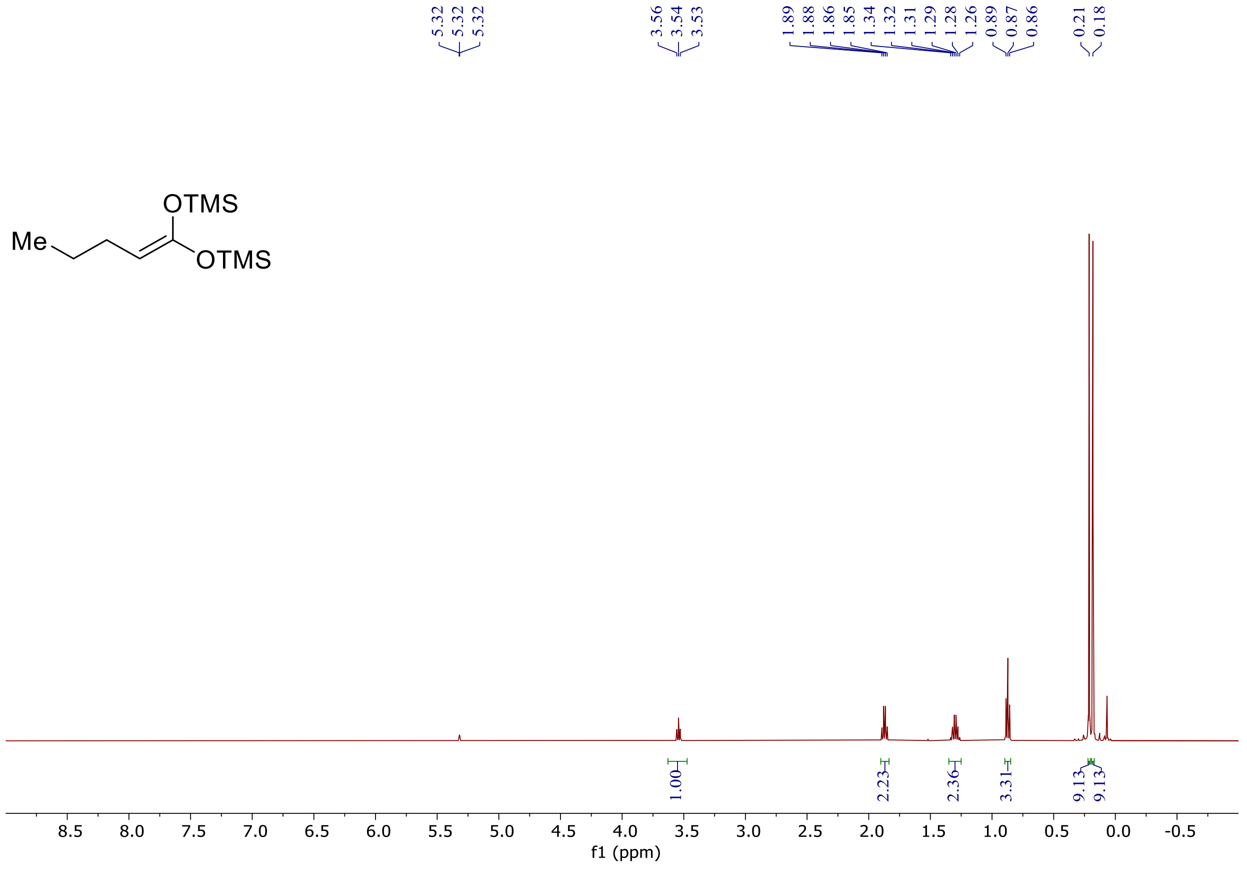
5.32
5.32
5.32
3.56
3.54
3.53
1.89
1.88
1.86
1.85
1.34
1.32
1.31
1.29
1.28
1.26
0.89
0.87
0.86
0.21
0.18
OTMS
Me
OTMS
1.00
2.23
2.36
3.31
9.13
9.13
8.5 8.0 7.5 7.0 6.5 6.0 5.5 5.0 4.5 4.0 3.5 3.0 2.5 2.0 1.5 1.0 0.5 0.0 -0.5
f1 (ppm)

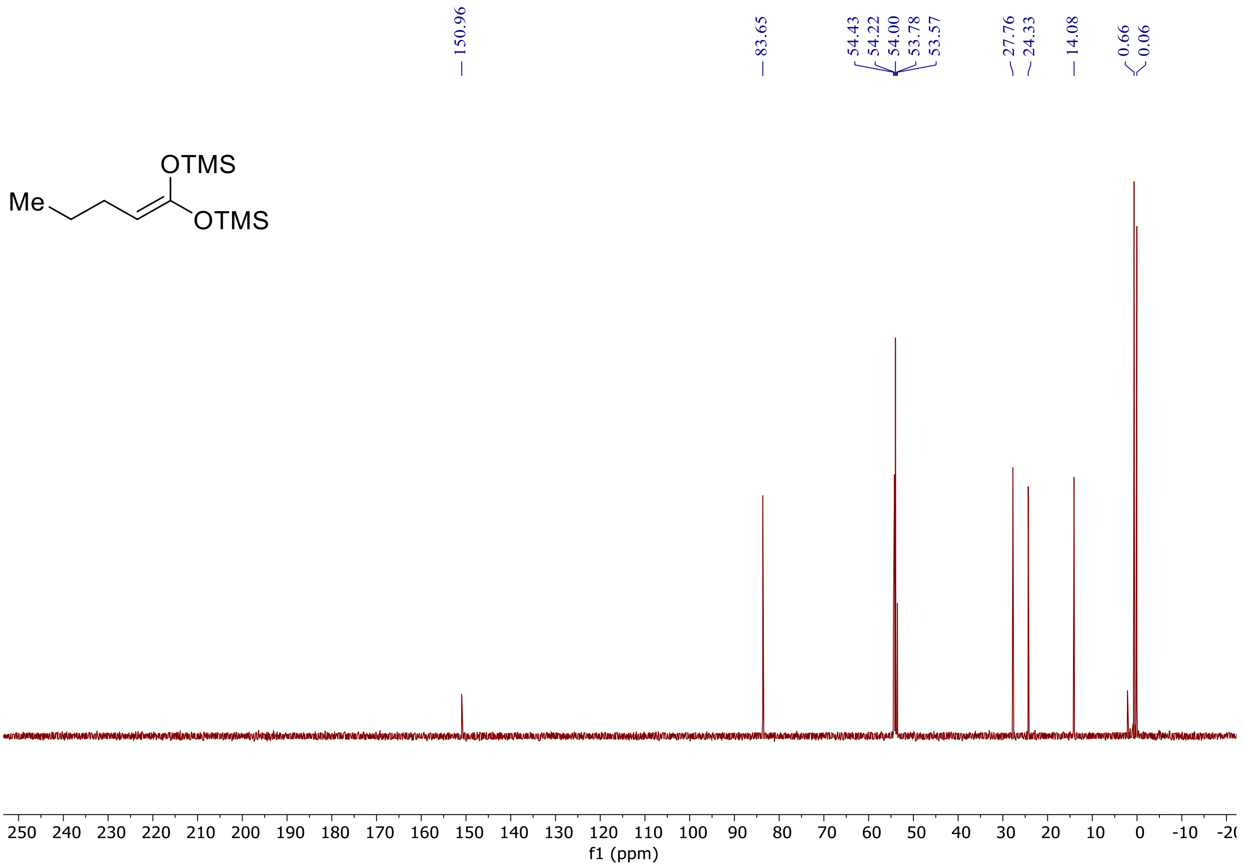

150.96
83.65
54.43
54.22
54.00
53.78
53.57
27.76
24.33
14.08
0.66
0.06
OTMS
Me
OTMS
250 240 230 220 210 200 190 180 170 160 150 140 130 120 110 100 90 80 70 60 50 40 30 20 10 0 -10
f1 (ppm)

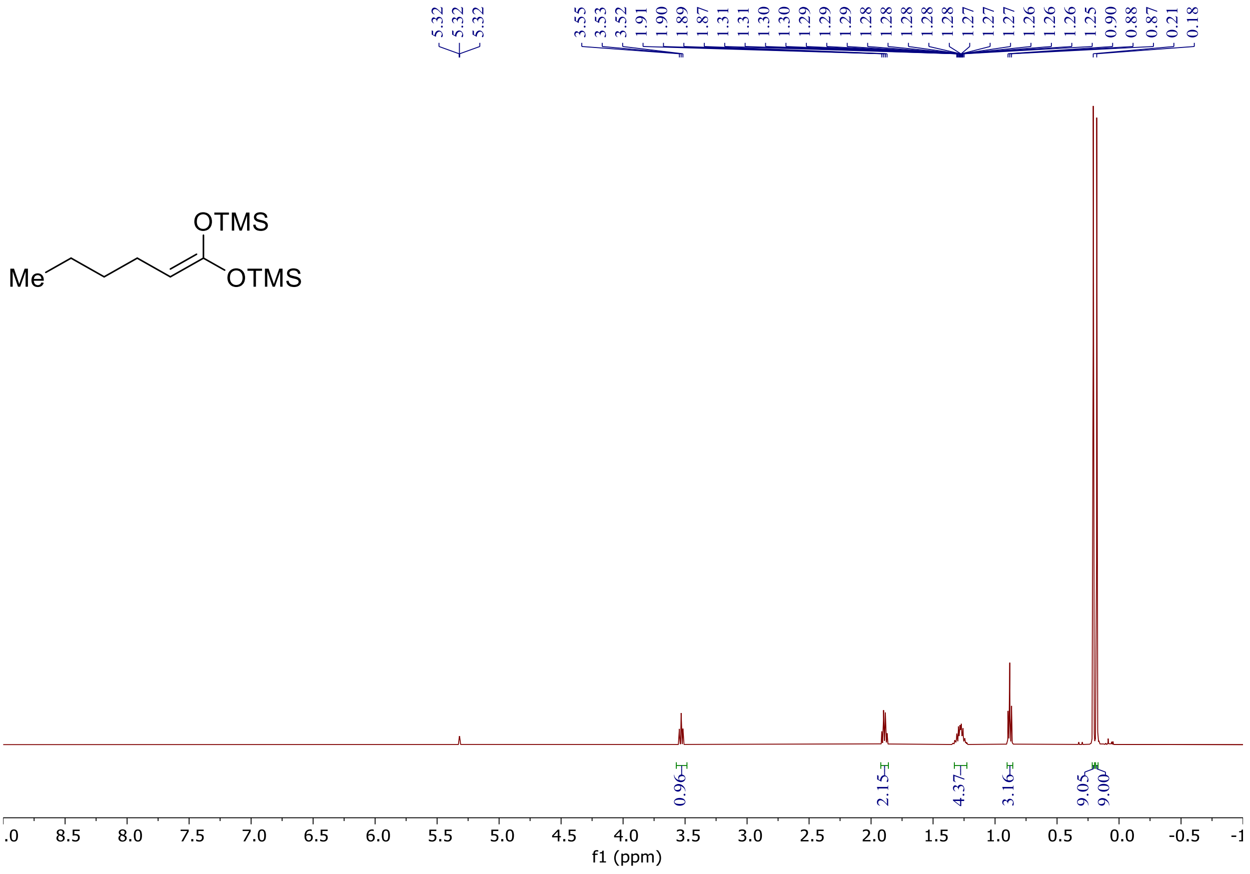

5.32
5.32
5.32
3.55
3.53
3.52
1.91
1.90
1.89
1.87
1.31
1.31
1.30
1.30
1.29
1.29
1.29
1.28
1.28
1.28
1.28
1.28
1.27
1.27
1.27
1.26
1.26
1.26
1.25
0.90
0.88
0.87
0.21
0.18
OTMS
Me
OTMS
0.96
2.15
4.37
3.16
9.05
9.00
8.5 8.0 7.5 7.0 6.5 6.0 5.5 5.0 4.5 4.0 3.5 3.0 2.5 2.0 1.5 1.0 0.5 0.0 -0.5
f1 (ppm)

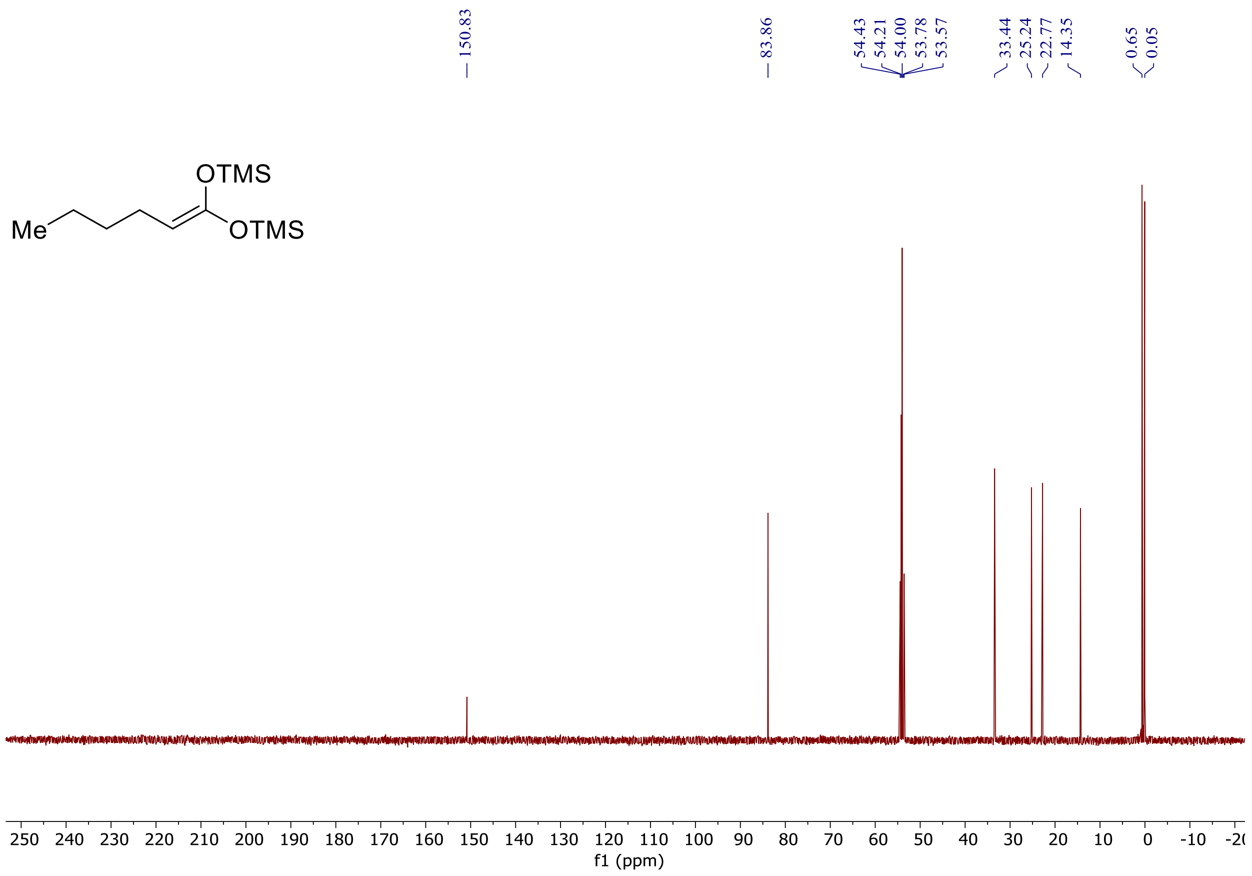
150.83
83.86
54.43
54.21
54.00
53.78
53.57
33.44
25.24
22.77
14.35
0.65
0.05
OTMS
Me
OTMS
250 240 230 220 210 200 190 180 170 160 150 140 130 120 110 100 90 80 70 60 50 40 30 20 10 0 -10
f1 (ppm)

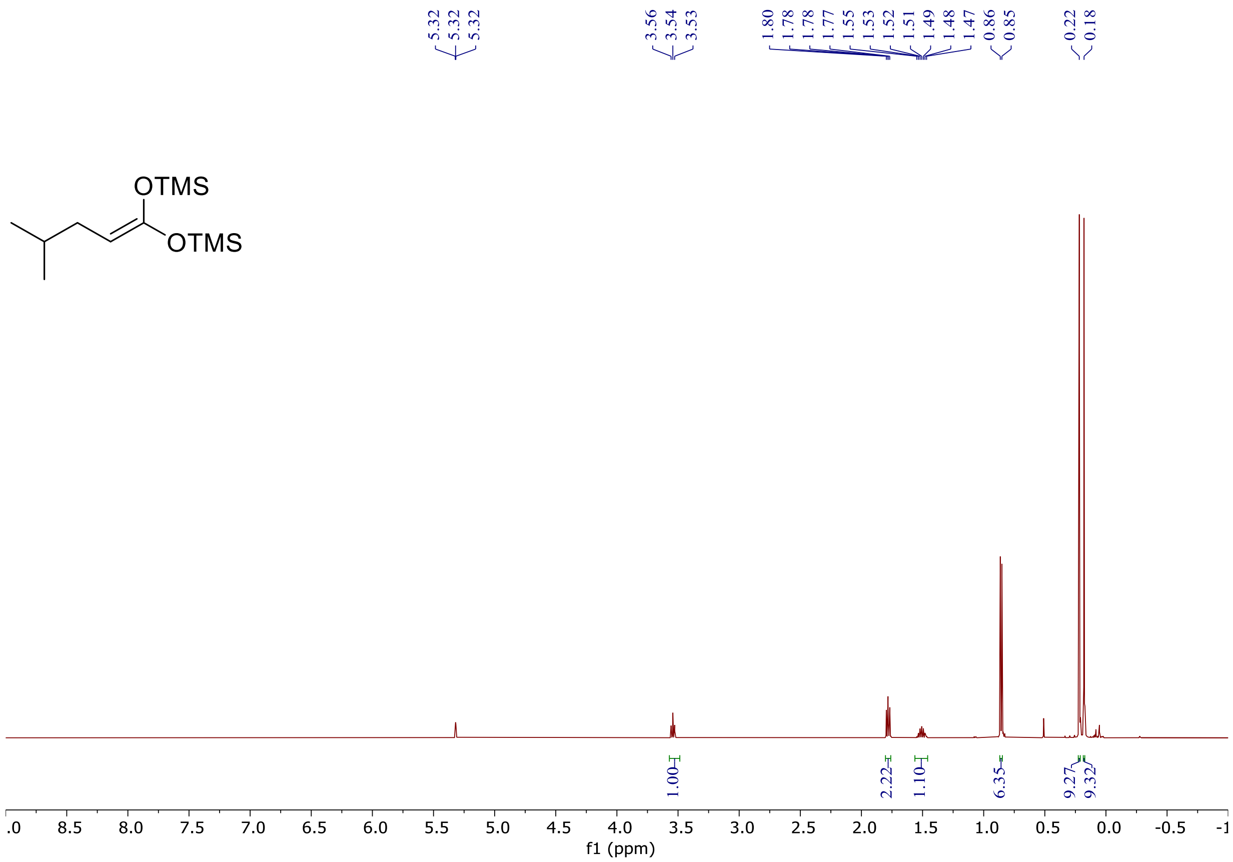
5.32
5.32
5.32
3.56
3.54
3.53
1.80
1.78
1.78
1.77
1.55
1.53
1.52
1.51
1.49
1.48
1.47
0.86
0.85
0.22
0.18
OTMS
OTMS
1.00
2.22
1.10
6.35
9.27
9.32
8.5 8.0 7.5 7.0 6.5 6.0 5.5 5.0 4.5 4.0 3.5 3.0 2.5 2.0 1.5 1.0 0.5 0.0 -0.5
f1 (ppm)

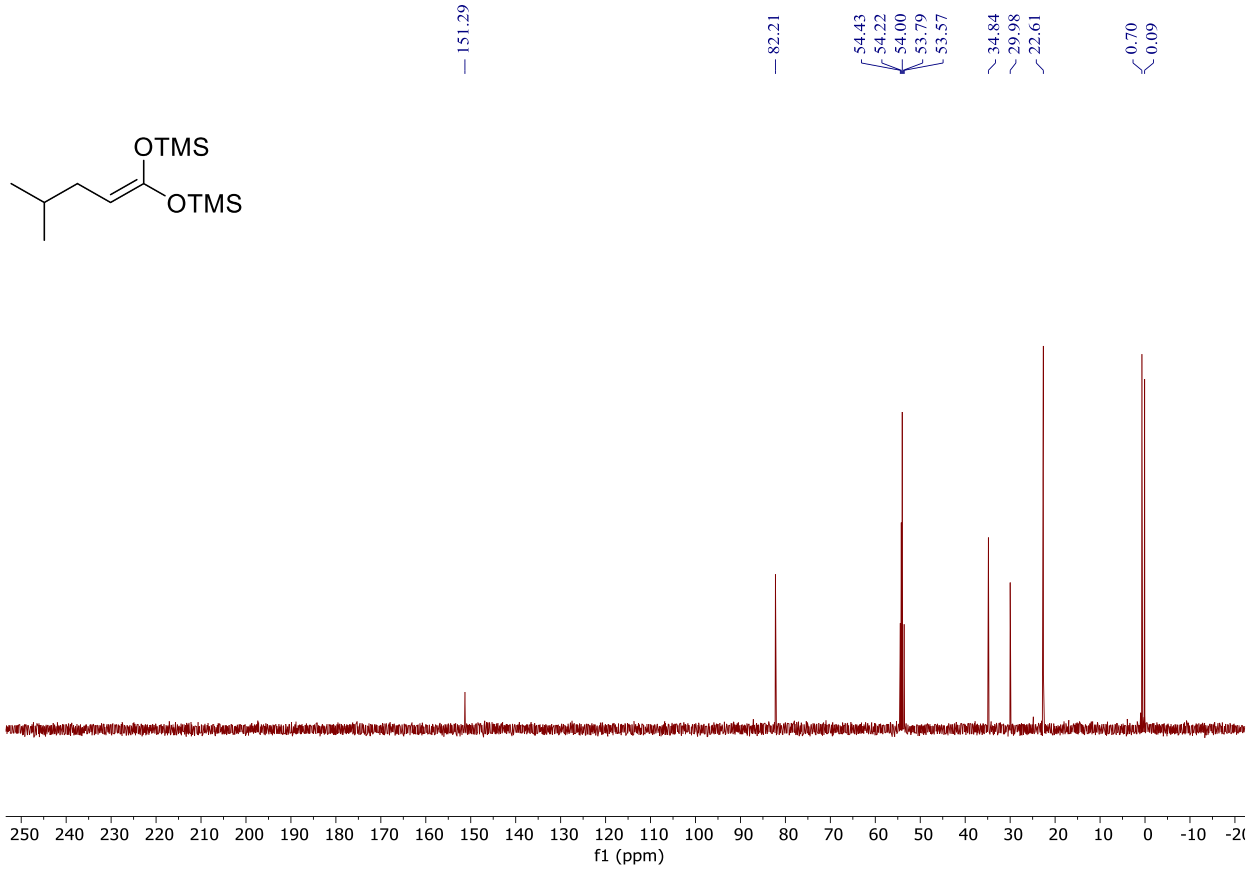
151.29
82.21
54.43
54.22
54.00
53.79
53.57
34.84
29.98
22.61
0.70
0.09
OTMS
OTMS
f1 (ppm)

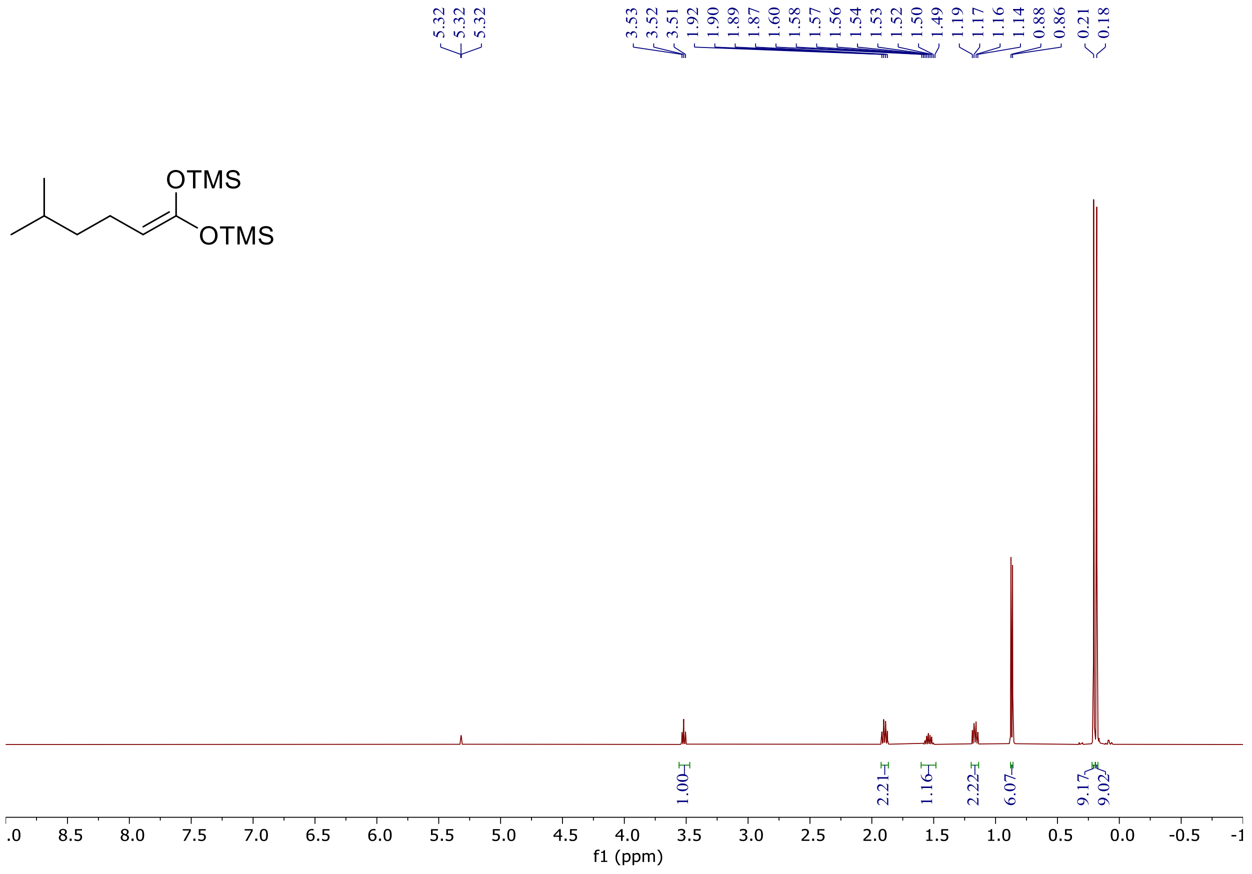
5.32
5.32
5.32
3.53
3.52
3.51
1.92
1.90
1.89
1.87
1.60
1.58
1.57
1.56
1.54
1.53
1.52
1.50
1.49
1.19
1.17
1.16
1.14
0.88
0.86
0.21
0.18
OTMS
OTMS
1.00
2.21
1.16
2.22
6.07
9.17
9.02
f1 (ppm)

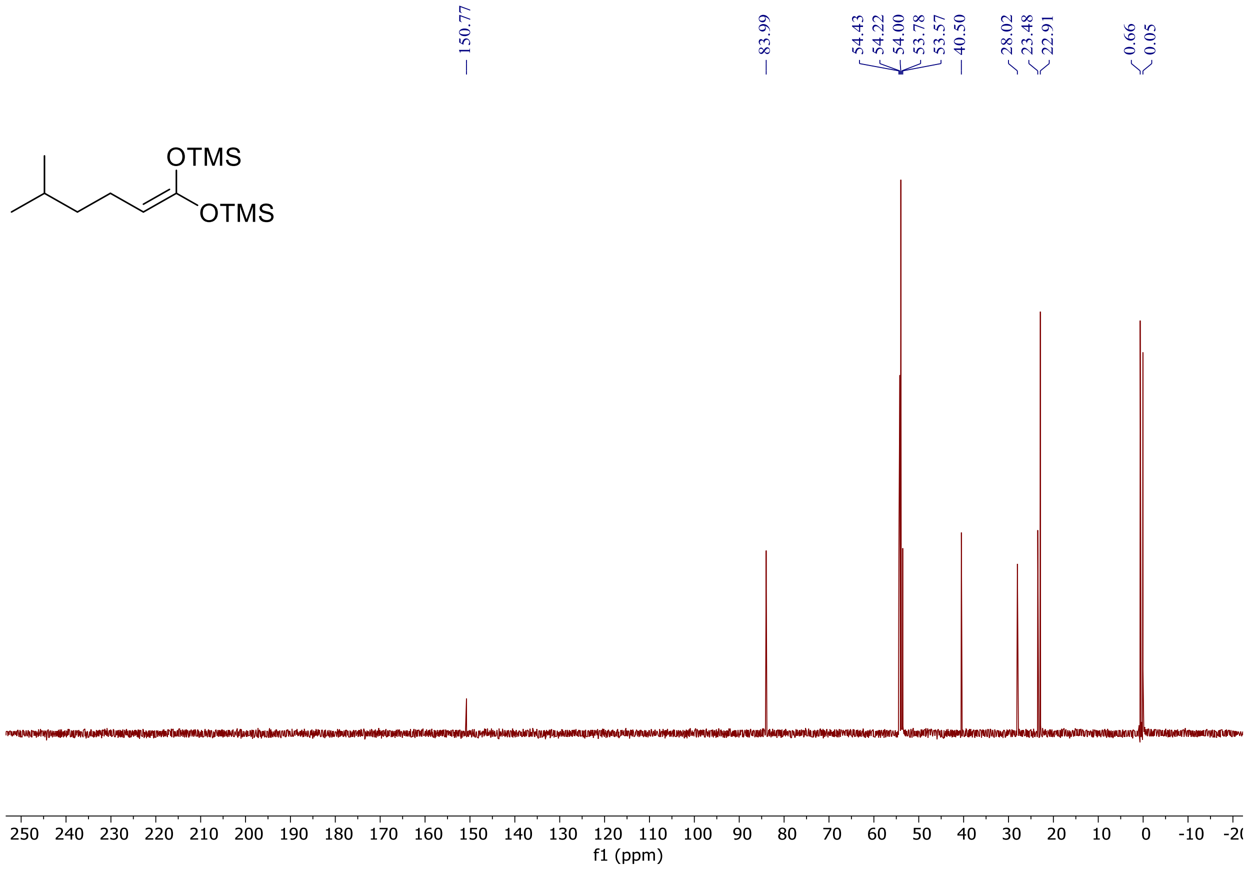
150.77
83.99
54.43
54.22
54.00
53.78
53.57
40.50
28.02
23.48
22.91
0.66
0.05
OTMS
OTMS
250 240 230 220 210 200 190 180 170 160 150 140 130 120 110 100 90 80 70 60 50 40 30 20 10 0 -10
f1 (ppm)

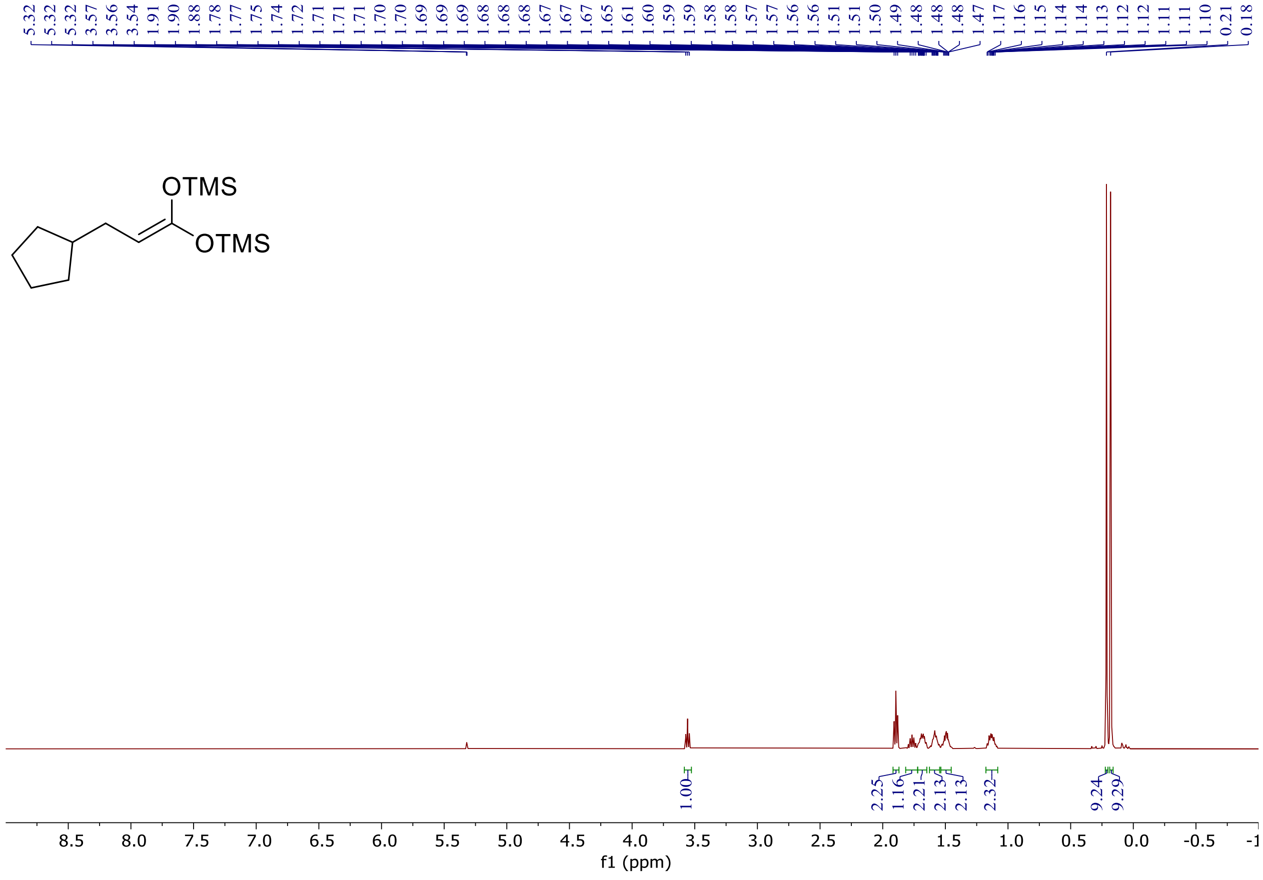
5.32 5.32 5.32 3.57 3.56 3.54 1.91 1.90 1.88 1.78 1.77 1.75 1.74 1.72 1.71 1.71 1.71 1.70 1.70 1.69 1.69 1.68 1.68 1.68 1.67 1.67 1.67 1.65 1.61 1.60 1.59 1.59 1.58 1.58 1.57 1.57 1.56 1.56 1.51 1.51 1.50 1.49 1.48 1.48 1.48 1.47 1.17 1.16 1.15 1.14 1.14 1.13 1.13 1.12 1.12 1.11 1.11 1.10 0.21 0.18
OTMS
OTMS
1.00
2.25
1.16
2.21
2.13
2.13
2.32
9.24
9.29
8.5 8.0 7.5 7.0 6.5 6.0 5.5 5.0 4.5 4.0 3.5 3.0 2.5 2.0 1.5 1.0 0.5 0.0 -0.5
f1 (ppm)

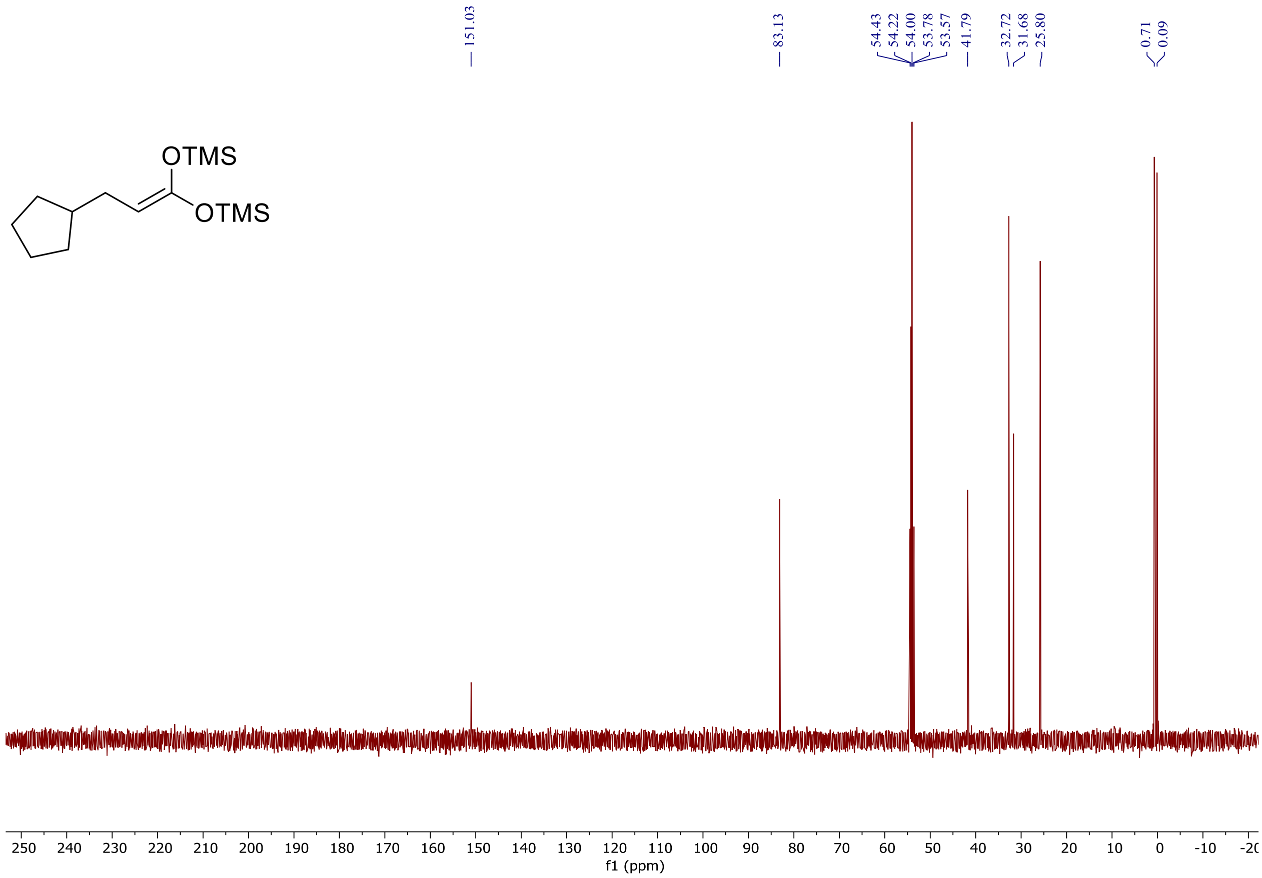
151.03
83.13
54.43
54.22
54.00
53.78
53.57
41.79
32.72
31.68
25.80
0.71
0.09
OTMS
OTMS
250 240 230 220 210 200 190 180 170 160 150 140 130 120 110 100 90 80 70 60 50 40 30 20 10 0 -10 -20
f1 (ppm)

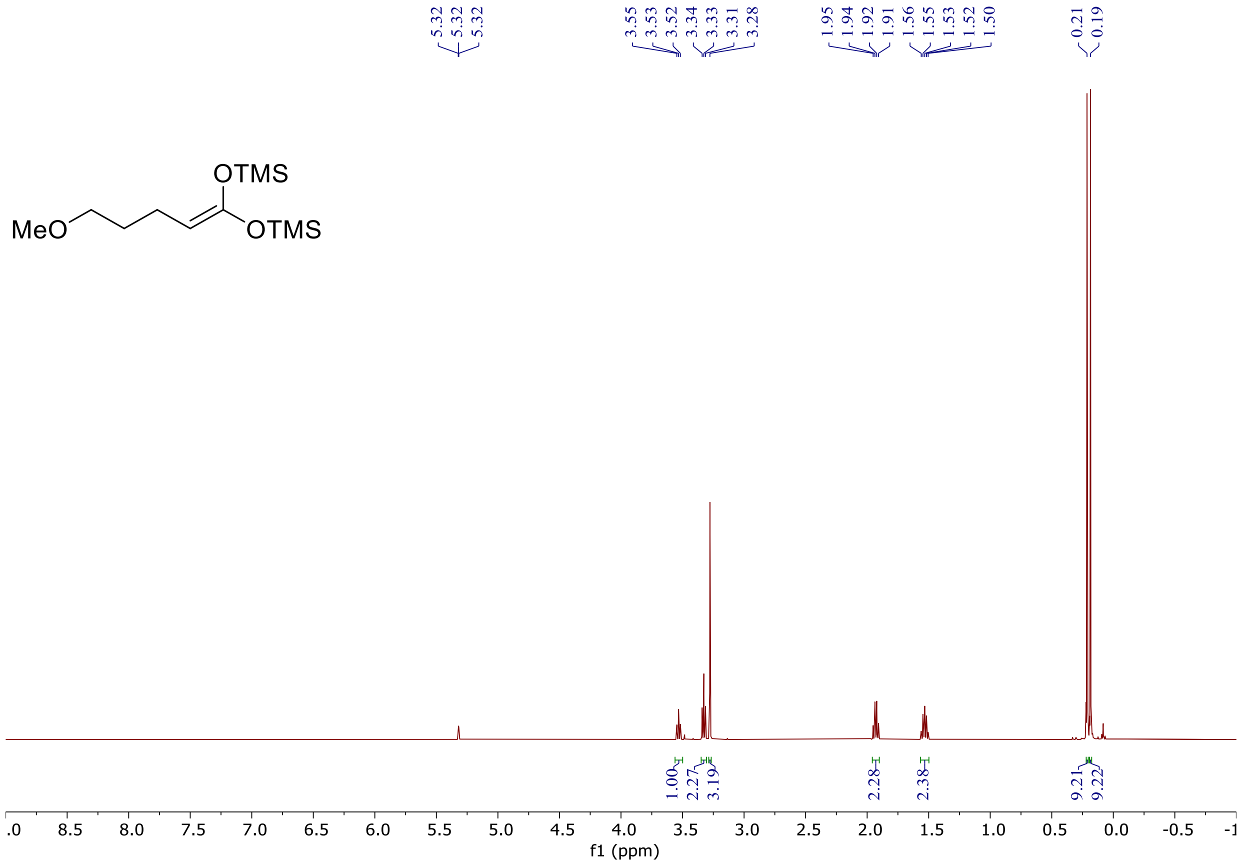
5.32
5.32
5.32
3.55
3.53
3.52
3.34
3.33
3.31
3.28
1.95
1.94
1.92
1.91
1.56
1.55
1.53
1.52
1.50
0.21
0.19
OTMS
MeO
OTMS
1.00
2.27
3.19
2.28
2.38
9.21
9.22
9.0 8.5 8.0 7.5 7.0 6.5 6.0 5.5 5.0 4.5 4.0 3.5 3.0 2.5 2.0 1.5 1.0 0.5 0.0 -0.5 -1
f1 (ppm)

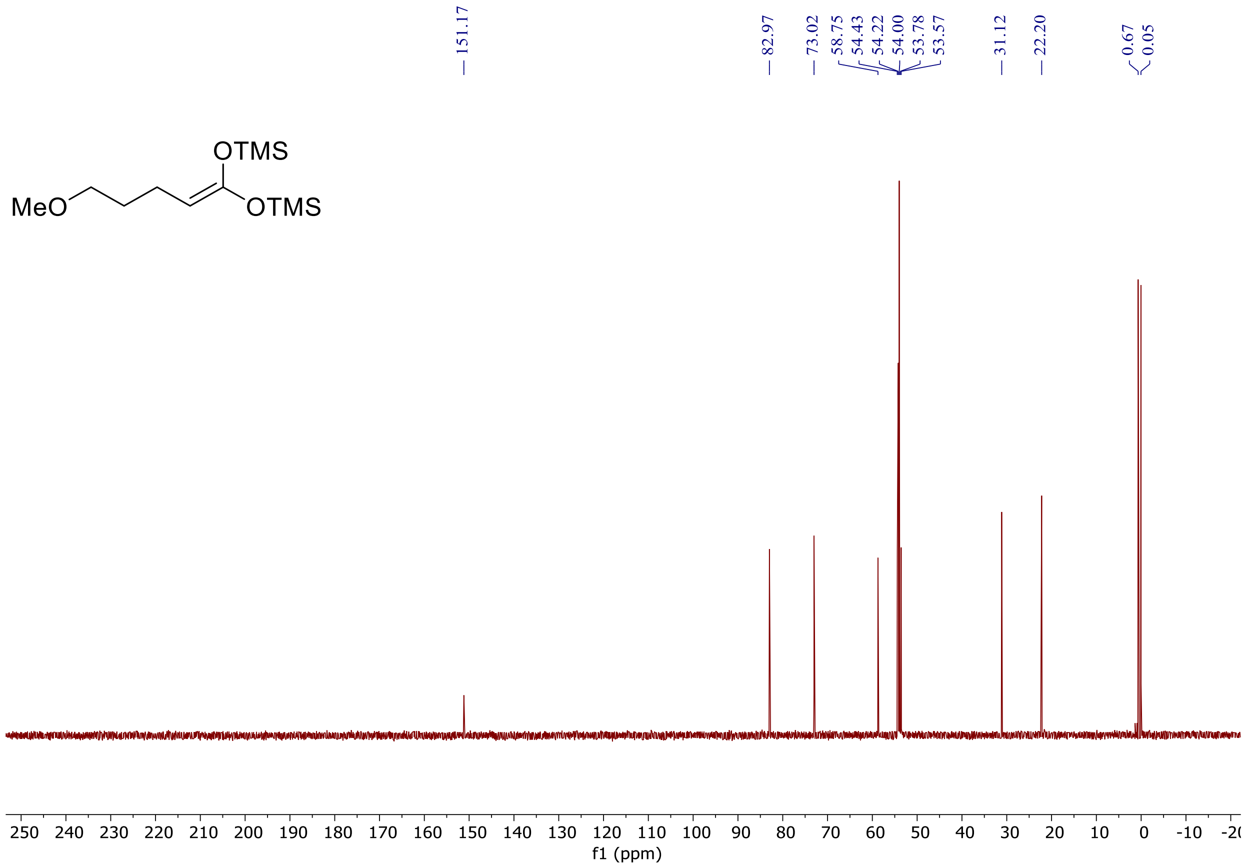
151.17
82.97
73.02
58.75
54.43
54.22
54.00
53.78
53.57
31.12
22.20
0.67
0.05
OTMS
MeO
OTMS
250 240 230 220 210 200 190 180 170 160 150 140 130 120 110 100 90 80 70 60 50 40 30 20 10 0 -10 -2(
f1 (ppm)

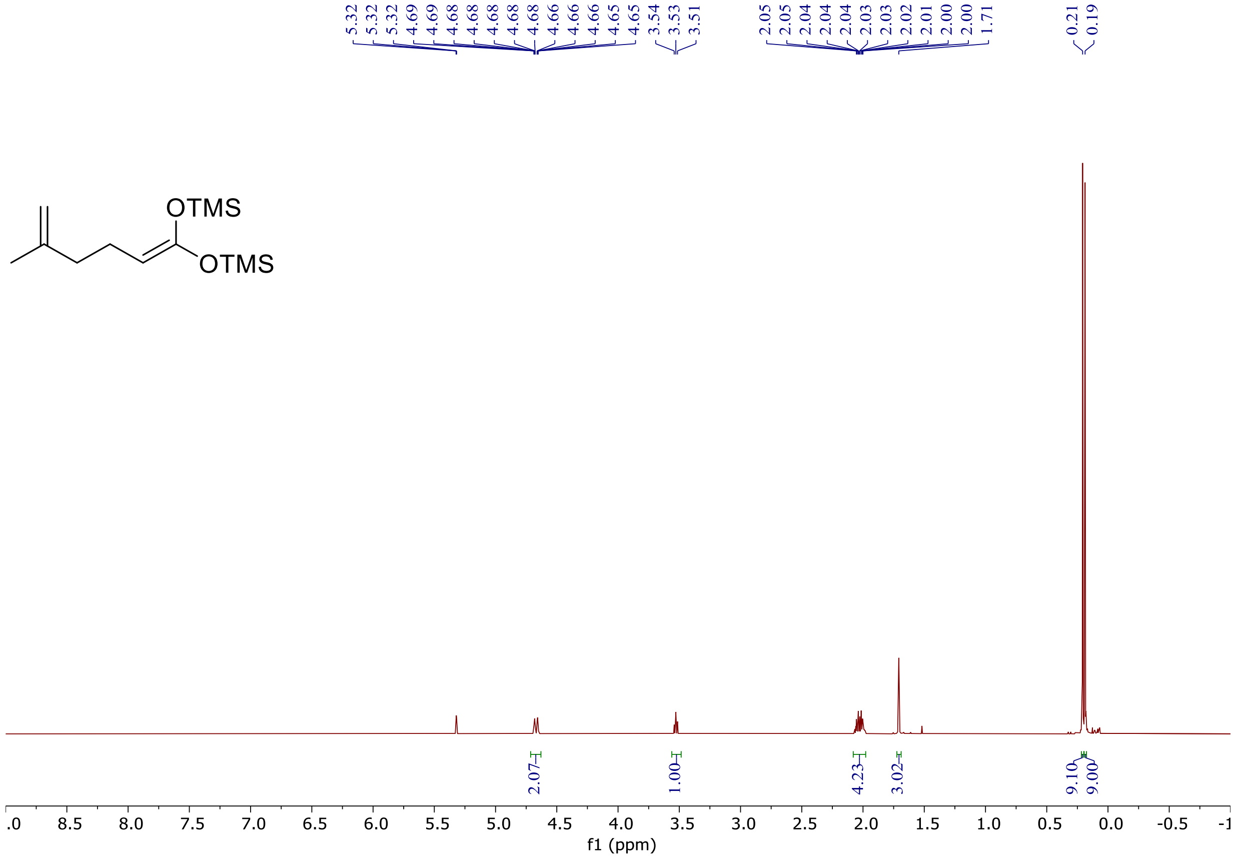
5.32
5.32
5.32
4.69
4.68
4.68
4.68
4.68
4.68
4.66
4.66
4.66
4.65
4.65
3.54
3.53
3.51
2.05
2.05
2.04
2.04
2.04
2.03
2.03
2.02
2.01
2.00
2.00
1.71
0.21
0.19
OTMS
OTMS
2.07
1.00
4.23
3.02
9.10
9.00
.0 8.5 8.0 7.5 7.0 6.5 6.0 5.5 5.0 4.5 4.0 3.5 3.0 2.5 2.0 1.5 1.0 0.5 0.0 -0.5 -1
f1 (ppm)

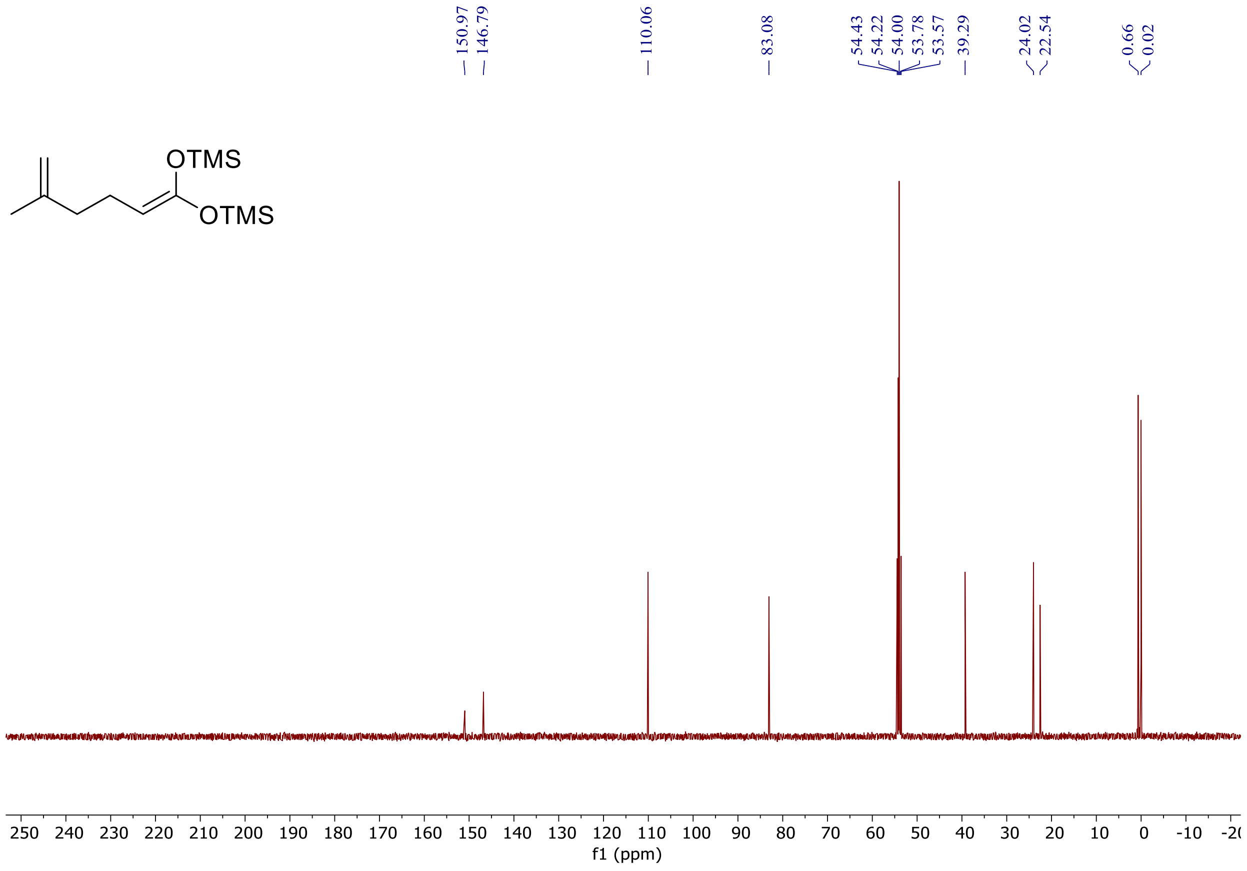
150.97
146.79
110.06
83.08
54.43
54.22
54.00
53.78
53.57
39.29
24.02
22.54
0.66
0.02
OTMS
OTMS
f1 (ppm)

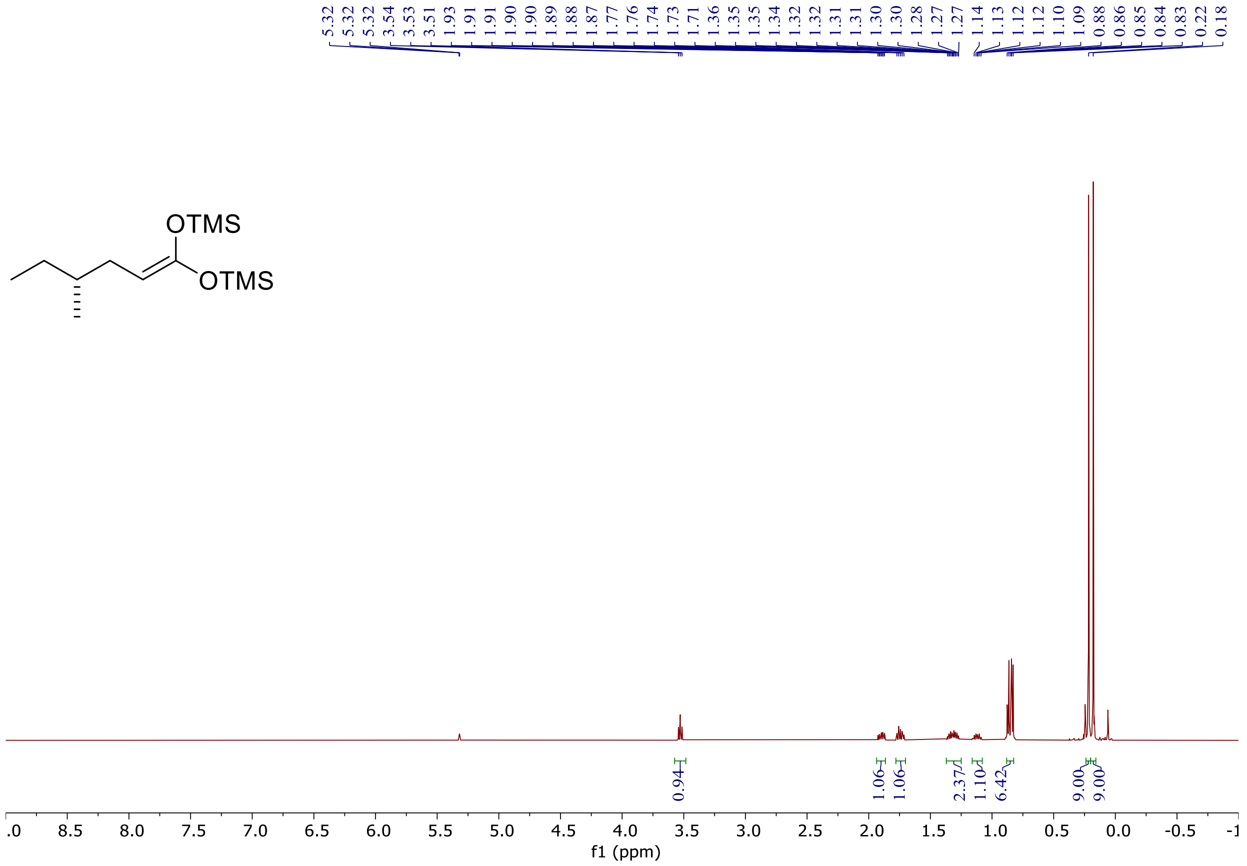
5.32
5.32
5.32
3.54
3.53
3.51
1.93
1.91
1.91
1.90
1.90
1.89
1.88
1.87
1.77
1.76
1.74
1.73
1.71
1.36
1.35
1.35
1.34
1.32
1.32
1.31
1.31
1.30
1.30
1.28
1.27
1.27
1.14
1.13
1.12
1.12
1.10
1.09
0.88
0.86
0.85
0.84
0.83
0.22
0.18
OTMS
OTMS
0.94
1.06
1.06
2.37
1.10
6.42
9.00
9.00
f1 (ppm)

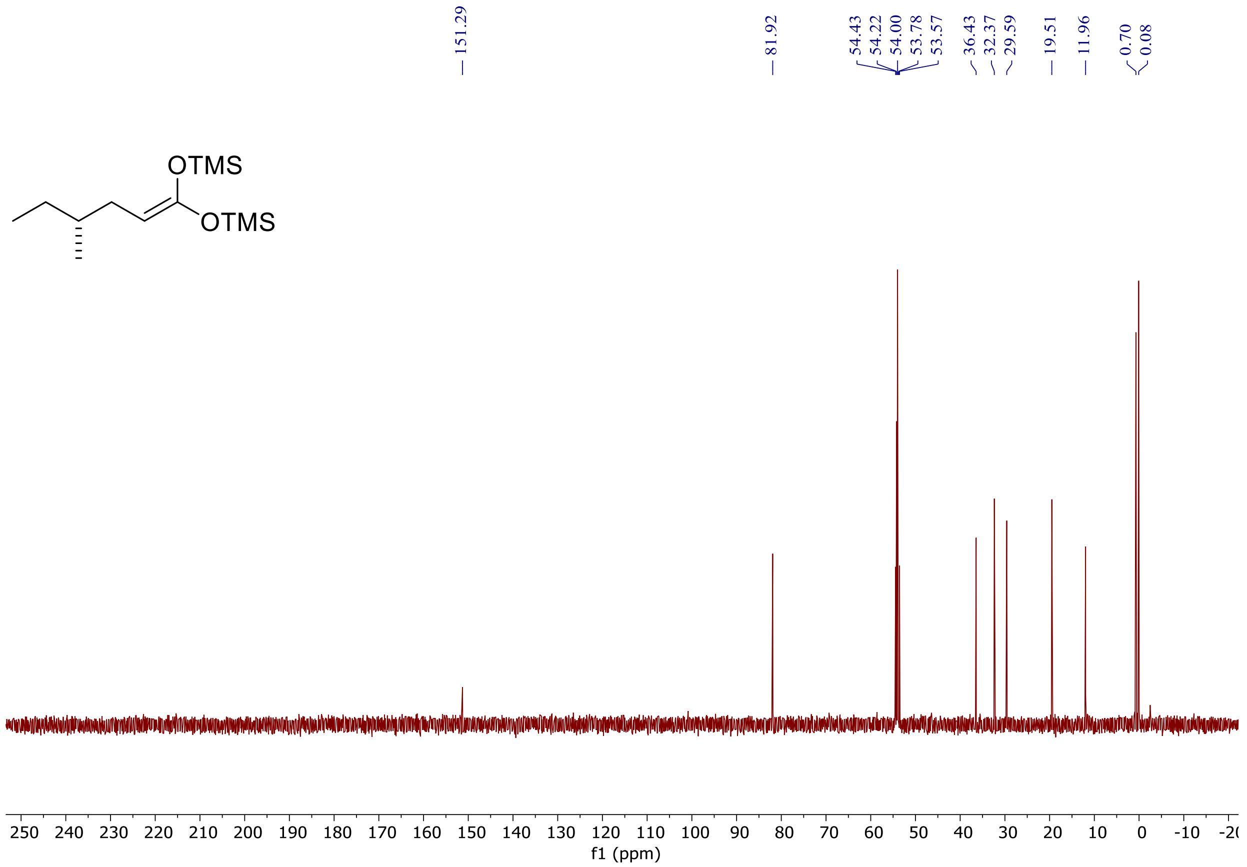
151.29
81.92
54.43
54.22
54.00
53.78
53.57
36.43
32.37
29.59
19.51
11.96
0.70
0.08
OTMS
OTMS
250 240 230 220 210 200 190 180 170 160 150 140 130 120 110 100 90 80 70 60 50 40 30 20 10 0 -10
f1 (ppm)

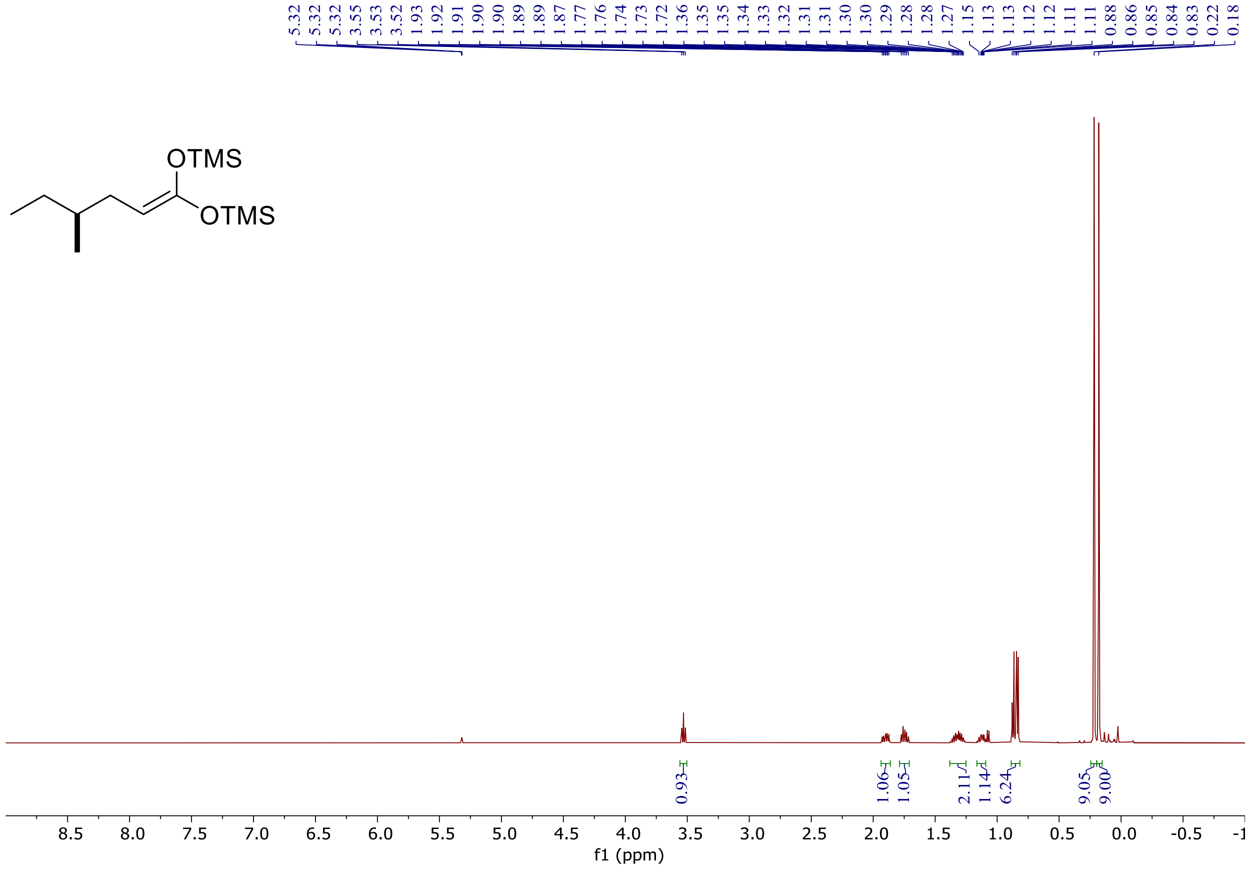
5.32 5.32 5.32 3.55 3.53 3.52 1.93 1.92 1.91 1.90 1.90 1.89 1.89 1.87 1.77 1.76 1.74 1.73 1.72 1.36 1.35 1.35 1.34 1.33 1.32 1.31 1.31 1.30 1.29 1.28 1.28 1.27 1.15 1.13 1.13 1.12 1.12 1.11 1.11 0.88 0.86 0.85 0.84 0.83 0.22 0.18
OTMS
OTMS
0.93
1.06
1.05
2.11
1.14
6.24
9.05
9.00
8.5 8.0 7.5 7.0 6.5 6.0 5.5 5.0 4.5 4.0 3.5 3.0 2.5 2.0 1.5 1.0 0.5 0.0 -0.5
f1 (ppm)

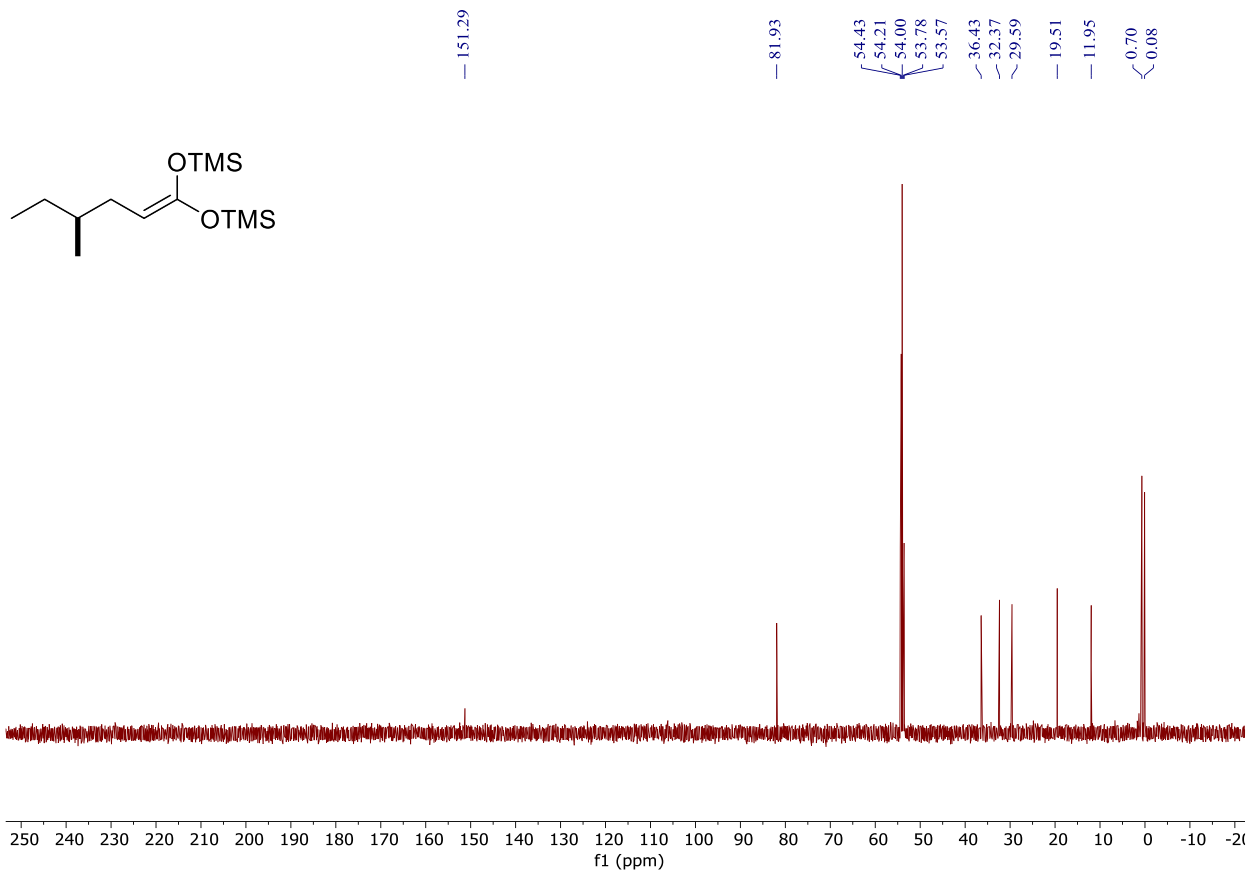
151.29
81.93
54.43
54.21
54.00
53.78
53.57
36.43
32.37
29.59
19.51
11.95
0.70
0.08
OTMS
OTMS
f1 (ppm)

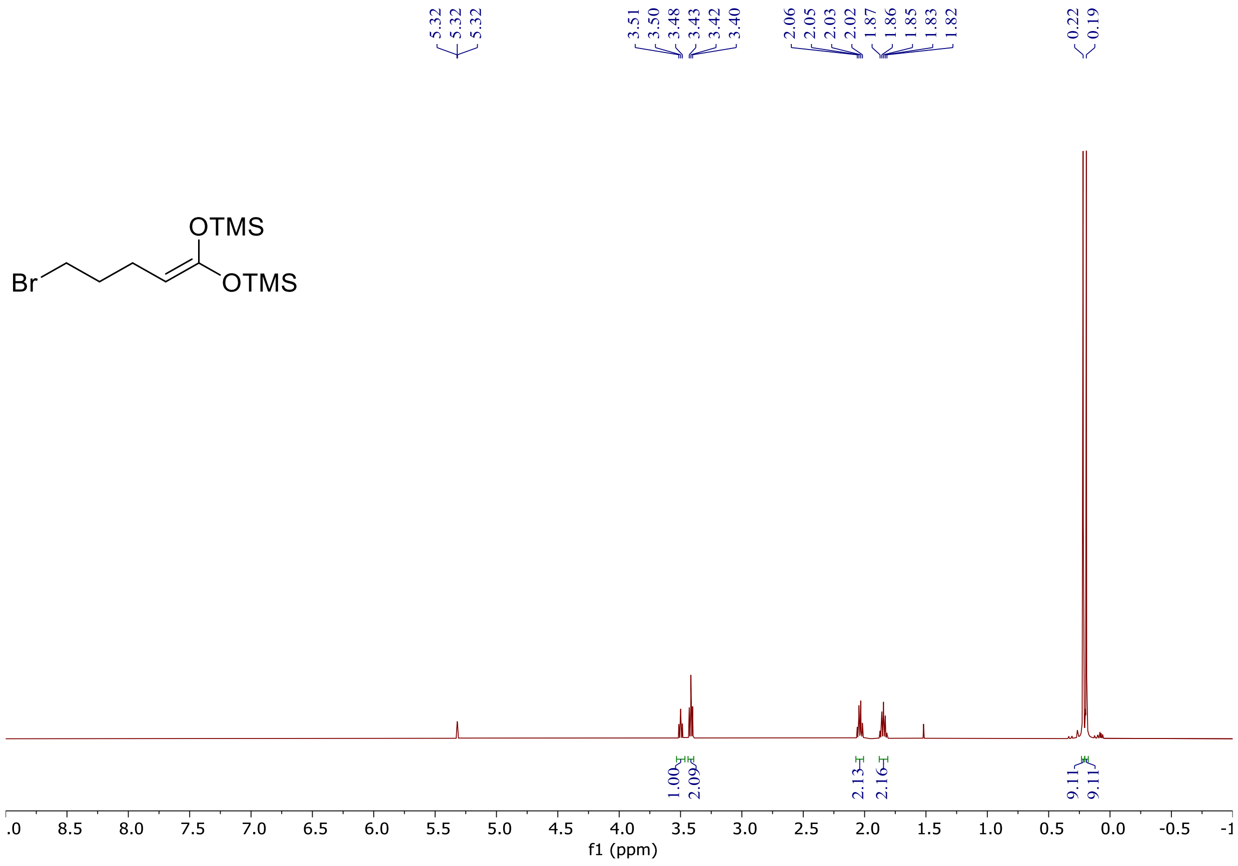
5.32
5.32
5.32
3.51
3.50
3.48
3.43
3.42
3.40
2.06
2.05
2.03
2.02
1.87
1.86
1.85
1.83
1.82
0.22
0.19
Br
OTMS
OTMS
1.00
2.09
2.13
2.16
9.11
9.11
f1 (ppm)

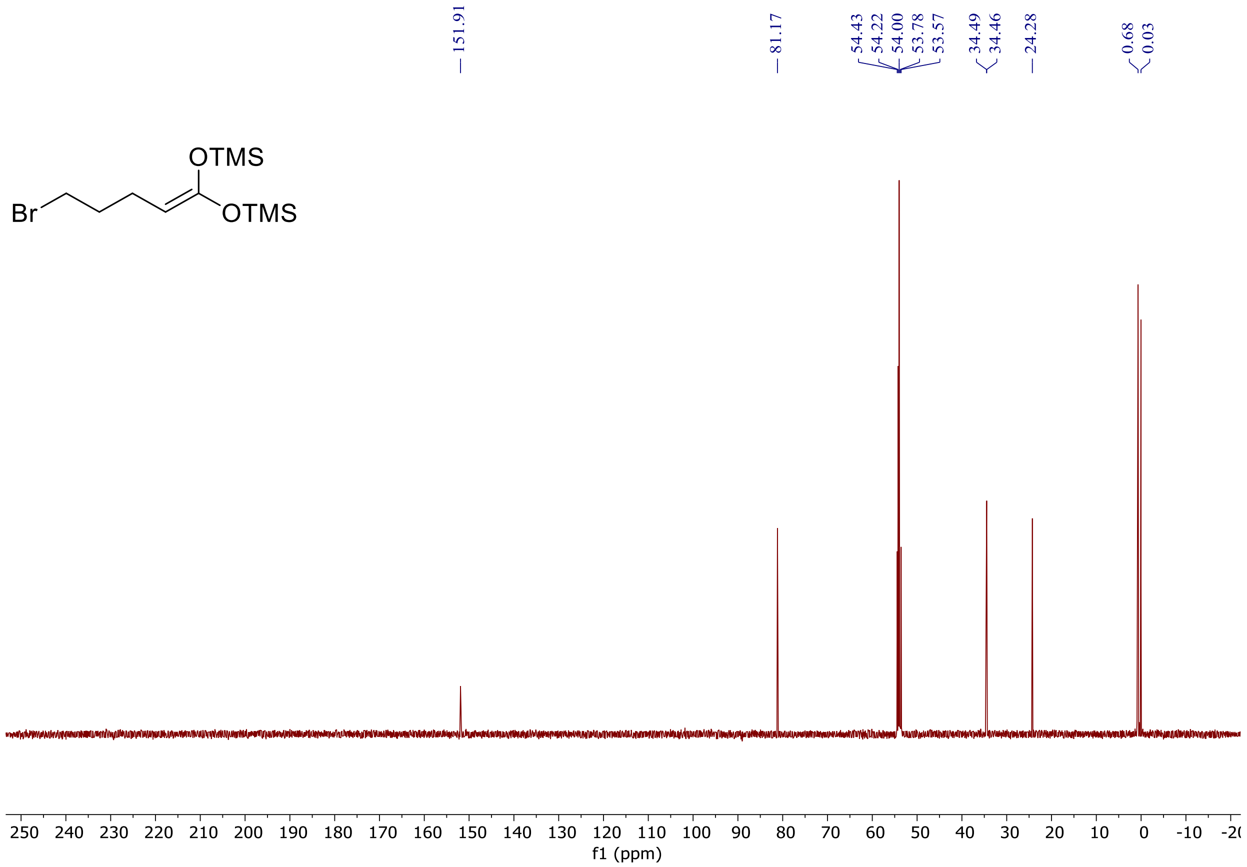
151.91
81.17
54.43
54.22
54.00
53.78
53.57
34.49
34.46
24.28
0.68
0.03
OTMS
Br
OTMS
250 240 230 220 210 200 190 180 170 160 150 140 130 120 110 100 90 80 70 60 50 40 30 20 10 0 -10
f1 (ppm)

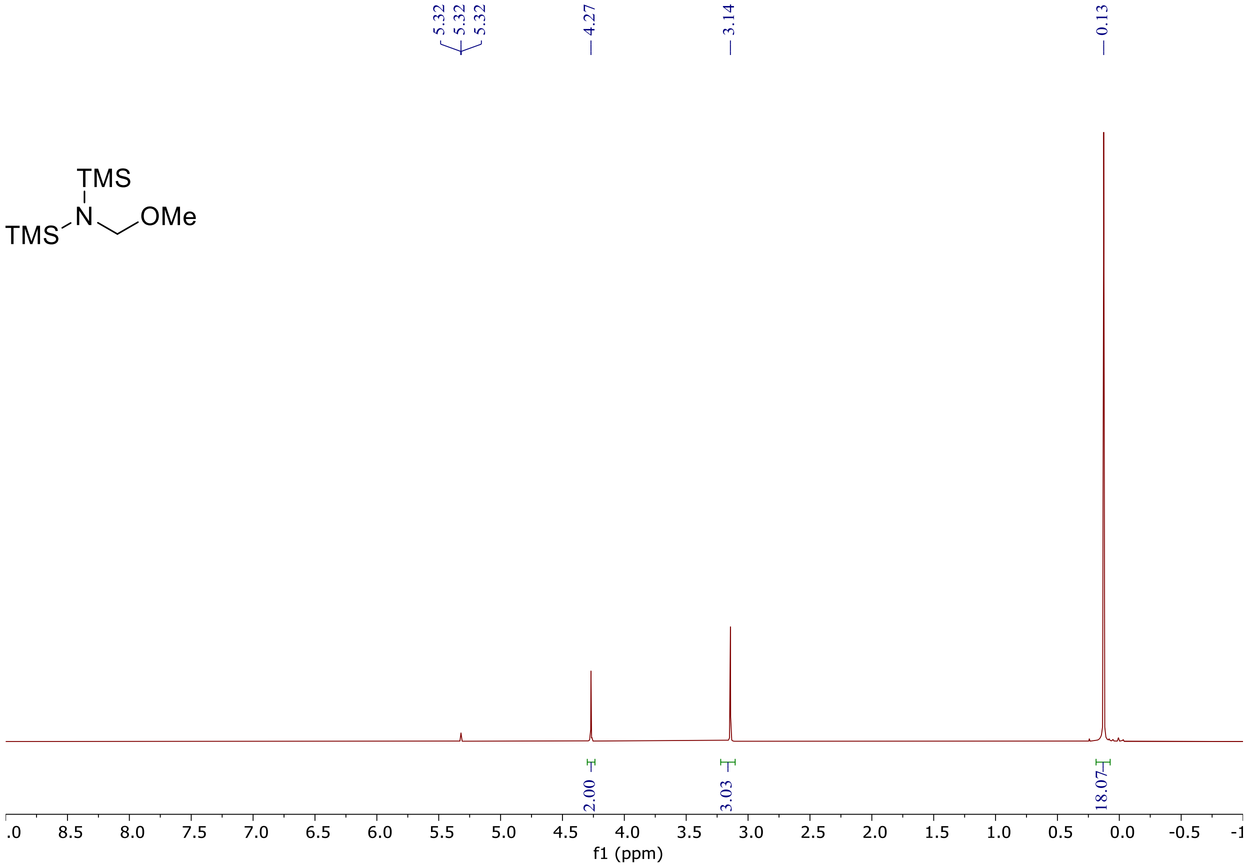
5.32
5.32
5.32
4.27
3.14
0.13
TMS
N
OMe
TMS
2.00
3.03
18.07
8.5 8.0 7.5 7.0 6.5 6.0 5.5 5.0 4.5 4.0 3.5 3.0 2.5 2.0 1.5 1.0 0.5 0.0 -0.5
f1 (ppm)

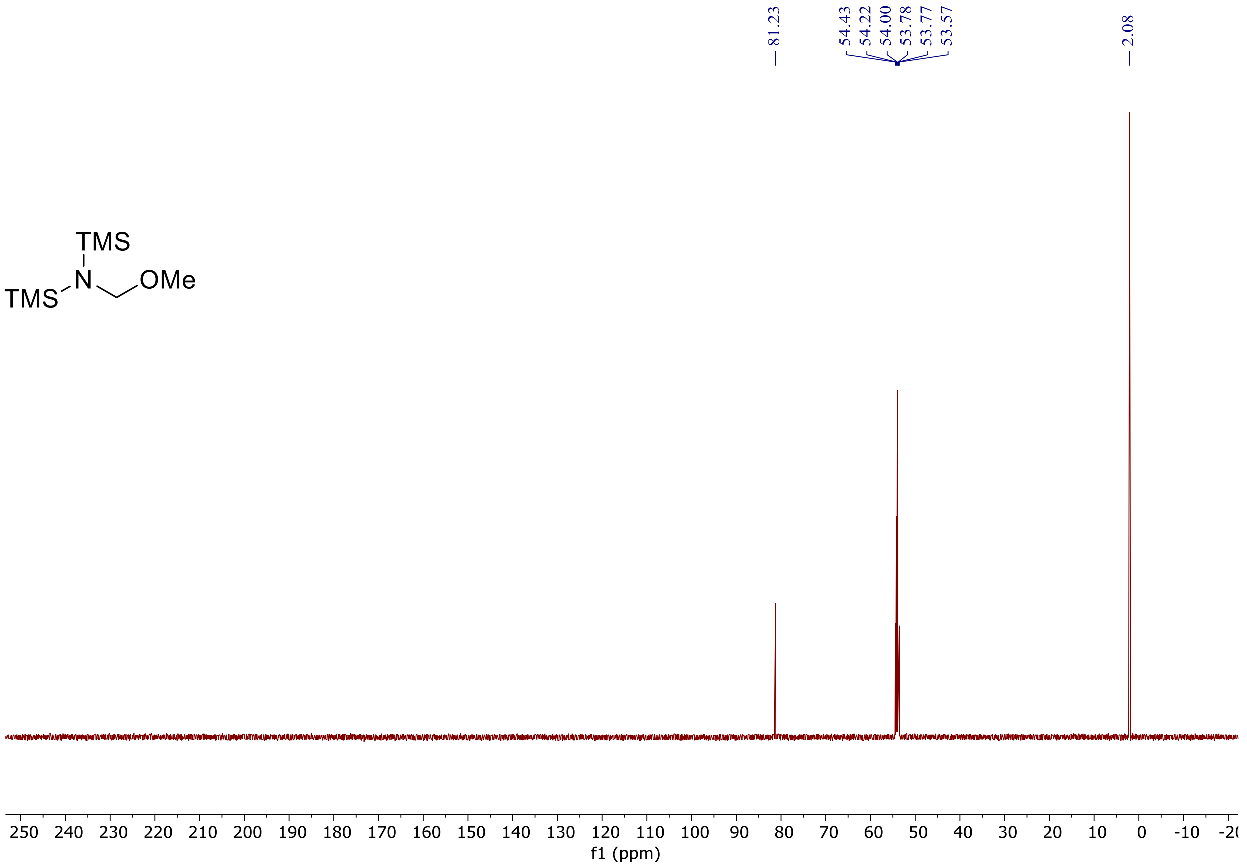
81.23
54.43
54.22
54.00
53.78
53.77
53.57
2.08
TMS
N
OMe
TMS
250 240 230 220 210 200 190 180 170 160 150 140 130 120 110 100 90 80 70 60 50 40 30 20 10 0 -10
f1 (ppm)

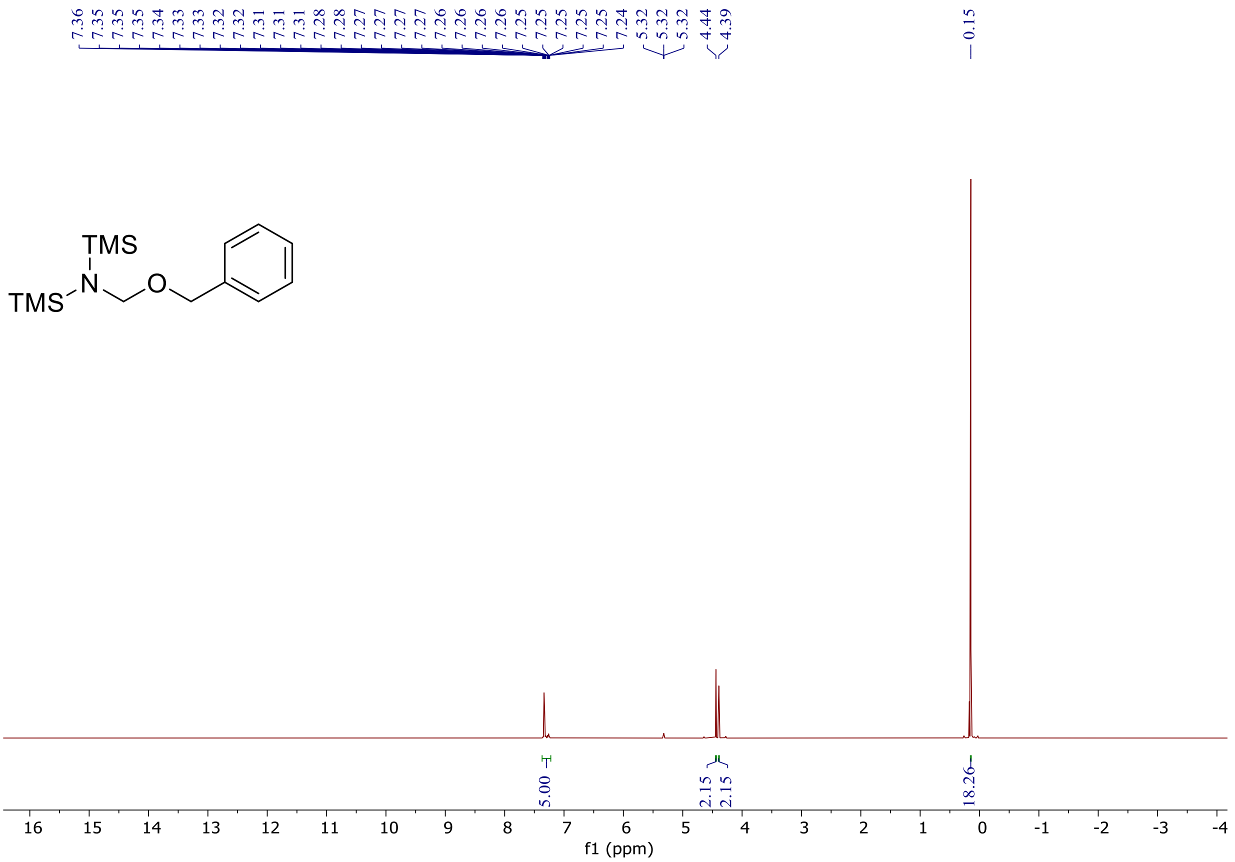
7.36 7.35 7.35 7.35 7.34 7.33 7.33 7.32 7.32 7.31 7.31 7.31 7.28 7.28 7.27 7.27 7.27 7.27 7.26 7.26 7.26 7.25 7.25 7.25 7.25 7.25 7.24
5.32 5.32 5.32
4.44 4.39
0.15
TMS
N
O
TMS
5.00
2.15
2.15
18.26
16 15 14 13 12 11 10 9 8 7 6 5 4 3 2 1 0 -1 -2 -3 -4
f1 (ppm)

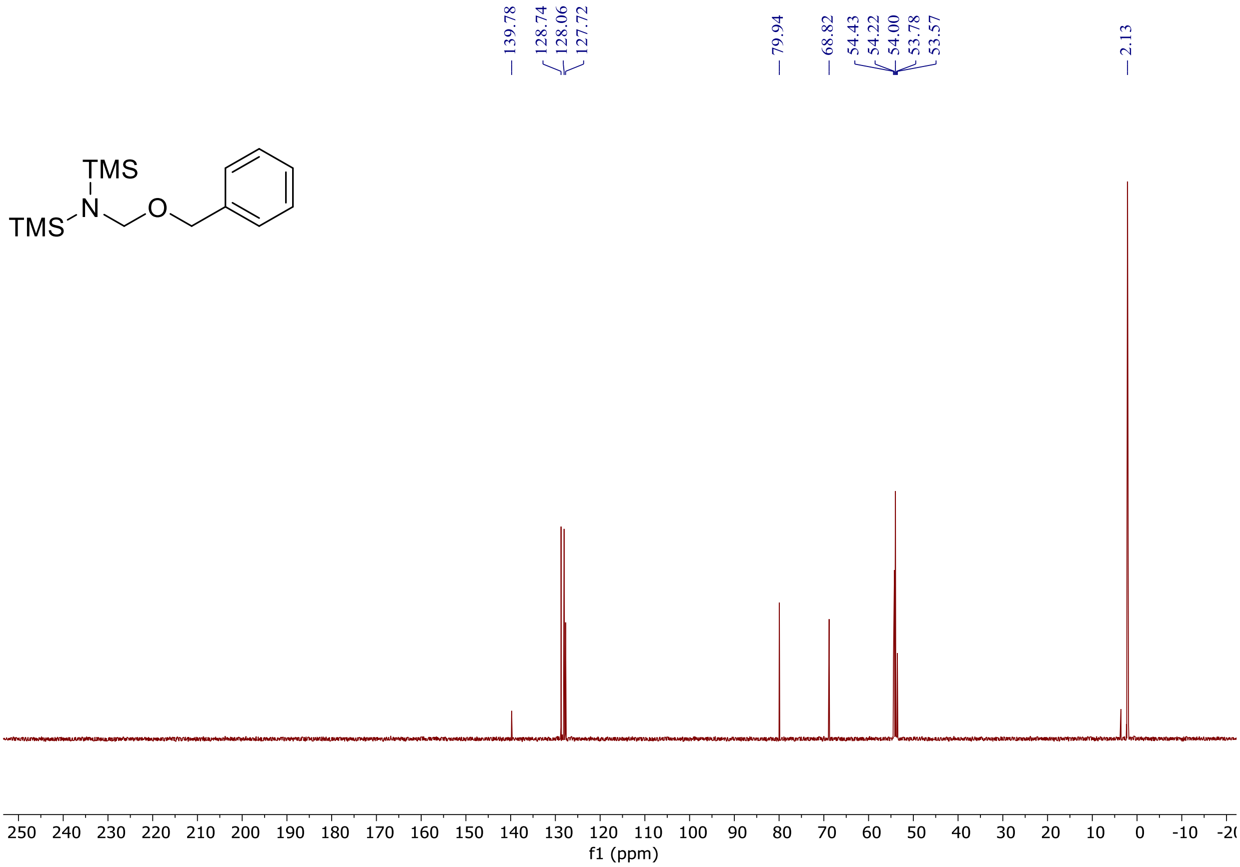
139.78
128.74
128.06
127.72
79.94
68.82
54.43
54.22
54.00
53.78
53.57
2.13
TMS
N
TMS
O
f1 (ppm)

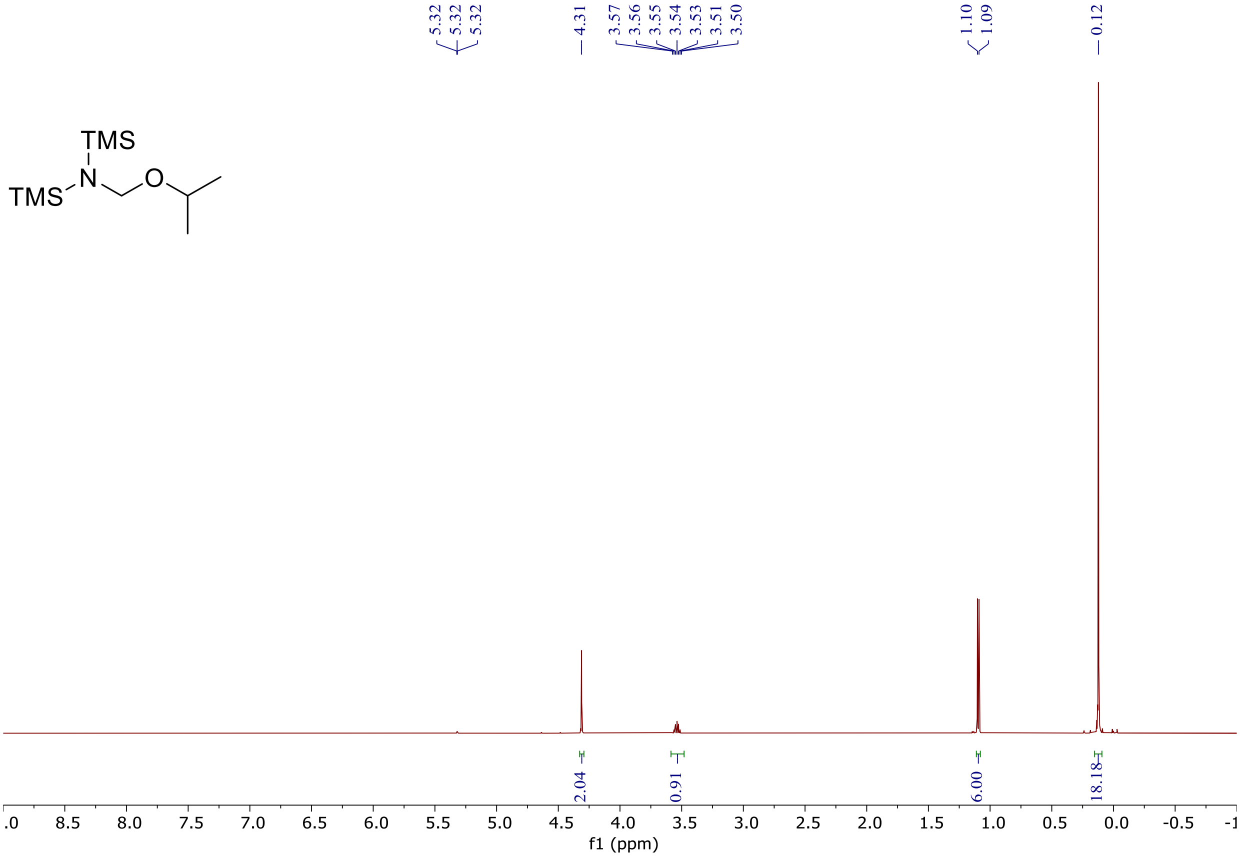
5.32
5.32
5.32
4.31
3.57
3.56
3.55
3.54
3.53
3.51
3.50
1.10
1.09
0.12
TMS
N
TMS
O
2.04
0.91
6.00
18.18
f1 (ppm)

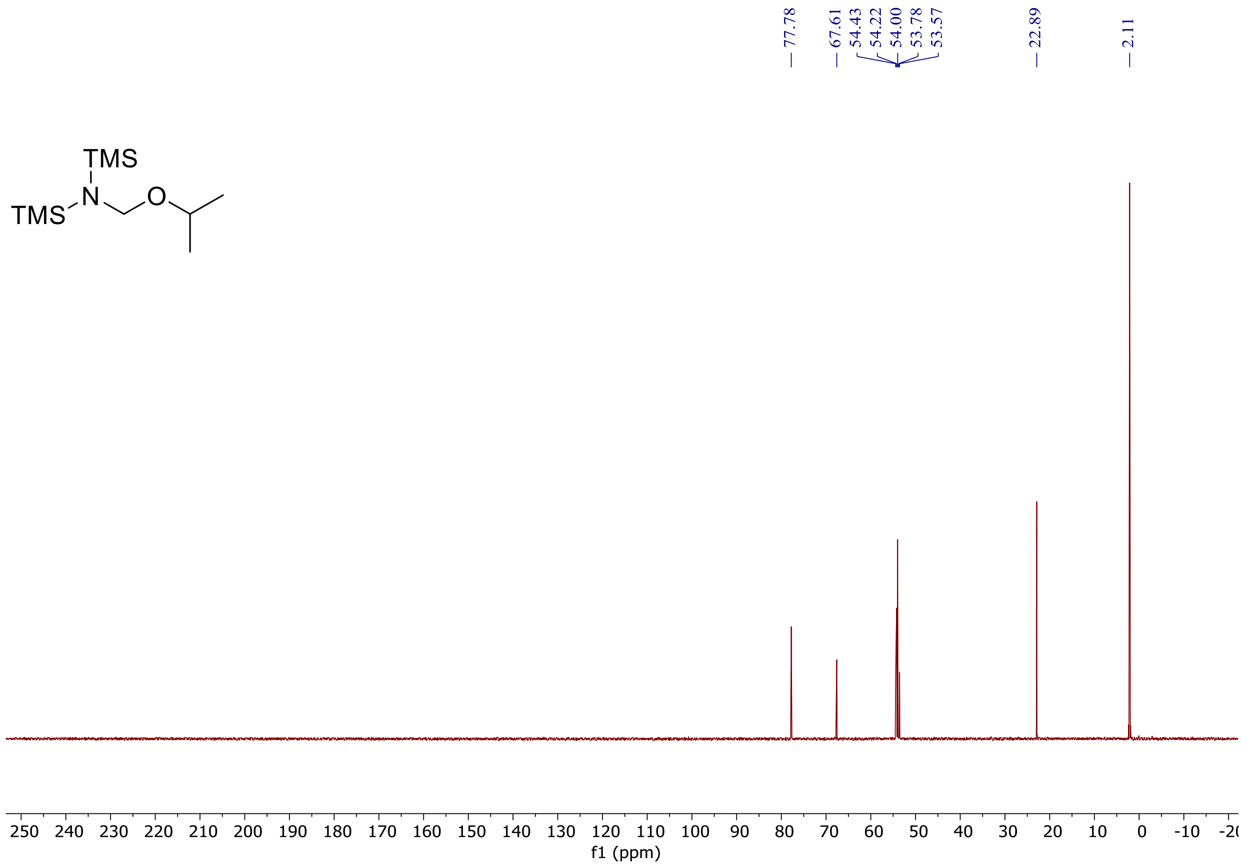
77.78
67.61
54.43
54.22
54.00
53.78
53.57
22.89
2.11
TMS
N
O
TMS
250 240 230 220 210 200 190 180 170 160 150 140 130 120 110 100 90 80 70 60 50 40 30 20 10 0 -10
f1 (ppm)

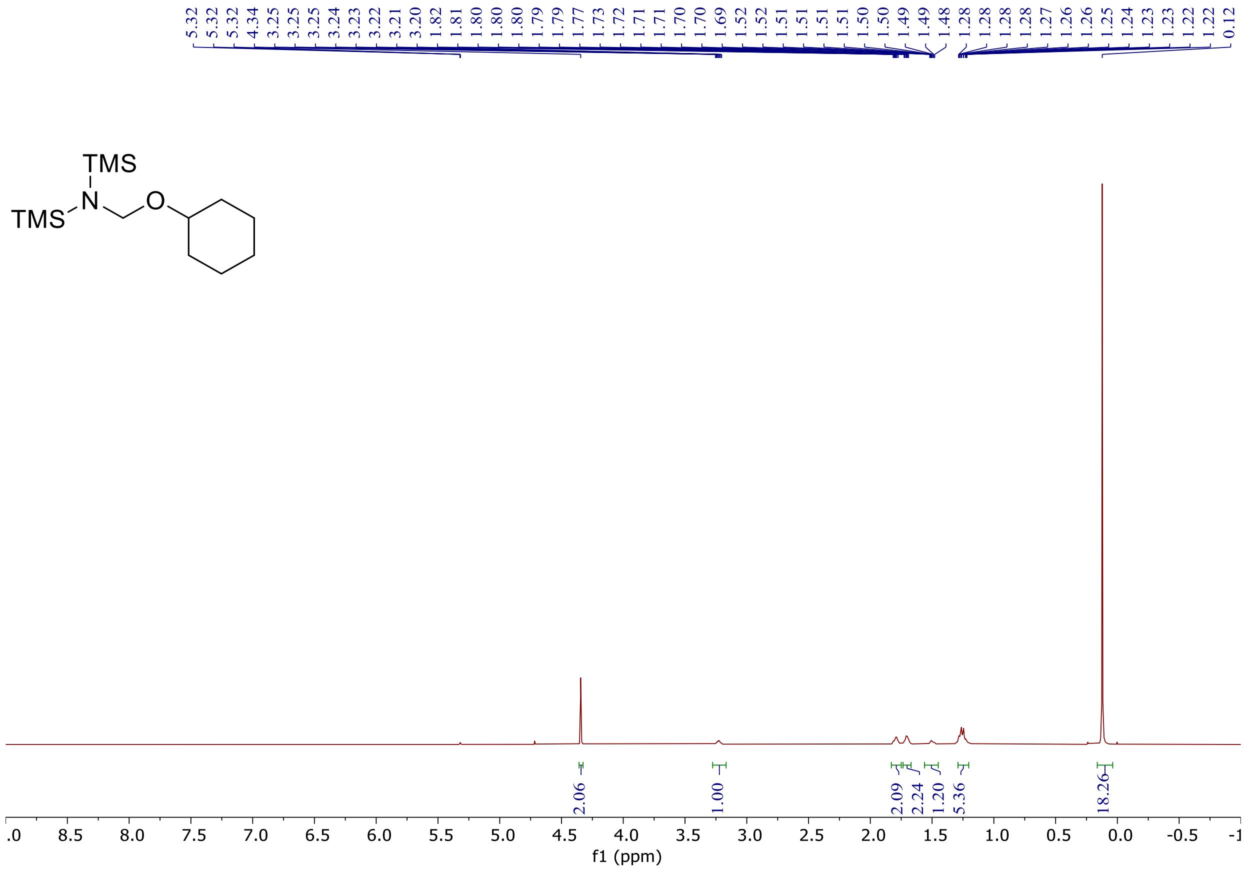
5.32 5.32 5.32 4.34 3.25 3.25 3.24 3.23 3.22 3.21 3.20 1.82 1.81 1.80 1.80 1.80 1.79 1.79 1.77 1.73 1.72 1.71 1.71 1.70 1.69 1.52 1.51 1.51 1.51 1.50 1.50 1.49 1.49 1.48 1.28 1.28 1.28 1.28 1.27 1.26 1.26 1.25 1.24 1.23 1.23 1.22 1.22 0.12
TMS
N
O
TMS
2.06
1.00
2.09
2.24
1.20
5.36
18.26
8.5 8.0 7.5 7.0 6.5 6.0 5.5 5.0 4.5 4.0 3.5 3.0 2.5 2.0 1.5 1.0 0.5 0.0 -0.5
f1 (ppm)

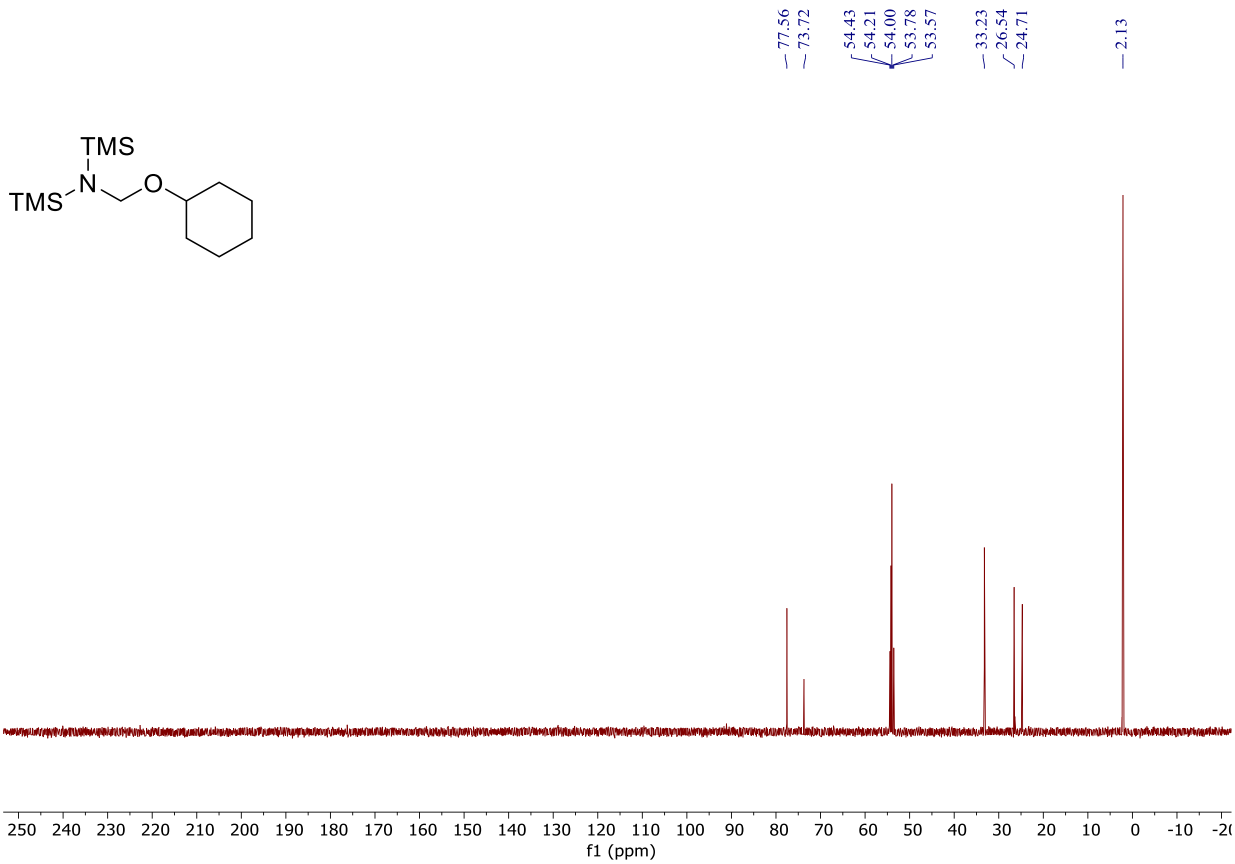
77.56
73.72
54.43
54.21
54.00
53.78
53.57
33.23
26.54
24.71
2.13
TMS
TMS
N
O
250 240 230 220 210 200 190 180 170 160 150 140 130 120 110 100 90 80 70 60 50 40 30 20 10 0 -10 -20
f1 (ppm)

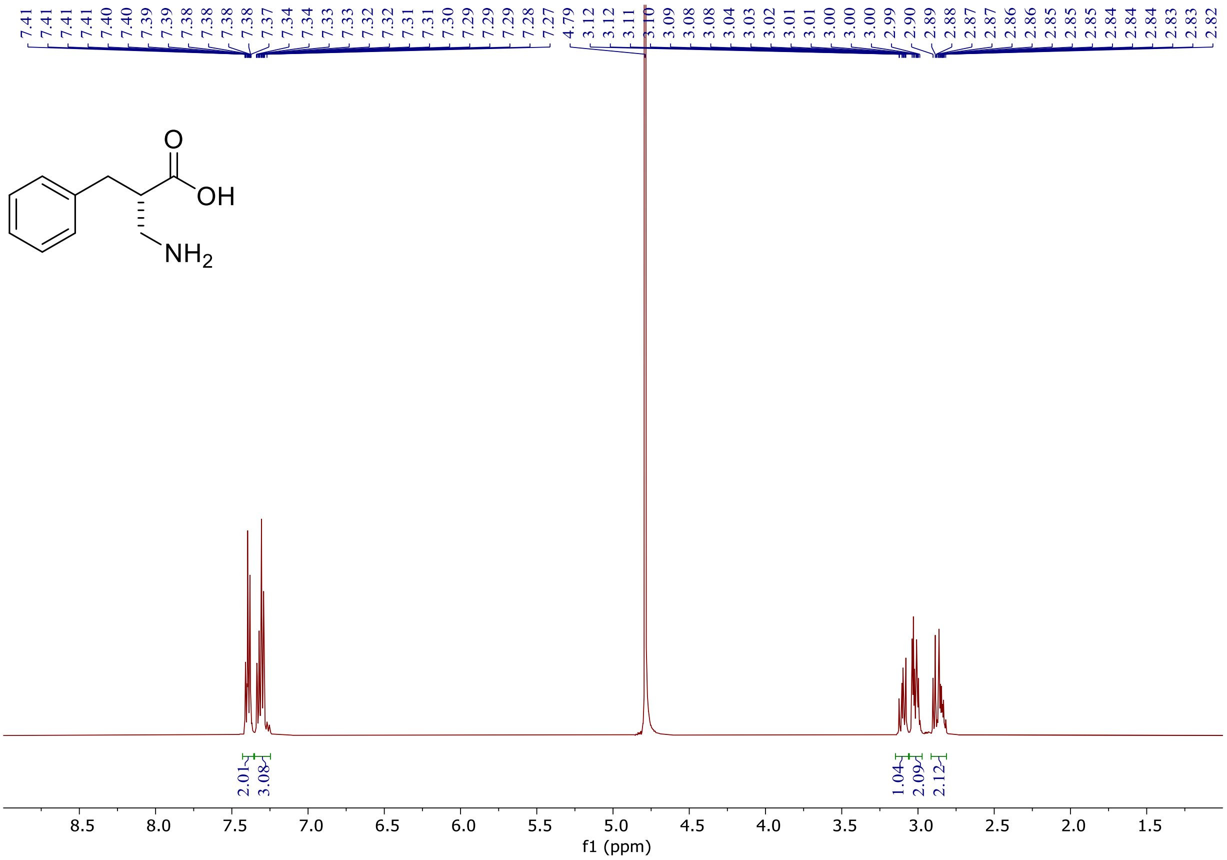
7.41 7.41 7.41 7.41 7.40 7.40 7.39 7.39 7.38 7.38 7.38 7.38 7.37 7.34 7.34 7.33 7.33 7.32 7.32 7.31 7.31 7.30 7.29 7.29 7.29 7.28 7.27
4.79
3.12 3.12 3.11 3.10 3.09 3.08 3.08 3.04 3.03 3.02 3.01 3.01 3.00 3.00 3.00 2.99 2.90 2.89 2.88 2.87 2.87 2.86 2.86 2.85 2.85 2.85 2.84 2.84 2.84 2.83 2.83 2.82
O
OH
NH2
2.01
3.08
1.04
2.09
2.12
8.5 8.0 7.5 7.0 6.5 6.0 5.5 5.0 4.5 4.0 3.5 3.0 2.5 2.0 1.5
f1 (ppm)

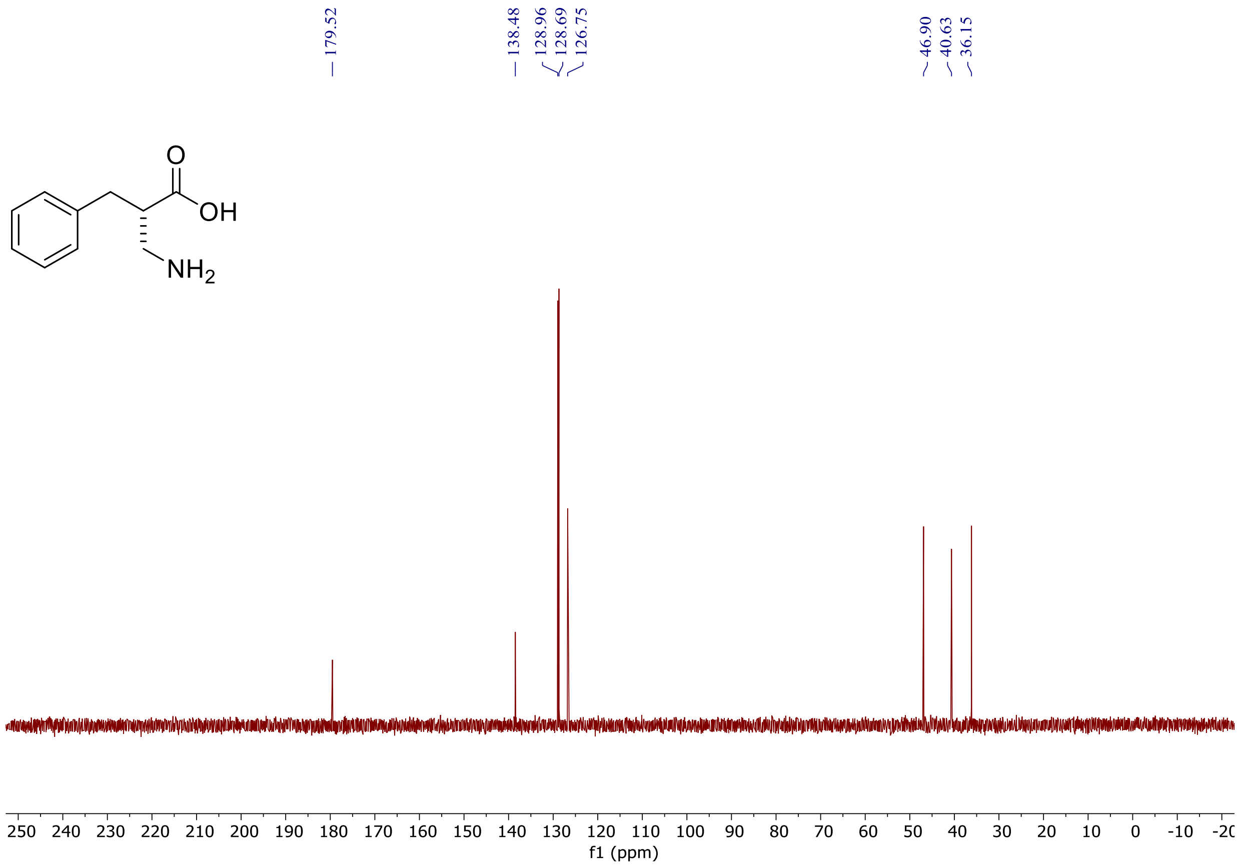
179.52
138.48
128.96
128.69
126.75
46.90
40.63
36.15
OH
O
NH2
f1 (ppm)

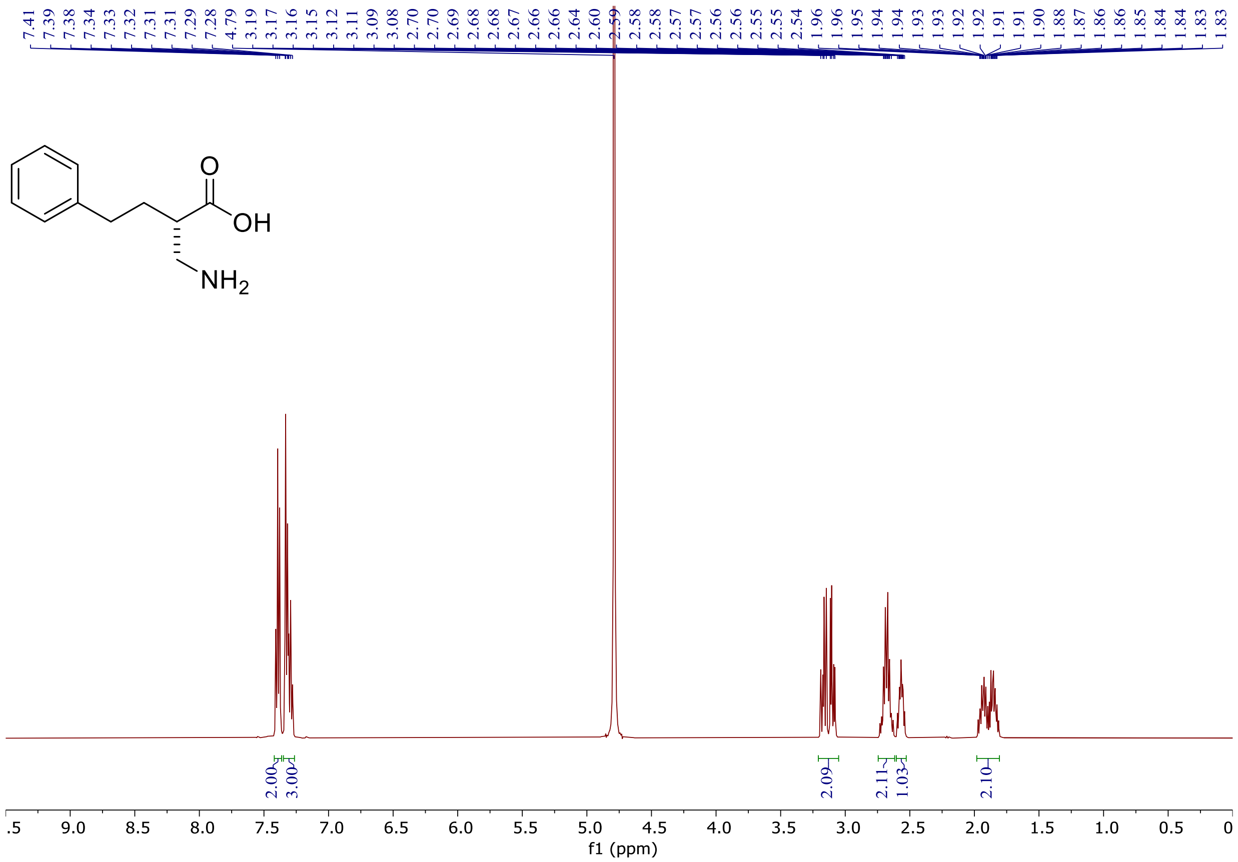
O
OH
NH2
2.00
3.00
2.09
2.11
1.03
2.10
f1 (ppm)

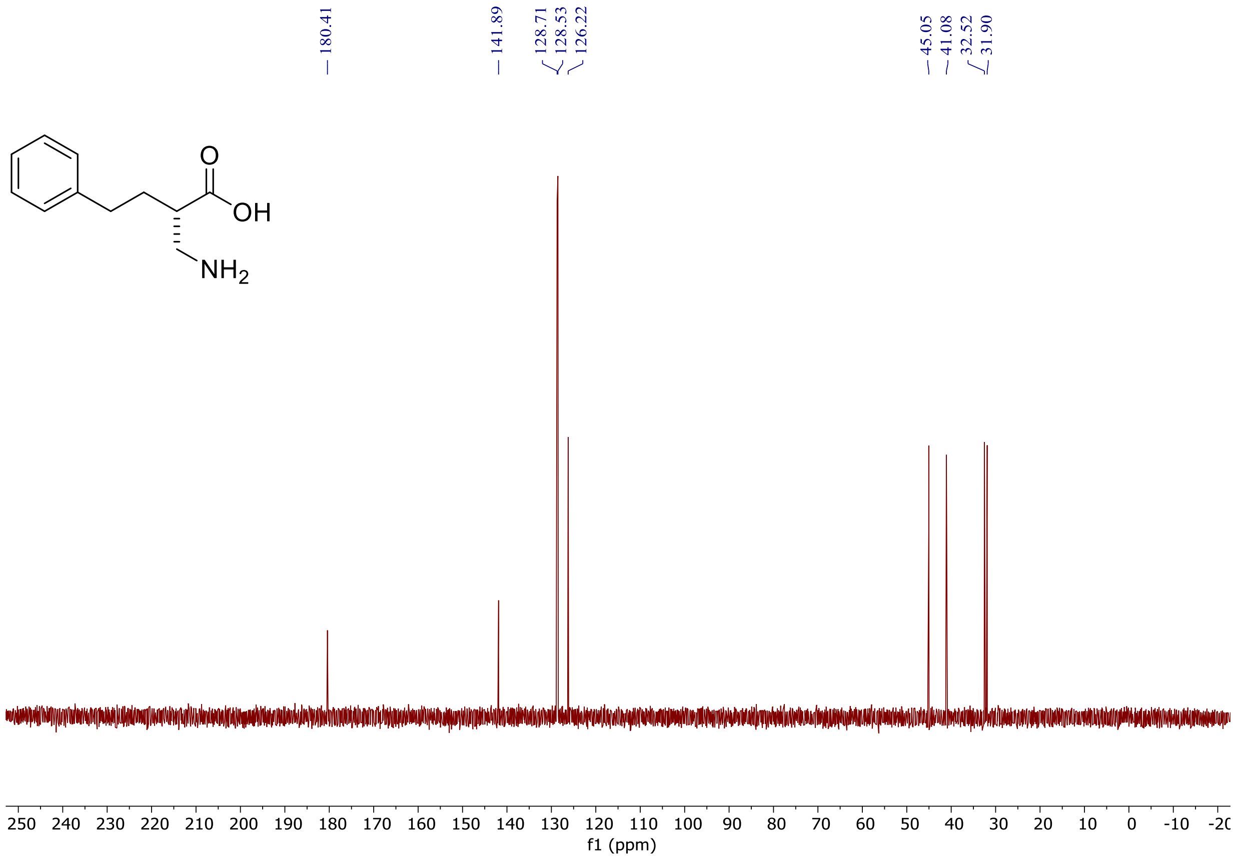
180.41
141.89
128.71
128.53
126.22
45.05
41.08
32.52
31.90
O
OH
NH_2
250 240 230 220 210 200 190 180 170 160 150 140 130 120 110 100 90 80 70 60 50 40 30 20 10 0 -10 -20
f1 (ppm)

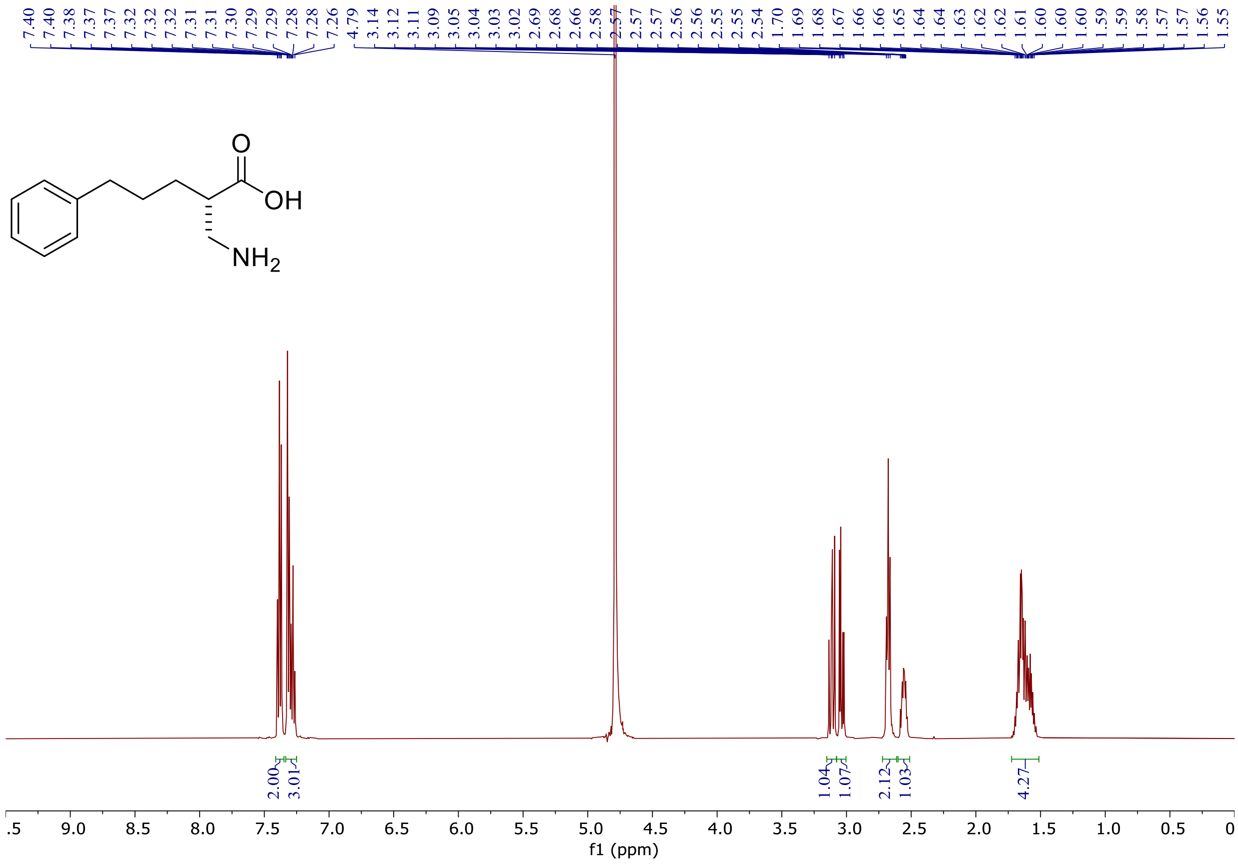
7.40 7.40 7.38 7.37 7.37 7.32 7.32 7.32 7.31 7.31 7.30 7.29 7.29 7.28 7.28 7.26 4.79 3.14 3.12 3.11 3.09 3.05 3.04 3.03 3.02 2.69 2.68 2.66 2.58 2.57 2.57 2.57 2.56 2.56 2.55 2.55 2.54 1.70 1.69 1.68 1.67 1.66 1.66 1.65 1.64 1.64 1.63 1.62 1.62 1.61 1.60 1.60 1.59 1.59 1.58 1.57 1.57 1.56 1.55
O
OH
NH_2
2.00
3.01
1.04
1.07
2.12
1.03
4.27
9.5 9.0 8.5 8.0 7.5 7.0 6.5 6.0 5.5 5.0 4.5 4.0 3.5 3.0 2.5 2.0 1.5 1.0 0.5 0
f1 (ppm)

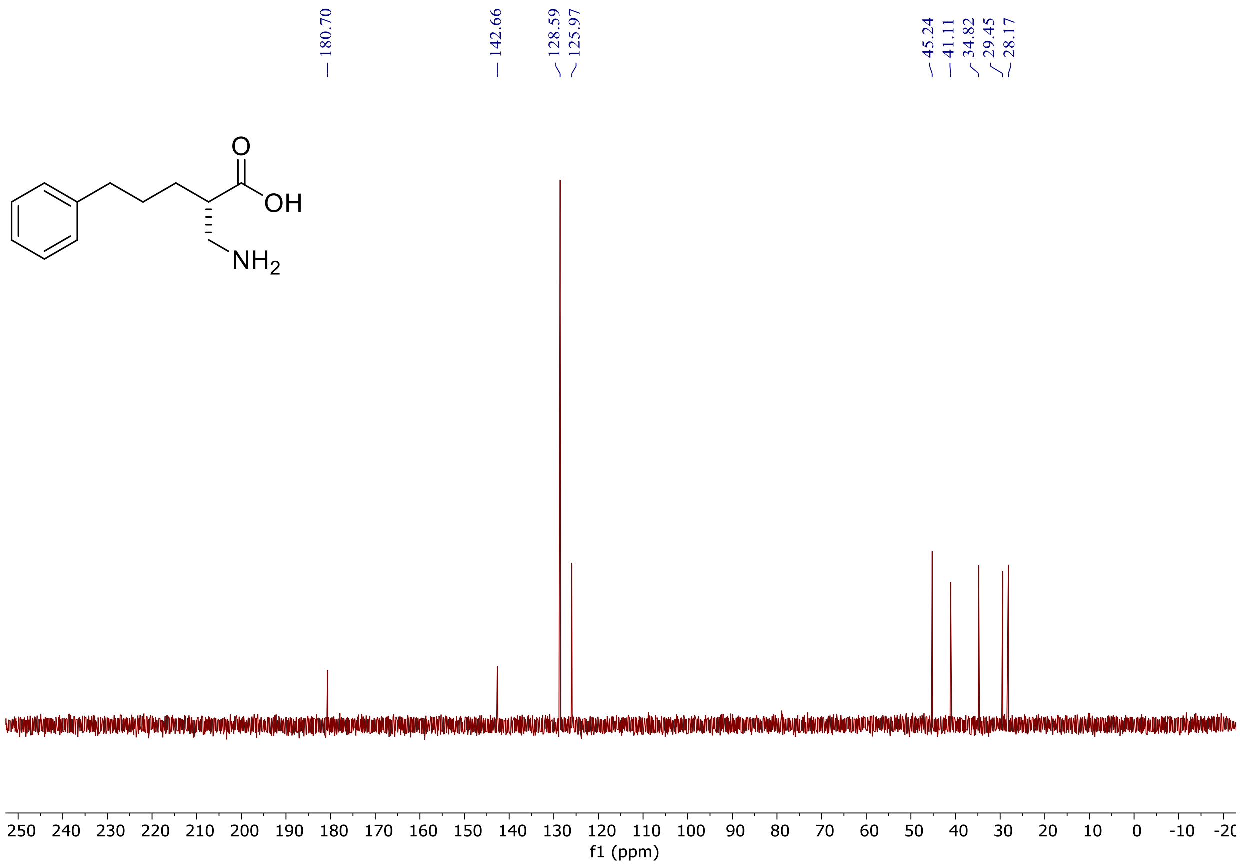

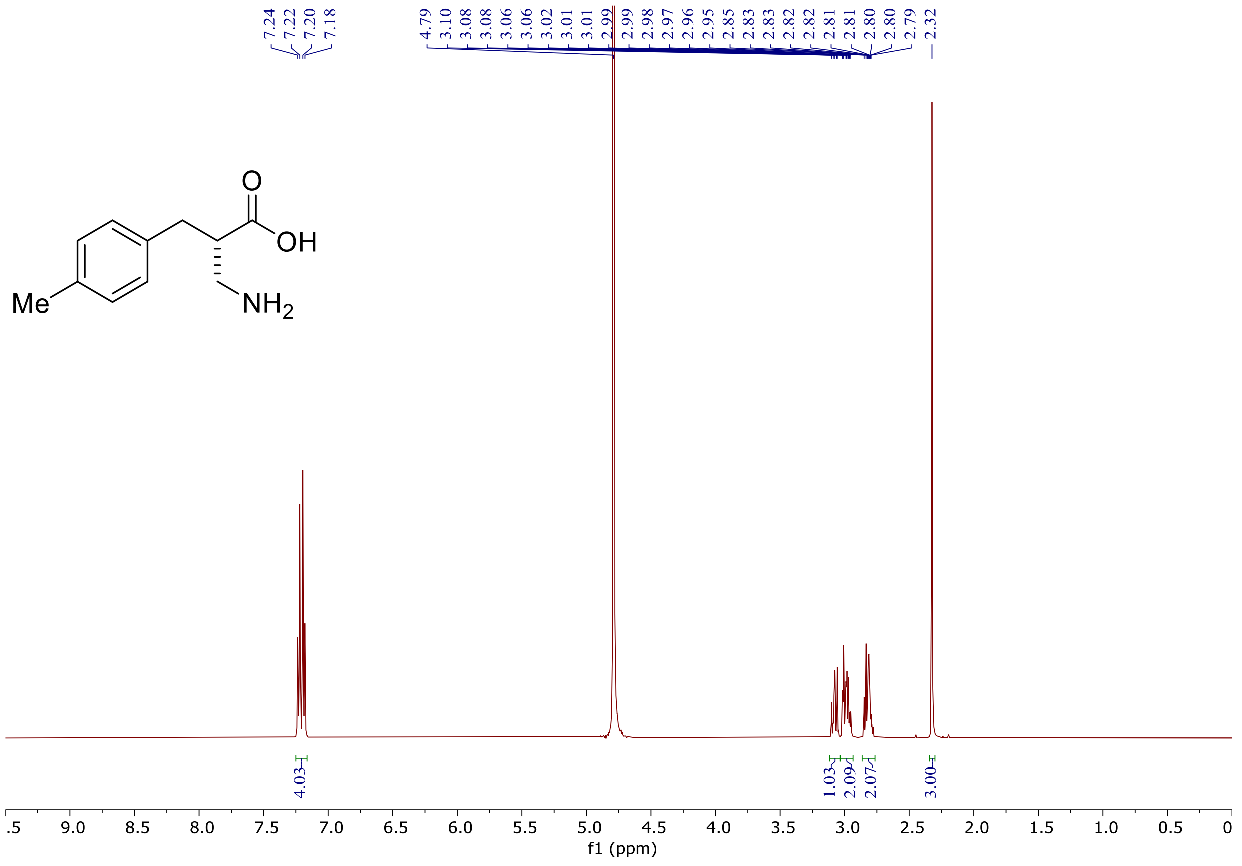

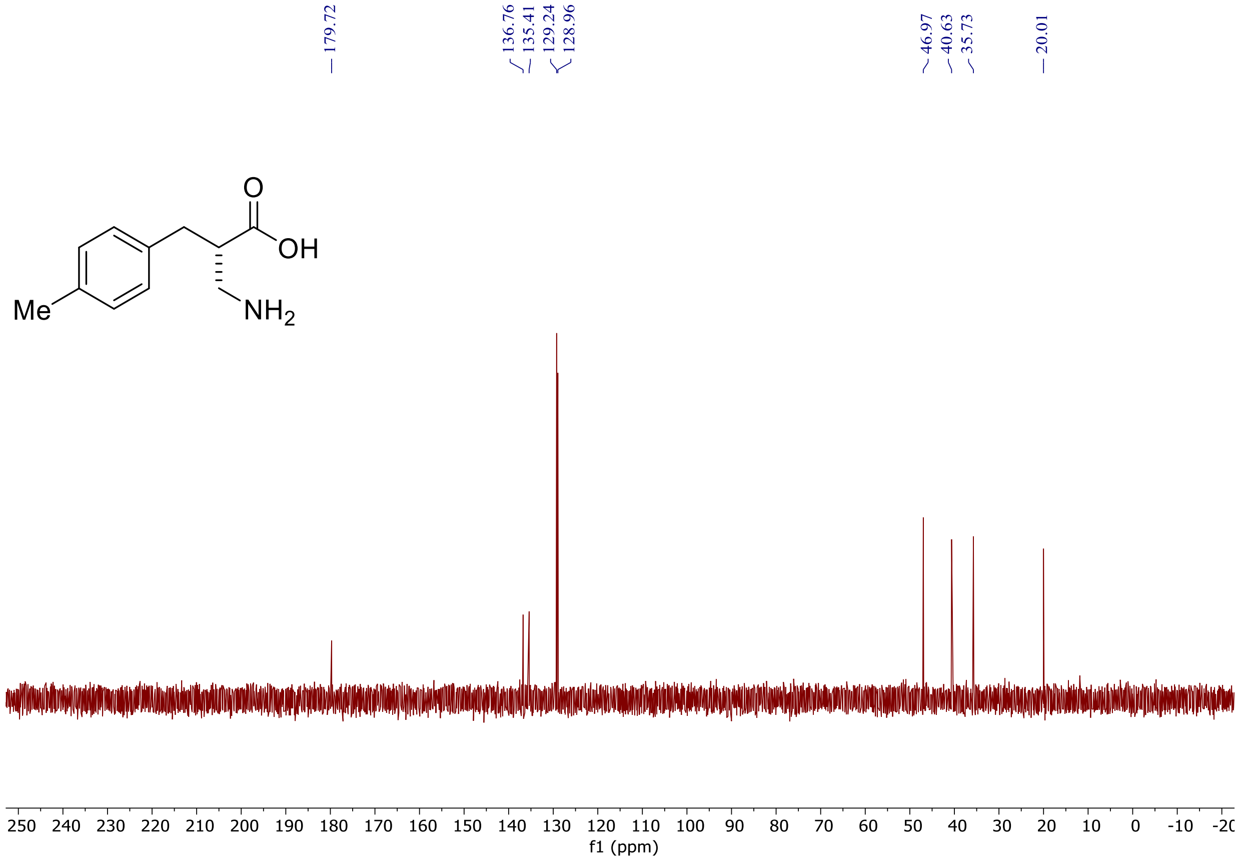
179.72
136.76
135.41
129.24
128.96
46.97
40.63
35.73
20.01
O
OH
Me
NH2
250 240 230 220 210 200 190 180 170 160 150 140 130 120 110 100 90 80 70 60 50 40 30 20 10 0 -10 -20
f1 (ppm)

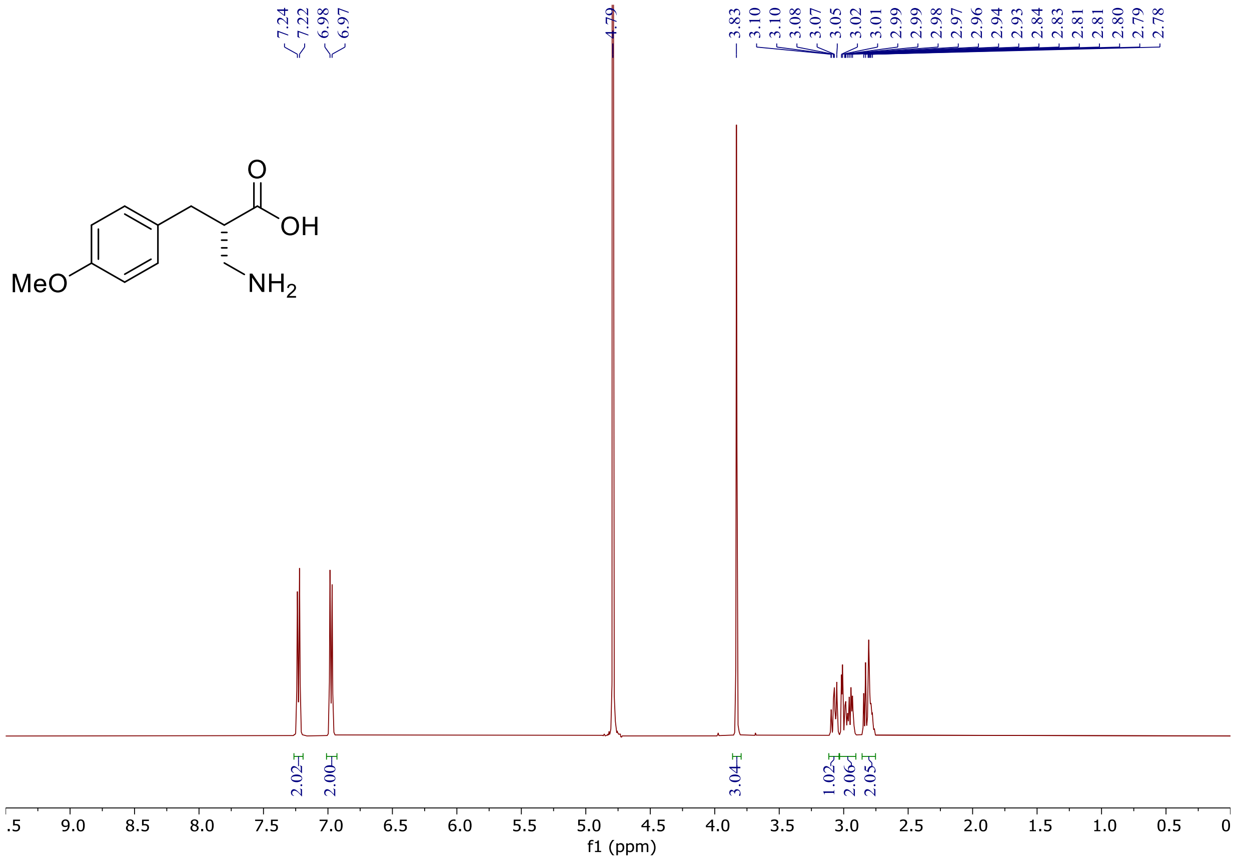
7.24
7.22
6.98
6.97
4.79
3.83
3.10
3.10
3.08
3.07
3.05
3.02
3.01
2.99
2.99
2.98
2.97
2.96
2.94
2.93
2.84
2.83
2.81
2.81
2.80
2.79
2.78
O
OH
MeO
NH2
2.02
2.00
3.04
1.02
2.06
2.05
9.0 8.5 8.0 7.5 7.0 6.5 6.0 5.5 5.0 4.5 4.0 3.5 3.0 2.5 2.0 1.5 1.0 0.5 0
f1 (ppm)

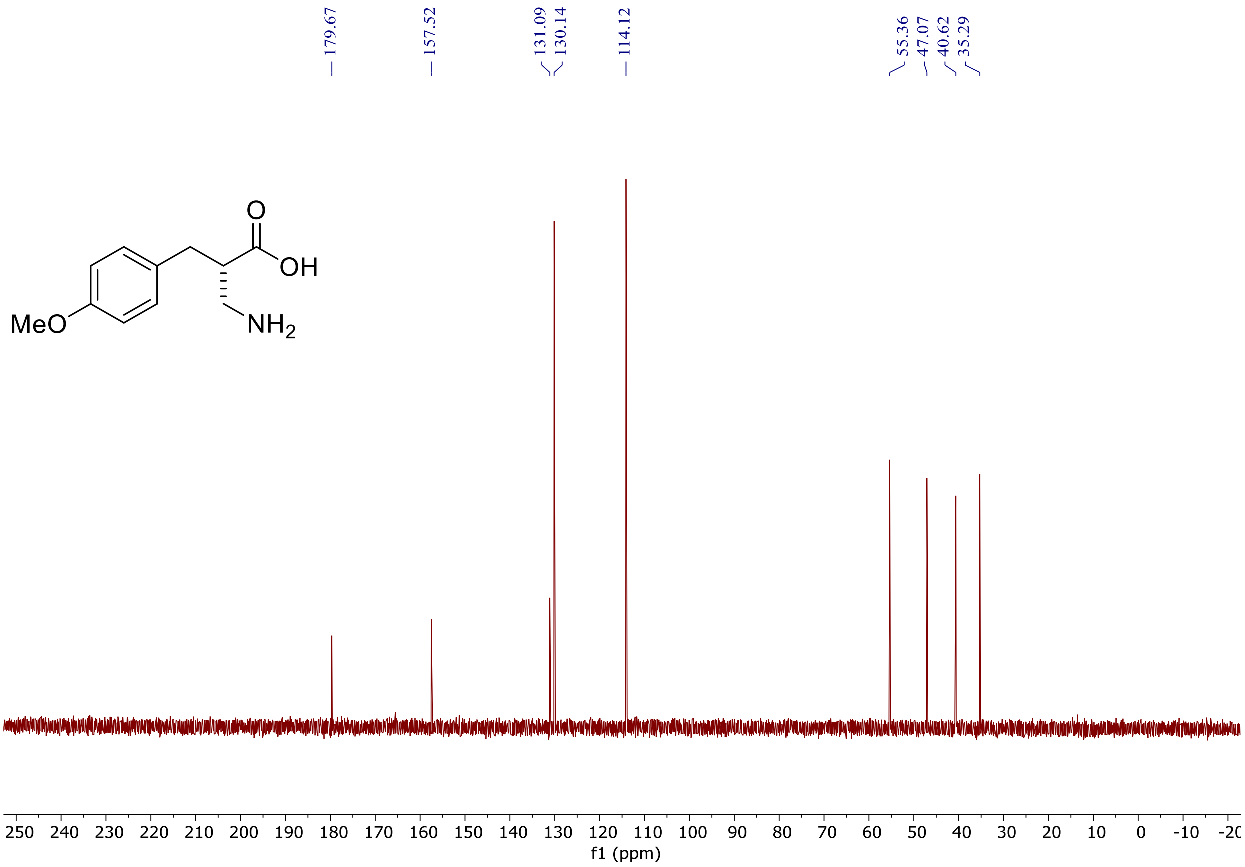
179.67
157.52
131.09
130.14
114.12
55.36
47.07
40.62
35.29
O
OH
MeO
NH2
250 240 230 220 210 200 190 180 170 160 150 140 130 120 110 100 90 80 70 60 50 40 30 20 10 0 -10
f1 (ppm)

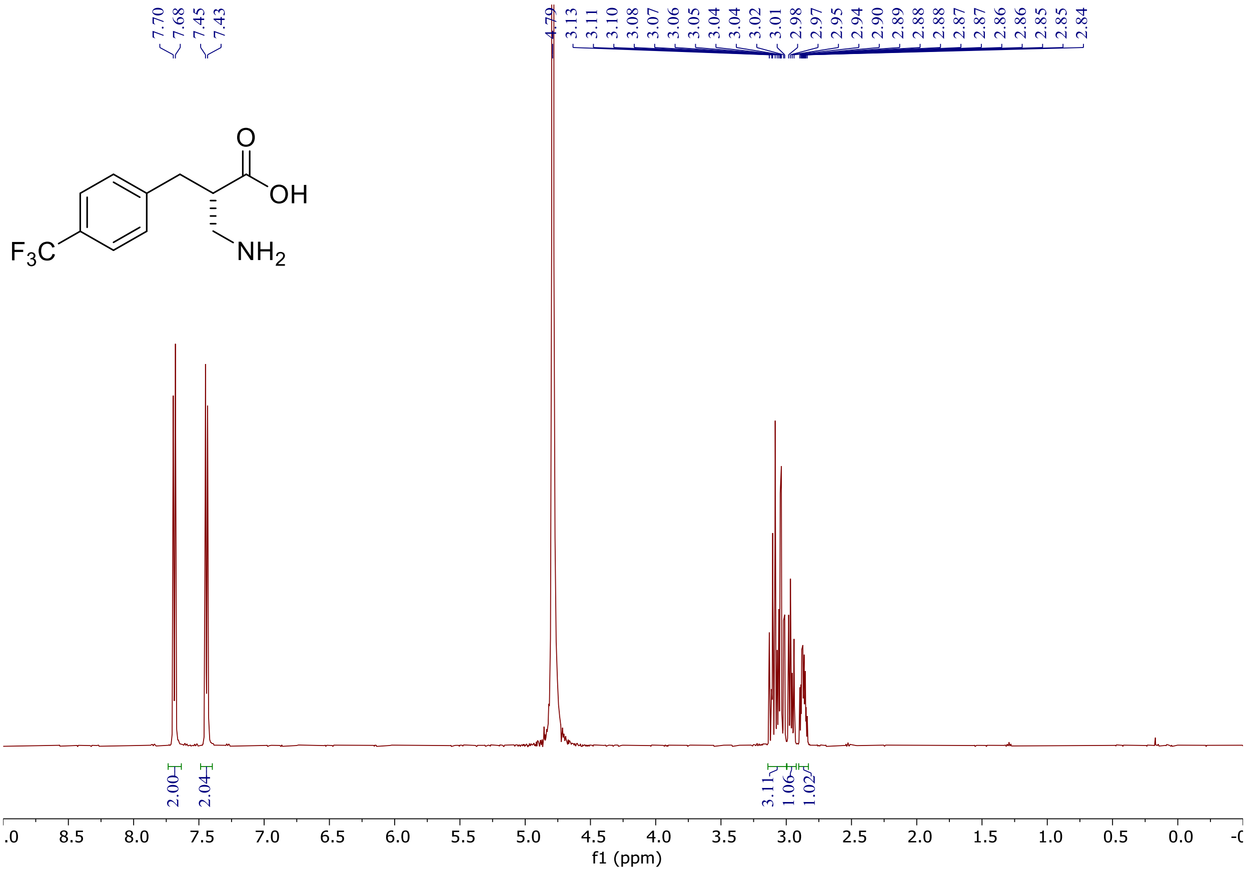
7.70
7.68
7.45
7.43
4.79
3.13
3.11
3.10
3.08
3.07
3.06
3.05
3.04
3.02
3.01
2.98
2.97
2.95
2.94
2.90
2.89
2.88
2.88
2.87
2.86
2.86
2.85
2.84
O
OH
F3C
NH2
2.00
2.04
3.11
1.06
1.02
8.5 8.0 7.5 7.0 6.5 6.0 5.5 5.0 4.5 4.0 3.5 3.0 2.5 2.0 1.5 1.0 0.5 0.0
f1 (ppm)

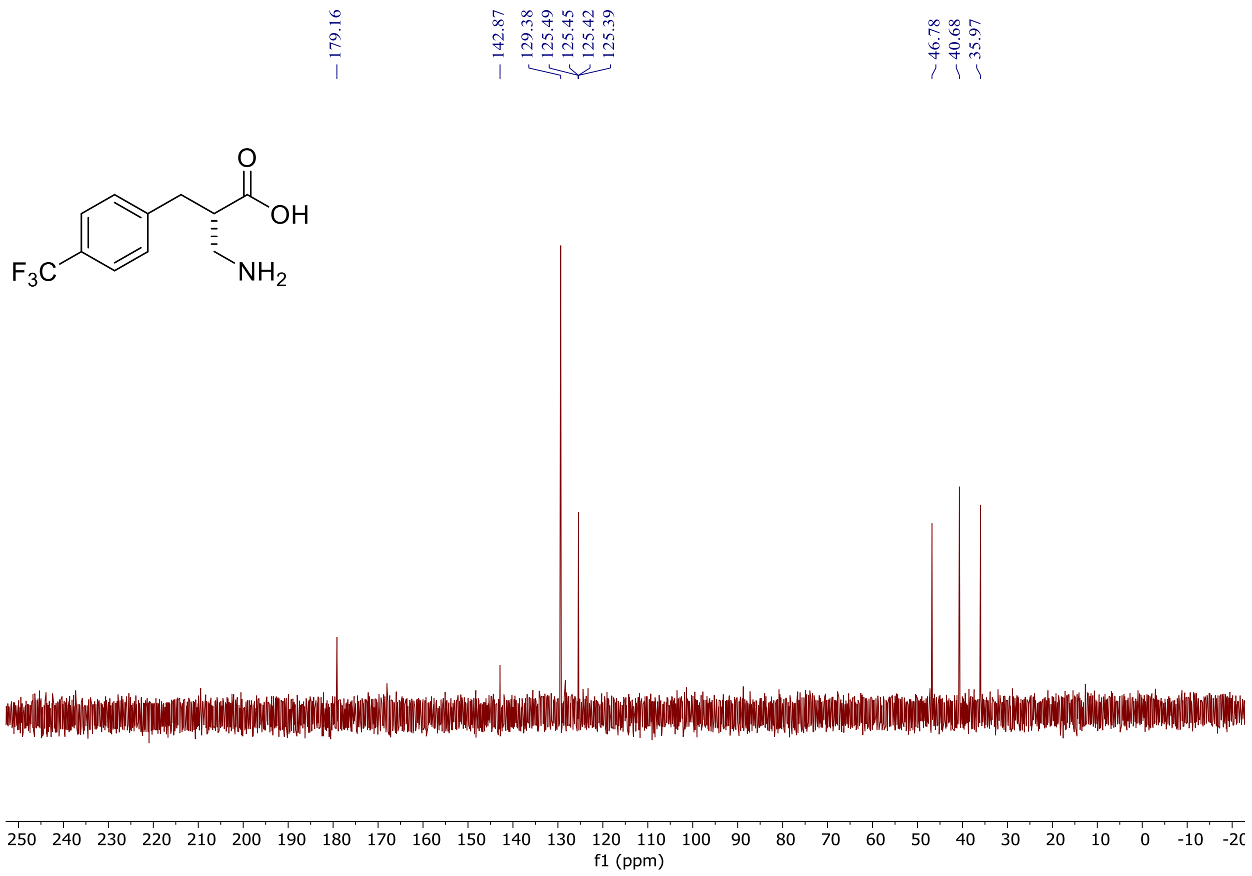
179.16
142.87
129.38
125.49
125.45
125.42
125.39
46.78
40.68
35.97
O
OH
F3C
NH2
250 240 230 220 210 200 190 180 170 160 150 140 130 120 110 100 90 80 70 60 50 40 30 20 10 0 -10 -20
f1 (ppm)

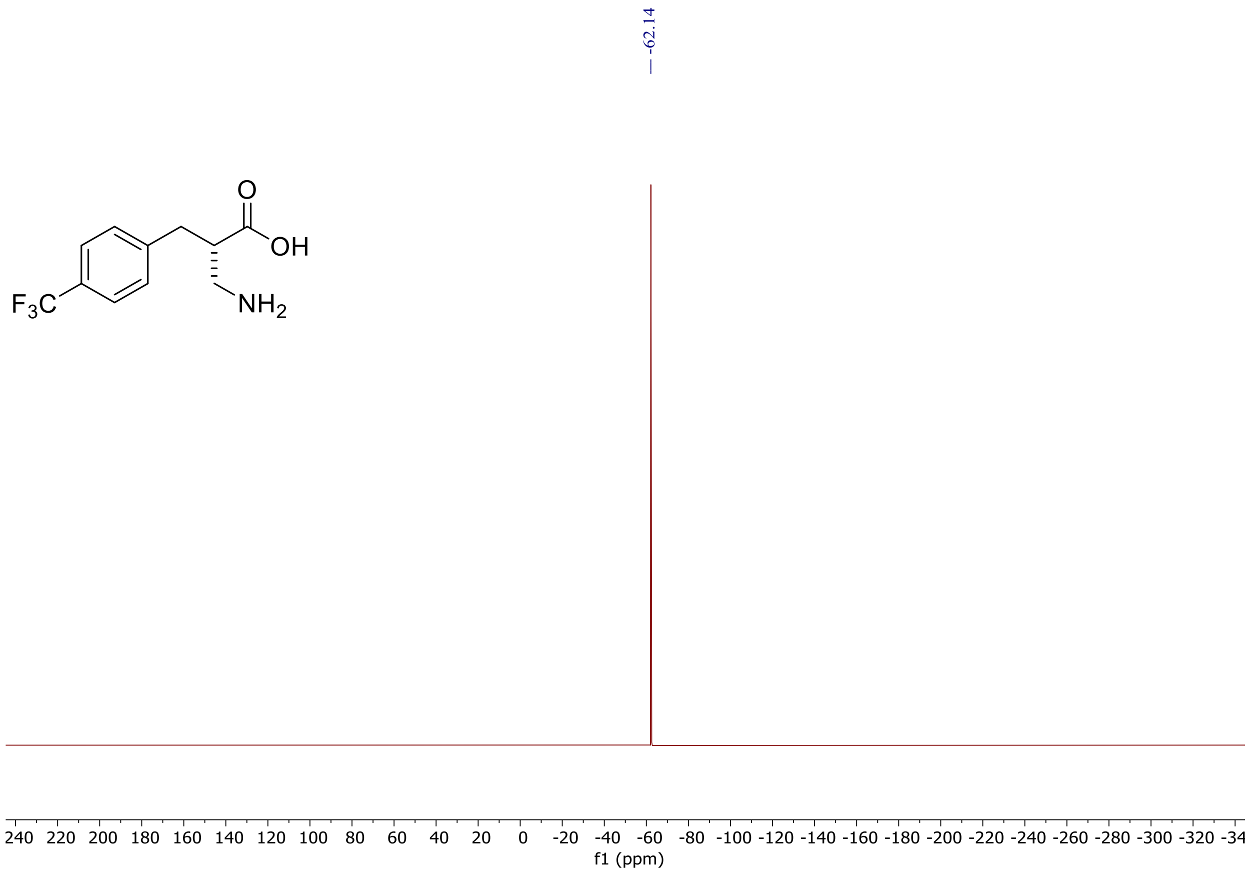
-62.14
O
OH
F3C
NH2
240 220 200 180 160 140 120 100 80 60 40 20 0 -20 -40 -60 -80 -100 -120 -140 -160 -180 -200 -220 -240 -260 -280 -300 -320 -34
f1 (ppm)

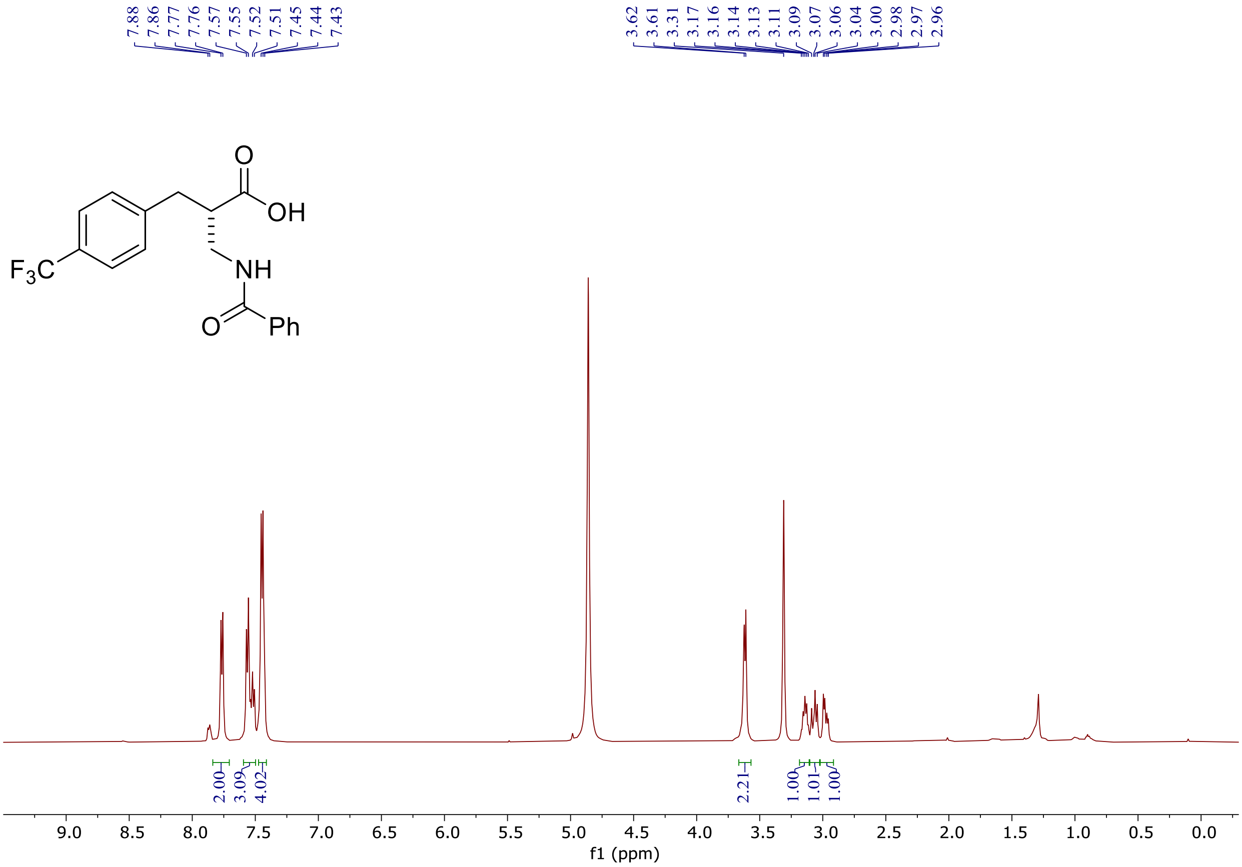
7.88
7.86
7.77
7.76
7.57
7.55
7.52
7.51
7.45
7.44
7.43
3.62
3.61
3.31
3.17
3.16
3.14
3.13
3.11
3.09
3.07
3.06
3.04
3.00
2.98
2.97
2.96
O
OH
F_3C
NH
O
Ph
2.00
3.09
4.02
2.21
1.00
1.01
1.00
9.0 8.5 8.0 7.5 7.0 6.5 6.0 5.5 5.0 4.5 4.0 3.5 3.0 2.5 2.0 1.5 1.0 0.5 0.0
f1 (ppm)

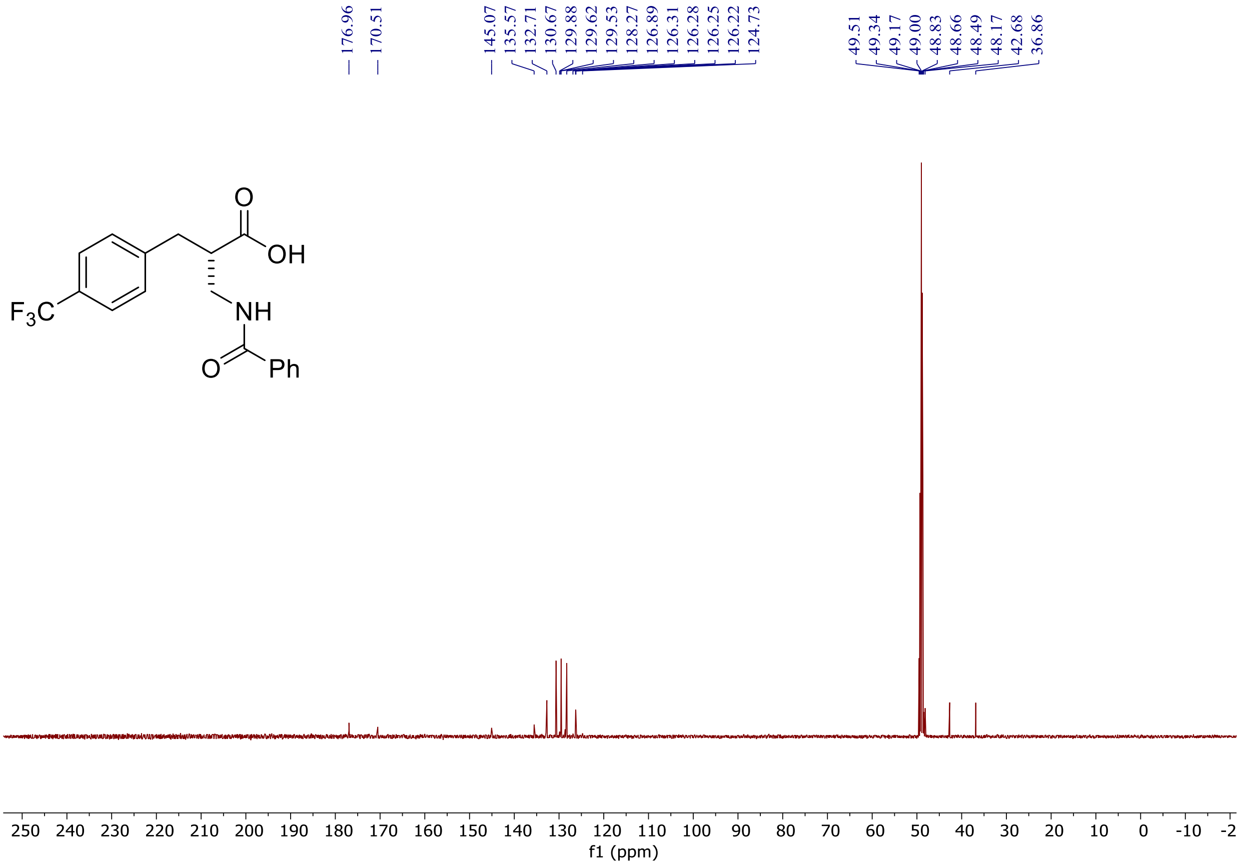
176.96
170.51
145.07
135.57
132.71
130.67
129.88
129.62
129.53
128.27
126.89
126.31
126.28
126.25
126.22
124.73
49.51
49.34
49.17
49.00
48.83
48.66
48.49
48.17
42.68
36.86
O
OH
F_3C
NH
O
Ph
250 240 230 220 210 200 190 180 170 160 150 140 130 120 110 100 90 80 70 60 50 40 30 20 10 0 -10 -2
f1 (ppm)

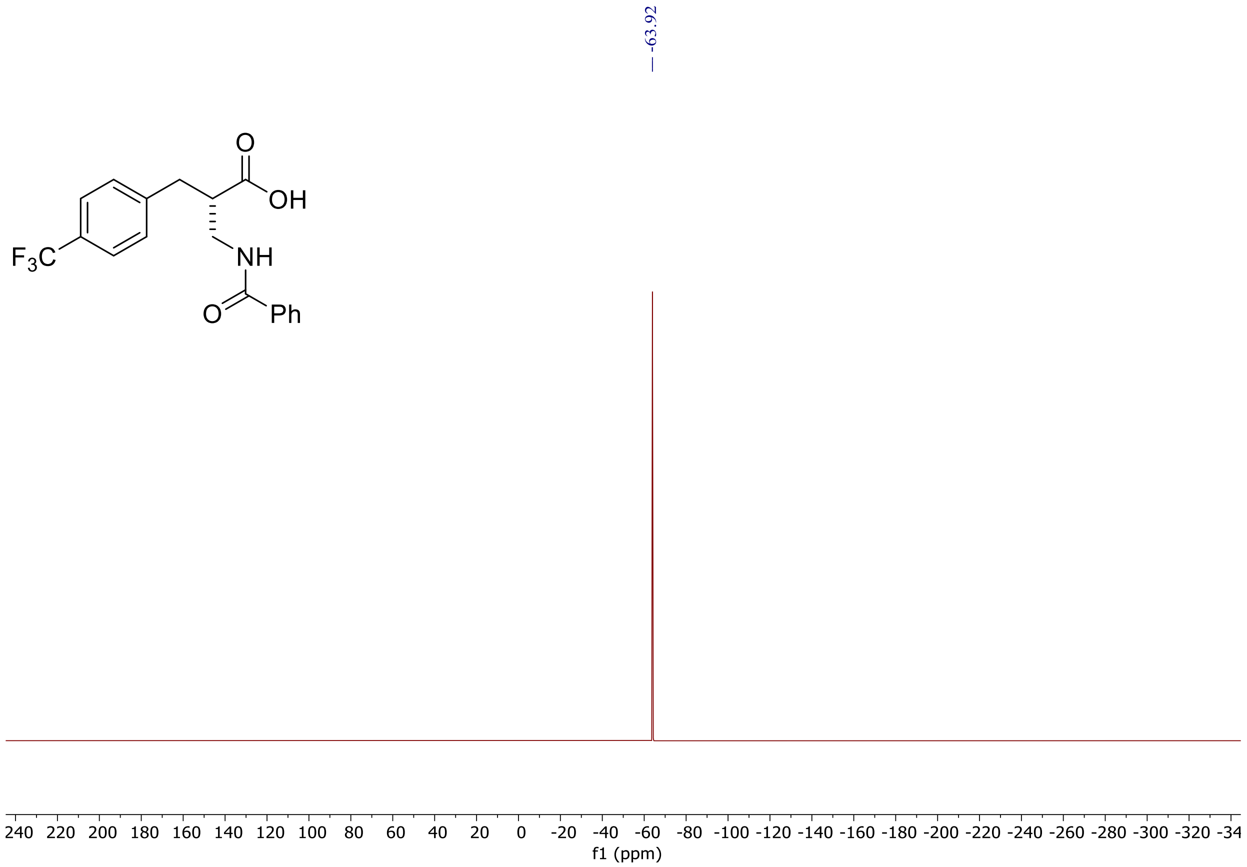
-63.92
OH
O
F3C
NH
O
Ph
240 220 200 180 160 140 120 100 80 60 40 20 0 -20 -40 -60 -80 -100 -120 -140 -160 -180 -200 -220 -240 -260 -280 -300 -320 -34
f1 (ppm)

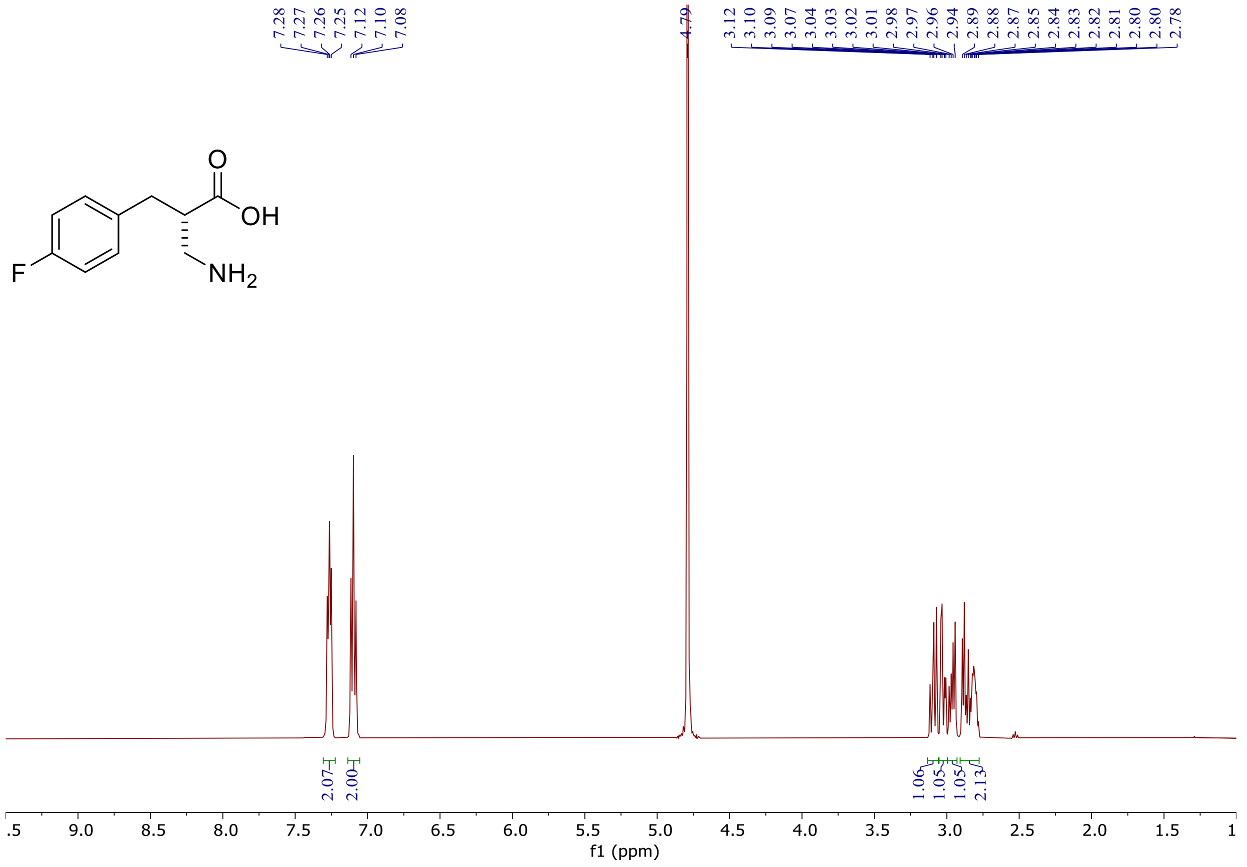
7.28
7.27
7.26
7.25
7.12
7.10
7.08
4.79
3.12
3.10
3.09
3.07
3.04
3.03
3.02
3.01
2.98
2.97
2.96
2.94
2.89
2.88
2.87
2.85
2.84
2.83
2.82
2.81
2.80
2.80
2.78
O
OH
F
NH2
2.07
2.00
1.06
1.05
1.05
2.13
.5 9.0 8.5 8.0 7.5 7.0 6.5 6.0 5.5 5.0 4.5 4.0 3.5 3.0 2.5 2.0 1.5 1
f1 (ppm)

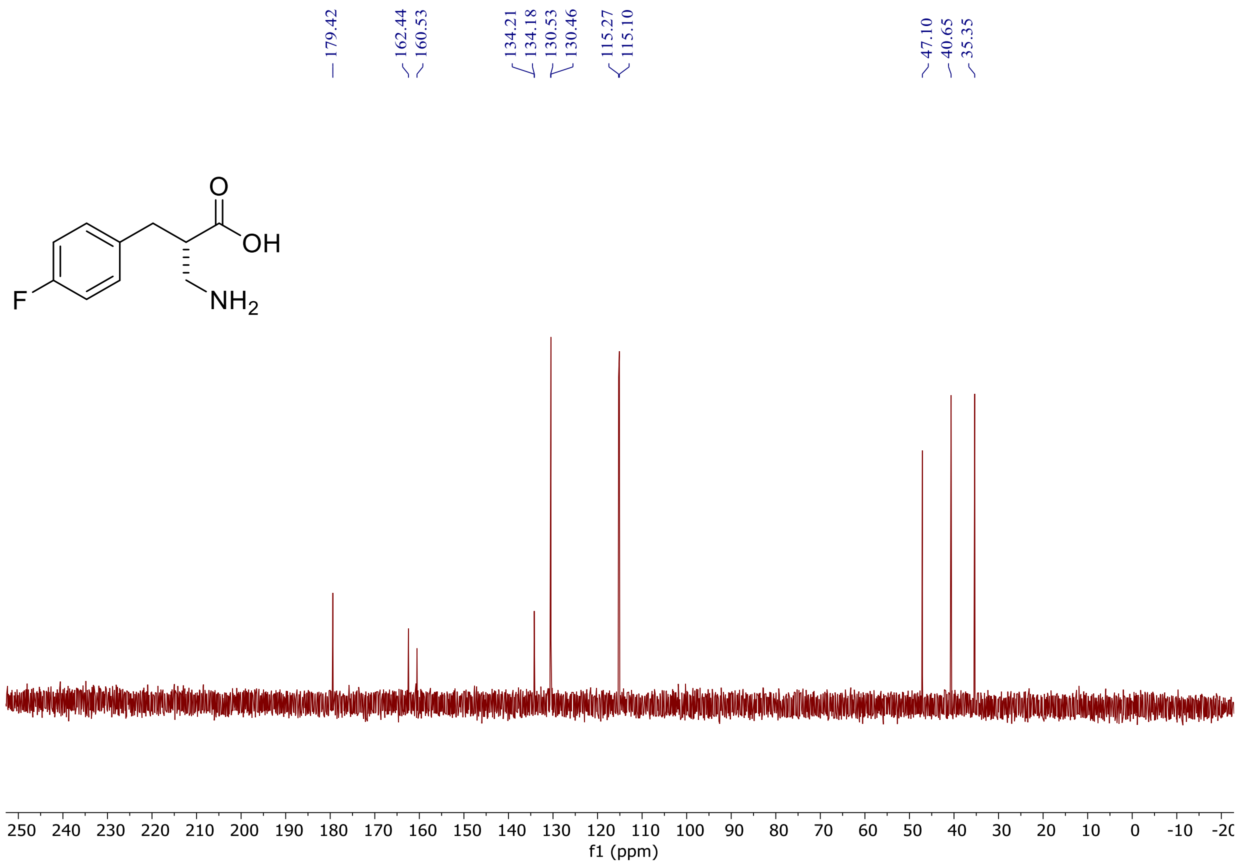
179.42
162.44
160.53
134.21
134.18
130.53
130.46
115.27
115.10
47.10
40.65
35.35
O
OH
F
NH2
250 240 230 220 210 200 190 180 170 160 150 140 130 120 110 100 90 80 70 60 50 40 30 20 10 0 -10 -20
f1 (ppm)

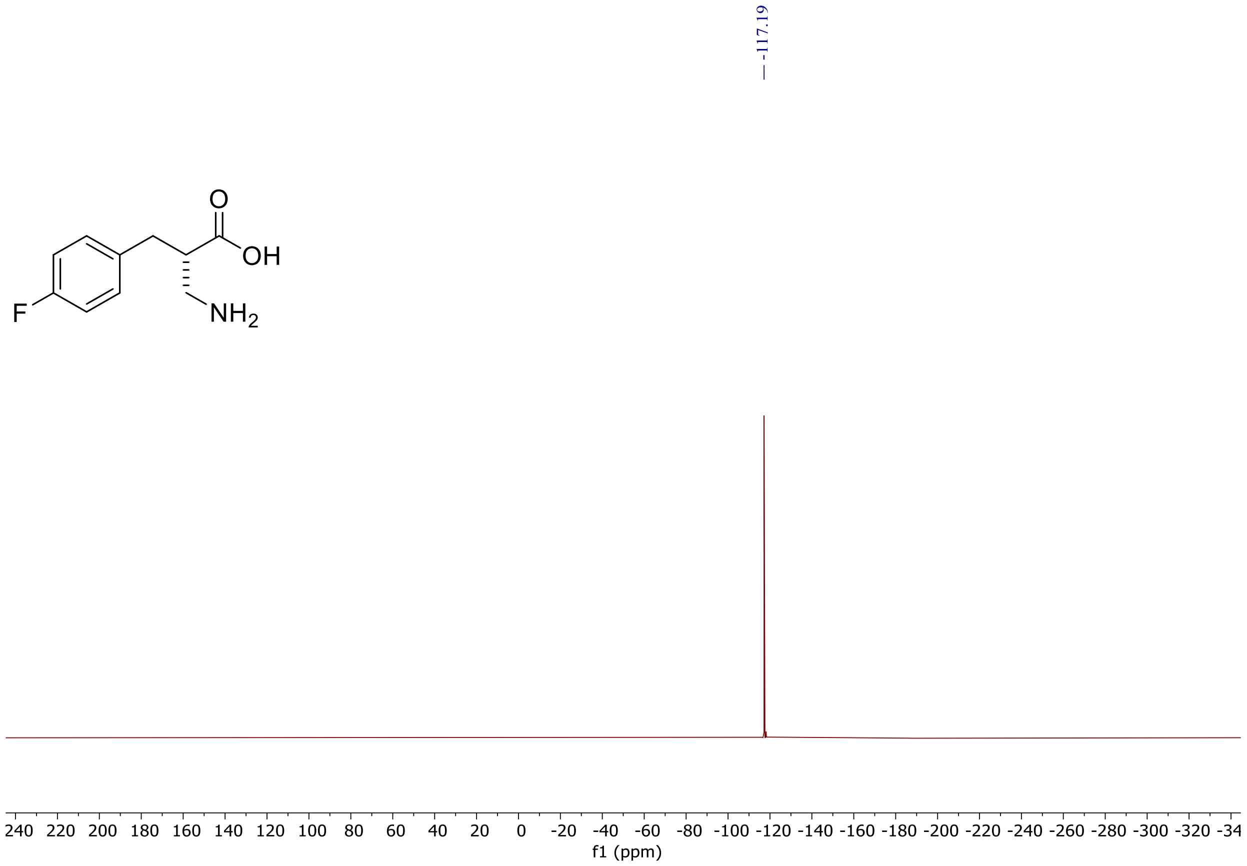
-117.19
O
OH
F
NH2
240 220 200 180 160 140 120 100 80 60 40 20 0 -20 -40 -60 -80 -100 -120 -140 -160 -180 -200 -220 -240 -260 -280 -300 -320 -34
f1 (ppm)

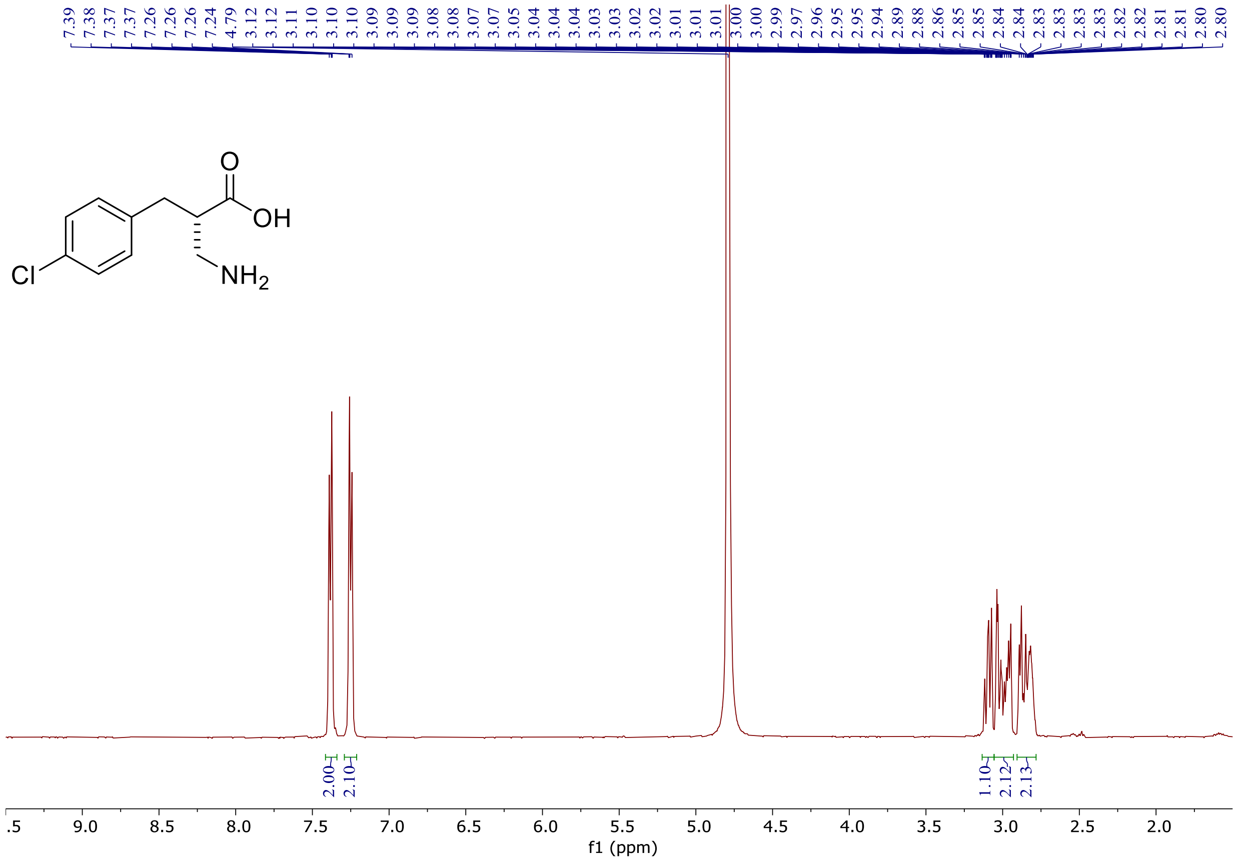
7.39 7.38 7.37 7.37 7.26 7.26 7.26 7.24 4.79 3.12 3.12 3.11 3.10 3.10 3.10 3.09 3.09 3.09 3.08 3.08 3.07 3.07 3.05 3.04 3.04 3.04 3.03 3.03 3.02 3.02 3.01 3.01 3.01 3.00 3.00 2.99 2.97 2.96 2.95 2.95 2.94 2.89 2.88 2.86 2.85 2.85 2.84 2.84 2.83 2.83 2.83 2.83 2.82 2.82 2.81 2.81 2.80 2.80
O
OH
Cl
NH_2
2.00
2.10
1.10
2.12
2.13
.5 9.0 8.5 8.0 7.5 7.0 6.5 6.0 5.5 5.0 4.5 4.0 3.5 3.0 2.5 2.0
f1 (ppm)

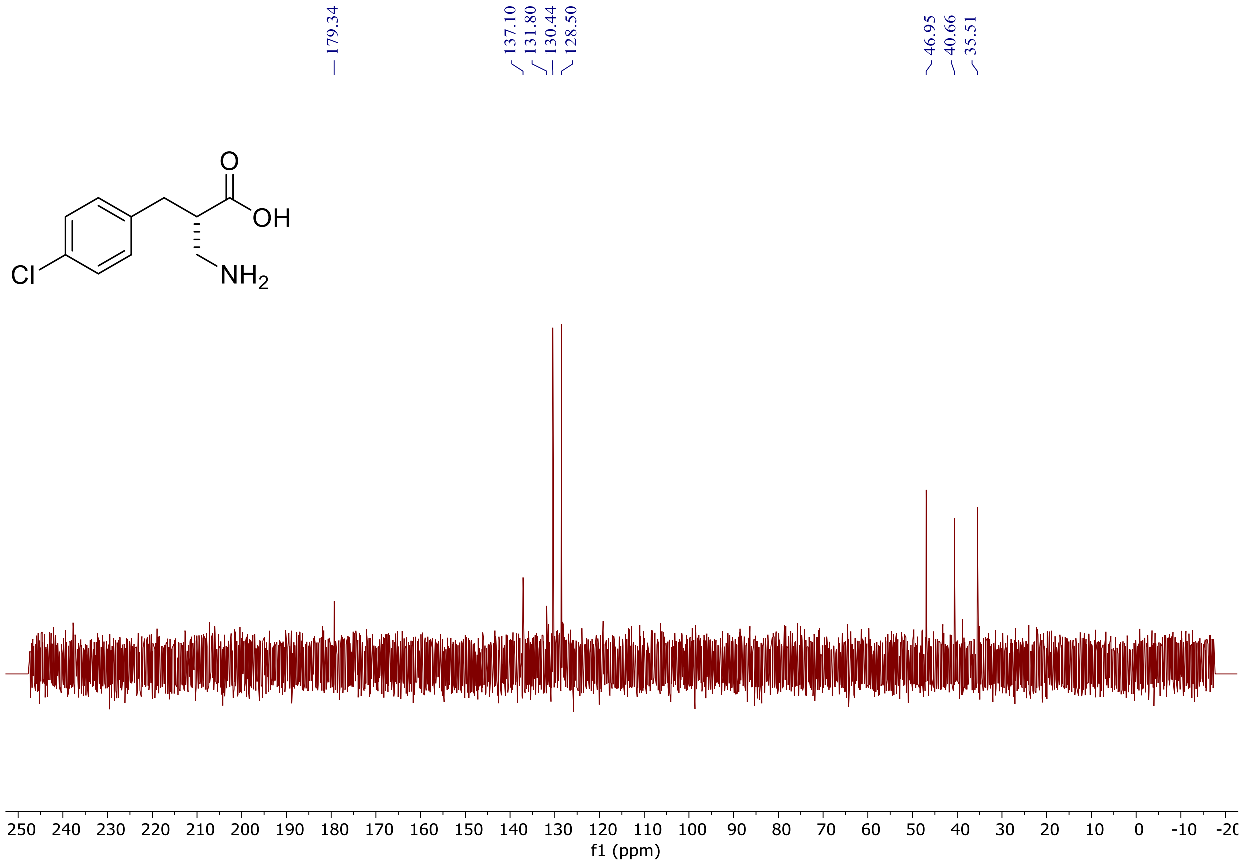
179.34
137.10 131.80 130.44 128.50
46.95 40.66 35.51
O
OH
Cl
NH_2
250 240 230 220 210 200 190 180 170 160 150 140 130 120 110 100 90 80 70 60 50 40 30 20 10 0 -10 -20
f1 (ppm)

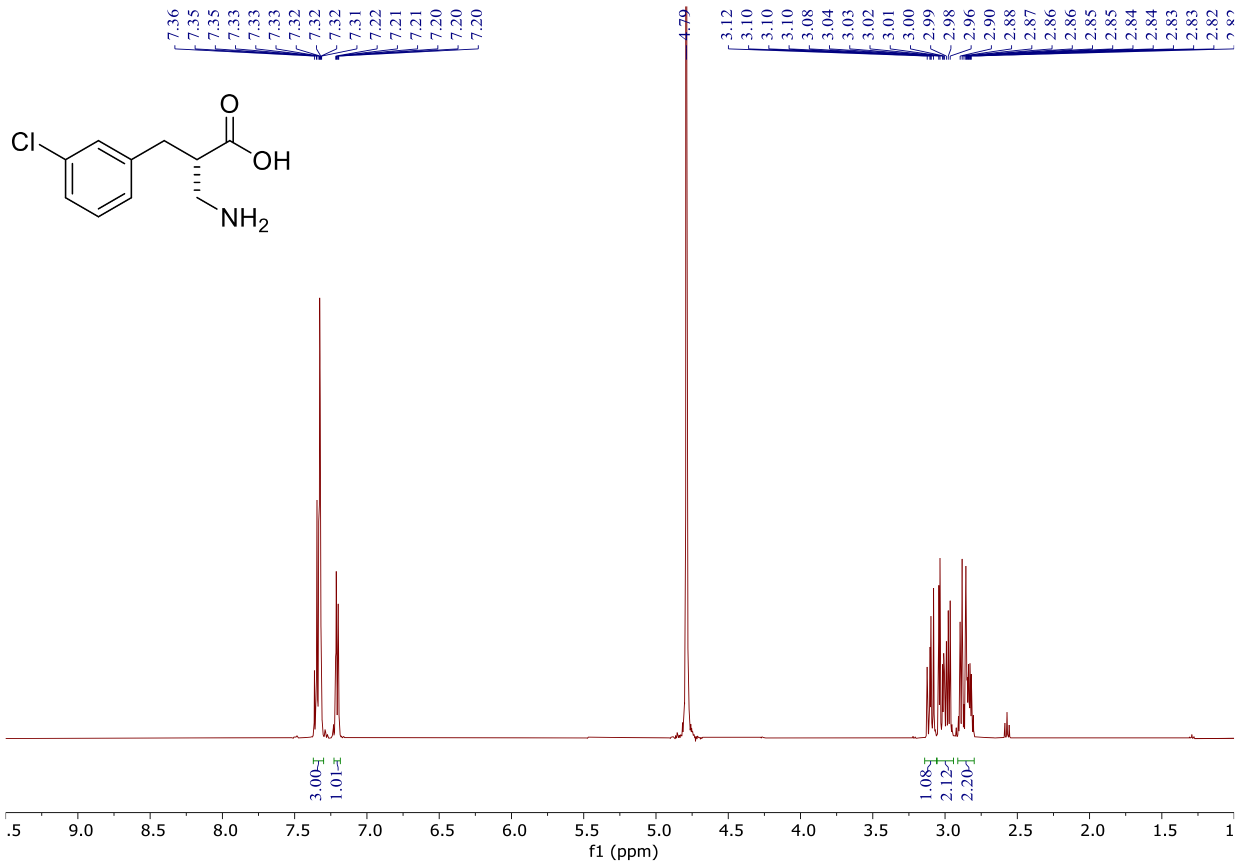
7.36 7.35 7.35 7.33 7.33 7.33 7.32 7.32 7.32 7.31 7.22 7.21 7.21 7.20 7.20 7.20
4.79
3.12 3.10 3.10 3.10 3.08 3.04 3.03 3.02 3.01 3.00 2.99 2.98 2.96 2.90 2.88 2.87 2.86 2.86 2.85 2.85 2.84 2.84 2.83 2.83 2.82
Cl
O
OH
NH2
3.00
1.01
1.08
2.12
2.20
f1 (ppm)

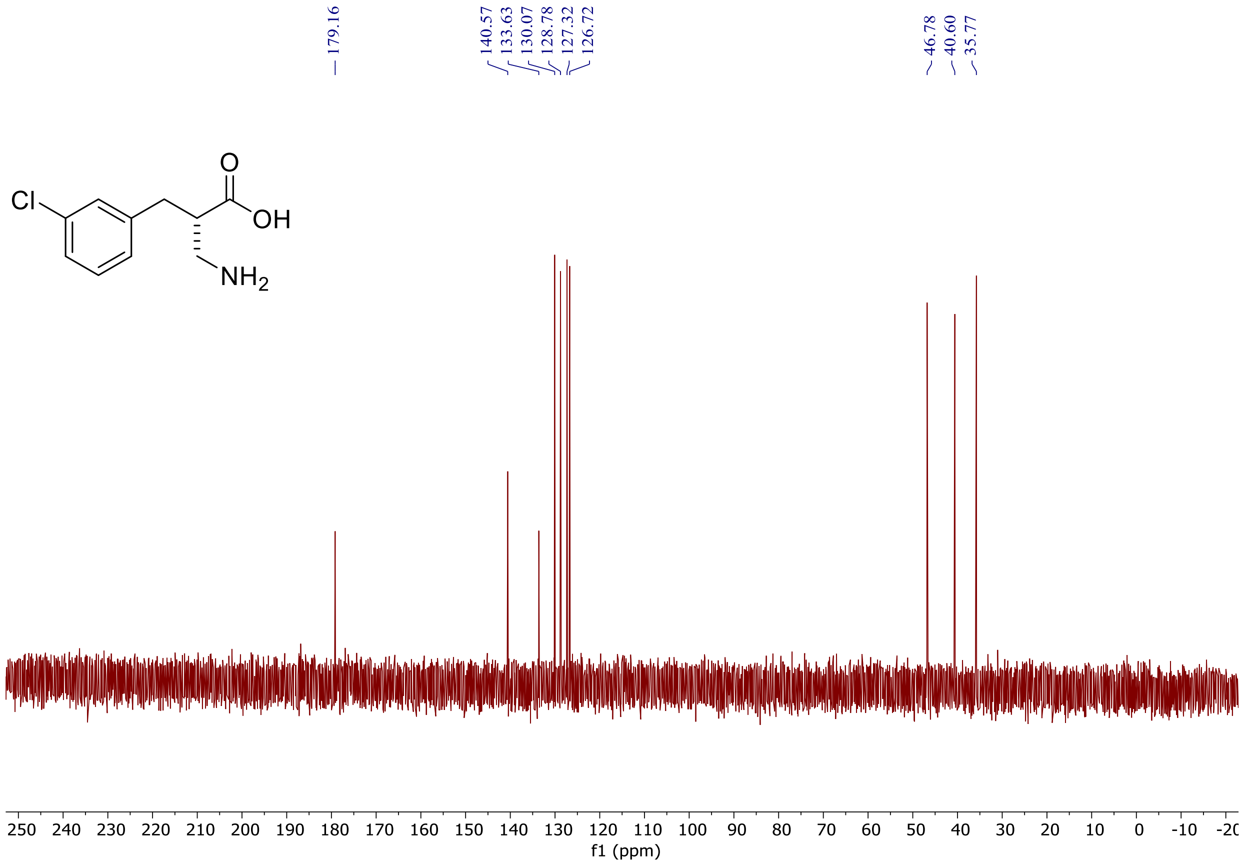
179.16
140.57 133.63 130.07 128.78 127.32 126.72
46.78 40.60 35.77
Cl
O
OH
NH2
f1 (ppm)

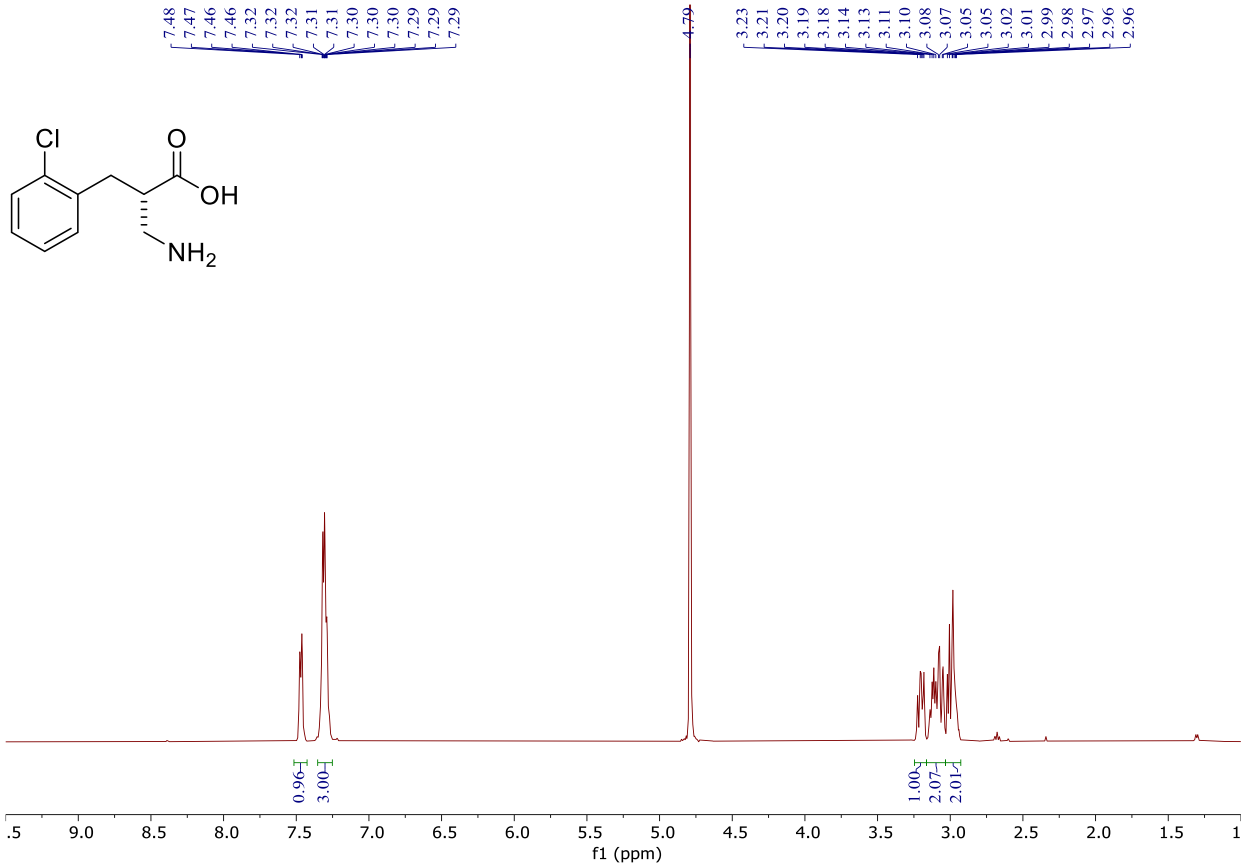
7.48 7.47 7.46 7.46 7.32 7.32 7.31 7.31 7.30 7.30 7.30 7.29 7.29 7.29
4.79
3.23 3.21 3.20 3.19 3.18 3.14 3.13 3.11 3.10 3.08 3.07 3.05 3.05 3.02 3.01 2.99 2.98 2.97 2.96 2.96
Cl
O
OH
NH2
0.96
3.00
1.00
2.07
2.01
f1 (ppm)

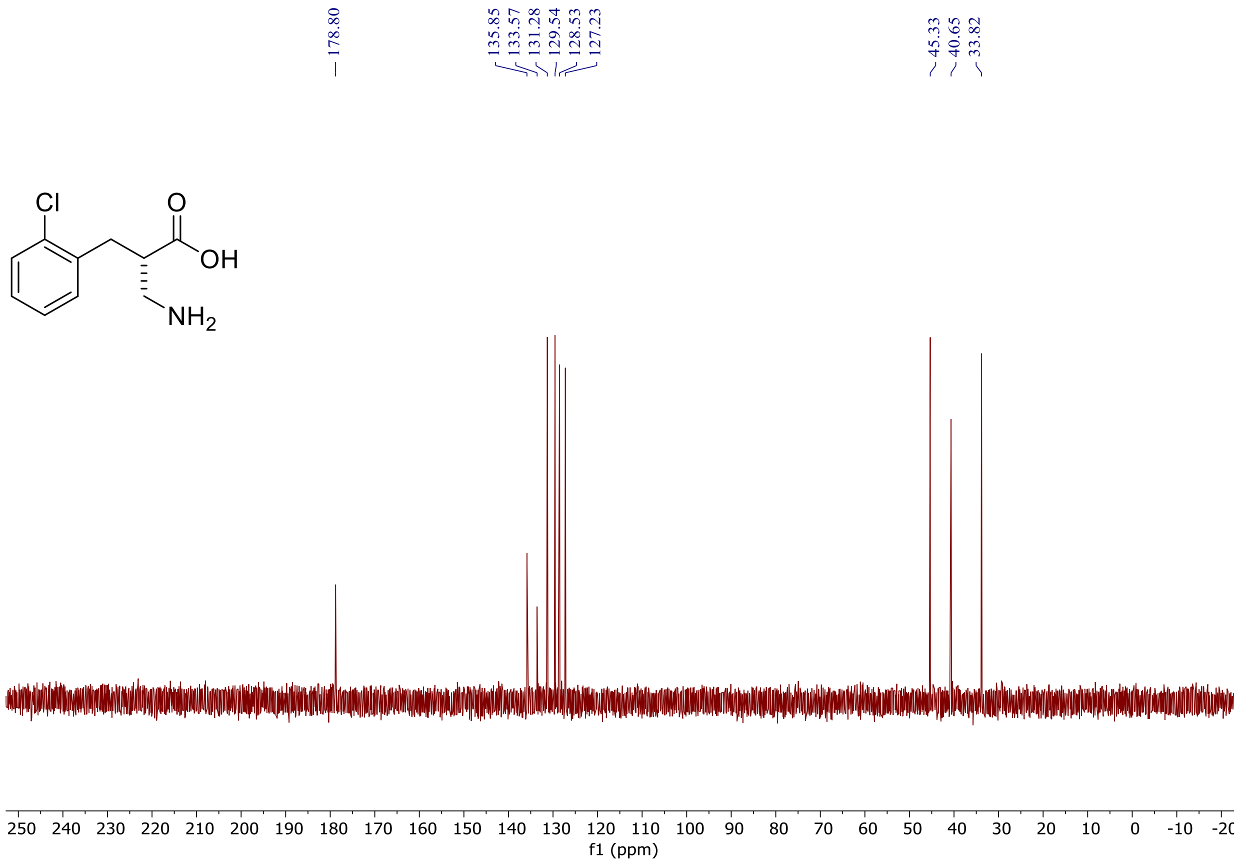
178.80
135.85 133.57 131.28 129.54 128.53 127.23
45.33 40.65 33.82
Cl
O
OH
NH2
f1 (ppm)

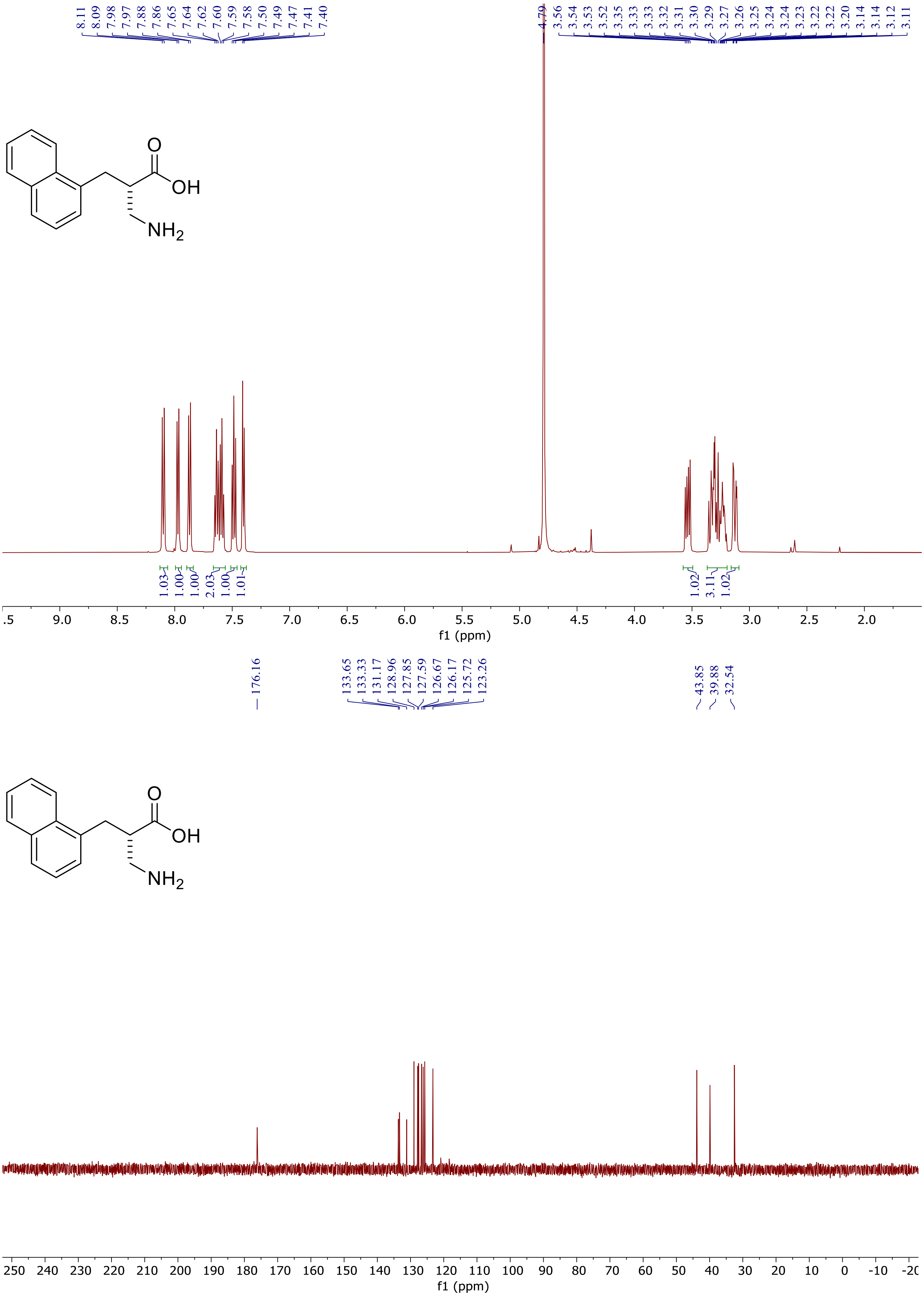

8.11 8.09 7.98 7.97 7.88 7.86 7.65 7.64 7.62 7.60 7.59 7.58 7.50 7.49 7.47 7.41 7.40
4.79 3.56 3.54 3.53 3.52 3.35 3.33 3.33 3.32 3.31 3.30 3.29 3.27 3.26 3.25 3.24 3.24 3.23 3.22 3.22 3.20 3.14 3.14 3.12 3.11
O
OH
NH2
1.03 1.00 1.00 2.03 1.00 1.01
1.02 3.11 1.02
f1 (ppm)
176.16
133.65 133.33 131.17 128.96 127.85 127.59 126.67 126.17 125.72 123.26
43.85 39.88 32.54
f1 (ppm)

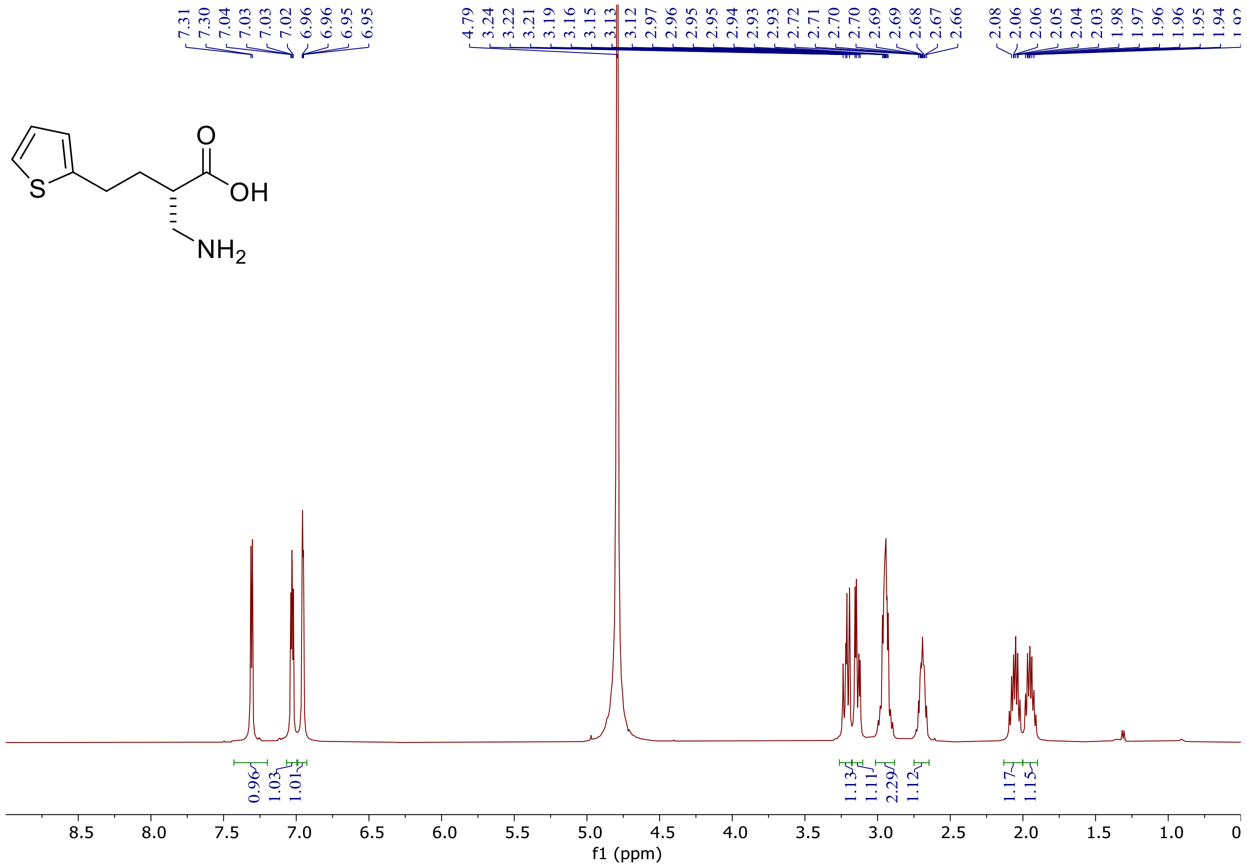

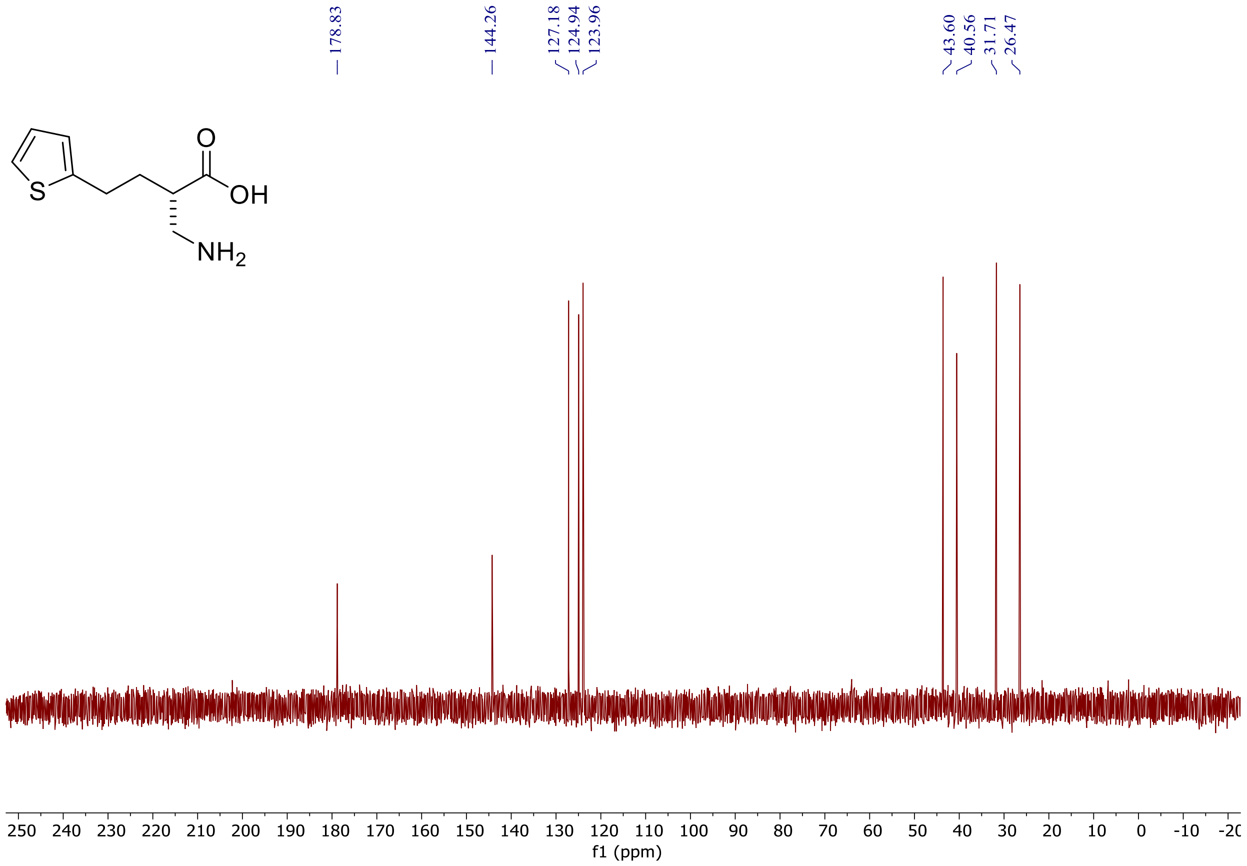

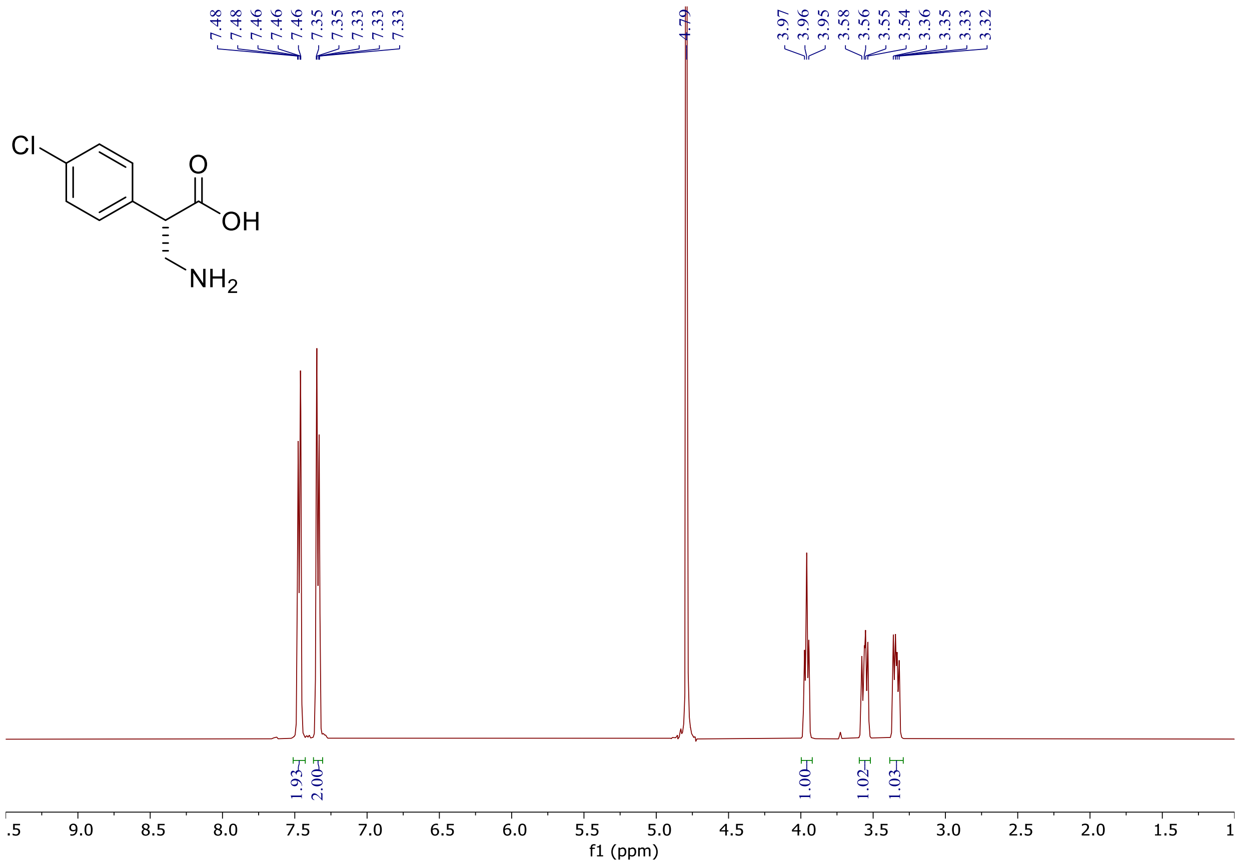
7.48
7.48
7.46
7.46
7.46
7.35
7.35
7.33
7.33
7.33
4.79
3.97
3.96
3.95
3.58
3.56
3.55
3.54
3.36
3.35
3.33
3.32
Cl
O
OH
NH2
1.93
2.00
1.00
1.02
1.03
f1 (ppm)

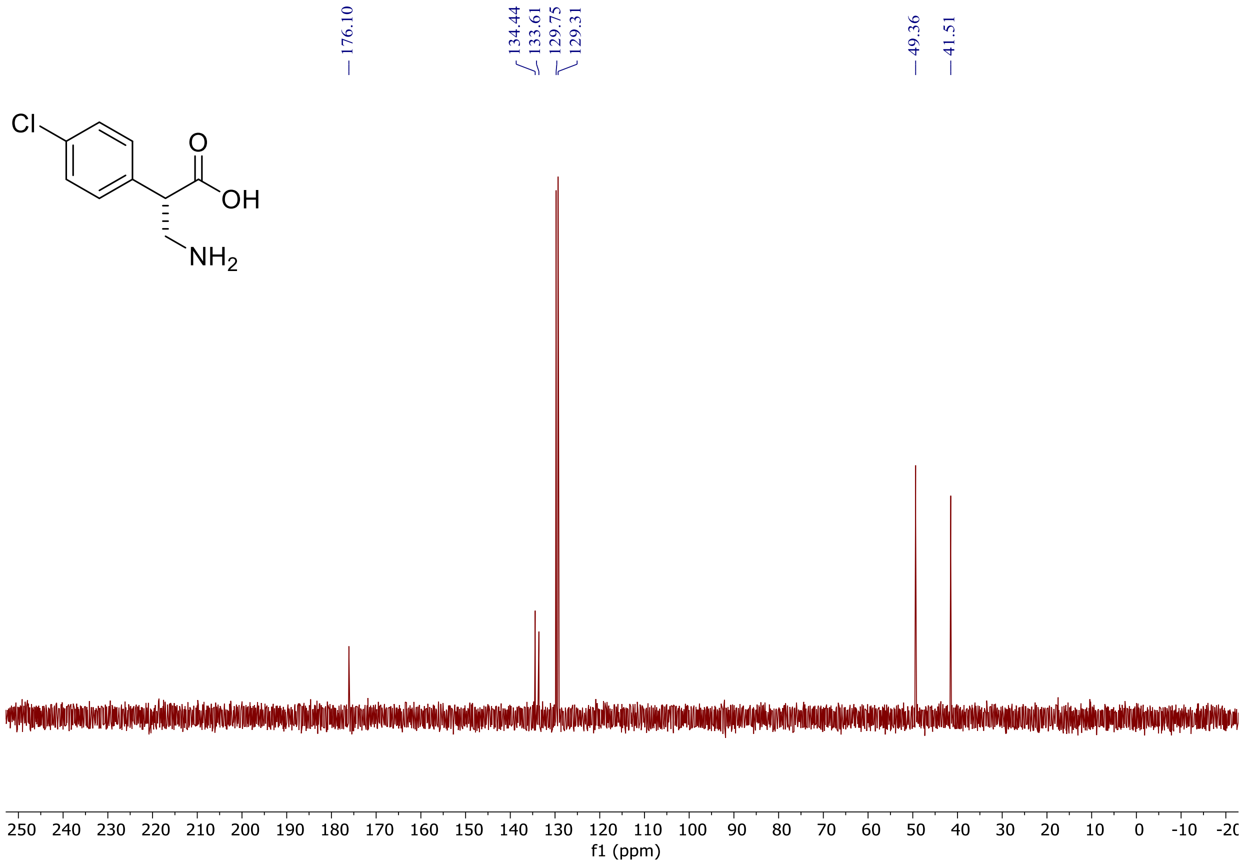
176.10
134.44
133.61
129.75
129.31
49.36
41.51
Cl
O
OH
NH2
f1 (ppm)

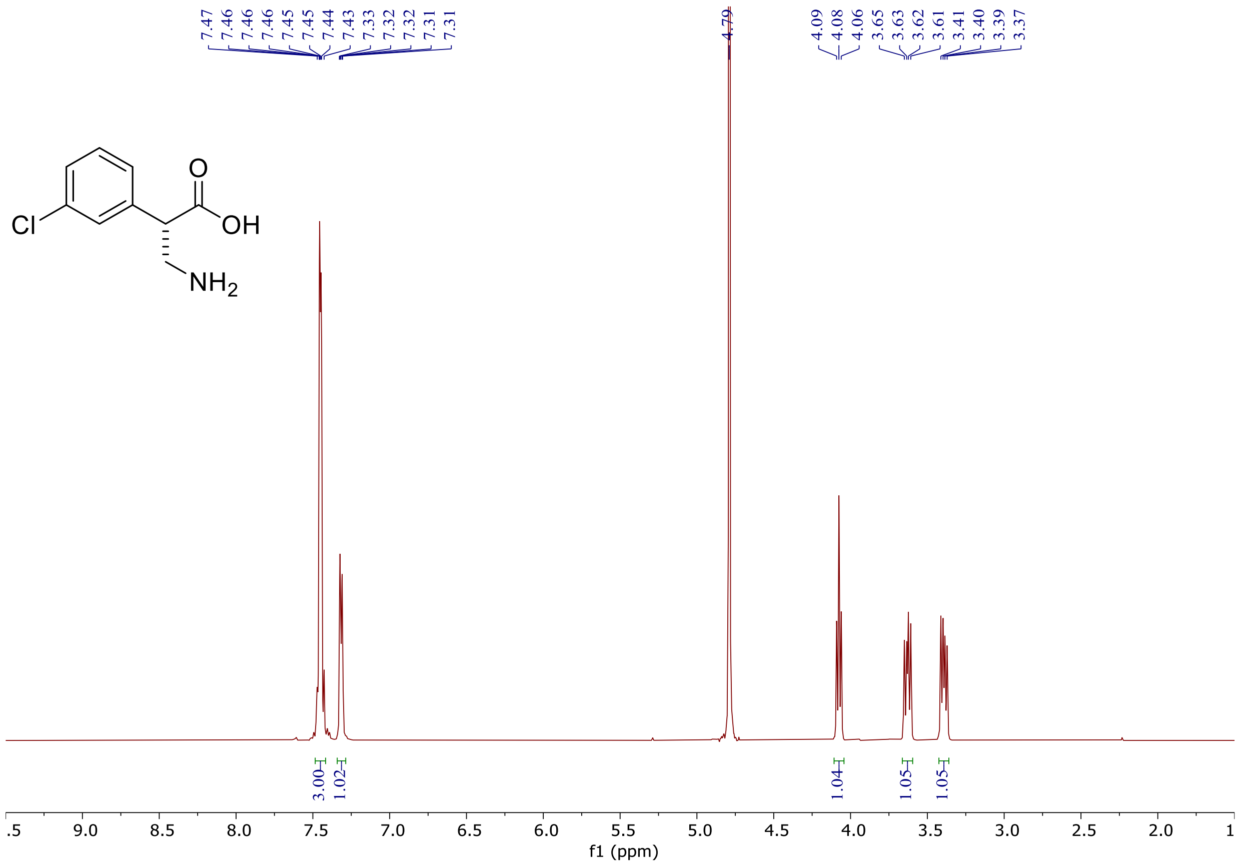
7.47
7.46
7.46
7.46
7.45
7.45
7.44
7.43
7.33
7.32
7.32
7.31
7.31
4.79
4.09
4.08
4.06
3.65
3.63
3.62
3.61
3.41
3.40
3.39
3.37
Cl
O
OH
NH2
3.00
1.02
1.04
1.05
1.05
f1 (ppm)

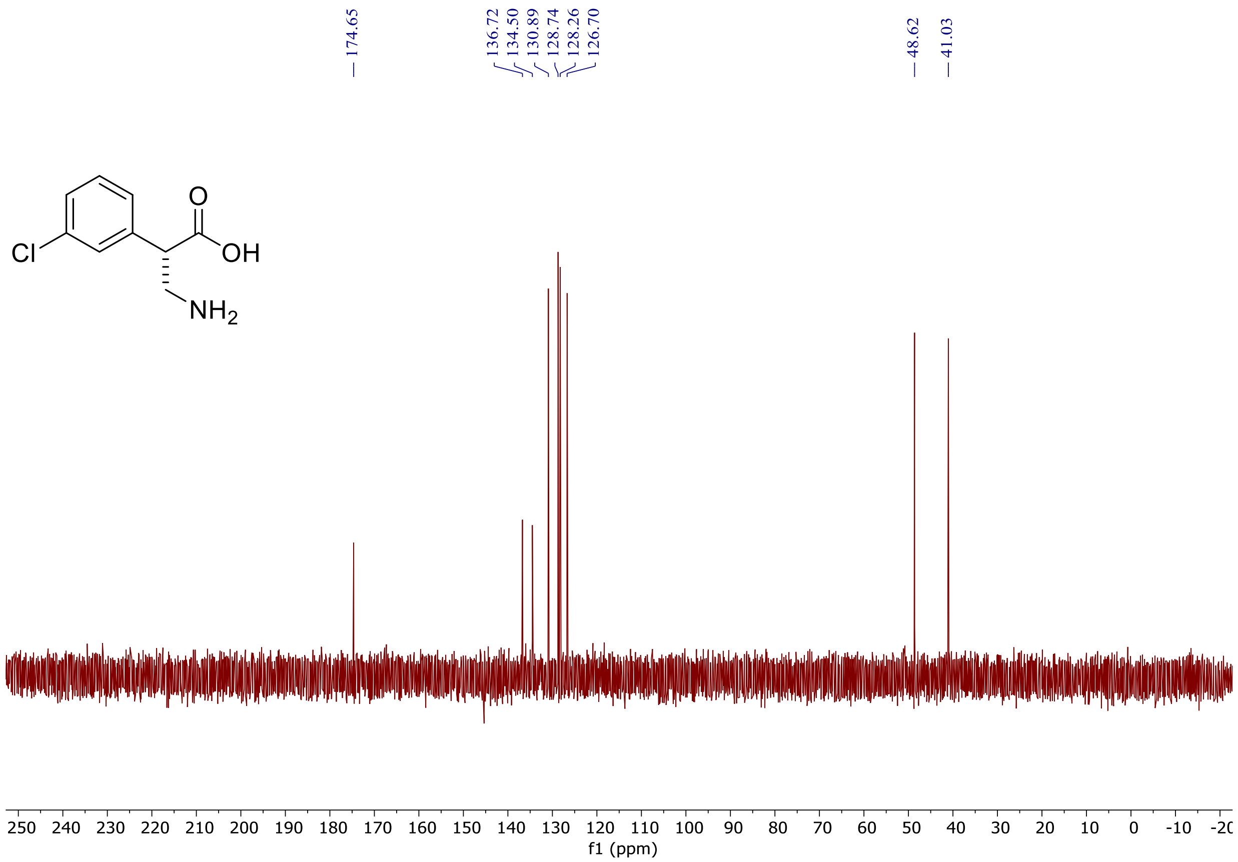
174.65
136.72
134.50
130.89
128.74
128.26
126.70
48.62
41.03
Cl
O
OH
NH2
f1 (ppm)

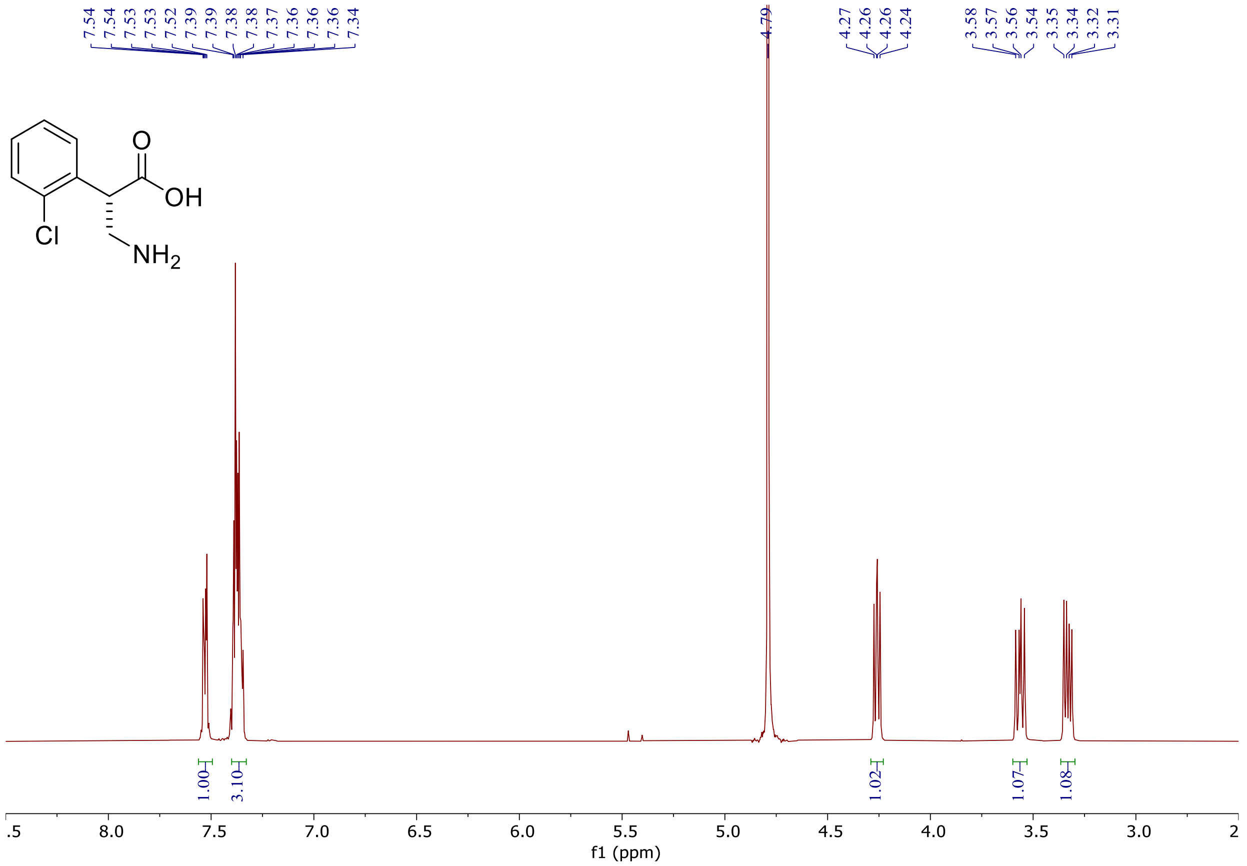
7.54
7.54
7.53
7.53
7.52
7.39
7.38
7.38
7.37
7.36
7.36
7.36
7.34
4.79
4.27
4.26
4.26
4.24
3.58
3.57
3.56
3.54
3.35
3.34
3.32
3.31
O
OH
Cl
NH2
1.00
3.10
1.02
1.07
1.08
f1 (ppm)

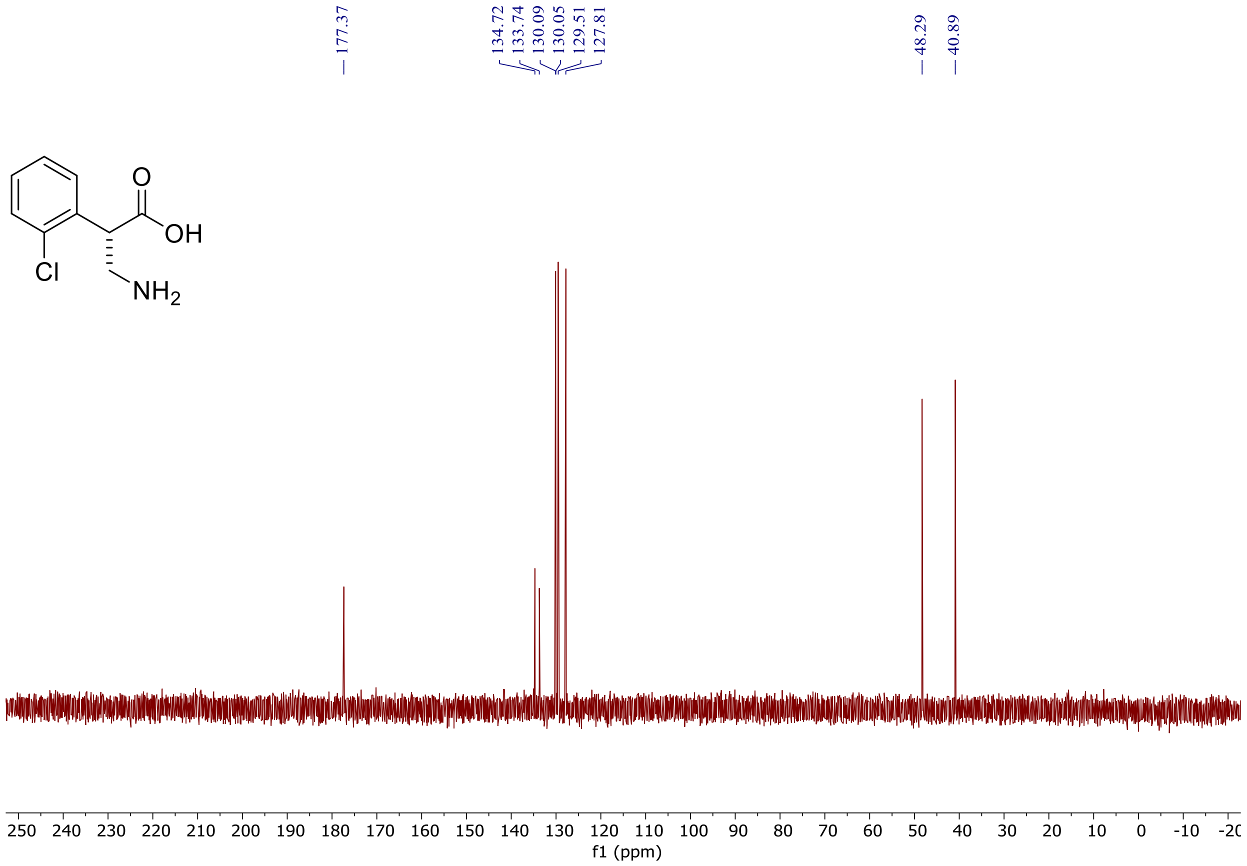
177.37
134.72
133.74
130.09
130.05
129.51
127.81
48.29
40.89
O
OH
Cl
NH2
f1 (ppm)

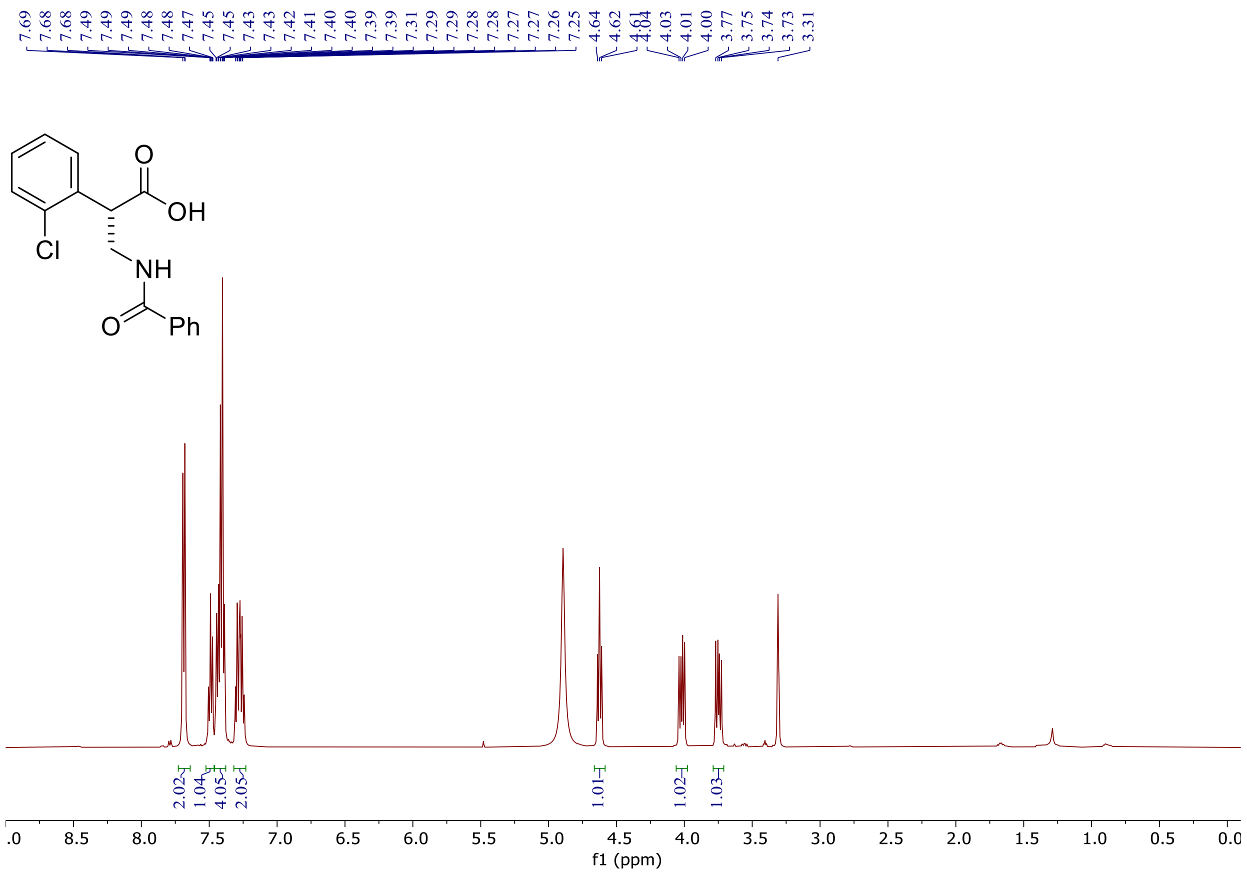
7.69
7.68
7.68
7.49
7.49
7.49
7.48
7.48
7.47
7.45
7.45
7.43
7.43
7.42
7.41
7.40
7.40
7.39
7.39
7.31
7.29
7.29
7.28
7.28
7.27
7.27
7.26
7.25
4.64
4.62
4.61
4.04
4.03
4.01
4.00
3.77
3.75
3.74
3.73
3.31
O
OH
Cl
NH
O
Ph
2.02
1.04
4.05
2.05
1.01
1.02
1.03
f1 (ppm)

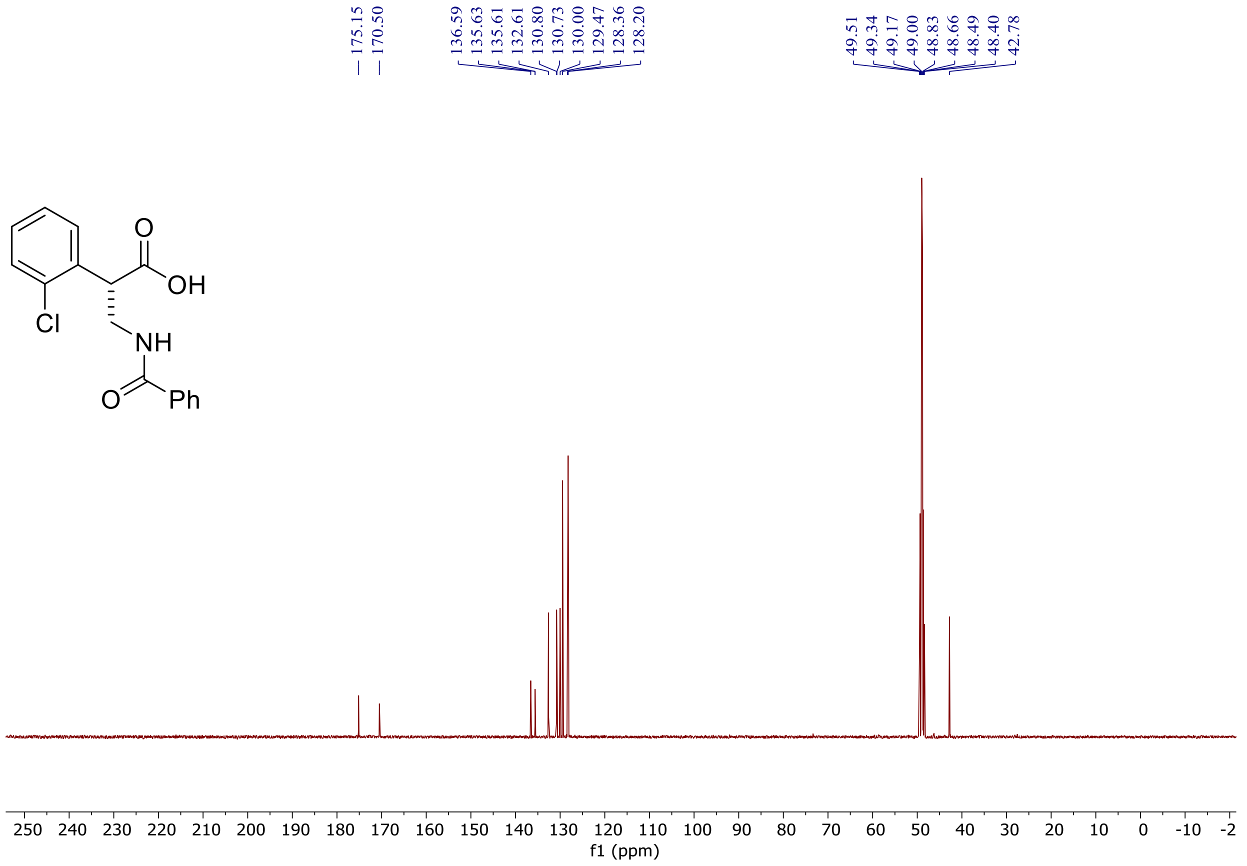
175.15
170.50
136.59
135.63
135.61
132.61
130.80
130.73
130.00
129.47
128.36
128.20
49.51
49.34
49.17
49.00
48.83
48.66
48.49
48.40
42.78
O
OH
Cl
NH
O
Ph
f1 (ppm)

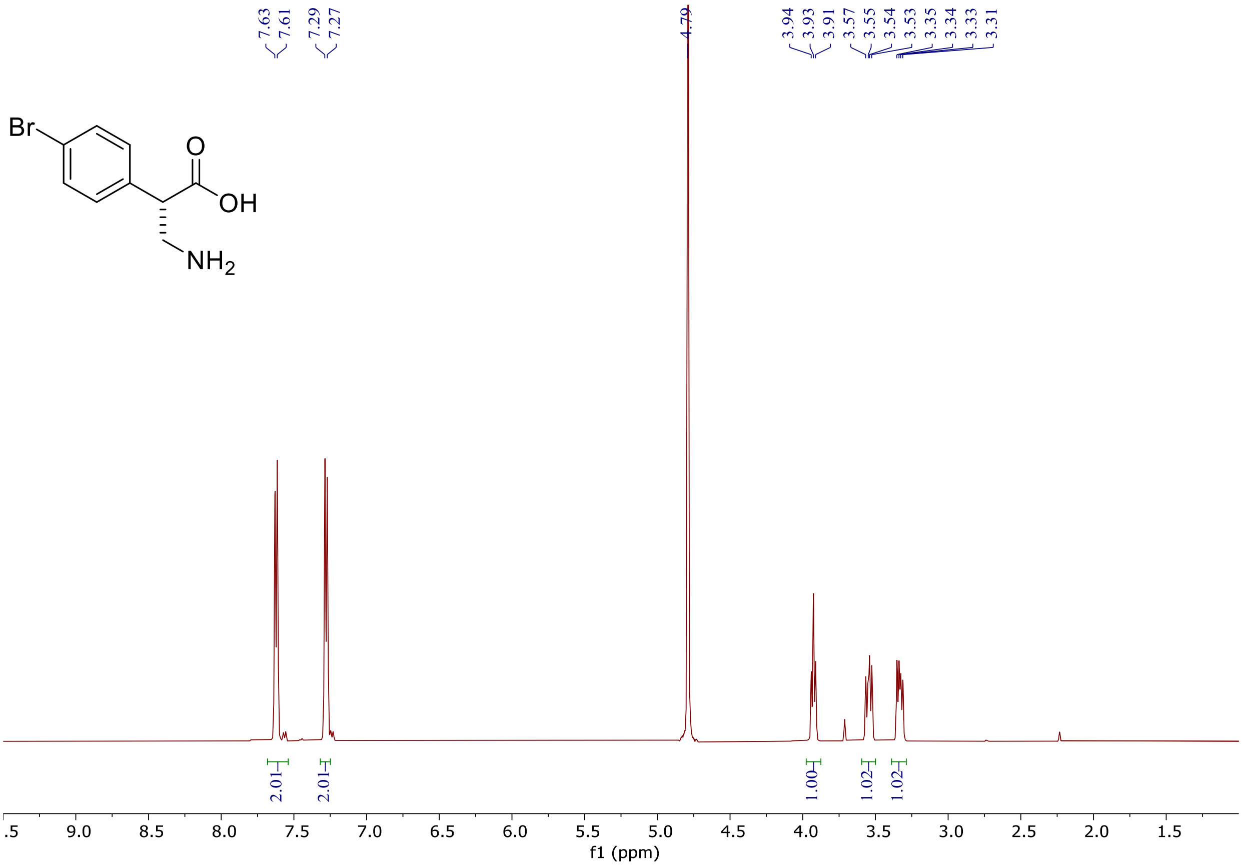
7.63
7.61
7.29
7.27
4.79
3.94
3.93
3.91
3.57
3.55
3.54
3.53
3.35
3.34
3.33
3.31
Br
O
OH
NH2
2.01
2.01
1.00
1.02
1.02
f1 (ppm)

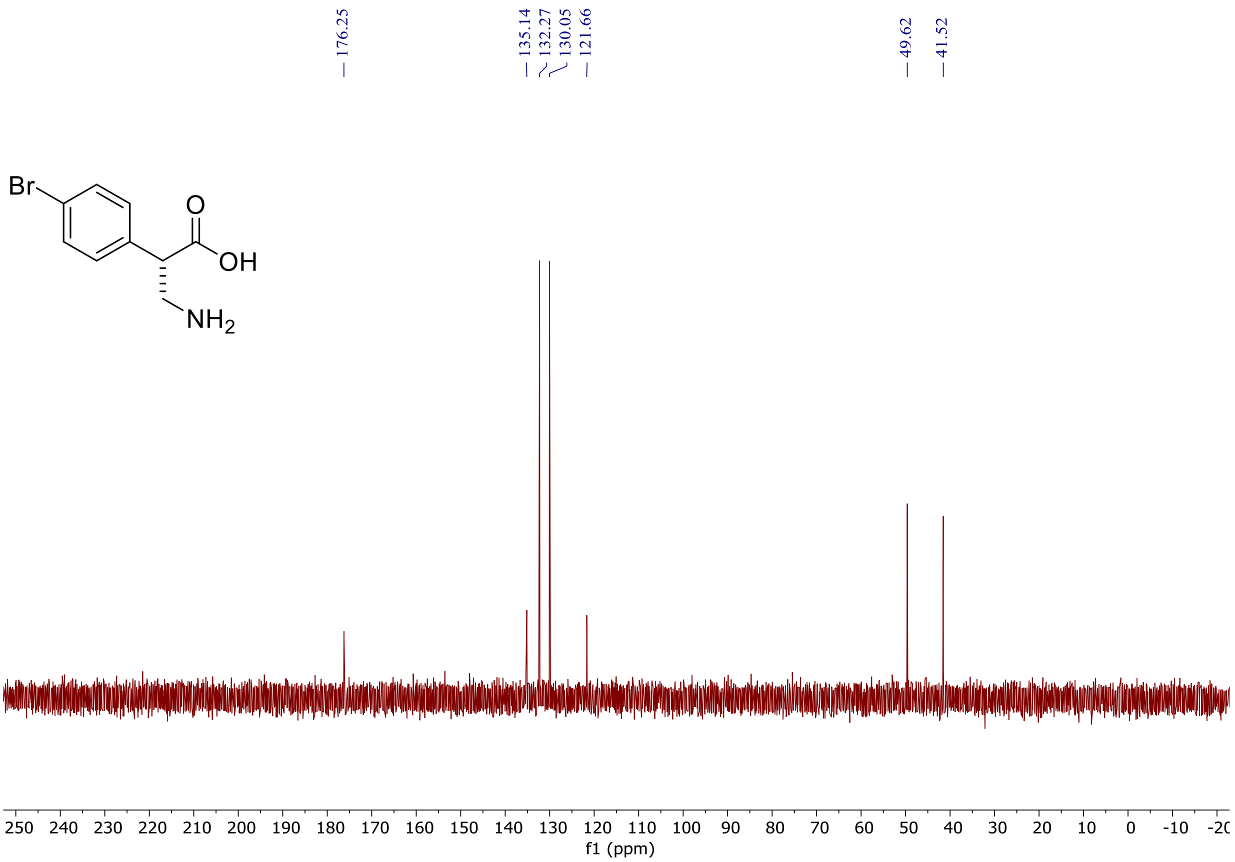
176.25
135.14
132.27
130.05
121.66
49.62
41.52
Br
O
OH
NH2
f1 (ppm)

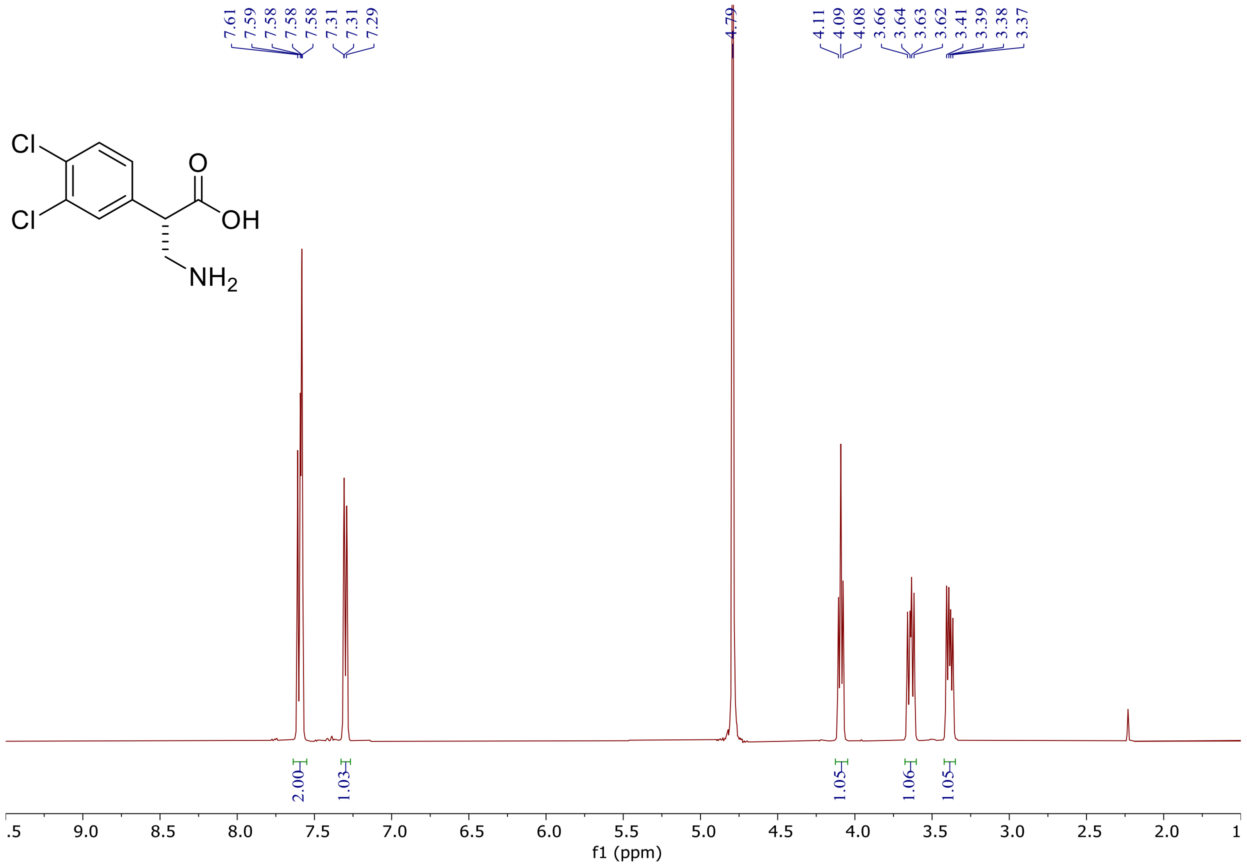
7.61
7.59
7.58
7.58
7.58
7.31
7.31
7.29
4.79
4.11
4.09
4.08
3.66
3.64
3.63
3.62
3.41
3.39
3.38
3.37
Cl
Cl
O
OH
NH2
2.00
1.03
1.05
1.06
1.05
f1 (ppm)

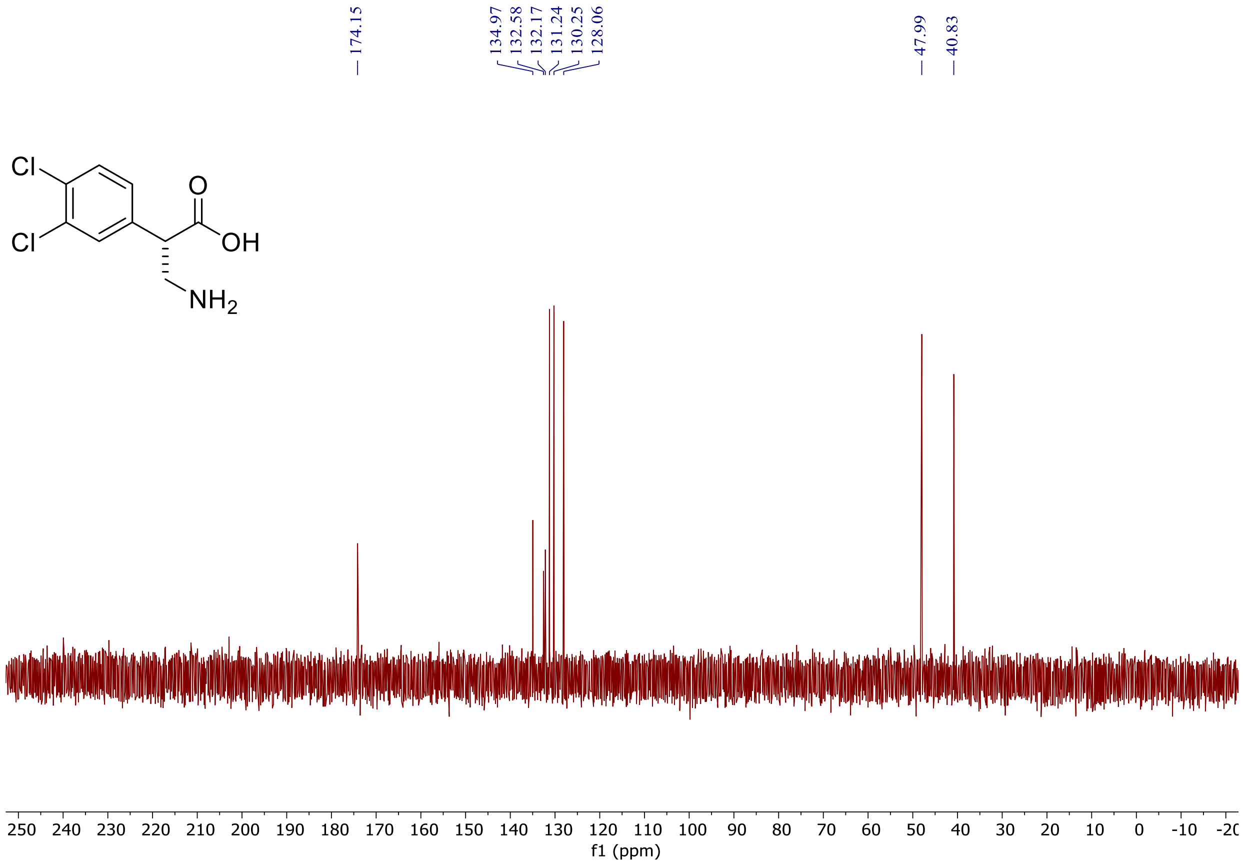
174.15
134.97
132.58
132.17
131.24
130.25
128.06
47.99
40.83
Cl
Cl
O
OH
NH2
f1 (ppm)

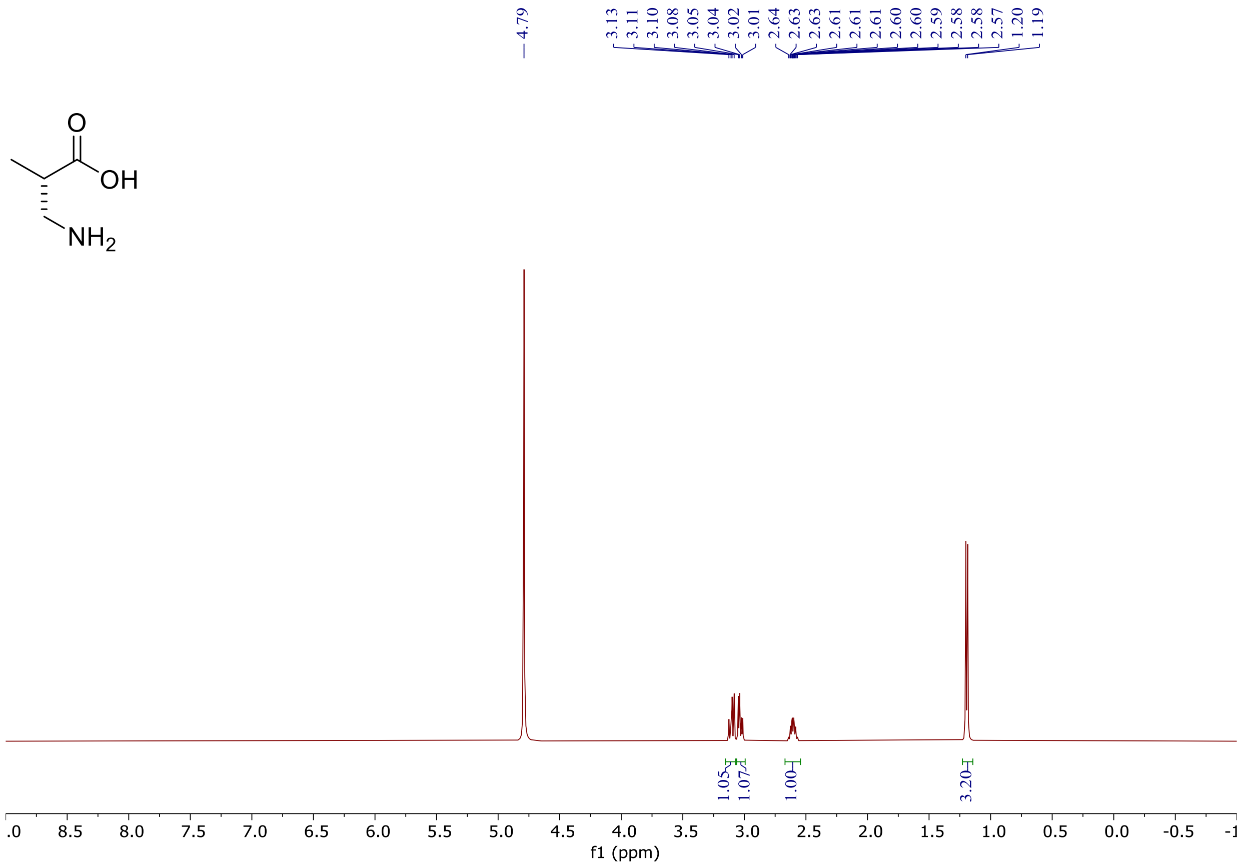
4.79
3.13 3.11 3.10 3.08 3.05 3.04 3.02 3.01 2.64 2.63 2.63 2.61 2.61 2.61 2.60 2.60 2.59 2.58 2.58 2.57 1.20 1.19
O
OH
NH2
1.05
1.07
1.00
3.20
f1 (ppm)

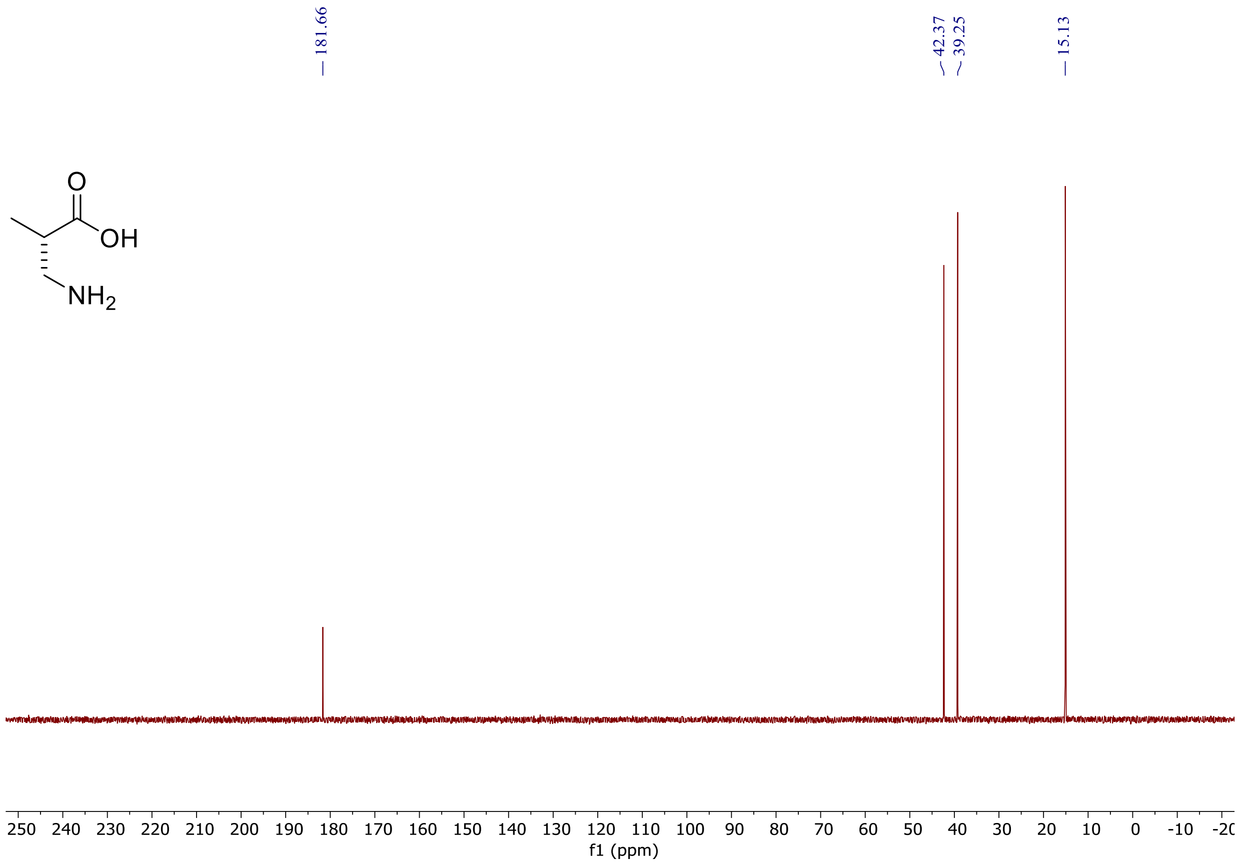
181.66
42.37
39.25
15.13
O
OH
NH2
f1 (ppm)

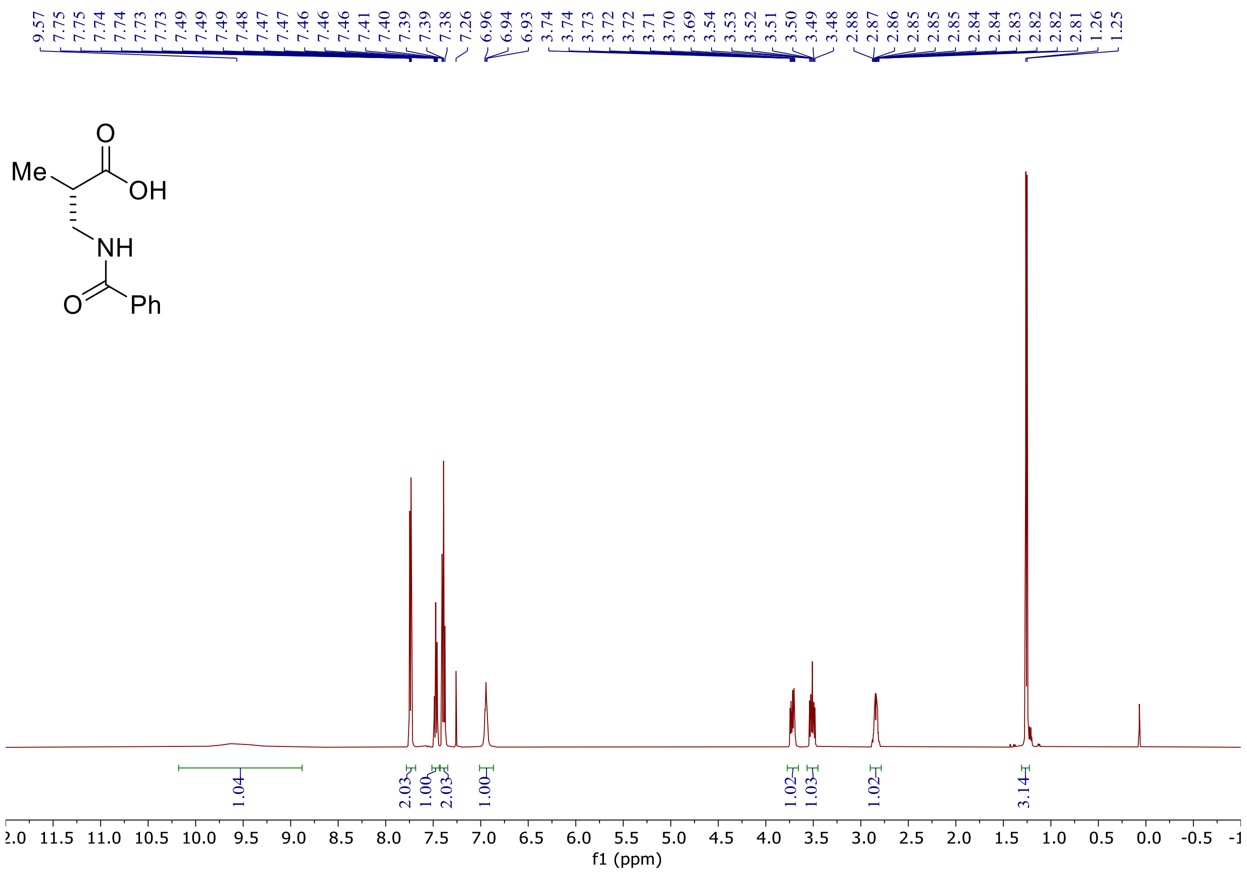

9.57
7.75
7.75
7.74
7.74
7.73
7.73
7.49
7.49
7.49
7.48
7.47
7.47
7.46
7.46
7.46
7.41
7.40
7.39
7.39
7.38
7.26
6.96
6.94
6.93
3.74
3.74
3.73
3.72
3.72
3.71
3.70
3.69
3.54
3.53
3.52
3.51
3.50
3.49
3.48
2.88
2.87
2.86
2.85
2.85
2.85
2.84
2.84
2.83
2.82
2.82
2.81
1.26
1.25
Me
O
OH
NH
O
Ph
1.04
2.03
1.00
2.03
1.00
1.02
1.03
1.02
3.14
f1 (ppm)

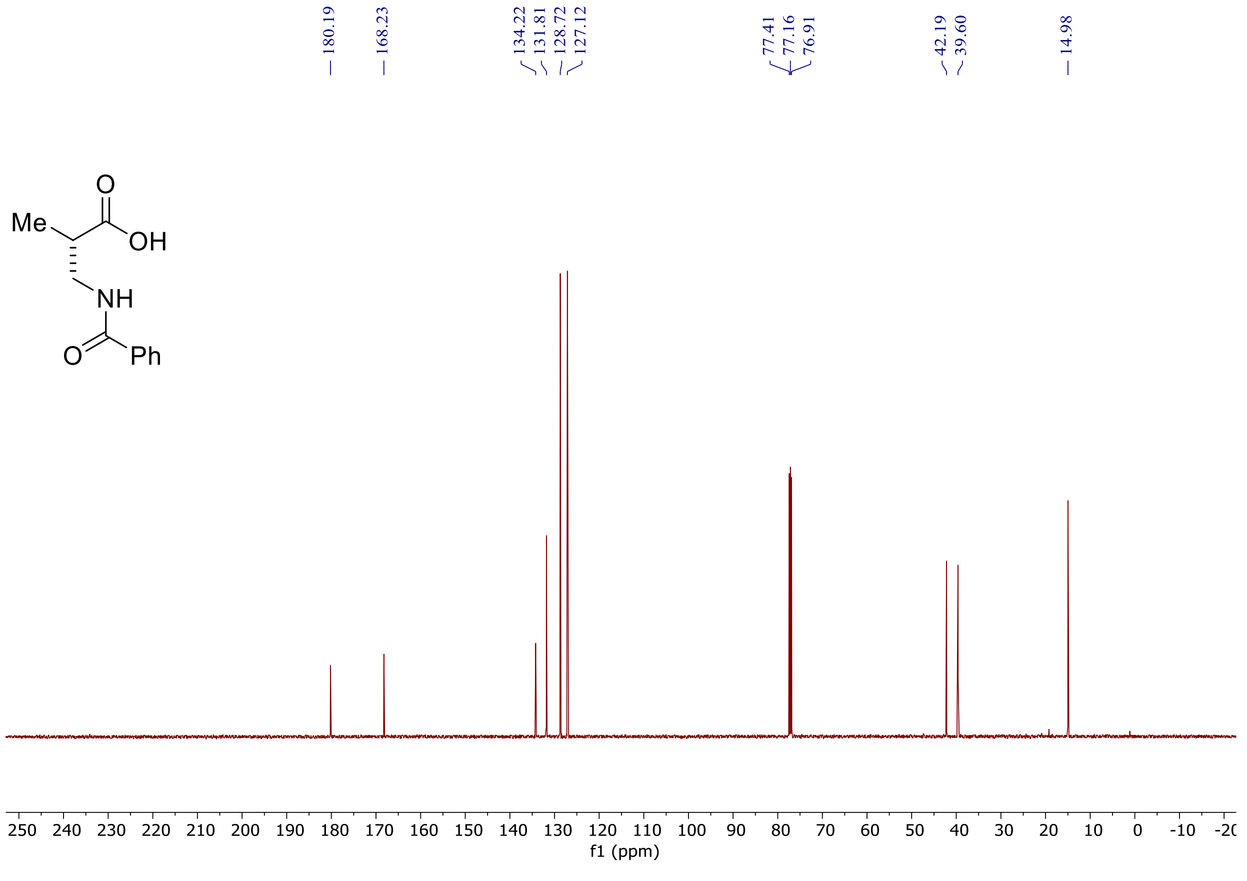

180.19
168.23
134.22
131.81
128.72
127.12
77.41
77.16
76.91
42.19
39.60
14.98
Me
O
OH
NH
O
Ph
f1 (ppm)

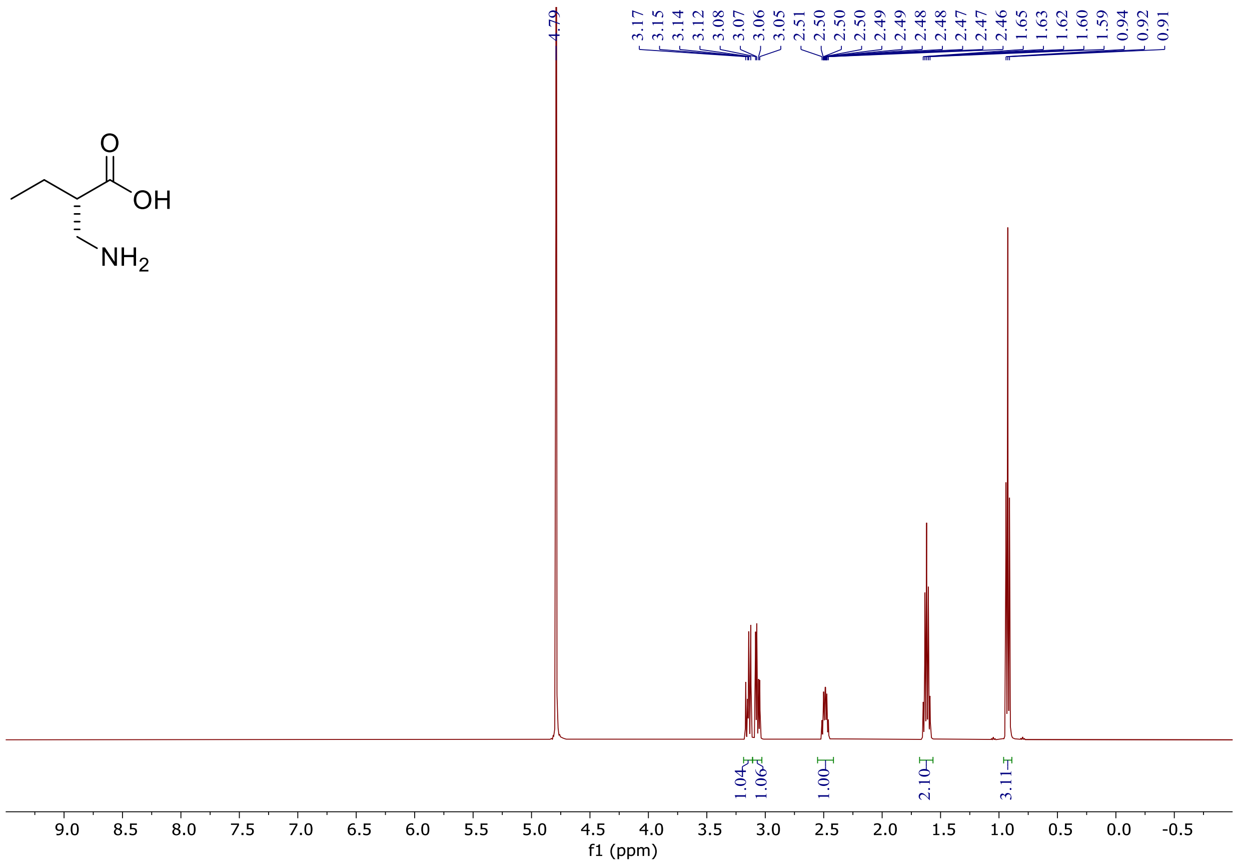
4.79
3.17 3.15 3.14 3.12 3.08 3.07 3.06 3.05 2.51 2.50 2.50 2.49 2.49 2.48 2.48 2.47 2.47 2.46 1.65 1.63 1.62 1.60 1.59 0.94 0.92 0.91
O
OH
NH2
1.04 1.06 1.00 2.10 3.11
9.0 8.5 8.0 7.5 7.0 6.5 6.0 5.5 5.0 4.5 4.0 3.5 3.0 2.5 2.0 1.5 1.0 0.5 0.0 -0.5
f1 (ppm)

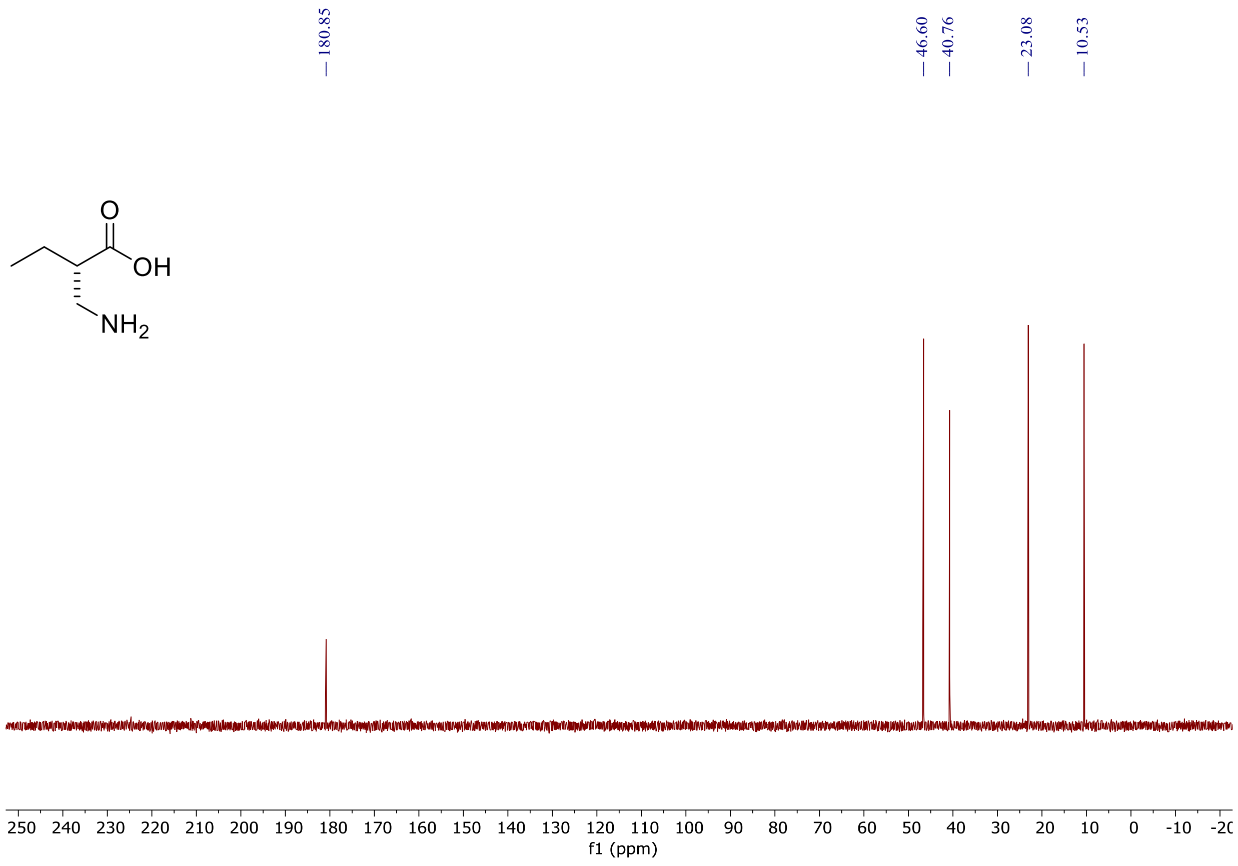
180.85
46.60 40.76 23.08 10.53
O
OH
NH2
250 240 230 220 210 200 190 180 170 160 150 140 130 120 110 100 90 80 70 60 50 40 30 20 10 0 -10 -20
f1 (ppm)

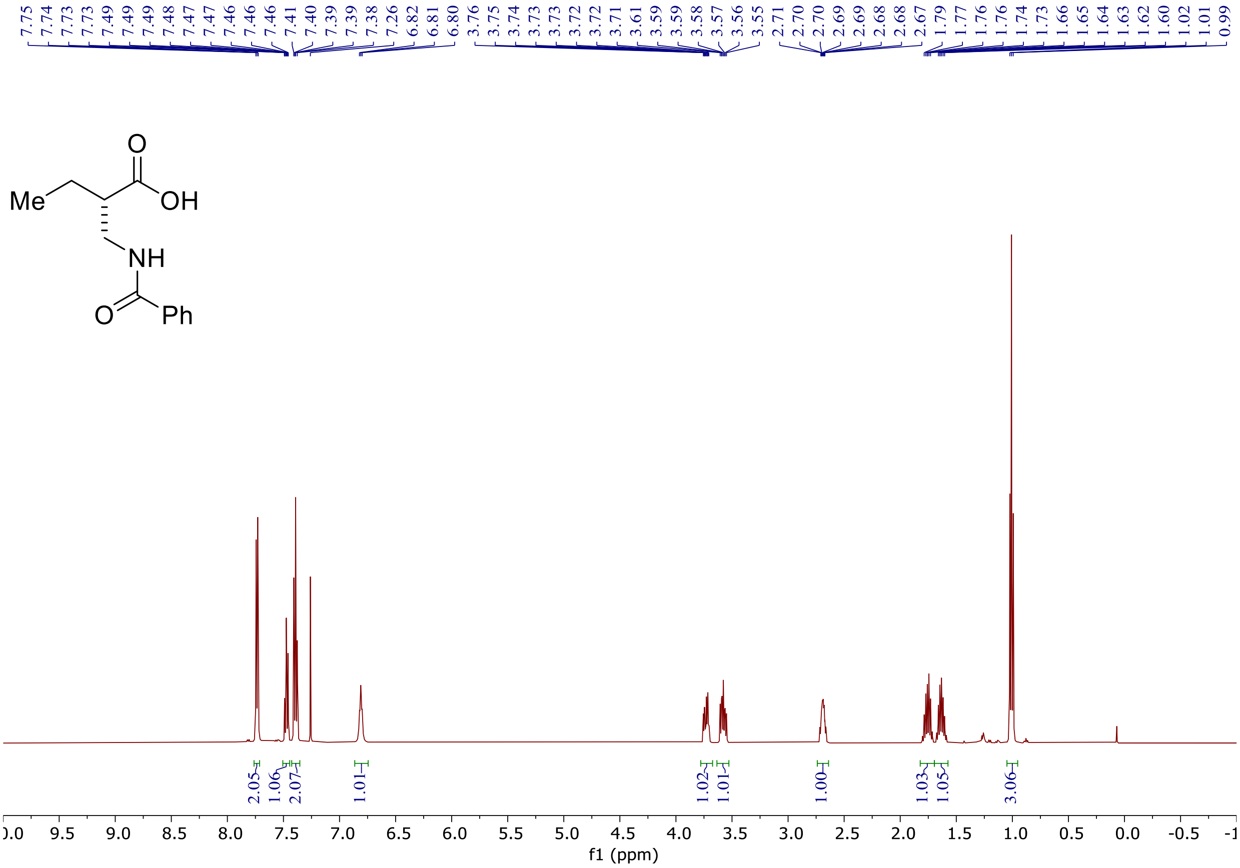

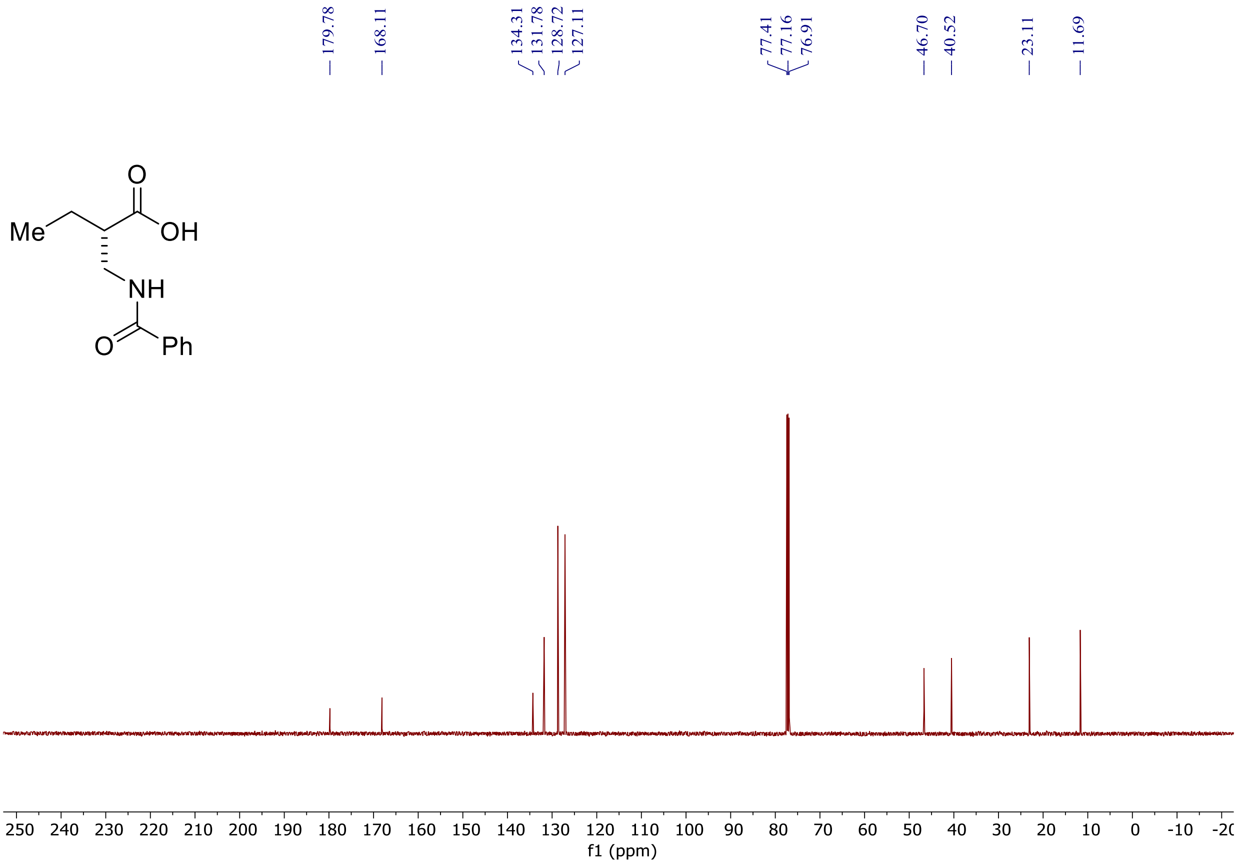

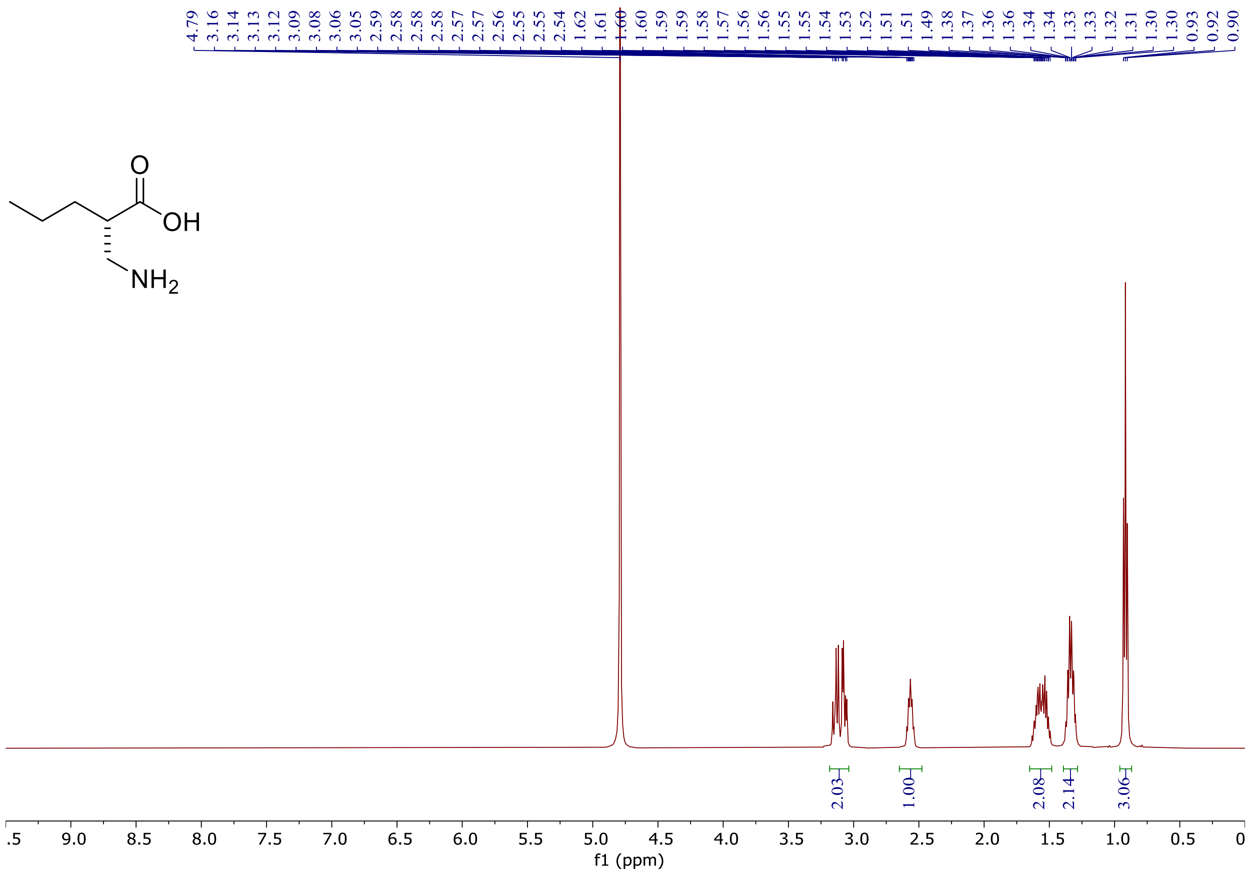

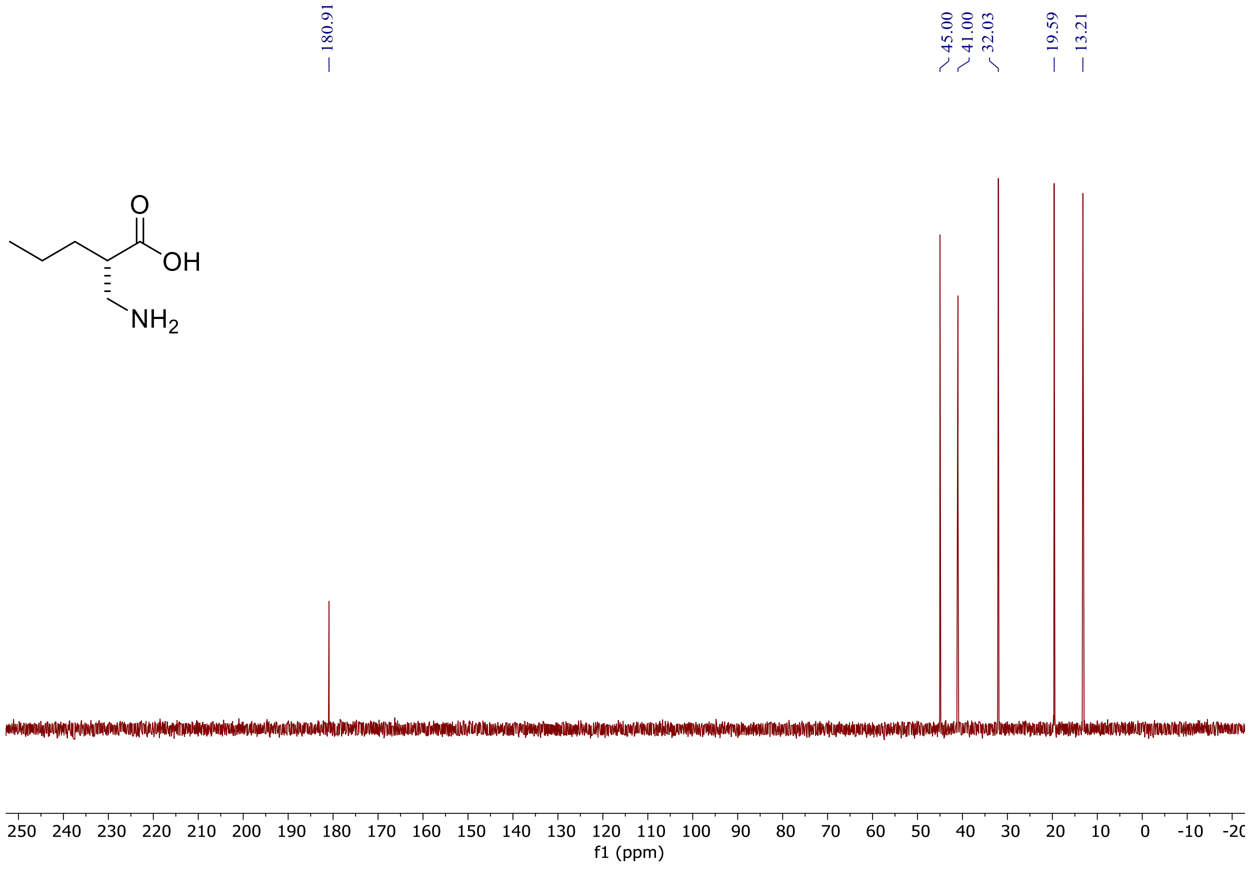

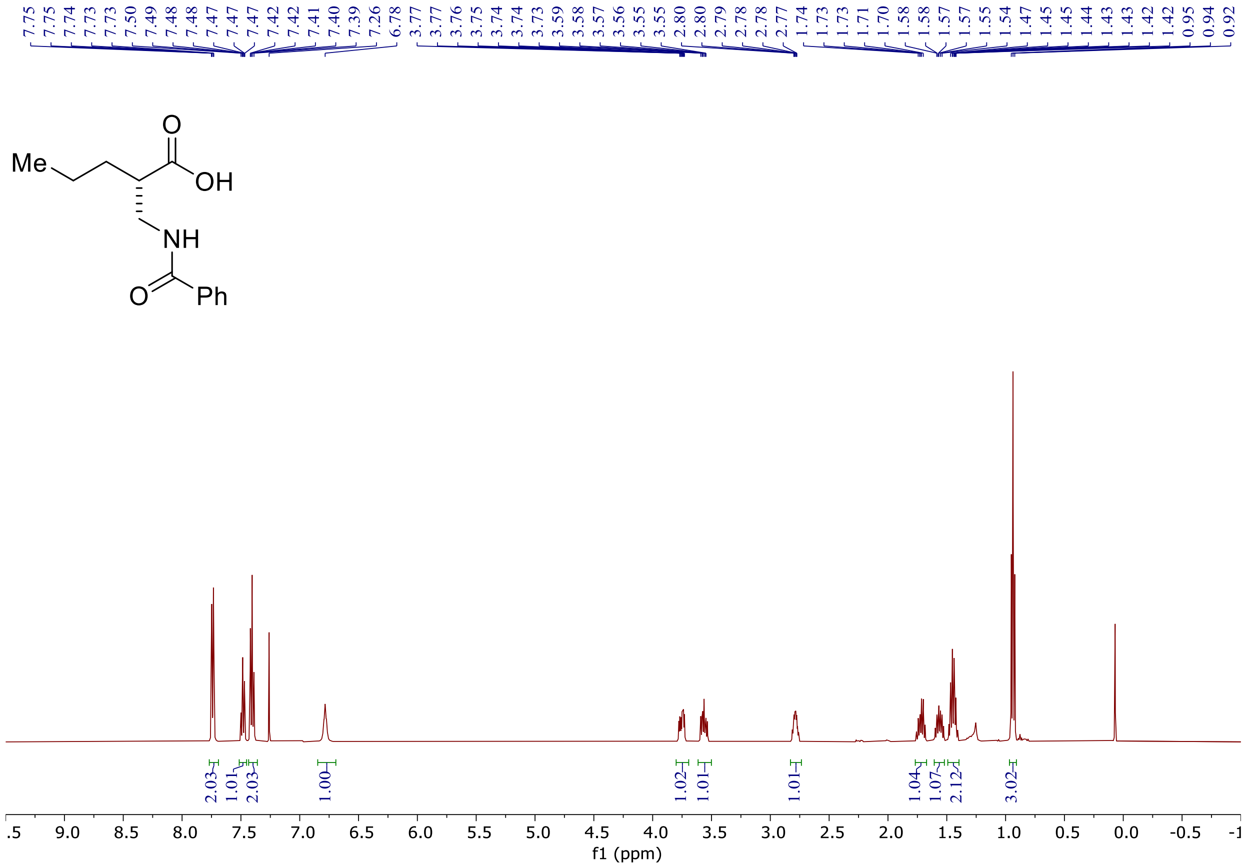
7.75
7.75
7.74
7.73
7.73
7.50
7.49
7.48
7.48
7.47
7.47
7.47
7.42
7.42
7.41
7.40
7.39
7.26
6.78
3.77
3.77
3.76
3.75
3.74
3.74
3.73
3.59
3.58
3.57
3.56
3.55
3.55
2.80
2.80
2.79
2.78
2.78
2.77
1.74
1.73
1.73
1.71
1.70
1.58
1.58
1.57
1.57
1.55
1.54
1.47
1.45
1.45
1.44
1.43
1.43
1.42
1.42
0.95
0.94
0.92
Me
O
OH
NH
O
Ph
2.03
1.01
2.03
1.00
1.02
1.01
1.01
1.04
1.07
2.12
3.02
f1 (ppm)

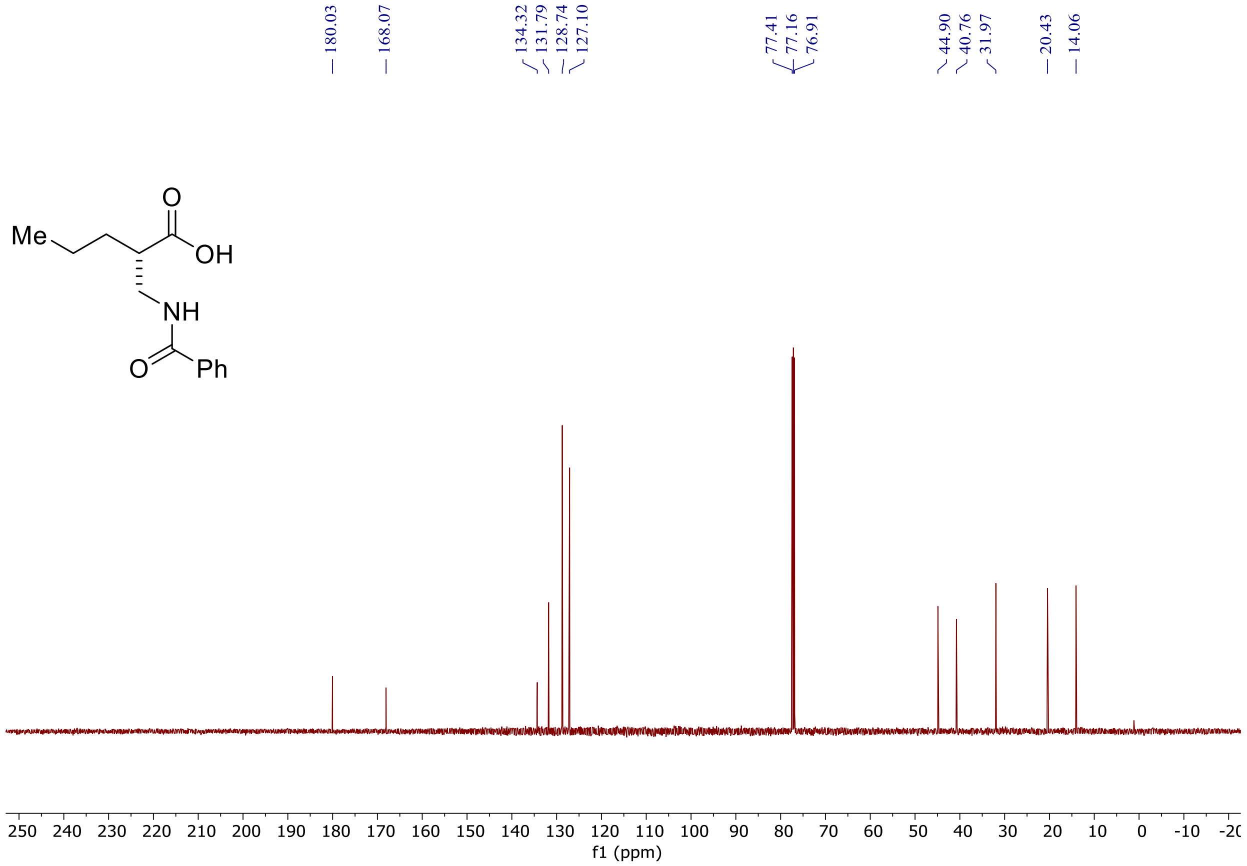
180.03
168.07
134.32
131.79
128.74
127.10
77.41
77.16
76.91
44.90
40.76
31.97
20.43
14.06
Me
O
OH
NH
O
Ph
f1 (ppm)

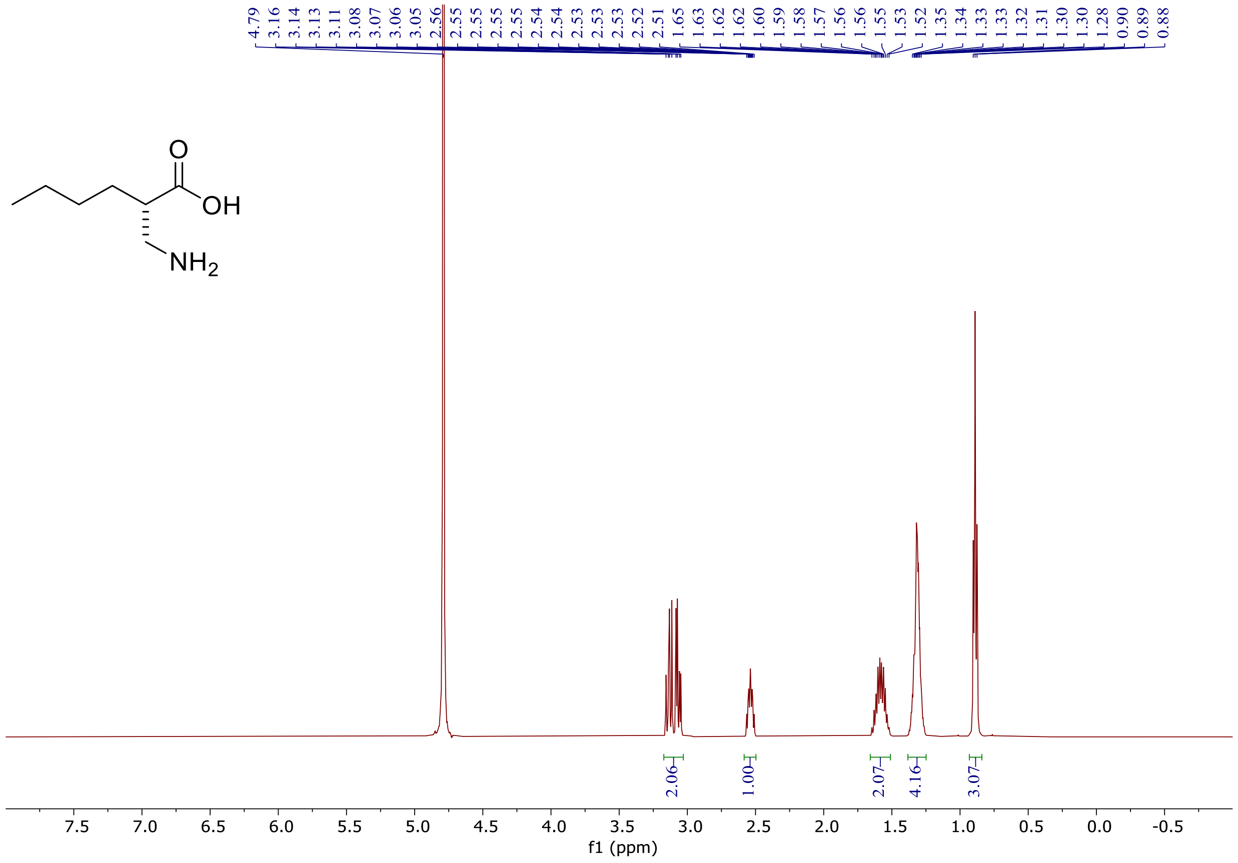
4.79
3.16
3.14
3.13
3.11
3.08
3.07
3.06
3.05
2.56
2.55
2.55
2.55
2.55
2.54
2.54
2.53
2.53
2.53
2.52
2.51
1.65
1.63
1.62
1.62
1.60
1.59
1.58
1.57
1.56
1.56
1.55
1.53
1.52
1.35
1.34
1.33
1.33
1.32
1.31
1.30
1.30
1.28
0.90
0.89
0.88
O
OH
NH2
2.06
1.00
2.07
4.16
3.07
f1 (ppm)

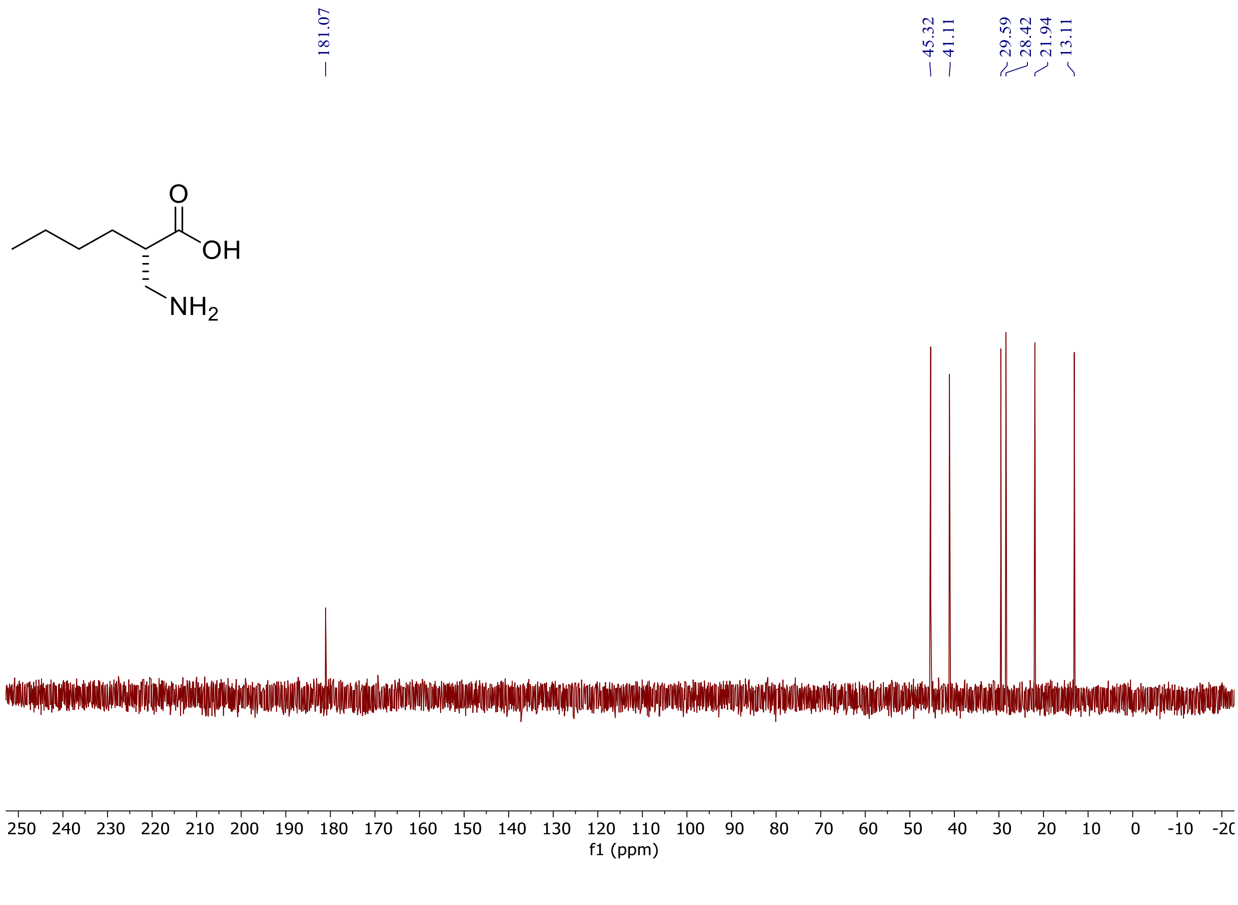
181.07
45.32
41.11
29.59
28.42
21.94
13.11
O
OH
NH2
f1 (ppm)

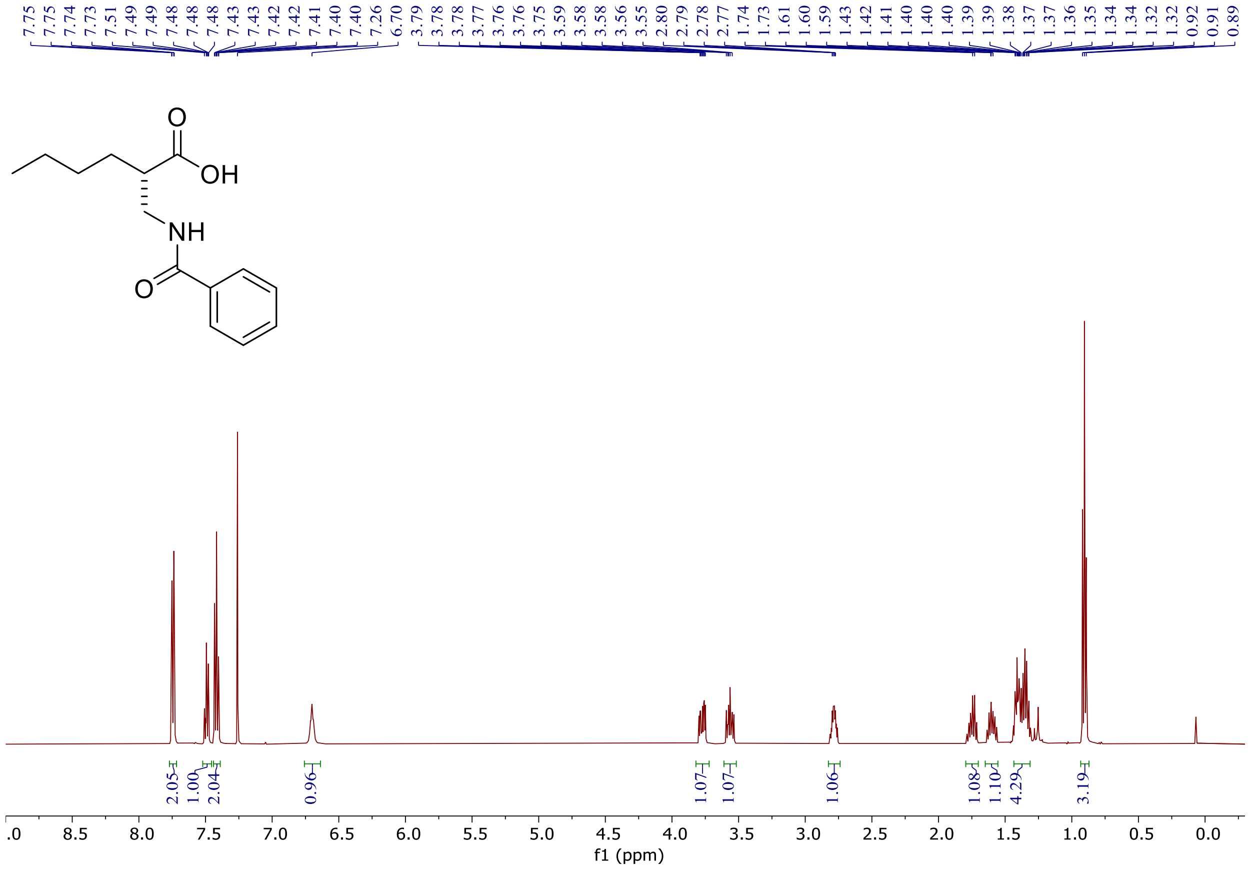
7.75 7.75 7.74 7.73 7.51 7.49 7.49 7.48 7.48 7.48 7.43 7.43 7.42 7.42 7.41 7.40 7.40 7.26 6.70 3.79 3.78 3.78 3.77 3.76 3.76 3.75 3.59 3.58 3.58 3.56 3.55 2.80 2.79 2.78 2.77 1.74 1.73 1.61 1.60 1.59 1.43 1.42 1.41 1.40 1.40 1.40 1.39 1.39 1.38 1.37 1.37 1.36 1.35 1.34 1.34 1.32 1.32 0.92 0.91 0.89
O
OH
NH
O
2.05
1.00
2.04
0.96
1.07
1.07
1.06
1.08
1.10
4.29
3.19
.0 8.5 8.0 7.5 7.0 6.5 6.0 5.5 5.0 4.5 4.0 3.5 3.0 2.5 2.0 1.5 1.0 0.5 0.0
f1 (ppm)

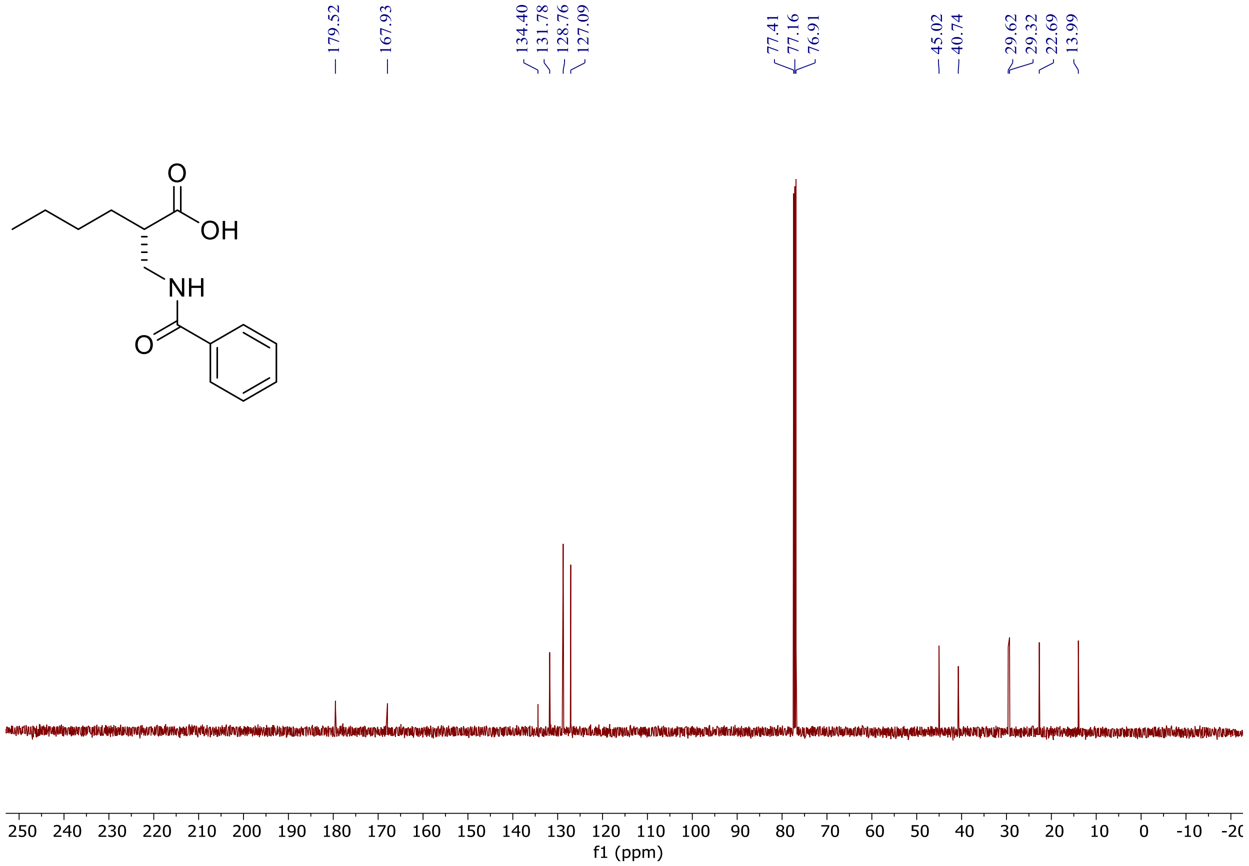
179.52
167.93
134.40 131.78 128.76 127.09
77.41 77.16 76.91
45.02
40.74
29.62 29.32 22.69 13.99
O
OH
NH
O
250 240 230 220 210 200 190 180 170 160 150 140 130 120 110 100 90 80 70 60 50 40 30 20 10 0 -10 -2(
f1 (ppm)

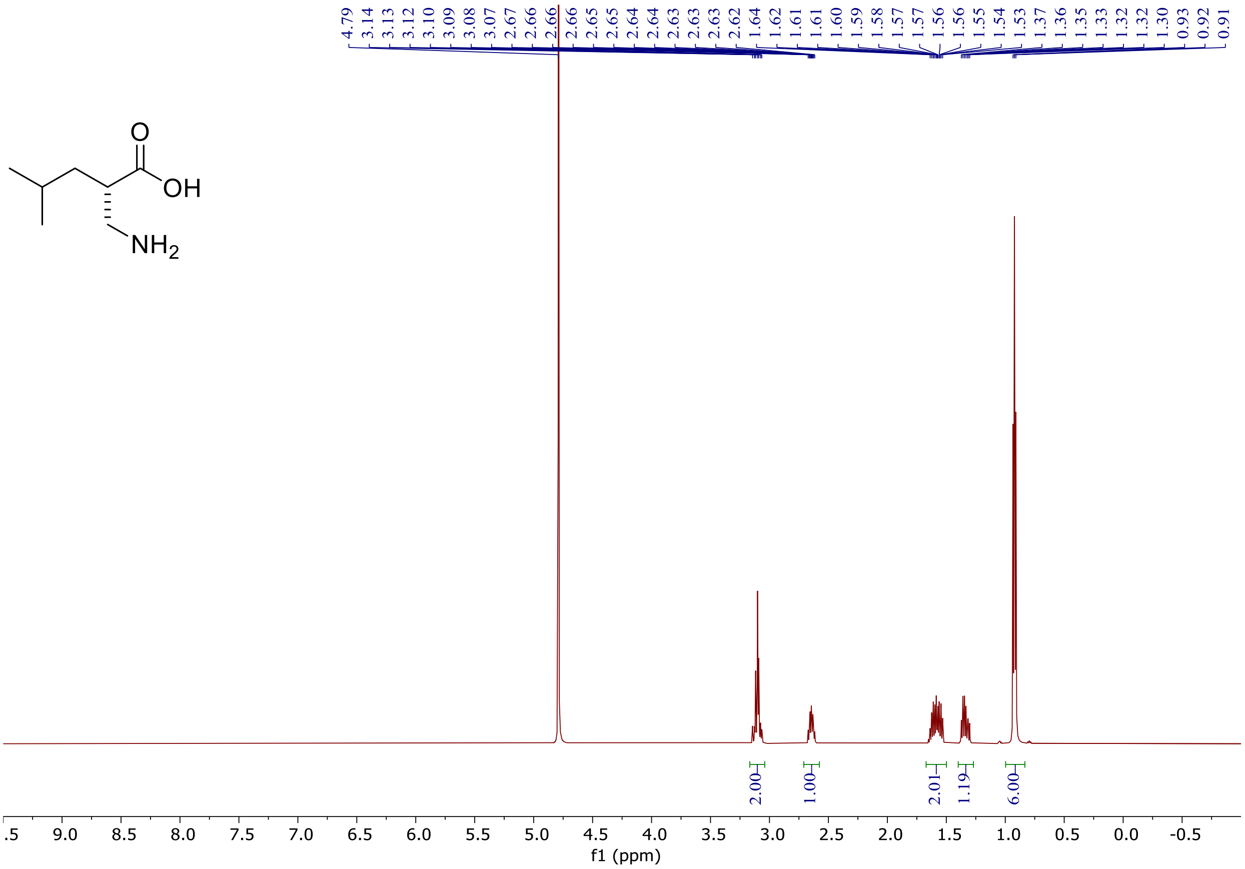
4.79 3.14 3.13 3.12 3.10 3.09 3.08 3.07 2.67 2.66 2.66 2.66 2.65 2.65 2.64 2.64 2.63 2.63 2.63 2.62 1.64 1.62 1.61 1.61 1.60 1.59 1.58 1.57 1.57 1.56 1.56 1.55 1.54 1.53 1.37 1.36 1.35 1.33 1.32 1.32 1.30 0.93 0.92 0.91
OH
O
NH2
2.00
1.00
2.01
1.19
6.00
f1 (ppm)

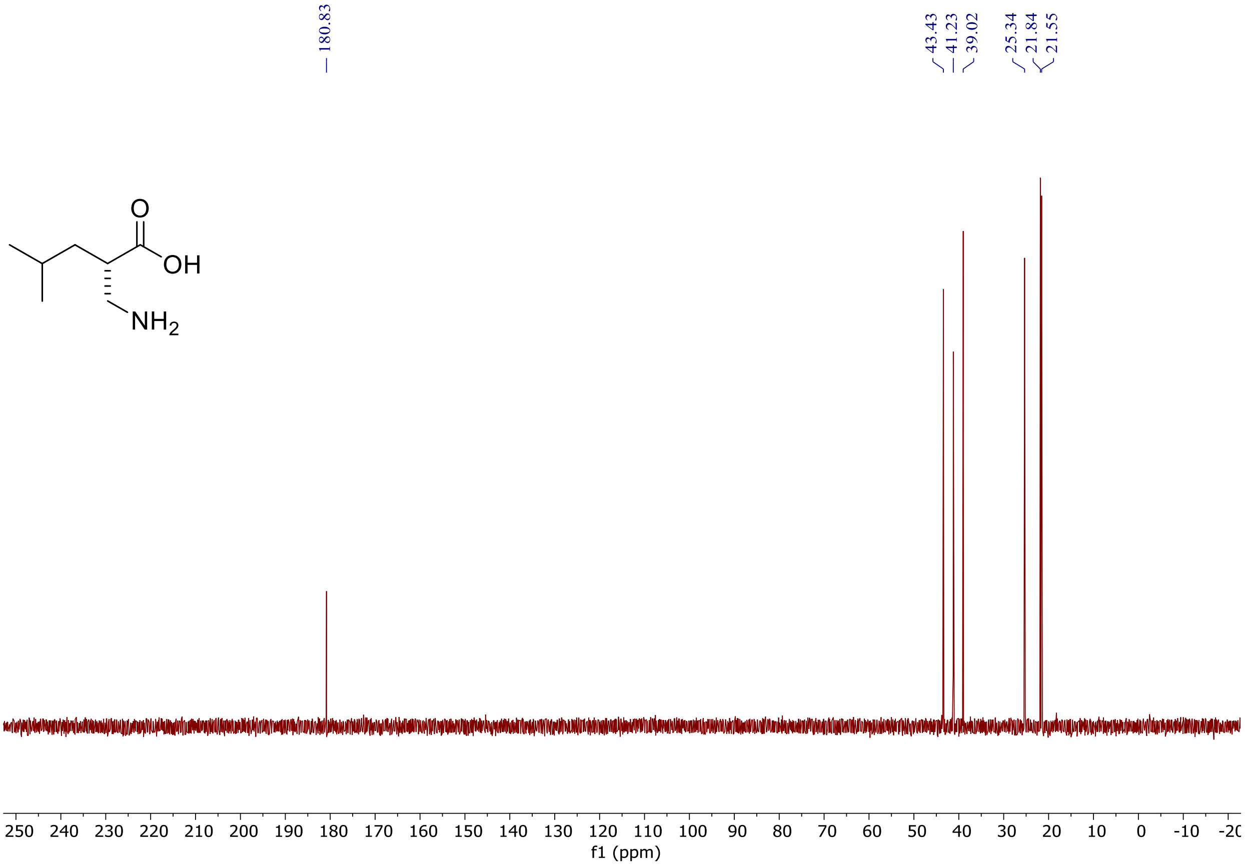
180.83
43.43 41.23 39.02
25.34 21.84 21.55
OH
O
NH2
f1 (ppm)

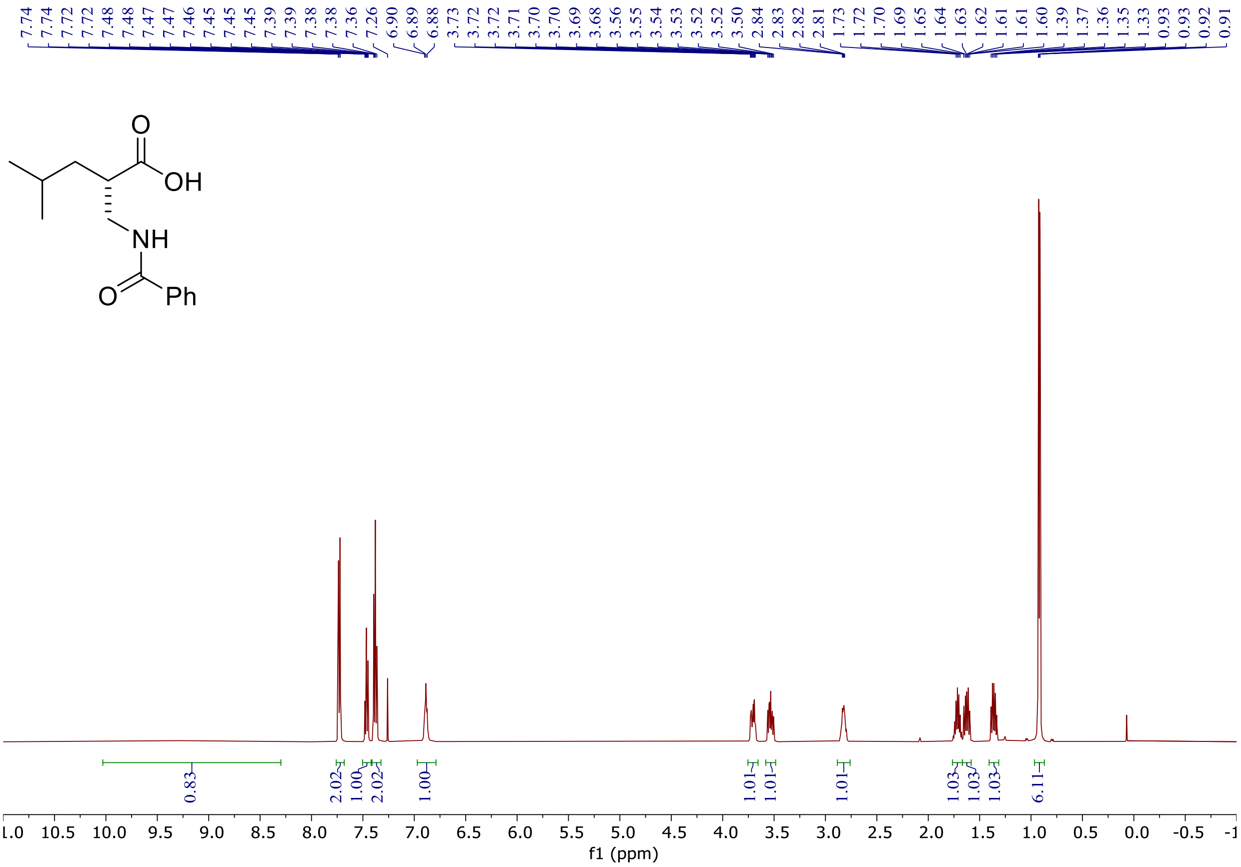

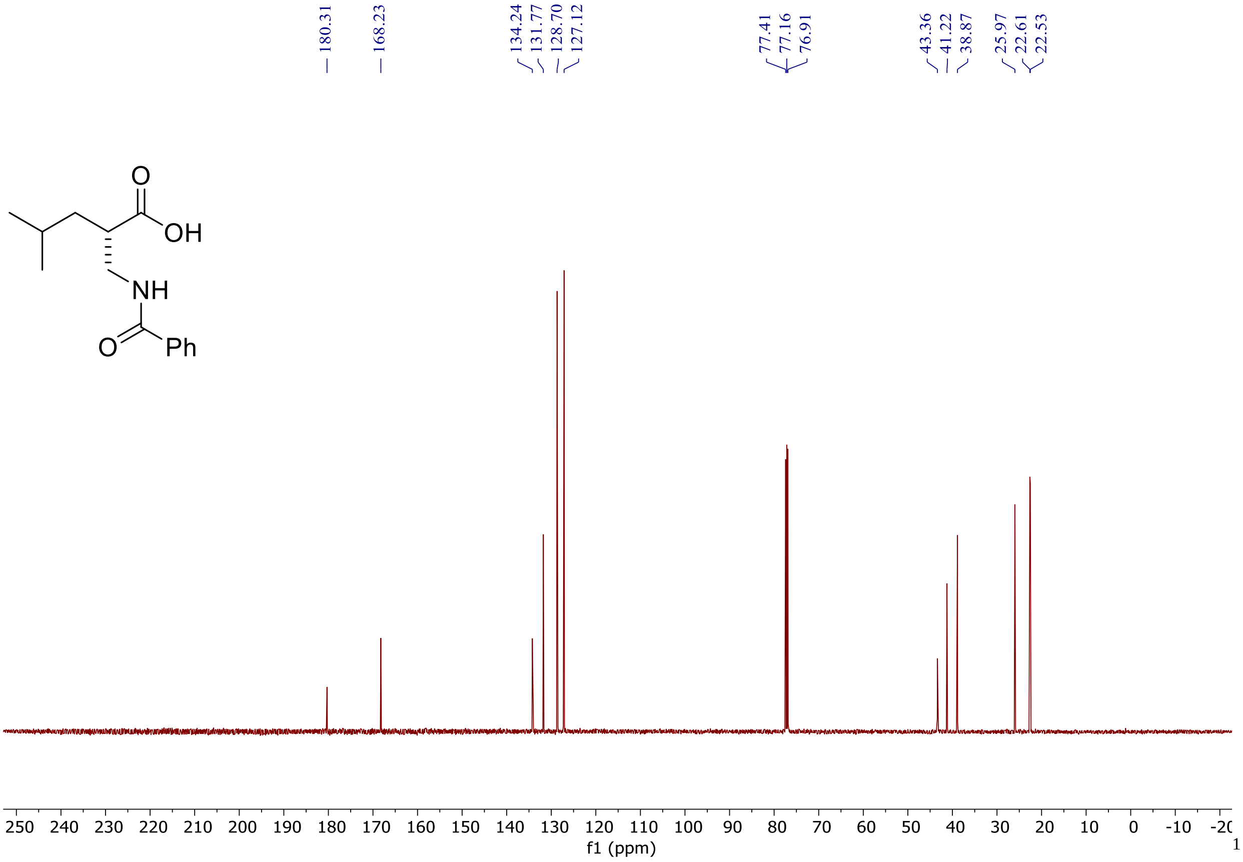

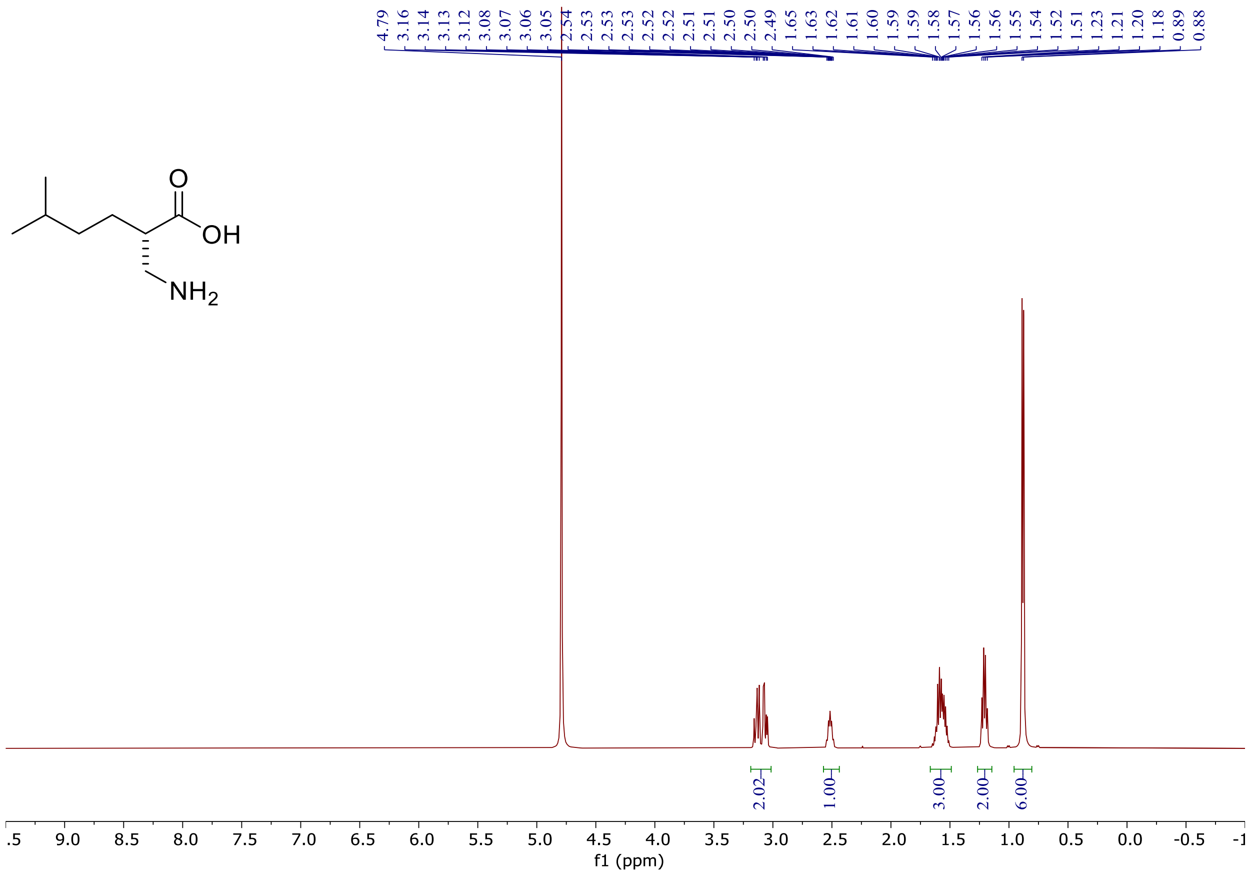
4.79
3.16
3.14
3.13
3.12
3.08
3.07
3.06
3.05
2.54
2.53
2.53
2.53
2.52
2.52
2.51
2.51
2.50
2.50
2.49
1.65
1.63
1.62
1.61
1.60
1.59
1.59
1.58
1.57
1.56
1.56
1.55
1.54
1.52
1.51
1.23
1.21
1.20
1.18
0.89
0.88
O
OH
NH2
2.02
1.00
3.00
2.00
6.00
f1 (ppm)

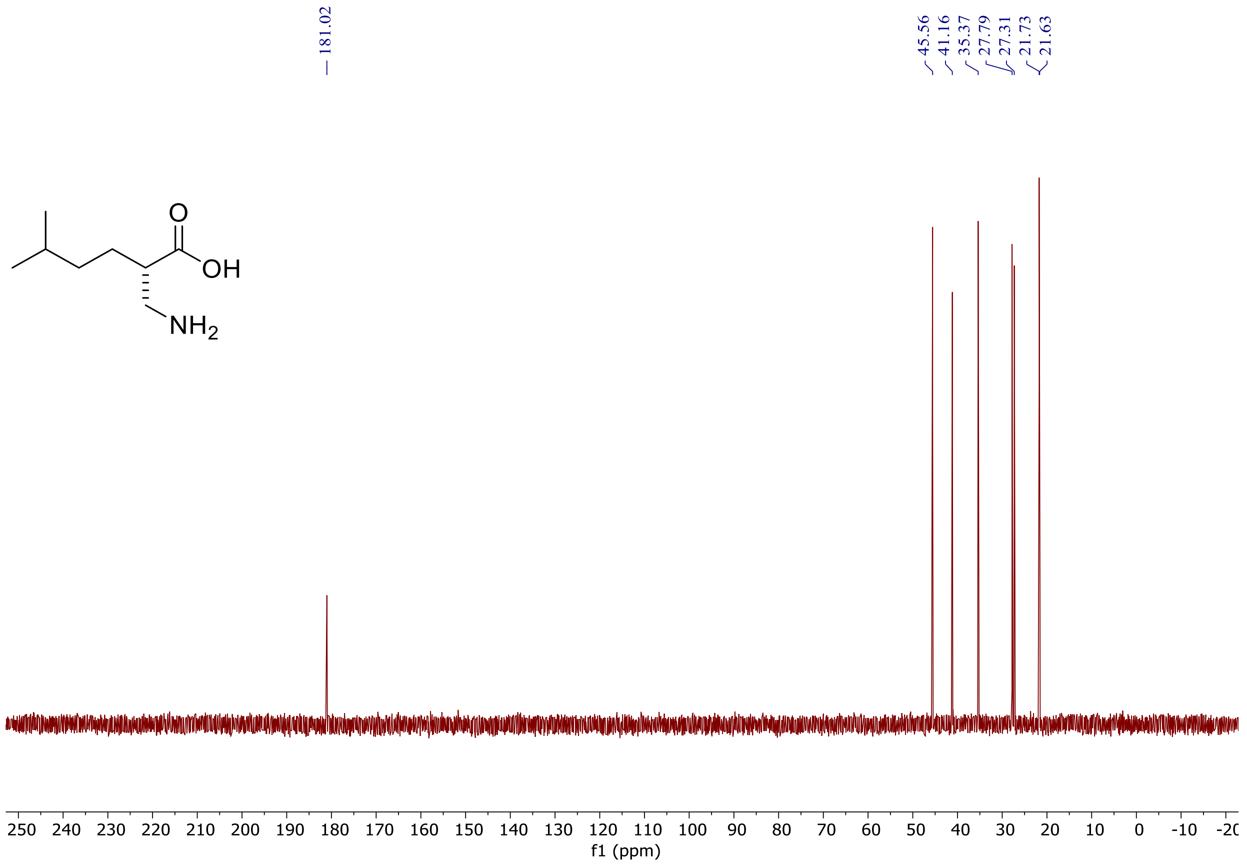
181.02
45.56
41.16
35.37
27.79
27.31
21.73
21.63
O
OH
NH2
f1 (ppm)

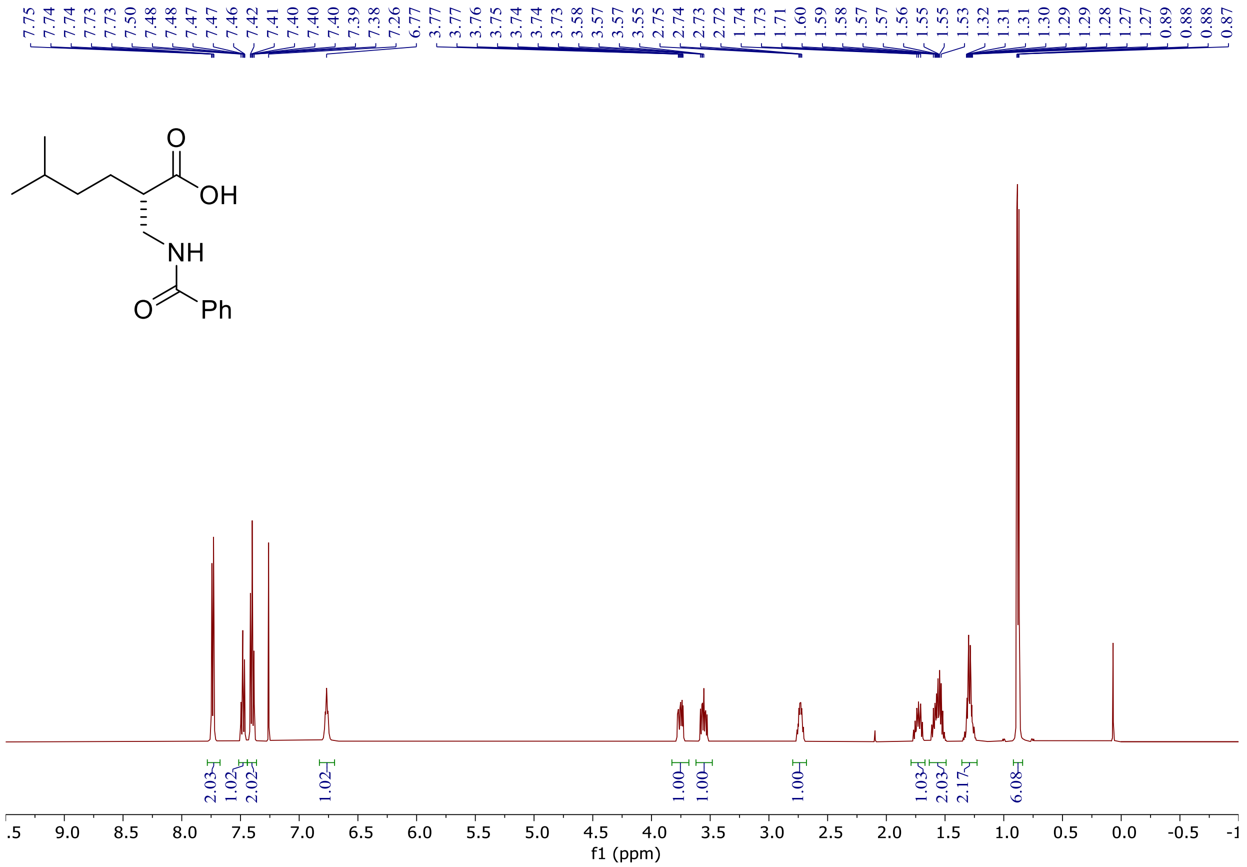

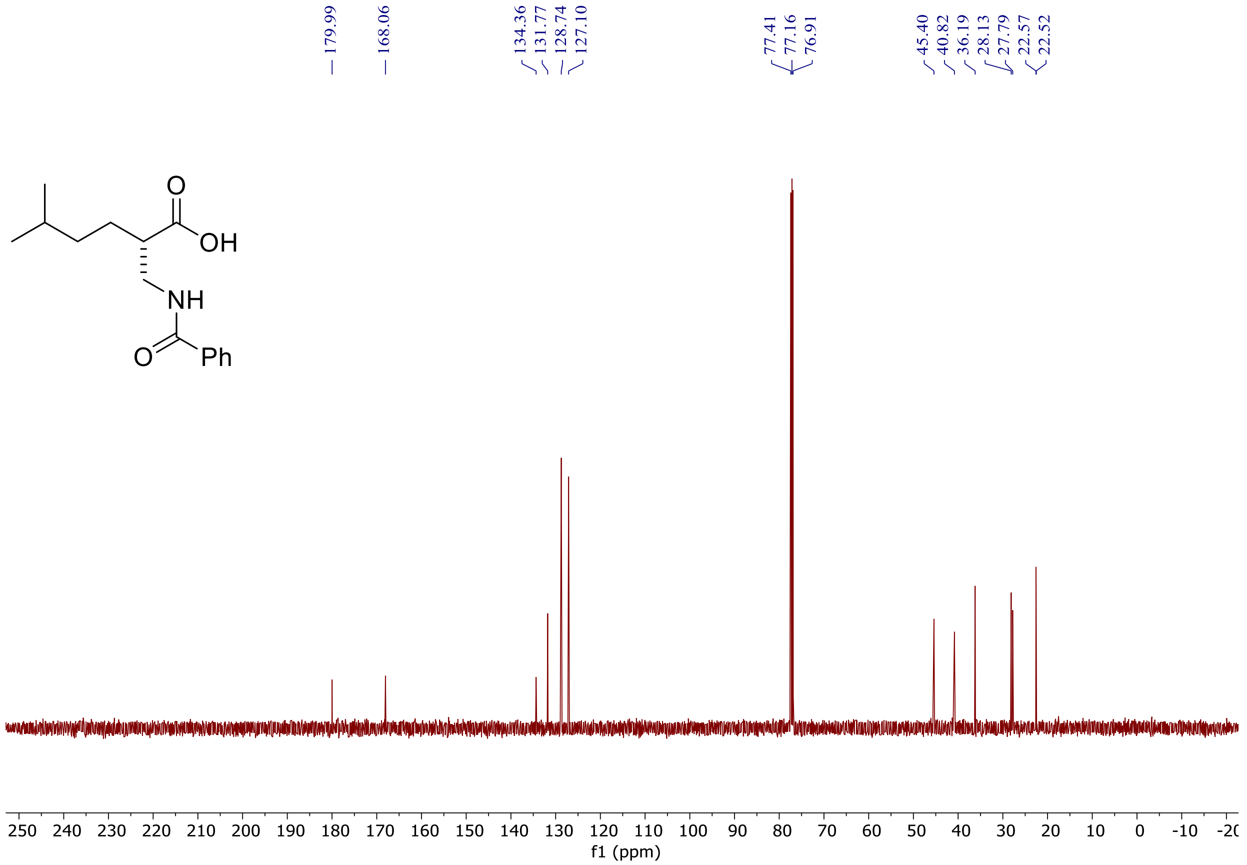

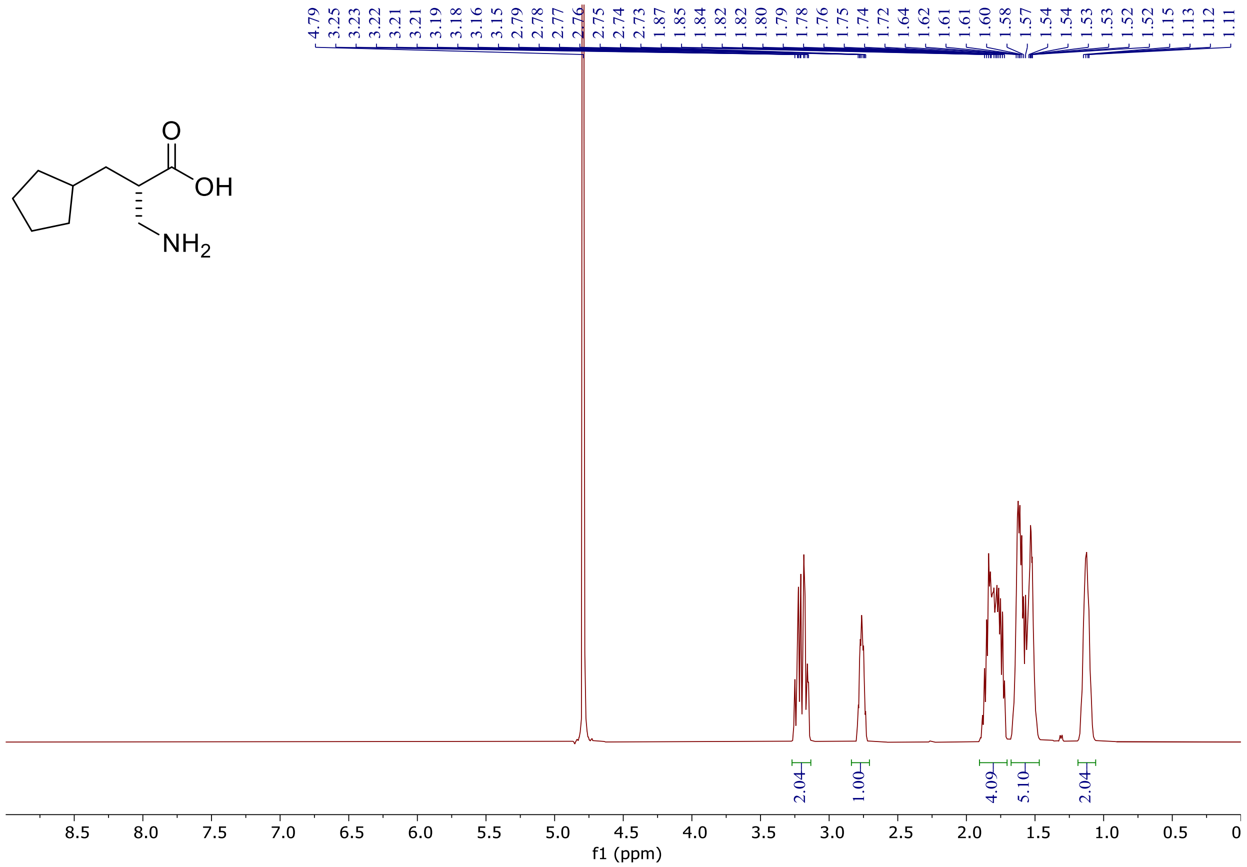
4.79 3.25 3.23 3.22 3.21 3.21 3.19 3.18 3.16 3.15 2.79 2.78 2.77 2.76 2.75 2.74 2.73 1.87 1.85 1.84 1.82 1.82 1.80 1.79 1.78 1.76 1.75 1.74 1.72 1.64 1.62 1.61 1.61 1.60 1.58 1.57 1.54 1.54 1.53 1.53 1.52 1.15 1.13 1.12 1.11
O
OH
NH2
2.04
1.00
4.09
5.10
2.04
8.5 8.0 7.5 7.0 6.5 6.0 5.5 5.0 4.5 4.0 3.5 3.0 2.5 2.0 1.5 1.0 0.5 0
f1 (ppm)

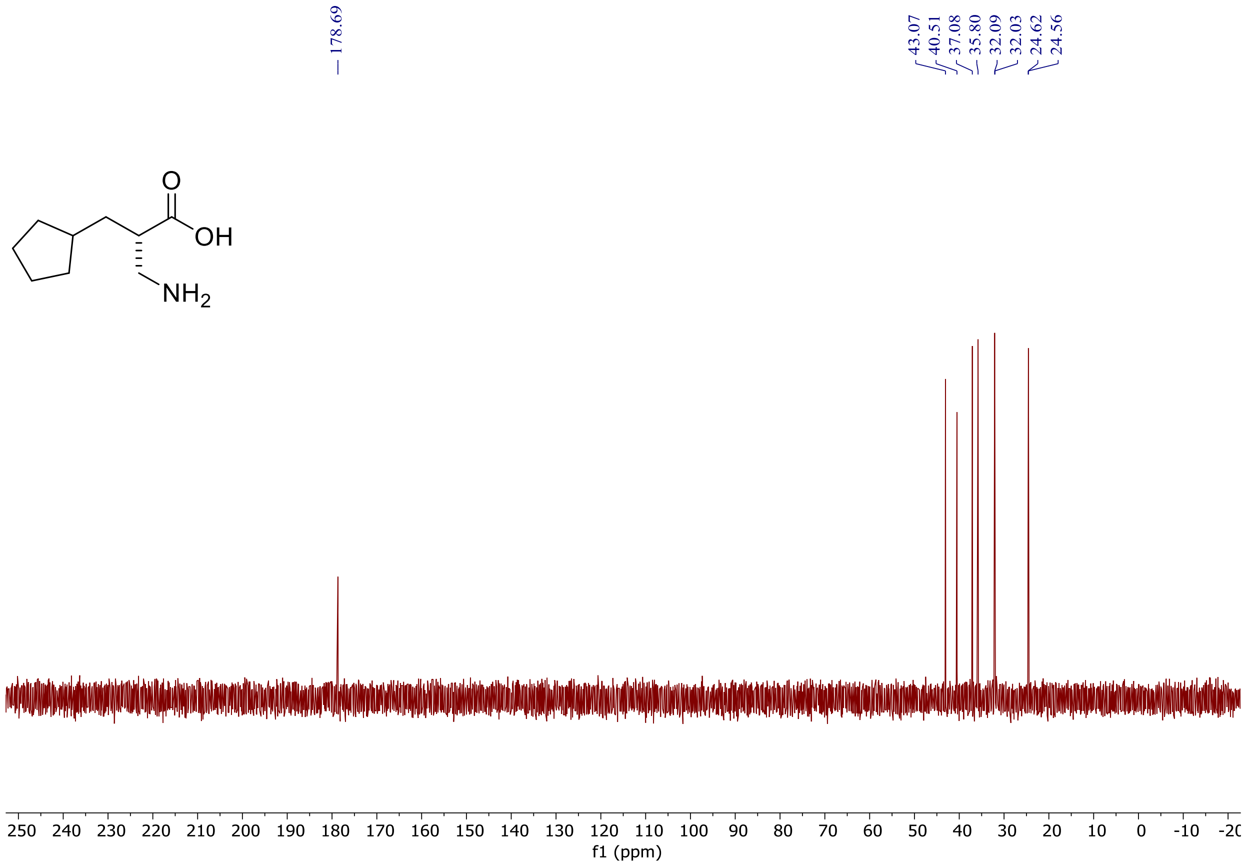
178.69
43.07 40.51 37.08 35.80 32.09 32.03 24.62 24.56
O
OH
NH2
250 240 230 220 210 200 190 180 170 160 150 140 130 120 110 100 90 80 70 60 50 40 30 20 10 0 -10 -20
f1 (ppm)

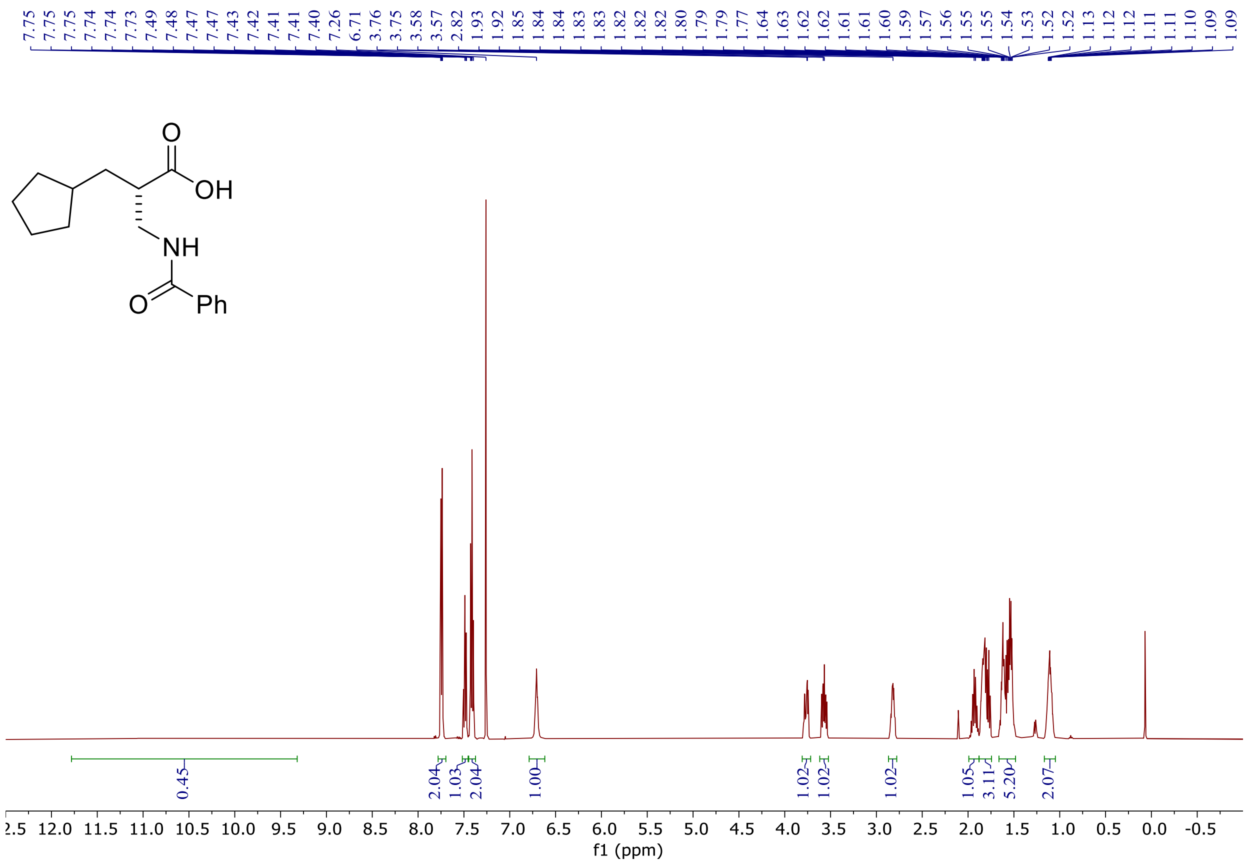
7.75 7.75 7.75 7.74 7.74 7.73 7.49 7.48 7.47 7.47 7.43 7.42 7.41 7.41 7.40 7.26 6.71 3.76 3.75 3.58 3.57 2.82 1.93 1.92 1.85 1.84 1.84 1.83 1.83 1.82 1.82 1.82 1.80 1.79 1.79 1.77 1.64 1.63 1.62 1.62 1.61 1.61 1.60 1.59 1.57 1.56 1.55 1.55 1.54 1.53 1.52 1.52 1.13 1.12 1.12 1.11 1.11 1.10 1.09 1.09
O
OH
NH
O
Ph
0.45
2.04
1.03
2.04
1.00
1.02
1.02
1.02
1.05
3.11
5.20
2.07
f1 (ppm)

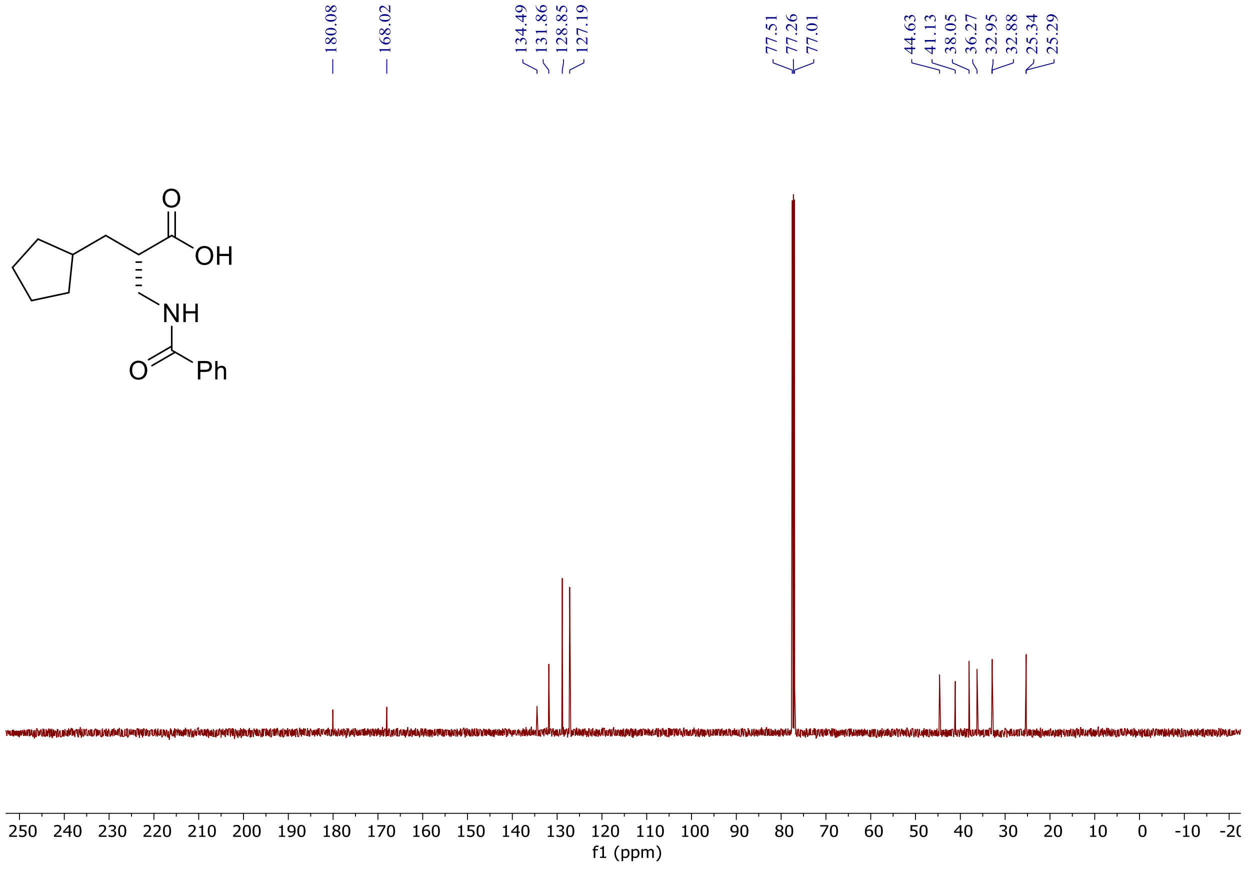
180.08
168.02
134.49 131.86 128.85 127.19
77.51 77.26 77.01
44.63 41.13 38.05 36.27 32.95 32.88 25.34 25.29
O
OH
NH
O
Ph
f1 (ppm)

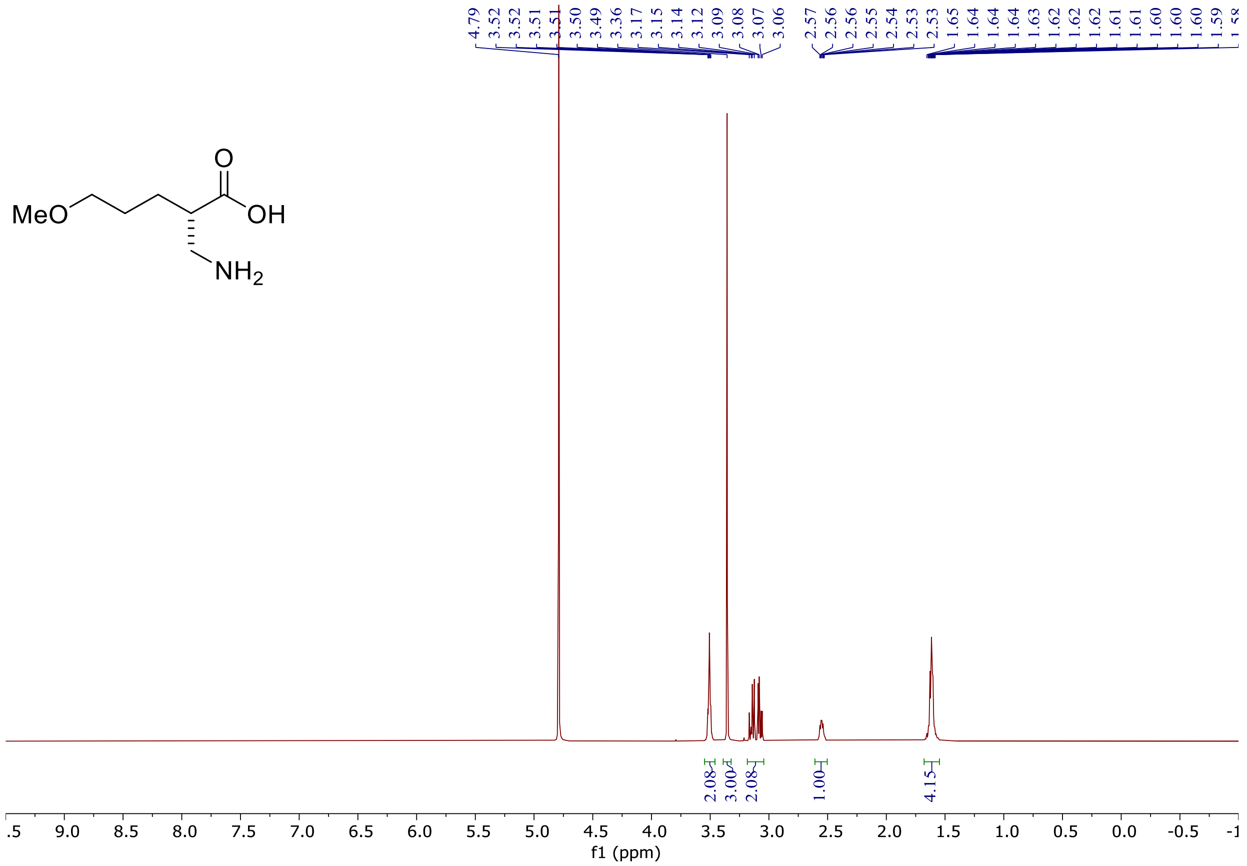

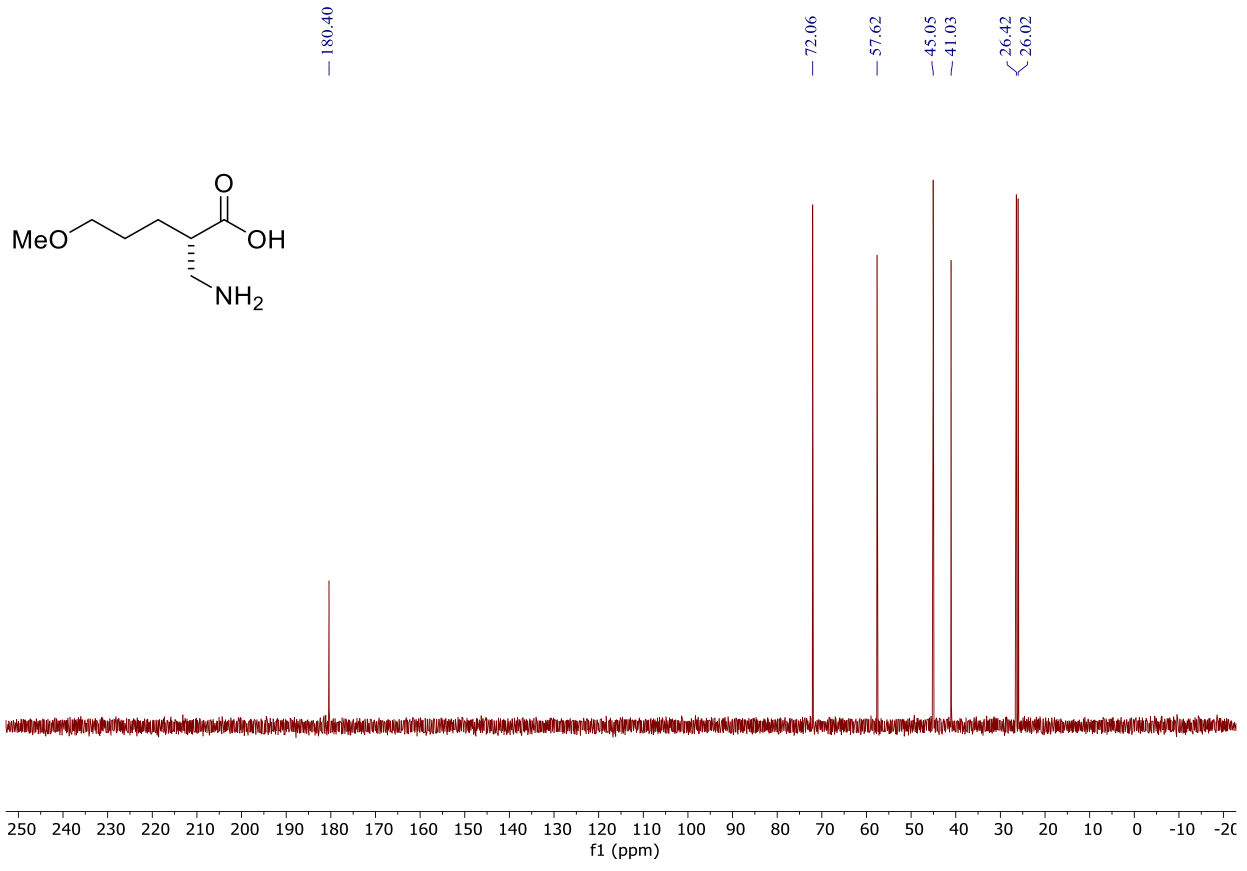

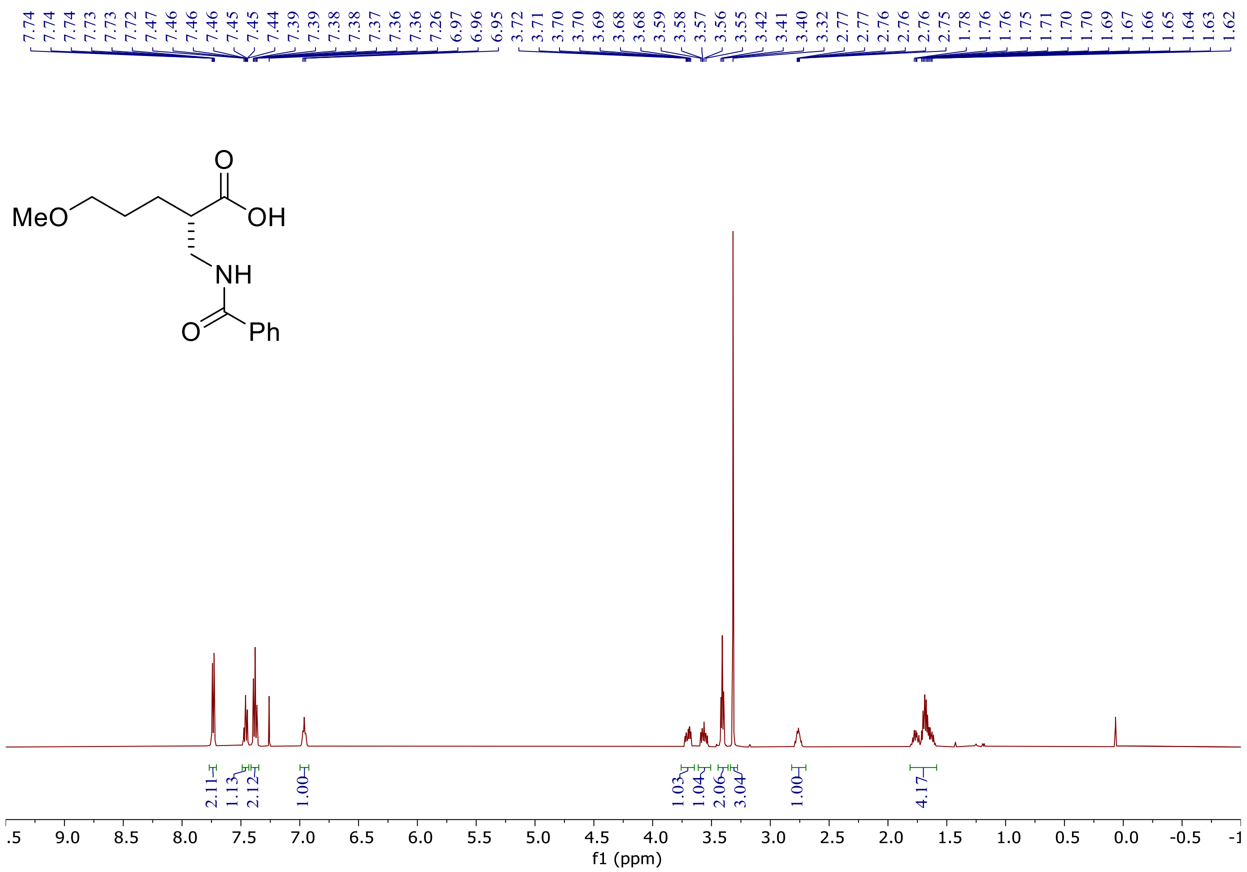
7.74 7.74 7.74 7.73 7.73 7.72 7.47 7.46 7.46 7.45 7.45 7.44 7.39 7.39 7.38 7.38 7.37 7.36 7.36 7.26 6.97 6.96 6.95 3.72 3.71 3.70 3.70 3.69 3.68 3.68 3.59 3.58 3.57 3.56 3.55 3.42 3.41 3.40 3.32 2.77 2.77 2.76 2.76 2.75 1.78 1.76 1.76 1.75 1.71 1.70 1.70 1.69 1.67 1.66 1.65 1.64 1.63 1.62
MeO
O
OH
NH
O
Ph
2.11
1.13
2.12
1.00
1.03
1.04
2.06
3.04
1.00
4.17
f1 (ppm)

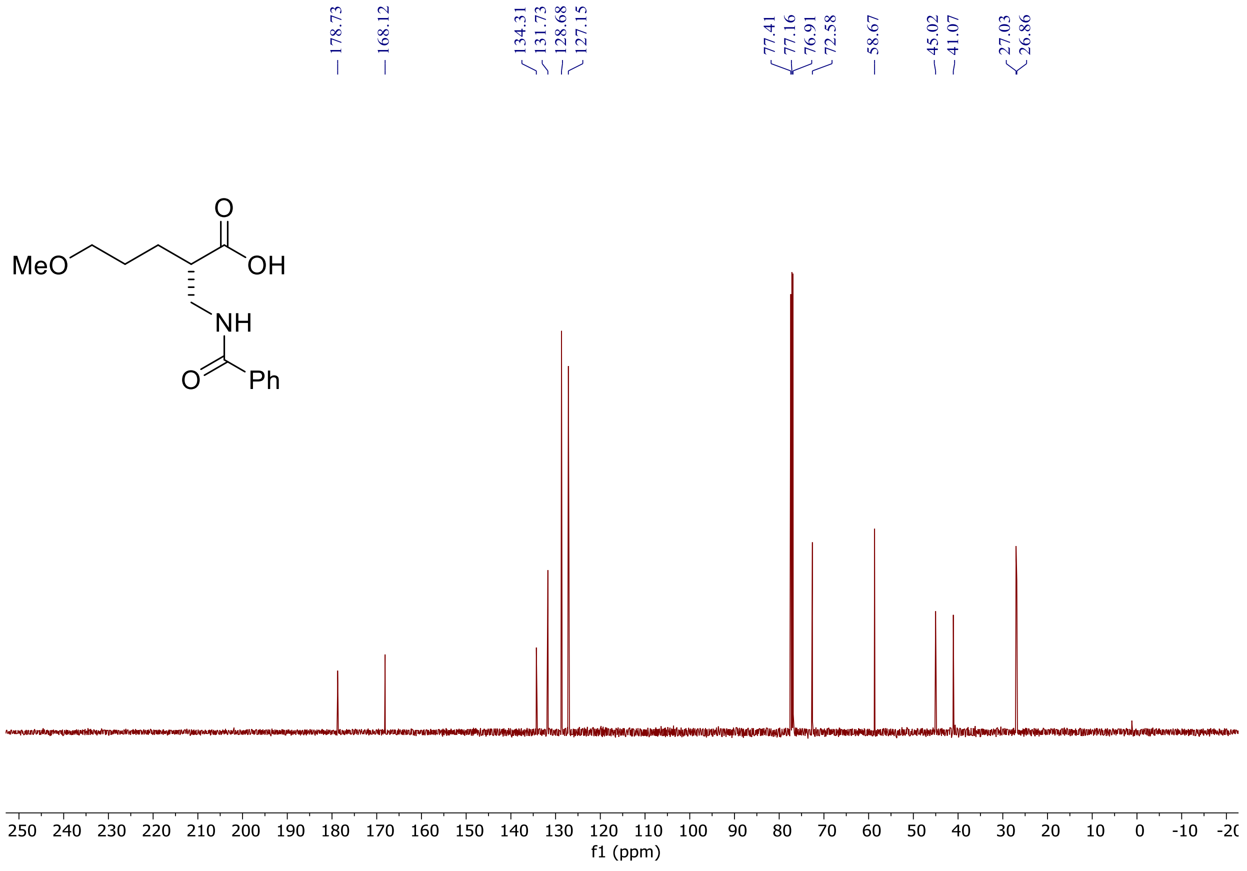
178.73
168.12
134.31 131.73 128.68 127.15
77.41 77.16 76.91 72.58
58.67
45.02 41.07
27.03 26.86
MeO
O
OH
NH
O
Ph
f1 (ppm)

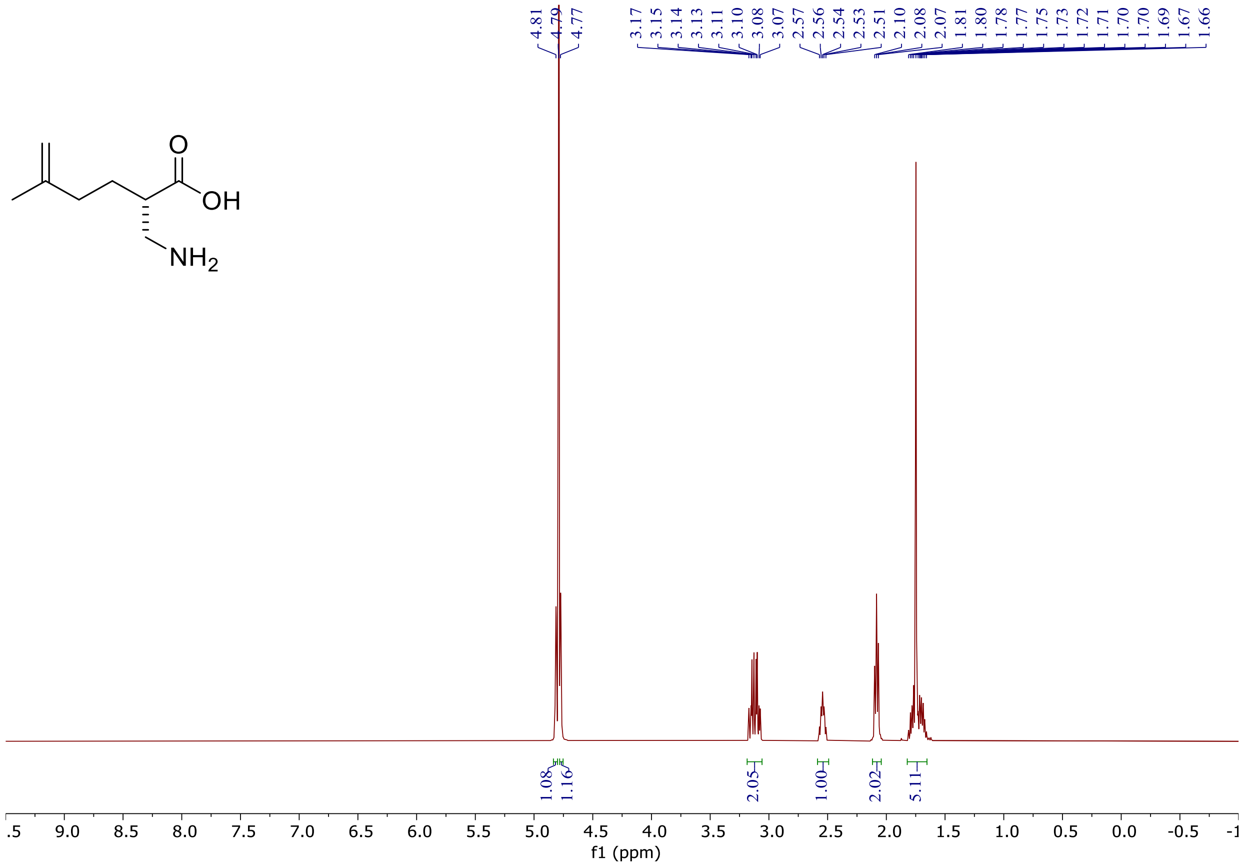
4.81 4.79 4.77
3.17 3.15 3.14 3.13 3.11 3.10 3.08 3.07 2.57 2.56 2.54 2.53 2.51 2.10 2.08 2.07 1.81 1.80 1.78 1.77 1.75 1.73 1.72 1.71 1.70 1.70 1.69 1.67 1.66
O
OH
NH_2
1.08
1.16
2.05
1.00
2.02
5.11
.5 9.0 8.5 8.0 7.5 7.0 6.5 6.0 5.5 5.0 4.5 4.0 3.5 3.0 2.5 2.0 1.5 1.0 0.5 0.0 -0.5 -1
f1 (ppm)

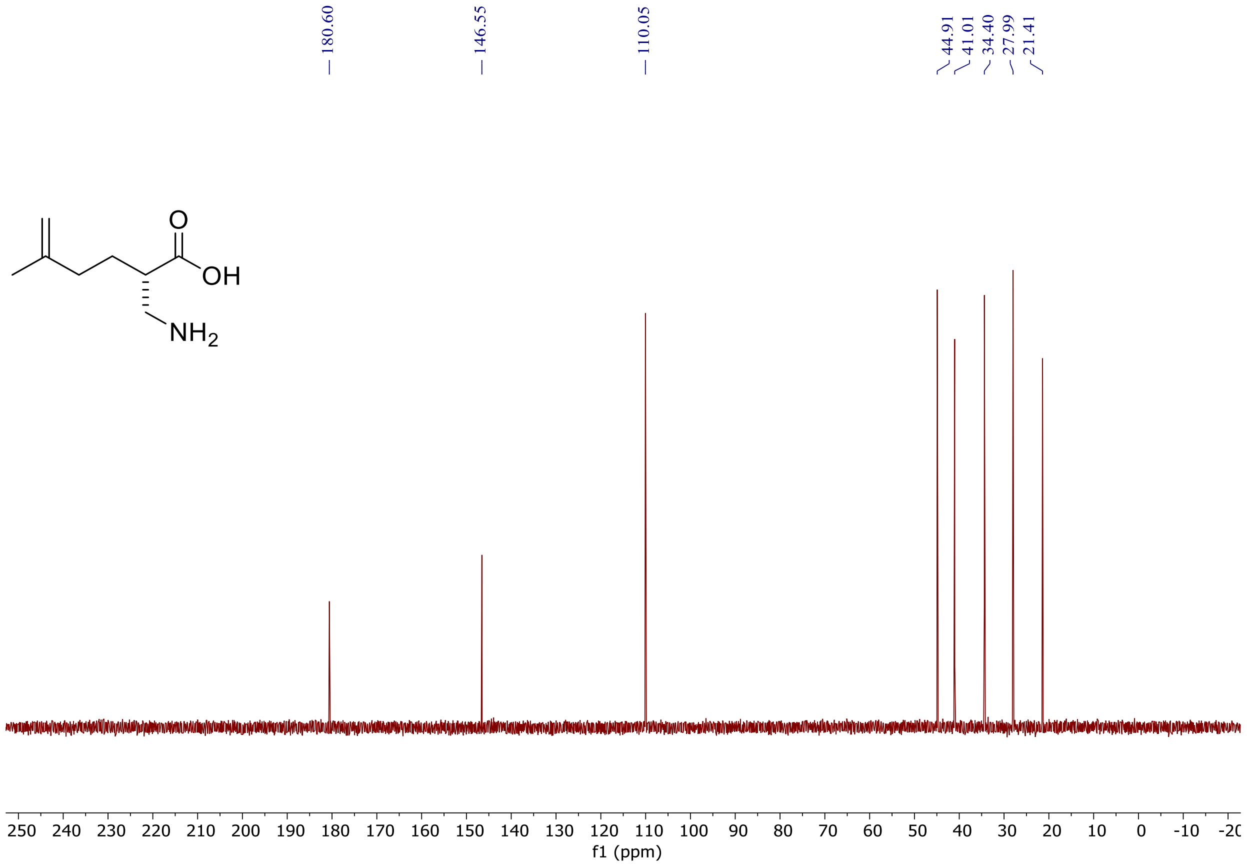
180.60
146.55
110.05
44.91 41.01 34.40 27.99 21.41
O
OH
NH_2
250 240 230 220 210 200 190 180 170 160 150 140 130 120 110 100 90 80 70 60 50 40 30 20 10 0 -10 -20
f1 (ppm)

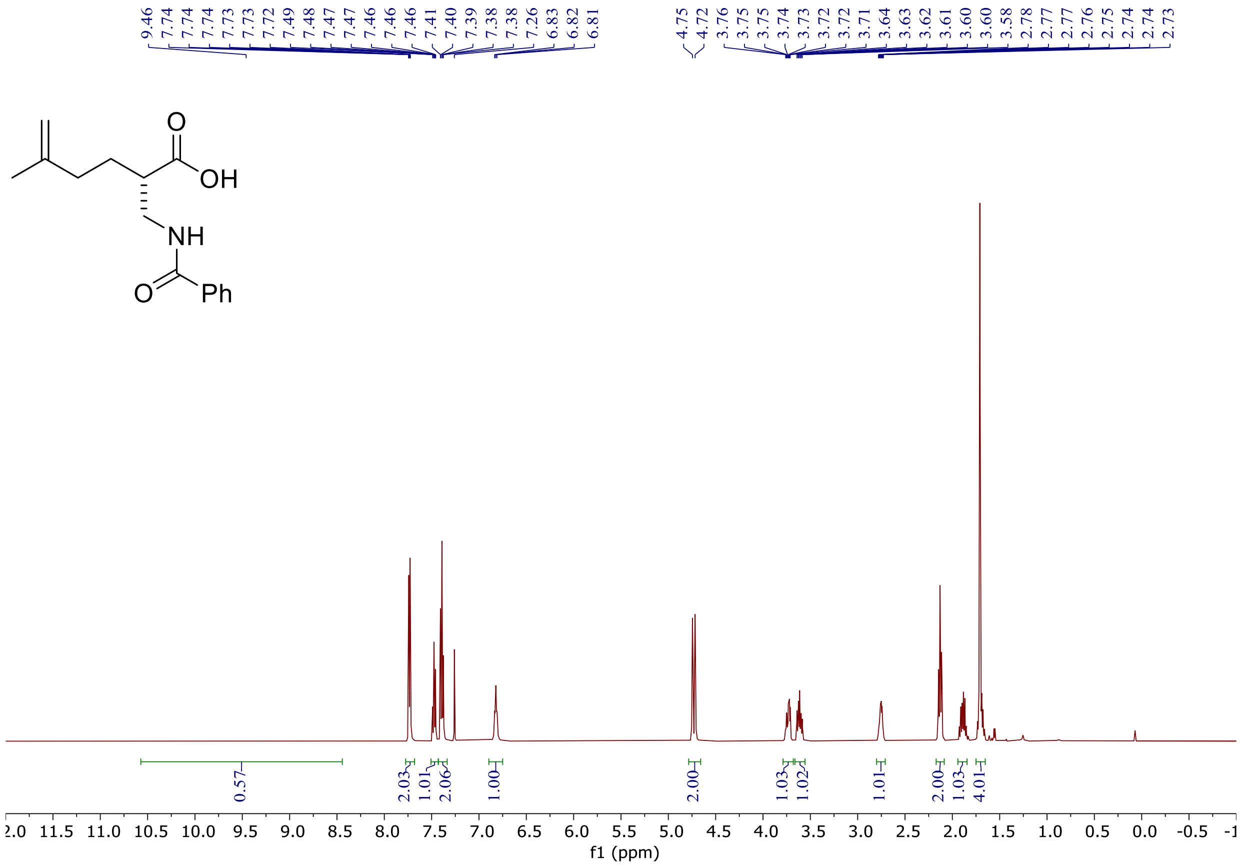
9.46
7.74
7.74
7.73
7.73
7.72
7.49
7.48
7.47
7.47
7.46
7.46
7.41
7.40
7.39
7.38
7.38
7.26
6.83
6.82
6.81
4.75
4.72
3.76
3.75
3.75
3.74
3.73
3.72
3.72
3.71
3.64
3.63
3.62
3.61
3.60
3.60
3.58
2.78
2.77
2.77
2.76
2.75
2.74
2.74
2.73
OH
NH
Ph
0.57
2.03
1.01
2.06
1.00
2.00
1.03
1.02
1.01
2.00
1.03
4.01
f1 (ppm)

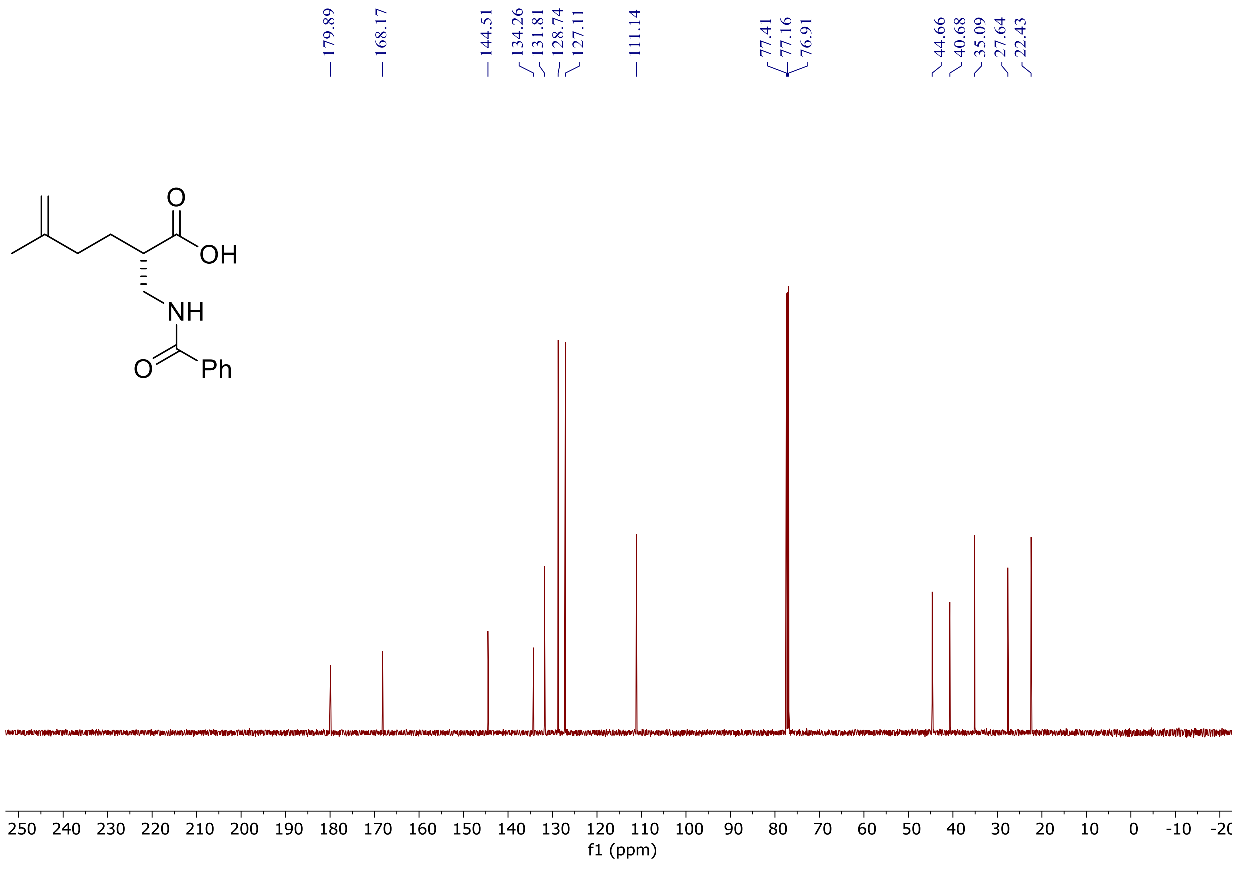
179.89
168.17
144.51
134.26
131.81
128.74
127.11
111.14
77.41
77.16
76.91
44.66
40.68
35.09
27.64
22.43
OH
NH
Ph
f1 (ppm)

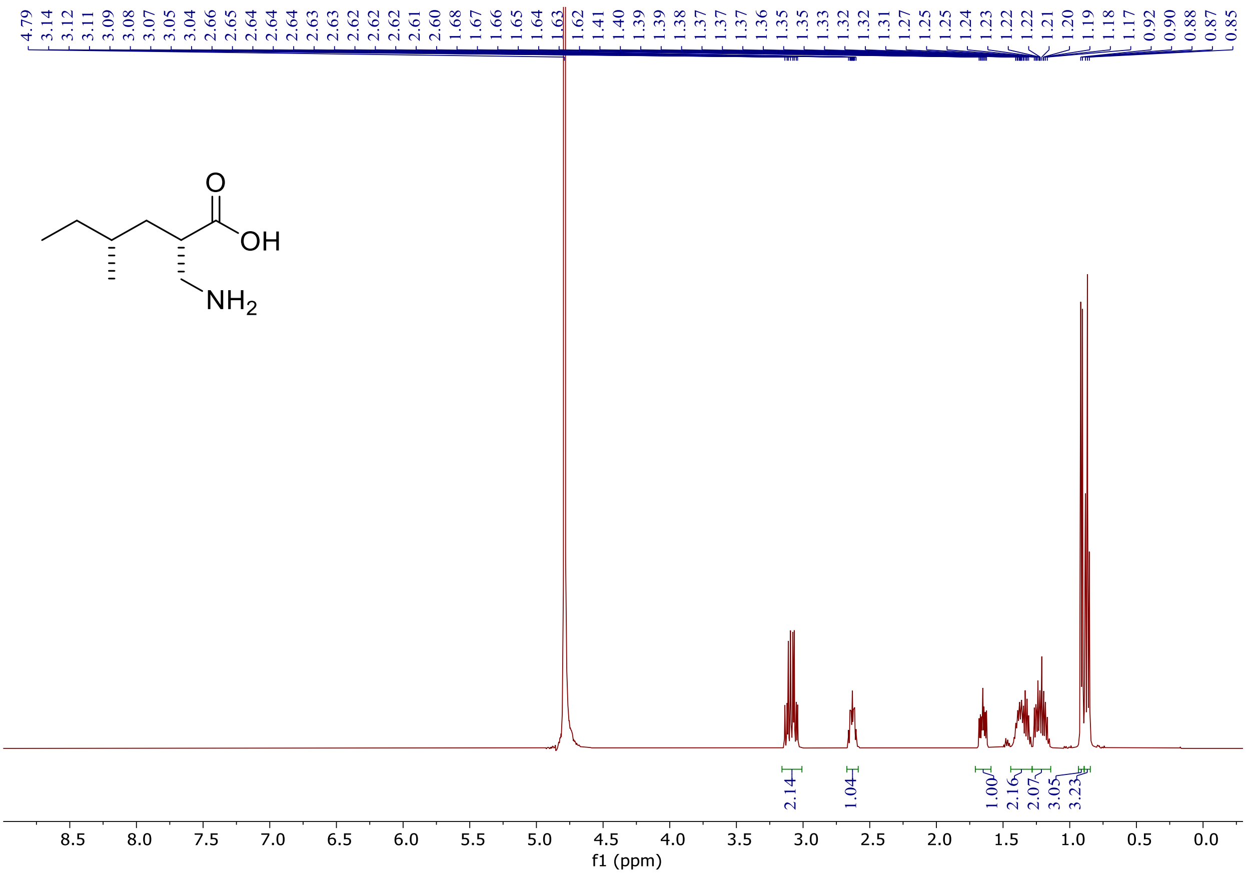
4.79 3.14 3.12 3.11 3.09 3.08 3.07 3.05 3.04 2.66 2.65 2.64 2.64 2.64 2.63 2.63 2.62 2.62 2.62 2.61 2.60 1.68 1.67 1.66 1.65 1.64 1.63 1.62 1.41 1.40 1.39 1.39 1.38 1.37 1.37 1.37 1.36 1.35 1.35 1.33 1.32 1.32 1.31 1.27 1.25 1.25 1.24 1.23 1.22 1.22 1.21 1.20 1.19 1.18 1.17 0.92 0.90 0.88 0.87 0.85
OH
NH2
2.14
1.04
1.00
2.16
2.07
3.05
3.23
f1 (ppm)

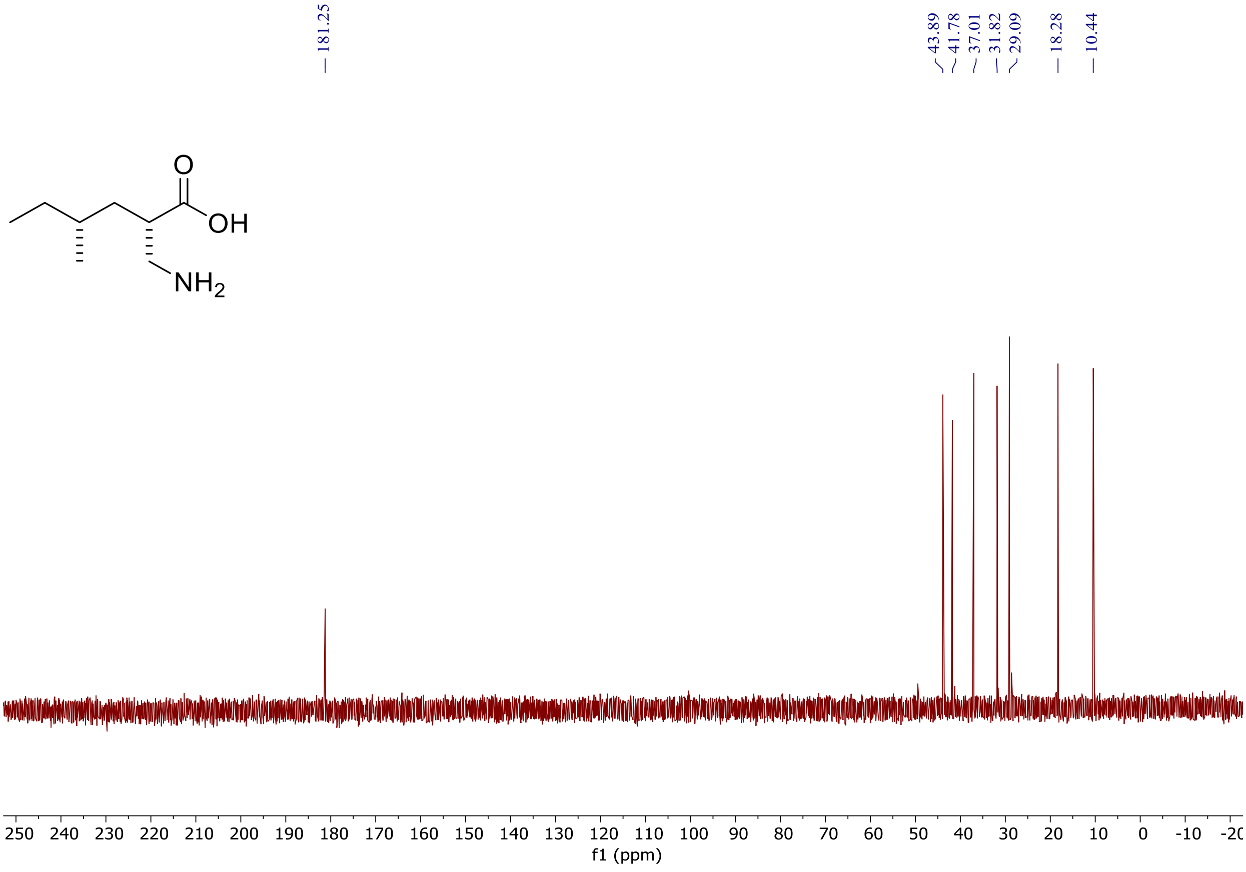
181.25
43.89
41.78
37.01
31.82
29.09
18.28
10.44
OH
NH2
f1 (ppm)

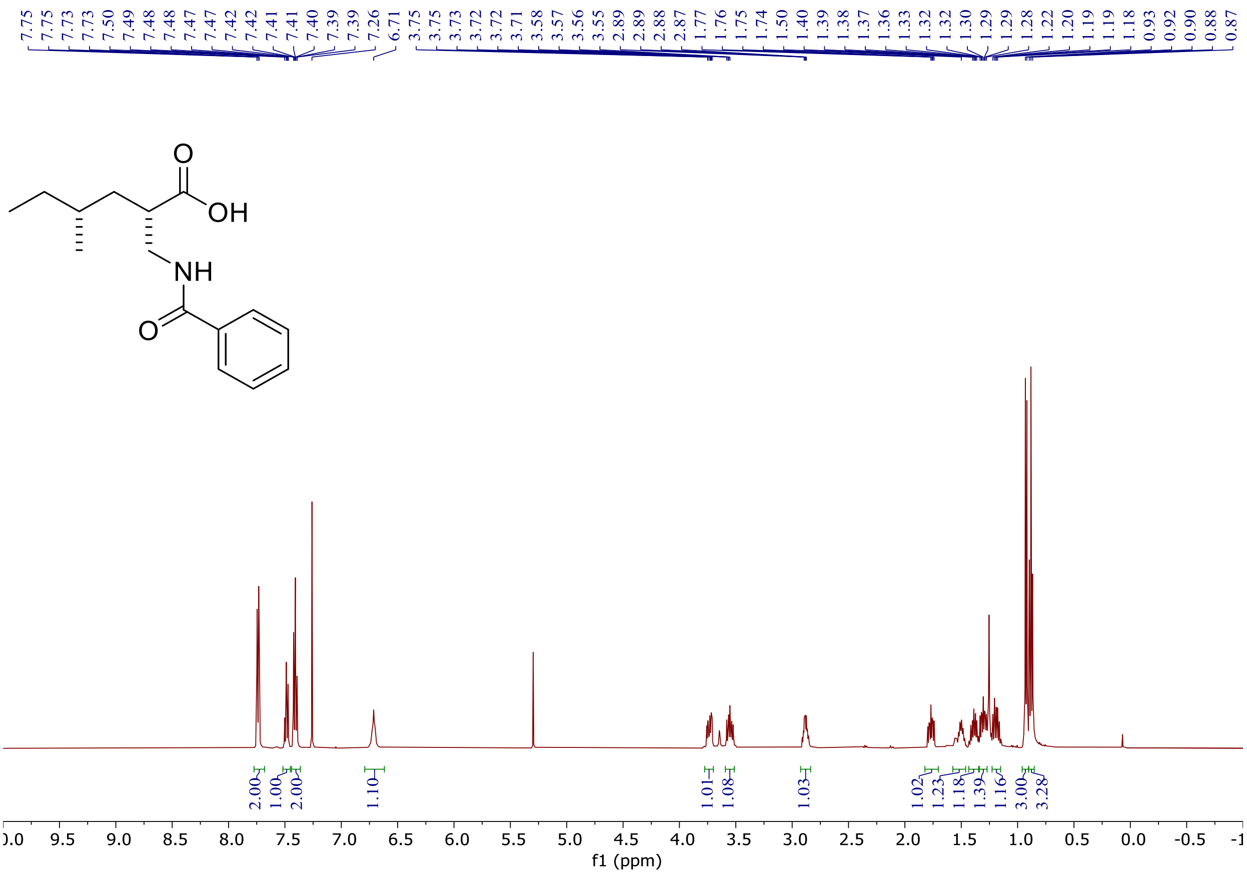
7.75 7.75 7.73 7.73 7.50 7.49 7.48 7.48 7.47 7.47 7.42 7.42 7.41 7.41 7.40 7.39 7.39 7.26 6.71 3.75 3.75 3.73 3.72 3.72 3.71 3.58 3.57 3.56 3.55 2.89 2.89 2.88 2.87 1.77 1.76 1.75 1.74 1.50 1.40 1.39 1.38 1.37 1.36 1.33 1.32 1.30 1.29 1.29 1.28 1.22 1.20 1.19 1.19 1.18 0.93 0.92 0.90 0.88 0.87
O
OH
NH
O
2.00 1.00 2.00 1.10 1.01 1.08 1.03 1.02 1.23 1.18 1.39 1.16 3.00 3.28
f1 (ppm)

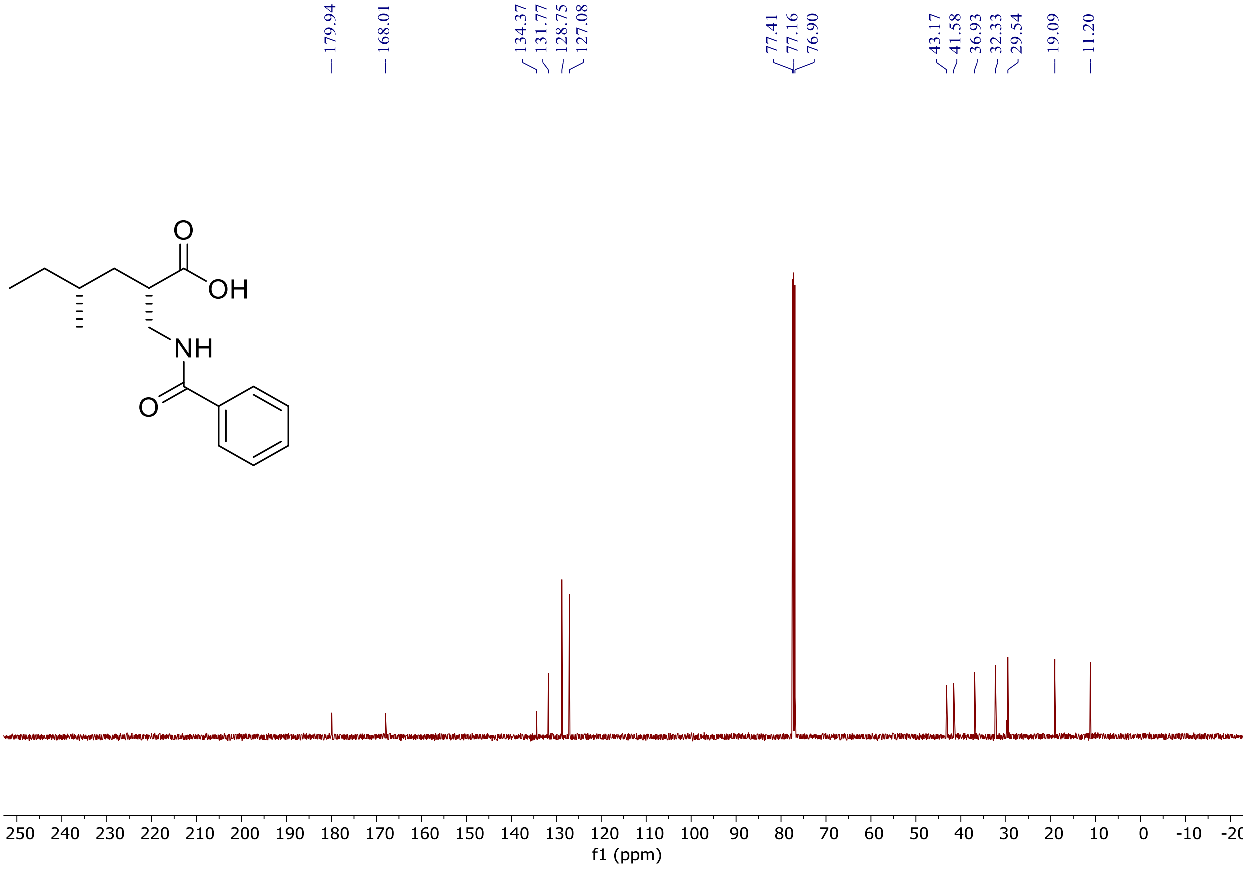
179.94
168.01
134.37 131.77 128.75 127.08
77.41 77.16 76.90
43.17 41.58 36.93 32.33 29.54 19.09 11.20
O
OH
NH
O
f1 (ppm)

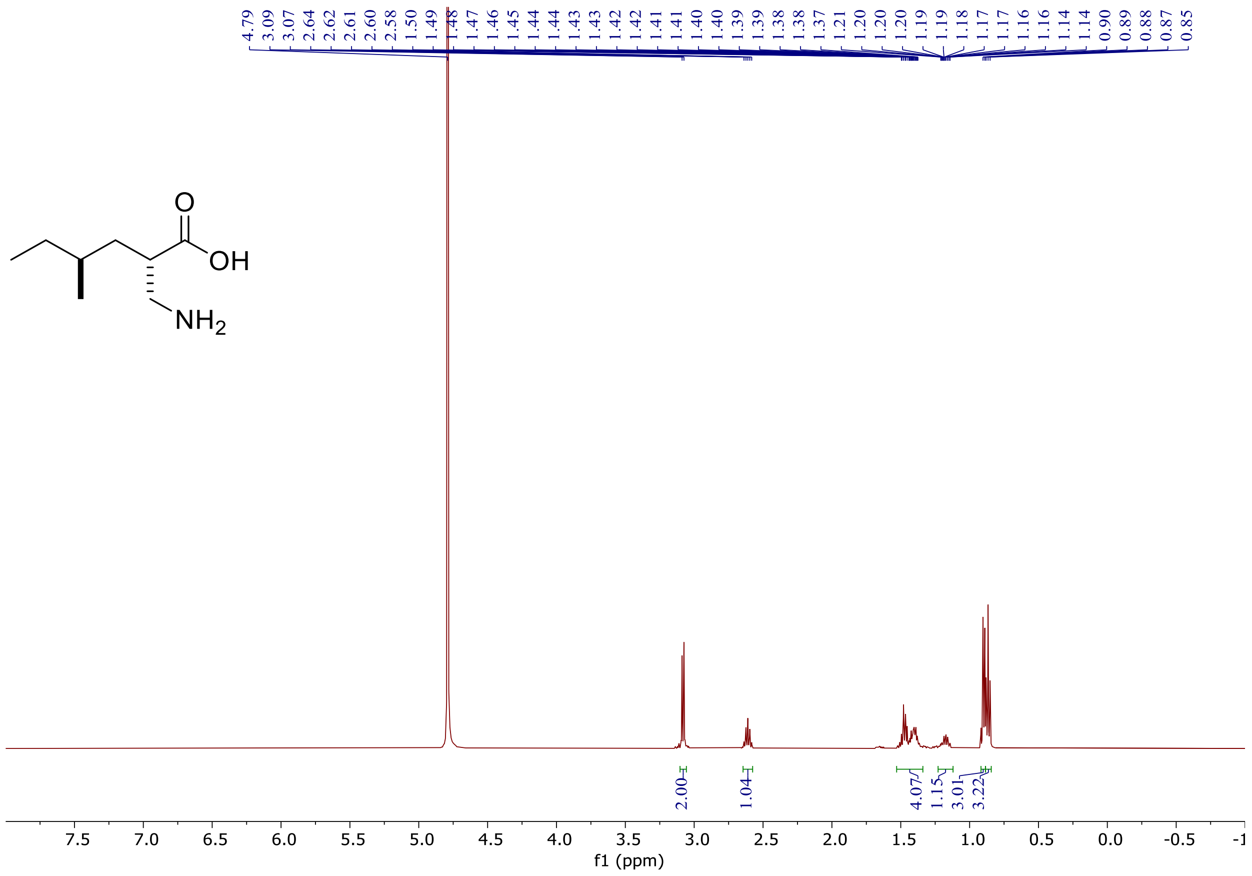
4.79
3.09
3.07
2.64
2.62
2.61
2.60
2.58
1.50
1.49
1.48
1.47
1.46
1.45
1.44
1.44
1.43
1.43
1.42
1.42
1.41
1.41
1.40
1.40
1.39
1.39
1.38
1.38
1.37
1.21
1.20
1.20
1.19
1.19
1.18
1.17
1.17
1.16
1.16
1.14
1.14
0.90
0.89
0.88
0.87
0.85
O
OH
NH2
2.00
1.04
4.07
1.15
3.01
3.22
f1 (ppm)

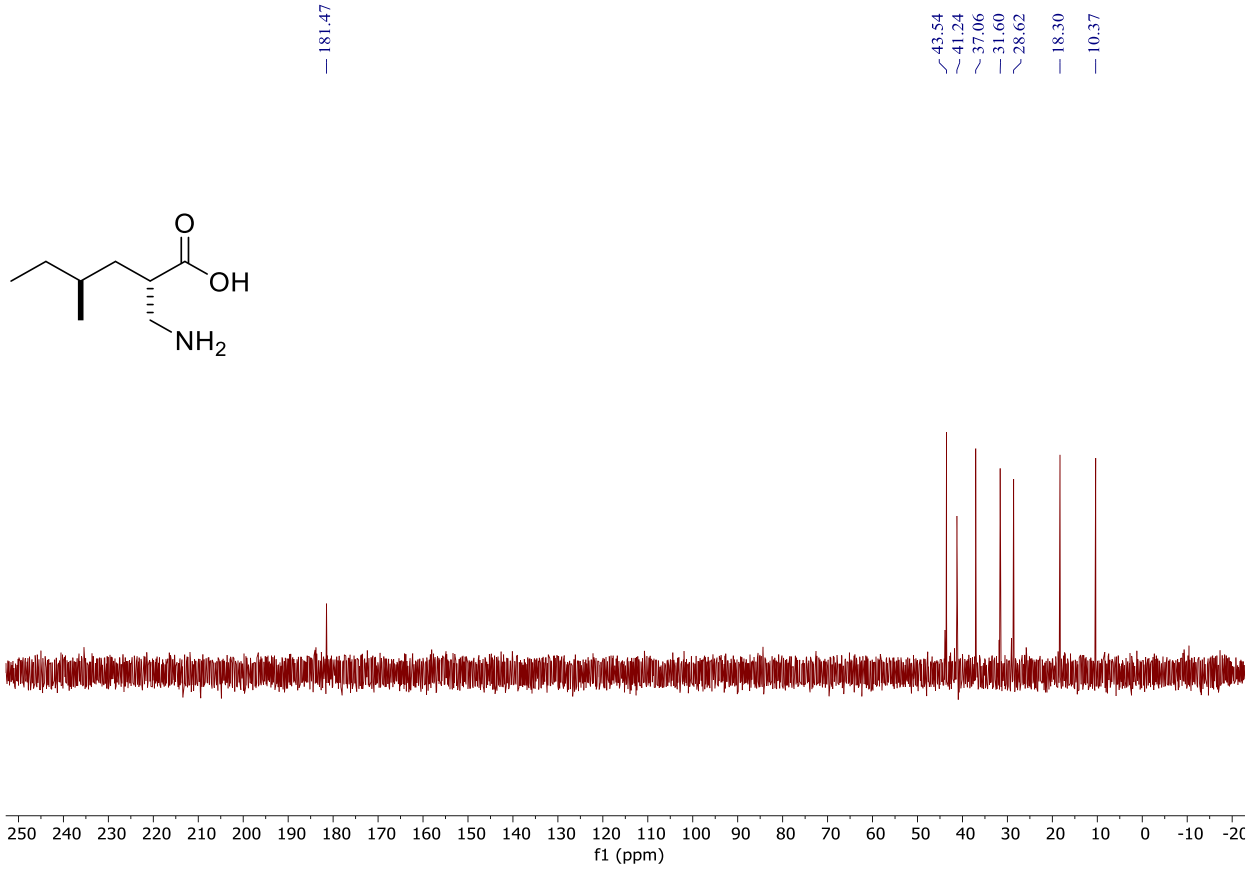
181.47
43.54
41.24
37.06
31.60
28.62
18.30
10.37
O
OH
NH2
f1 (ppm)

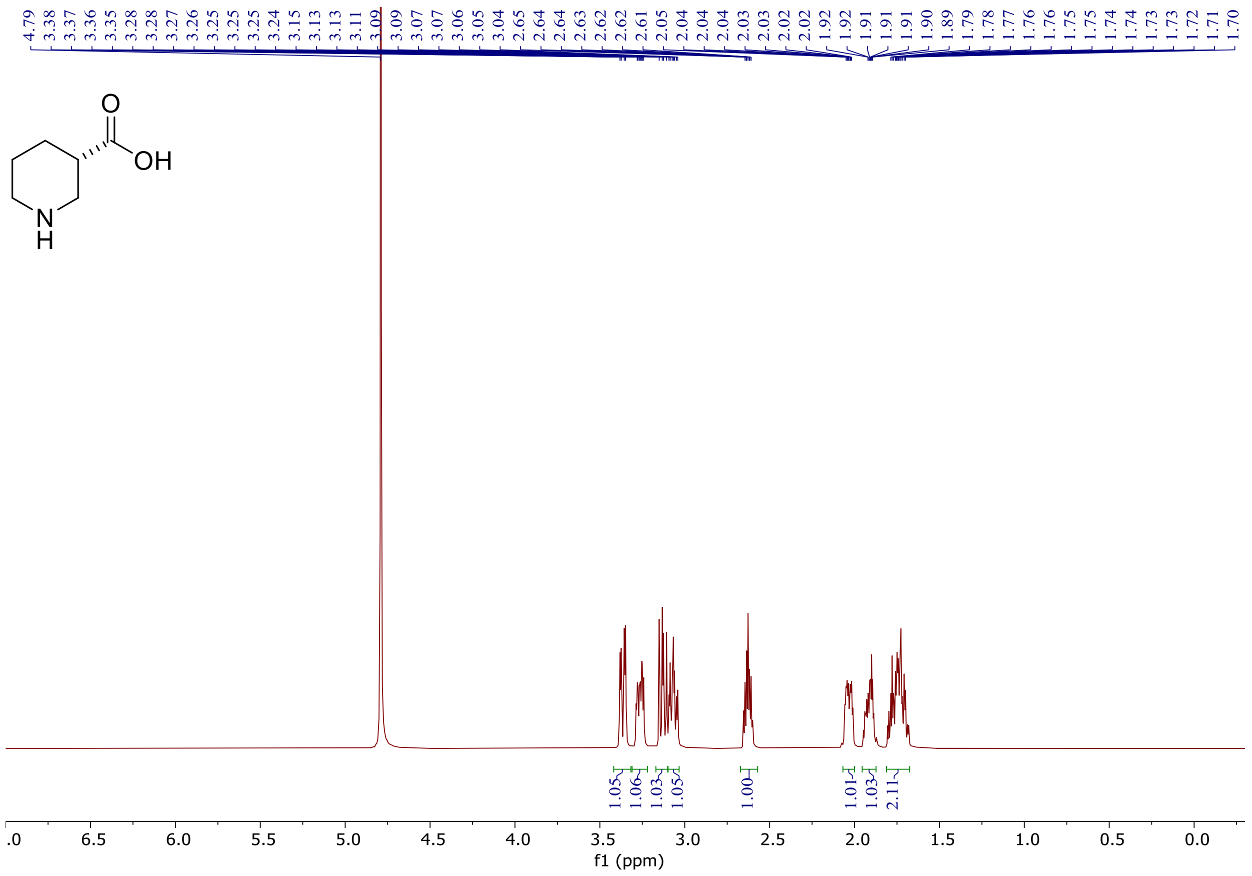

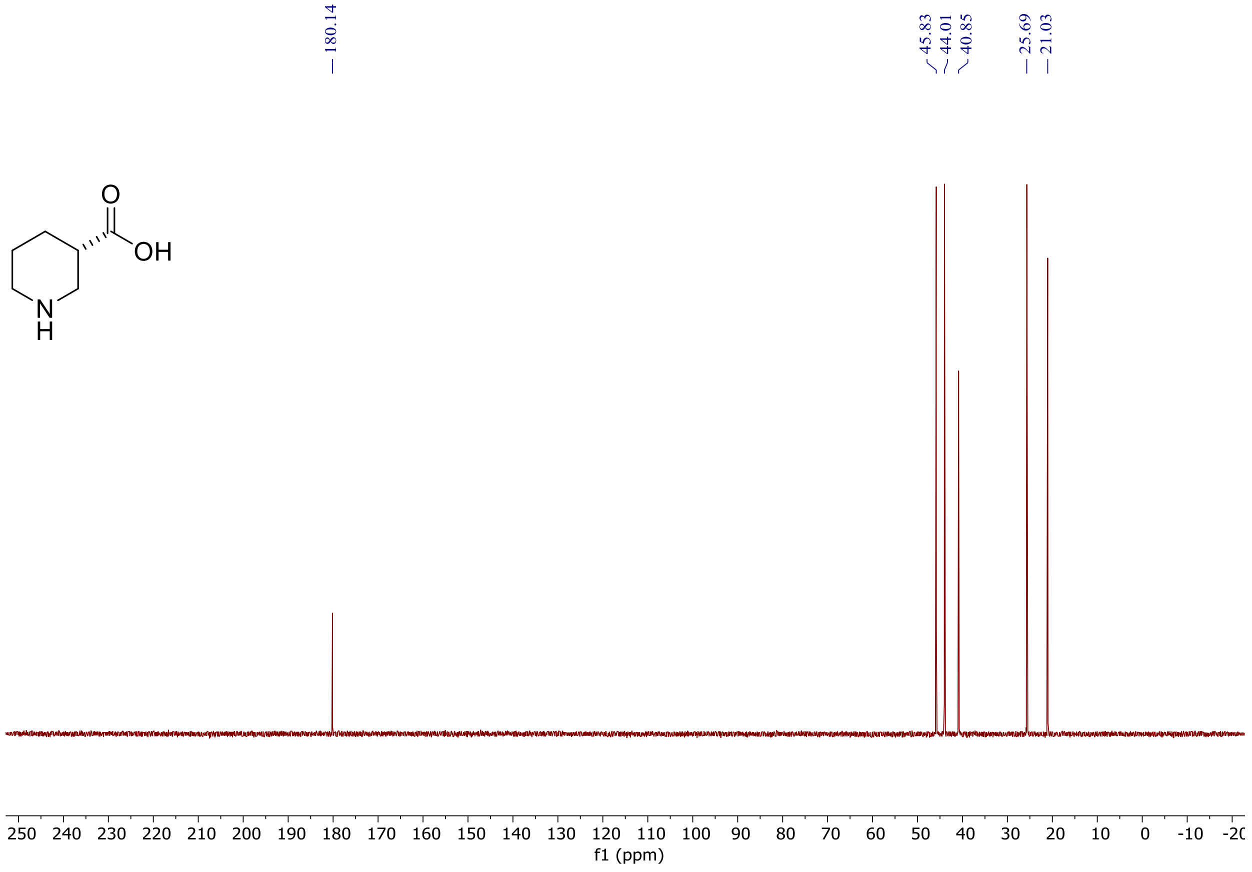

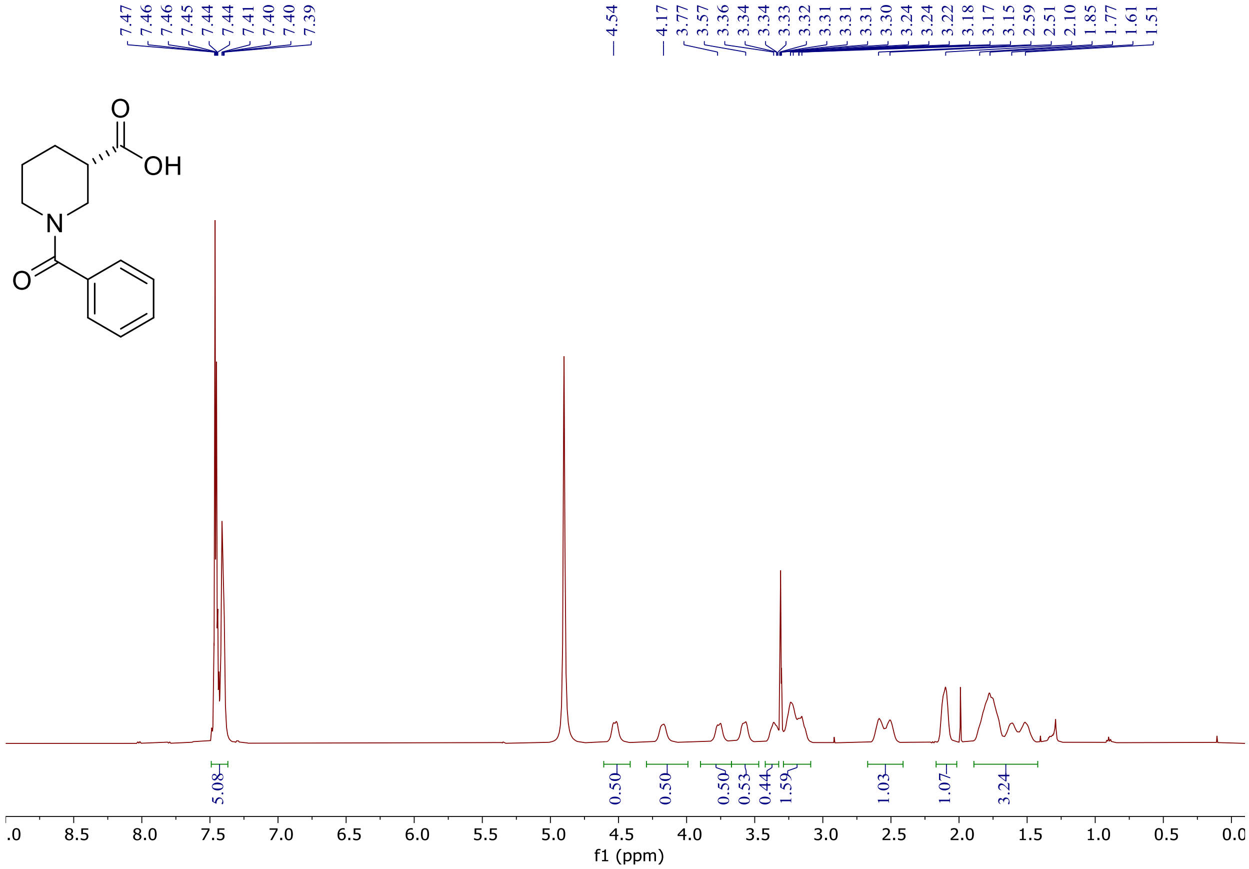
7.47 7.46 7.46 7.45 7.44 7.44 7.41 7.40 7.40 7.39
4.54
4.17 3.77 3.57 3.36 3.34 3.34 3.33 3.32 3.31 3.31 3.31 3.30 3.24 3.24 3.22 3.18 3.17 3.15 2.59 2.51 2.10 1.85 1.77 1.61 1.51
O
OH
N
O
5.08
0.50
0.50
0.50
0.53
0.44
1.59
1.03
1.07
3.24
f1 (ppm)

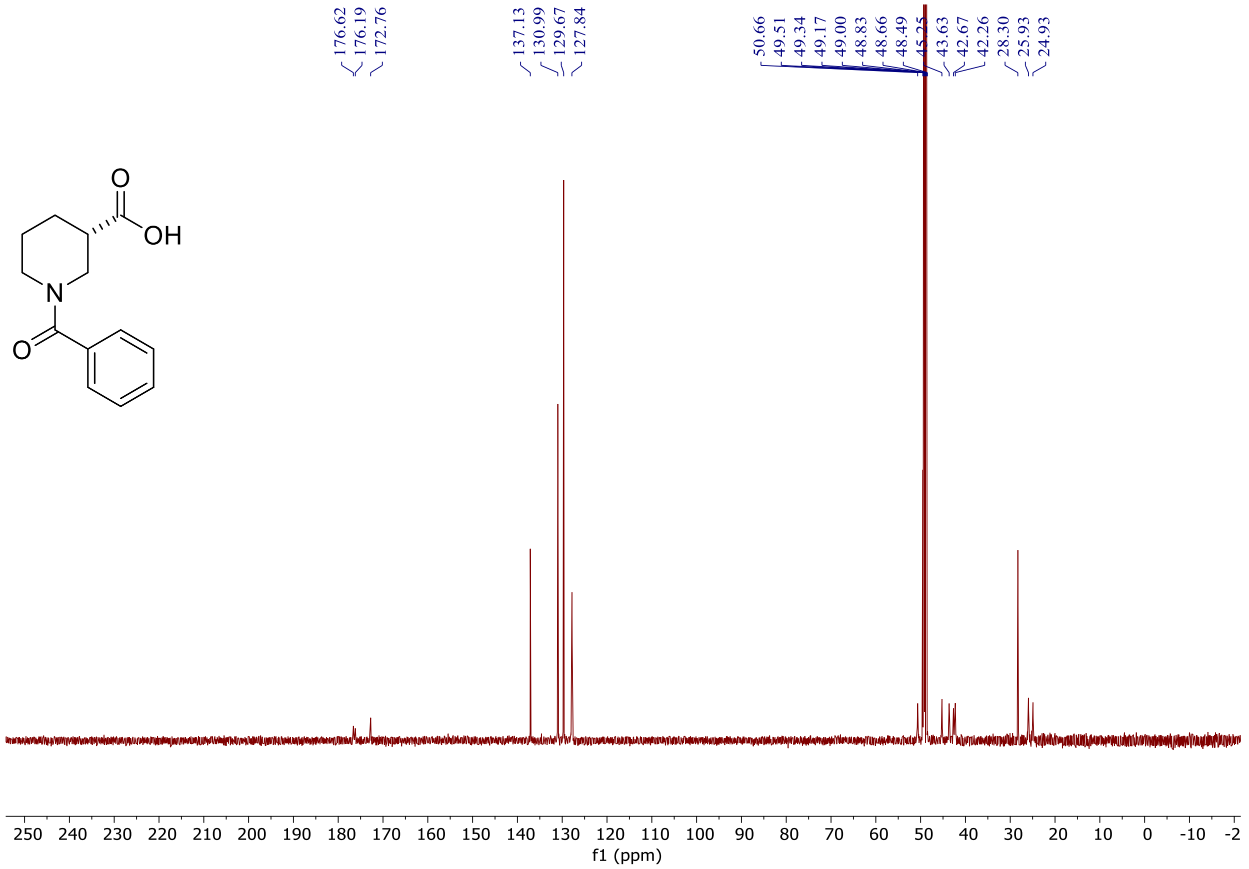
176.62 176.19 172.76
137.13 130.99 129.67 127.84
50.66 49.51 49.34 49.17 49.00 48.83 48.66 48.49 45.25 43.63 42.67 42.26 28.30 25.93 24.93
O
OH
N
O
f1 (ppm)

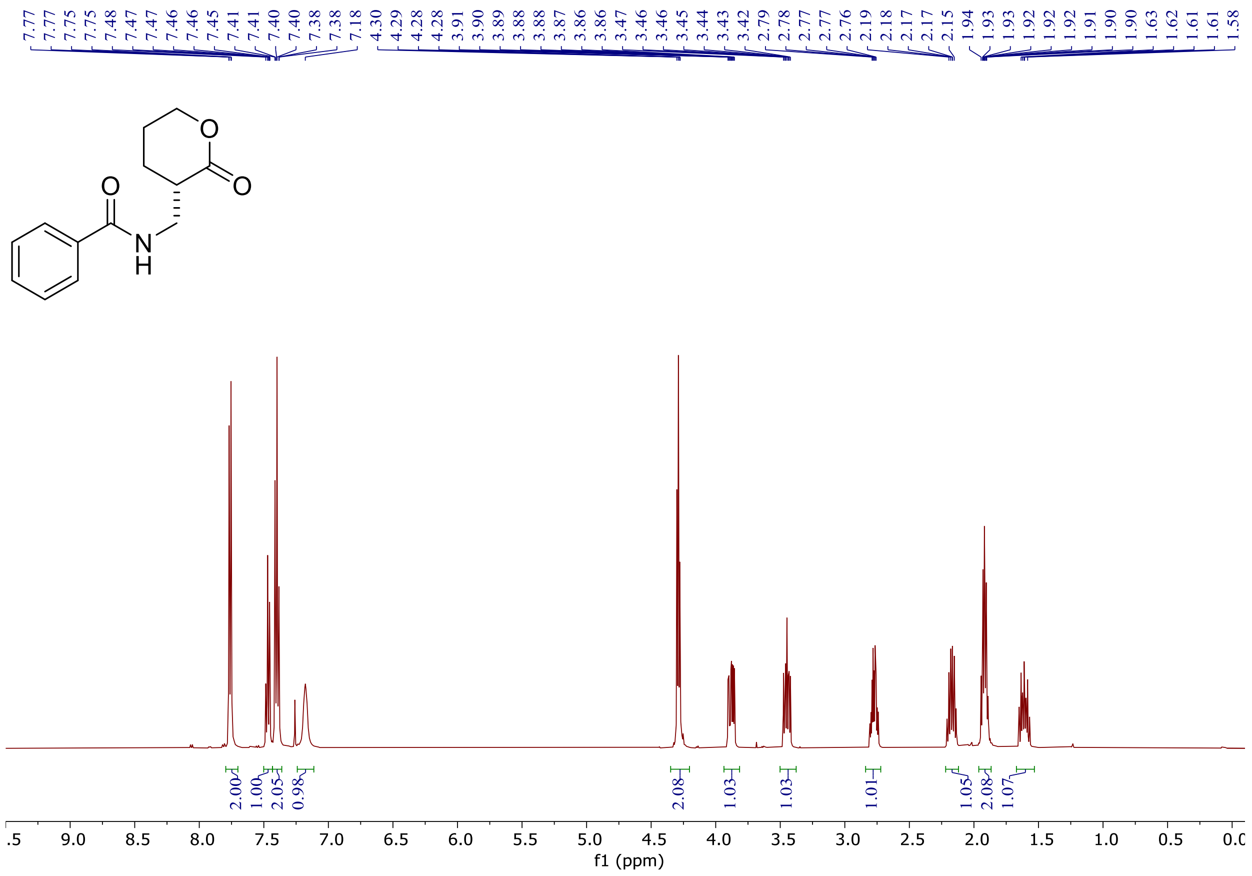
7.77 7.77 7.75 7.75 7.48 7.47 7.47 7.46 7.45 7.41 7.41 7.40 7.40 7.38 7.38 7.18 4.30 4.29 4.28 4.28 3.91 3.90 3.89 3.88 3.88 3.87 3.86 3.86 3.47 3.46 3.46 3.45 3.44 3.43 3.42 2.79 2.78 2.77 2.77 2.76 2.19 2.18 2.17 2.17 2.15 1.94 1.93 1.93 1.92 1.92 1.92 1.91 1.90 1.90 1.63 1.62 1.61 1.61 1.58
O
O
O
N
H
2.00
1.00
2.05
0.98
2.08
1.03
1.03
1.01
1.05
2.08
1.07
f1 (ppm)

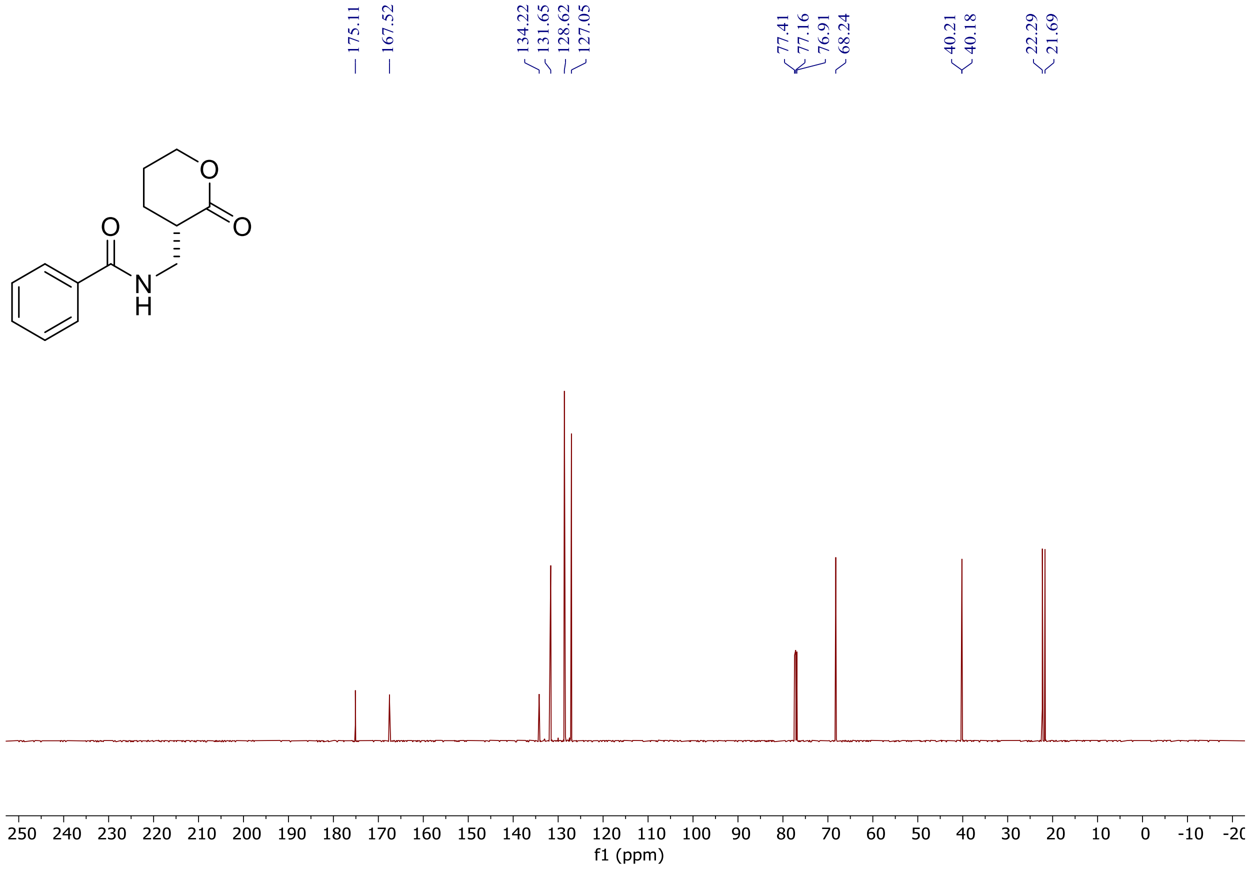
175.11
167.52
134.22 131.65 128.62 127.05
77.41 77.16 76.91 68.24
40.21 40.18
22.29 21.69
O
O
O
N
H
f1 (ppm)

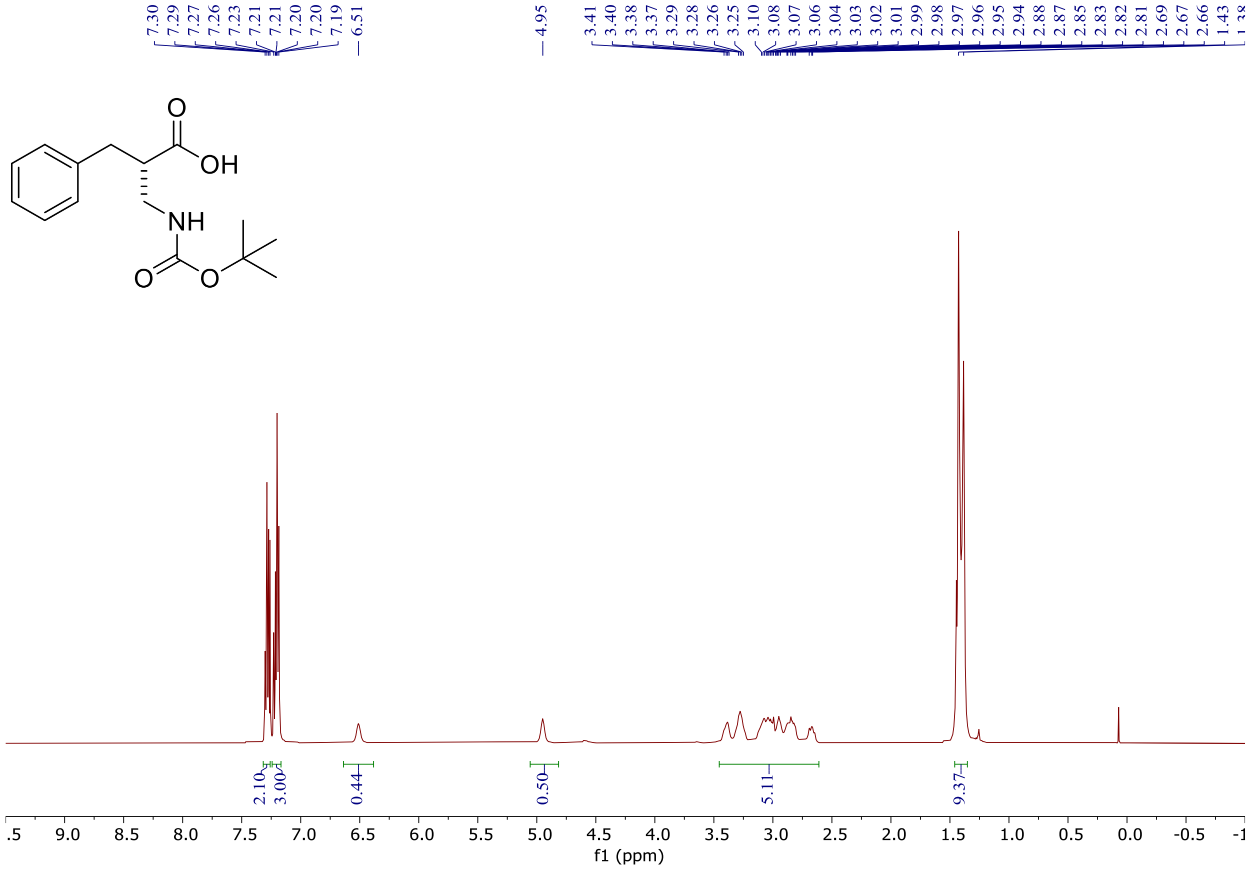

7.30
7.29
7.27
7.26
7.23
7.21
7.21
7.20
7.20
7.19
6.51
4.95
3.41
3.40
3.38
3.37
3.29
3.28
3.26
3.25
3.10
3.08
3.07
3.06
3.04
3.03
3.02
3.01
2.99
2.98
2.97
2.96
2.95
2.94
2.88
2.87
2.85
2.83
2.82
2.81
2.69
2.67
2.66
1.43
O
OH
NH
O
O
2.10
3.00
0.44
0.50
5.11
9.37
f1 (ppm)

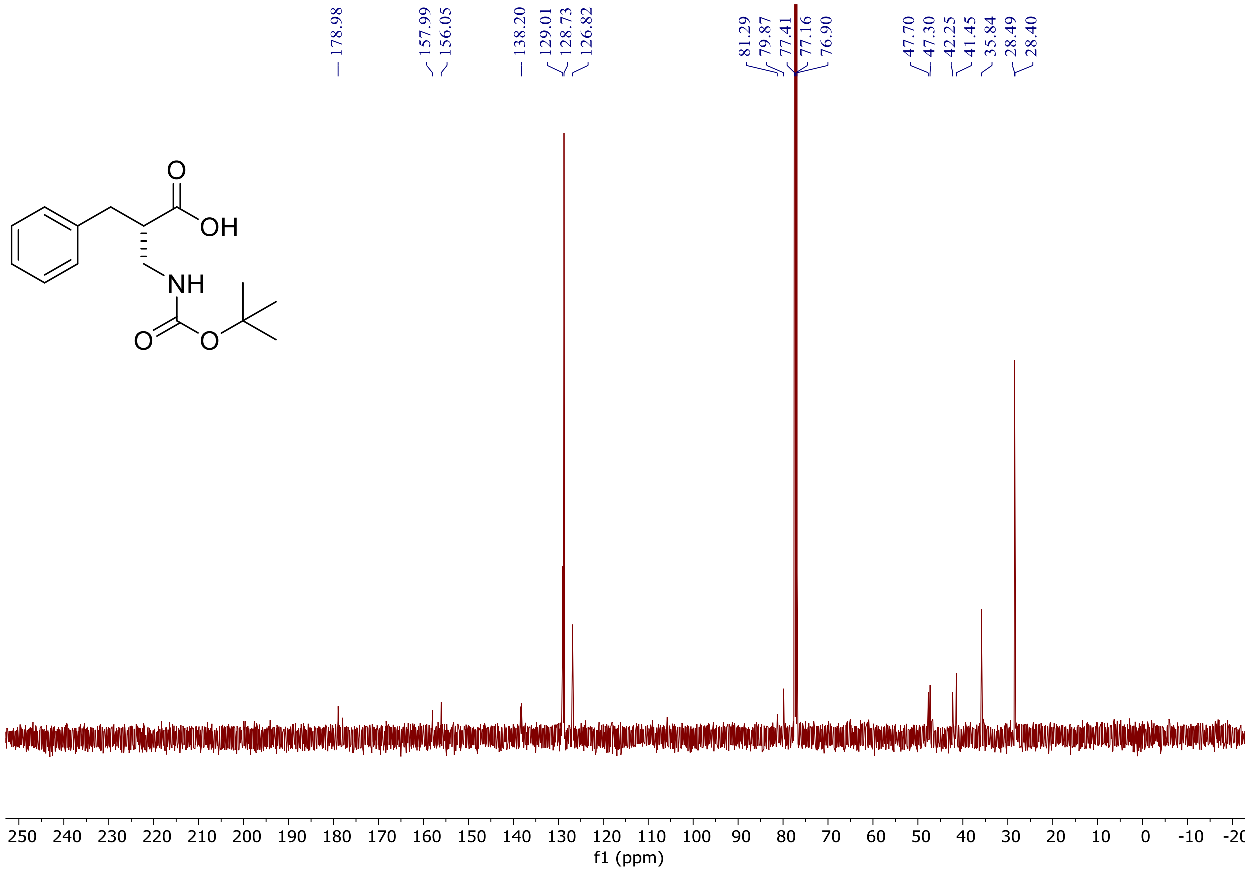

178.98
157.99
156.05
138.20
129.01
128.73
126.82
81.29
79.87
77.41
77.16
76.90
47.70
47.30
42.25
41.45
35.84
28.49
28.40
O
OH
NH
O
O
f1 (ppm)

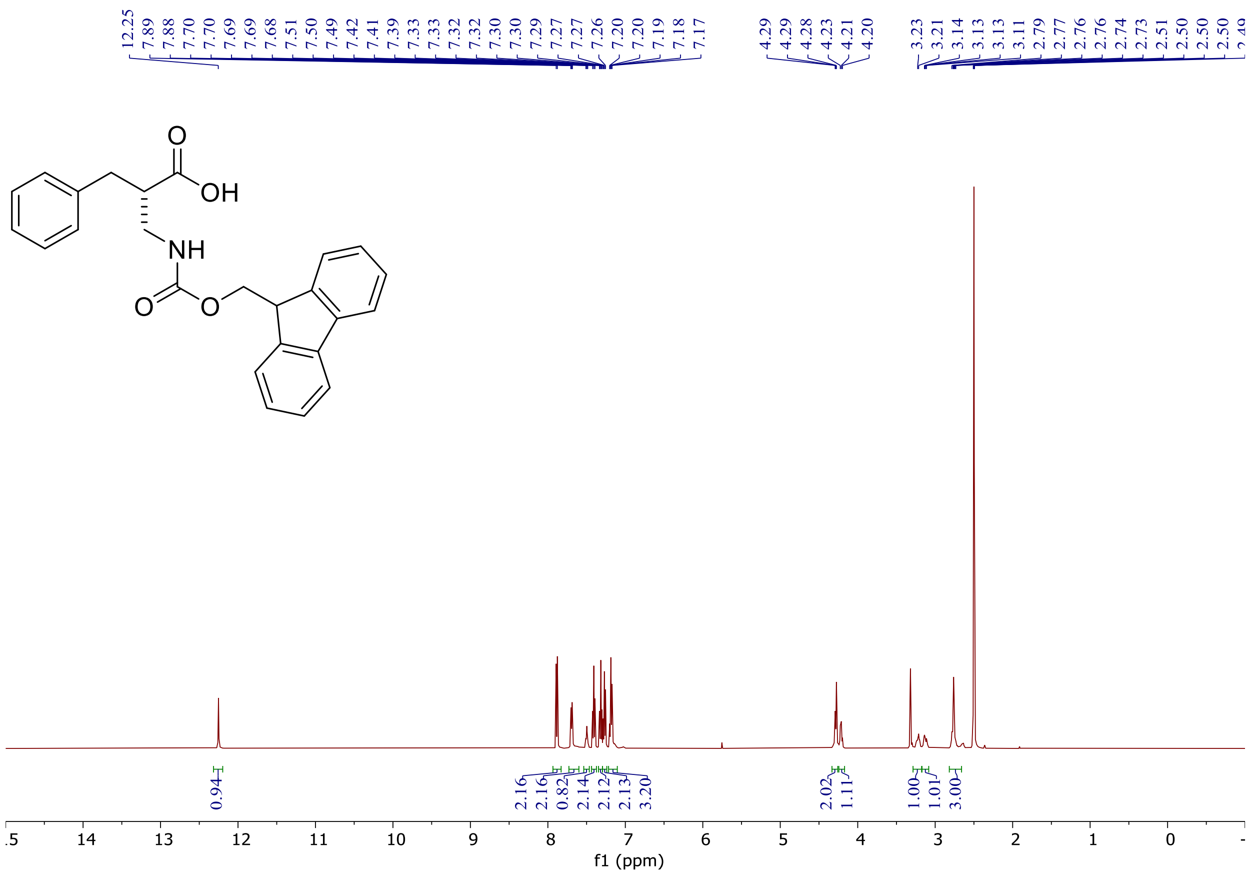

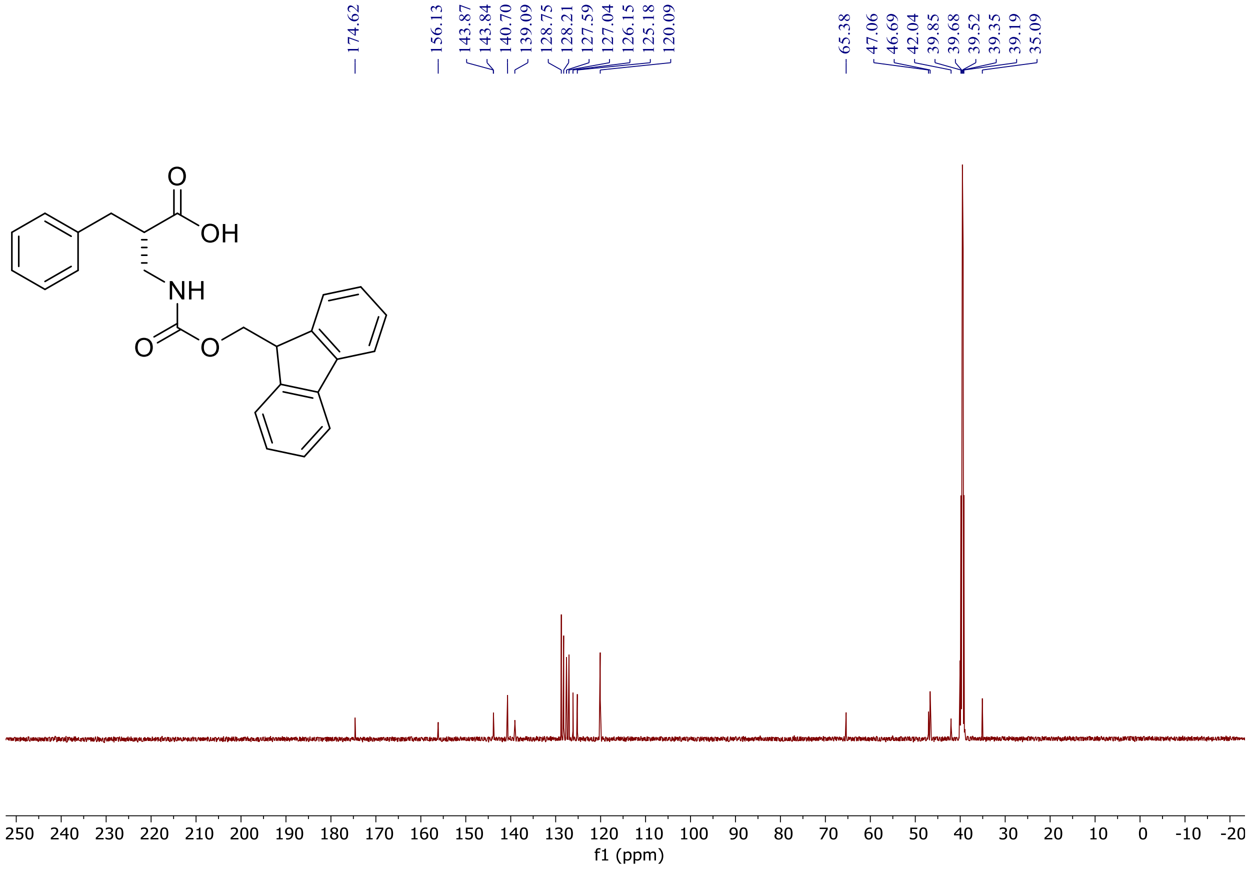

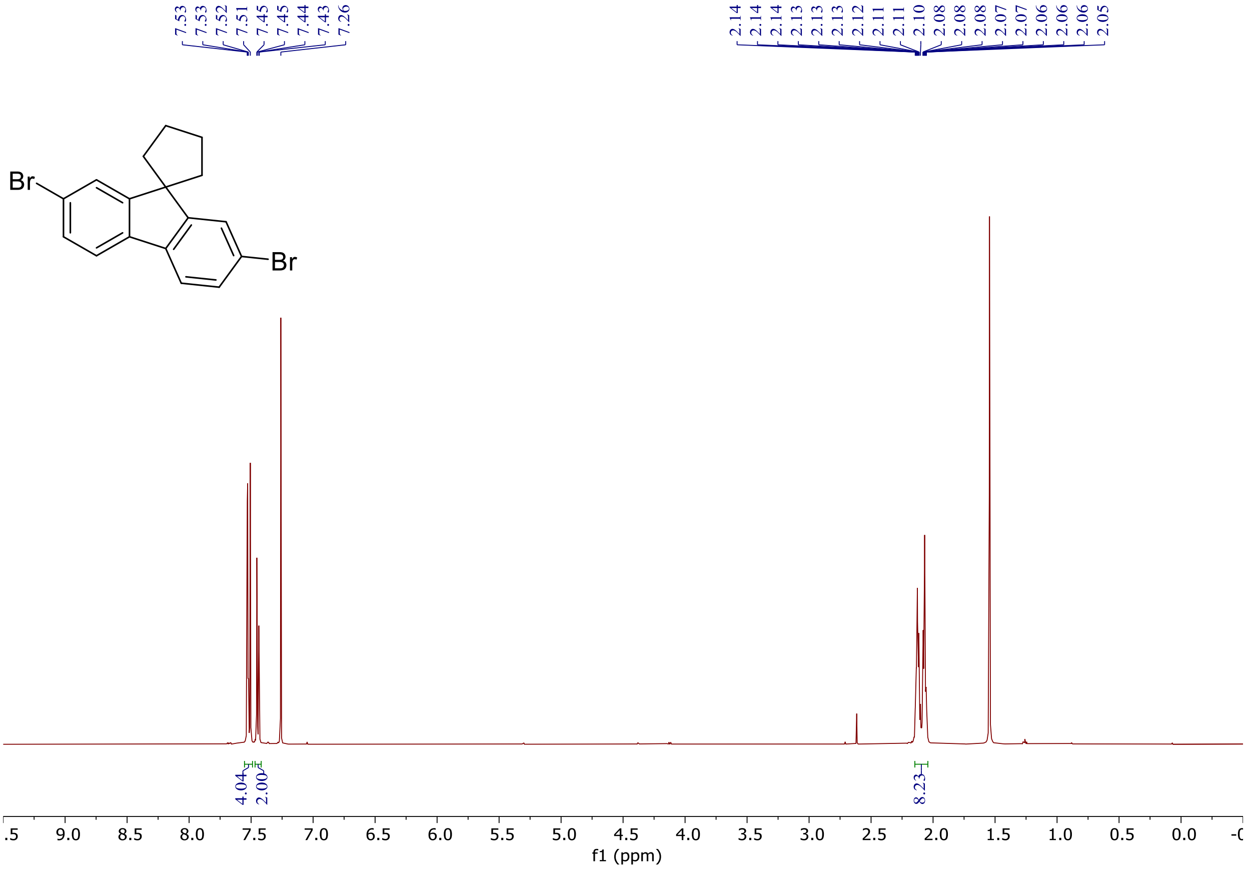
7.53 7.53 7.52 7.51 7.45 7.45 7.44 7.43 7.26
2.14 2.14 2.14 2.13 2.13 2.12 2.11 2.11 2.10 2.08 2.08 2.07 2.07 2.06 2.06 2.05
Br
Br
4.04
2.00
8.23
9.0 8.5 8.0 7.5 7.0 6.5 6.0 5.5 5.0 4.5 4.0 3.5 3.0 2.5 2.0 1.5 1.0 0.5 0.0
f1 (ppm)

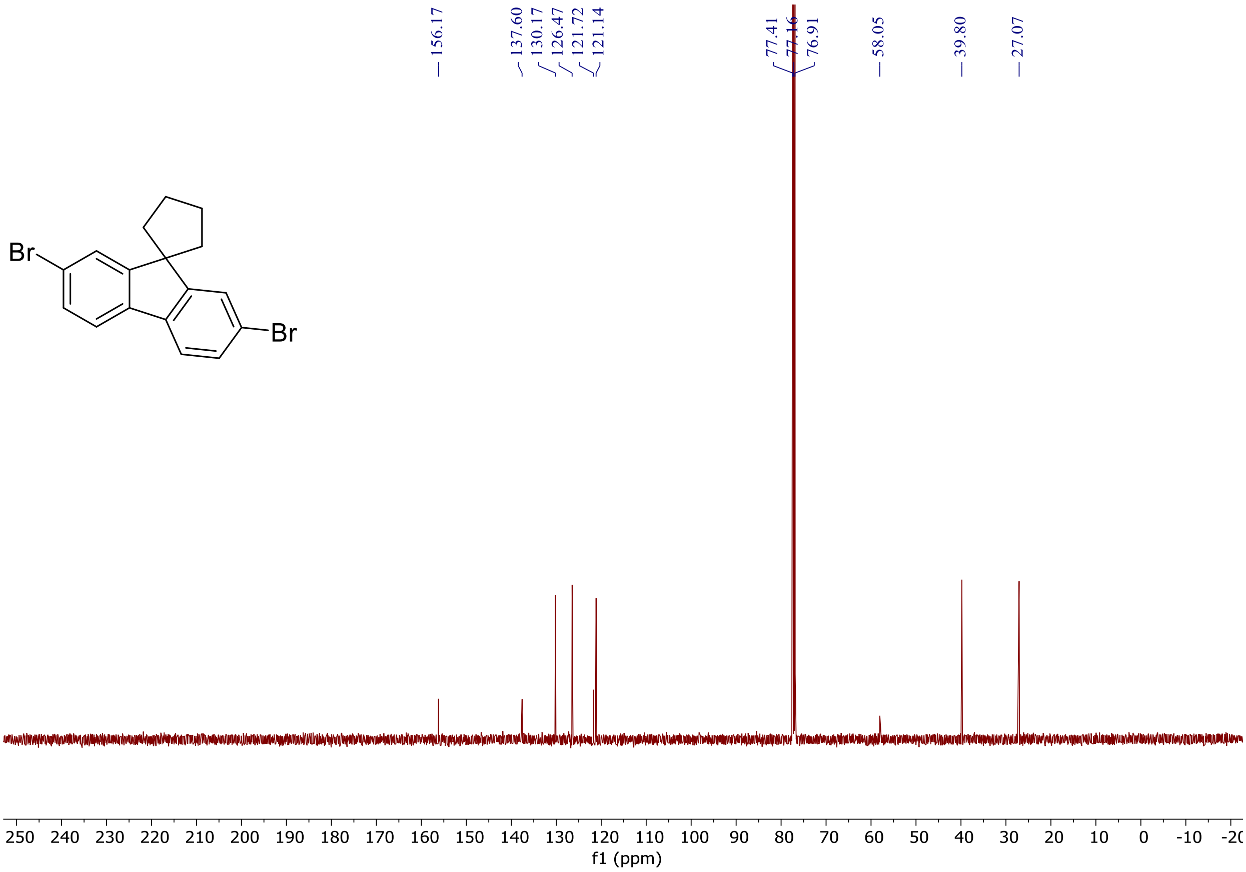
156.17
137.60 130.17 126.47 121.72 121.14
77.41 77.16 76.91
58.05
39.80
27.07
Br
Br
250 240 230 220 210 200 190 180 170 160 150 140 130 120 110 100 90 80 70 60 50 40 30 20 10 0 -10
f1 (ppm)

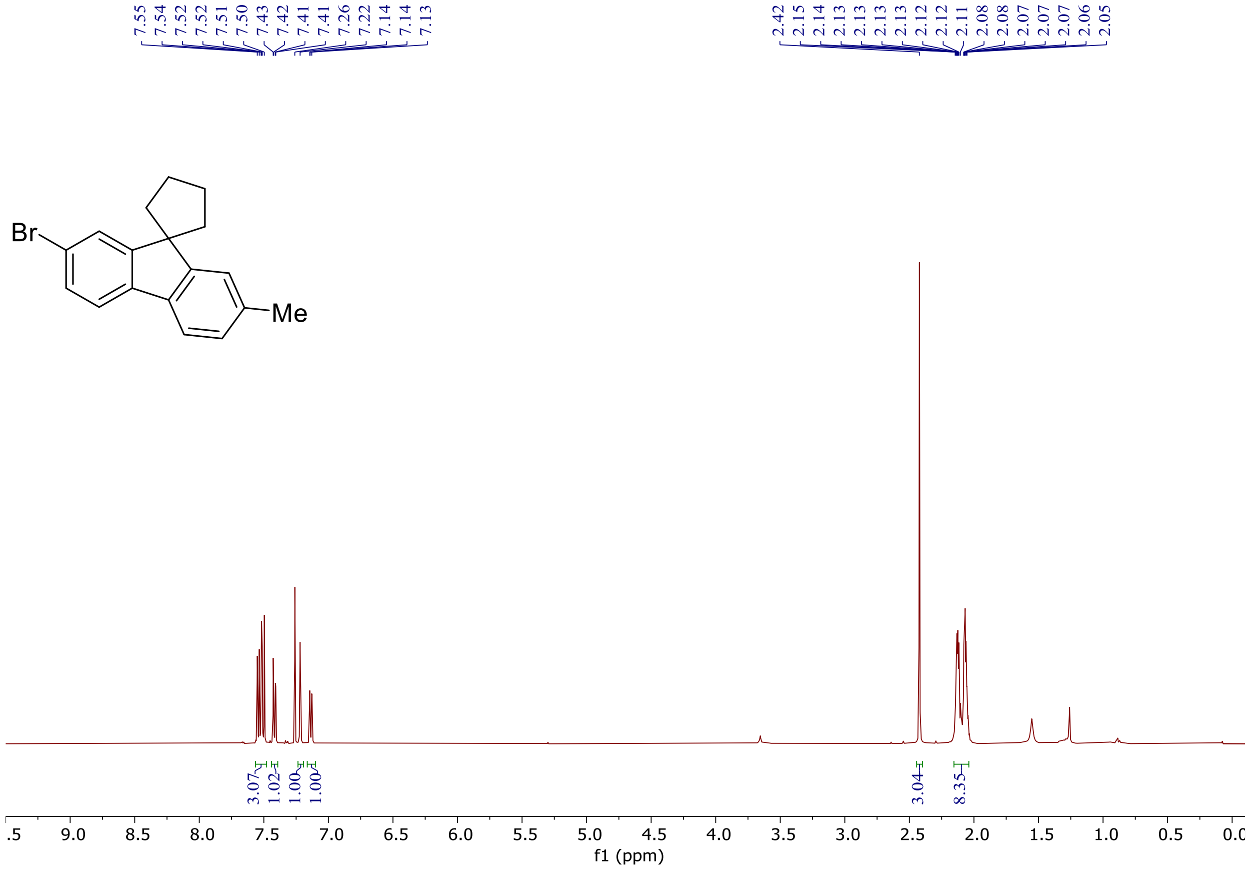

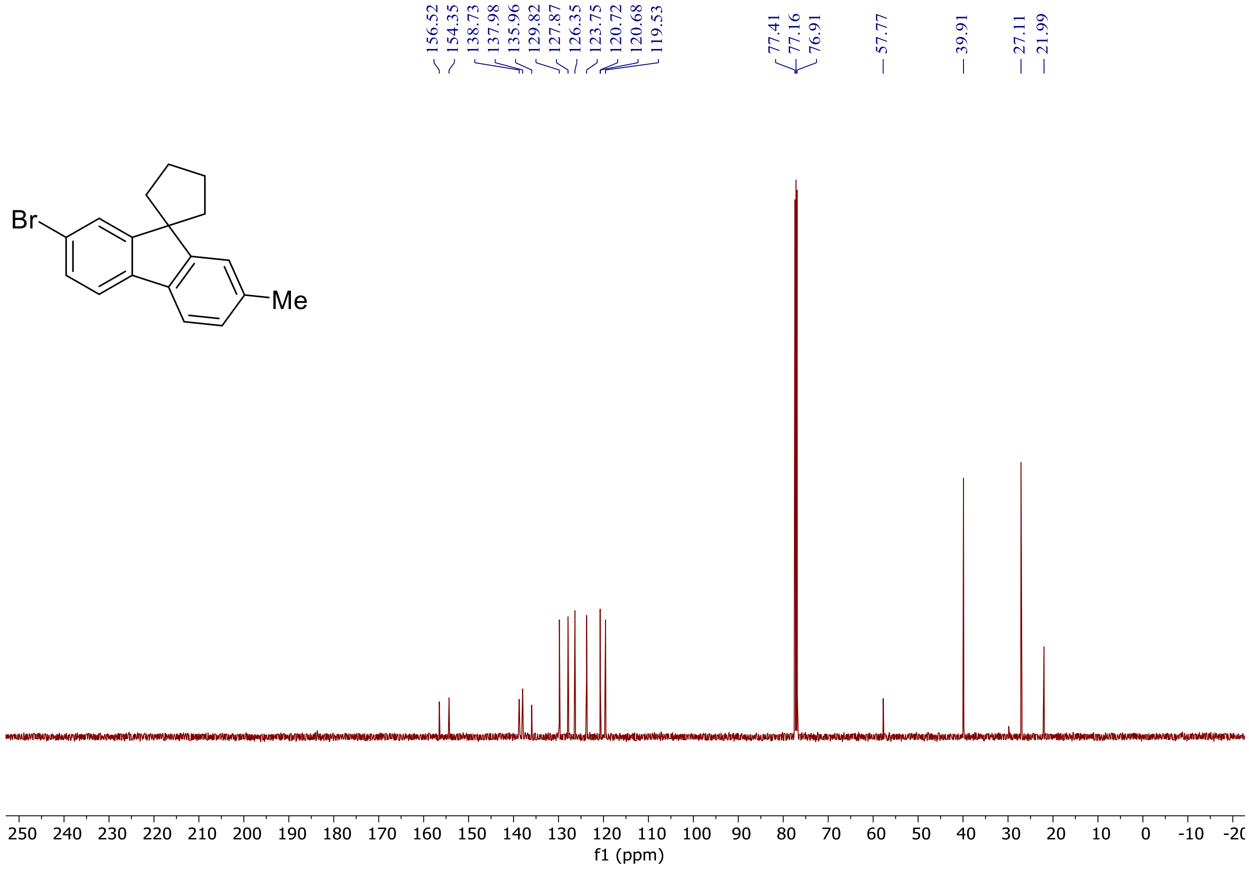

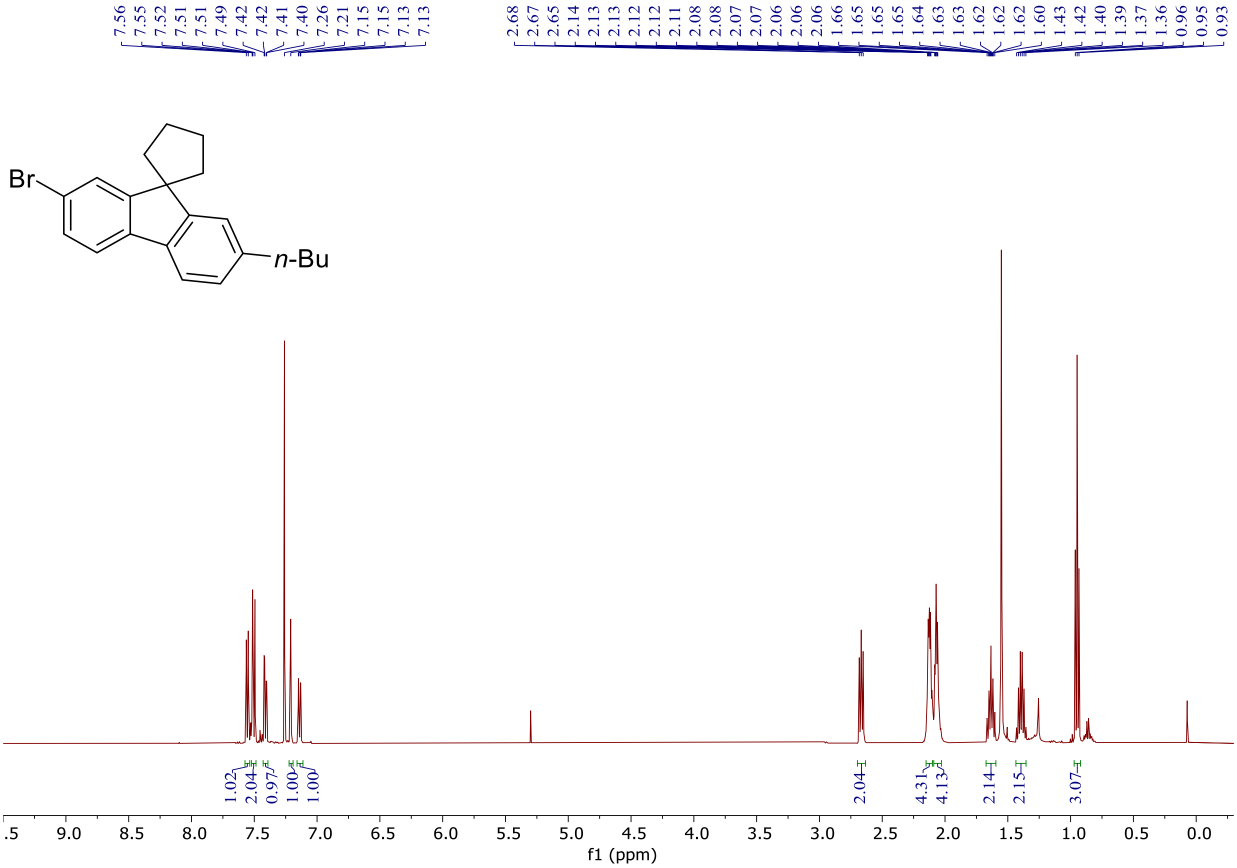

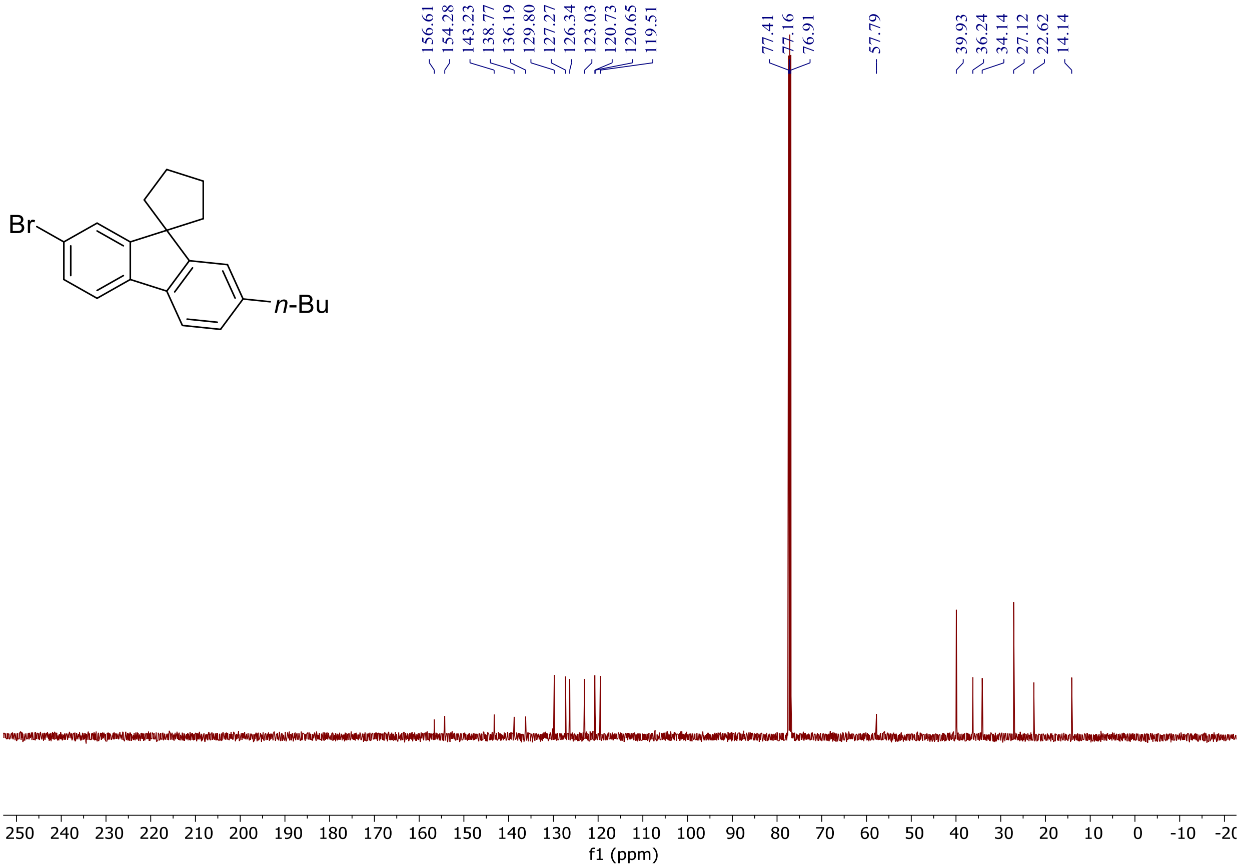

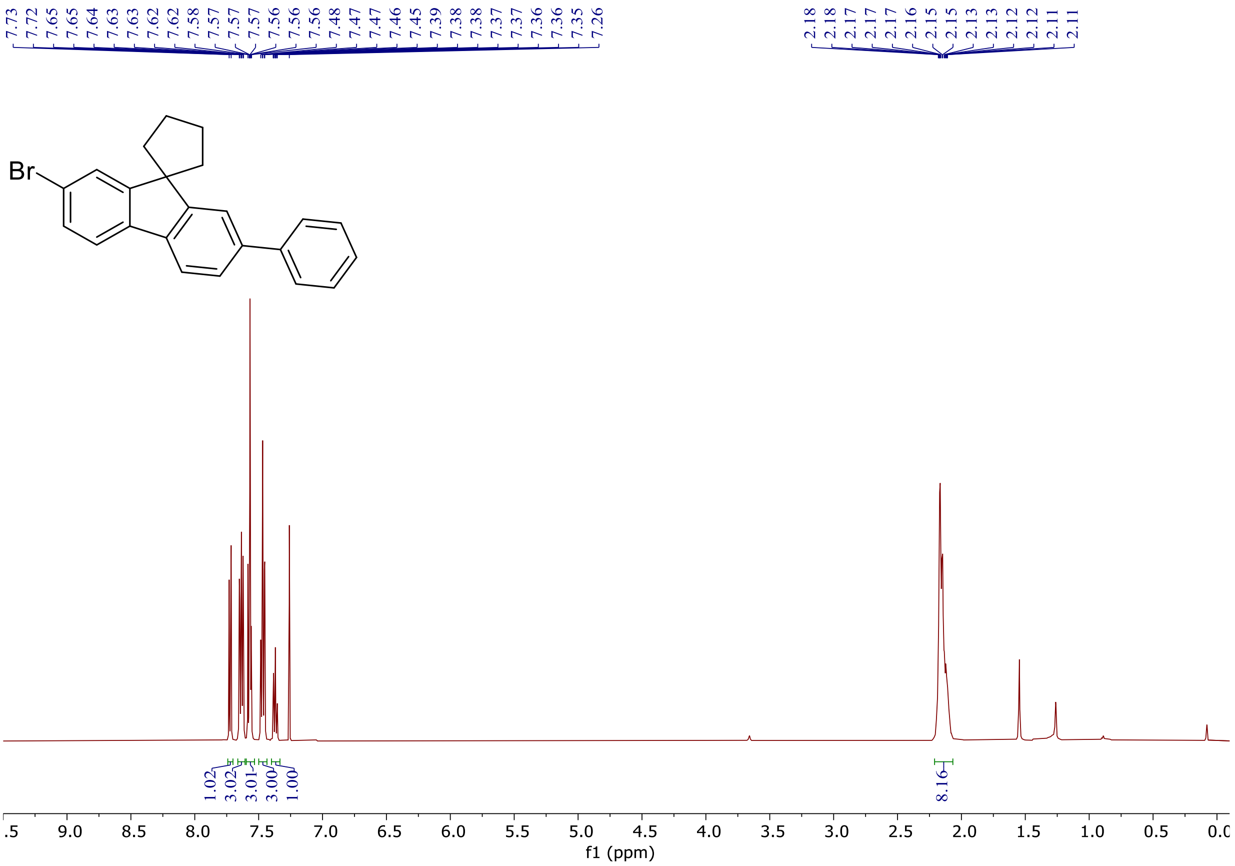
7.73 7.72 7.65 7.64 7.63 7.63 7.62 7.62 7.58 7.57 7.57 7.57 7.56 7.56 7.56 7.48 7.47 7.47 7.46 7.45 7.39 7.38 7.38 7.37 7.37 7.36 7.36 7.35 7.26
2.18 2.18 2.17 2.17 2.17 2.16 2.15 2.15 2.13 2.13 2.12 2.12 2.11 2.11
Br
1.02 3.02 3.01 3.00 1.00
8.16
.5 9.0 8.5 8.0 7.5 7.0 6.5 6.0 5.5 5.0 4.5 4.0 3.5 3.0 2.5 2.0 1.5 1.0 0.5 0.0
f1 (ppm)

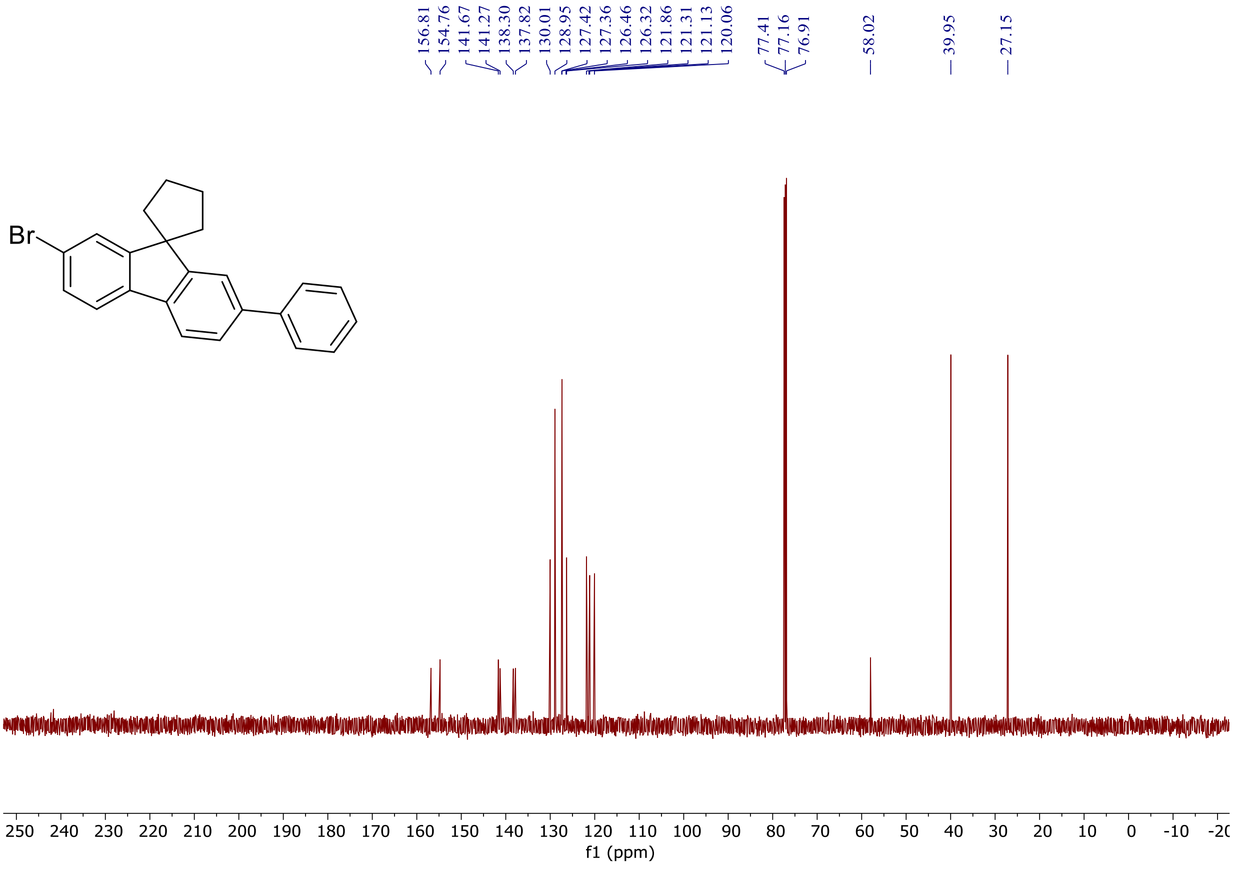
156.81 154.76 141.67 141.27 138.30 137.82 130.01 128.95 127.42 127.36 126.46 126.32 121.86 121.31 121.13 120.06
77.41 77.16 76.91
58.02
39.95
27.15
Br
250 240 230 220 210 200 190 180 170 160 150 140 130 120 110 100 90 80 70 60 50 40 30 20 10 0 -10 -20
f1 (ppm)

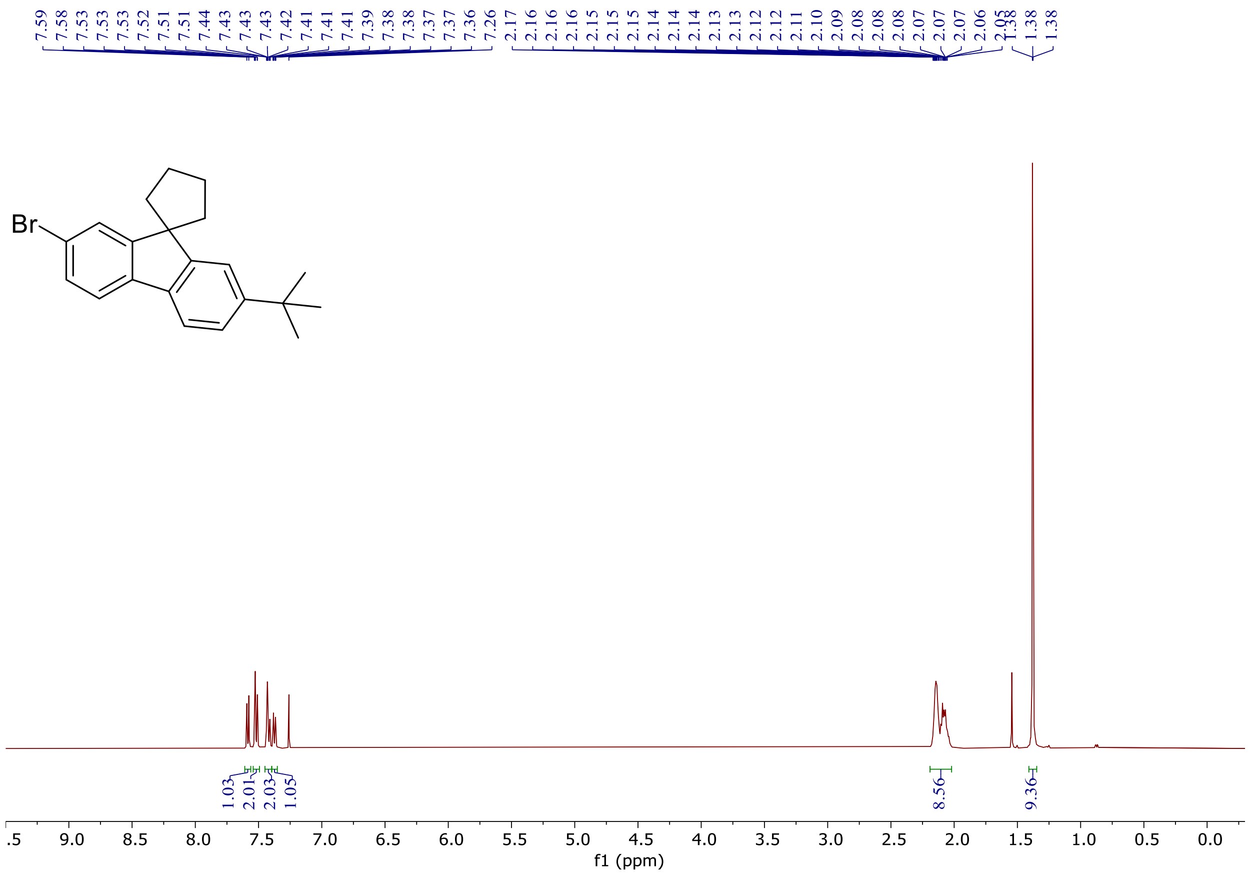
7.59
7.58
7.53
7.53
7.53
7.52
7.51
7.51
7.44
7.43
7.43
7.42
7.41
7.41
7.41
7.39
7.38
7.38
7.37
7.37
7.36
7.26
2.17
2.16
2.16
2.15
2.15
2.15
2.14
2.14
2.13
2.13
2.12
2.12
2.11
2.10
2.09
2.08
2.08
2.07
2.07
2.07
2.06
2.05
1.38
1.38
1.38
Br
1.03
2.01
2.03
1.05
8.56
9.36
f1 (ppm)

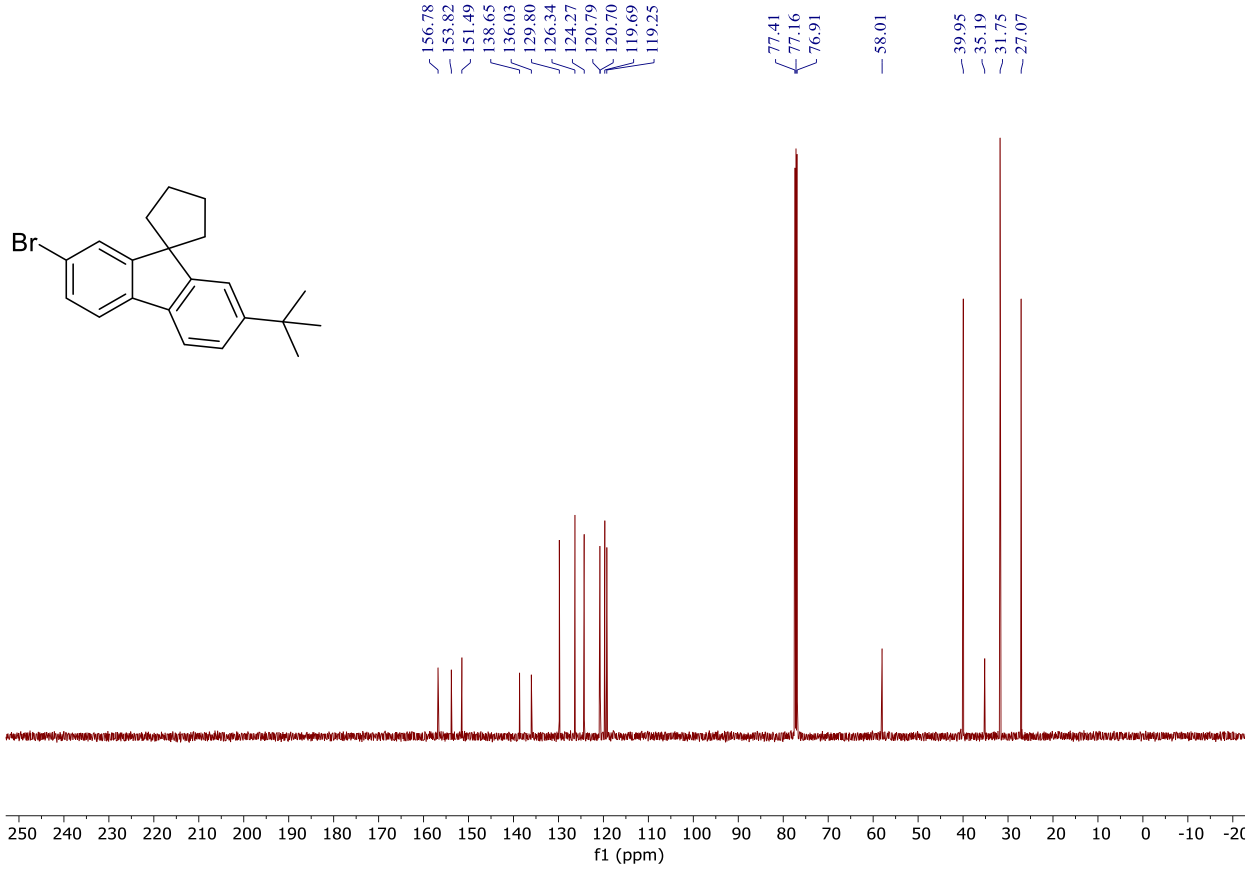
156.78
153.82
151.49
138.65
136.03
129.80
126.34
124.27
120.79
120.70
119.69
119.25
77.41
77.16
76.91
58.01
39.95
35.19
31.75
27.07
Br
f1 (ppm)

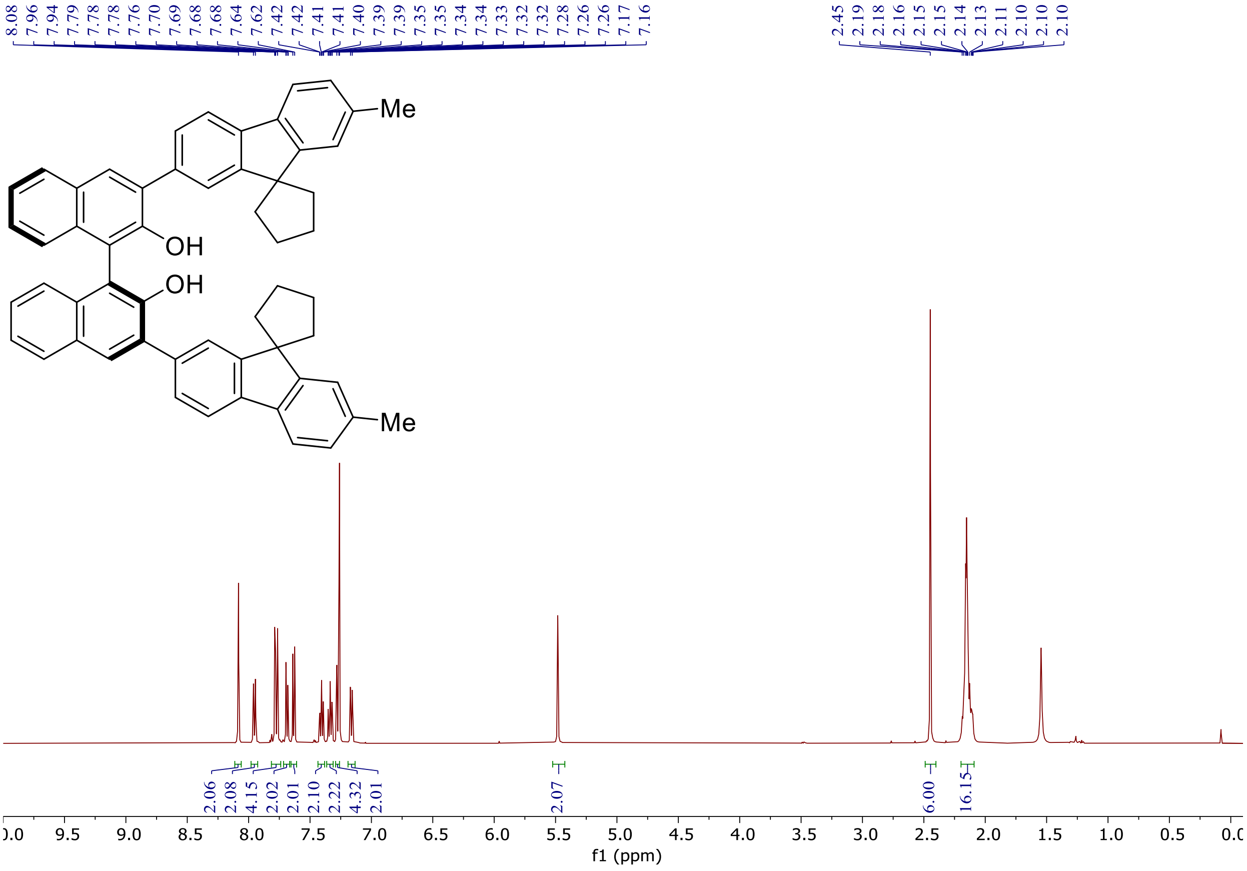

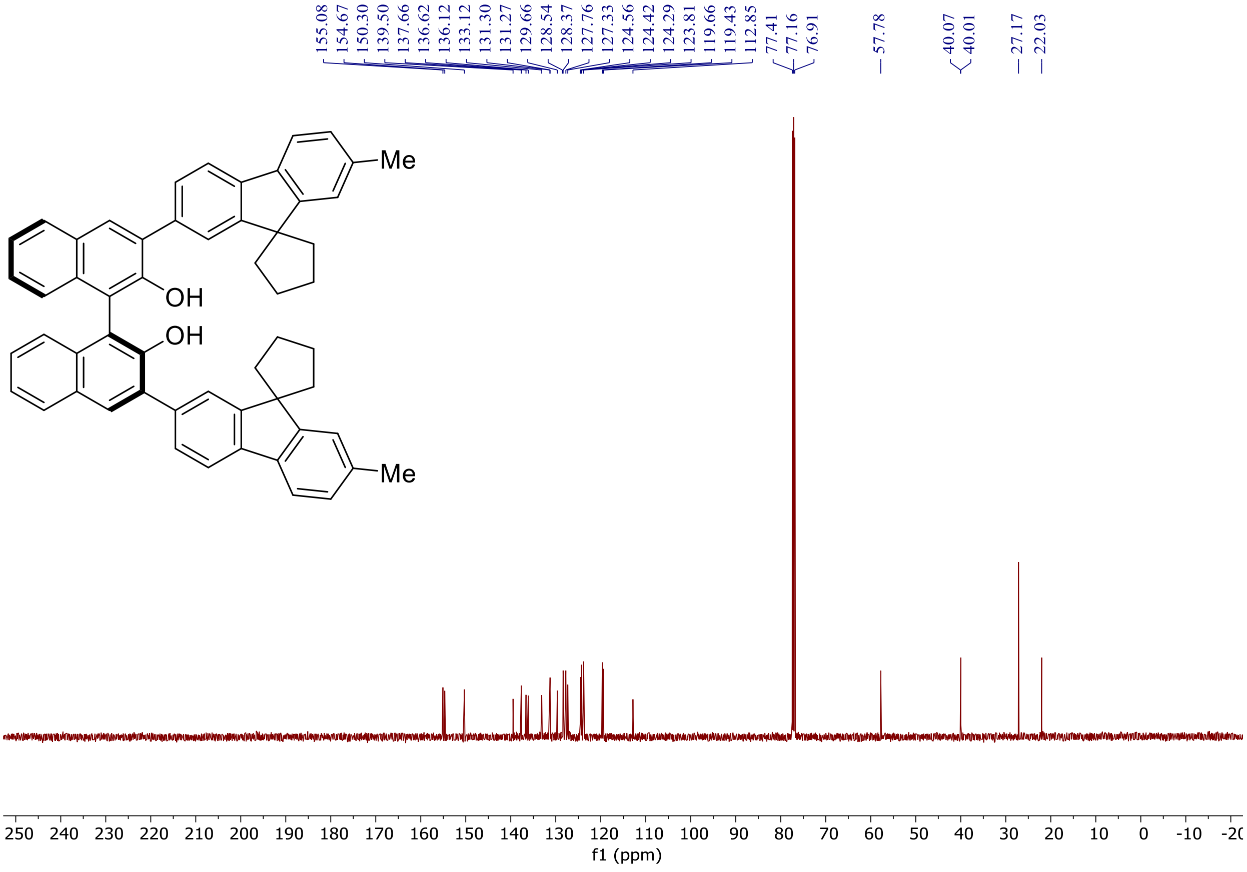

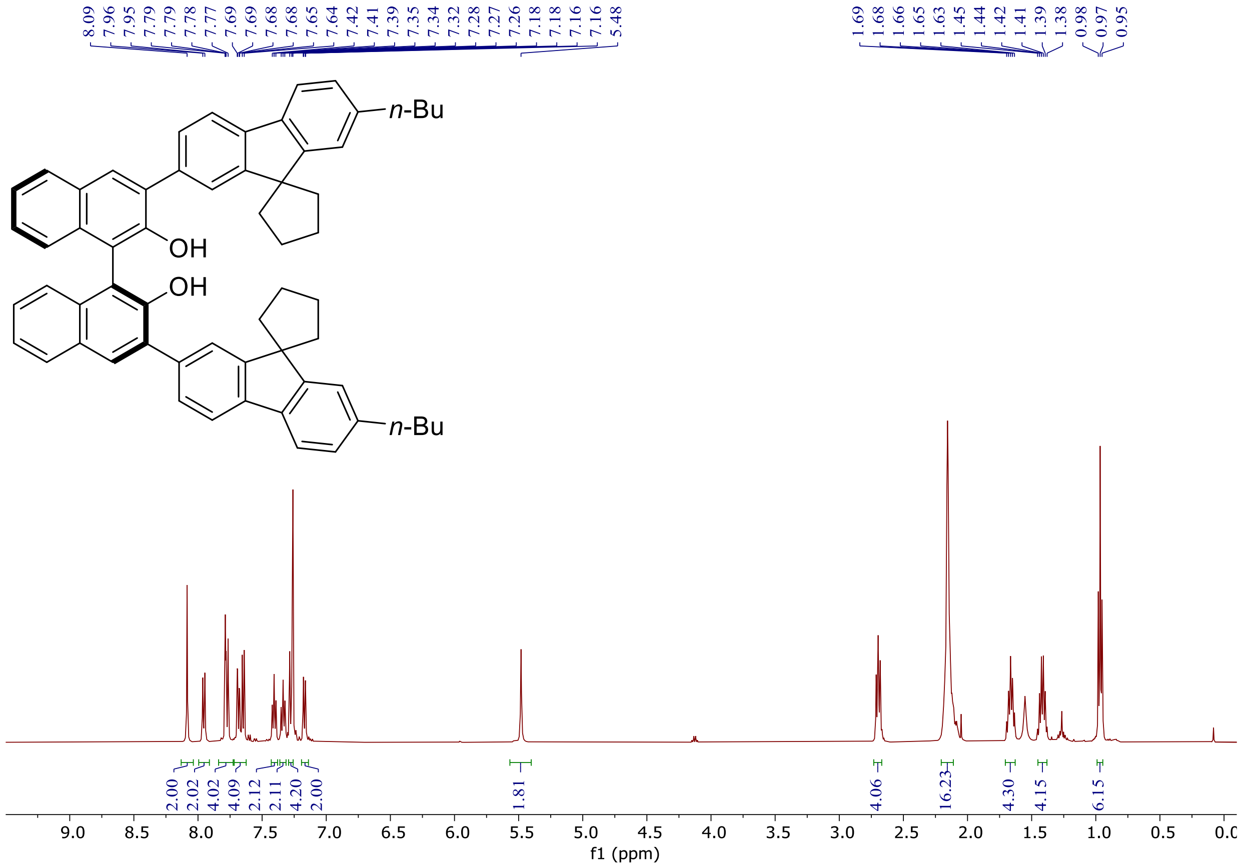
8.09 7.96 7.95 7.79 7.79 7.78 7.77 7.69 7.69 7.68 7.68 7.65 7.64 7.42 7.41 7.39 7.35 7.34 7.32 7.28 7.27 7.26 7.18 7.18 7.16 7.16 5.48
1.69 1.68 1.66 1.65 1.63 1.45 1.44 1.42 1.41 1.39 1.38 0.98 0.97 0.95
n-Bu
OH
OH
n-Bu
2.00 2.02 4.02 4.09 2.12 2.11 4.20 2.00
1.81
4.06
16.23
4.30
4.15
6.15
9.0 8.5 8.0 7.5 7.0 6.5 6.0 5.5 5.0 4.5 4.0 3.5 3.0 2.5 2.0 1.5 1.0 0.5 0.0
f1 (ppm)

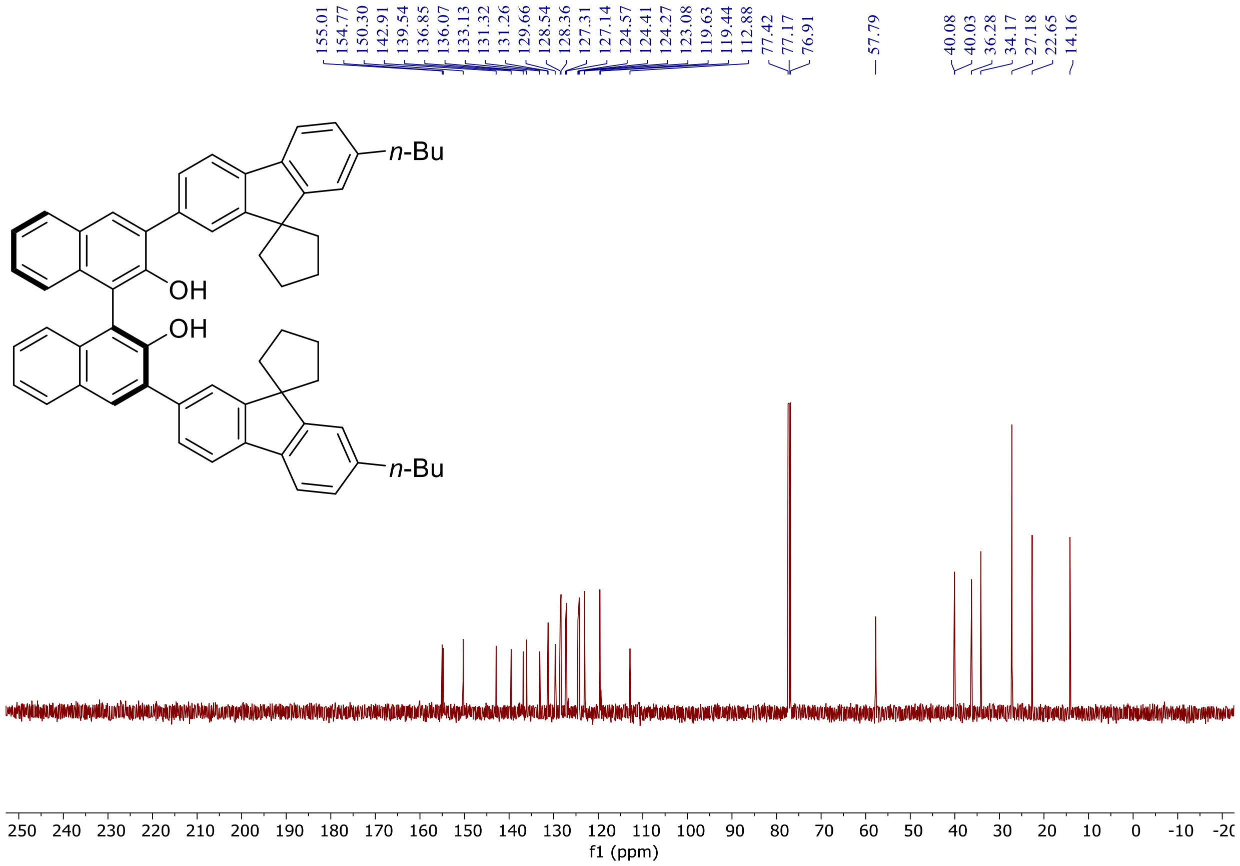
155.01 154.77 150.30 142.91 139.54 136.85 136.07 133.13 131.32 131.26 129.66 128.54 128.36 127.31 127.14 124.57 124.41 124.27 123.08 119.63 119.44 112.88 77.42 77.17 76.91
57.79
40.08 40.03 36.28 34.17 27.18 22.65 14.16
n-Bu
OH
OH
n-Bu
250 240 230 220 210 200 190 180 170 160 150 140 130 120 110 100 90 80 70 60 50 40 30 20 10 0 -10 -20
f1 (ppm)

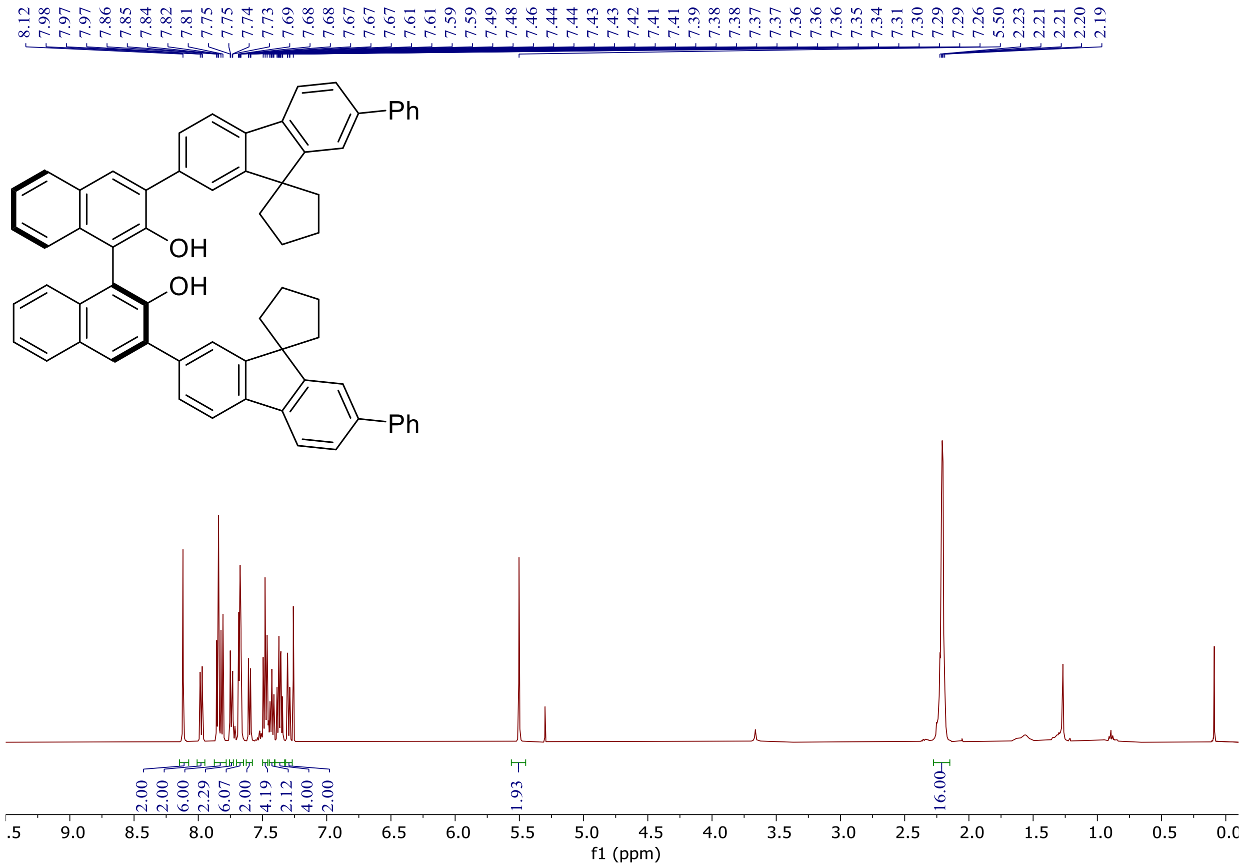
8.12
7.98
7.97
7.97
7.86
7.85
7.84
7.82
7.81
7.75
7.75
7.74
7.73
7.69
7.68
7.68
7.67
7.67
7.67
7.61
7.61
7.59
7.59
7.49
7.48
7.46
7.44
7.44
7.43
7.43
7.42
7.41
7.41
7.39
7.38
7.38
7.37
7.37
7.36
7.36
7.35
7.34
7.31
7.30
7.29
7.29
7.26
5.50
2.23
2.21
2.21
2.20
2.19
Ph
OH
OH
Ph
2.00
2.00
6.00
2.29
6.07
2.00
4.19
2.12
4.00
2.00
1.93
16.00
f1 (ppm)

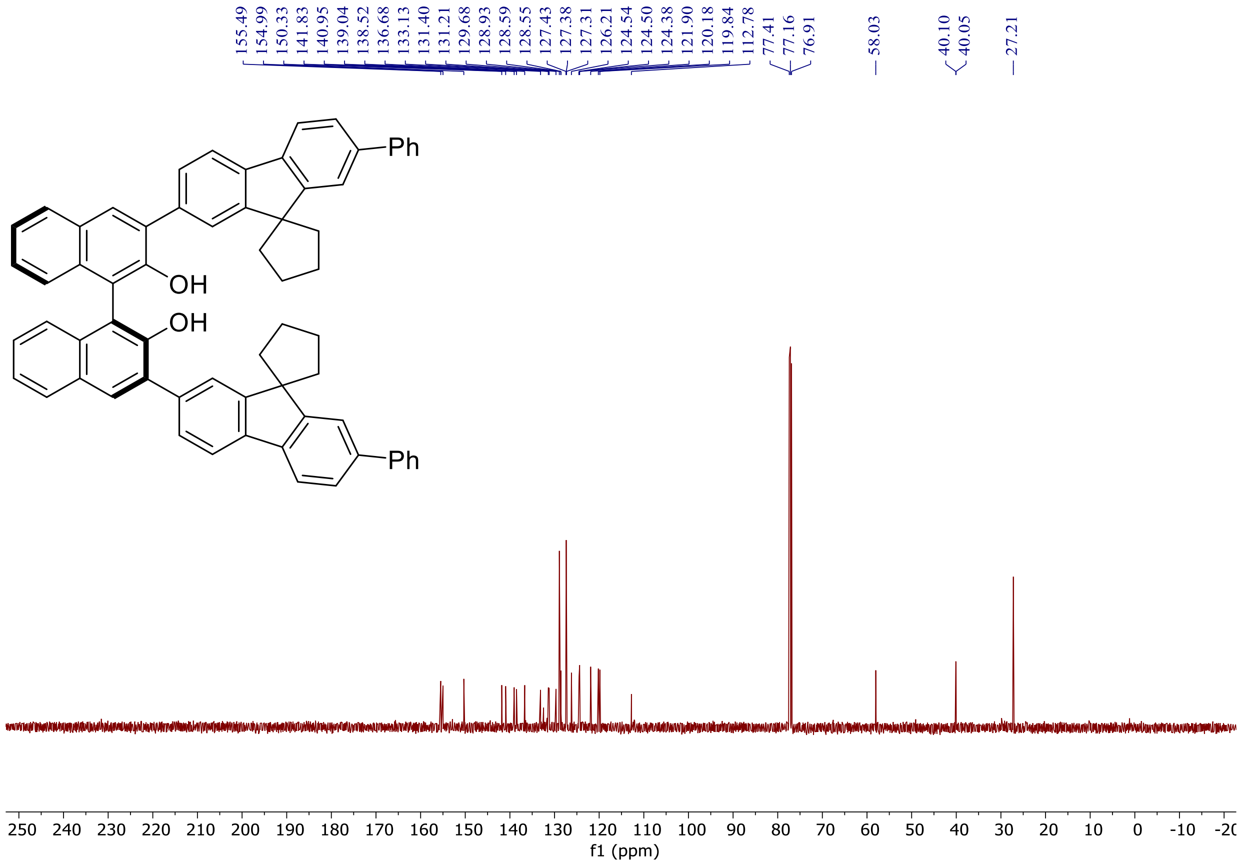
155.49
154.99
150.33
141.83
140.95
139.04
138.52
136.68
133.13
131.40
131.21
129.68
128.93
128.59
128.55
127.43
127.38
127.31
126.21
124.54
124.50
124.38
121.90
120.18
119.84
112.78
77.41
77.16
76.91
58.03
40.10
40.05
27.21
Ph
OH
OH
Ph
f1 (ppm)

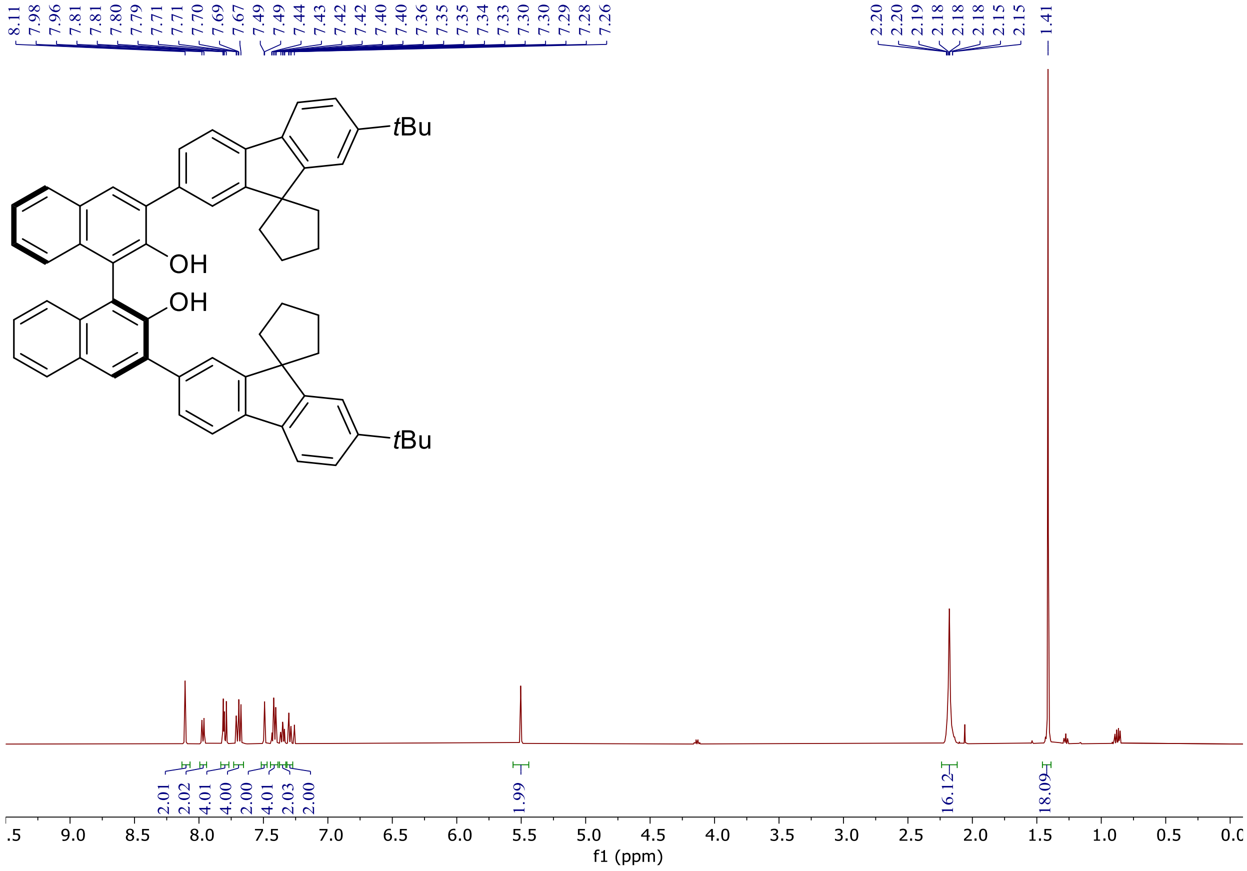
8.11 7.98 7.96 7.81 7.81 7.80 7.79 7.71 7.71 7.70 7.69 7.67 7.49 7.49 7.44 7.43 7.42 7.42 7.40 7.40 7.36 7.35 7.35 7.34 7.33 7.30 7.30 7.29 7.28 7.26
2.20 2.20 2.19 2.18 2.18 2.18 2.15 2.15
1.41
tBu
OH
OH
tBu
2.01 2.02 4.01 4.00 2.00 4.01 2.03 2.00
1.99
16.12
18.09
f1 (ppm)

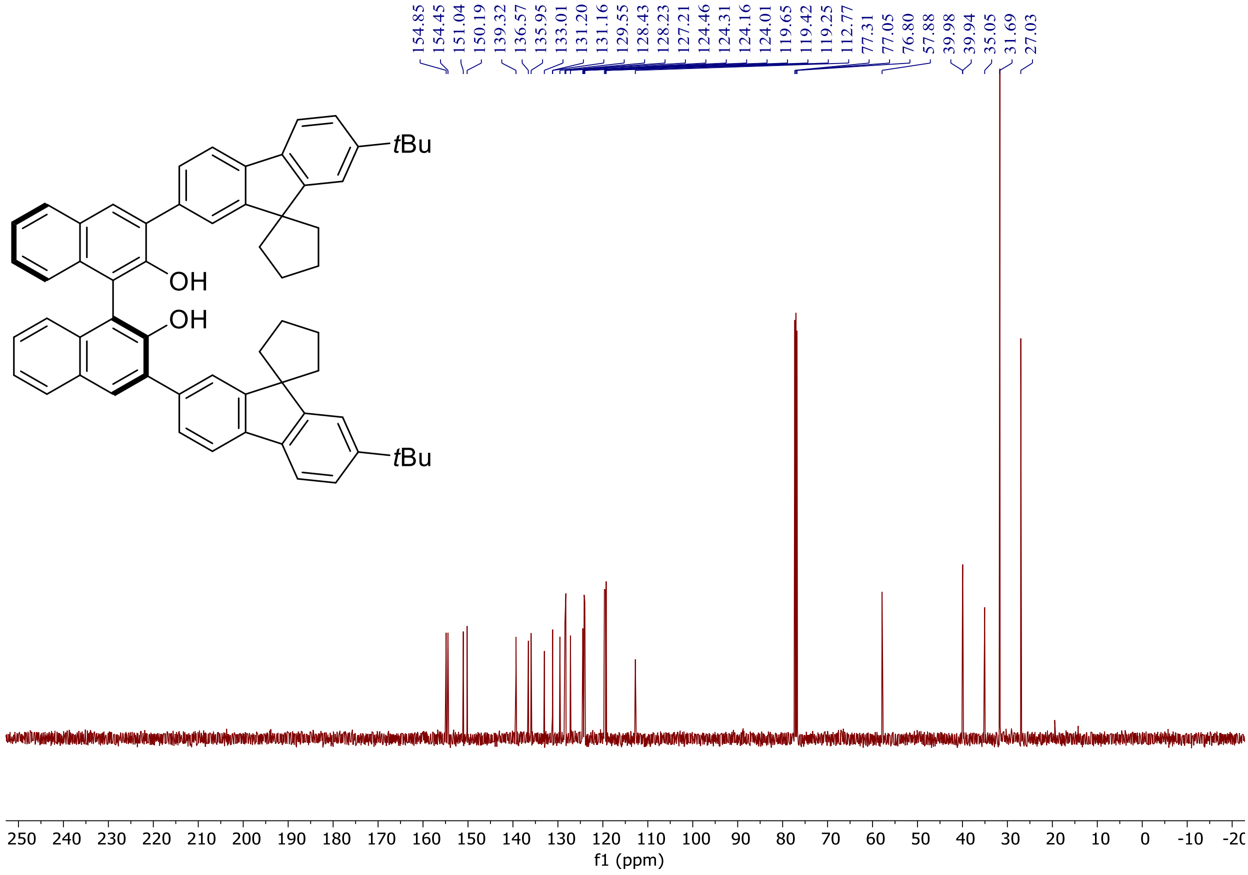
154.85 154.45 151.04 150.19 139.32 136.57 135.95 133.01 131.20 131.16 129.55 128.43 128.23 127.21 124.46 124.31 124.16 124.01 119.65 119.42 119.25 112.77 77.31 77.05 76.80 57.88 39.98 39.94 35.05 31.69 27.03
tBu
OH
OH
tBu
f1 (ppm)

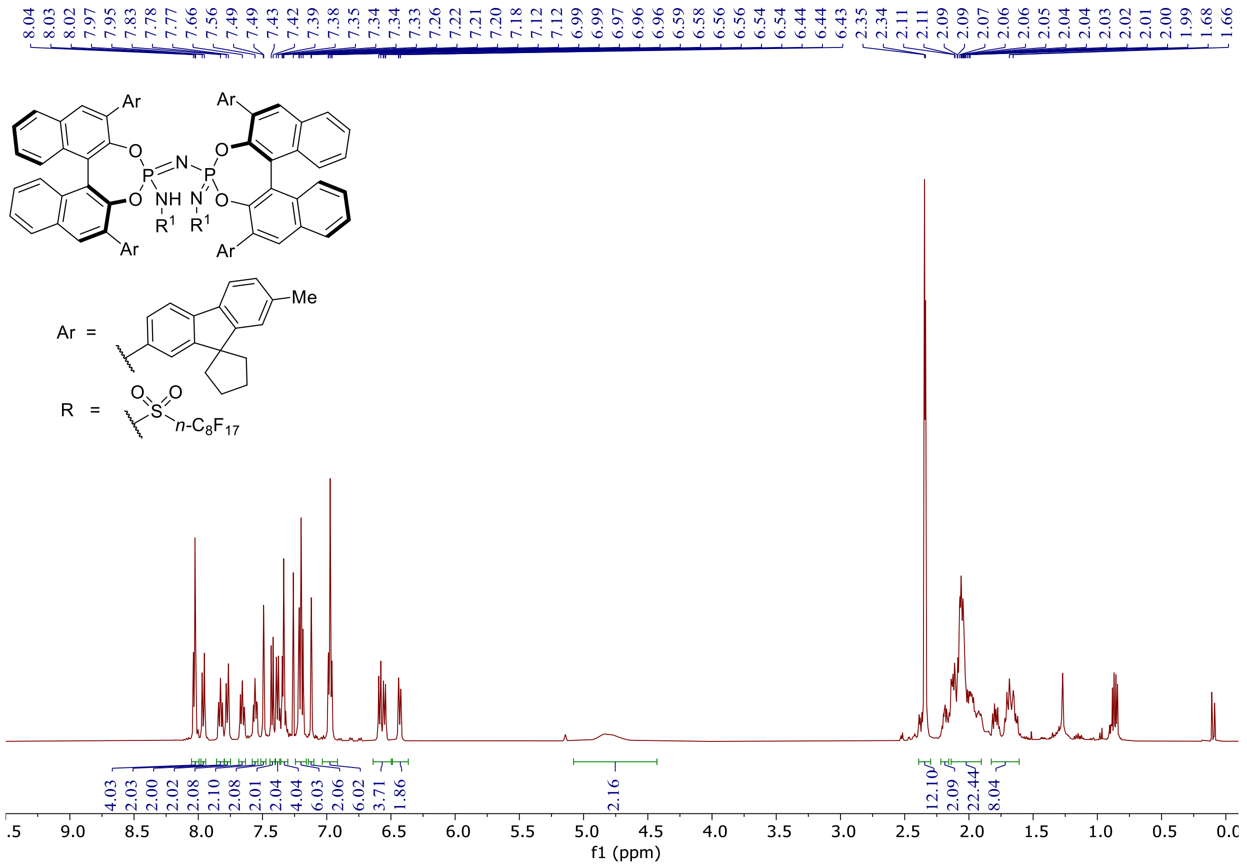
8.04 8.03 8.02 7.97 7.95 7.83 7.78 7.77 7.66 7.56 7.49 7.49 7.43 7.42 7.39 7.38 7.35 7.34 7.34 7.33 7.26 7.22 7.21 7.20 7.18 7.12 7.12 6.99 6.99 6.97 6.96 6.96 6.59 6.58 6.56 6.56 6.54 6.54 6.44 6.43 2.35 2.34 2.11 2.11 2.09 2.09 2.07 2.06 2.06 2.05 2.04 2.04 2.03 2.02 2.01 2.00 1.99 1.68 1.66
Ar
O
P
N
NH
R1
Ar =
Me
R =
O
S
n-C_8F_{17}
4.03 2.03 2.00 2.02 2.08 2.10 2.08 2.01 2.04 4.04 6.03 2.06 6.02 3.71 1.86
2.16
12.10 2.09 22.44 8.04
.5 9.0 8.5 8.0 7.5 7.0 6.5 6.0 5.5 5.0 4.5 4.0 3.5 3.0 2.5 2.0 1.5 1.0 0.5 0.0
f1 (ppm)

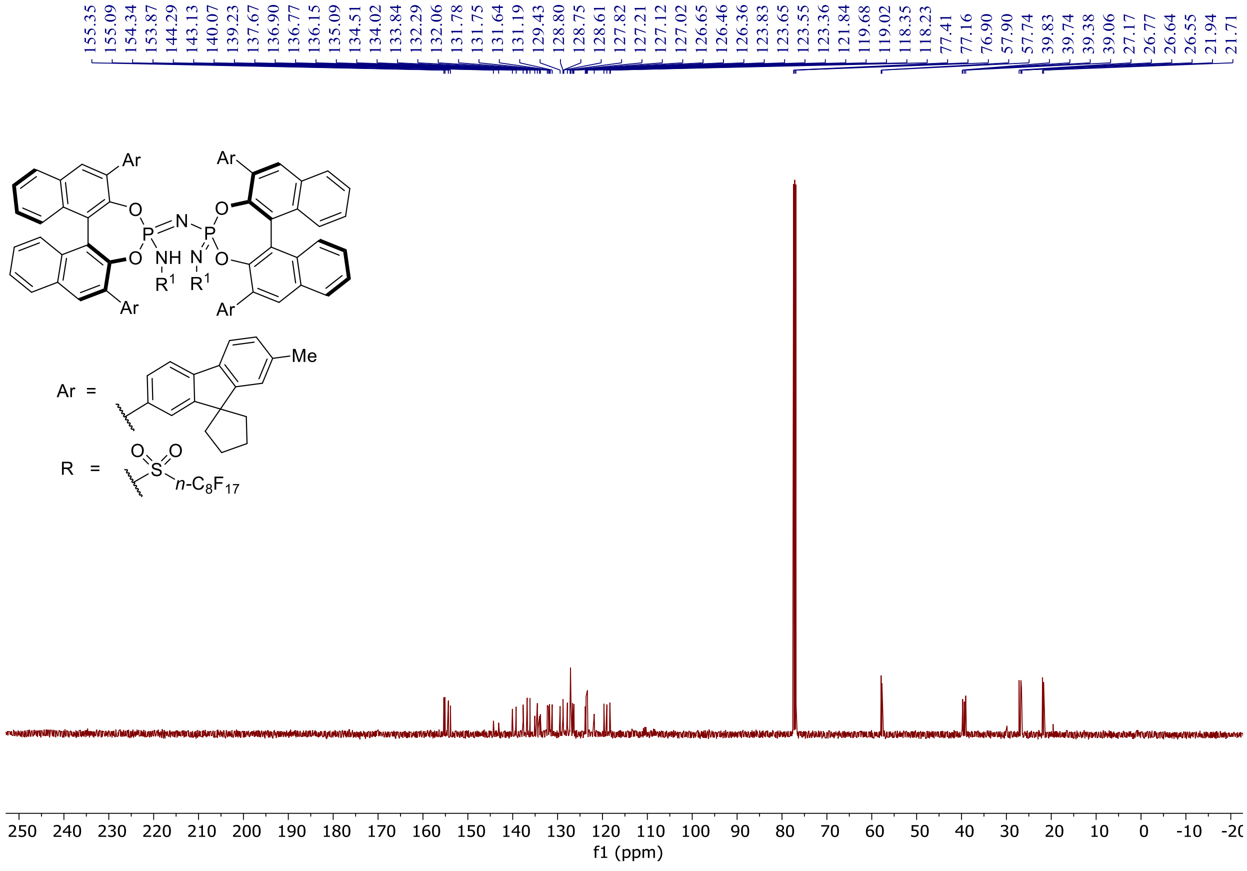
155.35 155.09 154.34 153.87 144.29 143.13 140.07 139.23 137.67 136.90 136.77 136.15 135.09 134.51 134.02 133.84 132.29 132.06 131.78 131.75 131.64 131.19 129.43 128.80 128.75 128.61 127.82 127.21 127.12 127.02 126.65 126.46 126.36 123.83 123.65 123.55 123.36 121.84 119.68 119.02 118.35 118.23 77.41 77.16 76.90 57.90 57.74 39.83 39.74 39.38 39.06 27.17 26.77 26.64 26.55 21.94 21.71
Ar
O
P
N
NH
R1
Ar =
Me
R =
O
S
n-C_8F_{17}
250 240 230 220 210 200 190 180 170 160 150 140 130 120 110 100 90 80 70 60 50 40 30 20 10 0 -10 -20
f1 (ppm)

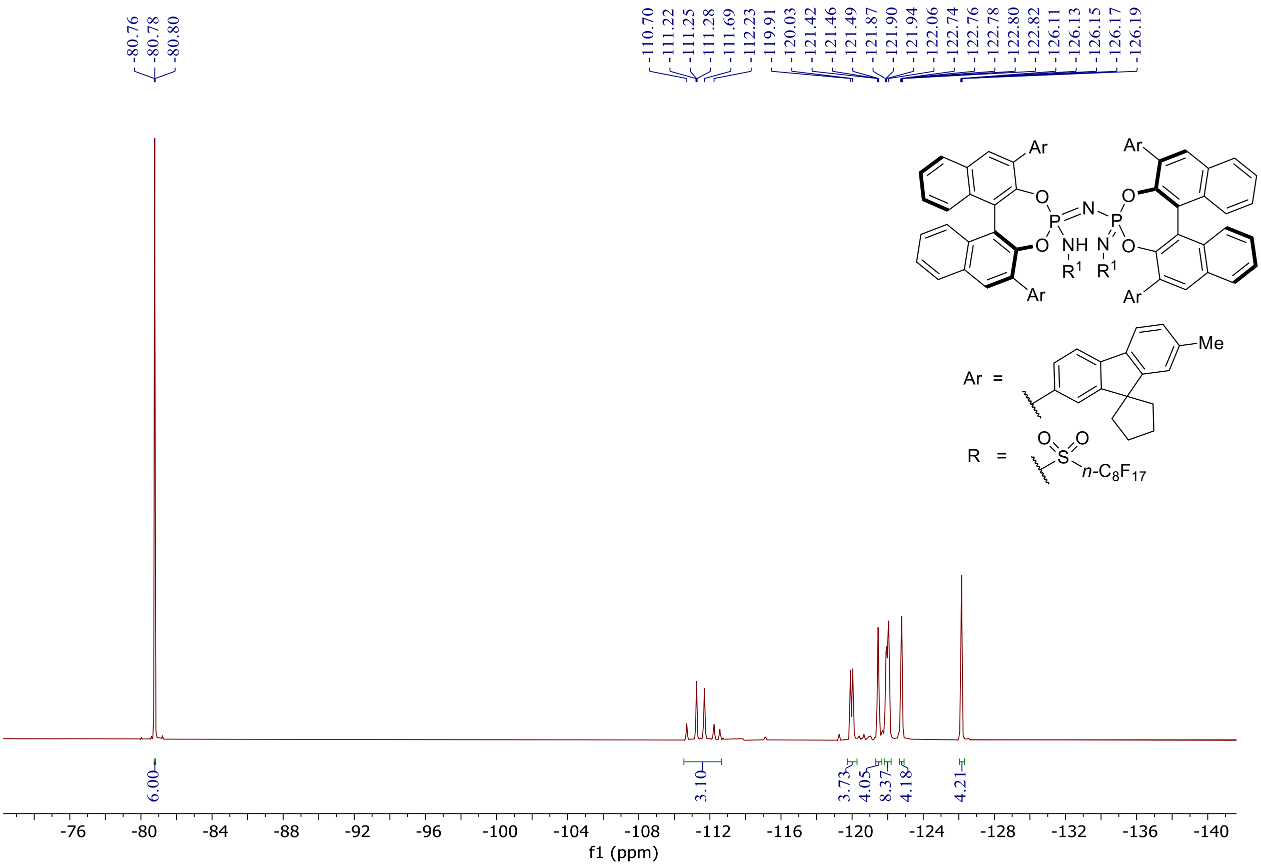
-80.76
-80.78
-80.80
-110.70
-111.22
-111.25
-111.28
-111.69
-112.23
-119.91
-120.03
-121.42
-121.46
-121.49
-121.87
-121.90
-121.94
-122.06
-122.74
-122.76
-122.78
-122.80
-122.82
-126.11
-126.13
-126.15
-126.17
-126.19
Ar
O
P
N
NH
R^1
Ar =
Me
R =
n-C_8F_{17}
6.00
3.10
3.73
4.05
8.37
4.18
4.21
f1 (ppm)

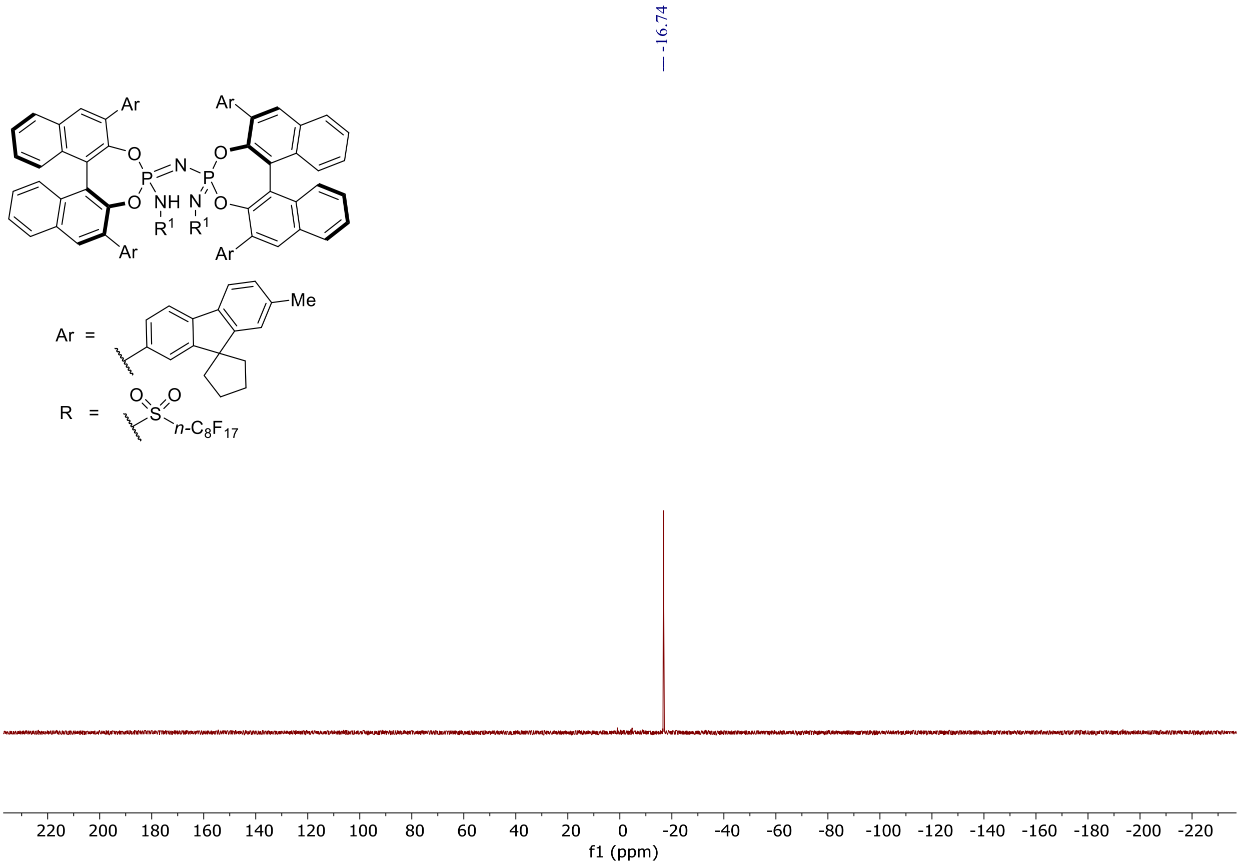
-16.74
Ar
O
P
N
NH
R^1
Ar =
Me
R =
n-C_8F_{17}
f1 (ppm)

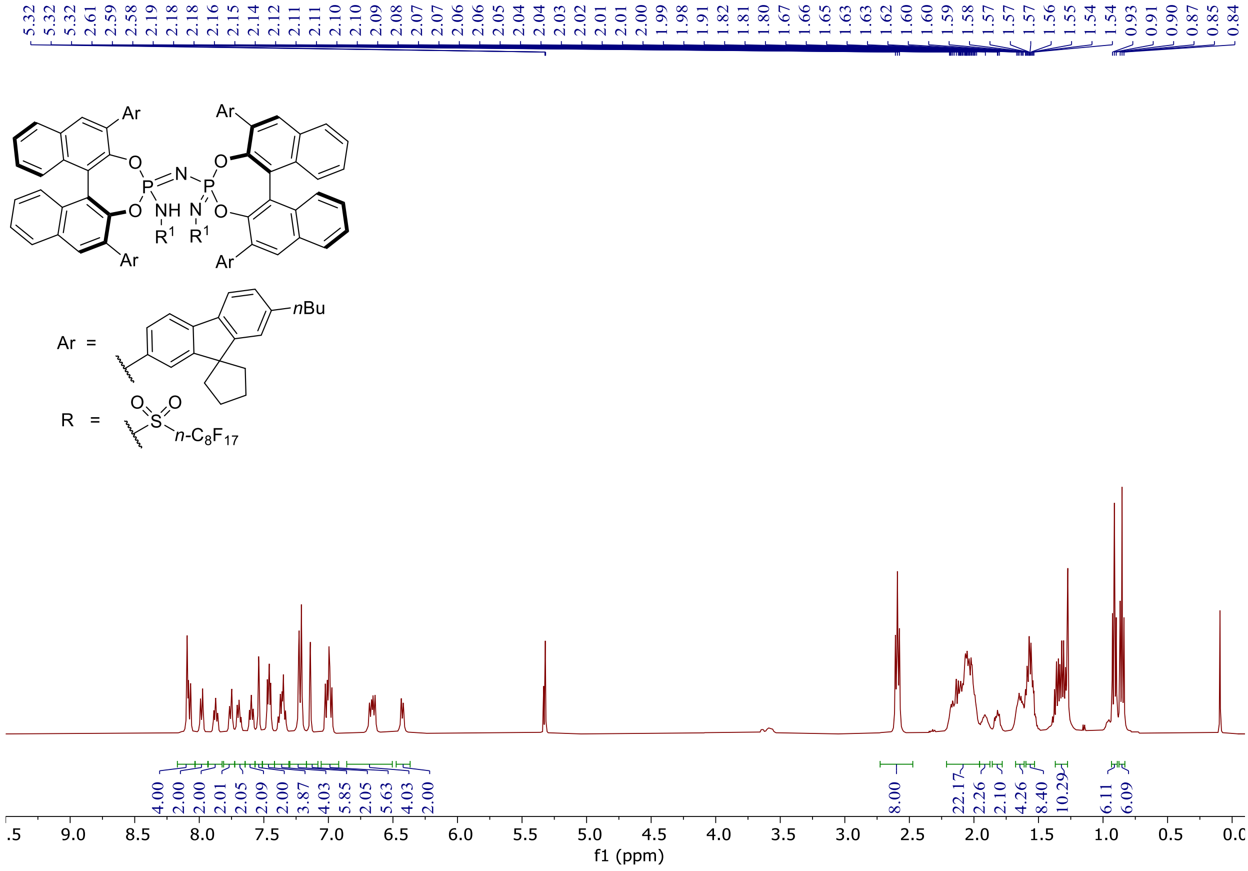

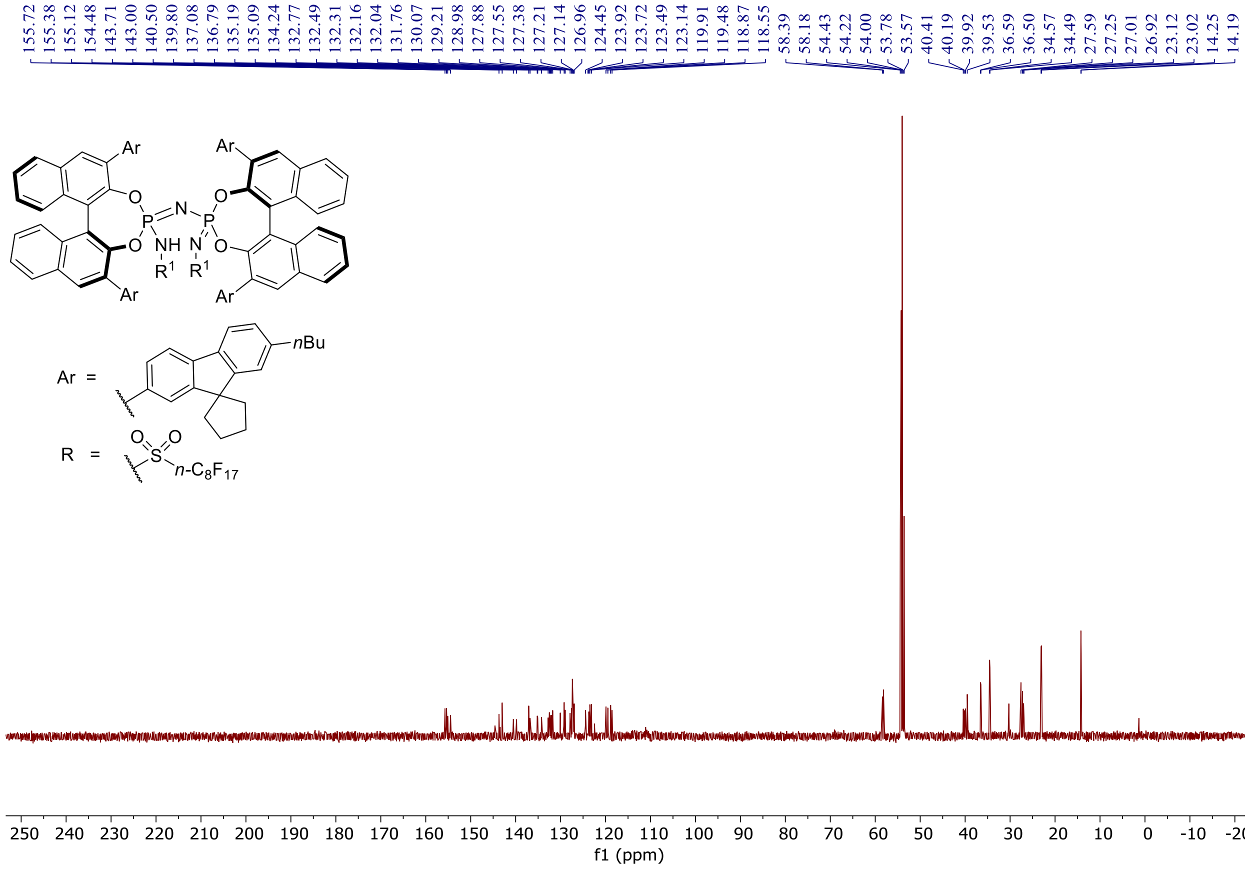

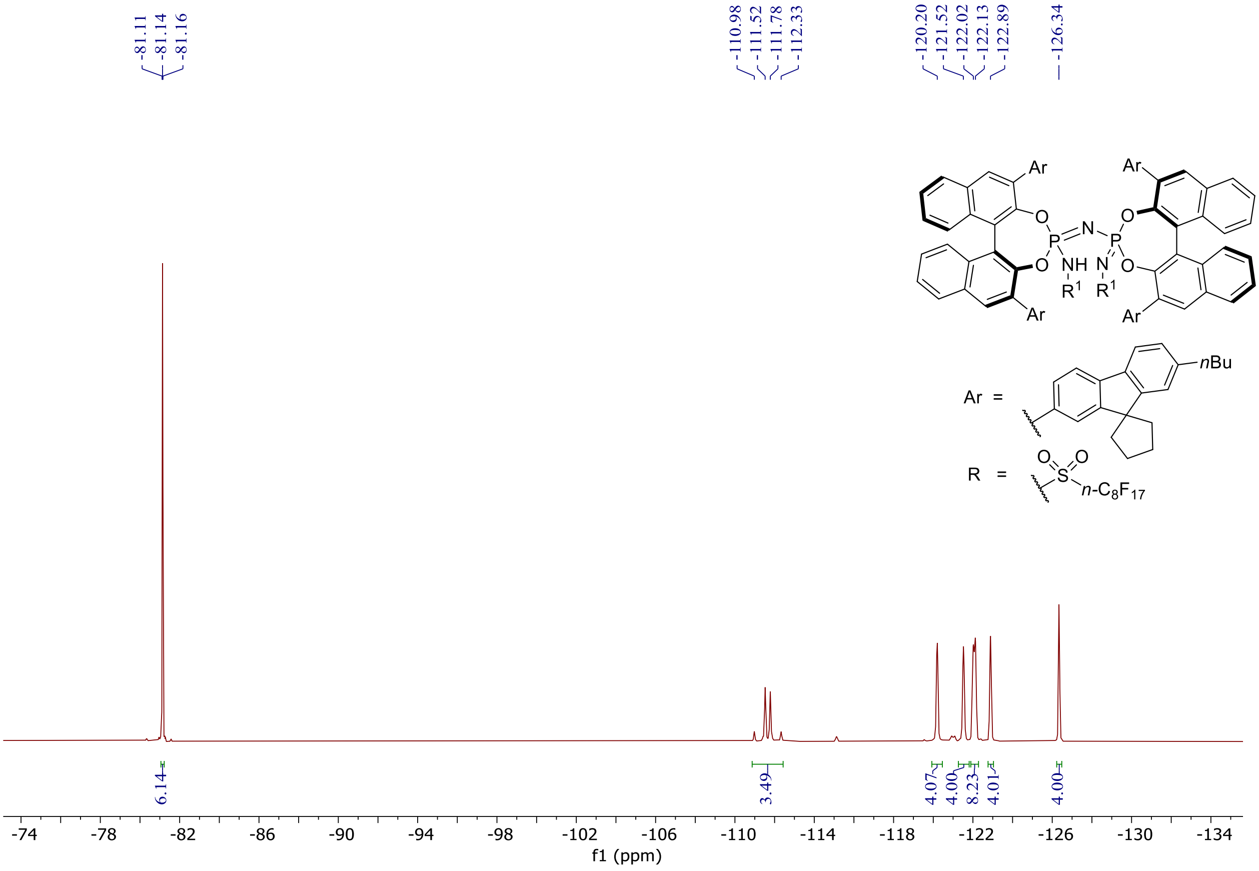
-81.11
-81.14
-81.16
-110.98
-111.52
-111.78
-112.33
-120.20
-121.52
-122.02
-122.13
-122.89
-126.34
Ar
O
P
N
NH
R^1
Ar =
nBu
R =
O
S
O
n-C_8F_{17}
6.14
3.49
4.07
4.00
8.23
4.01
4.00
-74 -78 -82 -86 -90 -94 -98 -102 -106 -110 -114 -118 -122 -126 -130 -134
f1 (ppm)

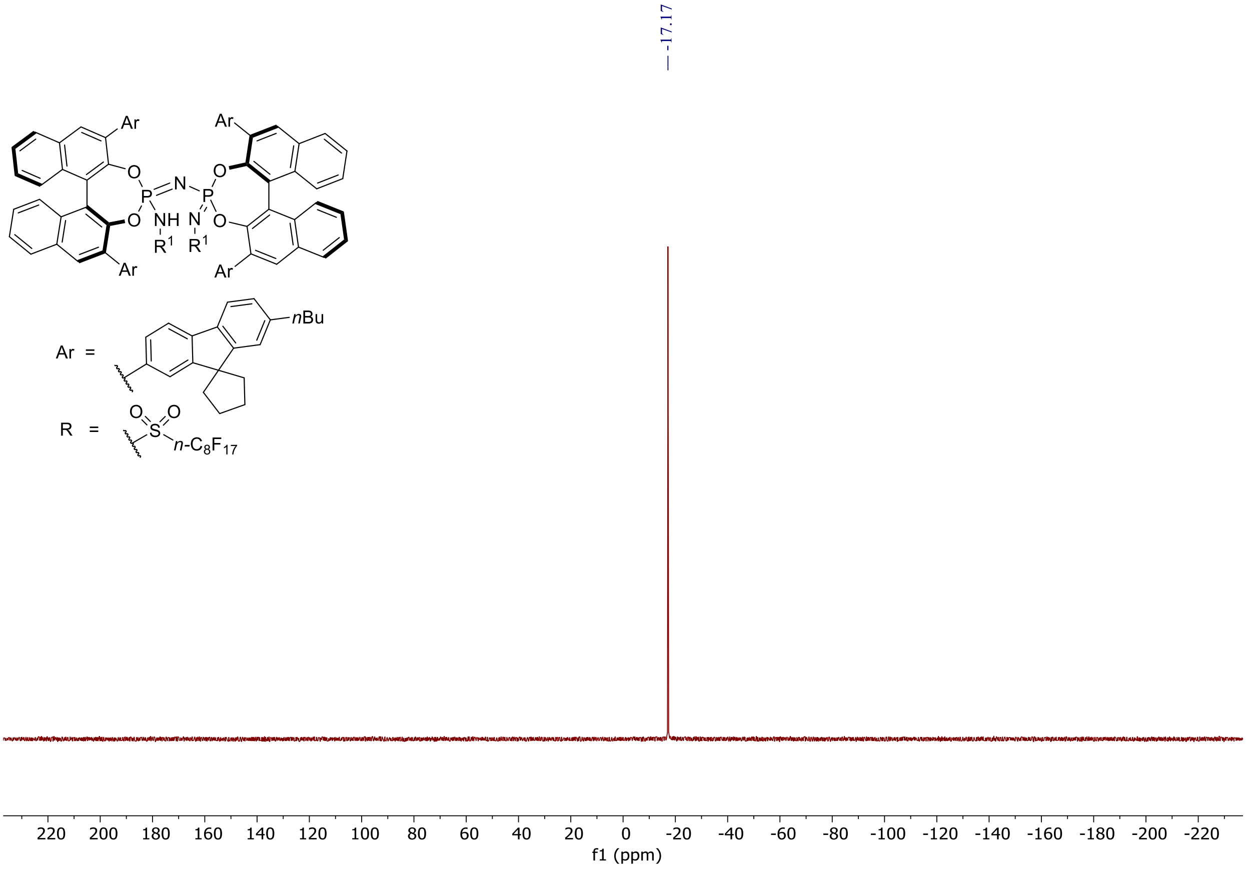
-17.17
Ar
O
P
N
NH
R^1
Ar =
nBu
R =
O
S
O
n-C_8F_{17}
220 200 180 160 140 120 100 80 60 40 20 0 -20 -40 -60 -80 -100 -120 -140 -160 -180 -200 -220
f1 (ppm)

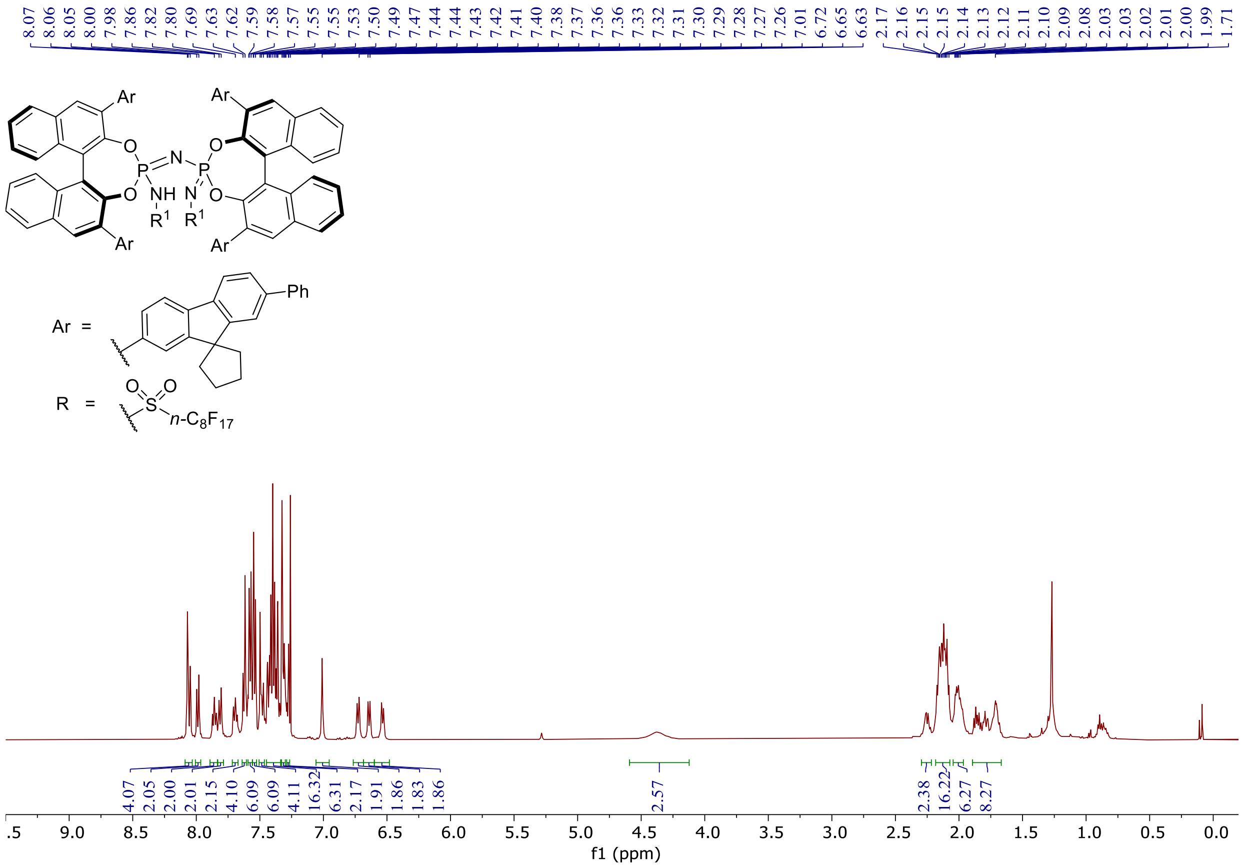

8.07 8.06 8.05 8.00 7.98 7.86 7.82 7.80 7.69 7.63 7.62 7.59 7.58 7.57 7.55 7.55 7.53 7.50 7.49 7.47 7.44 7.44 7.43 7.42 7.41 7.40 7.38 7.37 7.36 7.36 7.33 7.32 7.31 7.30 7.29 7.28 7.27 7.26 7.01 6.72 6.65 6.63 2.17 2.16 2.15 2.15 2.14 2.13 2.12 2.11 2.10 2.09 2.08 2.03 2.03 2.02 2.01 2.00 1.99 1.71
Ar
O
P
N
NH
R1
Ar =
Ph
R =
O
S
n-C8F17
4.07 2.05 2.00 2.01 2.15 4.10 6.09 6.09 4.11 16.32 6.31 2.17 1.91 1.86 1.83 1.86
2.57
2.38 16.22 6.27 8.27
.5 9.0 8.5 8.0 7.5 7.0 6.5 6.0 5.5 5.0 4.5 4.0 3.5 3.0 2.5 2.0 1.5 1.0 0.5 0.0
f1 (ppm)

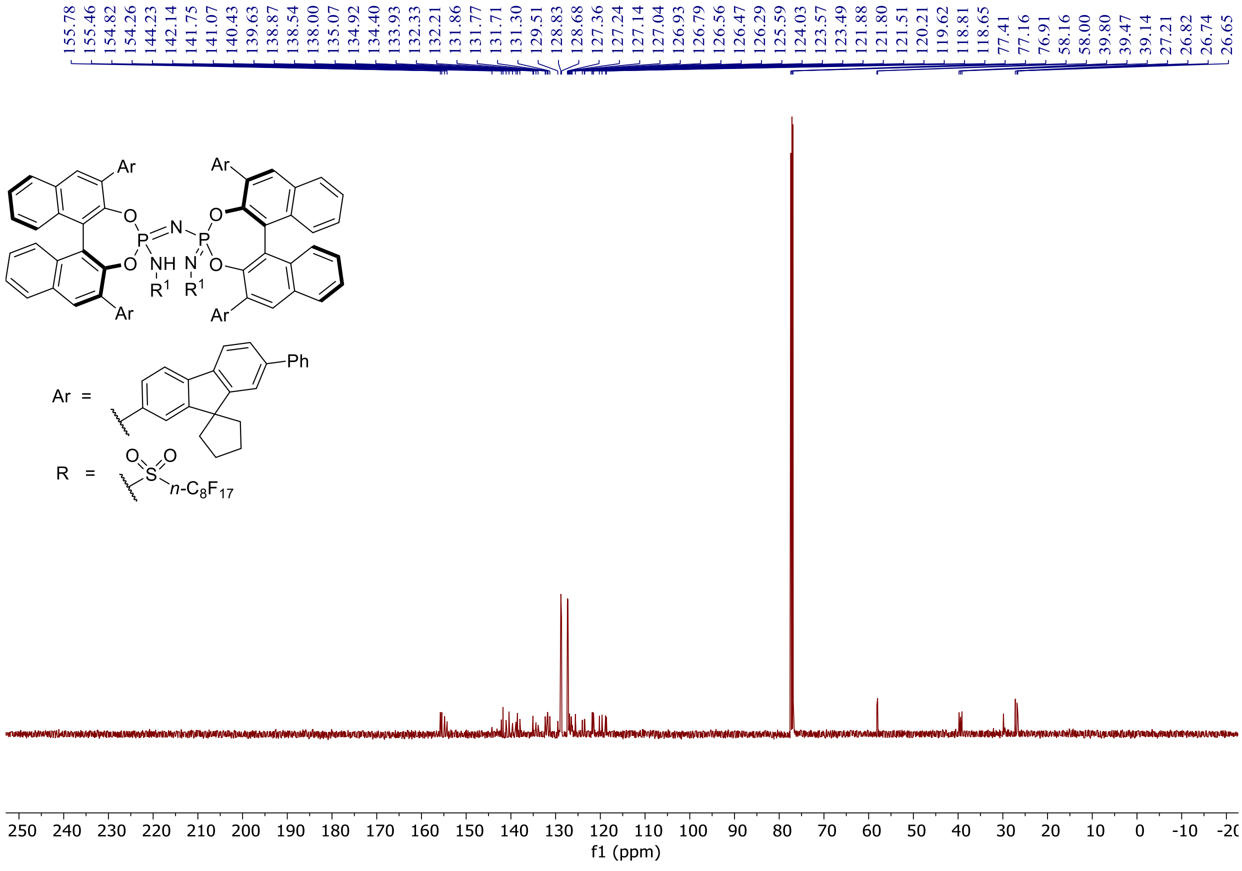

155.78 155.46 154.82 154.26 144.23 142.14 141.75 141.07 140.43 139.63 138.87 138.54 138.00 135.07 134.92 134.40 133.93 132.33 132.21 131.86 131.77 131.71 131.30 129.51 128.83 128.68 127.36 127.24 127.14 127.04 126.93 126.79 126.56 126.47 126.29 125.59 124.03 123.57 123.49 121.88 121.80 121.51 120.21 119.62 118.81 118.65 77.41 77.16 76.91 58.16 58.00 39.80 39.47 39.14 27.21 26.82 26.74 26.65
Ar
O
P
N
NH
R1
Ar =
Ph
R =
O
S
n-C8F17
250 240 230 220 210 200 190 180 170 160 150 140 130 120 110 100 90 80 70 60 50 40 30 20 10 0 -10 -20
f1 (ppm)

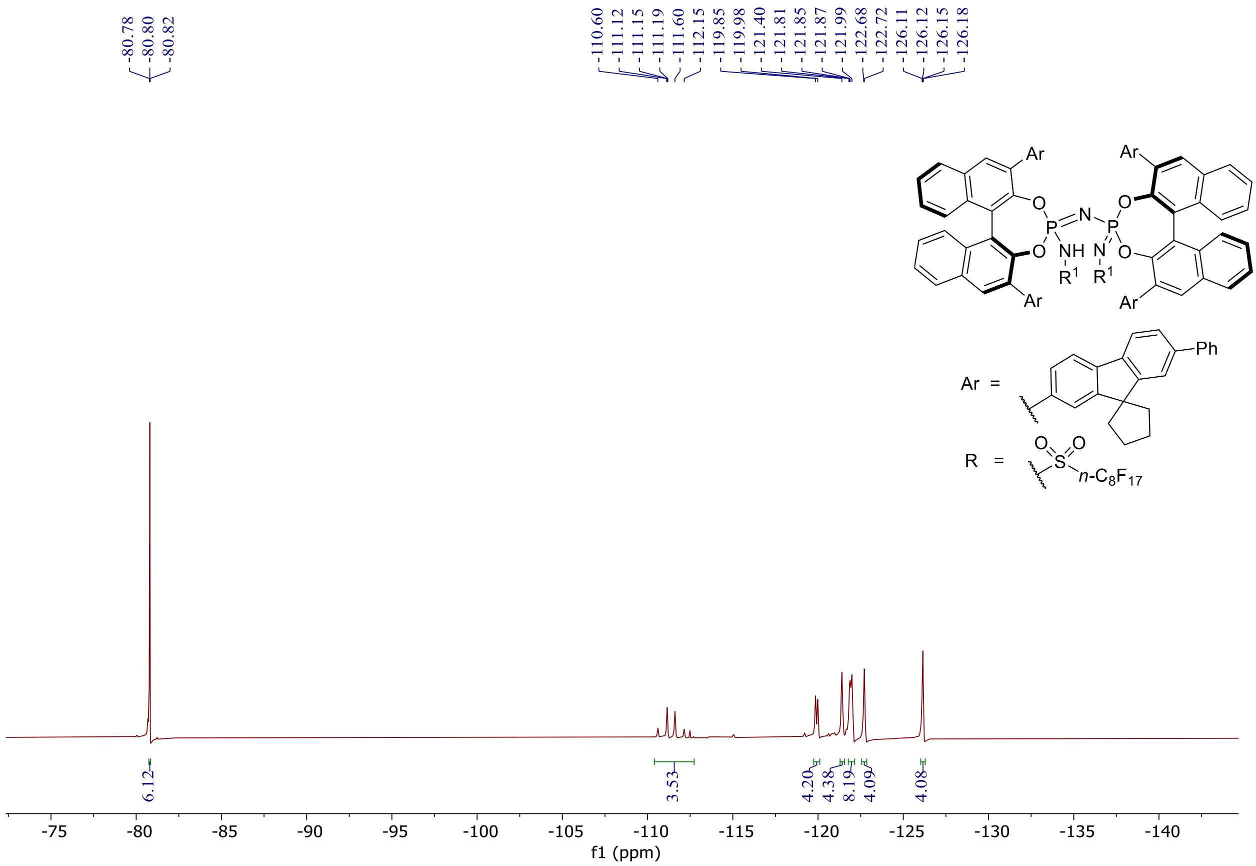
-80.78
-80.80
-80.82
-110.60
-111.12
-111.15
-111.19
-111.60
-112.15
-119.85
-119.98
-121.40
-121.81
-121.85
-121.87
-121.99
-122.68
-122.72
-126.11
-126.12
-126.15
-126.18
Ar
O
P
N
NH
O
R1
Ar =
Ph
R =
n-C8F17
6.12
3.53
4.20
4.38
8.19
4.09
4.08
-75 -80 -85 -90 -95 -100 -105 -110 -115 -120 -125 -130 -135 -140
f1 (ppm)

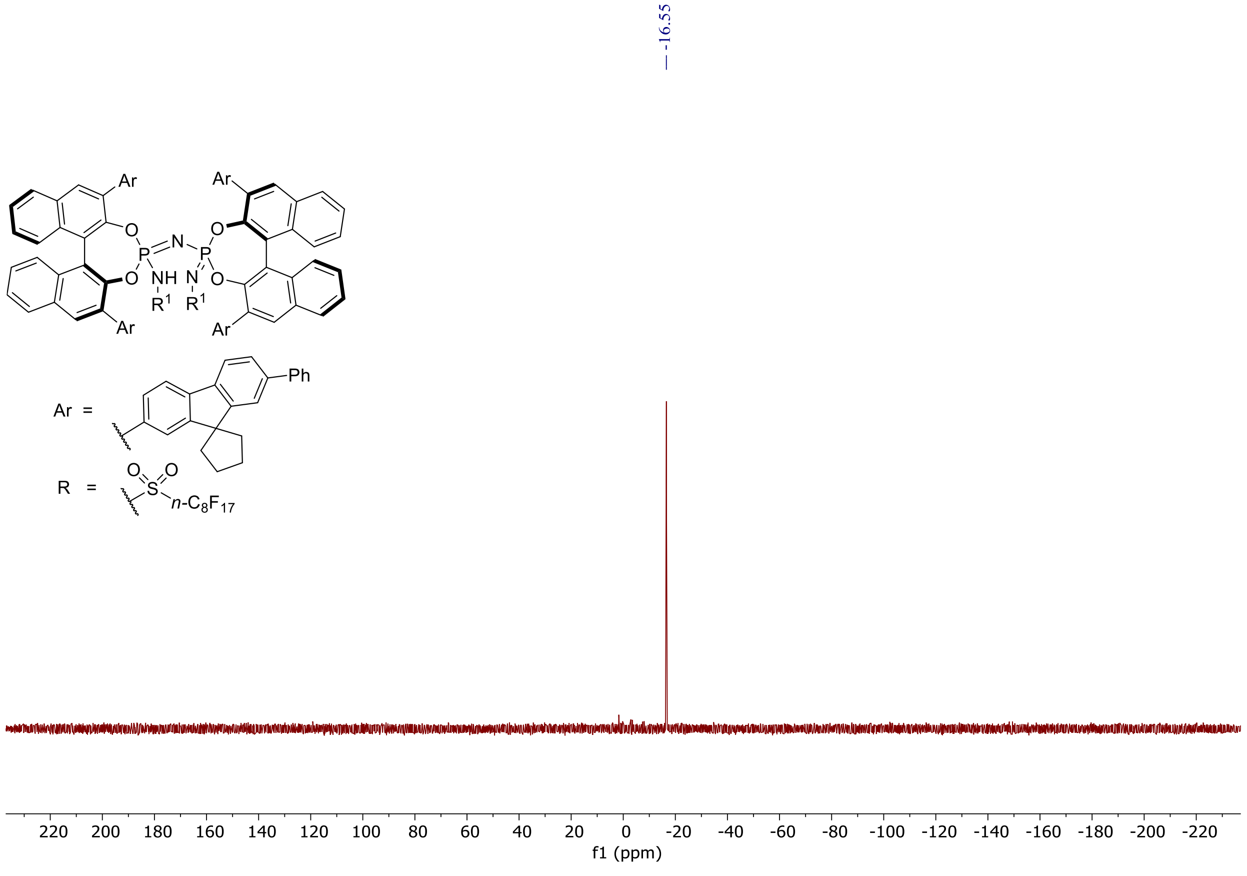
-16.55
Ar
O
P
N
NH
R1
Ar =
Ph
R =
n-C8F17
220 200 180 160 140 120 100 80 60 40 20 0 -20 -40 -60 -80 -100 -120 -140 -160 -180 -200 -220
f1 (ppm)

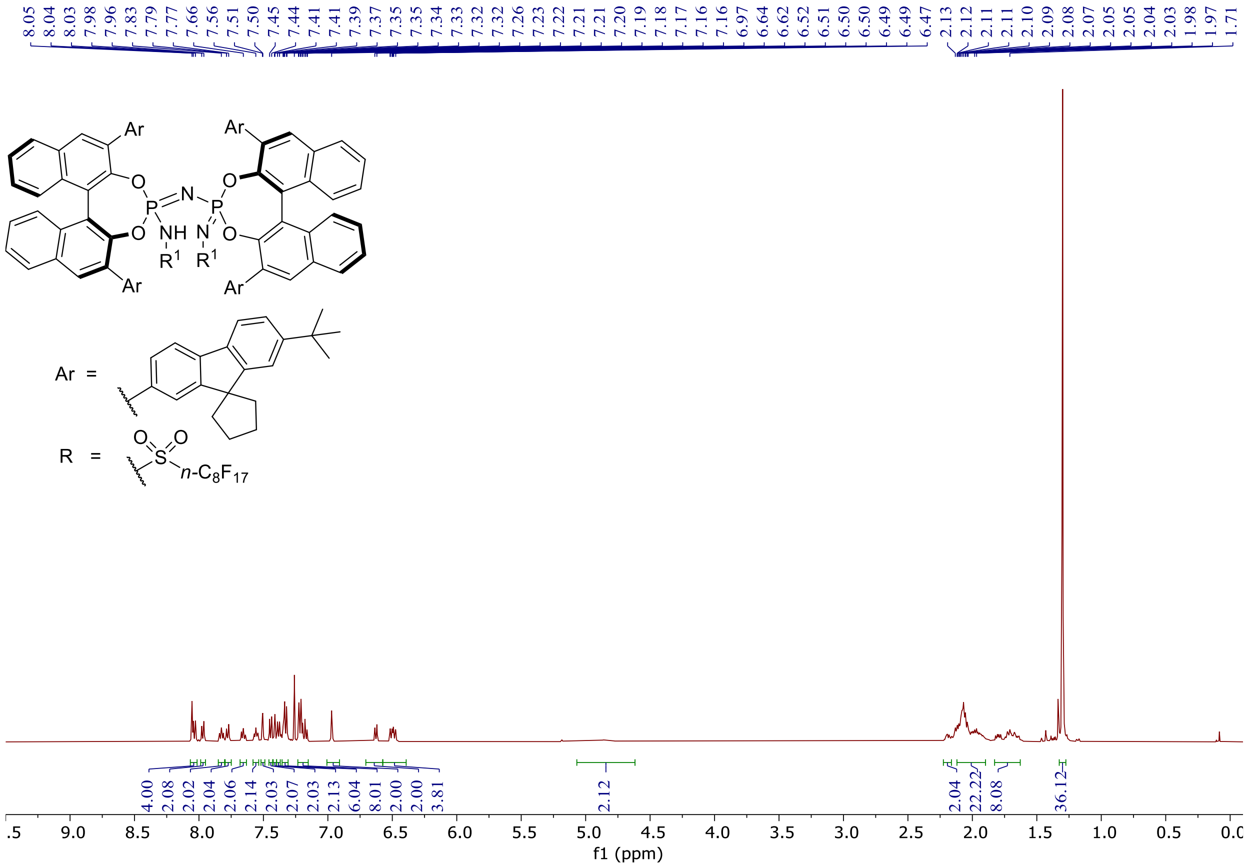

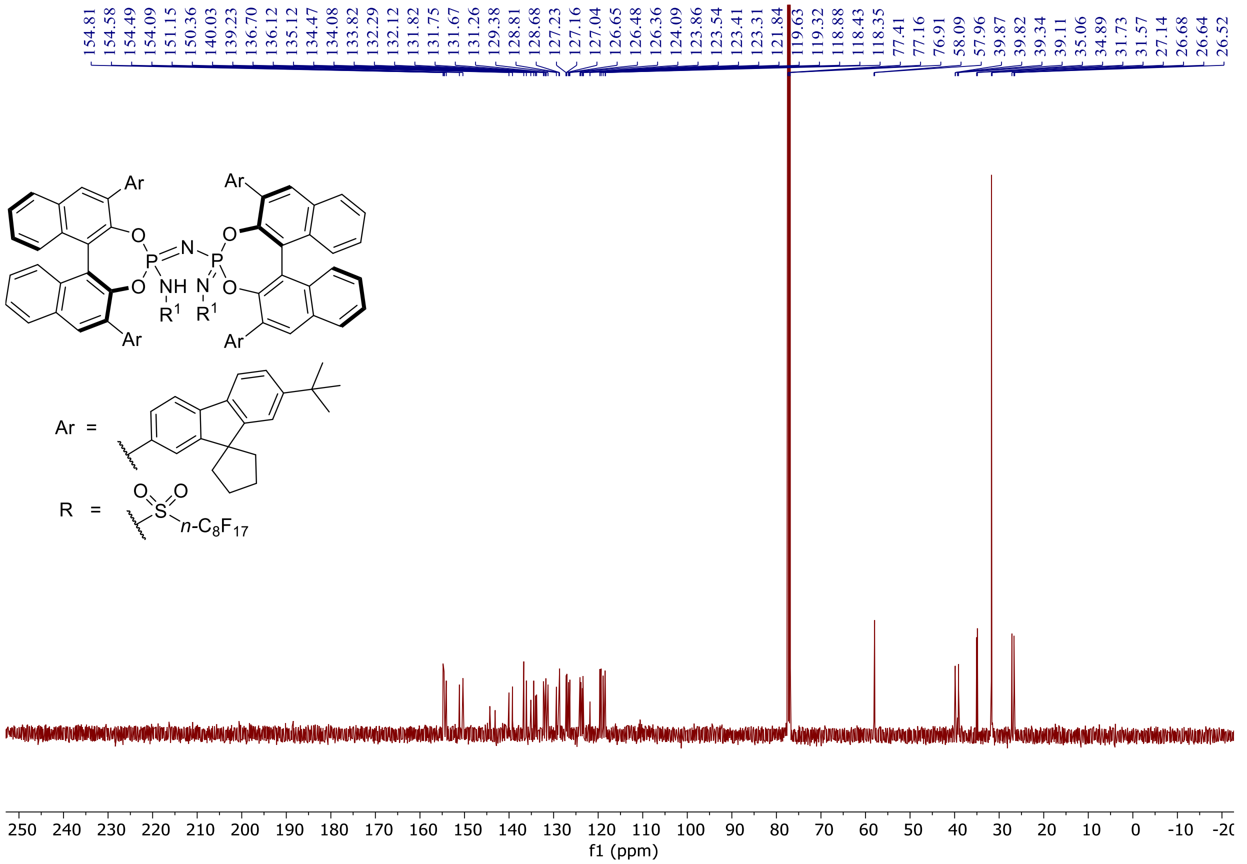

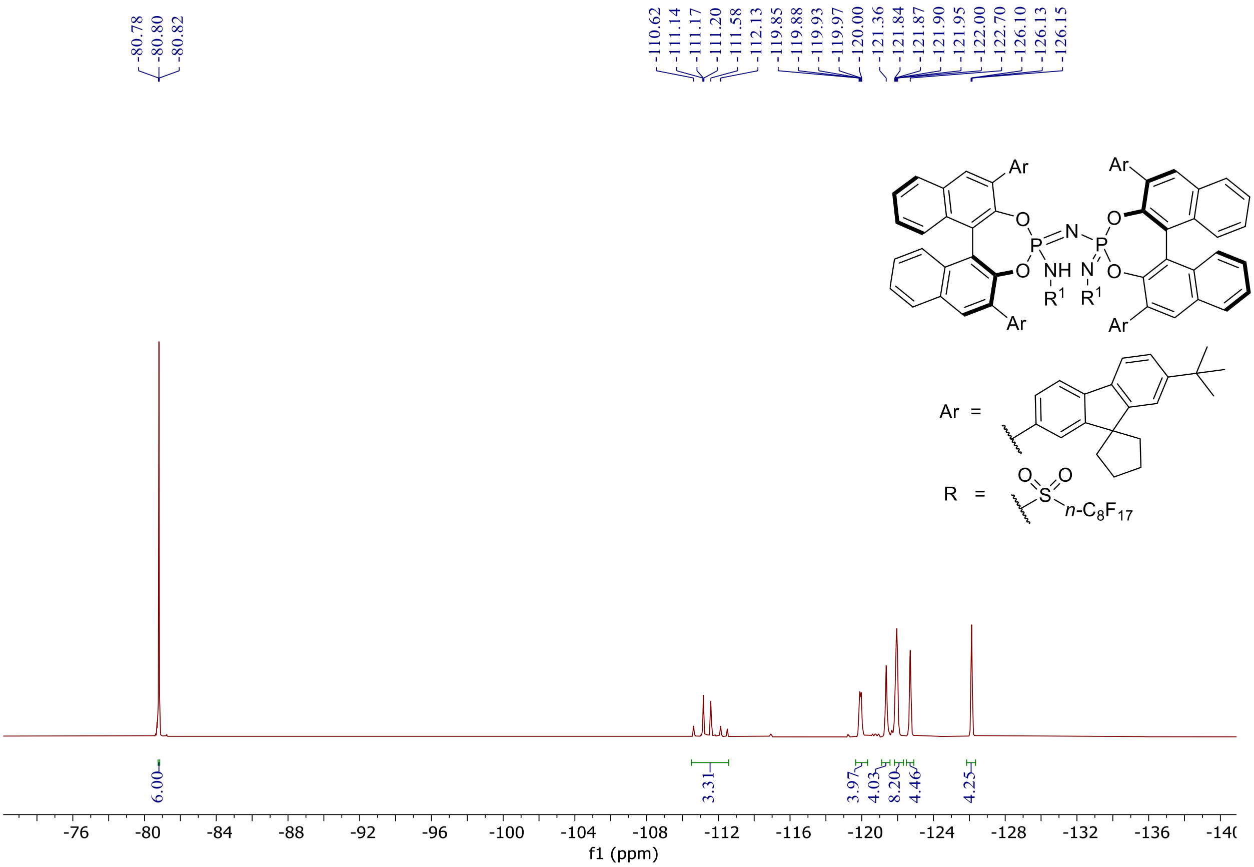
-80.78
-80.80
-80.82
-110.62
-111.14
-111.17
-111.20
-111.58
-112.13
-119.85
-119.88
-119.93
-119.97
-120.00
-121.36
-121.84
-121.87
-121.90
-121.95
-122.00
-122.70
-126.10
-126.13
-126.15
Ar
O
P
N
NH
R1
Ar =
R =
n-C8F17
6.00
3.31
3.97
4.03
8.20
4.46
4.25
-76
-80
-84
-88
-92
-96
-100
-104
-108
-112
-116
-120
-124
-128
-132
-136
f1 (ppm)

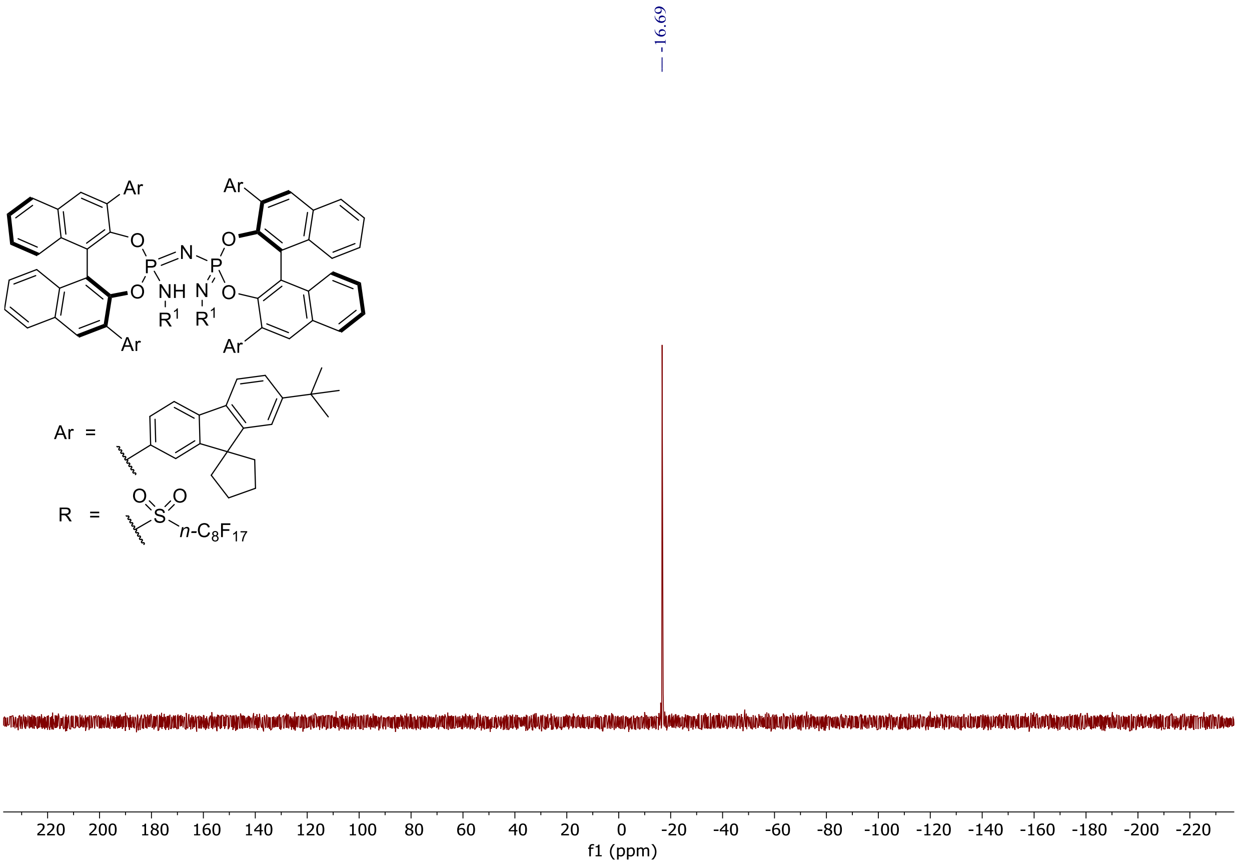
-16.69
Ar
O
P
N
NH
R1
Ar =
R =
n-C8F17
220
200
180
160
140
120
100
80
60
40
20
0
-20
-40
-60
-80
-100
-120
-140
-160
-180
-200
-220
f1 (ppm)

11. Computational Studies

11. 1. Method

All calculations were carried out with a development version of the ORCA suite of programs base on version 4.2.[1] Molecular geometries were optimized in the gas-phase using the PBE functional[2] in conjunction with the D3 version of Grimme's dispersion correction with Becke-Johnson damping function,[3] using the resolution of identity approximation. The def2-SVP basis set was used for all atoms with matching auxiliary basis.[4] In order to gain a qualitative understanding of the stereoinduction, a simplified version of catalyst **3h** has been used, in which the *tert*-butyl group of the fluorenyl substituent has been removed and the *n*-C_8H_{17} sulfonamide substituent has been replaced with a CF_2H group. An exhaustive manual conformational search has been performed on possible catalyst substrate orientations that are subsequently refined by running Single-point energies (gas phase) at the M06-2X/def2-TZVP[5] level of theory. Molecular structures were generated using CYLview program.[6]

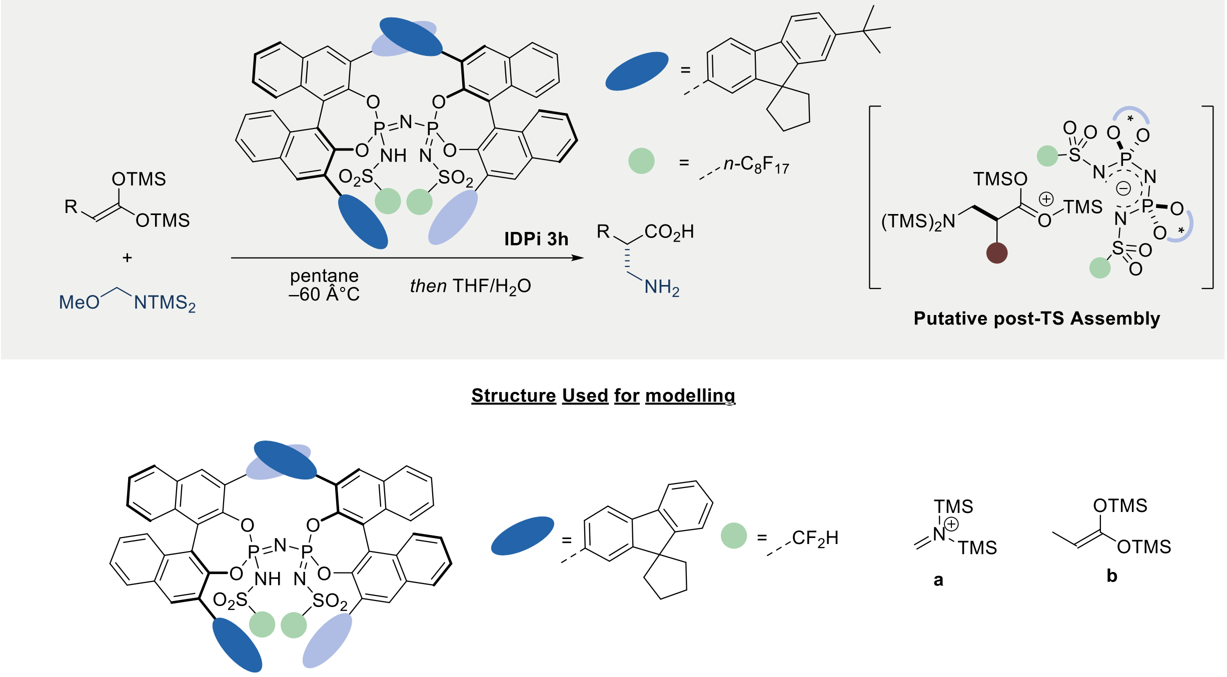

Figure S1. Summary of the overall Computational approaches undertaken for this work.

11. 2. Analysis

Based on our mechanistic hypothesis and previous analogous reports,[7] we anticipated the addition of bis-SKA to iminium IDPi ion-pair to be the stereoselectivity-determining step. Optimization of the methylene iminium ion - IDPi counterion complex highlights the importance of CH..O interactions in the recognition of the cationic substrate within the catalyst cavity (Figure S2).[8] Furthermore, given the close proximity of the silyl group attached to the iminium and the oxygen atoms in IDPi catalyst (3.2 A), favorable Si..O interactions may also contribute.[7] Together, these not only provide considerable stabilization to the iminium ion, but also enforce its distinct orientation within the catalyst cavity. Notably, this situation confines suitable trajectories of the nucleophilic attack. Modeling possible approaches suggests the nucleophilic addition of the bis-SKA onto the iminium ion - IDPi counterion complex to proceed via a *re facial attack*, which alleviates steric strain between the bis-SKA substituent and the cylopentyl ring of the catalyst (Figure S3). Consistent with this model, removal of the cylopentyl ring of the catalyst substituent (responsible for the destabilization of the $TS_{\mathbf{Minor}}$) leads to a significant reduction of stereoselectivity (see control experiments in the paragraph of HPLC Traces of the Products), consistent with this sterics-based stereoselectivity model.

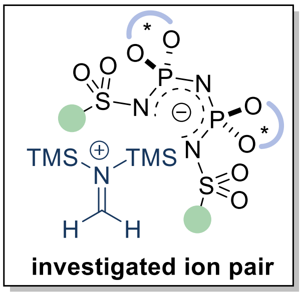

(I) Front View

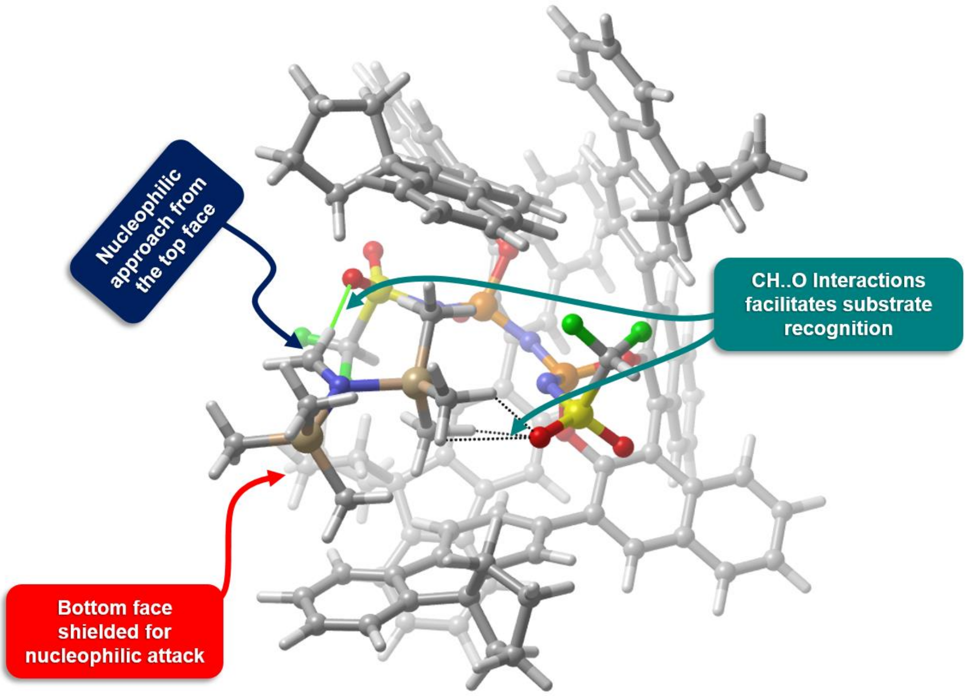

(II) Side View

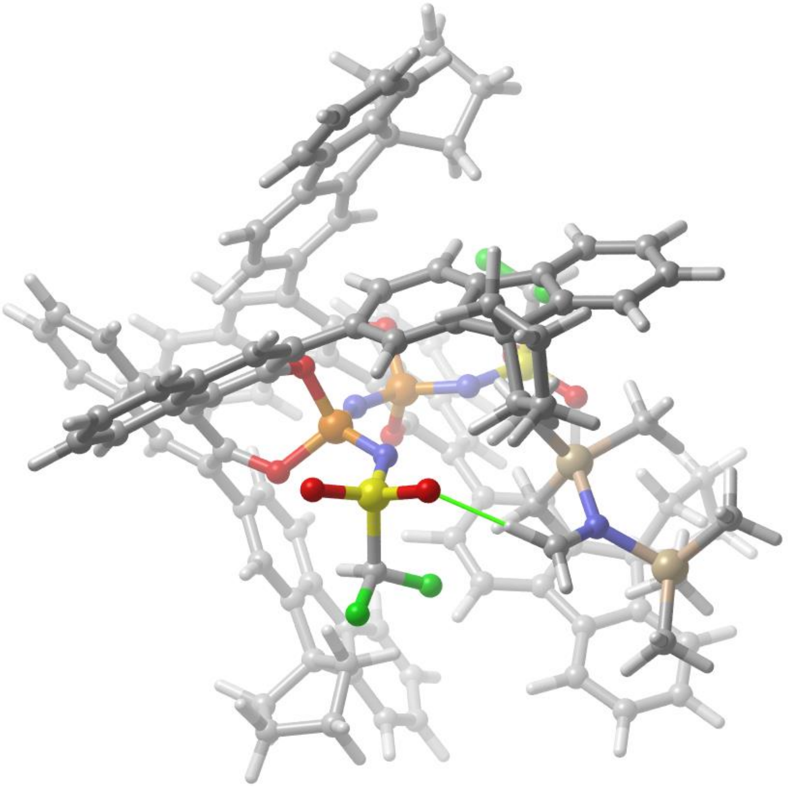

Figure S2. Optimized structure of the iminium-IDPi ion pair at the **PBE-D3/def2-SVP** level. (a) CH..O interactions act as recognition elements and assist the ion pairing. (b) Nucleophile can only approach from one side of the iminium ion due to steric shielding of its bottom face.

- *Proposed TS Model corresponding to the bis-SKA addition to the iminium IDPi ion pair.*

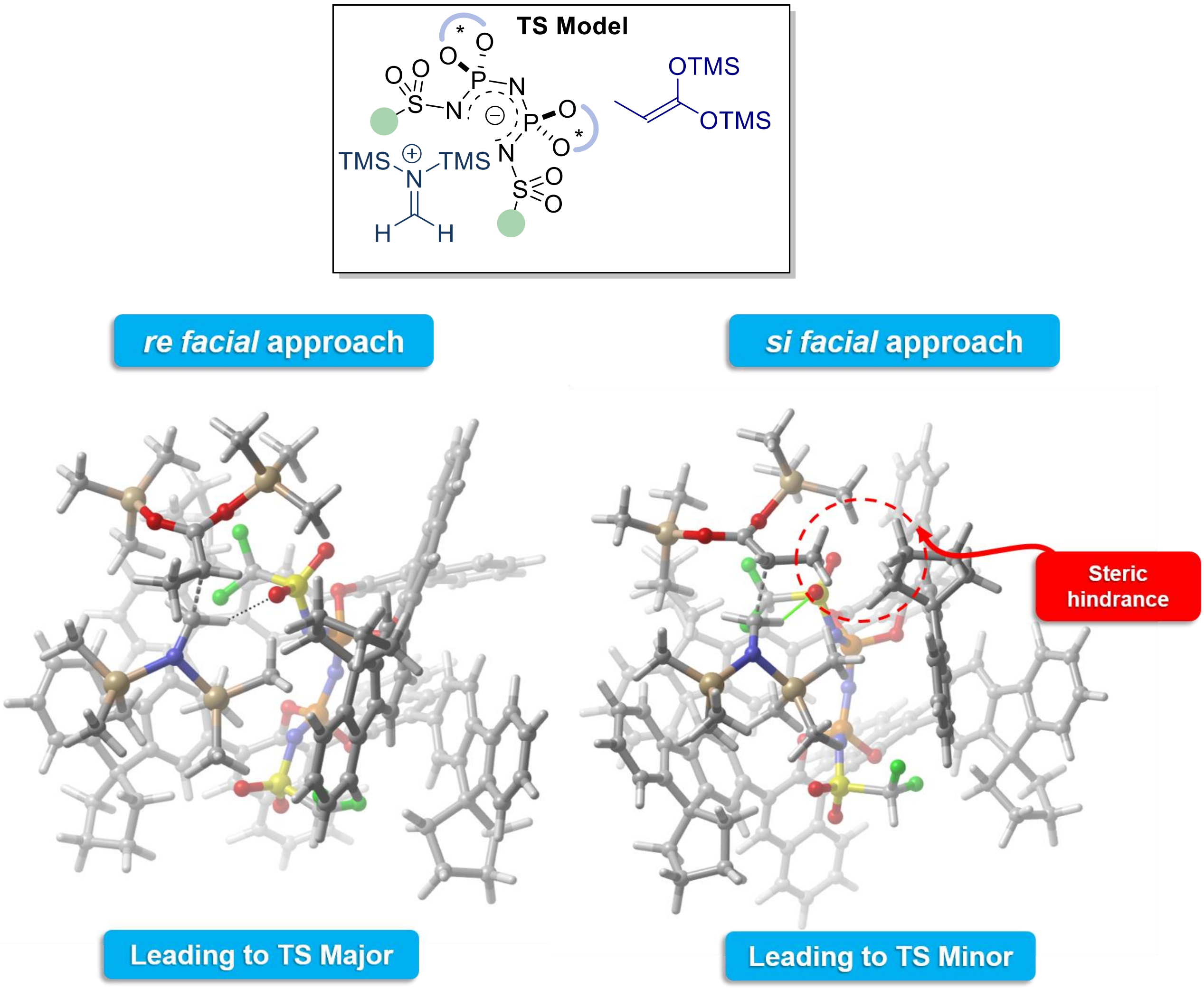

Figure S3. Putative TS arrangements to rationalize the observed selectivity.

11. 3. Optimized Cartesian Coordinates PBE-D3/def2-SVP

Lowest energy structure for Methylene iminium ion-IDPi anion pair

16	-7.238830000	30.959880000	6.439910000
16	-12.966870000	33.976780000	4.322170000
15	-7.651250000	33.313710000	4.819540000
15	-10.228250000	34.864630000	4.356490000
7	-8.744930000	34.359100000	4.396840000
7	-8.150380000	32.098680000	5.741410000
7	-11.391490000	33.794470000	4.648780000
8	-6.370880000	34.070380000	5.562900000
8	-6.973980000	32.863380000	3.380060000
8	-5.815930000	30.912510000	6.022730000
8	-7.999340000	29.682860000	6.505420000
8	-10.201080000	36.164220000	5.370470000
8	-10.581190000	35.625570000	2.915760000

8	-13.758510000	33.127360000	5.262830000
8	-13.445020000	35.353460000	4.069130000
6	-13.967010000	30.018750000	5.309610000
1	-14.934230000	29.642720000	4.920070000
14	-13.431410000	27.345280000	5.718560000
6	-7.181830000	31.550320000	8.211050000
6	-13.037430000	33.142860000	2.648000000
6	-11.893100000	26.662190000	4.915670000
6	-14.949350000	27.094940000	4.638190000
6	-13.761900000	26.818720000	7.489070000
6	-5.727760000	35.067050000	4.835140000
6	-5.681870000	32.374460000	3.302380000
6	-11.261190000	37.051760000	5.375990000
6	-9.913290000	36.811580000	2.622510000
9	-8.434150000	31.616710000	8.736470000
9	-6.630800000	32.784070000	8.235630000
9	-12.806810000	31.800770000	2.766620000
1	-6.560780000	30.830860000	8.790930000
9	-14.250900000	33.311080000	2.081560000
1	-12.242280000	33.613430000	2.031600000
1	-11.967830000	25.629880000	4.527740000
1	-11.654950000	27.282590000	4.029040000
1	-11.019490000	26.706890000	5.589690000
1	-15.863120000	27.575320000	5.042600000
1	-15.151170000	26.004150000	4.592670000
1	-14.784330000	27.445890000	3.599670000
6	-4.897230000	34.692890000	3.774040000
6	-4.392600000	35.719560000	2.897530000
6	-3.664660000	35.427290000	1.705000000
1	-3.437700000	34.380160000	1.462300000
6	-3.266340000	36.440110000	0.843630000
1	-2.719220000	36.187280000	-0.077070000
6	-3.565470000	37.797360000	1.136120000
1	-3.242590000	38.591470000	0.445960000
6	-4.270970000	38.115640000	2.286430000
1	-4.524010000	39.161450000	2.520850000
6	-4.708730000	37.097140000	3.184510000
6	-5.465940000	37.414230000	4.344150000
1	-5.657030000	38.472700000	4.577210000
6	-5.982320000	36.432570000	5.186090000
6	-4.619030000	33.251910000	3.518800000
6	-3.279010000	32.726980000	3.507180000
6	-2.137630000	33.519300000	3.827670000
1	-2.281750000	34.572740000	4.104950000

6	-0.860890000	32.975650000	3.804610000
1	0.004250000	33.605440000	4.061790000
6	-0.663040000	31.612770000	3.459100000
1	0.354240000	31.193380000	3.439980000
6	-1.753970000	30.811030000	3.160890000
1	-1.614990000	29.748210000	2.908030000
6	-3.080560000	31.334290000	3.187300000
6	-4.206530000	30.510180000	2.913830000
1	-4.037900000	29.455390000	2.647960000
6	-5.509960000	30.992320000	2.975890000
6	-6.785100000	36.817490000	6.374090000
6	-6.537180000	36.274210000	7.660170000
1	-5.750520000	35.515040000	7.784880000
6	-7.299650000	36.704330000	8.748980000
6	-7.186730000	36.326990000	10.223550000
6	-5.880100000	36.891440000	10.863190000
1	-5.042550000	36.677160000	10.166530000
1	-5.924110000	37.990610000	10.994810000
6	-7.043030000	34.818640000	10.549000000
1	-8.011390000	34.284510000	10.484030000
1	-6.376920000	34.360930000	9.794180000
6	-8.446280000	36.971010000	10.805210000
6	-9.023320000	36.841280000	12.074610000
1	-8.550000000	36.229670000	12.855850000
6	-10.254240000	37.471540000	12.340650000
1	-10.723410000	37.347720000	13.328600000
6	-10.901850000	38.233220000	11.352220000
1	-11.884590000	38.680890000	11.566050000
6	-10.319390000	38.393520000	10.085310000
1	-10.834760000	38.967470000	9.300010000
6	-9.094370000	37.761360000	9.818470000
6	-8.346800000	37.644870000	8.563330000
6	-8.576190000	38.214900000	7.301800000
1	-9.391010000	38.937700000	7.144140000
6	-7.786180000	37.804160000	6.222520000
1	-7.979750000	38.204430000	5.219130000
6	-6.672220000	30.107610000	2.724230000
6	-6.698650000	28.800750000	3.267030000
1	-5.896330000	28.489670000	3.953200000
6	-7.760760000	27.947680000	2.962500000
6	-8.007420000	26.523360000	3.446520000
6	-8.115050000	26.438000000	5.018960000
1	-8.218940000	27.442040000	5.476240000
1	-9.013500000	25.843620000	5.283930000

6	-6.833660000	25.541510000	3.091090000
1	-7.049270000	24.954950000	2.176700000
1	-5.923860000	26.141300000	2.883280000
6	-9.318480000	26.183880000	2.739510000
6	-10.047810000	24.989200000	2.737910000
1	-9.689410000	24.110890000	3.298430000
6	-11.245040000	24.911880000	1.998360000
1	-11.815170000	23.970270000	1.980060000
6	-11.726940000	26.032450000	1.295420000
1	-12.673910000	25.960660000	0.738390000
6	-11.002000000	27.236630000	1.289270000
1	-11.376650000	28.119670000	0.746410000
6	-9.784600000	27.298090000	1.989790000
6	-8.811970000	28.385870000	2.120590000
6	-8.801670000	29.682370000	1.584870000
1	-9.599980000	30.027090000	0.912140000
6	-7.741060000	30.536260000	1.899790000
1	-7.724950000	31.551620000	1.488290000
6	-11.347700000	37.965430000	4.325850000
6	-12.495030000	38.829550000	4.268290000
6	-12.745570000	39.709040000	3.174950000
1	-12.039690000	39.724100000	2.332720000
6	-13.864830000	40.529410000	3.159680000
1	-14.040940000	41.196430000	2.302170000
6	-14.788400000	40.507990000	4.238000000
1	-15.670600000	41.165600000	4.217800000
6	-14.585420000	39.646350000	5.304780000
1	-15.305930000	39.607210000	6.136740000
6	-13.453090000	38.779620000	5.347280000
6	-13.269850000	37.859240000	6.416050000
1	-14.008650000	37.845460000	7.232000000
6	-12.203390000	36.964860000	6.449330000
6	-10.257450000	37.983690000	3.307210000
6	-9.453030000	39.162800000	3.091300000
6	-9.648510000	40.376620000	3.818310000
1	-10.460220000	40.431960000	4.555760000
6	-8.816920000	41.470710000	3.624710000
1	-8.984870000	42.389880000	4.206040000
6	-7.747670000	41.411450000	2.692450000
1	-7.096220000	42.286410000	2.547140000
6	-7.522990000	40.245790000	1.976890000
1	-6.690980000	40.177510000	1.258730000
6	-8.353540000	39.099930000	2.158010000
6	-8.101230000	37.894560000	1.448360000

1	-7.275560000	37.868230000	0.721080000
6	-8.861690000	36.748400000	1.652540000
6	-12.069690000	35.922170000	7.495120000
6	-13.220190000	35.246210000	7.967050000
1	-14.202120000	35.480970000	7.528030000
6	-13.086500000	34.237270000	8.920550000
6	-14.170530000	33.368790000	9.539010000
6	-15.092290000	32.624550000	8.524800000
1	-15.158180000	33.219570000	7.592490000
1	-14.644830000	31.652700000	8.235980000
6	-15.194420000	34.212070000	10.353980000
1	-14.761050000	34.599710000	11.298210000
6	-13.348030000	32.407360000	10.399850000
6	-13.749080000	31.278630000	11.125450000
1	-14.807140000	30.981000000	11.173560000
6	-12.777300000	30.496170000	11.781010000
1	-13.089900000	29.609360000	12.353540000
6	-11.412830000	30.827720000	11.697930000
1	-10.665840000	30.195700000	12.202330000
6	-10.995880000	31.948300000	10.961150000
1	-9.925760000	32.179360000	10.847670000
6	-11.966330000	32.736690000	10.320720000
6	-11.810990000	33.881370000	9.420750000
6	-10.663840000	34.546830000	8.966750000
1	-9.662290000	34.267400000	9.322170000
6	-10.801750000	35.557070000	8.012720000
1	-9.907450000	36.075560000	7.656030000
6	-8.576200000	35.498240000	0.905150000
6	-9.609050000	34.795770000	0.237230000
1	-10.638180000	35.185060000	0.277320000
6	-9.310050000	33.629170000	-0.465880000
6	-10.252160000	32.718430000	-1.248090000
6	-10.872540000	33.407070000	-2.531160000
1	-10.473660000	34.431020000	-2.664530000
1	-10.579510000	32.827480000	-3.428970000
6	-11.505960000	32.287590000	-0.428290000
1	-11.331690000	31.387560000	0.192980000
1	-11.746680000	33.116460000	0.265460000
6	-9.321050000	31.565070000	-1.614360000
6	-9.618060000	30.355100000	-2.251220000
1	-10.642760000	30.127560000	-2.583800000
6	-8.588820000	29.416360000	-2.457940000
1	-8.817560000	28.456050000	-2.944570000
6	-7.273200000	29.692100000	-2.043750000

1	-6.482350000	28.944470000	-2.206710000
6	-6.964440000	30.907070000	-1.412520000
1	-5.940060000	31.113130000	-1.066170000
6	-7.991970000	31.839660000	-1.197070000
6	-7.982740000	33.128100000	-0.494130000
6	-6.948680000	33.827280000	0.148510000
1	-5.915960000	33.446230000	0.144920000
6	-7.252840000	35.008750000	0.837420000
1	-6.459270000	35.540840000	1.378440000
1	-15.472310000	35.093160000	9.736360000
7	-13.102190000	29.191430000	5.792910000
1	-13.814560000	31.121080000	5.267160000
1	-14.666530000	27.327360000	7.879990000
1	-13.939670000	25.724800000	7.537900000
1	-12.909680000	27.056040000	8.153090000
14	-11.352860000	29.870330000	6.314380000
6	-11.713560000	31.511490000	7.093200000
1	-12.092410000	31.397980000	8.125120000
1	-10.783910000	32.112980000	7.112630000
1	-12.447290000	32.057910000	6.472310000
6	-10.668680000	28.690680000	7.606210000
1	-9.709580000	29.155550000	7.911950000
1	-11.333530000	28.662100000	8.493350000
1	-10.436690000	27.662620000	7.274440000
6	-10.528500000	29.965530000	4.647420000
1	-10.402770000	28.983760000	4.160820000
1	-11.110610000	30.637980000	3.988380000
1	-9.526310000	30.407420000	4.800680000
6	-16.480740000	32.482820000	9.208210000
1	-17.274530000	32.887140000	8.547880000
1	-16.746550000	31.422500000	9.396910000
6	-16.404270000	33.288340000	10.531340000
1	-16.235890000	32.610750000	11.392990000
1	-17.336730000	33.847120000	10.748850000
6	-6.854780000	25.691120000	5.481010000
1	-6.976990000	25.218200000	6.476540000
1	-5.999780000	26.397050000	5.557660000
6	-6.604510000	24.690400000	4.347310000
1	-5.599800000	24.221610000	4.373510000
1	-7.350990000	23.867270000	4.400760000
6	-12.654840000	32.174730000	-1.433520000
1	-13.648820000	32.186440000	-0.941730000
1	-12.582450000	31.230000000	-2.014350000
6	-12.402750000	33.385190000	-2.342440000

1	-12.736330000	34.307210000	-1.819390000
1	-12.952360000	33.342590000	-3.304210000
6	-5.703100000	36.113900000	12.179610000
1	-4.632530000	36.007630000	12.445880000
1	-6.182950000	36.660330000	13.016720000
6	-6.397030000	34.741410000	11.951030000
1	-5.682870000	33.895800000	12.013660000
1	-7.160920000	34.551330000	12.732030000

11. 4 References

(1) Neese, F. Wiley Interdiscip. *Rev.: Comput. Mol. Sci.* **2011**, *2*, 73–78.

(2) Zhang, Y.; Yang, W. Comment on "Generalized gradient approximation made simple". *Phys. Rev. Lett.* **1998**, *80*, 890–890.

(3) (a) Grimme, S.; Antony, J.; Ehrlich, S.; Krieg, H. A consistent and accurate *ab initio* parametrization of density functional dispersion correction (DFT-D) for the 94 elements H-Pu. *J. Chem. Phys.* **2010**, *132*, 154104. (b) Grimme, S.; Ehrlich, S.; Goerigk, L. Effect of the damping function in dispersion corrected density functional theory. *J. Comput. Chem.* **2011**, *32*, 1456–1465.

(4) Weigend, F.; Ahlrichs, R. Balanced basis sets of split valence, triple zeta valence and quadruple zeta valence quality for H to Rn: Design and assessment of accuracy. *Phys. Chem. Chem. Phys.* **2005**, *7*, 3297–3305.

(5) Zhao, Y.; Truhlar, D. G. The M06 suite of density functionals for main group thermochemistry, thermochemical kinetics, noncovalent interactions, excited states, and transition elements: two new functionals and systematic testing of four M06-class functionals and 12 other functionals. *Theor. Chem. Acc.* **2008**, *120*, 215–241.

(6) Legault, C. Y.: CYLview, 1.0b; Université de Sherbrooke, 2009 (http://www.cylview.org).

(7) Schreyer, L.; Kaib, P. S. J.; Wakchaure, V. N.; Obradors, C.; Properzi, R.; Lee, S.; List, B. Confined acids catalyze asymmetric single aldolizations of acetaldehyde enolates. *Science* **2018**, *362*, 216–219.

(8) (a) Belding, L.; Taimoory, S. M.; Dudding, T. Mirroring enzymes: The role of hydrogen bonding in an asymmetric organocatalyzed aza-Henry reaction—a DFT study. *ACS Catal.* **2015**, *5*, 343–349. (b) Johnston, R. C.; Cheong, P. H.-Y. C–H···O non-classical hydrogen bonding in the stereomechanics of organic transformations: theory and recognition. *Org. Biomol. Chem.* **2013**, *11*, 5057–5064.
